# Phosphate-enabled mechanochemical PFAS destruction for fluoride reuse

**DOI:** 10.1038/s41586-025-08698-5

**Published:** 2025-03-26

**Authors:** Long Yang, Zijun Chen, Christopher A. Goult, Thomas Schlatzer, Robert S. Paton, Véronique Gouverneur

**Affiliations:** 1 Chemistry Research Laboratory, University of Oxford, Oxford, UK; 2 Department of Chemistry, Colorado State University, Fort Collins, CO USA

**Keywords:** Organic chemistry, Chemical synthesis, Polymer chemistry

## Abstract

Perfluoroalkyl and polyfluoroalkyl substances (PFASs) are persistent, bioaccumulative and anthropogenic pollutants that have attracted the attention of the public and private sectors because of their adverse impact on human health^1^. Although various technologies have been deployed to degrade PFASs with a focus on non-polymeric functionalized compounds (perfluorooctanoic acid and perfluorooctanesulfonic acid)^2–4^, a general PFAS destruction method coupled with fluorine recovery for upcycling is highly desirable. Here we disclose a protocol that converts multiple classes of PFAS, including the fluoroplastics polytetrafluoroethylene and polyvinylidene fluoride, into high-value fluorochemicals. To achieve this, PFASs were reacted with potassium phosphate salts under solvent-free mechanochemical conditions, a mineralization process enabling fluorine recovery as KF and K_2_PO_3_F for fluorination chemistry. The phosphate salts can be recovered for reuse, implying no detrimental impact on the phosphorus cycle. Therefore, PFASs are not only destructible but can now contribute to a sustainable circular fluorine economy.

## Abstract (web summary)

This study highlights a protocol that converts various perfluoroalkyl and polyfluoroalkyl substances (PFASs), including fluoroplastics, into valuable fluorochemicals through a solvent-free mechanochemical process, thereby enabling fluorine recovery and contributing to a sustainable circular fluorine economy.

## Main

**Fig. 1:**
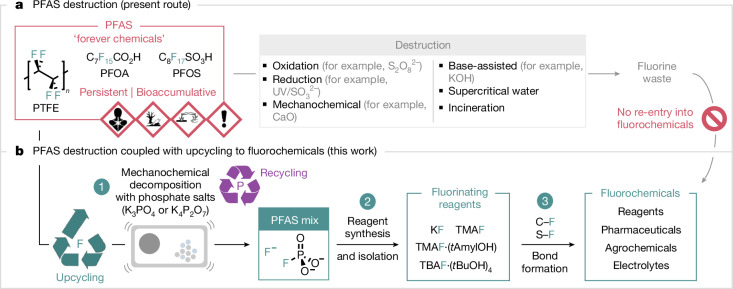
Synthesis of fluorochemicals from PFASs. **a**, Selection of existing routes for PFAS degradation. **b**, PFAS mineralization coupled with upcycling into essential fluorochemicals (this study). Credits: upcycling symbol in **b** adapted from https://thenounproject.com/icon/upcycle-18167/, under a PDM 1.0 licence (https://creativecommons.org/publicdomain/mark/1.0/); recycling symbol in **b** reproduced from https://revvitysignals.com/products/research/chemdraw.

Since the 1940s, anthropogenic perfluoroalkyl and polyfluoroalkyl substances (PFASs) have been produced for applications such as textile impregnation, firefighting foams, food packaging and cookware materials. Medical uses include implanted devices, orthopaedic components, catheters, pacemakers and surgical tools^5^. Structurally, PFASs feature multiple carbon–fluorine (C–F) bonds that account for their unique and valuable properties but are also responsible for their resistance to biological or chemical degradation^1^. Today, environmental persistence and bioaccumulation have resulted in global PFAS contamination in drinking water, livestock and agricultural products, with evidence of a negative impact on human health upon chronic exposure^6,7^. This state of play requires immediate action, including the development of PFAS removal approaches^2–4^ and responsible management of PFAS-contaminated waste streams^8,9^. The PFAS degradation methods reported to date include chemical- and photochemical-initiated oxidation and reduction processes^10–13^, mechanical^14–24^ and base-assisted destruction, including low-temperature mineralization^25,26^, and incineration^27^ among other techniques^28,29^ (Fig. 1a).

With the knowledge that all fluorochemicals are produced from fluorite^30,31^, a naturally occurring mineral currently categorized as critical by the European Union and other countries, the availability of a mild method that destroys PFASs with recovery of the fluorine content for upcycling would represent a paradigm shift in PFAS management. Such an upcycling approach would contribute to diminishing the root cause of impending global challenges, such as resource shortages and uncertain supply chains linked to geopolitical turmoil. Here we disclose an operationally simple solution entailing the reaction of various PFAS classes with potassium phosphate salts applying mechanical energy. The process enables close to quantitative recovery of PFAS fluorine content as KF and K_2_PO_3_F. Because we demonstrated that K_2_PO_3_F can be converted into KF or tetraalkylammonium fluorides, PFASs as diverse as polytetrafluoroethylene (PTFE; Teflon), polyvinylidene fluoride (PVDF), perfluorooctanoic acid (PFOA) or perfluorooctanesulfonic acid (PFOS) are not only destructible but can also serve as a viable fluorine source for upcycling into critically needed fluorochemicals for life and material sciences. This process enables the recovery of phosphate salts for reuse, which is an advantage in the era of phosphorus insecurity for fertilizer production (Fig. 1b).

## Results and discussion

**Fig. 2:**
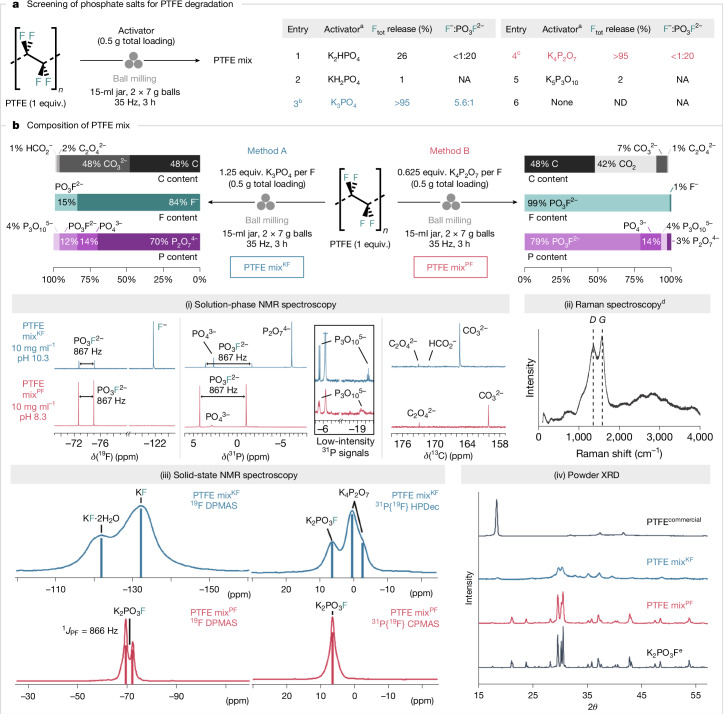
Phosphate-enabled mechanochemical mineralization of PTFE. **a**, Screening of various phosphate salts as activators for PTFE. The total yield of released fluoride (both F^−^ and PO_3_F^2−^) and their ratio were determined by quantitative ^19^F NMR spectroscopy (in 10% D_2_O in H_2_O using NaOTf as an internal standard). **b**, Identification of the C/F/P contents of PTFE mix by quantitative ^13^C/^19^F/^31^P NMR spectroscopy (in 10% D_2_O in H_2_O using KOAc or NaOTf as an internal standard) and Raman spectroscopy of water-insoluble black residue after aqueous extraction. Further analysis of PTFE mix by solid-state ^19^F/^31^P NMR spectroscopy and powder X-ray diffraction (XRD). ^a^Activator stoichiometry was kept constant at 1.25 equiv. P per F. ^b^Reaction was also performed in a 15-ml zirconia jar with 2 × 6 g zirconia balls affording 98% F_tot_ release (F^−^:PO_3_F^2−^ = 5.5:1). ^c^Reaction was also performed in a 15-ml zirconia jar with 2 × 6 g zirconia balls affording 99% F_tot_ release (F^−^:PO_3_F^2−^ <1:20). ^d^The observed bands are denoted as *D* (disordered carbon) and *G* (graphitic carbon). ^e^Prepared mechanochemically from KF and KPO_3_. NA, not applicable; ND, not detected.

An observation made in the course of our study on the synthesis of fluorochemicals from fluorspar (CaF_2_) served as a starting point of investigation^31^. We noted that ball milling CaF_2_ with a phosphate salt (K_2_HPO_4_) in a stainless-steel jar with sealing rings made of PTFE (Teflon) instead of rubber gave higher yields of K_3_(HPO_4_)F and K_2-*x*_Ca_*y*_(PO_3_F)_*a*_(PO_4_)_*b*_, a new reagent for fluorination (Supplementary Information). This result suggests that fluoride leached from PTFE under these conditions, prompting the use of PTFE-free systems for CaF_2_ chemistry. This outcome was unexpected because C–F bond cleavage is mechanistically distinct from an ion exchange process. Methods for repurposing fluoroplastics, such as PTFE, are limited^12,32–34^ and involve harsh reaction conditions^35^; therefore, further investigation ensued (Fig. 2a). Ball milling PTFE with K_3_PO_4_ (1.25 equivalents (equiv.) per F) at 35 Hz for 3 h in a steel milling jar with a rubber sealing ring gave a solid material (PTFE mix^KF^) for which the water-soluble fraction was analysed by ^19^F nuclear magnetic resonance (NMR) spectroscopy (10% D_2_O in H_2_O). Signals at −120.6 and −73.2 ppm (^1^*J*_PF_ = 867 Hz) ascribed to F^−^ (84%) and PO_3_F^2−^ (15%), respectively, indicated near-quantitative fluorine recovery. Alternative phosphate salts, including K_2_HPO_4_, KH_2_PO_4_ and K_5_P_3_O_10_, were less effective, but K_4_P_2_O_7_ stood out with the formation of a distinct mixture (PTFE mix^PF^) for which ^19^F NMR spectroscopy analysis (10% D_2_O in H_2_O) showed predominantly P–F bond formation (99% PO_3_F^2−^; *δ*_F_ = −73.7; ^1^*J*_PF_ = 867 Hz) and only trace amounts of F^−^ (less than 1%; *δ*_F_ = −120.1). Replacing the steel components (milling jar and balls) with zirconia gave close to identical results, and a control experiment confirmed that PTFE degradation is induced by the phosphate salt under the reaction conditions and is not linked to metal leaching from the steel components. For comparison, KOH (1.25 equiv. per F), which is known to decompose PFOA and PFOS by applying mechanical energy^14,16^, was found to be ineffective for releasing fluoride from PTFE (less than 10%) (Supplementary Information). These results prompted an in-depth investigation because potassium phosphate salts may offer a general and direct route to fluorinating reagents, such as KF, from PTFE and other PFASs, thereby contributing to a circular economy for the fluorochemical sector.

Further analysis gave useful information on the composition of PTFE mixes (Fig. 2b and Supplementary Information). Quantitative ^31^P NMR spectroscopy (10% D_2_O in H_2_O) indicated that different ratios of PO_3_F^2−^, PO_4_^3−^, P_2_O_7_^4−^ and P_3_O_10_^5−^ were present in the PTFE mix^KF^ and PTFE mix^PF^. For the PTFE mix^KF^, P_2_O_7_^4−^ (*δ*_P_ = −6.3; 70% of total phosphorus (P_tot_)) was the predominant phosphorus species, followed by PO_4_^3−^ (*δ*_P_ = 2.7; 14% P_tot_), PO_3_F^2−^ (*δ*_P_ = 1.0; ^1^*J*_PF_ = 867 Hz; 12% P_tot_) and P_3_O_10_^5−^ (*δ*_P_ = −5.5; −20.3; ^2^*J*_PP_ = 20 Hz; 4% P_tot_). In contrast, PO_3_F^2−^ (*δ*_P_ = 1.5; ^1^*J*_PF_ = 867 Hz; 79% P_tot_) was the major species in the PTFE mix^PF^, followed by PO_4_^3−^ (*δ*_P_ = 2.9; 14% P_tot_), P_3_O_10_^5−^ (*δ*_P_ = −5.5; −19.5; ^2^*J*_PP_ = 20 Hz; 4% P_tot_) and P_2_O_7_^4−^ (*δ*_P_ = −6.3; 3% P_tot_). Quantitative ^13^C NMR spectroscopy gave insights into the fate of the carbon skeleton. The water-soluble fraction of the PTFE mix^KF^ consisted mainly of CO_3_^2−^ (*δ*_C_ = 165.5; 48% C_tot_), with trace amounts of C_2_O_4_^2−^ (*δ*_C_ = 173.0; 2% C_tot_) and HCO_2_^−^ (*δ*_C_ = 171.1; 1% C_tot_). For PTFE mix^PF^, CO_3_^2−^ (*δ*_C_ = 160.2; 7% C_tot_) was predominant, followed by C_2_O_4_^2−^ (*δ*_C_ = 173.0; 1% C_tot_); HCO_2_^−^ was not detected.

Taken together, these species accounted for only a fraction of the total carbon content of PTFE, prompting gas capture analysis. A significant quantity of CO_2_ (42% C_tot_) was released in the case of K_4_P_2_O_7_-induced PTFE degradation, but no CO_2_ was detected with K_3_PO_4_, which is an advantage in the era of decarbonization (Supplementary Information). The water-insoluble black solid isolated from the PTFE mix^KF^ and PTFE mix^PF^ was analysed using Raman spectroscopy. Bands at 1,355 and 1,579 cm^−1^ were observed, consistent with the disordered and graphitic bands of carbon (48% C_tot_ for both samples)^36^. Solid-state ^19^F, ^19^F{^31^P} and ^31^P{^19^F} NMR spectroscopy showed that the PTFE mix^KF^ contains KF, KF∙2H_2_O, K_2_PO_3_F and K_4_P_2_O_7_, whereas the PTFE mix^PF^ is mainly composed of K_2_PO_3_F. The presence of K_2_PO_3_F in the PTFE mix^KF^ and PTFE mix^PF^ with no residual PTFE was confirmed by powder X-ray diffraction analysis.

**Extended Data Fig. 1:**
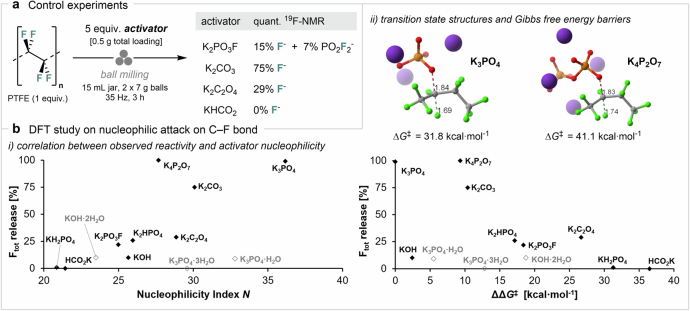
Control experiments and DFT study. **a**, Control experiments employing by-products as activators for PTFE. Yields of F^−^ and ${{\rm{PO}}}_{2}{{\rm{F}}}_{2}^{-}$ were determined by quantitative ^19^F NMR spectroscopy (in 10% D_2_O in H_2_O using NaOTf as an internal standard). **b**, DFT computational studies showing a correlation between observed reactivity and (i) oxyanion nucleophilicity parameter as well as an inverse correlation with (ii) S_N_2 barrier height; hydrated salts are shown in grey.

Control experiments were performed to further understand this solvent-free mechanochemical process (Extended Data Fig. 1a). Both K_3_PO_4_ and K_4_P_2_O_7_ were subjected independently to ball milling in the absence of PTFE. No reactivity was observed for K_3_PO_4_, but minor quantities of K_3_PO_4_ (3%) and K_5_P_3_O_10_ (2%) were formed upon milling K_4_P_2_O_7_ (Supplementary Information). We also investigated the ability of the PTFE degradation products K_2_CO_3_, K_2_C_2_O_4_, K_2_PO_3_F and KHCO_2_ to decompose PTFE under otherwise similar ball milling conditions, demonstrating that K_2_CO_3_ was the most effective, yielding KF in 75%. These results indicate a complex mechanistic regime involving multiple oxyanion species. Further experiments demonstrated that ball milling K_2_PO_3_F with K_3_PO_4_ gave quantitative conversion to KF along with K_4_P_2_O_7_; when ball milled with K_4_P_2_O_7_, K_2_PO_3_F was mainly recovered, with 13% of KF being formed (Supplementary Information).

To gain insight into the ability of different oxyanions to decompose PTFE, we studied their global reactivity descriptors with *ω*B97xD/6-311++G(2d,2p) density functional theory (DFT) calculations (Extended Data Fig. 1b)^37^. Specifically, the global nucleophilicity index *N*^38–41^ was calculated and compared against the total yield of mineralized products KF and K_2_PO_3_F. A positive correlation suggests nucleophilic behaviour of the oxyanions in the milling process (Extended Data Fig. 1b(i)). Alternative theoretical formulations of the nucleophilicity indices yielded consistent results (Supplementary Information). A putative nucleophilic substitution reaction between various oxyanions and the model PFAS perfluorobutane was therefore examined next (Extended Data Fig. 1b(ii)). The reaction with K_3_PO_4_ favoured an S_N_2 transition structure at the internal carbon, with the lowest Gibbs free energy barrier (*ΔG*^‡^ = 31.8 kcal mol^−1^). K_4_P_2_O_7_ and K_2_CO_3_ gave the third and fourth lowest activation barriers (*ΔΔG*^‡^ = 9.3 and 10.4 kcal mol^−1^ versus K_3_PO_4_, respectively), demonstrating kinetic feasibility and the superiority of these three oxyanions for PFAS degradation. The ordering of computed activation barriers correlated with the experimental yields. For benchmarking, it is noteworthy that the weakest homolytic bond dissociation of perfluorobutane is 103 kcal mol^−1^ (ref. ^42^), which is comparable to the average C–C bond energy of 90 kcal mol^−1^ in PTFE^43,44^. Because we experimentally determined the composition of the PTFE mix^KF^ (Fig. 2b), the standard enthalpy change (*ΔH*^o^) of an idealized process ([C_2_F_4_]_*n*_ and K_3_PO_4_ leading to C, K_2_CO_3_, KF and K_4_P_2_O_7_; Supplementary Information) was determined as −143.5 kcal mol^−1^. This compared to a value of −107.9 kcal mol^−1^ for the decomposition of PTFE mix^PF^ ([C_2_F_4_]_*n*_ and K_4_P_2_O_7_ leading to C, CO_2_ and K_2_PO_3_F; Supplementary Information). The data are consistent with the favourable formation of KF (−2.95 eV per atom) and K_2_PO_3_F (−2.76 eV per atom) (Supplementary Information). Therefore, the phosphate-mediated decomposition of PTFE is enthalpically and entropically favourable.

The superiority of K_3_PO_4_ over KOH for the destruction of PTFE warrants further study of the solvation effect (that is, hydration). The putative S_N_2 reaction of perfluorobutane at an internal carbon with anhydrous KOH has the second lowest Gibbs free energy barrier (*ΔG*^‡^ = 34.3 kcal mol^−1^) of all oxyanions investigated. However, as expected, for mono- and dihydrate clusters of KOH, nucleophilic substitution is a kinetically more demanding process in comparison with anhydrous KOH (*ΔΔG*^‡^ = 8.9 and 16.3 kcal mol^−1^, respectively). Such solvation effects also impact K_3_PO_4_ but to a lesser extent (*ΔΔG*^‡^ = 5.5 kcal mol^−1^ (K_3_PO_4_∙H_2_O), 8.7 kcal mol^−1^ (K_3_PO_4_∙2H_2_O) and 12.8 kcal mol^−1^ (K_3_PO_4_∙3H_2_O)). Experimentally, inefficient PTFE decomposition was observed with KOH, irrespective of its water content (less than 10% fluoride release; Supplementary Information). By contrast, PTFE degradation was quantitative with anhydrous K_3_PO_4_, was poorly effective with K_3_PO_4_∙H_2_O (9% fluoride recovery) and did not occur with K_3_PO_4_∙3H_2_O. These data can be accounted for considering that the degradation of PTFE with KOH produces water, decreasing the hydroxide nucleophilicity through strong solvation as the reaction proceeds.

**Fig. 3:**
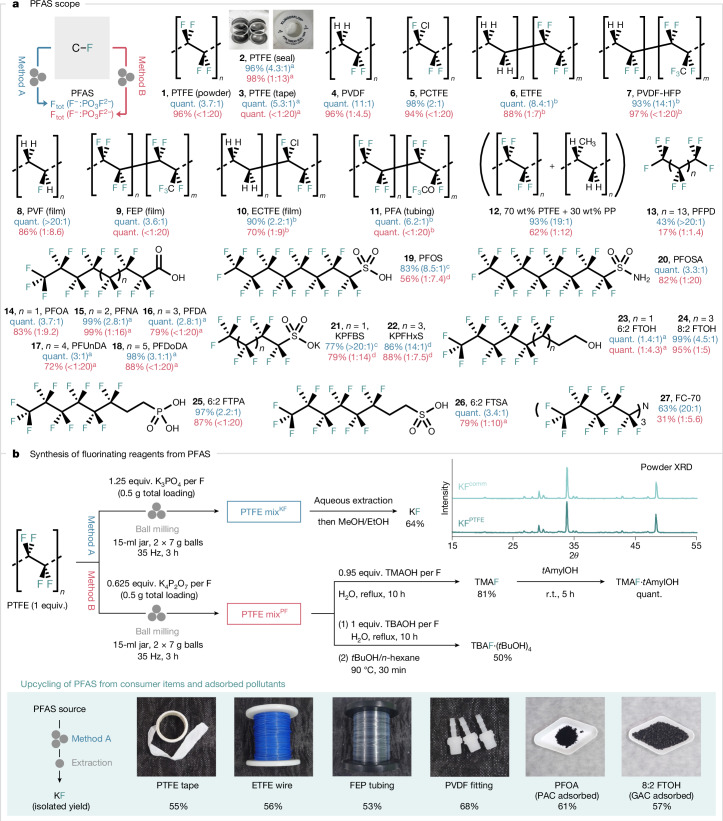
PFAS destruction and fluorinating reagents from PFAS. **a**, Scope of PFAS destruction. The total yield of released fluoride (both F^−^ and PO_3_F^2−^) and their ratio were determined by quantitative ^19^F NMR spectroscopy (in 10% D_2_O in H_2_O using NaOTf as an internal standard). Reactions were performed in triplicates, and average yields are reported. **b**, Synthesis of KF and tetraalkylammonium fluoride from PTFE and other PFAS sources under mechanochemical conditions. Yields of isolated products are reported. ^a^Reaction was performed once. ^b^The yield was calculated on the basis of the fluorine content of the copolymer as determined by elemental analysis. ^c^The amount of K_3_PO_4_ was increased to 2 equiv. per F. ^d^Reaction was carried out for 6 h. quant., quantitative; r.t., room temperature; TMAOH, tetramethylammonium hydroxide; TBAOH, tetrabutylammonium hydroxide.

Having established the optimal conditions for the formation of fluoride and fluorophosphate from PTFE, we investigated the generality of this process with PFASs other than PTFE (Fig. 3a). The methodology was applied successfully to both polymeric (**1**–**12**) and non-polymeric PFASs (**13**–**27**); these include PTFE in various forms, including consumer Teflon seal and tape (**1**–**3**), PVDF (**4**), polychlorotrifluoroethylene (PCTFE) (**5**), ethylene tetrafluoroethylene (ETFE) (**6**), poly(vinylidene fluoride-co-hexafluoropropylene) (PVDF–HFP) (**7**), polyvinyl fluoride (PVF) film (**8**), fluorinated ethylene propylene (FEP) film (**9**), ethylene-chlorotrifluoroethylene (ECTFE) film (**10**), perfluoroalkoxy alkane (PFA) tubing (**11**), a mixture of polypropylene (PP) and PTFE (**12**), perfluoropentadecane (PFPD) (**13**), PFOA (**14**), as well as long-chain derivatives (**15**–**18**), PFOS (**19**), perfluorooctanesulfonamide (PFOSA) (**20**), potassium perfluorobutanesulfonate (KPFBS) (**21**), potassium perfluorohexanesulfonate (KPFHxS) (**22**), 6:2 fluorotelomer alcohol (6:2 FTOH) (**23**), 8:2 fluorotelomer alcohol (8:2 FTOH) (**24**), 6:2 fluorotelomer phosphonic acid (6:2 FTPA) (**25**) and 6:2 fluorotelomer sulfonic acid (6:2 FTSA) (**26**). Noteworthy, the process also degraded FC-70 (**27**), which is a coolant liquid used in electronics.

**Fig. 4:**
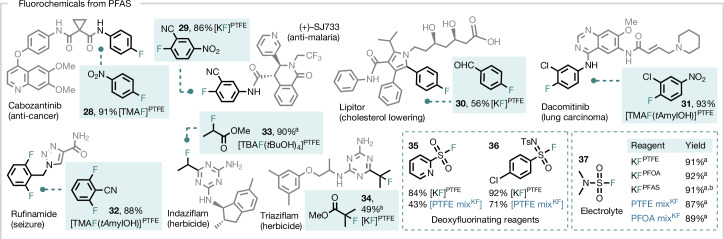
Synthesis of fluorochemicals from PFAS. Building block synthesis of high-value fluorochemicals using PFAS-derived fluorinating reagents (0.5-mmol scale unless otherwise stated). Yields of isolated products are reported. Detailed reaction conditions are stated in the Supplementary Information. ^a^Yield was determined by quantitative ^19^F NMR (in CDCl_3_ using 4-fluoroanisole as an internal standard). ^b^KF was isolated from a mixture of decomposed PFASs, including PFOS, PFOA, perfluorononanoic acid (PFNA), perfluorodecanoic acid (PFDA), PFOSA and 8:2 FTOH (Supplementary Information).

With a general method to destroy PFAS, we developed an efficient route to isolate the PTFE-derived potassium fluoride (KF) (KF^PTFE^) from the PTFE mix^KF^. The extractive purification of PTFE mix^KF^ with H_2_O and MeOH/EtOH enabled the isolation of KF^PTFE^ in 64% yield and 93% purity (calculated from PTFE; Supplementary Information). For comparison, the isolation of KF from PTFE ball milled with K_2_CO_3_ was more challenging and led to KF with lower yield (32%) and purity (55%). PTFE mix^PF^, resulting from the activation of PTFE with K_4_P_2_O_7_, served as the starting material for synthesizing tetramethylammonium fluoride (TMAF; 81%) upon treatment with tetramethylammonium hydroxide (TMAOH)^45^. TMAF was derivatized into its *tert*-amyl alcohol complex [TMAF·(*t*AmylOH)], a fluorinating reagent well documented for nucleophilic aromatic fluorination (S_N_Ar)^46^. The bench-stable reagent tetrabutylammonium tetra(*tert*-butyl alcohol) fluoride [TBAF·(*t*BuOH)_4_] (50%) was prepared in a similar manner^47^. To demonstrate the applicability of this method, KF was successfully isolated from real-world consumer items, including PTFE tape, ETFE wire, FEP tubing and PVDF fittings. Furthermore, samples of PFAS adsorbed on powdered activated carbon (PAC) or granular activated carbon (GAC) were subjected to complete destruction, yielding KF for upcycling under identical conditions (Fig. 3b). All fluorinating reagents prepared from PTFE reacted as anticipated in the synthesis of various fluorochemical classes. KF^PTFE^ performed comparably to commercial KF (KF^comm^) in substitution chemistry, leading to 2,4-dinitrofluorobenzene (Supplementary Information). It was used to prepare 2-fluoro-5-nitrobenzonitrile (**29**) and methyl 2-fluoroisobutyrate (**34**), the precursors of (+)-SJ733 (anti-malaria) and triaziflam (herbicide), respectively. The deoxyfluorinating reagents PyFluor (**35**) and SulfoxFluor (**36**), as well as the electrolyte dimethylsulfamoyl fluoride (**37**) were also prepared in good yields from KF^PTFE^ (Fig. 4). Notably, KF isolated from non-polymeric PFOA (KF^PFOA^) and other PFAS (KF^PFAS^), as well as crude mixtures (PTFE mix^KF^ and PFOA mix^KF^) were efficient fluorinating reagents for S–F bond construction (**35**–**37**). The copper-mediated fluorination^48^ of potassium 4-formylphenyltrifluoroborate (prepared from KF^PTFE^) with KF^PTFE^ afforded 4-fluorobenzaldehyde (**30**), a precursor of LIPITOR (cholesterol-lowering drug)^49^. Tetraalkylammonium fluoride reagents derived from PTFE mix^PF^ were used to access 4-fluoronitrobenzene (**28**), 2-chloro-1-fluoro-4-nitrobenzene (**31**), 2,6-difluorobenzonitrile (**32**) and methyl 2-fluoropropanoate (**33**). These fluorochemicals are the precursors of cabozantinib (anti-cancer), dacomitinib (lung carcinoma), rufinamide (seizure) and indaziflam (herbicide) (Fig. 4).

**Fig. 5:**
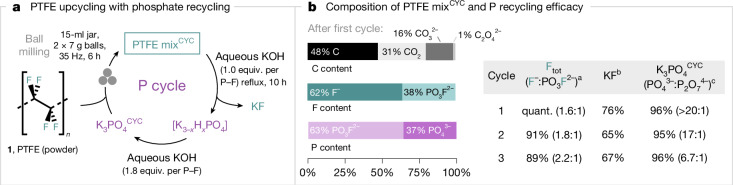
Phosphate recycling. **a**, Recovery of phosphate salts. **b**, Identification of C/F/P contents of PTFE mix^CYC^ after first cycle and efficacy of recovered phosphate salts. Ball milling conditions: PTFE (1 equiv.; 86 mg) milled with K_3_PO_4_ (0.625 equiv. per F; 457 mg) or K_3_PO_4_^CYC^ (457 mg) in a 15-ml stainless-steel milling jar, two chrome steel balls (2 × 7 g) at 35 Hz for 6 h. ^a^The total yield of released fluoride (F^−^ and PO_3_F^2−^) as well as F^−^:PO_3_F^2−^ ratio was determined by quantitative ^19^F NMR spectroscopy (in 10% D_2_O in H_2_O using NaOTf as an internal standard). ^b^Yields for isolated KF are reported. ^c^The total yield of recovered phosphate (PO_4_^3−^ and P_2_O_7_^4−^), as well as PO_4_^3−^:P_2_O_7_^4−^ ratio was determined by quantitative ^31^P NMR spectroscopy (in 10% D_2_O in H_2_O using PO(OEt)_3_ as an internal standard); recovered phosphate salts contain up to 8% carbonate.

With a technology relying on K_3_PO_4_ for PFAS destruction, we considered its possible environmental impact on the phosphorus cycle, including wastewater contamination and anthropogenic eutrophication^50^. Therefore, our next goal was to establish that PTFE can be upcycled with a process enabling both the isolation of KF and the recovery of the phosphate salt for reuse (Fig. 5a). Because K_4_P_2_O_7_ was identified as the predominant by-product of PTFE mix^KF^ and is itself an effective activator with formation of PTFE mix^PF^, the quantity of K_3_PO_4_ was adjusted for PTFE destruction coupled with KF recovery and K_3_PO_4_ recycling. Specifically, milling PTFE with K_3_PO_4_ (0.625 equiv. per F) at 35 Hz for 6 h in a steel milling jar gave a solid material (PTFE mix^CYC^) with quantitative fluorine release (F^−^:PO_3_F^2−^ = 1.6:1), and no K_4_P_2_O_7_ was observed by ^31^P NMR. PTFE mix^CYC^ was subjected to aqueous extraction to remove residual carbon black, followed by treatment with KOH (1 equiv. per P–F) at reflux for 10 h to ensure complete hydrolysis of K_2_PO_3_F. This process afforded KF isolated in 76% yield, along with K_3_PO_4_^CYC^ recovered (96% of total P content) after further treatment with aqueous KOH (1.8 equiv. per P–F). K_3_PO_4_^CYC^ is mainly composed of K_3_PO_4_ (85 wt%) along with traces of K_4_P_2_O_7_ (4 wt%). The performance of the recycled K_3_PO_4_^CYC^ was maintained over two more cycles with effective KF and K_3_PO_4_ recovery (Fig. 5b).

## Conclusion

In summary, this study presents a new approach to PFAS management, entailing a mineralization method coupled with the recovery of fluorine content for re-entry into the fluorochemical industry. This method stands out because it is applicable to all classes of PFAS, including harmful PFOA and the fluoroplastics PTFE and PVDF. The best results, both in terms of PFAS destruction and isolation of fluorinating reagents, were obtained by ball milling PFASs with potassium phosphate salts. These salts can be recovered for reuse; therefore, minimal impact is imposed on the longevity of finite phosphate rock reserves and the phosphorus cycle. Alternative oxyanions (including K_2_CO_3_) were competent but less effective, with the yield of fluoride release correlating with oxyanion nucleophilicity. This approach offers a route that not only controls the environmental impact of PFASs through highly effective mineralization, but it also contributes to the circularity of the fluorochemical industry.

## Online content

Any methods, additional references, Nature Portfolio reporting summaries, source data, extended data, supplementary information, acknowledgements, peer review information; details of author contributions and competing interests; and statements of data and code availability are available at 10.1038/s41586-025-08698-5.

## Supplementary information

Supplementary Information This file contains Supplementary Sections 1–15, including Supplementary Figs. 1–81 and Supplementary Tables 1–23 – see contents for details.

## Data Availability

All data are in the Supplementary Information or are available from the corresponding authors upon request.
