# Supplementary material for: Phosphate-enabled mechanochemical PFAS destruction for fluoride reuse
Source: Nature. 2025 Mar 26;640(8057):100–6. doi: 10.1038/s41586-025-08698-5 (PMC11964924; doi:10.1038/s41586-025-08698-5)

nature portfolio

https://doi.org/10.1038/s41586-025-08698-5

Supplementary information

Phosphate-enabled mechanochemical PFAS destruction for fluoride reuse

In the format provided by the authors and unedited

Phosphate-Enabled Mechanochemical PFAS Destruction for Fluoride Reuse

Supplementary Information

Long Yang,[1¶] Zijun Chen,[1¶] Christopher A. Goult,[1] Thomas Schlatzer,[1] Robert S. Paton,[2*] Véronique Gouverneur[1*]

[1] University of Oxford, Chemistry Research Laboratory, Oxford, OX1 3TA, UK; [2] Department of Chemistry, Colorado State University, Fort Collins, CO 80528, USA.

Correspondence to: veronique.gouverneur@chem.ox.ac.uk

1–50

Table of Contents

1. Materials and methods 3
2. Discovery of PTFE destruction 4
3. Optimisation studies on the degradation of PTFE 5
3.1 Activator screening 5
3.2 Effect of material of milling jar and balls 5
3.3 Effect of hydration state of the activator 6
3.4 Optimisation of activator stoichiometry 6
3.5 Optimisation of mechanochemical parameters 8
3.6 Optimisation of total material loading 11
3.7 Optimisation of milling time 12
3.8 PFAS degradation in solution 13
3.9 Fluoropolymer particle size effect 14
4. Composition of PTFE-mix 15
4.1 Composition of PTFE-mix^{KF} 15
4.2 Composition of PTFE-mix^{PF} 18
4.3 Powder X-ray diffraction of PTFE-mix 21
4.4 Solid-state NMR spectroscopy of PTFE-mix 22
5. Control experiments 24
6. Isolation of KF, TMAF, TMAF(*t*AmOH), and TBAF(*t*BuOH)$_4$ 26
6.1 Isolation of KF from PFAS-mix^{KF} 26
6.1.1 Qualitative analysis of KF^{PTFE} by NMR spectroscopy 36
6.1.2 Elemental analysis of KF^{PTFE} 38
6.1.3 Powder X-ray diffraction pattern of KF^{PTFE} 38
6.1.4 Performance of KF^{PTFE} in S$_N$Ar fluorination of 2,4-dinitrochlorobenzene 39
6.2 Isolation of KF from PTFE-mix^{PF} 39
6.3 Synthesis of 4-OHCC$_6$H$_4$BF$_3$K from KF^{PTFE} 40
6.4 Synthesis of TMAF and TMAF(*t*AmylOH) from PTFE-mix^{PF} 40
6.5 Synthesis of TBAF(*t*BuOH)$_4$ from PTFE-mix^{PF} 41
7. Phosphate recovery and recycling 43
8. Synthesis of fluorochemicals using fluorinating reagents derived from PFAS 54
9. Photographic guide for experimental setup (ball milling) 61
10. Mechanochemical destruction of various PFAS 62
10.1. General procedures 62
10.2. PFAS scope 62
11. Thermochemistry 72
12. DFT calculations 73
13. References 96
14. PFAS-mix NMR characterization 101
15. Reagents and fluorochemicals NMR characterization 155

1. Materials and methods

Unless otherwise stated, all reagents were purchased from commercial suppliers (Sigma-Aldrich, Alfa Aesar, Fluorochem, Apollo Scientific and Fisher Chemicals), used without further purification and stored under ambient conditions. Solvents were purchased from commercial suppliers and used as provided without further purification. Potassium pyrophosphate ($K_4P_2O_7$, Fluorochem, ≥99.0%, CAS 7320-34-5), sodium pyrophosphate ($Na_4P_2O_7$, Strem, ≥94.0%, CAS 13845-36-8), dipotassium hydrogen phosphate (K_2HPO_4, ≥ 98.0%, Alfa Aesar, CAS 7758-11-4), potassium dihydrogen phosphate (KH_2PO_4, ≥ 99.0%, Alfa Aesar, CAS 7778-77-0), sodium pyrophosphate ($Na_4P_2O_7$, ≥95.0%, Sigma Aldrich, CAS 7722-88-5), potassium phosphate (K_3PO_4, ≥98%, Sigma Aldrich, CAS 7778-53-2), sodium phosphate (Na_3PO_4, 96%, Sigma Aldrich, CAS 7601-54-9), lithium phosphate (Li_3PO_4, Sigma Aldrich, CAS 10377-52-3), potassium hydroxide (KOH, ≥ 85% pellets, Sigma Aldrich, CAS 1310-58-3), and sodium hydroxide (NaOH, ≥ 97.0% pellets, Sigma Aldrich, CAS 1310-73-2) were dried before use under high vacuum at 100 °C overnight and stored in a desiccator. 18-Crown-6 ($C_{12}H_{24}O_6$, ≥ 99.0%, Sigma Aldrich, CAS 17455-13-9) was recrystallized from MeCN and stored in a desiccator. Fluorspar (acid grade) was purchased from Mistral Industrial Chemicals (UK), sourced from Minersa group (Asturias region, Spain) and contains CaF_2 (> 97%), total carbonates (< 1.50%), SiO_2 (<1.00%), $BaSO_4$ (<0.50 %), Pb (< 0.10%), Fe_2O_3 (< 0.10%), S (< 0.15%), H_2O (< 1.0%).

^{1}H NMR, ^{13}C NMR, ^{31}P NMR and ^{19}F NMR spectra were recorded on Bruker AVIIIHD 400 (Operating frequencies; ^{1}H 400.130 MHz, ^{19}F 376.498 MHz, ^{31}P 161.976 MHz, ^{13}C 100.613 MHz), AVIIIHD 500 (Operating frequencies; ^{1}H 500.130 MHz, ^{19}F 470.592 MHz, ^{31}P 202.457 MHz, ^{13}C 125.758 MHz), or AVIII 500 (Operating frequencies; ^{1}H 500.130 MHz, ^{19}F 470.592 MHz, ^{31}P 202.457 MHz, ^{13}C 125.758 MHz) spectrometers equipped with a 5 mm z-gradient multinuclear BBFO probe, a 5 mm z-gradient broadband X-^{19}F/^{1}H BBFO SMART probe, or a 5 mm triple-resonance TBO probe, respectively. ^{13}C and ^{31}P NMR spectra were recorded with ^{1}H decoupling. ^{1}H NMR, ^{13}C NMR, ^{31}P NMR and ^{19}F NMR spectral data are reported as chemical shifts (δ) in parts per million (ppm) relative to the solvent peak using the Bruker internal referencing procedure (edlock). Coupling constants, J, are reported in Hz to the nearest 0.1 Hz. Unless otherwise stated, ^{13}C spectra are ^{1}H decoupled and reported coupling constants for ^{13}C spectra correspond to ^{19}F–^{13}C heteronuclear coupling. Data are reported as follows: chemical shift, multiplicity (s = singlet, d = doublet, t = triplet, q = quartet, pent = pentet, hept = heptet, br = broad, m = multiplet), coupling constants (Hz) and integration. NMR spectra were processed with MestReNova 14.1.2 or Topspin 3.5 or 4.0. Quantitative ^{19}F NMR spectra were recorded using a 10 second recycle delay, D1, using sodium triflate as an internal standard. Quantitative ^{31}P NMR spectra were recorded using a 40 second recycle delay, D1. Quantitative ^{13}C NMR spectra were recorded on a Bruker AVIIIHD 500 MHz using a 40 second D1 delay using potassium acetate as an internal standard.

Solid state NMR spectra were recorded on a Bruker AVIII 400 spectrometer (Operating frequencies; ^{1}H 400.130 MHz, ^{19}F 376.498 MHz, ^{31}P 161.976 MHz, ^{13}C 100.613 MHz) equipped with a HFXY 3.2 mm probe. Spectra were collected at 298 K and samples were acquired spinning at the magic angle $\theta = 54.7356$ °. Values of magic-angle spinning, MAS, speeds, ν_{rot}, range between 13 – 20 kHz. Powdered samples were loaded into 3.2 mm ZrO_2 rotors fitted with a Kel-F drive cap. Spectra are reported in chemical shift, δ, in parts per million (ppm) referenced against a secondary external standard ^{1}H adamantane ($\delta_H = 1.85$) [51], ^{19}F PTFE ($\delta_F = -122.7$) [52], ^{31}P the downfield resonance of ADP ($\delta_P = 10.2$) [53], and ^{13}C the downfield resonance of adamantane ($\delta_C = 38.6$) [54].

Ball milling was carried out using either a Retsch MM 400 mixer mill (30 Hz experiments), Retsch MM 500 Vario mixer mill (35 Hz experiments), an Insolido IST636 mixer mill (35 Hz experiments), or a Fritsch MicroMill 7 Pulverisette (planetary ball milling experiments). Unless otherwise stated, mechanochemical reactions were carried out in 15 mL FormTech Scientific (FTS) or 30 mL Retsch MM 500 Vario mixer mill stainless steel jars with hardened chrome grade 100 steel ball bearings (4 g (10 mm), 7 g (12 mm), 16 g (15 mm)), 15 mL or 30 mL FormTech Scientific (FTS) zirconia jar with zirconia balls (12 mm, 15 mm), or 12 mL zirconia jars with zirconia balls (3.2 g). No precaution was taken to exclude air and moisture.

Powder X-ray diffraction (PXRD) data were collected using a Bruker D8 Advance X-ray diffractometer (Bragg−Brentano geometry); the radiations Cu $K\alpha_{1,2}$ were used. Raman spectra were recorded using a LabRAM Aramis Raman Spectrometer with an Andor CCD detector. A 532 nm excitation laser and an 1800 g/mm grating were used. Spectra were recorded between 100 cm^{-1} and 4000 cm^{-1} Raman shift, using 10 s acquisition time, and a 50× objective lens.

2. Discovery of PTFE destruction

To a 15 mL (*armed with rubber sealing ring*), 30 mL (*armed with PTFE sealing ring*) or 35 mL (*armed with rubber sealing ring*) stainless-steel milling jar was added two hardened chrome steel bearings (2 × 16 g or 2 × 7 g), acid grade Fluorspar (1 equiv.) and $K_4P_2O_7$ (1 equiv. or 2.5 equiv.) or K_3PO_4 (5.0 equiv.). The total loading of material in the jar (CaF_2 and activator) was kept constant at 1000 mg or 500 mg. The jar was closed and securely fitted to the mill which was set for 3 h at a frequency of 35 Hz. Upon completion, the jar was opened and the powder was collected. An aliquot of Fluoromix (30-70 mg) and sodium triflate (10 mg, as internal standard) was extracted with D_2O (10 atom% D), centrifugated for 15-30 min and analysed by quantitative ^{19}F-NMR spectroscopy.

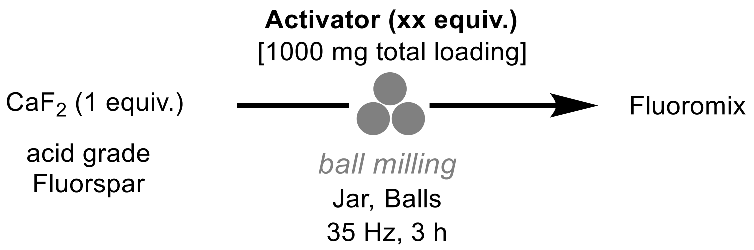

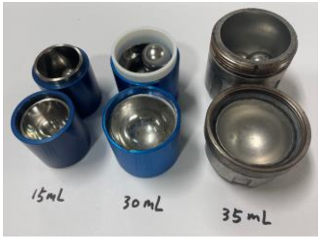

Table S1. PTFE destruction discovery.[a]

Entry	Activator (xx equiv.)	Jar	seal	Ball	PO_3F^{2-}	F^-	Fluoromix color
1	$K_4P_2O_7$ (1.0 equiv.)	35 mL	rubber	2 × 16 g	4%	52%	colorless
2	$K_4P_2O_7$ (2.5 equiv.)	35 mL	rubber	2 × 16 g	10%	70%	colorless
3	$K_4P_2O_7$ (1.0 equiv.)	30 mL	PTFE	2 × 16 g	51%	26%	grey-black
4	$K_4P_2O_7$ (2.5 equiv.)	30 mL	PTFE	2 × 16 g	140%	39%	grey-black
5	$K_4P_2O_7$ (1.0 equiv.)	15 mL	rubber	2 × 7 g	3%	52%	colorless
6	$K_4P_2O_7$ (2.5 equiv.)	15 mL	rubber	2 × 7 g	4%	85%	colorless
7[b]	K_3PO_4 (5.0 equiv.)	15 mL	rubber	2 × 7 g	n.d.	71%	colorless
8[b]	K_3PO_4 (5.0 equiv.)	30 mL	PTFE	2 × 16 g	28%	162%	grey-black
9[b]	K_3PO_4 (5.0 equiv.)	35 mL	rubber	2 × 16 g	n.d.	81%	colorless

[a] Yields were determined by quantitative ^{19}F-NMR spectroscopy in D_2O (10 atom% D) using sodium triflate as an internal standard; n.d. = not detected. [b] 30 Hz, 0.5 g total loading.

The over-stoichiometric total fluoride release observed with the 30 mL jar (armed with a PTFE sealing ring) indicated fluoride leaching from the PTFE sealing ring. This finding served as the basis for initiating the study on PFAS destruction.

3. Optimisation studies on the degradation of PTFE

PTFE degradation was investigated with a range of activators under mechanochemical conditions (ball milling). The amount of activator was calculated based on the monomer unit (CF_2CF_2) of PTFE. The yields of KF and K_2PO_3F were calculated with respect to the total fluorine content of PTFE.

3.1 Activator screening

To a 15 mL stainless-steel milling jar was added two hardened chrome steel bearings (2 × 7 g), PTFE (1 equiv.) and pre-dried activator (5 equiv. per C_2F_4). The total loading of material in the jar (PTFE and activator) was kept constant at 500 mg. The jar was closed and securely fitted to the mill which was set for 3 h at a frequency of 35 Hz. Upon completion, the jar was opened and the powder was collected. An aliquot of PTFE-mix (30-70 mg) and sodium triflate (10 mg, as internal standard) was extracted with D_2O (10 atom% D), centrifugated for 15-30 min and analysed by quantitative ^{19}F-NMR spectroscopy.

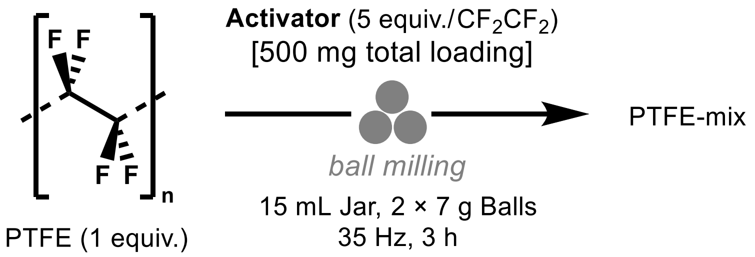

Table S2. Screening of activators for PTFE degradation.[a]

**Entry**	Activator	PO_3F^{2-}	F^-
**1**	KH_2PO_4	1%	n.d.
**2**	K_2HPO_4	26%	<1%
**3**	K_3PO_4	15%	84%
**4**	Na_3PO_4	n.d.	66%
**5**	Li_3PO_4	n.d.	7%
**6**[b]	$K_4P_2O_7$	99%	1%
**7**[b]	$Na_4P_2O_7$	15%	31%
**8**[c]	$K_5P_3O_{10}$	2%	<1%
**9**[d]	KOH	n.d.	10%
**10**	NaOH	n.d.	4%
**11**	none	n.d.	n.d.

[a] Yields were determined by quantitative ^{19}F-NMR spectroscopy in D_2O (10 atom% D) using sodium triflate as internal standard; n.d. = not detected. [b] 2.5 equiv. of activator per C_2F_4. [c] 1.67 equiv. of activator per C_2F_4. [d] KOH was heated above its melting point under high vacuum for 20 min.

3.2 Effect of material of milling jar and balls

To a 15 mL milling jar was added two bearings (materials and weight specified below), PTFE (1 equiv.) and activator (5 equiv. per C_2F_4). The total loading of material in the jar (PTFE and activator) was kept constant at 500 mg. The jar was closed and securely fitted to the mill which was set for 3 h at a frequency of 35 Hz. Upon completion, the jar was opened and the powder was collected. An aliquot of PTFE-mix (30-70 mg) and sodium triflate (10 mg, as internal standard) was extracted with D_2O (10 atom% D), centrifugated for 15-30 min and analysed by quantitative ^{19}F-NMR spectroscopy.

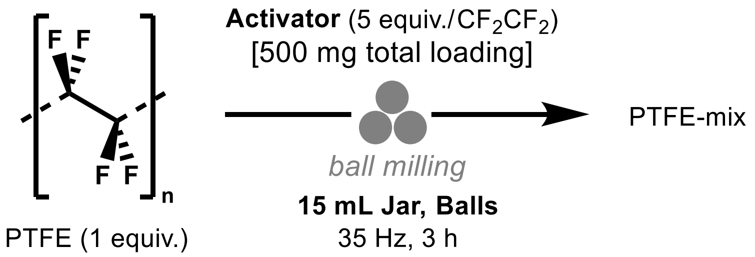

Table S3. Effect of material of milling jar and balls.[a]

Entry	Activator	Jar	Ball	PO_3F^{2-}	F^-
1	K_3PO_4	15 mL stainless-steel	2 × 7 g chrome steel	15%	84%
2	K_3PO_4	15 mL zirconia	2 × 6 g zirconia	15%	83%
3[b]	$K_4P_2O_7$	15 mL stainless-steel	2 × 7 g chrome steel	99%	1%
4[b]	$K_4P_2O_7$	15 mL zirconia	2 × 6 g zirconia	99%	<1%

[a] Yields were determined by quantitative ^{19}F-NMR spectroscopy in D_2O (10 atom% D) using sodium triflate as internal standard. [b] 2.5 equiv. of activator per C_2F_4.

3.3 Effect of hydration state of the activator

To a 15 mL stainless-steel milling jar was added two chrome steel balls (2 × 7 g), PTFE (1 equiv.) and activator (5 equiv. per C_2F_4), spiked with H_2O (as specified below). The total loading of material in the jar (PTFE, activator and H_2O) was kept constant at 500 mg. The jar was closed and securely fitted to the mill which was set for 3 h at a frequency of 35 Hz. Upon completion, the jar was opened and the powder was collected. An aliquot of PTFE-mix (30-70 mg) and sodium triflate (10 mg, as internal standard) was extracted with D_2O (10 atom% D), centrifugated for 15-30 min and analysed by quantitative ^{19}F-NMR spectroscopy.

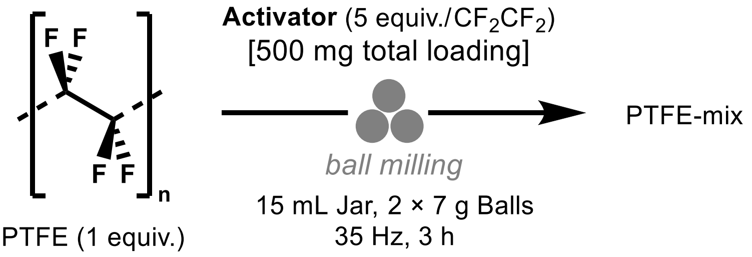

Table S4. Effect of hydration state of activator on PTFE degradation.[a]

Entry	Activator	H_2O	PO_3F^{2-}	F^-
**1**	K_3PO_4 (pre-dried[b])	none	15%	84%
**2**	$K_3PO_4 \cdot H_2O$	none	< 1%	9%
**3**	$K_3PO_4 \cdot 3H_2O$	none	n.d.	n.d.
**4**	K_3PO_4 (pre-dried[b])	2.5 equiv.	6%	79%
**5**	K_3PO_4 (pre-dried[b])	5.0 equiv.	< 1%	22%
**6**	KOH (pellets)	none	n.d.	10%
**7**	KOH (pre-dried[c])	none	n.d.	10%

[a] Yields were determined by quantitative ^{19}F-NMR spectroscopy in D_2O (10 atom% D) using sodium triflate as internal standard; n.d. = not detected. [b] K_3PO_4 was dried at 120 °C under high vacuum overnight. [c] KOH was heated above its melting point under high vacuum for 20 min.

3.4 Optimisation of activator stoichiometry

To a 15 mL stainless-steel milling jar was added two hardened chrome steel bearings (2 × 7 g), PTFE (1 equiv.) and K_3PO_4 (x equiv. per C_2F_4). The total loading of material in the jar (PTFE and K_3PO_4) was kept constant at 500 mg. The jar was closed and securely fitted to the mill which was set for 3 h at a frequency of 35 Hz. Upon completion, the jar was opened and the powder was collected. An aliquot of PTFE-mix^{KF} (30-70 mg) and sodium triflate (10 mg, as internal standard) was extracted with D_2O (10 atom% D), centrifugated for 15-30 min and analysed by quantitative ^{19}F-NMR spectroscopy.

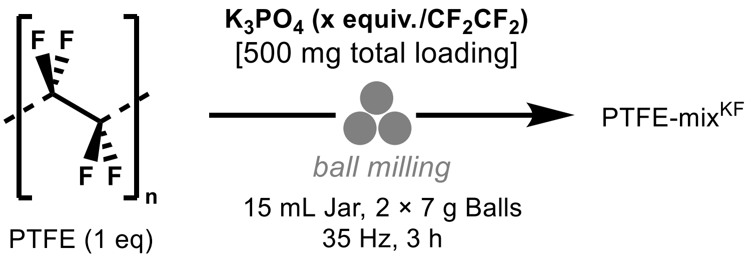

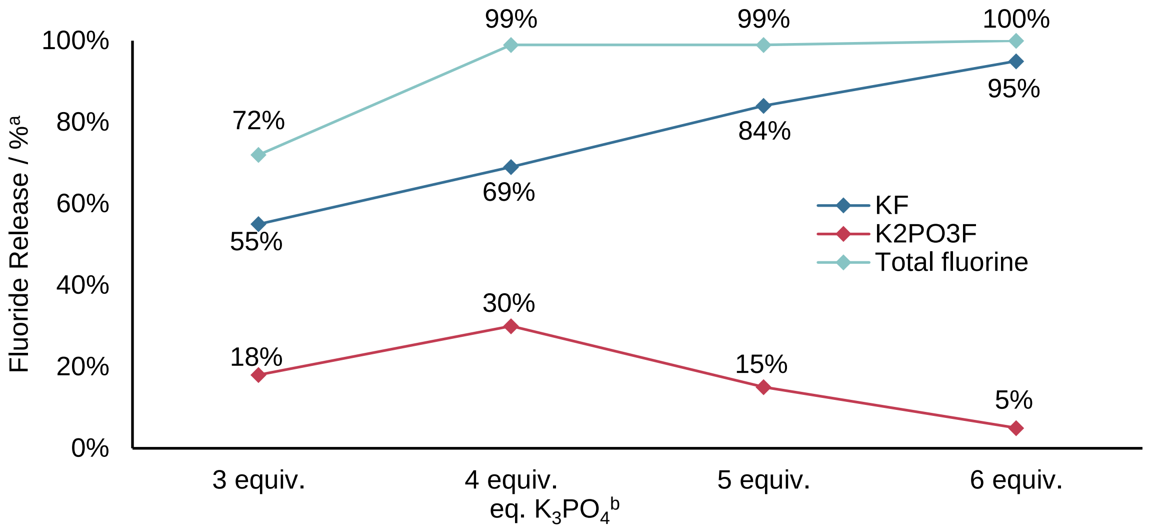

Figure S1. Optimisation of K_3PO_4 equivalents for PTFE-mix^{KF} formation. [a] Fluoride release quantified as water soluble F^- and PO_3F^{2-} determined by quantitative ^{19}F NMR spectroscopy, expressed as percent of the total fluorine content of PTFE. [b] Number of equivalents given relative to a C_2F_4 subunit of PTFE.

To a 15 mL stainless-steel milling jar was added two chrome steel balls (2 × 7 g), PTFE (1 equiv.) and $K_4P_2O_7$ (x equiv. per C_2F_4). The total loading of material in the jar (PTFE and $K_4P_2O_7$) was kept constant at 500 mg. The jar was closed and securely fitted to the mill which was set for 3 h at a frequency of 35 Hz. Upon completion, the jar was opened and the powder was collected. An aliquot of PTFE-mix^{PF} (30-70 mg) and sodium triflate (10 mg, as internal standard) was extracted with D_2O (10 atom% D), centrifugated for 15-30 min and analysed by quantitative ^{19}F-NMR spectroscopy.

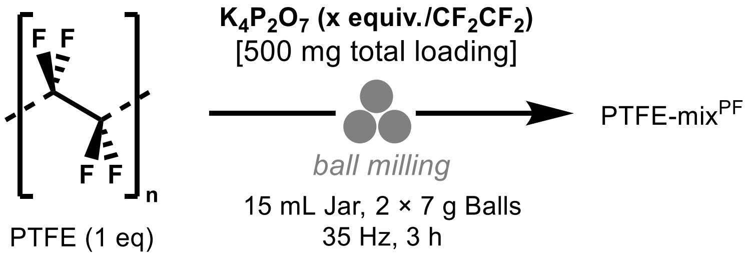

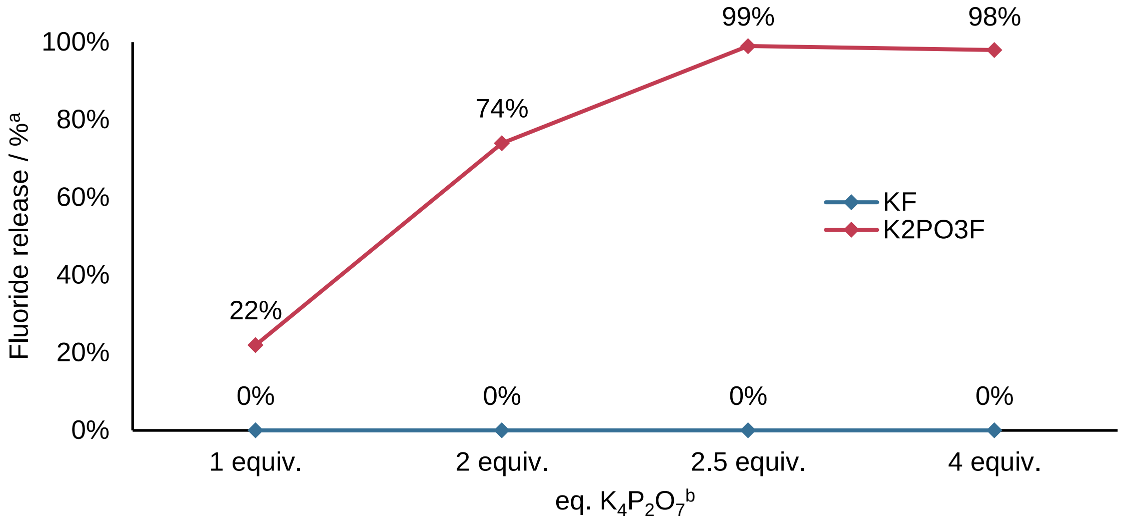

Figure S2. Optimisation of $K_4P_2O_7$ equivalents for PTFE-mix^{PF} formation. [a]Yields were determined by quantitative ^{19}F-NMR spectroscopy in D_2O (10 atom% D) using sodium triflate as internal standard, expressed as percent of the total fluorine content of PTFE. [b] Number of equivalents given relative to a C_2F_4 subunit of PTFE.

3.5 Optimisation of mechanochemical parameters

Jar size and ball bearings:

To a stainless-steel milling jar was added two hardened chrome steel bearings (2 × 7 g), PTFE (1 equiv., 43 mg) and K_3PO_4 (1.25 equiv./F, 457 mg). The total loading of material in the jar (PTFE and K_3PO_4) was kept constant at 500 mg. The jar was closed and securely fitted to the mill which was set for 3 h at a frequency of 35 Hz. Upon completion, the jar was opened and the powder was collected. An aliquot of PTFE-mix^{KF} (30-70 mg) and sodium triflate (10 mg, as internal standard) was extracted with D_2O (10 atom% D), centrifugated for 15-30 min and analysed by quantitative ^{19}F-NMR spectroscopy.

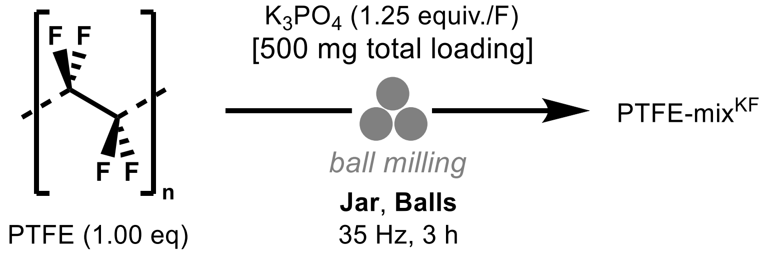

Table S5. Optimisation of jar and ball size for K_3PO_4-mediated PTFE degradation.[a]

Entry	Jar Volume	Ball	PO_3F^{2-}	F^-
1	15 mL	1 × 4 g	< 1%	15%
2	15 mL	2 × 4 g	14%	82%
3	15 mL	1 × 7 g	< 1%	37%
4	15 mL	2 × 7 g	15%	84%
5[b]	15 mL	2 × 6 g	15%	84%
6	15 mL	1 × 16 g	25%	43%
7[c]	35 mL	2 × 16 g	20%	80%

[a] Yields were determined by quantitative ^{19}F-NMR spectroscopy in D_2O (10 atom% D) using sodium triflate as internal standard. [b] Ball milling as performed in a 15 mL zirconium jar with 2 × 6 g zirconium balls. [c] 1000 mg total loading.

To a stainless-steel milling jar was added hardened chrome steel bearings, PTFE (1 equiv., 54 mg) and $K_4P_2O_7$ (0.625 equiv./F, 446 mg). The total loading of material in the jar (PTFE and $K_4P_2O_7$) was kept constant at 500 mg. The jar was closed and securely fitted to the mill which was set for 3 h at a frequency of 35 Hz. Upon completion, the jar was opened and the powder was collected. An aliquot of PTFE-mix^{PF} (30-70 mg) and sodium triflate (10

mg, as internal standard) was extracted with D_2O (10 atom% D), centrifugated for 15-30 min and analysed by quantitative ^{19}F-NMR spectroscopy.

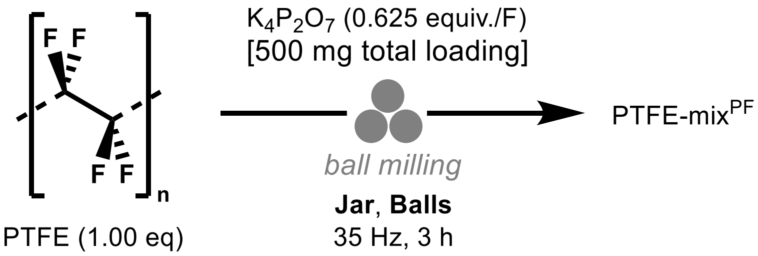

Table S6. Optimisation of jar and ball size for $K_4P_2O_7$-mediated PTFE destruction.[a]

Entry	Jar Volume	Ball	PO_3F^{2-}	F^-
1	15 mL	1 × 4 g	10%	n.d.
2	15 mL	2 × 4 g	90%	n.d.
3	15 mL	1 × 7 g	20%	n.d.
4	15 mL	2 × 7 g	99%	1%
5[b]	15 mL	2 × 6 g	99%	1%
6	15 mL	1 × 16 g	49%	5%
7[c]	35 mL	2 × 16 g	99%	n.d.

[a] Yields were determined by quantitative ^{19}F-NMR spectroscopy in D_2O (10 atom% D) using sodium triflate as internal standard; n.d. = not detected. [b] Ball milling as performed in a 15 mL zirconium jar with 2 × 6 g zirconium balls. [c] 1000 mg total loading.

Milling frequency:

To a 15 mL stainless-steel milling jar was added two hardened chrome steel bearings (2 × 7 g), PTFE (1 equiv., 43) and K_3PO_4 (1.25 equiv./F, 457 mg). The total loading of material in the jar (PTFE and activator) was kept constant at 500 mg. The jar was closed and securely fitted to the mill which was set for 3 h at a frequency of 30 Hz or 35 Hz. Upon completion, the jar was opened and the powder was collected. An aliquot of PTFE-mix^{KF} (30-70 mg) and sodium triflate (10 mg, as internal standard) was extracted with D_2O (10 atom% D), centrifugated for 15-30 min and analysed by quantitative ^{19}F-NMR spectroscopy.

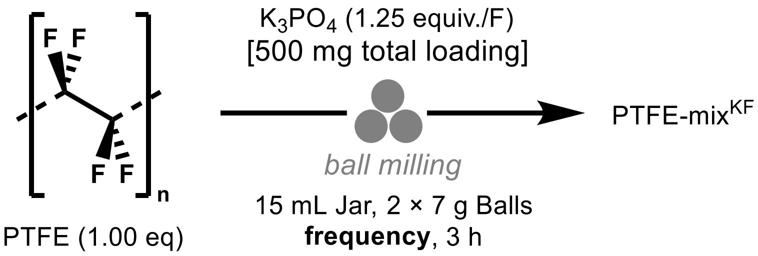

Table S7. Effect of milling frequency on PTFE-mix^{KF} formation.[a]

Entry	Frequency (Hz)	PO_3F^{2-}	F^-
1	30	19%	80%
2	35	15%	84%

[a] Yields were determined by quantitative ^{19}F-NMR spectroscopy in D_2O (10 atom% D) using sodium triflate as an internal standard.

To a 15 mL stainless-steel milling jar was added two hardened chrome steel bearings (2 × 7 g), PTFE (1 equiv., 54 mg) and $K_4P_2O_7$ (0.625 equiv./F, 446 mg). The total loading of material in the jar (PTFE and activator) was kept constant at 500 mg. The jar was closed and securely fitted to the mill which was set for 3 h at the frequency of 30 Hz or 35 Hz. Upon completion, the jar was opened and the powder was collected. An aliquot of PTFE-mix^{PF} (30-70 mg) and sodium triflate (10 mg, as internal standard) was extracted with D_2O (10 atom% D), centrifugated for 15-30 min and analysed by quantitative ^{19}F-NMR spectroscopy.

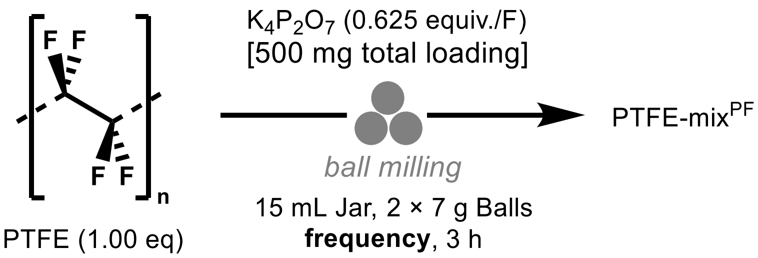

Table S8. Effect of milling frequency on PTFE-mix^{PF} formation.[a]

Entry	Frequency (Hz)	PO_3F^{2-}	F^-
1	30	59%	7%
2	35	99%	1%

[a] Yields were determined by quantitative ^{19}F-NMR spectroscopy in D_2O (10 atom% D) using sodium triflate as an internal standard.

Planetary ball milling:

To a 12 mL zirconia milling jar was added six zirconia bearings (6 × 3.2 g), PTFE (1 equiv., 43 mg) and K_3PO_4 (1.25 equiv./F, 457 mg). The total loading of material in the jar (PTFE and activator) was kept constant at 500 mg. The jar was closed and securely fitted to the mill which was set for multiple 3 h cycles at a frequency of 800 rpm. Upon completion, the jar was opened and the powder was collected. An aliquot of PTFE-mix^{KF} (30-70 mg) and sodium triflate (10 mg, as internal standard) was extracted with D_2O (10 atom% D), centrifugated for 15-30 min and analysed by quantitative ^{19}F-NMR spectroscopy.

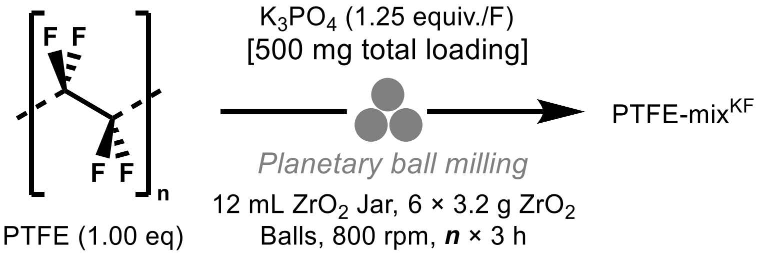

Table S9. Effect of planetary ball milling on PTFE-mix^{KF} formation.[a]

Entry	milling time	PO_3F^{2-}	F^-
1	3 h	no deg.[b]	no deg.[b]
2	3 × 3 h	2%	52%
3	8 × 3 h	24%	84%

[a] Yields were determined by quantitative ^{19}F-NMR spectroscopy in D_2O (10 atom% D) using sodium triflate as an internal standard. [b] No degradation observed after 3 h.

To a 12 mL zirconia milling jar was added six zirconia bearings (6 × 3.2 g), PTFE (1 equiv., 53 mg) and $K_4P_2O_7$ (0.625 equiv./F, 447 mg). The total loading of material in the jar (PTFE and activator) was kept constant at 500 mg. The jar was closed and securely fitted to the mill which was set for multiple 3 h cycles at a frequency of 800 rpm. Upon completion, the jar was opened and the powder was collected. An aliquot of PTFE-mix^{KF} (30-70 mg) and sodium triflate (10 mg, as internal standard) was extracted with D_2O (10 atom% D), centrifugated for 15-30 min and analysed by quantitative ^{19}F-NMR spectroscopy.

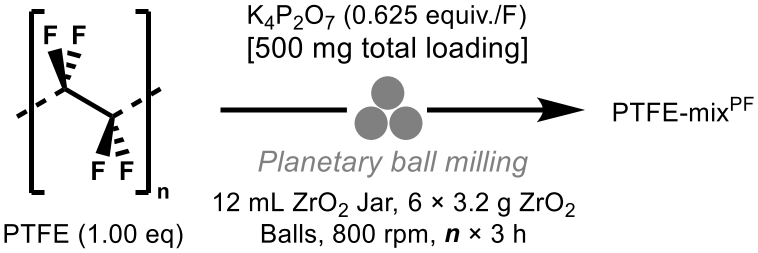

Table S10. Effect of planetary ball milling on PTFE-mix^{PF} formation.[a]

Entry	milling time	PO_3F^{2-}	F^-
1	3 h	no deg.[b]	no deg.[b]
2	3 × 3 h	38%	6%
3	8 × 3 h	96%	10%

[a] Yields were determined by quantitative ^{19}F-NMR spectroscopy in D_2O (10 atom% D) using sodium triflate as an internal standard. [b] No degradation observed after 3 h.

3.6 Optimisation of total material loading

To a 15 mL stainless-steel milling jar was added two hardened chrome steel bearings (2 × 7 g), PTFE (1 equiv.) and K_3PO_4 (1.25 equiv./F). The total loading of material in the jar (PTFE and K_3PO_4) was varied as specified in the table below. The jar was closed and securely fitted to the mill which was set for 3 h at a frequency of 35 Hz. Upon completion, the jar was opened and the powder was collected. An aliquot of PTFE-mix^{KF} (30-70 mg) and sodium triflate (10 mg, as internal standard) was extracted with D_2O (10 atom% D), centrifugated for 15-30 min and analysed by quantitative ^{19}F-NMR spectroscopy.

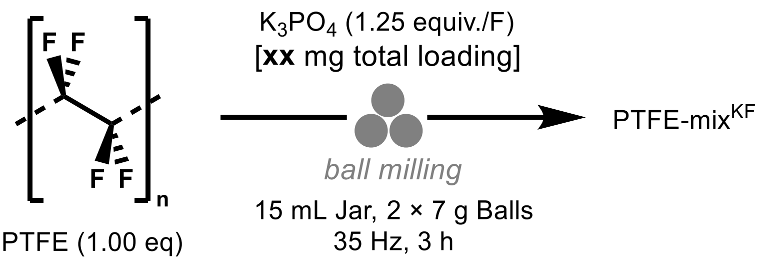

Table S11. Optimisation of total loading for PTFE-mix^{KF} formation.[a]

Entry	Total material loading (mg)	PO_3F^{2-}	F^-
1	500	15%	84%
2	1000	2%	84%
3	1500	2%	62%

[a] Yields were determined by quantitative ^{19}F-NMR spectroscopy in D_2O (10 atom% D) using sodium triflate as an internal standard.

To a 15 mL stainless-steel milling jar was added two hardened chrome steel bearings (2 × 7 g), PTFE (1 equiv.) and $K_4P_2O_7$ (0.625 equiv./F). The total loading of material in the jar (PTFE and $K_4P_2O_7$) was varied as specified in the table below. The jar was closed and securely fitted to the mill which was set for 3 h at a frequency of 30 Hz or 35 Hz. Upon completion, the jar was opened and the powder was collected. An aliquot of PTFE-mix^{PF} (30-70 mg) and sodium triflate (10 mg, as internal standard) was extracted with D_2O (10 atom% D), centrifugated for 15-30 min and analysed by quantitative ^{19}F-NMR spectroscopy.

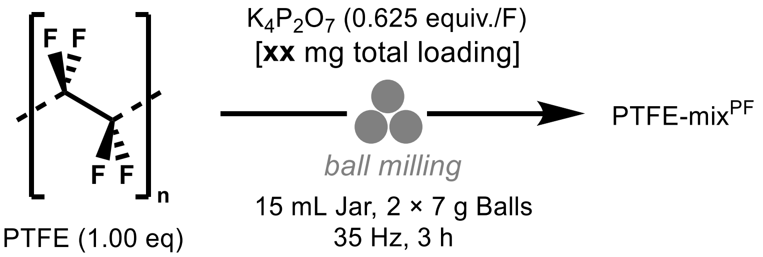

Table S12. Optimisation of total loading for PTFE-mix^{PF} formation.[a]

Entry	Total material loading (mg)	PO_3F^{2-}	F^-
1	500	99%	1%
2	1000	53%	8%
3	1500	32%	7%

[a] Yield were determined by quantitative ^{19}F-NMR spectroscopy in D_2O (10 atom% D) using sodium triflate as an internal standard.

3.7 Optimisation of milling time

To a 15 mL stainless-steel milling jar was added two hardened chrome steel bearings (2 × 7 g), PTFE (1 equiv., 43 mg) and K_3PO_4 (1.25 equiv./F, 457 mg). The total loading of material in the jar (PTFE and K_3PO_4) was kept constant at 500 mg. The jar was closed and securely fitted to the mill which was set for a specified amount time (vide infra) at a frequency of 35 Hz. Upon completion, the jar was opened and the powder was collected. An aliquot of PTFE-mix^{KF} (30-70 mg) and sodium triflate (10 mg, as internal standard) was extracted with D_2O (10 atom% D), centrifugated for 15-30 min and analysed by quantitative ^{19}F-NMR spectroscopy.

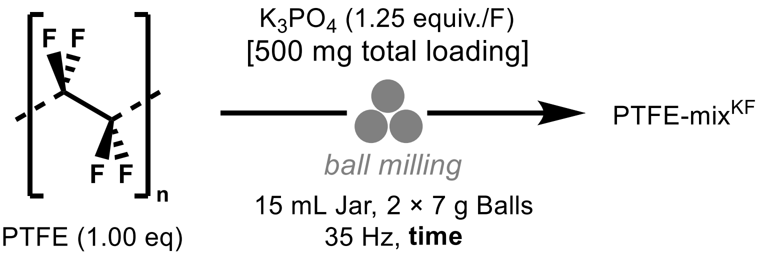

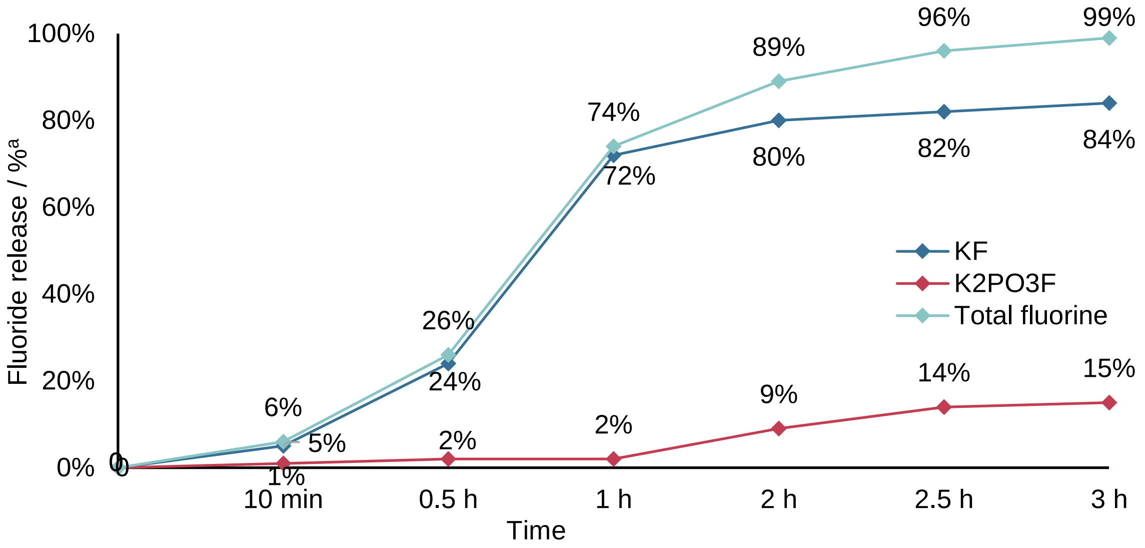

Figure S3. Time course for the mineralisation of PTFE with K_3PO_4. [a]Yields were determined by quantitative ^{19}F-NMR spectroscopy in D_2O (10 atom% D) using sodium triflate as an internal standard, expressed as percent of the total fluorine content of PTFE.

To a 35 mL stainless-steel milling jar was added two hardened chrome steel bearings (2 × 16 g), PTFE (1 equiv., 108 mg) and $K_4P_2O_7$ (0.625 equiv./F, 892 mg). The total loading of material in the jar (PTFE and $K_4P_2O_7$) was kept constant at 1000 mg The jar was closed and securely fitted to the mill which was set for a specified amount time (vide infra) at a frequency of 35 Hz. Upon completion, the jar was opened and the powder was collected. An aliquot of 30-70 mg PTFE-mix^{PF} and sodium triflate (10 mg, as internal standard) was extracted with D_2O (10 atom% D), centrifugated for 15-30 min and analysed by quantitative ^{19}F-NMR spectroscopy.

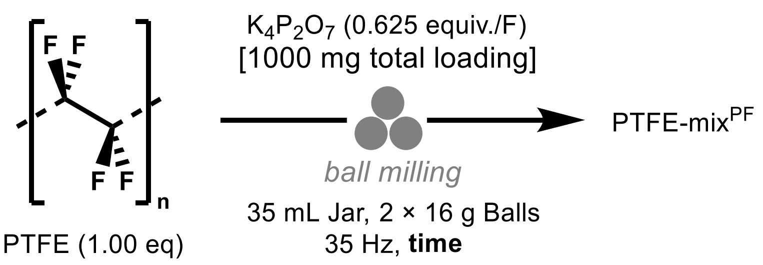

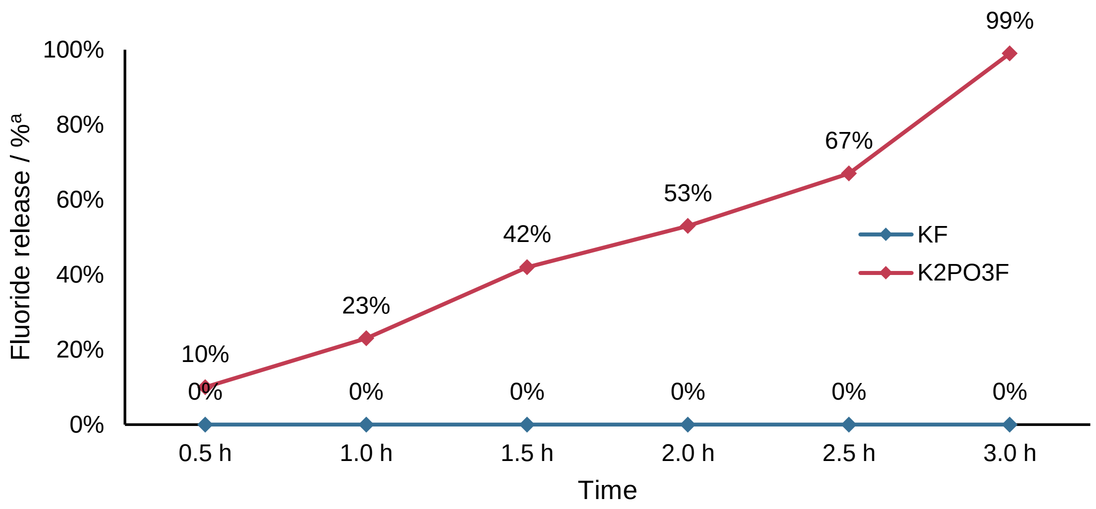

Figure S4. Time course for the mineralisation of PTFE with $K_4P_2O_7$. [a]Yields were determined by quantitative ^{19}F-NMR spectroscopy in D_2O (10 atom% D) using sodium triflate as an internal standard, expressed as a percent of the total fluorine content of the starting PTFE.

3.8 PFAS degradation in solution

To a vial was added PTFE (1 equiv., 25 mg) or perfluorooctane (C_8F_{18}, 1 equiv., 0.25 mmol, 110 mg), activator (2.0 equiv./F), and solvent (2.7 mL). After stirring at 120 °C in an oil bath for 24 h, the resulting suspension was cooled to room temperature, and analysed by quantitative ^{19}F-NMR spectroscopy with sodium triflate (10 mg) as an internal standard.

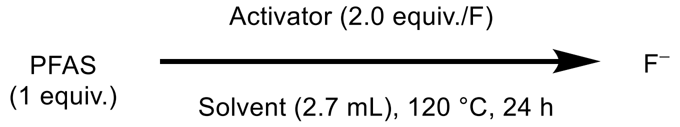

Table S13. Screening of PFAS degradation in solution.[a]

Entry	PFAS	activator	solvent	Yield of F^-
1	PTFE	K_3PO_4 (2 equiv./ F)	H_2O	n.d.
2	PTFE	K_3PO_4 (2 equiv./ F)	DMSO: H_2O (8:1)	n.d.
3	PTFE	$K_4P_2O_7$ (2 equiv./ F)	H_2O	n.d.
4	PTFE	$K_4P_2O_7$ (2 equiv./ F)	DMSO: H_2O (8:1)	n.d.
5	C_8F_{18}	K_3PO_4 (2 equiv./ F)	H_2O	n.d.
6	C_8F_{18}	K_3PO_4 (2 equiv./ F)	DMSO: H_2O (8:1)	n.d.
7	C_8F_{18}	$K_4P_2O_7$ (2 equiv./ F)	H_2O	n.d.
8	C_8F_{18}	$K_4P_2O_7$ (2 equiv./ F)	DMSO: H_2O (8:1)	n.d.

[a] Yields were determined by quantitative ^{19}F-NMR spectroscopy in D_2O (10 atom% D) using sodium triflate as an internal standard, expressed as percent of the total fluorine content of PFAS; n.d. = not detected.

3.9 Fluoropolymer particle size effect

To a 15 mL stainless-steel milling jar was added two hardened chrome steel bearings (2 × 7 g), fluoropolymer (1 equiv.) and K_3PO_4 (1.25 equiv./F). The total loading of material in the jar (PTFE and K_3PO_4) was kept constant at 500 mg. The jar was closed and securely fitted to the mill which was set for 3 h at a frequency of 35 Hz. Upon completion, the jar was opened and the powder was collected. An aliquot of PTFE-mix^{KF} (30-70 mg) and sodium triflate (10 mg, as internal standard) was extracted with D_2O (10 atom% D), centrifugated for 15-30 min and analysed by quantitative ^{19}F-NMR spectroscopy.

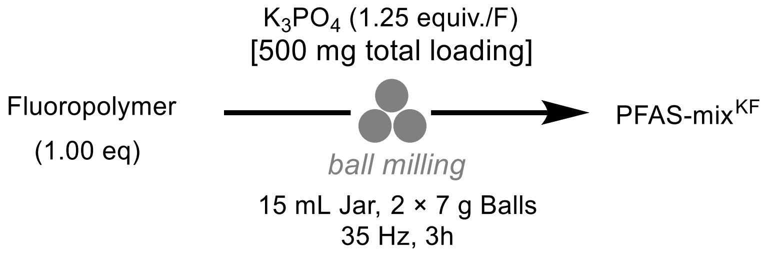

Table S14. A (Entry 1–5) Effect of PTFE starting particle size on mechanochemical activation[a]. B (Entry 6–8) Effect of PVDF M_w on mechanochemical activation[a]. C (Entry 9–10) Effect of PVDF-HFP M_w on mechanochemical activation[a].

Entry	Polymer	Particle Size [µm][b]	F^{total} Release [%][c]	$F^-:PO_3^{2-}$
1	PTFE	1	>99	3.6:1.0
2	PTFE	<12	98	4.2:1.0
3	PTFE	35	>99	3.9:1.0
4	PTFE	>40	>99	4.6:1.0
5	PTFE	200	>99	2.7:1.0
**Entry**	**Polymer**	**M_w [g·mol^{-1}][d]**	**F^{total} Release [%][c]**	**$F^-:PO_3^{2-}$**
6	PVDF	180,000	94	11:1.0
7[e]	PVDF	180,000	99	1.0:6.0
8	PVDF	534,000	>99	11:1.0
**Entry**	**Polymer**	**M_w [g·mol^{-1}][d]**	**F^{total} Release [%][c]**	**$F^-:PO_3^{2-}$**
9	PVDF-HFP	400,000	96	8.0:1.0
10	PVDF-HFP	455,000	93	8.3:1.0

[a] 0.5 g total mass of sample loaded into 15 mL steel jars with 2×7 g chrome hardened steel bearings. Samples milled for 3 h at 35 Hz using a Retsch MM500 Vario mixer mill. [b] Particle size as stated by manufacturer. [c] Yield determined by quantitative ^{19}F-NMR spectroscopy in D_2O (10 atom% D) using sodium triflate as an internal standard. [d] M_w as stated by manufacturer, calculated by GPC. [e] $K_4P_2O_7$ (0.625 equiv./F) used instead of K_3PO_4.

4. Composition of PTFE-mix

4.1 Composition of PTFE-mix^{KF}

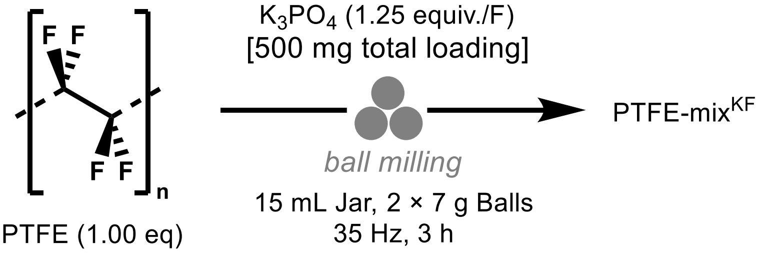

To a 15 mL stainless-steel milling jar was added two hardened chrome steel bearings (2 × 7 g), PTFE (1 equiv., 43 mg) and K_3PO_4 (1.25 equiv./F, 457 mg). The total loading of material in the jar (PTFE and K_3PO_4) was kept constant at 500 mg. The jar was closed and securely fitted to the mill which was set for 3 h at a frequency of 35 Hz. Upon completion, the jar was opened and the powder was collected and extracted with H_2O/D_2O = 9/1 (5 mL). Subsequently, KOAc (200 µL, 0.509 M solution in D_2O) was added as internal standard, the sample was concentrated to a volume of approx. 1 mL and centrifugated. The clear supernatant was analysed by NMR spectroscopy. The water-insoluble black solid (5 mg) was analysed by Raman spectroscopy.

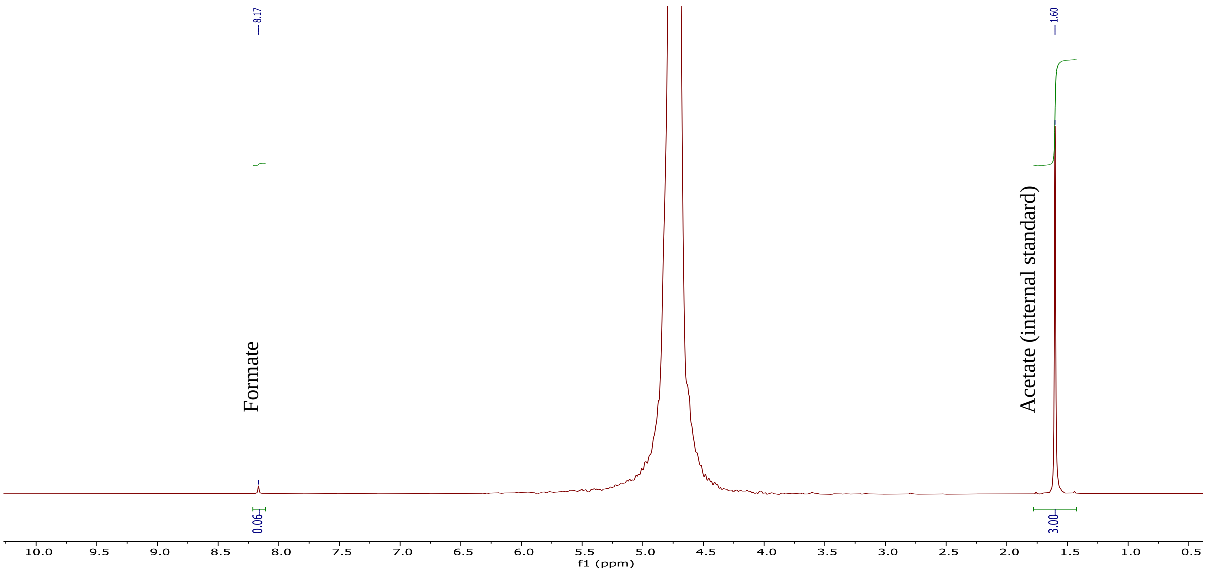

Figure S5. Quantitative ^{1}H NMR spectrum (400 MHz, 4 scans at 25 s recycle delay) of the whole jar PTFE-mix^{KF} in D_2O (10 atom% D) with KOAc (200 µL, 0.509 M solution in D_2O) as an internal standard indicates the presence of formate (δ_H = 8.17, 1%).

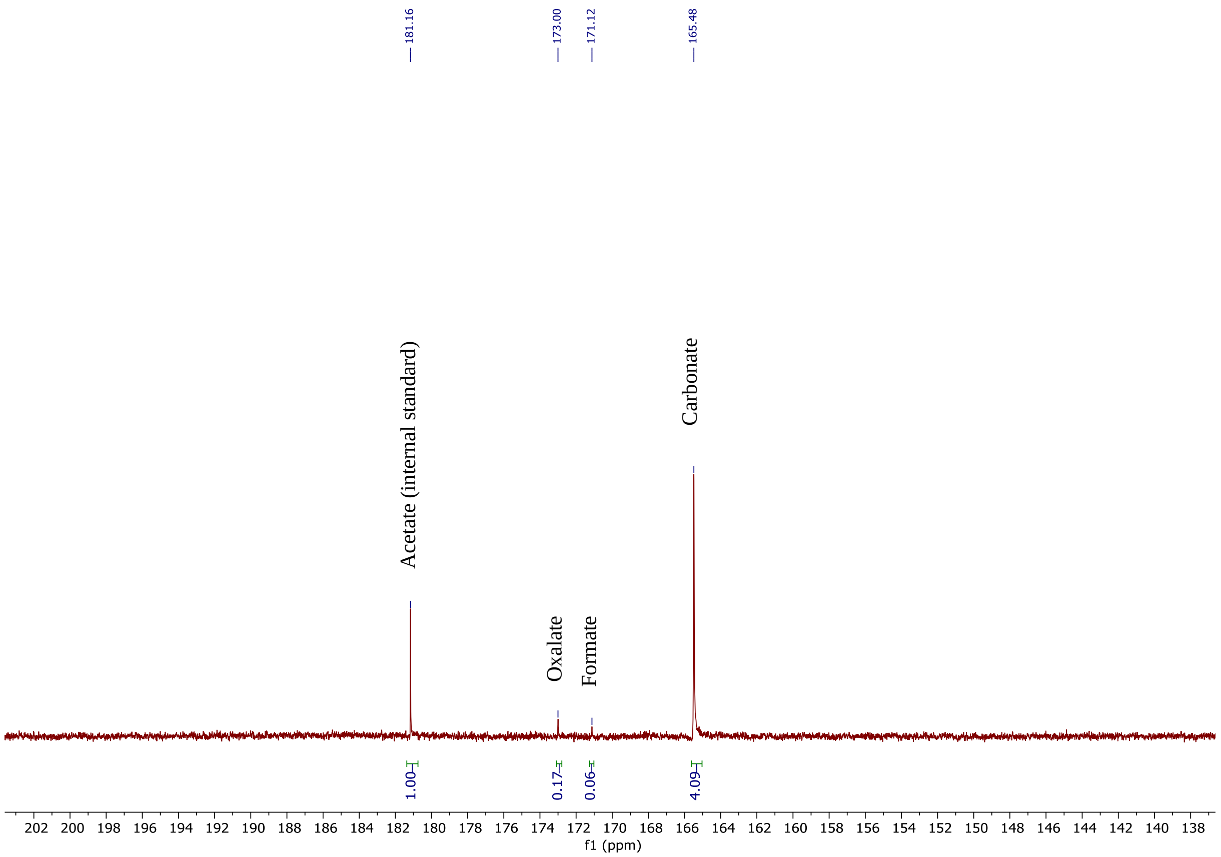

Figure S6. Quantitative $^{13}C\{^1H\}$ NMR spectrum (126 MHz, 1500 scans at 40 s recycle delay) of the whole jar PTFE-mix^{KF} in D_2O (10 atom% D) with KOAc (200 µL, 0.509 M solution in D_2O) as an internal standard indicates carbon content of carbonate (δ_C = 165.5, 48%), formate (δ_C = 171.1, 1%), and oxalate (δ_C = 173.0, 2%).

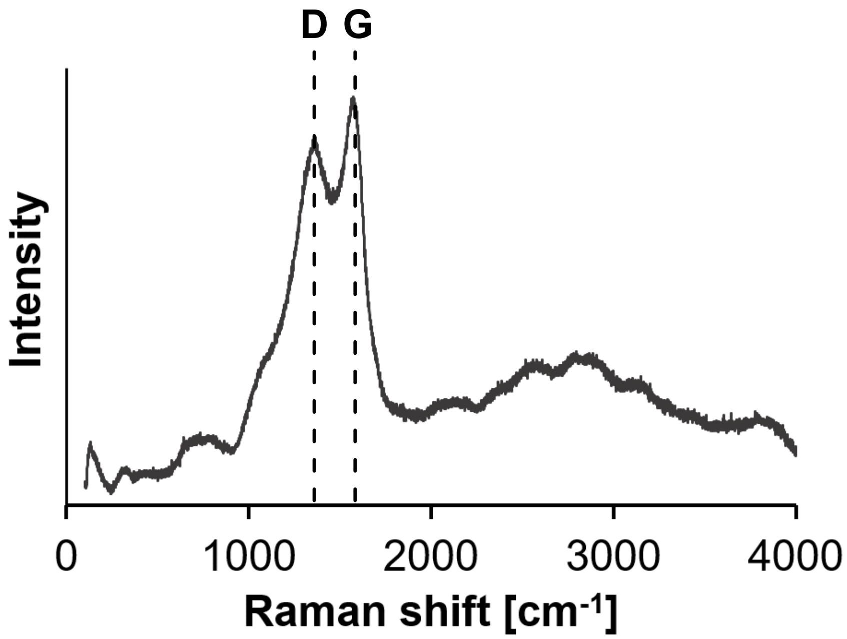

Figure S7. Raman spectra of the isolated water-insoluble black residue (5 mg) from the whole jar PTFE-mix^{KF}, indicating the presence of carbon, isolated yield 48%. Disordered (D) and graphitic (G) vibrational modes have been labelled.

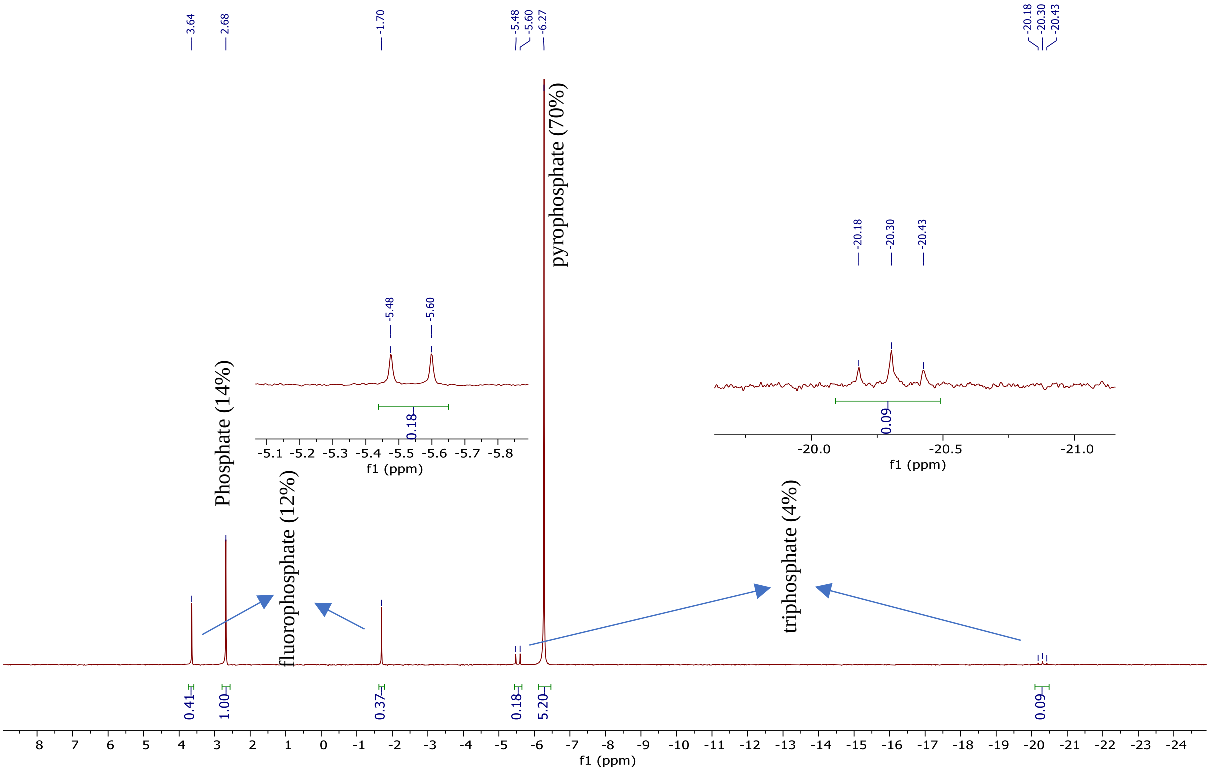

Figure S8. Quantitative ^{31}P NMR spectrum (162 MHz, 16 scans at 40 s recycle delay) of an aliquot of PTFE-mix^{KF} in D_2O (10 atom% D) indicates phosphorus content of fluorophosphate (δ_P = 1.0 (d, $^1J_{PF}$ = 867 Hz), 12%), phosphate (δ_P = 2.7, 14%), pyrophosphate (δ_P = −6.3, 70%), and triphosphate (δ_P = −5.5 (d, $^2J_{PP}$ = 20 Hz), −20.3 (t, $^2J_{PP}$ = 20 Hz), 4%).

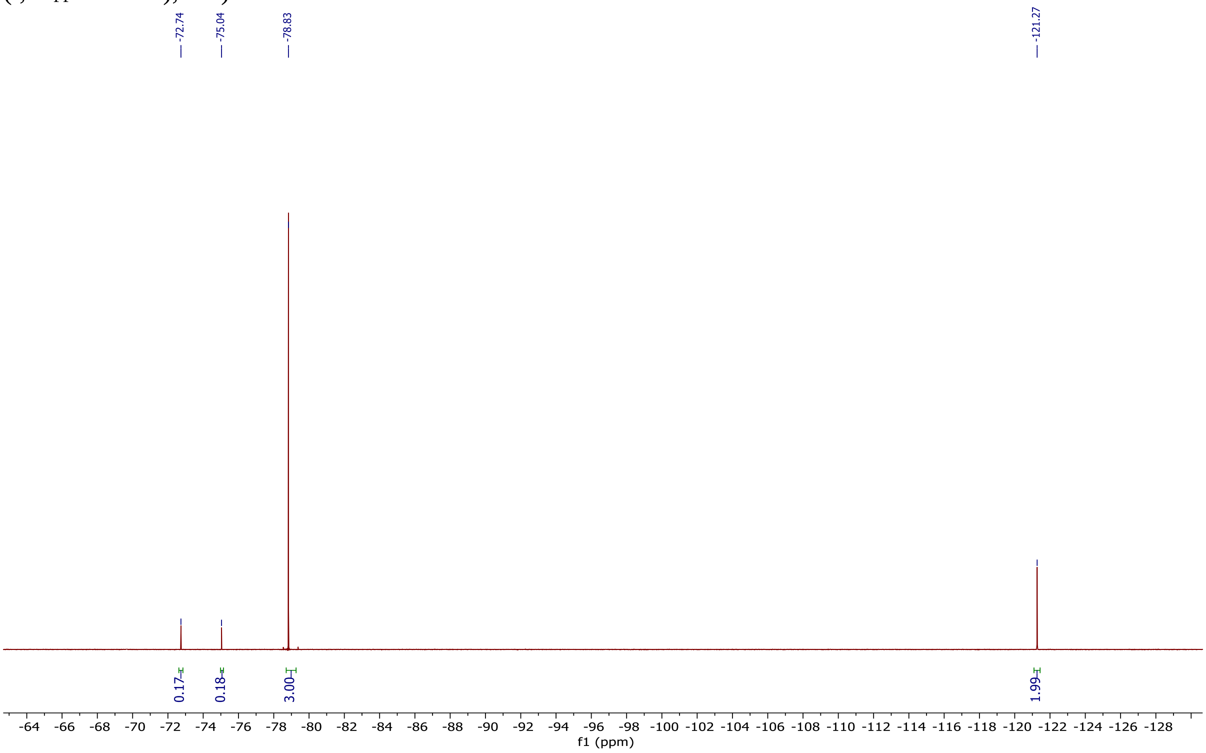

Figure S9. Quantitative ^{19}F NMR spectrum (377 MHz, 16 scans at 10 s recycle delay) of an aliquot of PTFE-mix^{KF} in D_2O (10 atom% D) using sodium triflate as an internal standard, indicates fluorine content of fluoride (δ_F = −121.3, 84%), and fluorophosphate (δ_P = −73.7 (d, $^1J_{PF}$ = 867 Hz), 15%).

4.2 Composition of PTFE-mix^{PF}

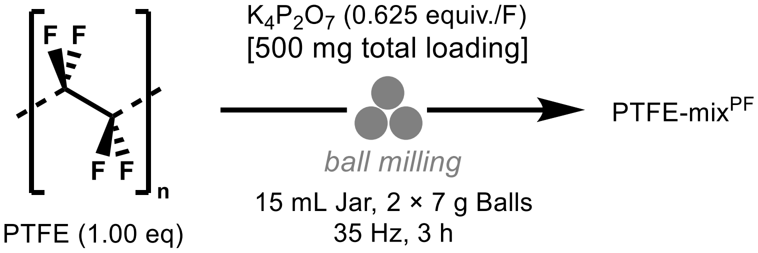

To a 15 mL stainless-steel milling jar was added two hardened chrome steel bearings (2 × 7 g), PTFE (1 equiv., 54 mg) and $K_4P_2O_7$ (0.625 equiv./F, 446 mg). The total loading of material in the jar (PTFE and $K_4P_2O_7$) was kept constant at 500 mg. The jar was closed and securely fitted to the mill which was set for 3 h at a frequency of 35 Hz. Upon completion, the jar was opened and the powder was collected and extracted with H_2O/D_2O = 9/1 (5 mL). Subsequently, KOAc (200 µL, 0.509 M solution in D_2O) was added as internal standard, the sample was concentrated to a volume of approx. 1 mL and centrifugated. The clear supernatant was analysed by NMR spectroscopy. The water-insoluble black solid (6.2 mg) was analysed by Raman spectroscopy.

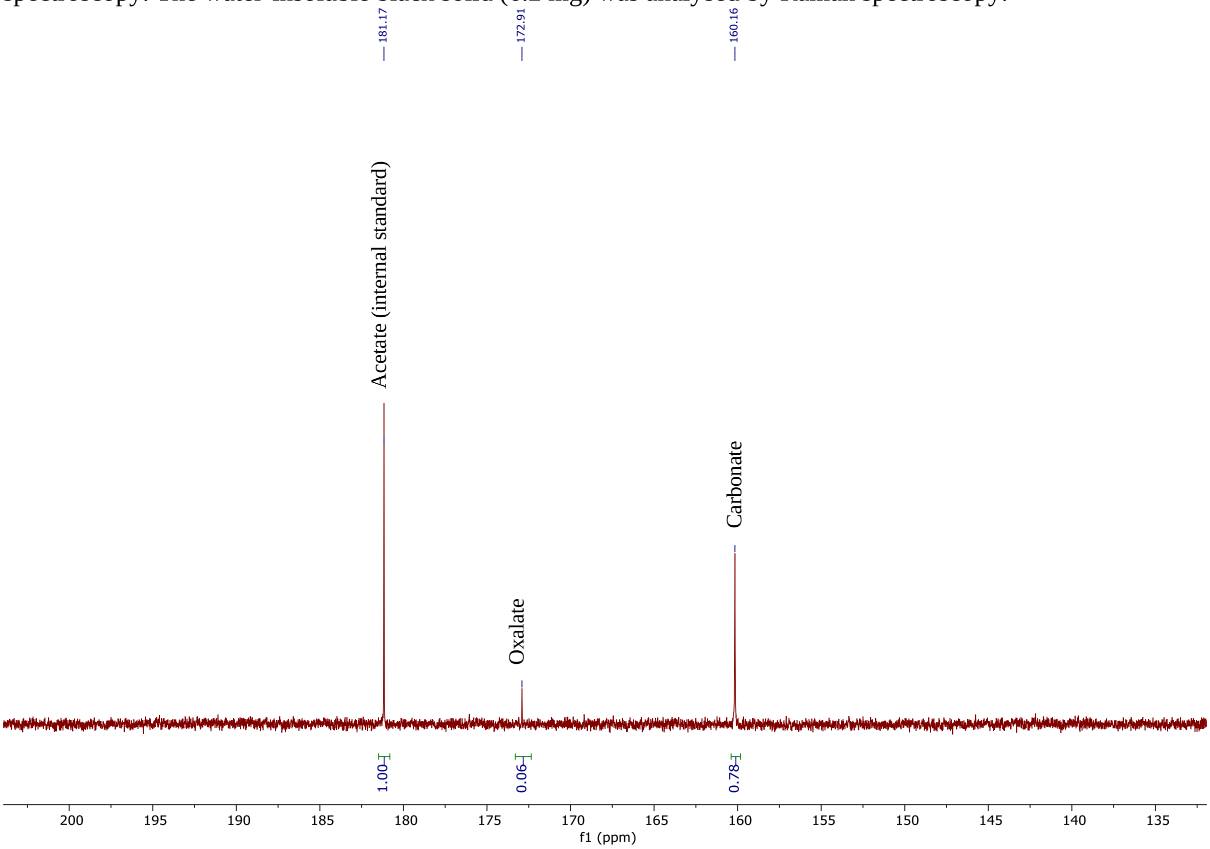

Figure S10. Quantitative $^{13}C\{^1H\}$ NMR spectrum (126 MHz, 2600 scans at 40 s recycle delay) of the whole jar PTFE-mix^{PF} in D_2O (10 atom% D) with KOAc (200 µL, 0.509 M solution in D_2O) as an internal standard indicates carbon content of carbonate (δ_C = 160.2, 7%), and oxalate (δ_C = 173.0, 1%).

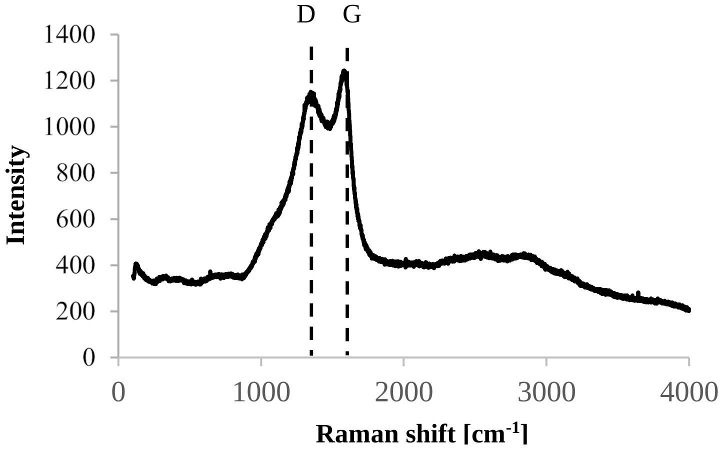

Figure S11. Raman spectra of the water-insoluble black residue (6.2 mg) from the whole jar PTFE-mix^{PF} indicates the presence of carbon, isolated yield 48%. Disordered (D) and graphitic (G) vibrational modes have been labelled.

CO_2 detection and quantification

For quantification any CO_2 released was captured with aqueous KOH (100 mg/mL) using KOAc as an internal standard.

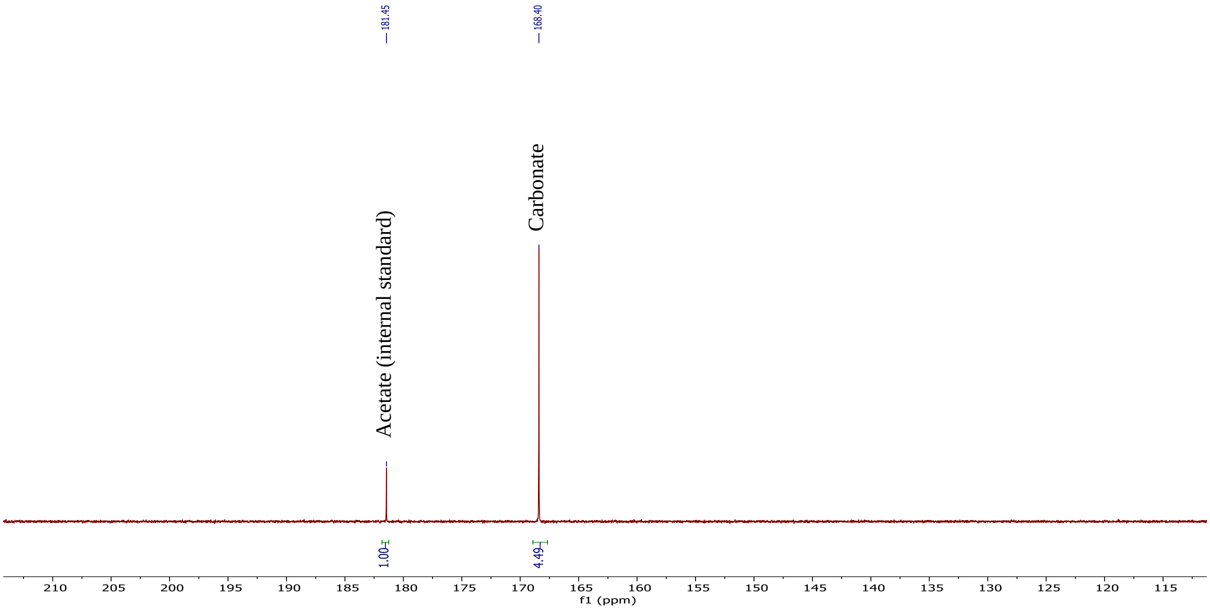

Figure S12. Quantitative $^{13}C\{^{1}H\}$ NMR spectrum (126 MHz, 2500 scans at 40 s recycle delay) in D_2O (10 atom% D) with KOAc (200 µL of a 0.509 M solution in D_2O) as an internal standard indicates carbon content of carbonate (δ_C = 168.4, 42%).

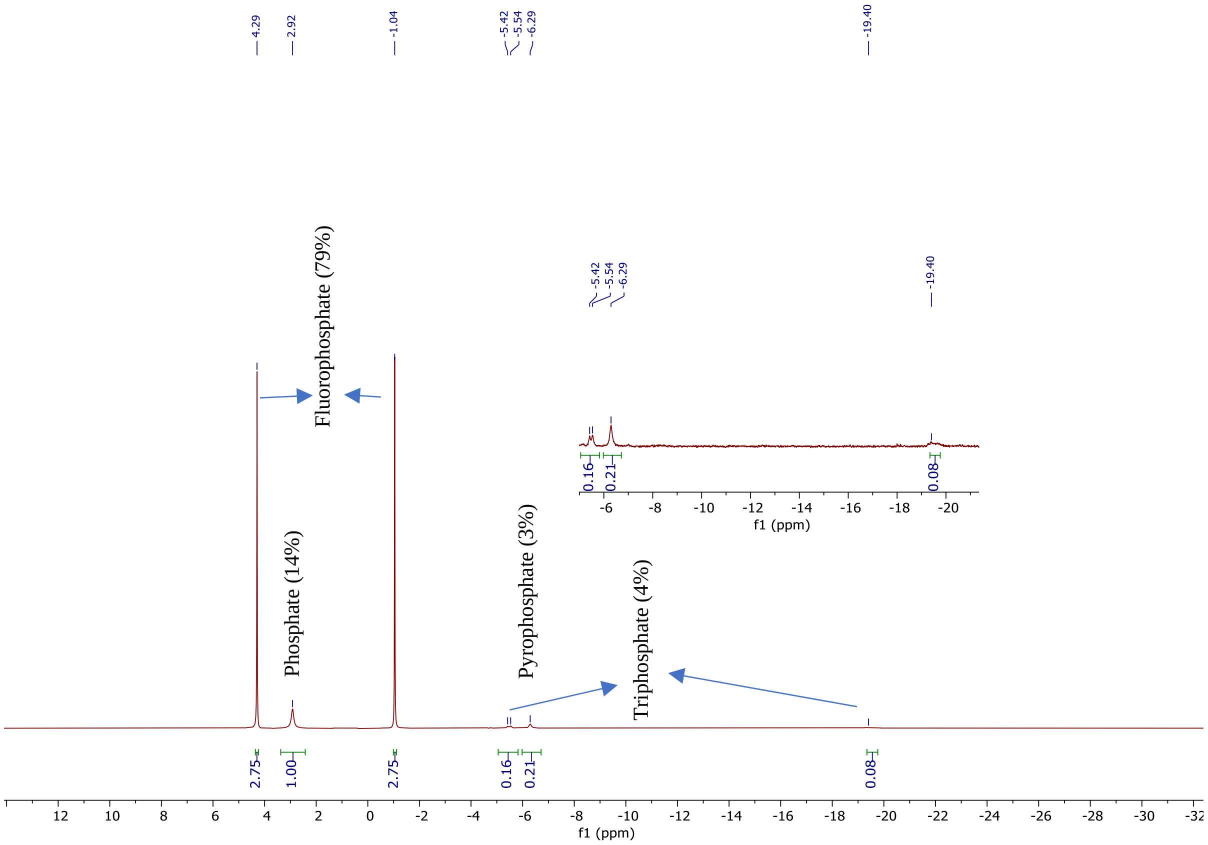

Figure S13. Quantitative ^{31}P NMR spectrum (162 MHz, 16 scans at 40 s recycle delay) of an aliquot of PTFE-mix^{PF} in D_2O (10 atom% D) indicates phosphorus content of fluorophosphate (δ_P = 1.5, (d, $^1J_{PF}$ = 866 Hz), 79%), phosphate (δ_P = 2.9, 14%), pyrophosphate (δ_P = −6.3, 3%), and triphosphate (δ_P = −5.5 (d, $^2J_{PP}$ = 20 Hz), −19.4 (t, $^2J_{PP}$ = 20 Hz), 4%).

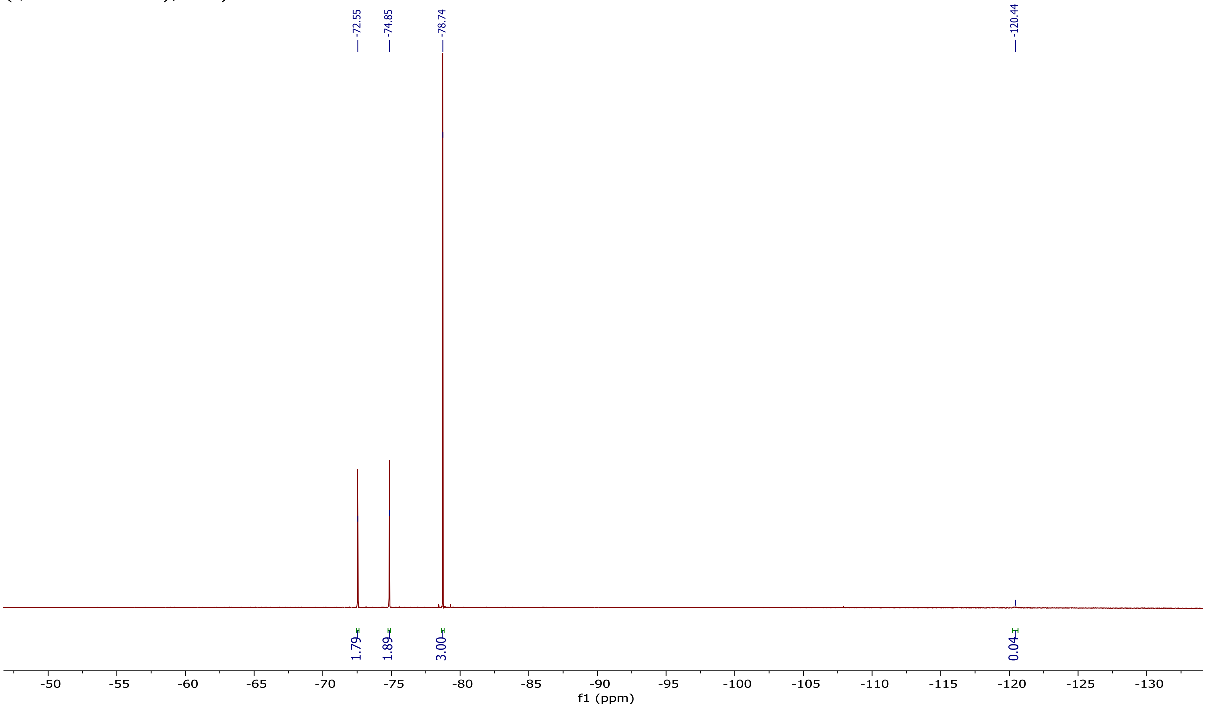

Figure S14. Quantitative ^{19}F NMR spectrum (377 MHz, 16 scans at 10 s recycle delay) of an aliquot of PTFE-mix^{PF} in D_2O (10 atom% D) using sodium triflate as an internal standard, indicates fluorine content of fluoride (δ_F= 120, 1%), fluorophosphate (δ_P = −73.7 (d, $^1J_{PF}$ = 867 Hz), 99%).

4.3 Powder X-ray diffraction of PTFE-mix

To a 15 mL stainless-steel milling jar was added two hardened chrome steel bearings (2 × 7 g), PTFE (1 equiv.) and K_3PO_4 (1.25 equiv./F) or $K_4P_2O_7$ (0.625 equiv./F). The total loading of material in the jar (PTFE and activator) was kept constant at 500 mg. The jar was closed and securely fitted to the mill which was set for 3 h at a frequency of 35 Hz. Upon completion, the jar was opened and the powder was collected and analysed by PXRD.

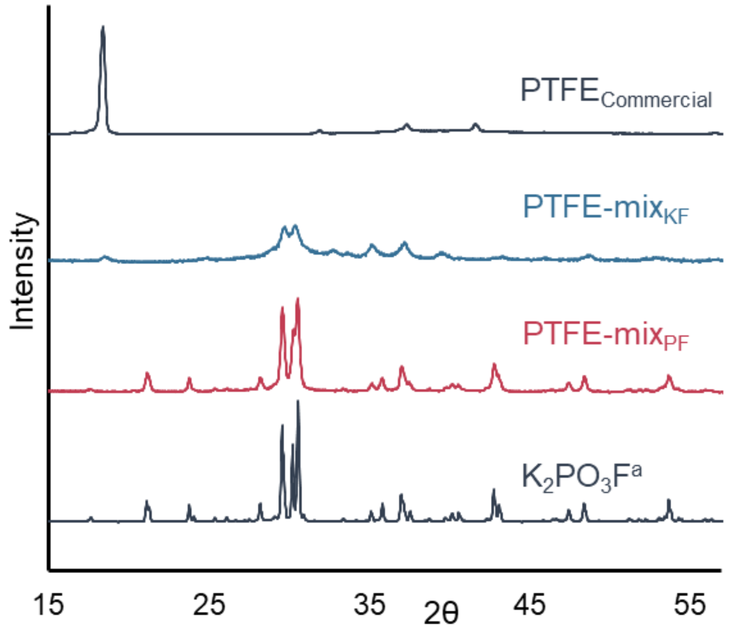

Figure S15. X-ray powder diffraction data of PTFE, PTFE-mix^{KF}, PTFE-mix^{PF}, K_2PO_3F (top to bottom). [a] K_2PO_3F was synthesised quantitatively by milling equimolar amounts of KF and KPO_3 (see below).

To a 15 mL stainless-steel milling jar was added two hardened chrome steel bearings (2 × 7 g), KF (1 equiv.) and KPO_3 (1.00 equiv.). The total loading of material in the jar (KF and KPO_3) was kept constant at 1000 mg. The jar was closed and securely fitted to the mill which was set for 3 h at a frequency of 35 Hz. Upon completion, the jar was opened and the powder was collected and analysed by PXRD.

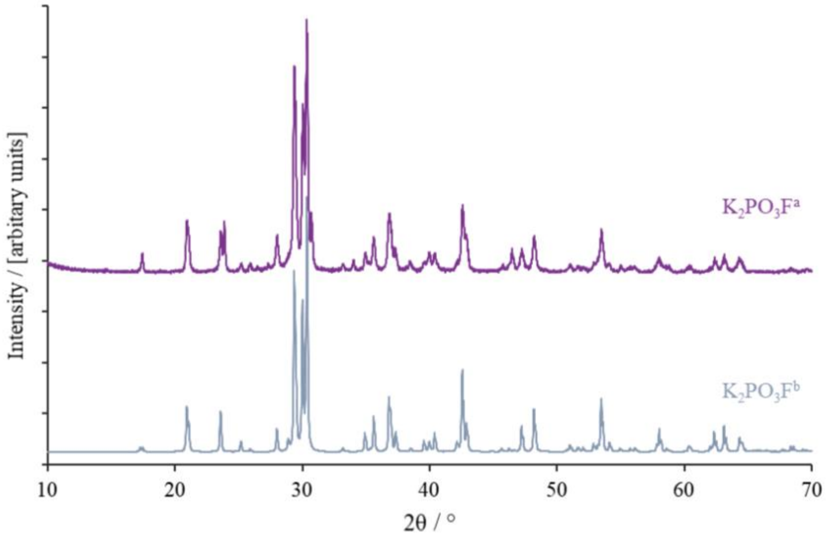

Figure S16. [a] X-ray powder diffraction data of mechanochemically synthesised K_2PO_3F according to experimental procedure above. [b] X-ray powder diffraction pattern simulated from single crystal X-ray diffraction data, ICSD collection code 15691 [55]. Powder diffraction pattern generated in Mercury 2022.1.0 with the FWHM set to 0.1 2θ, using Cu Ka1 and Ka2 wavelength radiations in a 2:1 ratio (1.54056 Å and 1.54439 Å, respectively).

4.4 Solid-state NMR spectroscopy of PTFE-mix

To a 15 mL stainless-steel milling jar was added two chrome steel bearings (2 × 7 g), PTFE (1 equiv.) and K_3PO_4 (1.25 equiv./F) or $K_4P_2O_7$ (0.625 equiv./F). The total loading of material in the jar (PTFE and activator) was kept constant at 500 mg. The jar was closed and securely fitted to the mill which was set for 3 h at a frequency of 35 Hz. Upon completion, the jar was opened and the powder was collected and analysed by solid-state NMR spectroscopy.

^{19}F DPMAS SS NMR spectra were recorded on samples of PTFE-mix^{KF} and PTFE-mix^{PF} directly after mechanochemical activation revealed the emergence of new resonances. The ^{19}F DPMAS SS NMR spectra of PTFE-mix^{KF} showed two broad resonances at $\delta_F = -122.0$ and -132.1 ppm. An independently recorded ^{19}F DPMAS spectra was acquired on a sample of KF which revealed sharp resonances at $\delta_F = -122.6$ and -132.9 ppm, matching well with the literature reported values for KF·2H_2O and anhydrous KF of $\delta_F = -123$ and -133 ppm. [56] The spectra show that after milling the mixture contains highly amorphous KF, with both resonances experiencing a large degree of broadening and intensity in the sidebands. Due to the intensity of the sidebands, the fluorophosphate region is obscured in this sample irrespective of ν_{rot}.

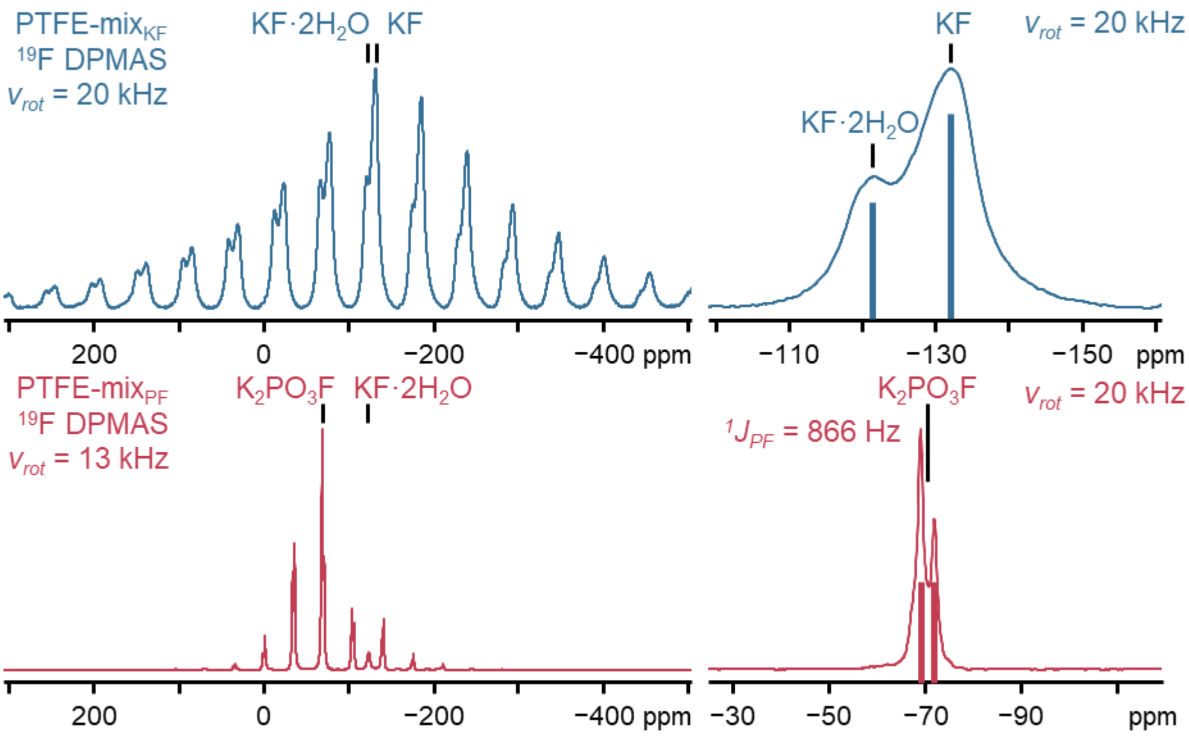

Figure S17. ^{19}F DPMAS SS NMR spectra of PTFE-mix^{KF} (Blue, Top Left), and PTFE-mix^{PF} (Red, Bottom Left) recorded at a spinning speed of ν_{rot} = 20 kHz or 13 kHz respectively. An expanded view of the major isotropic resonance is shown for PTFE-mix^{KF} (Blue, Top Right), and PTFE-mix^{PF} (Red, Bottom Right) recorded at a spinning speed of ν_{rot} = 20 kHz. Isotropic resonances are denoted by a vertical line |.

The ^{19}F DPMAS SS NMR spectra of PTFE-mix^{PF} revealed a different distribution of fluorine post-milling. Two resonances were observed at $\delta_F = -70.7$ and -124.1 ppm. The latter can be attributed to the formation of the dihydrate of KF, KF·2H_2O, as seen in PTFE-mix^{KF}. The formation of a potassium salt of F^- is in line with the small quantity of aqueous F^- observed by solution phase quantitative ^{19}F NMR spectroscopy of the mixture (1 %). The second resonance observed at $\delta_F = -70.7$ is observed at a similar chemical shift to the doublet resonance observed by solution phase quantitative ^{19}F NMR spectroscopy of the mixture ($\delta_F = -73.7$ ppm, $^1J_{PF}$ = 866 Hz), and is also observed as a doublet resonance with a coupling constant of $^1J_{PF}$ = 866 Hz. Performing $^{19}F\{^{31}P\}$ SS NMR spectra with high-power decoupling resolves this doublet to a singlet resonance. This resonance can be assigned as the potassium salt of the fluorophosphate anion observed in solution: K_2PO_3F. This assignment is supported by the observation of K_2PO_3F reflections by PXRD.

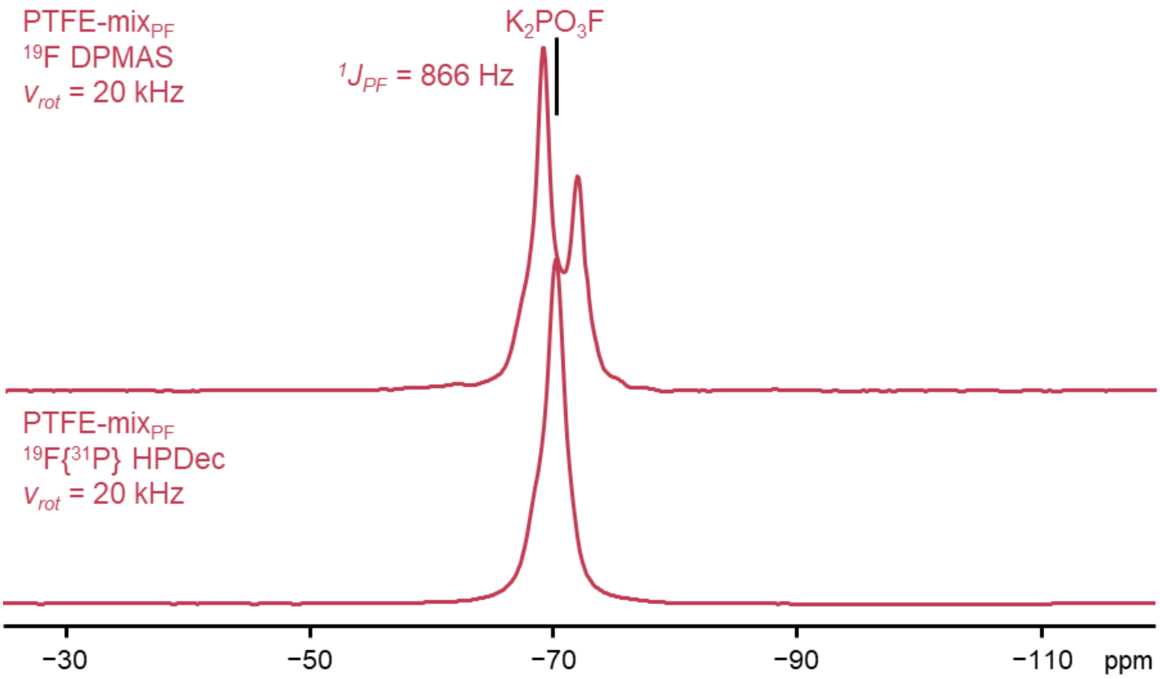

Figure S18. ^{19}F SS NMR spectra of PTFE-mix^{PF} (Top) and ^{19}F{^{31}P} SS NMR spectra of PTFE-mix^{PF} (Bottom). ^{31}P{^{19}F} CPMAS experiments were performed on samples of PTFE-mix^{KF} and PTFE-mix^{PF} directly after mechanochemical activation. The ^{31}P{^{19}F} CPMAS SS NMR spectra of PTFE-mix^{KF} showed a single major contribution at δ_P = 6.3 ppm, featuring a shoulder upfield. Further ^{31}P{^{19}F} spectra recorded with high power decoupling revealed this shoulder in more detail as a broad resonance with maximum intensities at $\delta_P \approx 0.3$, −3.2 ppm. The appearance of this resonance can be explained by the presence of $K_4P_2O_7$, which is observed as a group of 5 sharp isotropic resonances between δ_P = 1.4 and −4.0 when crystalline. [57] Milling of the sample causes broadening of these resonances into a single broad feature.

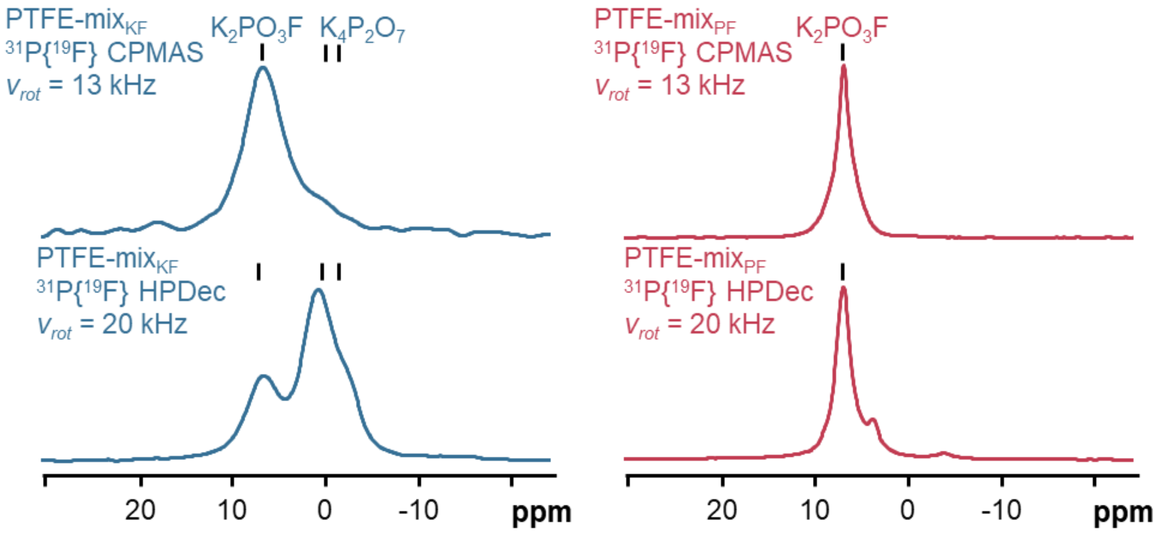

Figure S19. ^{31}P{^{19}F} DPMAS and CPMAS SS NMR spectra of PTFE^{-mixKF} (Blue, Top Left and Bottom Left respectively), and PTFE-mix^{PF} (Red, Top Right and Bottom Right respectively) recorded at a spinning speed of ν_{rot} = 20 kHz between δ_P = 30 – −25 ppm, no isotropic resonances were observed outside of this range. ^{31}P{^{19}F} CPMAS SS NMR spectra of PTFE-mix^{PF} show a single resonance at δ_P = 6.4 ppm, in line with the large quantity of K_2PO_3F observed by PXRD and solution phase NMR spectroscopy. A ^{31}P{^{19}F} spectrum recorded with high power decoupling showed the expected decrease in intensity of the K_2PO_3F and the observation of a resonance at δ_P = 3.2 ppm which has been attributed to the formation of an orthophosphate salt.

Multinuclear SS NMR spectroscopy revealed that PTFE-mix^{KF} and PTFE-mix^{PF} obtained directly after milling show a different composition, with the major fluorine content being identified as KF (and its dihydrate) and

K_2PO_3F, respectively. The formation of $K_4P_2O_7$ is observed in PTFE-mix^{KF}, whereas the major phosphorus containing component in PTFE-mix^{PF} is K_2PO_3F.

Water-insoluble carbon black, isolated by centrifugation after the degradation of PTFE, was collected and dried *in vacuo*. As the carbon black has previously been shown to contain graphitic moieties by Raman spectroscopy, Figures S7 and S11, the carbon black was diluted by co-grinding with $MgSO_4$. Dilution of the sample was performed to counter the tuning difficulties when recording spectra on rapidly rotating conductive solids. [58]
The spectra are shown in Figure S20. In both cases, a single broad line-shape was observed irrespective of decoupling nuclei, with minimal changes to the line-shape decoupling from either ^{1}H or ^{19}F. The similarity of the line shape on both spectra suggests that the carbon black generated does not closely incorporate ^{1}H or ^{19}F nuclei, and in both cases the broad resonance is centered at δ_C = 113.6 ppm. The central chemical shift and high anisotropy is consistent with the presence of graphitic carbon, with the anisotropy being associated with magnetic susceptibility and is not fully averaged by MAS conditions. [59]

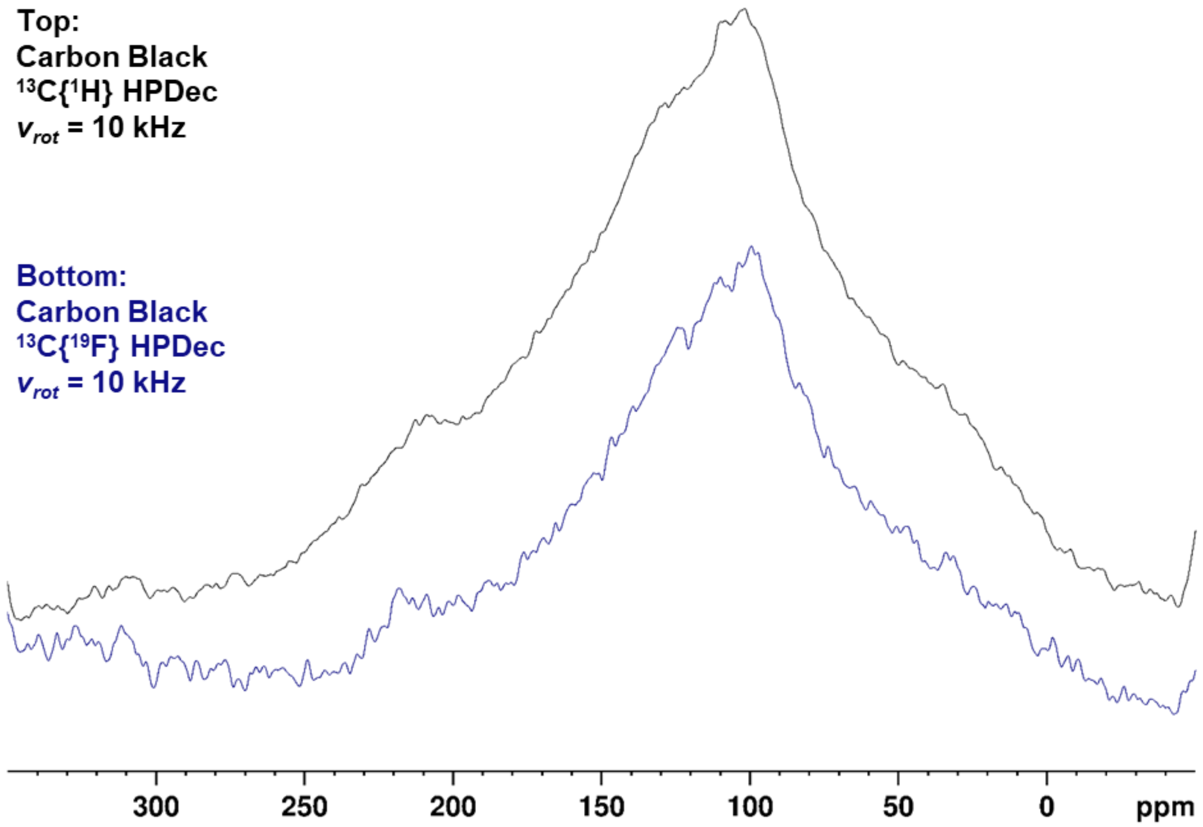

Figure S20. $^{13}C\{^{1}H\}$ (Top, Black) and $^{13}C\{^{19}F\}$ (Bottom, Blue) spectra recorded with HPDec at a spinning speed of ν_{rot} = 10 kHz.

5. Control experiments

Control experiments were carried out under ball milling conditions (15 mL jar, 2 × 7 g balls, 35 Hz, 3 h and 500 mg total material loading). Upon completion, the jar was opened and an aliquot of the powder (30-70 mg) and sodium triflate (10 mg, as internal standard) was extracted with D_2O (10 atom% D), centrifugated for 15-30 min and analysed by quantitative ^{19}F-NMR or ^{31}P-NMR spectroscopy. These experiments revealed the following key observations: i) K_3PO_4 was stable under ball milling conditions, while $K_4P_2O_7$ gave rise to 3% phosphate and 2% triphosphate, respectively. ii) K_2PO_3F could not be generated from KF upon ball milling with either K_3PO_4 or $K_4P_2O_7$. iii) K_2PO_3F readily decomposed into KF when milled with K_3PO_4 and $K_4P_2O_7$. iv) The by-products K_2CO_3, $K_2C_2O_4$, and K_2PO_3F formed upon mechanochemical disassembly of PTFE under ball milling conditions were themselves competent activators of PTFE.

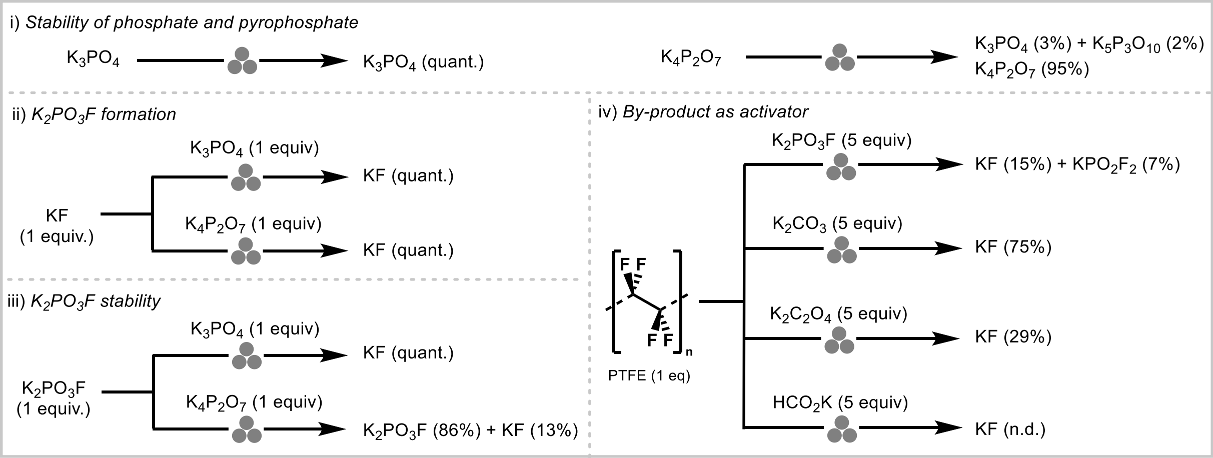

Figure S21. Control experiments performed under mechanochemical conditions. All reactions were performed in 15 mL stainless steel jars (500 mg total material loading) loaded with 2 × 7 g hardened chrome steel bearings and milled for 3 h at 35 Hz.

6. Isolation of KF, TMAF, TMAF(*t*AmOH), and TBAF(*t*BuOH)$_4$

6.1 Isolation of KF from PFAS-mix^{KF}

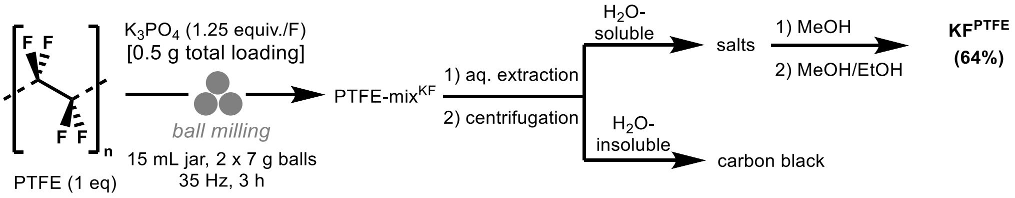

To six 15 mL stainless-steel milling jars was added two chrome steel balls (2 × 7 g), PTFE (1 equiv., 43 mg) and K_3PO_4 (5.0 equiv., 457 mg) each. The total material of each jar (PTFE and K_3PO_4) was kept constant at 500 mg. The jars were closed and securely fitted to a mill which was set for 3 h at a frequency of 35 Hz. Upon completion, the jars were opened, the powder was collected and extracted with H_2O (60 mL). The resulting suspension was centrifuged for 30 min to eliminate water-insoluble carbon black. The resulting clear supernatant was decanted and concentrated under reduced pressure to obtain a solid residue, which was sonicated with MeOH (40 mL) for 30 min and centrifugated for another 30 min. The clear supernatant was decanted and concentrated under reduced pressure to give crude KF. For further purification, the crude product was extracted with a mixture MeOH (6 mL) and EtOH (2 mL), sonicated for 10 min, and centrifugated for 30 min leading to the precipitation of insoluble salts. The clear supernatant was decanted and concentrated under reduced pressure to afford KF (408 mg) in 64% yield (93% purity determined by quantitative ^{19}F NMR spectroscopy).

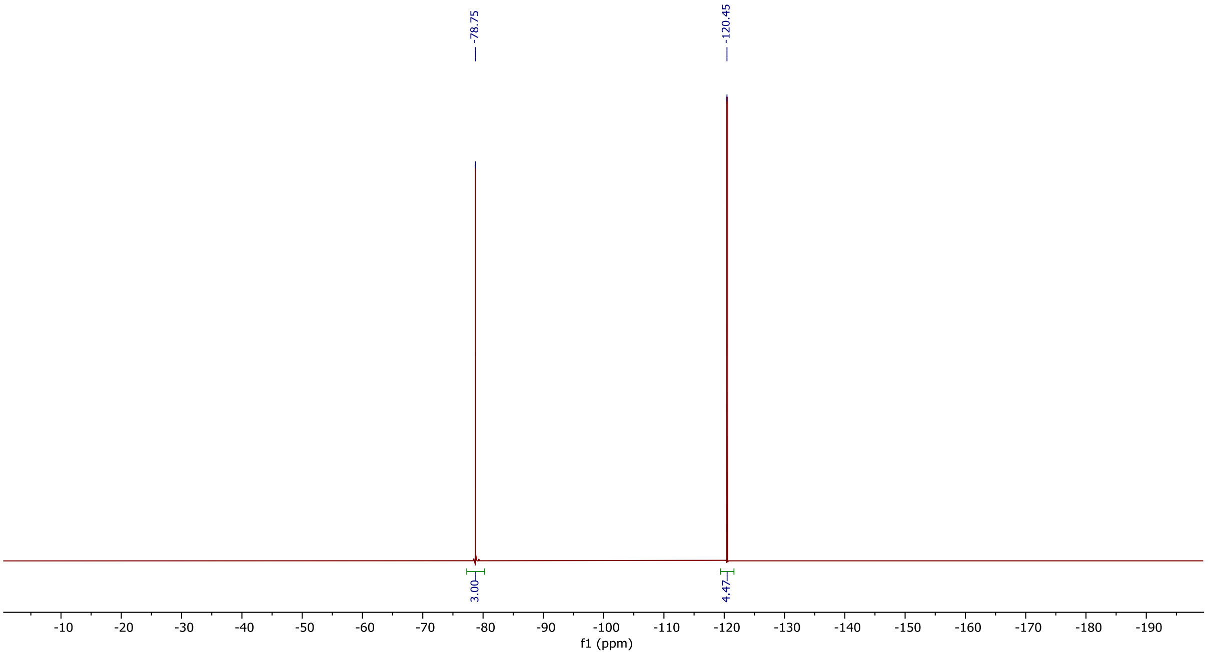

Figure S22. Quantitative ^{19}F NMR spectrum (377 MHz) of 16.3 mg KF^{PTFE} and 10.0 mg NaOTf (as an internal standard) in D_2O (10 atom% D).

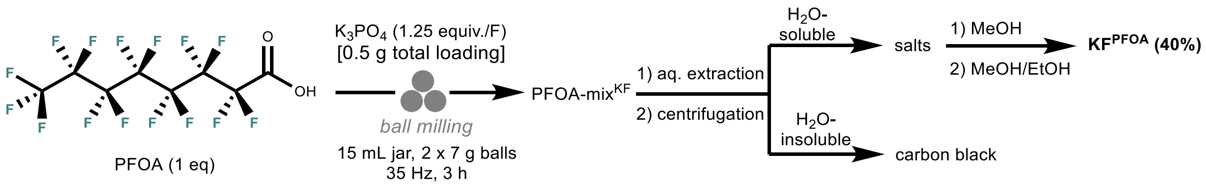

To six 15 mL stainless-steel milling jars was added two chrome steel balls (2 × 7 g), PFOA (1 equiv., 47.8 mg) and K_3PO_4 (1.25 equiv./F, 452.2 mg) each. The total material of each jar (PFOA and K_3PO_4) was kept constant at 500 mg. The jars were closed and securely fitted to a mill which was set for 3 h at a frequency of 35 Hz. Upon completion, the jars were opened, the powder was collected and extracted with H_2O (60 mL). The resulting suspension was centrifuged for 30 min. The resulting supernatant was decanted and concentrated under reduced pressure to obtain a solid residue, which was sonicated with MeOH (40 mL) for 30 min and centrifugated for another 30 min. The clear supernatant was decanted and concentrated under reduced pressure to give crude KF.

For further purification, the crude product was extracted with a mixture MeOH (6 mL) and EtOH (2 mL), sonicated for 30 min, and centrifugated for 30 min. The clear supernatant was decanted and concentrated under reduced pressure. The crude product was further extracted with a mixture of MeOH (5 mL) and EtOH (2 mL), sonicated for 30 min, and centrifugated for 30 min to afford KF (267 mg) in 40% yield (89% purity determined by quantitative ^{19}F NMR spectroscopy).

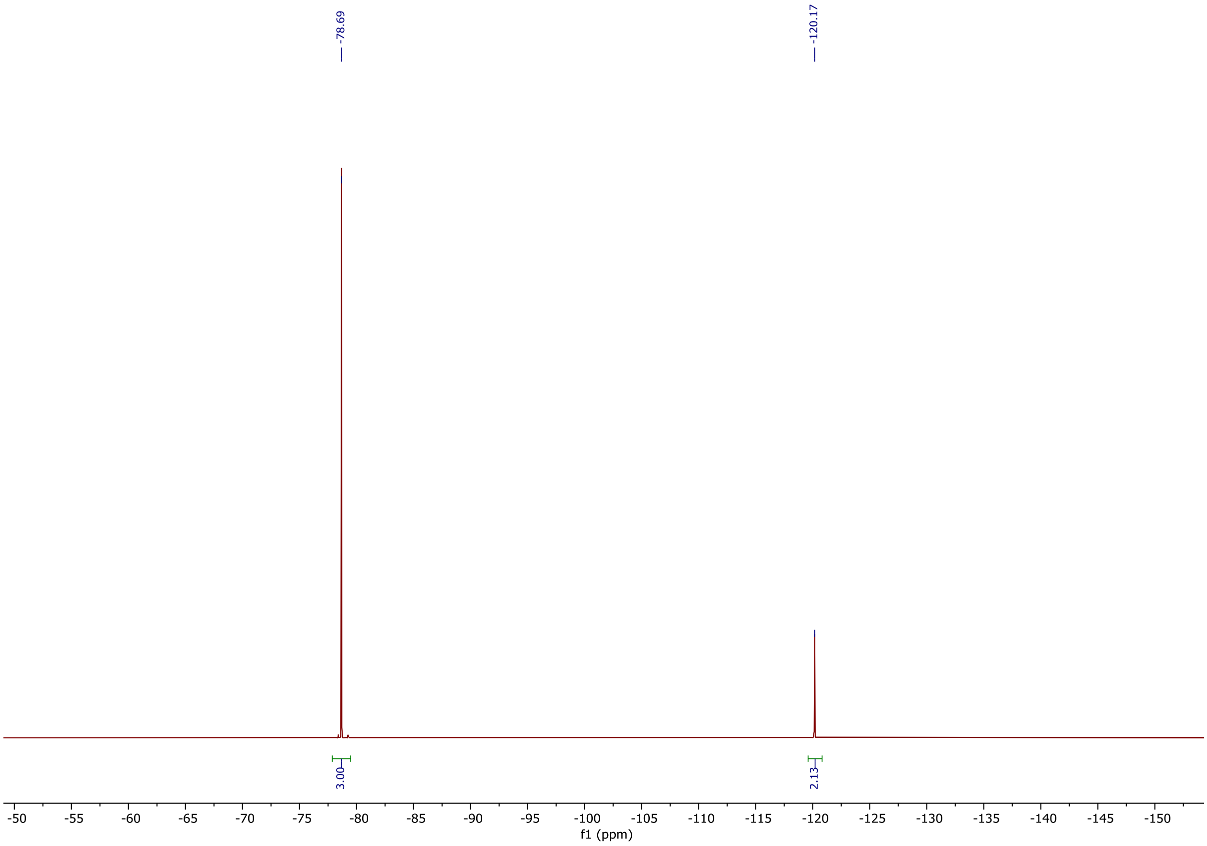

Figure S23. Quantitative ^{19}F NMR spectrum (377 MHz) of 12.0 mg isolated KF^{PFOA} and 15.0 mg NaOTf (as an internal standard) in D_2O (10 atom% D).

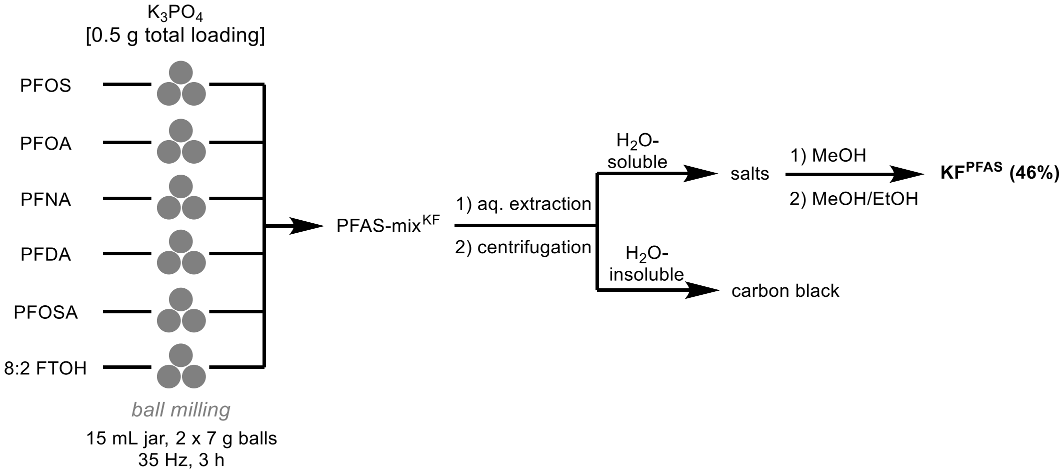

To six 15 mL stainless-steel milling jars was added two chrome steel balls (2 × 7 g), PFAS (1 equiv.) and K_3PO_4 (1.25 or 2.0 equiv./F) each (see table below). The total material of each jar (PFAS and K_3PO_4) was kept constant at 500 mg. The jars were closed and securely fitted to a mill which was set for 3 h at a frequency of 35 Hz. Upon completion, the jars were opened, the powders were collected, mixed and extracted with H_2O (60 mL). The resulting suspension was centrifuged for 30 min. The resulting supernatant was decanted and concentrated under reduced pressure to obtain a solid residue, which was sonicated with MeOH (40 mL) for 30 min and centrifugated for another 30 min. The clear supernatant was decanted and concentrated under reduced pressure to give crude KF. For further purification, the crude product was extracted with a mixture MeOH (6 mL) and EtOH (2 mL), sonicated for 30 min, and centrifugated for 30 min. The clear supernatant was decanted and concentrated under

reduced pressure. The crude product was further extracted with a mixture of MeOH (5 mL) and EtOH (2 mL), sonicated for 30 min, and centrifugated for 30 min to afford KF (302 mg) in 46% yield (84% purity determined by quantitative ^{19}F NMR spectroscopy).

Table S15. PFAS and K_3PO_4 added to each jar for isolation of KF.

Jar	PFAS	phosphate salt
1	PFOS (1 equiv., 32.4 mg)	K_3PO_4 (2.0 equiv./F, 467.6 mg)
2	PFOA (1 equiv., 47.1 mg)	K_3PO_4 (1.25 equiv./F, 452.2 mg)
3	PFNA (1 equiv., 46.7 mg)	K_3PO_4 (1.25 equiv./F, 453.3 mg)
4	PFDA (1 equiv., 46.3 mg)	K_3PO_4 (1.25 equiv./F, 453.7 mg)
5	PFOSA (1 equiv., 49.8 mg)	K_3PO_4 (1.25 equiv./F, 450.2 mg)
6	8:2 FTOH (1 equiv., 46.6 mg)	K_3PO_4 (1.25 equiv./F, 453.4 mg)

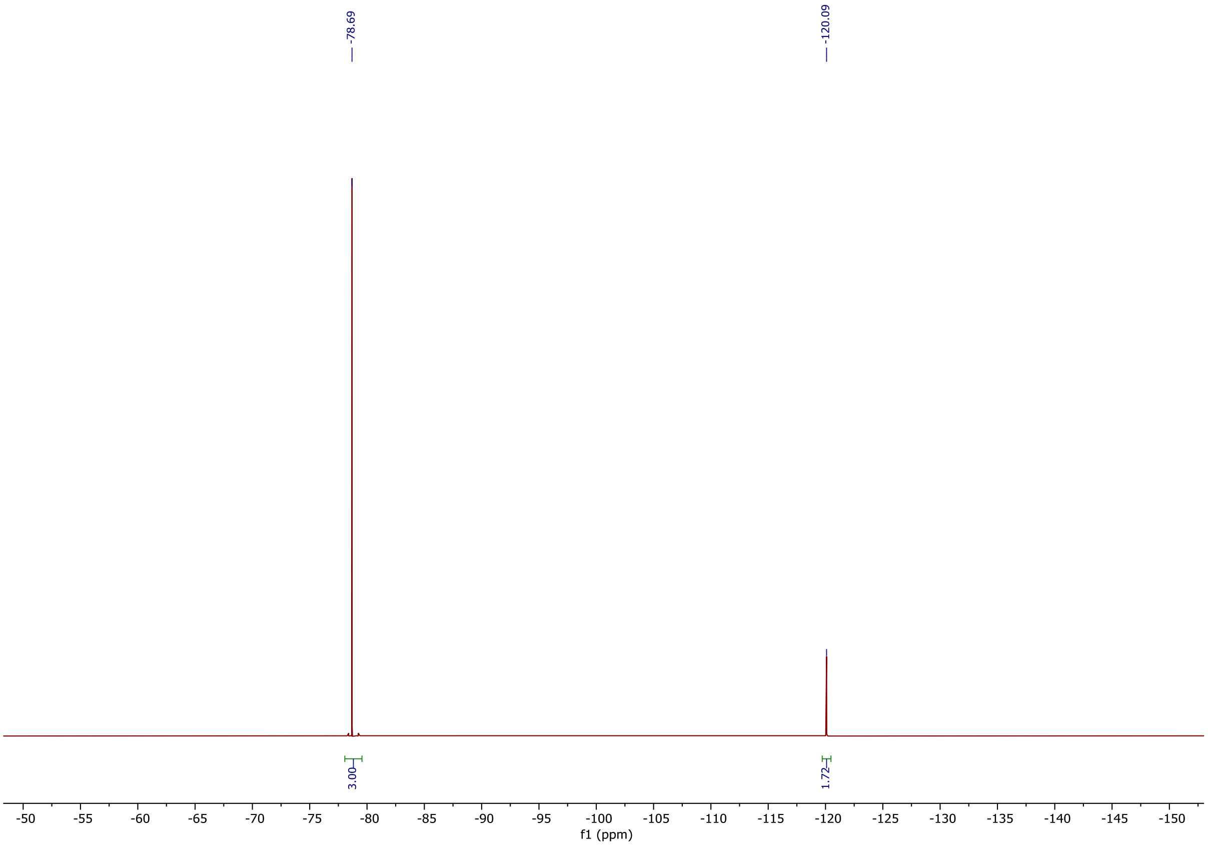

Figure S24. Quantitative ^{19}F NMR spectrum (377 MHz) of 13.0 mg isolated KF^{PFAS} and 18.9 mg NaOTf (as an internal standard) in D_2O (10 atom% D).

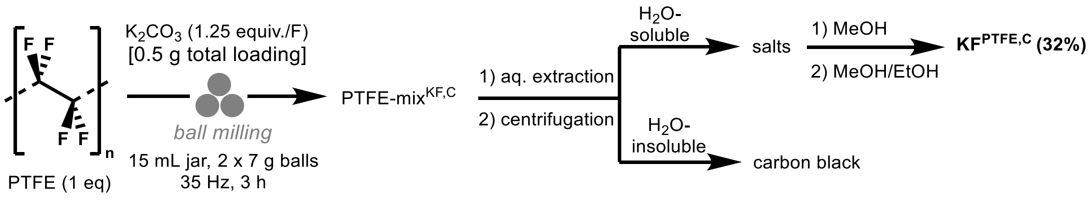

To a 15 mL stainless-steel milling jars was added two chrome steel balls (2 × 7 g), PTFE (1 equiv., 63 mg) and K_2CO_3 (5.0 equiv., 437 mg). The total material of each jar (PTFE and K_3PO_4) was kept constant at 500 mg. The jar was closed and securely fitted to a mill which was set for 3 h at a frequency of 35 Hz. Upon completion, the jar was opened, the powder was collected and extracted with H_2O (10 mL). The resulting suspension was centrifuged for 30 min to eliminate water-insoluble carbon black. The resulting clear supernatant was decanted and concentrated under reduced pressure to obtain a solid residue, which was sonicated with MeOH (6 mL) for 30 min and centrifugated for another 30 min. The clear supernatant was decanted and concentrated under reduced pressure to give crude KF. For further purification, the crude product was extracted with a mixture MeOH (2 mL) and EtOH (0.5 mL), sonicated for 10 min, and centrifugated for 30 min leading to the precipitation of insoluble salts. The clear supernatant was decanted and concentrated under reduced pressure to afford KF (95 mg) in 32% yield (55% purity determined by quantitative ^{19}F NMR spectroscopy).

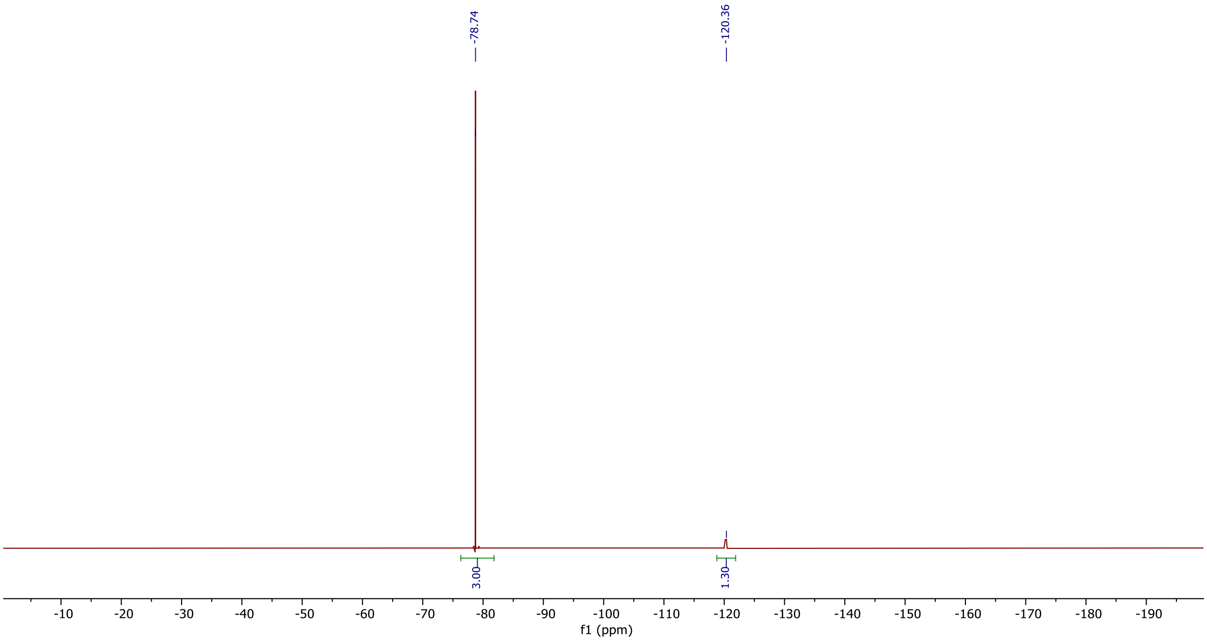

Figure S25. Quantitative ^{19}F NMR spectrum (377 MHz) of 8.0 mg KF^{PTFE}_C and 10.0 mg NaOTf (as an internal standard) in D_2O (10 atom% D).

PTFE tape

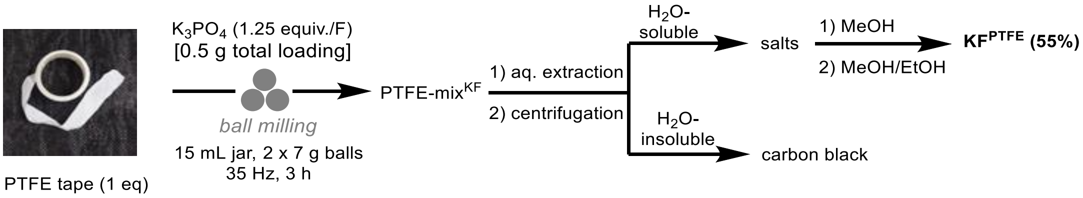

PTFE tape was cut into pieces with scissors before milling. To six 15 mL stainless-steel milling jars was added two chrome steel balls (2 × 7 g), PTFE tape (1 equiv., 43 mg) and K_3PO_4 (5.0 equiv., 457 mg) each. The total material of each jar (PTFE and K_3PO_4) was kept constant at 500 mg. The jars were closed and securely fitted to a mill which was set for 3 h at a frequency of 35 Hz. Upon completion, the jars were opened, the powder was collected and extracted with H_2O (60 mL). The resulting suspension was centrifuged for 30 min to eliminate water-insoluble carbon black. The resulting clear supernatant was decanted and concentrated under reduced pressure to obtain a solid residue, which was sonicated with MeOH (40 mL) for 30 min and centrifugated for another 20 min. The clear supernatant was decanted and concentrated under reduced pressure to give crude KF. The centrifugate was dissolved in MeOH (40 mL) and stirred at 40 °C for 16 hours. The resulting suspension was centrifuged for 20 min. The clear supernatant was decanted and concentrated under reduced pressure to give crude KF. The crude KF obtained from both steps were combined for further purification. The crude product was extracted with a mixture MeOH (6 mL) and EtOH (2 mL), sonicated for 10 min, and centrifugated for 20 min leading to the precipitation of insoluble salts. The clear supernatant was decanted and concentrated under reduced pressure. The obtained solid was again extracted with a mixture MeOH (6 mL) and EtOH (2 mL), sonicated for 10 min, and centrifugated for 20 min. The clear supernatant was decanted and concentrated under reduced pressure to afford KF (332 mg) in 55% yield (96% purity determined by quantitative ^{19}F NMR spectroscopy).

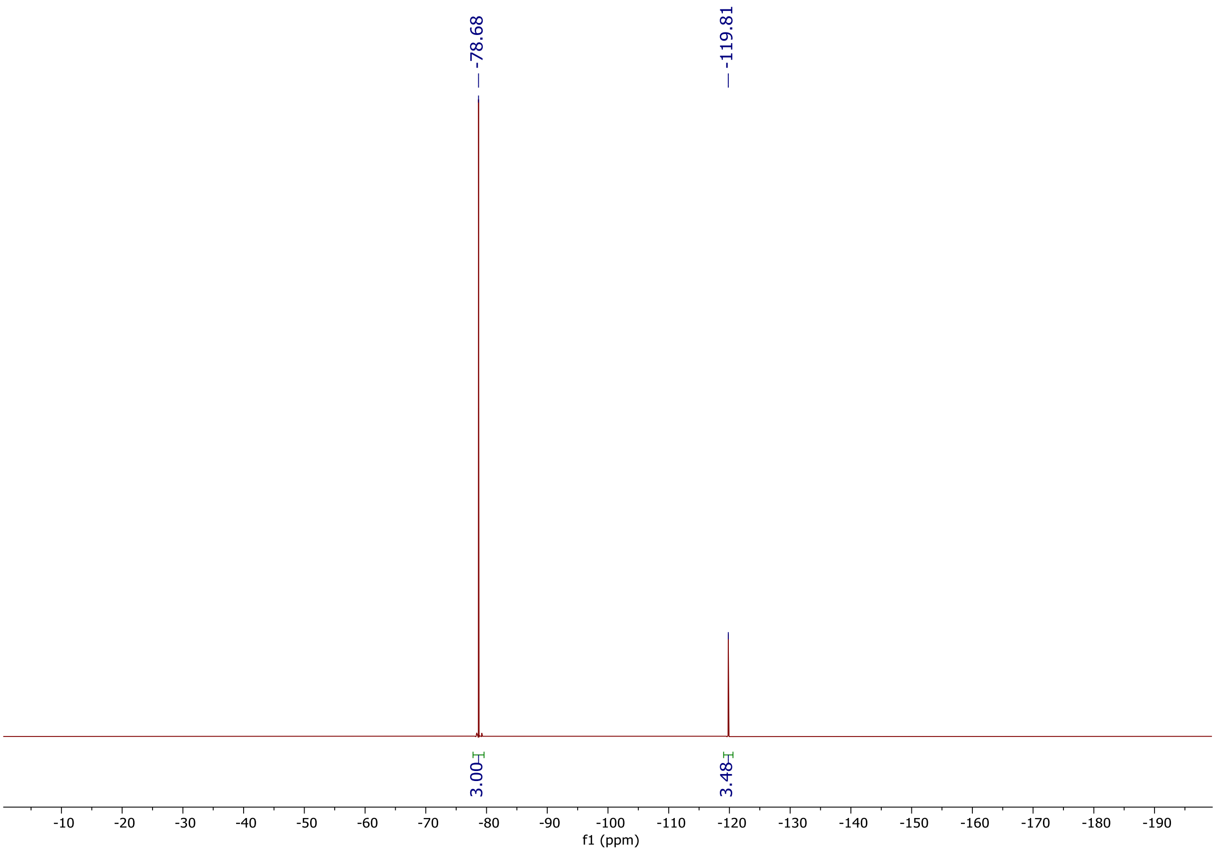

Figure S26. Quantitative ^{19}F NMR spectrum (377 MHz) of 12.4 mg KF^{PTFE} and 10.1 mg NaOTf (as an internal standard) in D_2O (10 atom% D).

ETFE wire

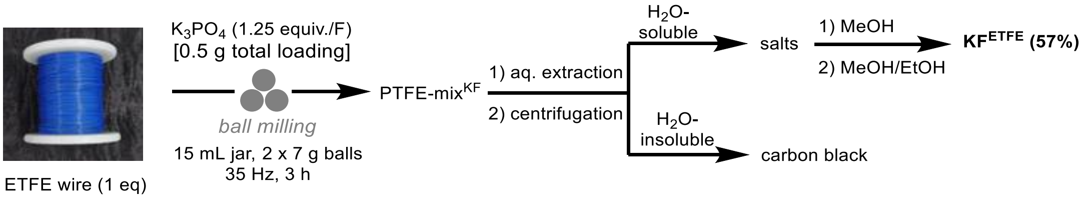

ETFE wire was cut into pieces with scissors and the metal core was removed. To six 15 mL stainless-steel milling jars was added two chrome steel balls (2 × 7 g), ETFE wire (1 equiv., 54 mg) and K_3PO_4 (5.0 equiv., 446 mg) each. The total material of each jar (PTFE and K_3PO_4) was kept constant at 500 mg. The jars were closed and securely fitted to a mill which was set for 3 h at a frequency of 35 Hz. Upon completion, the jars were opened, the powder was collected and extracted with H_2O (60 mL). The resulting suspension was centrifuged for 30 min to eliminate water-insoluble carbon black. The resulting clear supernatant was decanted and concentrated under reduced pressure to obtain a solid residue, which was sonicated with MeOH (40 mL) for 30 min and centrifugated for another 20 min. The clear supernatant was decanted and concentrated under reduced pressure to give crude KF. The centrifugate was dissolved in MeOH (40 mL) and stirred at 40 °C for 16 hours. The resulting suspension was centrifuged for 20 min. The clear supernatant was decanted and concentrated under reduced pressure to give crude KF. The crude KF obtained from both steps were combined for further purification. The crude product was extracted with a mixture MeOH (6 mL) and EtOH (2 mL), sonicated for 10 min, and centrifugated for 20 min leading to the precipitation of insoluble salts. The clear supernatant was decanted and concentrated under reduced pressure. The obtained solid was again extracted with a mixture MeOH (6 mL) and EtOH (2 mL), sonicated for 10 min, and centrifugated for 20 min. The clear supernatant was decanted and concentrated under reduced pressure to afford KF (331 mg) in 57% yield (95% purity determined by quantitative ^{19}F NMR spectroscopy).

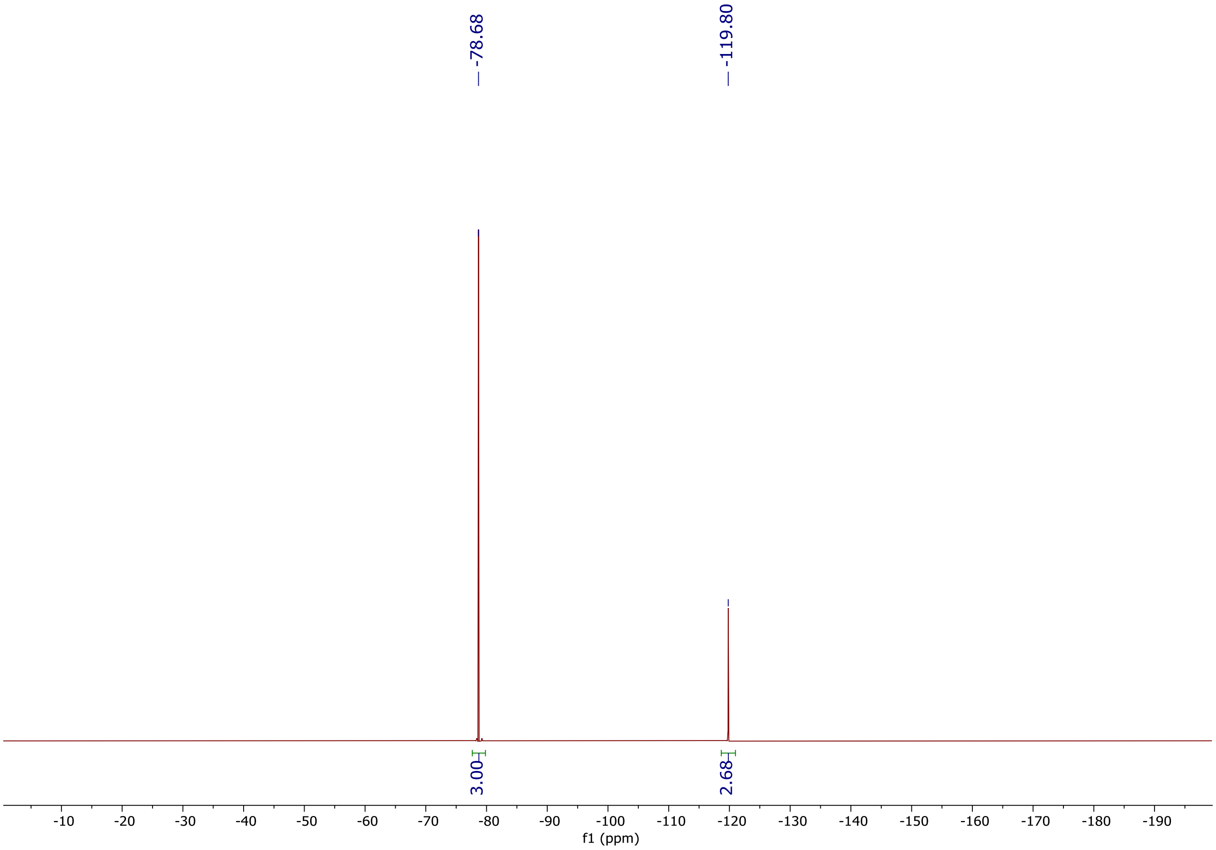

Figure S27. Quantitative ^{19}F NMR spectrum (377 MHz) of 12.0 mg KF^{ETFE} and 12.6 mg NaOTf (as an internal standard) in D_2O (10 atom% D).

PVDF fitting

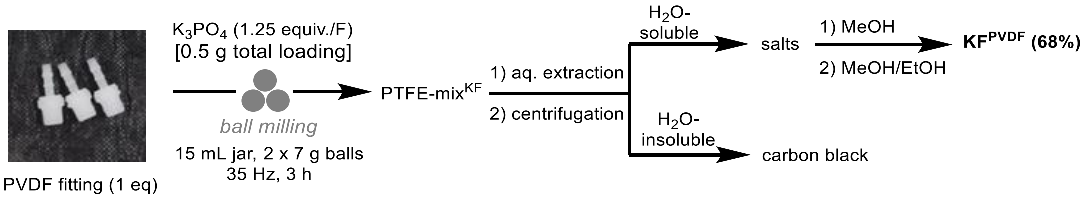

To a 15 mL stainless-steel milling jar was added one chrome steel ball (7 g) and a PVDF fitting. The jar was closed and securely fitted to a mill which was set for 3 h at a frequency of 35 Hz. Upon completion, the jar was opened and PVDF flakes were collected for further use. To three 15 mL stainless-steel milling jars was added two chrome steel balls (2 × 7 g), PVDF flakes (1 equiv., 54 mg) and K_3PO_4 (5.0 equiv., 446 mg) each. The total material of each jar (PTFE and K_3PO_4) was kept constant at 500 mg. The jars were closed and securely fitted to a mill which was set for 3 h at a frequency of 35 Hz. Upon completion, the jars were opened, the powder was collected and extracted with H_2O (30 mL). The resulting suspension was centrifuged for 30 min to eliminate water-insoluble carbon black. The resulting clear supernatant was decanted and concentrated under reduced pressure to obtain a solid residue, which was sonicated with MeOH (20 mL) for 30 min and centrifugated for another 20 min. The clear supernatant was decanted and concentrated under reduced pressure to give crude KF. The centrifugate was dissolved in MeOH (20 mL) and stirred at 40 °C for 16 hours. The resulting suspension was centrifuged for 20 min. The clear supernatant was decanted and concentrated under reduced pressure to give crude KF. The crude KF obtained from both steps were combined for further purification. The crude product was extracted with a mixture MeOH (3 mL) and EtOH (1 mL), sonicated for 10 min, and centrifugated for 20 min leading to the precipitation of insoluble salts. The clear supernatant was decanted and concentrated under reduced pressure. The obtained solid was again extracted with a mixture MeOH (3 mL) and EtOH (1 mL), sonicated for 10 min, and centrifugated for 20 min. The clear supernatant was decanted and concentrated under reduced pressure to afford KF (199 mg) in 68% yield (98% purity determined by quantitative ^{19}F NMR spectroscopy).

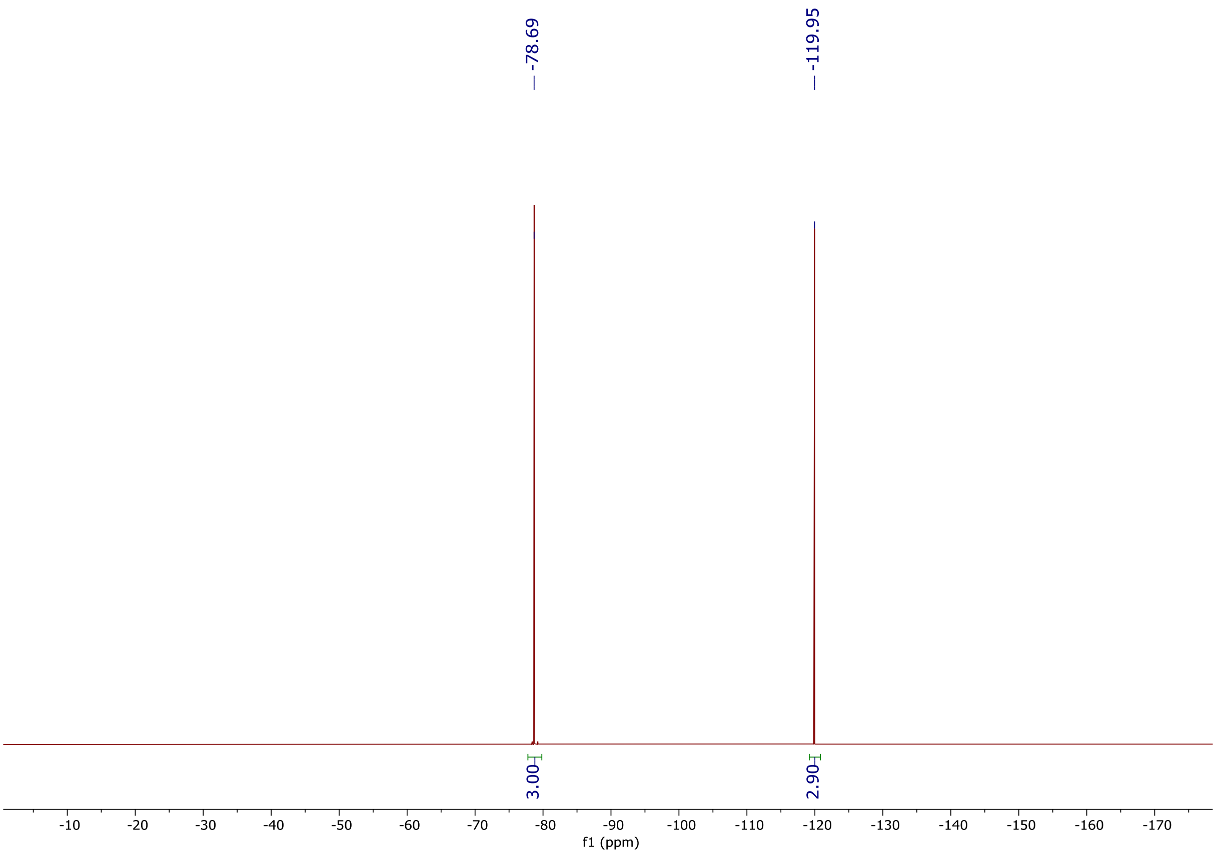

Figure S28. Quantitative ^{19}F NMR spectrum (377 MHz) of 14.6 mg KF^{PVDF} and 14.6 mg NaOTf (as an internal standard) in D_2O (10 atom% D).

FEP tubing

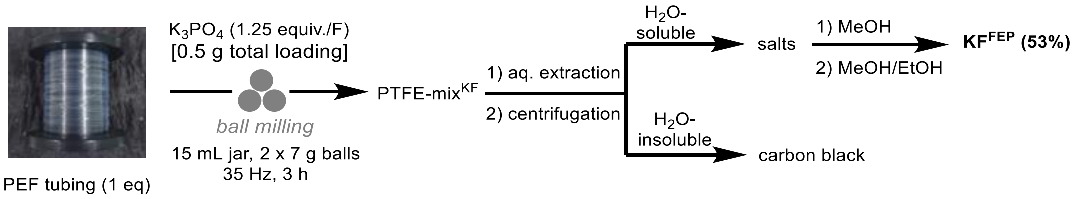

FEP tubing was cut into pieces with scissors before milling. To six 15 mL stainless-steel milling jars was added two chrome steel balls (2 × 7 g), FEP tubing (1 equiv., 43 mg) and K_3PO_4 (5.0 equiv., 457 mg) each. The total material of each jar (PTFE and K_3PO_4) was kept constant at 500 mg. The jars were closed and securely fitted to a mill which was set for 3 h at a frequency of 35 Hz. Upon completion, the jars were opened, the powder was collected and extracted with H_2O (60 mL). The resulting suspension was centrifuged for 30 min to eliminate water-insoluble carbon black. The resulting clear supernatant was decanted and concentrated under reduced pressure to obtain a solid residue, which was sonicated with MeOH (40 mL) for 30 min and centrifugated for another 30 min. The clear supernatant was decanted and concentrated under reduced pressure to give crude KF. The centrifugate was dissolved in MeOH (40 mL) and stirred at 40 °C for 16 hours. The resulting suspension was centrifuged for 20 min. The clear supernatant was decanted and concentrated under reduced pressure to give crude KF. The crude KF obtained from both steps were combined for further purification. The crude product was extracted with a mixture MeOH (6 mL) and EtOH (2 mL), sonicated for 10 min, and centrifugated for 20 min leading to the precipitation of insoluble salts. The clear supernatant was decanted and concentrated under reduced pressure. The obtained solid was again extracted with a mixture MeOH (6 mL) and EtOH (2 mL), sonicated for 10 min, and centrifugated for 20 min. The clear supernatant was decanted and concentrated under reduced pressure to afford KF (317 mg) in 53% yield (97% purity determined by quantitative ^{19}F NMR spectroscopy).

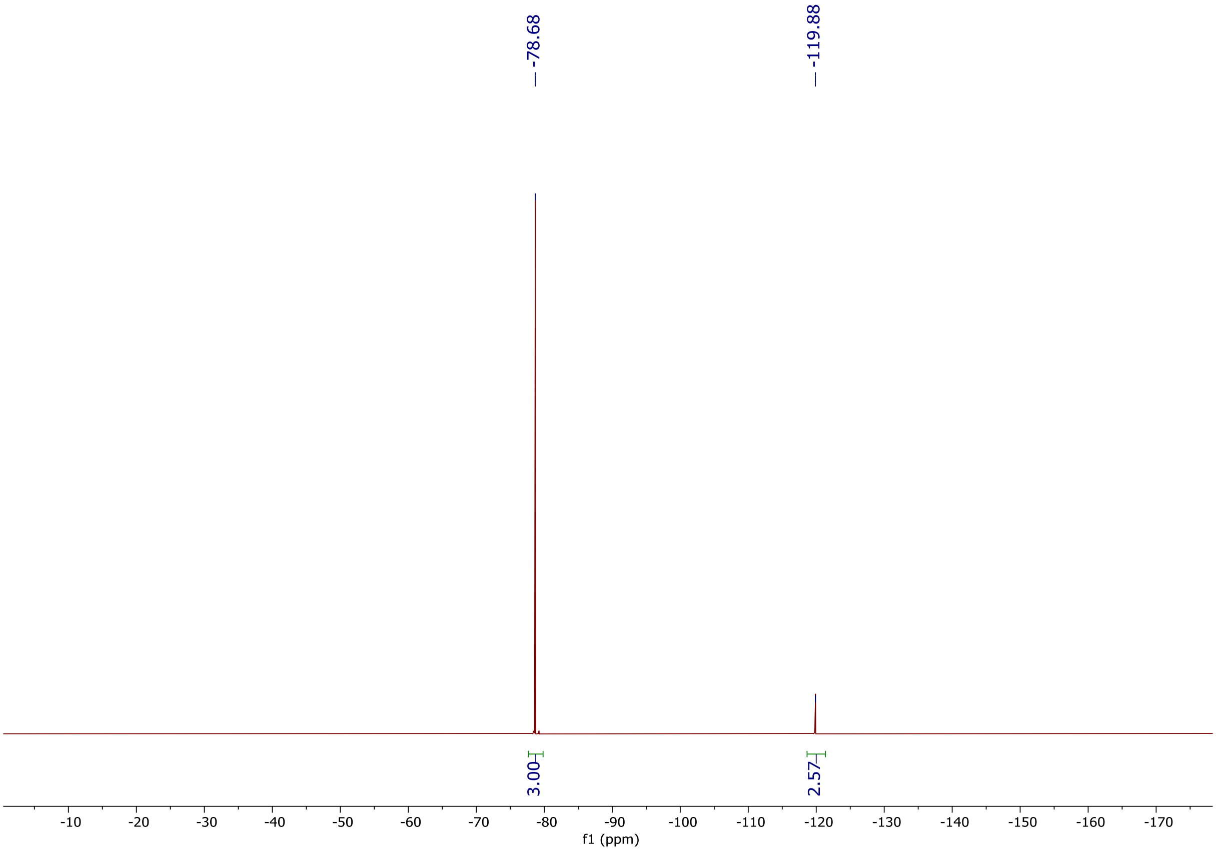

Figure S29. Quantitative ^{19}F NMR spectrum (377 MHz) of 10.9 mg KF^{FEP} and 12.2 mg NaOTf (as an internal standard) in D_2O (10 atom% D).

PFOA adsorbed Powdered Activated Carbon

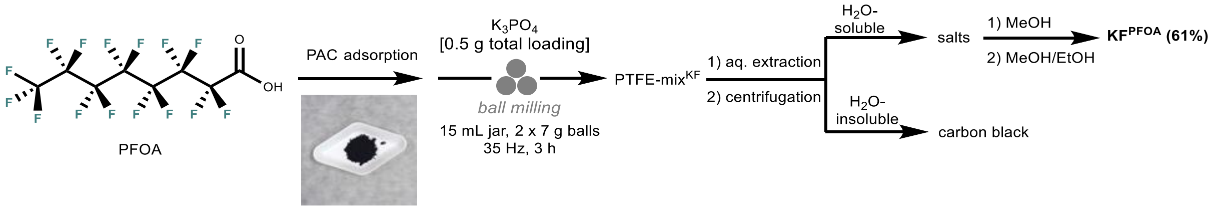

PFOA (200 mg) and Powered Activated Carbon (PAC) from Sigma Aldrich (400 mg) were added to a round-bottom flask and stirred in water (100 mL) at room temperature for 16 hours. The solid mixture was collected by vaccum filtration and dried under reduced pressure. To six 15 mL stainless-steel milling jars was added two chrome steel balls (2 × 7 g), PFOA adsorbed PAC (100 mg) and K_3PO_4 (400 mg) each. The total material of each jar (PFOA, PAC and K_3PO_4) was kept constant at 500 mg. The jars were closed and securely fitted to a mill which was set for 3 h at a frequency of 35 Hz. Upon completion, the jars were opened and a sample mixture was prepared for quantitative ^{19}F NMR analysis: 85% KF, 14% K_2PO_3F, total yield: quantitative.

The powder was collected and extracted with H_2O (60 mL). The resulting suspension was centrifuged for 30 min to eliminate water-insoluble carbon black. The resulting clear supernatant was decanted and concentrated under reduced pressure to obtain a solid residue, which was sonicated with MeOH (40 mL) for 30 min and centrifugated for another 30 min. The clear supernatant was decanted and concentrated under reduced pressure to give crude KF. The centrifugate was dissolved in MeOH (40 mL) and stirred at 40 °C for 16 hours. The resulting suspension was centrifuged for 20 min. The clear supernatant was decanted and concentrated under reduced pressure to give crude KF. The crude KF obtained from both steps were combined for further purification. The crude product was extracted with a mixture MeOH (6 mL) and EtOH (2 mL), sonicated for 10 min, and centrifugated for 20 min leading to the precipitation of insoluble salts. The clear supernatant was decanted and concentrated under reduced pressure. The obtained solid was again extracted with a mixture MeOH (6 mL) and EtOH (2 mL), sonicated for 10 min, and centrifugated for 20 min. The clear supernatant was decanted and concentrated under reduced pressure to afford KF (257 mg) in 61% yield (96% purity determined by quantitative ^{19}F NMR spectroscopy).

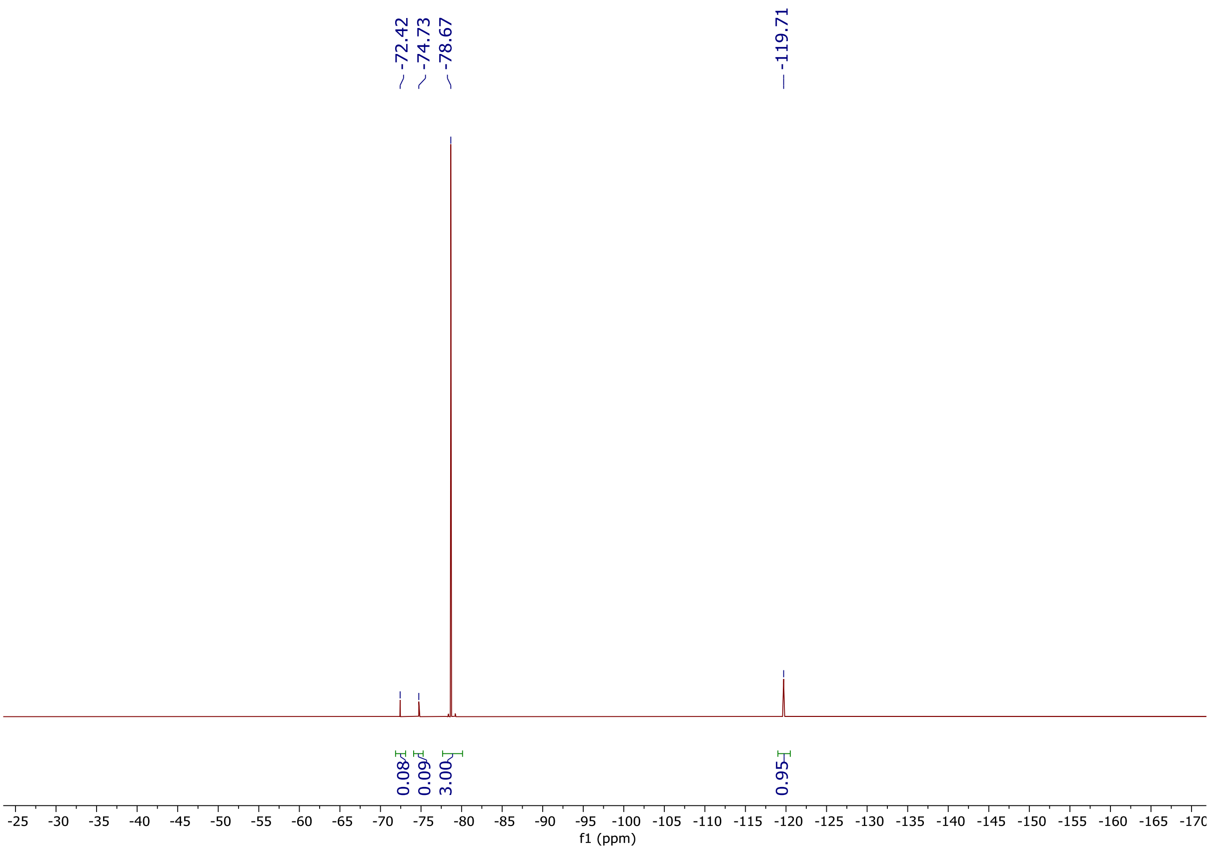

Figure S30. Quantitative ^{19}F NMR spectrum (377 MHz) of 54.2 mg PFOA-mix and 20.2 mg NaOTf (as an internal standard) in D_2O (10 atom% D).

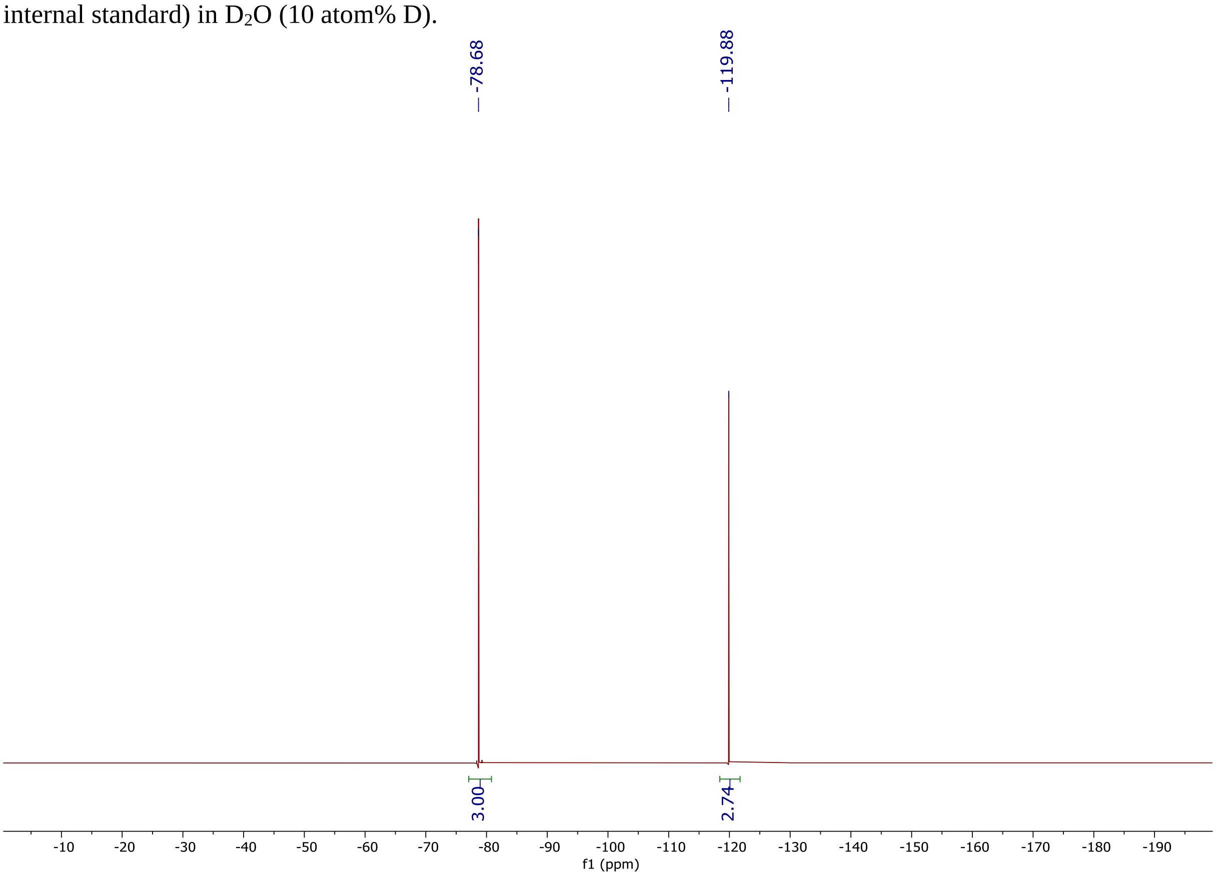

Figure S31. Quantitative ^{19}F NMR spectrum (377 MHz) of 12.3 mg KF^{PFOA} and 12.7 mg NaOTf (as an internal standard) in D_2O (10 atom% D).

8:2 FTOH adsorbed Filtrasorb® 400 Granular Activated Carbon

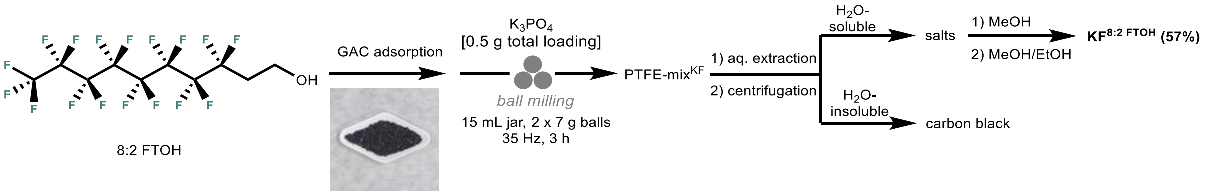

8:2 FTOH (200 mg) and Filtrasorb® 400 Granular Activated Carbon (GAC) from Calgon Carbon (1000 mg) were added to a round-bottom flask and stirred in water (100 mL) at room temperature for 16 hours. The solid mixture was collected by vaccum filtration and dried under reduced pressure. To twelve 15 mL stainless-steel milling jars was added two chrome steel balls (2 × 7 g), 8:2 FTOH adsorbed GAC (100 mg) and K_3PO_4 (400 mg) each. The total material of each jar (8:2 FTOH, GAC and K_3PO_4) was kept constant at 500 mg. The jars were closed and securely fitted to a mill which was set for 3 h at a frequency of 35 Hz. Upon completion, the jars were opened and a sample mixture was prepared for quantitative ^{19}F NMR analysis: 94% KF, 3% K_2PO_3F, total yield: quantitative.

the powder was collected and extracted with H_2O (60 mL). The resulting suspension was centrifuged for 30 min to eliminate water-insoluble carbon black. The resulting clear supernatant was decanted and concentrated under reduced pressure to obtain a solid residue, which was sonicated with MeOH (40 mL) for 30 min and centrifugated for another 30 min. The clear supernatant was decanted and concentrated under reduced pressure to give crude KF. The centrifugate was dissolved in MeOH (40 mL) and stirred at 40 °C for 16 hours. The resulting suspension was centrifuged for 20 min. The clear supernatant was decanted and concentrated under reduced pressure to give crude KF. The crude KF obtained from both steps were combined for further purification. The crude product was extracted with a mixture MeOH (6 mL) and EtOH (2 mL), sonicated for 10 min, and centrifugated for 20 min leading to the precipitation of insoluble salts. The clear supernatant was decanted and concentrated under reduced pressure. The obtained solid was again extracted with a mixture MeOH (6 mL) and EtOH (2 mL), sonicated for 10 min, and centrifugated for 20 min. The clear supernatant was decanted and concentrated under reduced pressure to afford KF (243 mg) in 57% yield (93% purity determined by quantitative ^{19}F NMR spectroscopy).

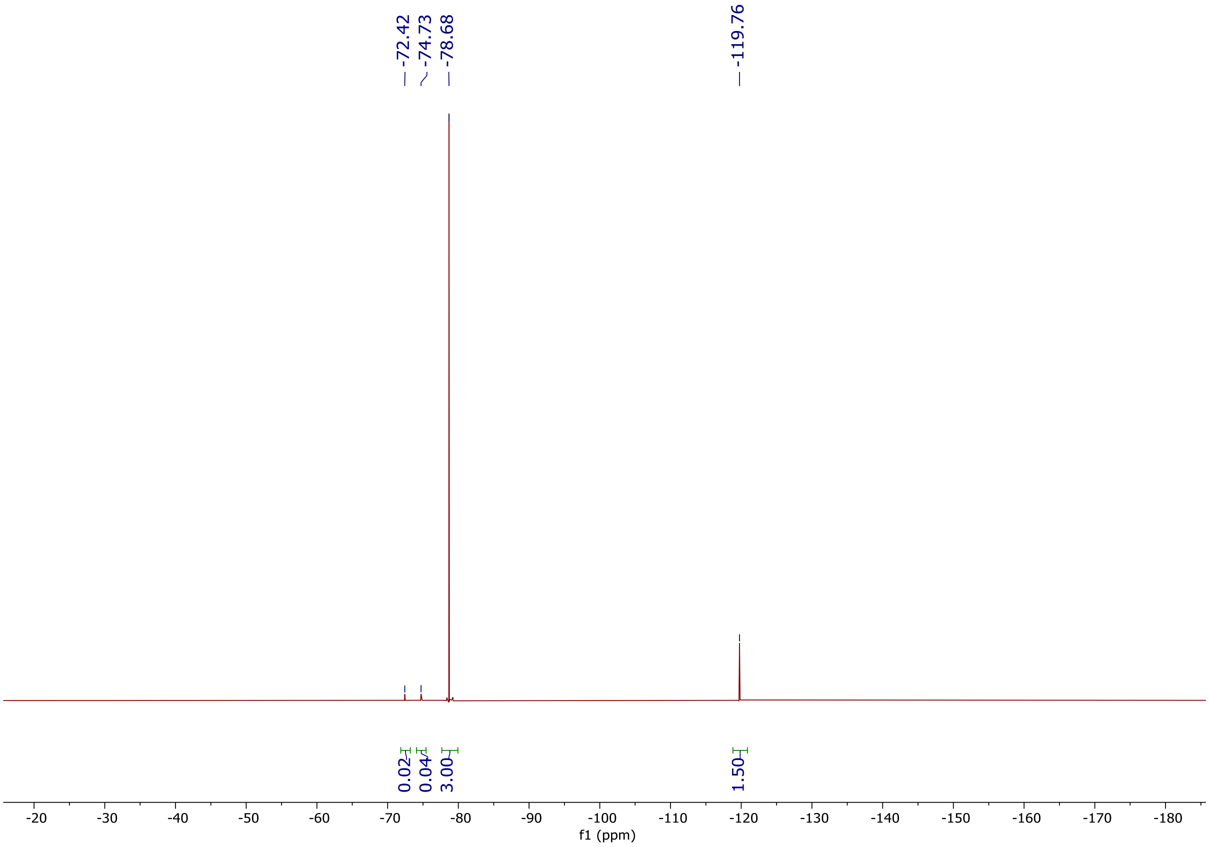

Figure S32. Quantitative ^{19}F NMR spectrum (377 MHz) of 88.1 mg 8:2 FTOH-mix and 11.6 mg NaOTf (as an internal standard) in D_2O (10 atom% D).

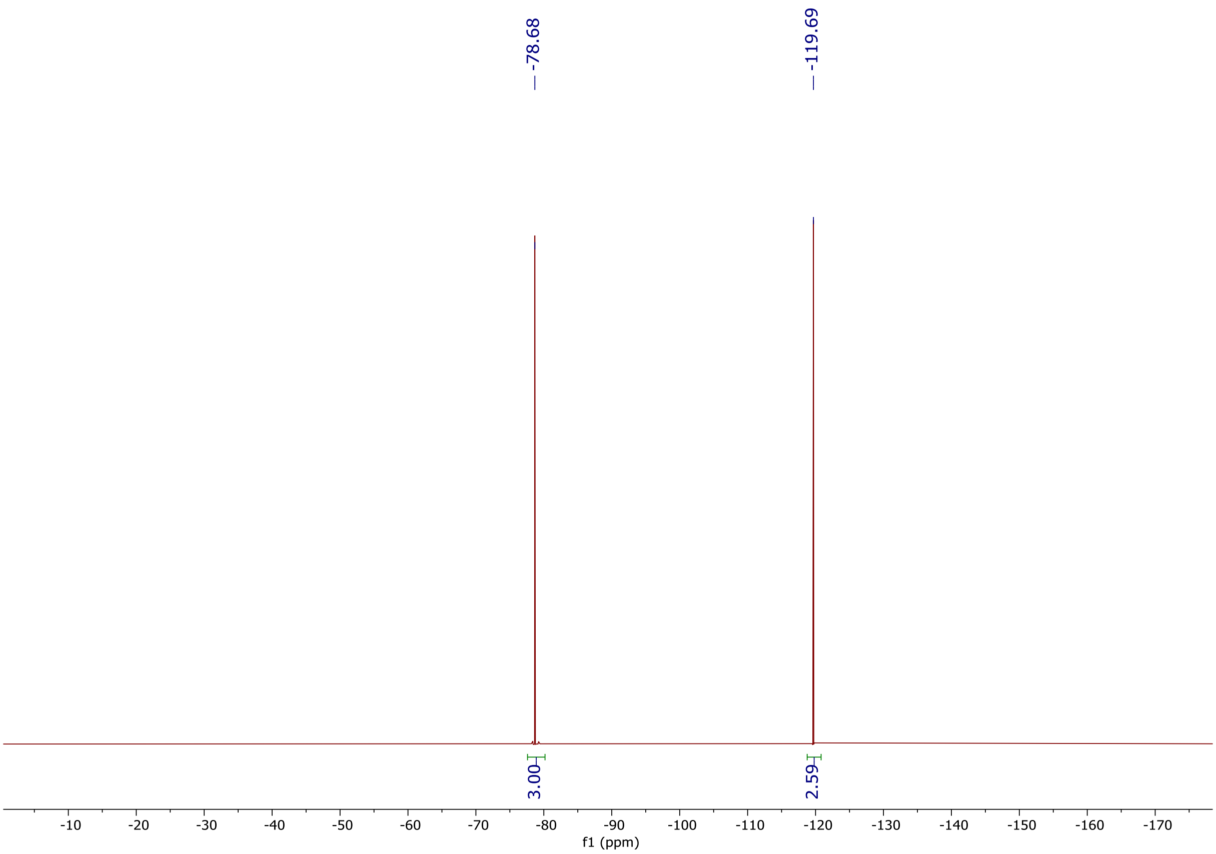

Figure S33. Quantitative ^{19}F NMR spectrum (377 MHz) of 12.4 mg $KF^{8:2FTOH}$ and 13.2 mg NaOTf (as an internal standard) in D_2O (10 atom% D).

6.1.1 Qualitative analysis of KF^{PTFE} by NMR spectroscopy

200 mg KF prepared according to procedure 6.1 were dissolved in D_2O (10 atom% D) (0.4 mL) and analysed by ^{19}F NMR, ^{31}P NMR, ^{13}C NMR, ^{1}H NMR spectroscopy.

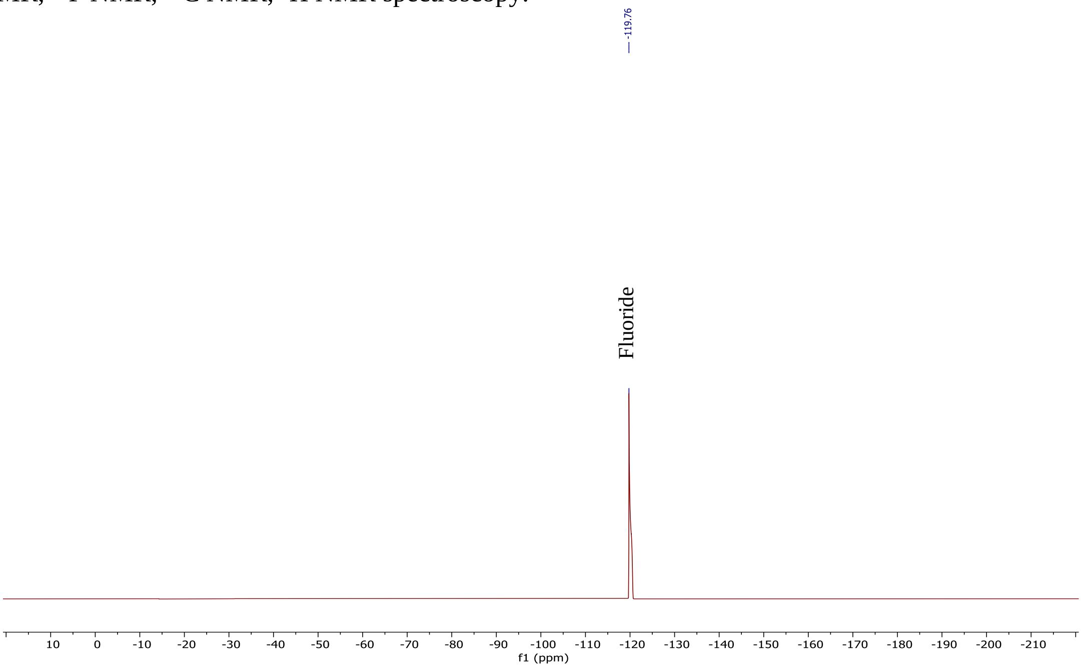

Figure S34. ^{19}F NMR spectrum (471 MHz) of 200 mg isolated KF^{PTFE} in D_2O (10 atom% D) indicating only fluoride as the sole water-soluble fluorine containing component.

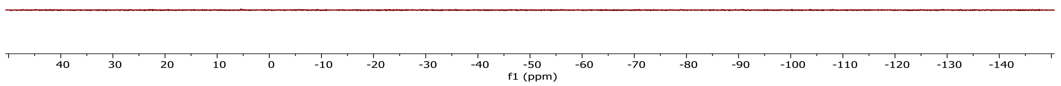

Figure S35. ^{31}P NMR spectrum (162 MHz) of 200 mg isolated KF^{PTFE} in D_2O (10 atom% D) indicating no water-soluble phosphorus containing components.

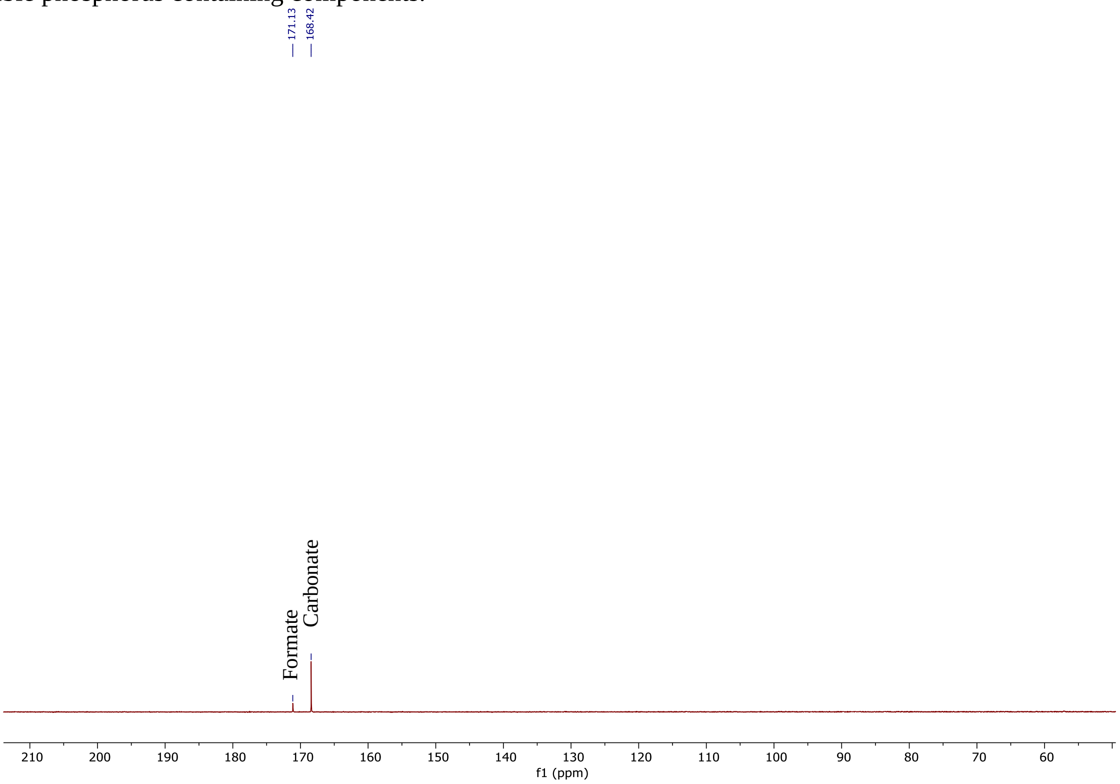

Figure S36. $^{13}C\{^1H\}$ NMR spectrum (126 MHz) of 200 mg isolated KF^{PTFE} in D_2O (10 atom% D) indicating formate and carbonate as impurities.

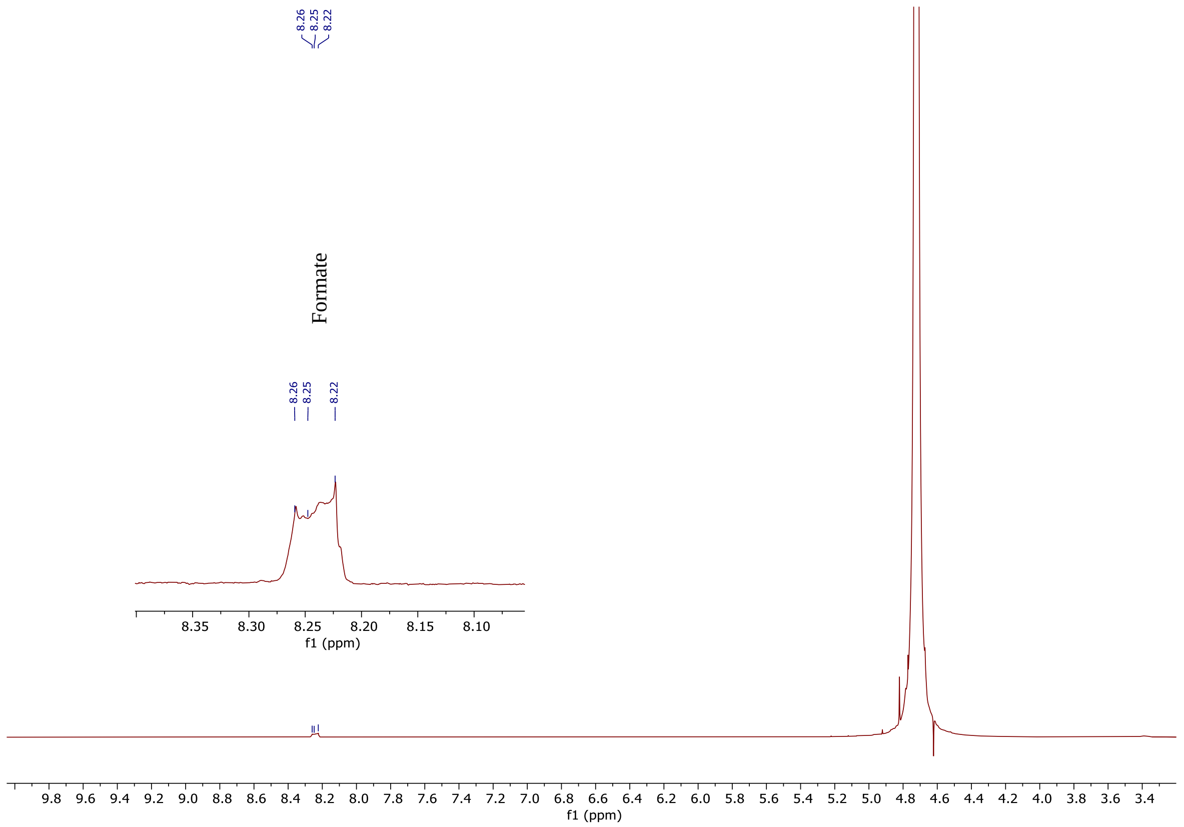

Figure S37. ^{1}H NMR spectrum (400 MHz) of 200 mg isolated KF^{PTFE} in D_2O (10 atom% D) indicating formate as an impurity.

6.1.2 Elemental analysis of KF^{PTFE}

Elemental analyses (F, K) of commercial KF samples, and KF^{PTFE} was carried out by Mikroanalytisches Laboratorium Kolbe.

Table S16. Elemental analyses of KF samples performed in duplicates.

Element	KF (theoretical)	KF Thermo Scientific 99% (metals basis)	KF Sigma Aldrich >99.9% (metals basis)	KF^{PTFE}
F (wt%)	32.70	31.34	32.15	31.10
K (wt%)	67.30	64.09	65.63	63.80

6.1.3 Powder X-ray diffraction pattern of KF^{PTFE}

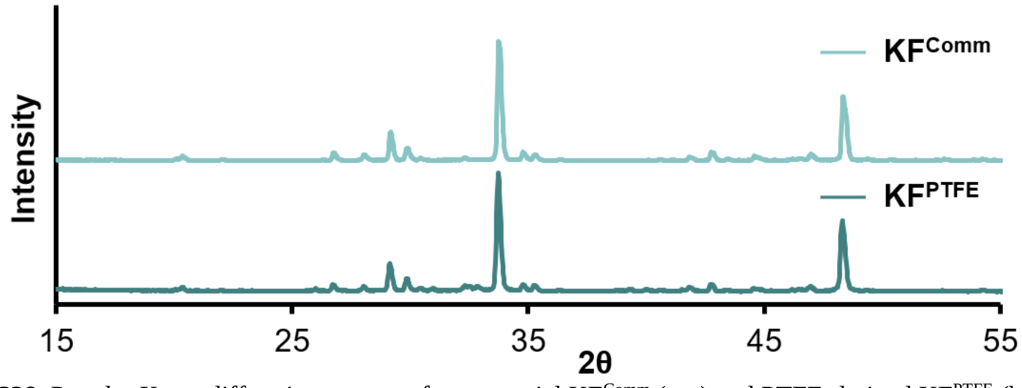

Figure S38. Powder X-ray diffraction pattern of commercial KF^{Comm} (top) and PTFE-derived KF^{PTFE} (bottom).

6.1.4 Performance of KF^{PTFE} in S$_N$Ar fluorination of 2,4-dinitrochlorobenzene

The performance of PTFE-derived KF (KF^{PTFE}) was assessed in the fluorination of 2,4-dinitrochlorobenzene. To this end, an oven dried Schlenk tube under N_2 atmosphere was added KF^{PTFE} (1.5 equiv.), 2,4-dinitrochlorobenzene (0.5 mmol, 1 equiv.) and anhydrous DMSO (0.2 M). After stirring at 100 °C in an oil bath for 1 h, the resulting suspension was cooled to room temperature, and the fluorination yield was determined by quantitative ^{19}F NMR spectroscopy in $CDCl_3$ using 4-fluoroanisole as internal standard.

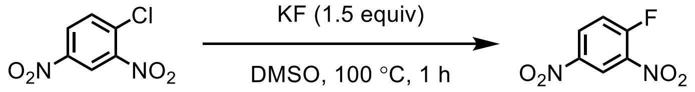

2,4-dinitrochlorobenzene 2,4-dinitrofluorobenzene

Entry	KF	ArF (%)[a]
1	KF^{Comm}	95%
2	KF^{PTFE}	95%

[a] The fluorination yield was determined by quantitative ^{19}F NMR spectroscopy in $CDCl_3$ using 4-fluoroanisole as internal standard.

6.2 Isolation of KF from PTFE-mix^{PF}

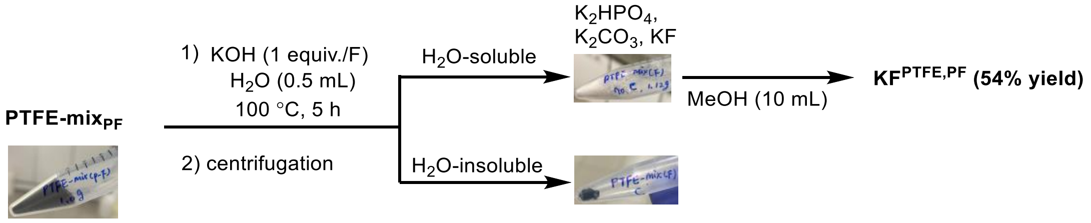

A 7 mL vial was charged with PTFE-mix^{PF} (1.00 g, corresponding to 108 mg PTFE), KOH (4 equiv.), and H_2O (0.5 mL). The vial was closed and heated to 100 °C for 5 h until no K_2PO_3F was observed by ^{19}F NMR spectroscopy. Subsequently, all the reaction mixture was transferred into a centrifugation vial with H_2O (10 mL) and centrifugated for 30 min to eliminate water-insoluble carbon black. The resulting clear supernatant was decanted and concentrated under reduced pressure to obtain a colorless solid, which was extracted with MeOH (10 mL) and filtered. The filtrate was concentrated under reduced pressure to afford KF (136 mg) in 54% yield (92% purity determined by quantitative ^{19}F NMR spectroscopy).

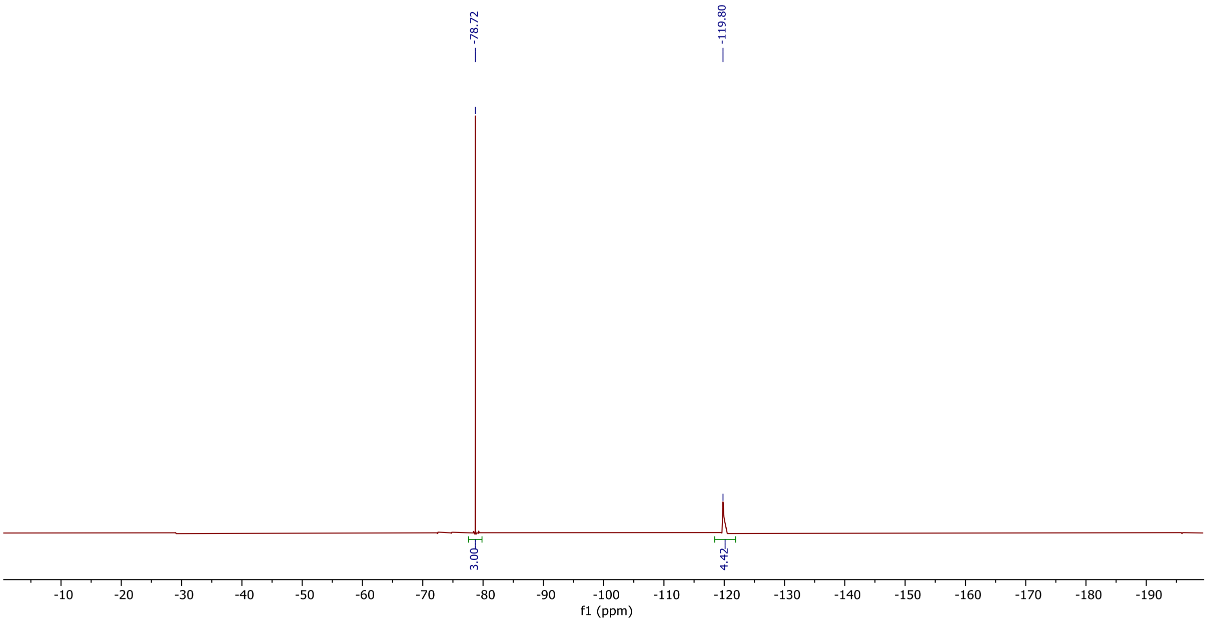

Figure S39. Quantitative ^{19}F NMR spectrum (377 MHz) of 18.0 mg KF^{PTFE}_{PF} and 10.0 mg NaOTf (as an internal standard) in D_2O (10 atom% D).

6.3 Synthesis of 4-$OHCC_6H_4BF_3K$ from KF^{PTFE}

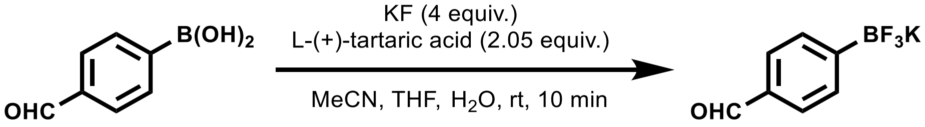

To a suspension of the 4-$OHCC_6H_4B(OH)_2$ (0.5 mmol, 61 mg) in acetonitrile (2 mL) was added a solution of KF^{PTFE} (4 equiv., 2 mmol, 116 mg) in H_2O (0.2 mL) at room temperature. The mixture was stirred until complete dissolution of the organoboronic acid. Subsequently, a solution of L-(+)-tartaric acid (2.05 equiv., 1.03 mmol, 154 mg) in THF (0.7 mL) was added dropwise over a period of one minute. The resulting suspension was stirred for another 10 min. The reaction mixture was then diluted with acetonitrile (1 mL) and filtered through a sintered glass frit and washed with acetonitrile (3 x 5 mL). The combined filtrates were concentrated under reduced pressure and the obtained solid was washed with Et_2O (10 mL) to afford pure title compound (99 mg, 93%).

^{1}H NMR (400 MHz, DMSO-d_6) δ 9.89 (s, 1H), 7.64 (d, J = 7.5 Hz, 2H), 7.53 (d, J = 7.4 Hz, 2H).
^{13}C NMR (101 MHz, DMSO-d_6) δ 193.4, 134.0, 131.8, 127.9. C-BF_3 signal not observed due to quadrupolar relaxation.
^{19}F NMR (377 MHz, DMSO-d_6) δ -139.9 (s).
NMR data are in accordance with the literature [60].

6.4 Synthesis of TMAF and TMAF(*t*AmylOH) from PTFE-mix^{PF}

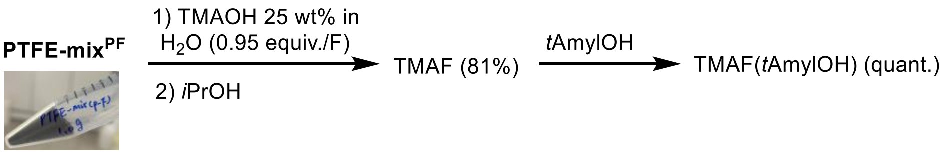

To a 7 mL vial was added PTFE-mix^{PF} (1.00 g) and TMAOH (25wt% in H_2O, 0.95 equiv./F). The vial was closed and heated at reflux for 10 h until complete consumption of K_2PO_3F (reaction monitoring by ^{19}F NMR spectroscopy). The reaction mixture was cooled to room temperature, extracted with *i*PrOH (3 x 8 mL) and the combined organic layers were concentrated under reduced pressure. The obtained colorless solid was extracted with a mixture of anhydrous *i*PrOH (3 mL) and *n*-pentane (10 mL) and filtered through a sintered glass frit. The filtrate was concentrated under reduced pressure to obtain pure TMAF as a colorless solid (326 mg, 81%).

A 25 mL round bottom flask, equipped with a magnetic stirring bar, was charged with the obtained TMAF solid and anhydrous *t*AmylOH (5 mL). The resulting mixture was stirred at room temperature for 5 h, Then, the majority of the solvent was removed under reduced pressure using a rotary evaporator. The resulting wet solid was then further dried under high vacuum (< 0.1 mbar) overnight. The solids were periodically broken up using a spatula to ensure that no large clumps persist. After this process, TMAF(*t*AmylOH) (634 mg, quant.) was obtained as a colorless free-flowing solid.

To monitor other potential tetramethylammonium salts present in the isolated TMAF, ^{1}H and ^{19}F NMR spectroscopic analyses of TMAF in MeOD-d_4 using 1,3,5-trifluorobenzene as an internal standard were performed.

$$F^- : NMe_4^+ = \frac{molar\ ratio\ in\ ^{19}F\ NMR}{molar\ ratio\ in\ ^{1}H\ NMR} = \frac{3.60}{43.26/12} = 1.00 : 1$$

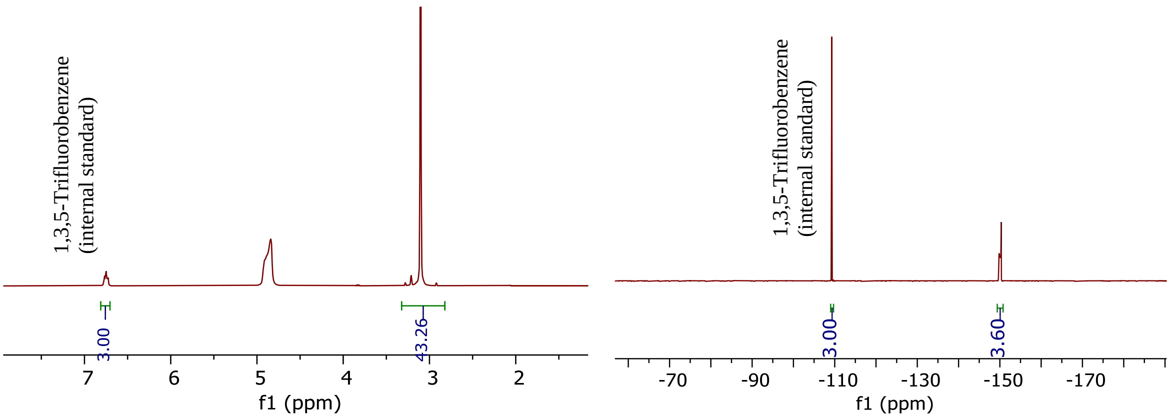

Figure S40. Quantitative ^{1}H NMR spectrum (400 MHz, left) and quantitative ^{19}F NMR spectrum (377 MHz, right) of TMAF^{PTFE} in MeOD-d_4 using 1,3,5-trifluorobenzene as an internal standard.

TMAF

^{1}H NMR (400 MHz, D_2O) δ 3.17 (s, 12H).
^{13}C NMR (101 MHz, D_2O) δ 55.2 (t, $^{1}J_{CN}$ = 4 Hz).
^{19}F NMR (377 MHz, D_2O) δ -122.1 (s).
The spectroscopic data corresponds to that of an authentic commercial sample.

TMAF(*t*AmylOH)

^{1}H NMR (400 MHz, D_2O) δ 3.18 (s, 12H), 1.50 (q, J = 7.6 Hz, 2H), 1.18 (s, 6H), 0.87 (t, J = 7.6 Hz, 3H).
^{13}C NMR (101 MHz, D_2O) δ 72.0, 55.2 (t, $^{1}J_{CN}$ = 4 Hz), 35.2, 27.1, 7.9.
^{19}F NMR (377 MHz, D_2O) δ -122.6 (s).
Spectroscopic data are in accordance with those in literature [46].

6.5 Synthesis of TBAF(*t*BuOH)$_4$ from PTFE-mix^{PF}

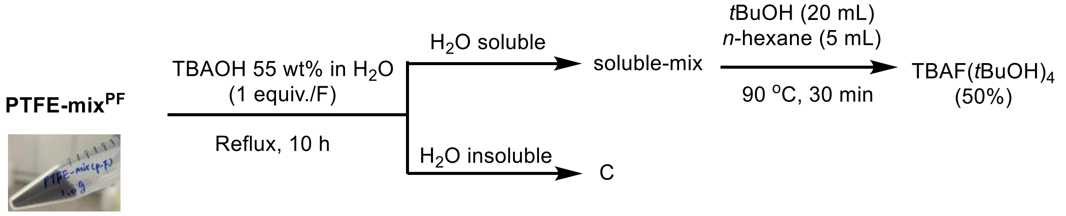

To a 7 mL vial was added PTFE-mix^{PF} (1.00 g) and TBAOH (55wt% in H_2O, 1 equiv./F). The vial was closed and heated at reflux for 10 h until complete consumption of K_2PO_3F (reaction monitoring by ^{19}F NMR spectroscopy). The reaction mixture was diluted with H_2O (10 mL), centrifugated for 30 min to remove H_2O-insoluble carbon black, and the clear supernatant was decanted and concentrated under reduced pressure. The resulting solid was re-dissolved in a mixture of *t*BuOH (20 mL) and *n*-hexane (5 mL) and stirred for 30 min at 90 °C. Afterwards, the solution was cooled to room temperature and a colorless, crystalline solid precipitated. The crystallised solid was isolated by filtration through a sintered glass frit, rapidly washed with 5 mL of tBuOH/*n*-hexane (7/1) and dried in vacuo for 10 min to afford 1.2 g of TBAF(*t*BuOH)$_4$ as a colorless, crystalline solid (50% yield).

TBAF(*t*BuOH)$_4$

^{1}H NMR (400 MHz, $CDCl_3$) δ 3.40 – 3.26 (m, 8H), 1.69 – 1.60 (m, 8H), 1.43 (h, J = 7.4 Hz, 8H), 1.24 (s, 36H), 0.99 (t, J = 7.3 Hz, 12H).
^{13}C NMR (101 MHz, D_2O) δ 69.1, 58.0, 30.1, 23.2, 19.2, 13.1.
^{19}F NMR (377 MHz, D_2O) δ -121.3 (s).
Spectroscopic data are in accordance with those in literature [47].

To monitor other potential tetrabutylammonium salts of the isolated TBAF(tBuOH)$_4$, ^{1}H and ^{19}F NMR spectroscopic analyses of TBAF(tBuOH)$_4$ in MeOD-d_4 using 1,3,5-trifluorobenzene as an internal standard were performed.

$$F^- : NBu_4^+ = \frac{molar\ ratio\ in\ ^{19}F\ NMR}{molar\ ratio\ in\ ^1H\ NMR} = \frac{0.57}{4.66/8} = 0.98 : 1$$

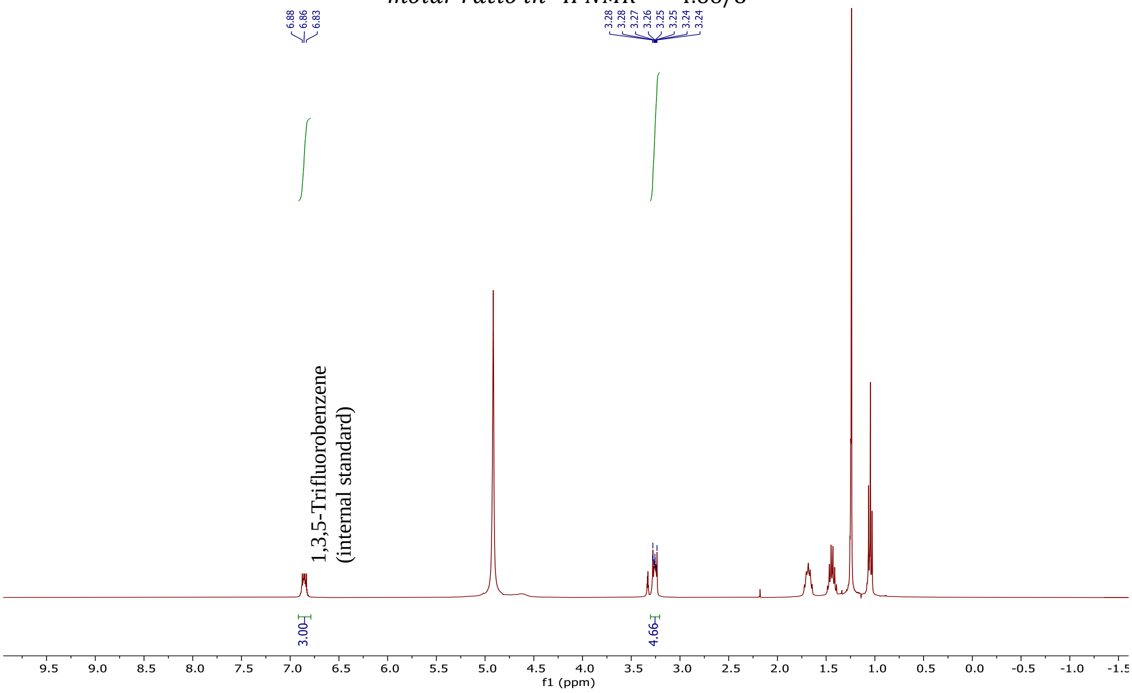

Figure S41. Quantitative ^{1}H NMR spectrum (400 MHz) of TBAF(tBuOH)$_4$ in MeOD-d_4 using 1,3,5-trifluorobenzene as an internal standard.

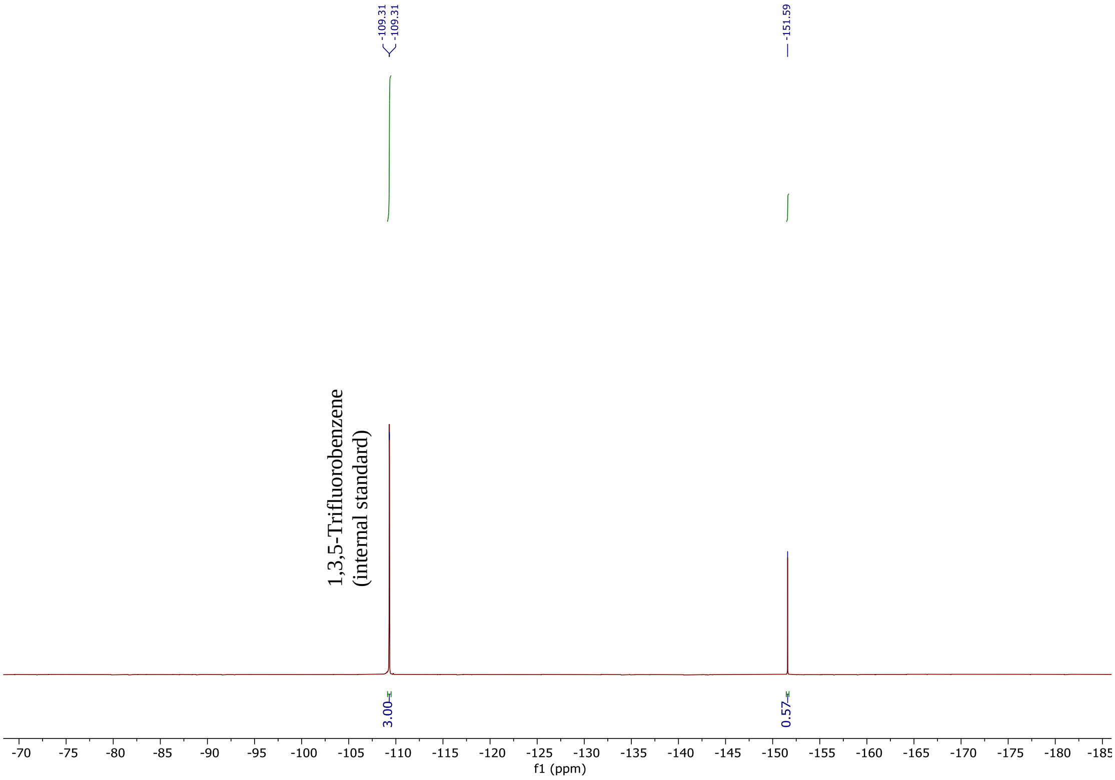

Figure S42. Quantitative ^{19}F NMR spectrum (377 MHz) of TBAF(tBuOH)$_4$ in MeOD-d_4 using 1,3,5-trifluorobenzene as an internal standard.

7. Phosphate recovery and recycling

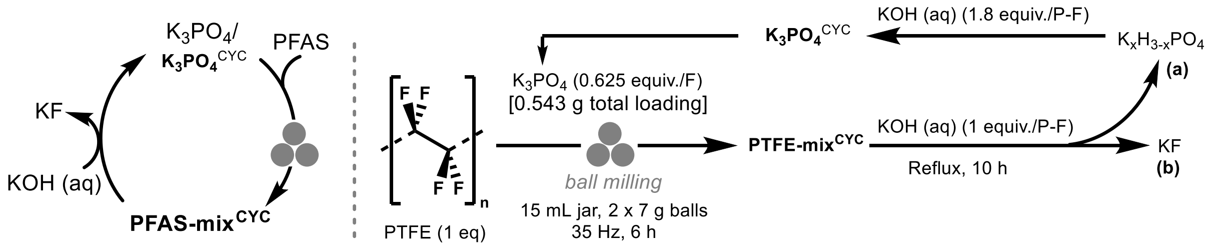

Run	PTFE-mix^{CYC} [F_{tot} (F^-:PO_3F^{2-})]	KF isolation yield	Phosphate recovery (K_3PO_4/$K_4P_2O_7$)
1st	Quant. (1.6:1)	76%	90%/3%
2nd	91% (1.8:1)	65%	85%/5%
3rd	89% (2.2:1)	67%	74%/11%

General Procedure: To *n* 15 mL stainless-steel milling jars, two chrome steel balls (2 × 7 g), PTFE (1 equiv., 86 mg) and K_3PO_4 (0.625 equiv./F, 457 mg) or $K_3PO_4^{CYC}$ (457 mg) were added. The jars were sealed and securely fitted to a mill which was set for 6 h at a frequency of 35 Hz. Upon completion, the jars were opened. An aliquot of PTFE-mix* (~20-30 mg) spiked with sodium triflate (~10 mg, as internal standard) was extracted with D_2O (10 atom% D), centrifugated for 15–30 minutes and the supernatant analysed by quantitative ^{19}F-NMR spectroscopy.

The obtained *n* jars of PTFE-mix were extracted with H_2O (~20 mL per jar) and combined. The resulting suspension was centrifugated for 10 min to sediment water-insoluble carbon black. The resulting clear supernatant was decanted and concentrated to 5 mL under reduced pressure. KOH (1 equiv./P–F*, as determined by quantitative ^{19}F-NMR spectroscopy) was added and the solution heated at reflux for 10 h until no PO_3F^{2-} was detected by quantitative ^{19}F-NMR spectroscopy. MeOH (10–20 mL per jar) was added and the reaction placed in an ultrasonic bath for 1.5 h. The supernatant was decanted and collected, while the remaining solid was further extracted with H_2O (~0.3 mL per jar) and MeOH (10–20 mL per jar) and further subjected to 1.5 h of ultrasonic activation. All extracts were combined and centrifugated for 10 min, leading to the sedimentation of insoluble salts $K_xH_{3-x}PO_4$ **(a)**. The clear supernatant was then decanted and concentrated under reduced pressure to afford crude KF. Crude KF was further purified by suspension in MeOH:EtOH (6:1, ~3 mL per jar) and placed in an ultrasonic bath for 30 min, followed by centrifugation for 10 min leading to the precipitation of insoluble salts. The clear supernatant was decanted and the solvent removed *in vacuo* to afford KF **(b)**.

To the insoluble salts **(a)**, H_2O (2.5 mL per jar) and KOH (1.8 equiv./P–F*, as determined by quantitative ^{19}F-NMR spectroscopy) were added to adjust the pH to 14 before the mixture was dried under reduced pressure. The resulting residue was further dried *in vacuo* with further heating for 12 h to afford a mixture of recovered phosphate salts, $K_3PO_4^{CYC}$. $K_3PO_4^{CYC}$ was analysed by quantitative ^{31}P-NMR, quantitative ^{19}F-NMR and ^{13}C-NMR spectroscopy in D_2O (10 atom% D) using triethyl phosphate, sodium triflate and potassium acetate as internal standards, respectively.

Table S17. The procedure was run for three cycles, key data summarized in the table below:

Run	*n*	PTFE-mix^{CYC} [F_{tot} (F^{-}:PO_3F^{2-})]*	Isolated KF		$K_3PO_4^{CYC}$						
			Yield / mg (%)	Purity / %	Yield / g	$[P]^{Rcov.}$ (K_3PO_4/$K_4P_2O_7$) / %	K_3PO_4 / %	$K_4P_2O_7$ / %	KF / %	K_2CO_3 / %	$K_2C_2O_4$ / %
1	6	Quant. (1.6:1)	947 (76)	96	2.92	90/3	85	4	4	7	0
2	4	91% (1.8:1)	600 (65)	94	1.78	85/5	84	9	6	8	1
3	2	89% (2.2:1)	335 (67)	91	0.87	74/11	74	17	3	8	0

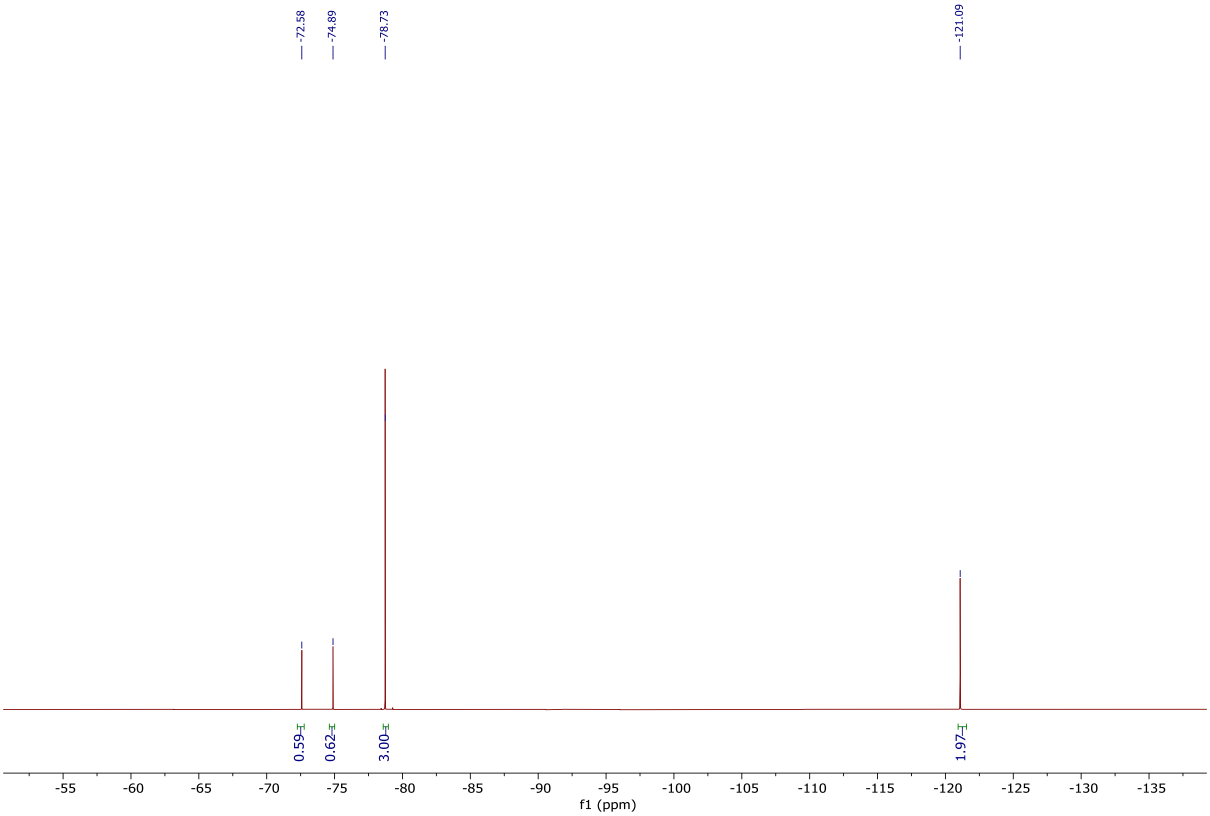

Figure S43. Quantitative ^{19}F NMR spectrum (377 MHz) of an aliquot of first run PTFE-mix^{CYC} in D_2O (10 atom% D) using sodium triflate as an internal standard, indicates fluorine content of fluoride ($\delta_F = -121.1$, 62%), and fluorophosphate ($\delta_P = -73.7$ (d, $^1J_{PF} = 867$ Hz), 38%).

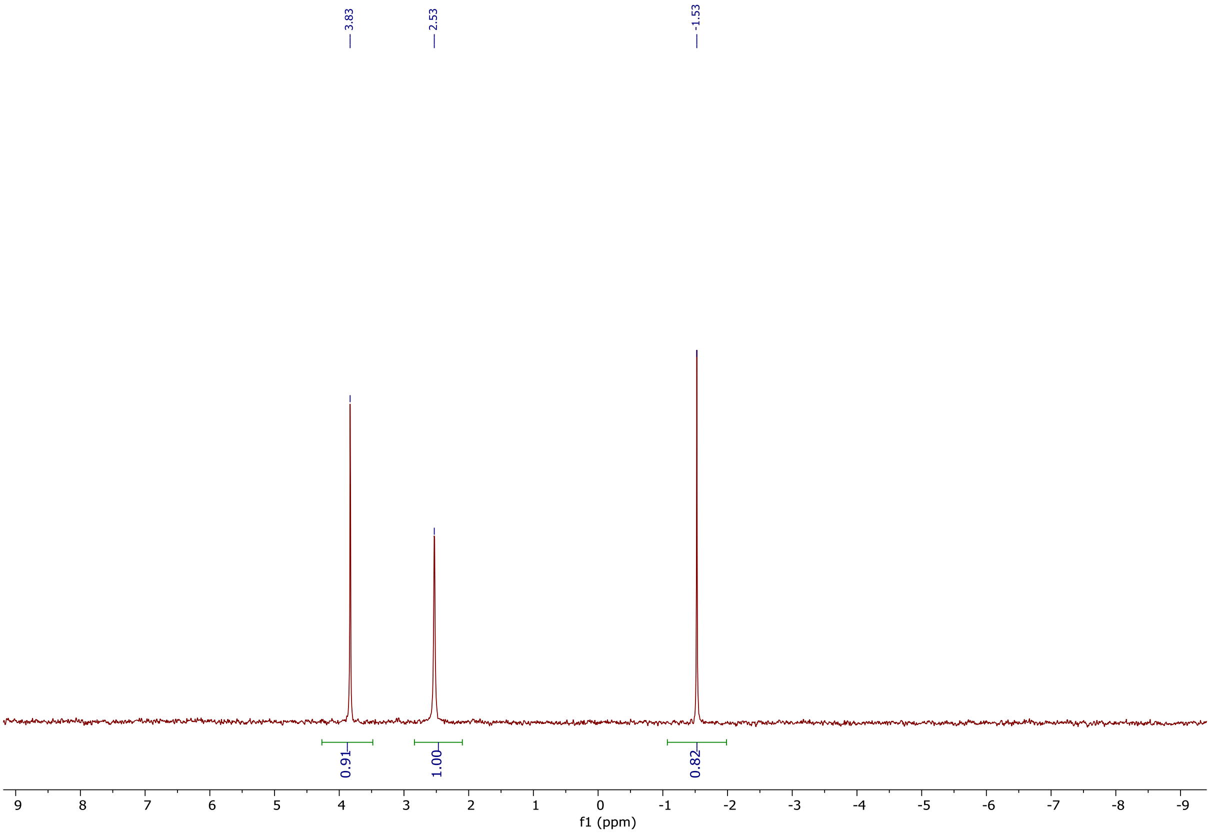

Figure S44. Quantitative ^{31}P NMR spectrum (162 MHz) of an aliquot of first run PTFE-mix^{CYC} in D_2O (10 atom% D) indicates phosphorus content of fluorophosphate (δ_P = 1.2, (d, $^1J_{PF}$ = 866 Hz), 63%), phosphate (δ_P = 2.5, 37%).

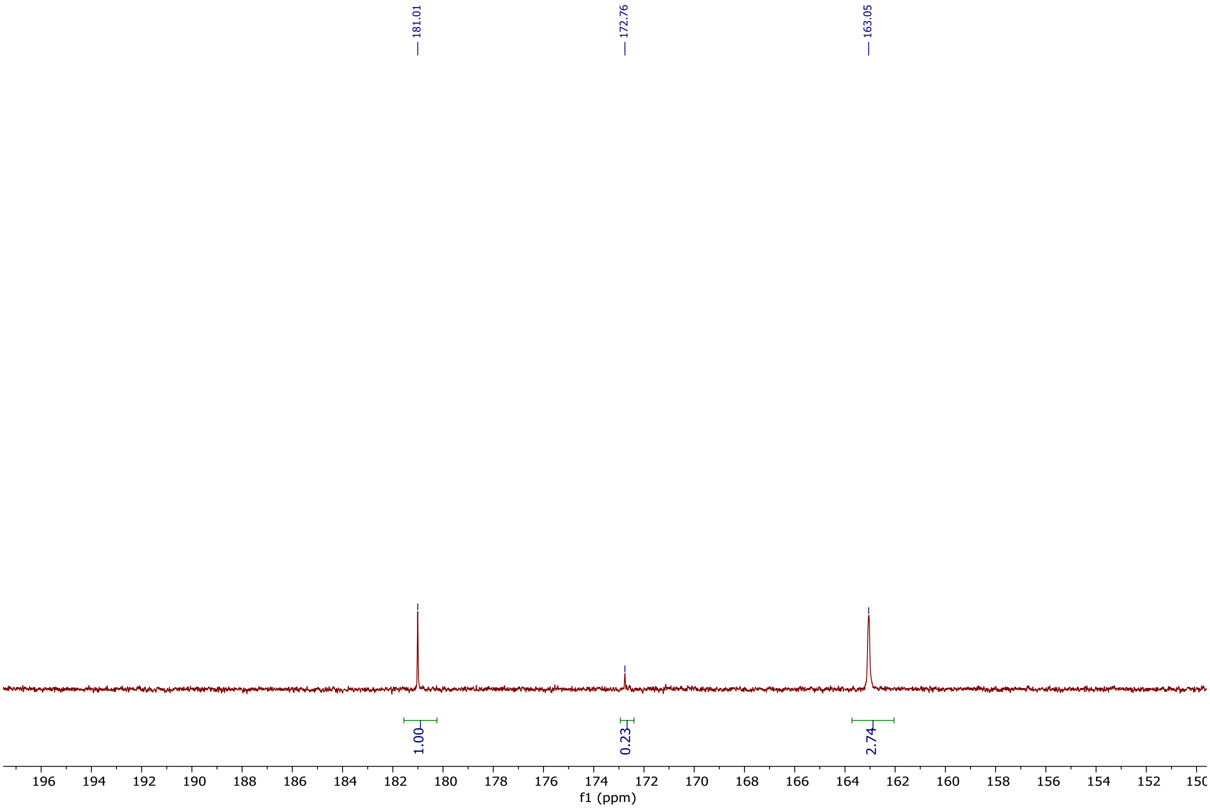

Figure S45. Quantitative $^{13}C\{^1H\}$ NMR spectrum (126 MHz, 1477 scans at 40 s recycle delay) of the whole jar PTFE-mix^{CYC} in D_2O (10 atom% D) with KOAc (200 µL, 0.509 M solution in D_2O) as an internal standard indicates carbon content of carbonate (δ_C = 163.0, 16%), and oxalate (δ_C = 172.8, 1%).

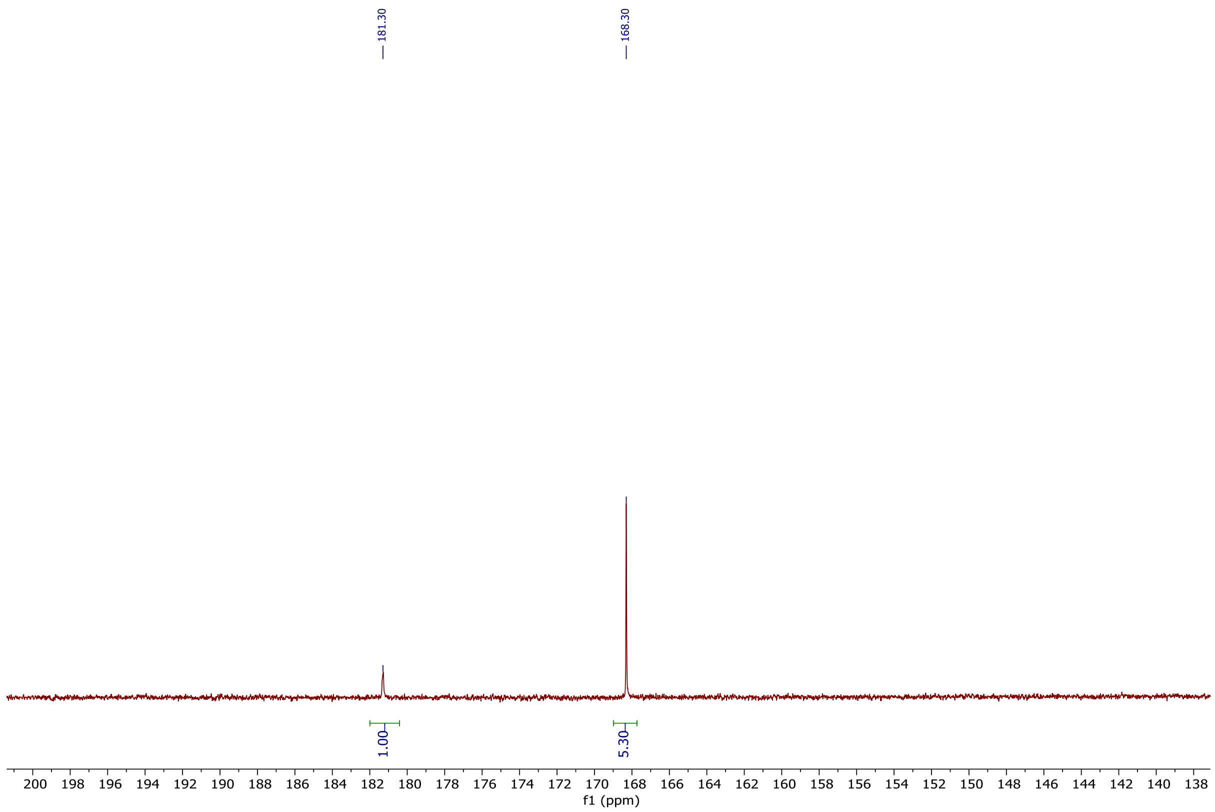

Figure S46. PTFE-mix^{CYC} released CO_2 was captured with aqueous KOH (100 mg/mL, 1mL) and analyzed by quantitative $^{13}C\{^1H\}$ NMR spectrum (126 MHz, 800 scans at 40 s recycle delay) in D_2O (10 atom% D) with KOAc (200 µL of a 0.509 M solution in D_2O) as an internal standard indicates carbon content of carbonate (δ_C = 168.3, 31%).

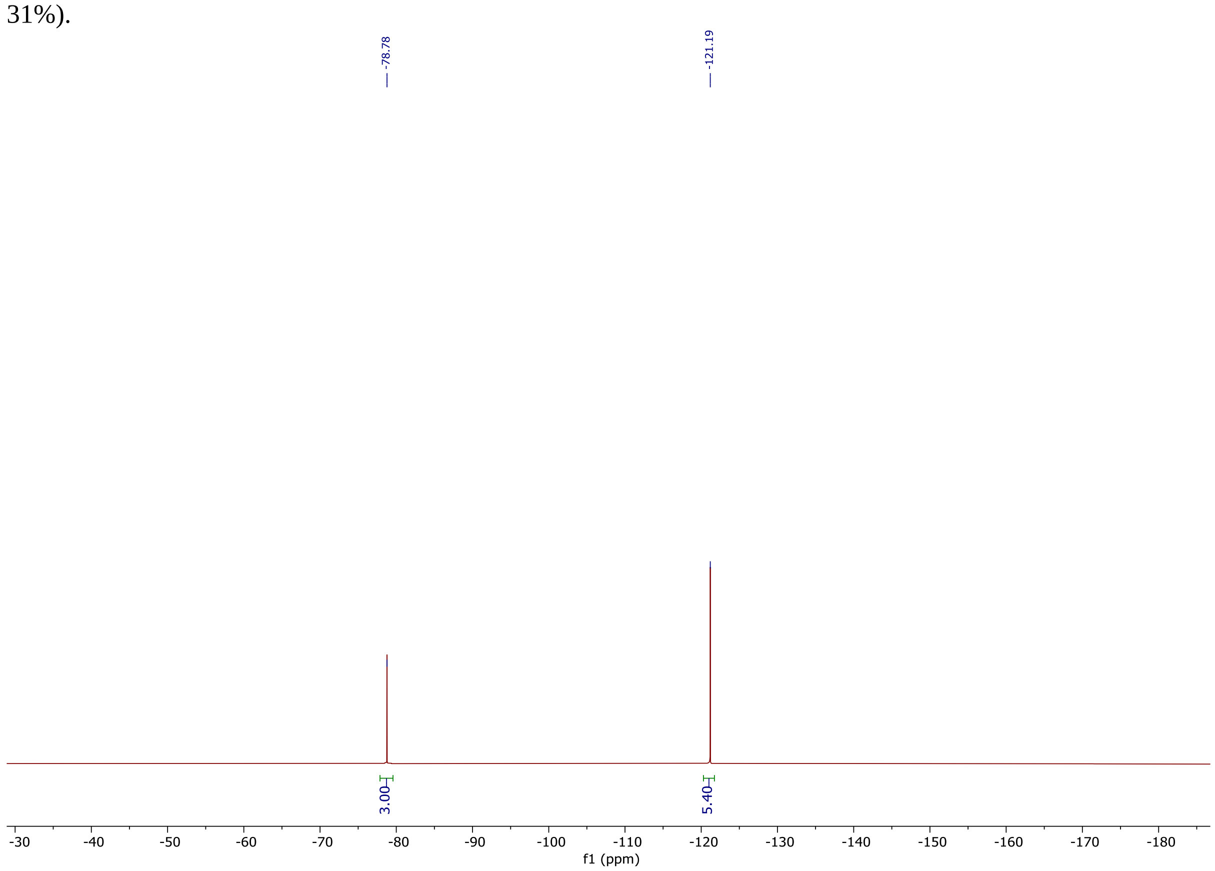

Figure S47. Quantitative ^{19}F NMR spectrum (377 MHz) of KF (18.0 mg, isolated from first run) and NaOTf (10.0 mg, as an internal standard) in D_2O (10 atom% D) indicates 96% purity of KF. During the KF isolation, black carbon (10 mg, 48%) was also isolated.

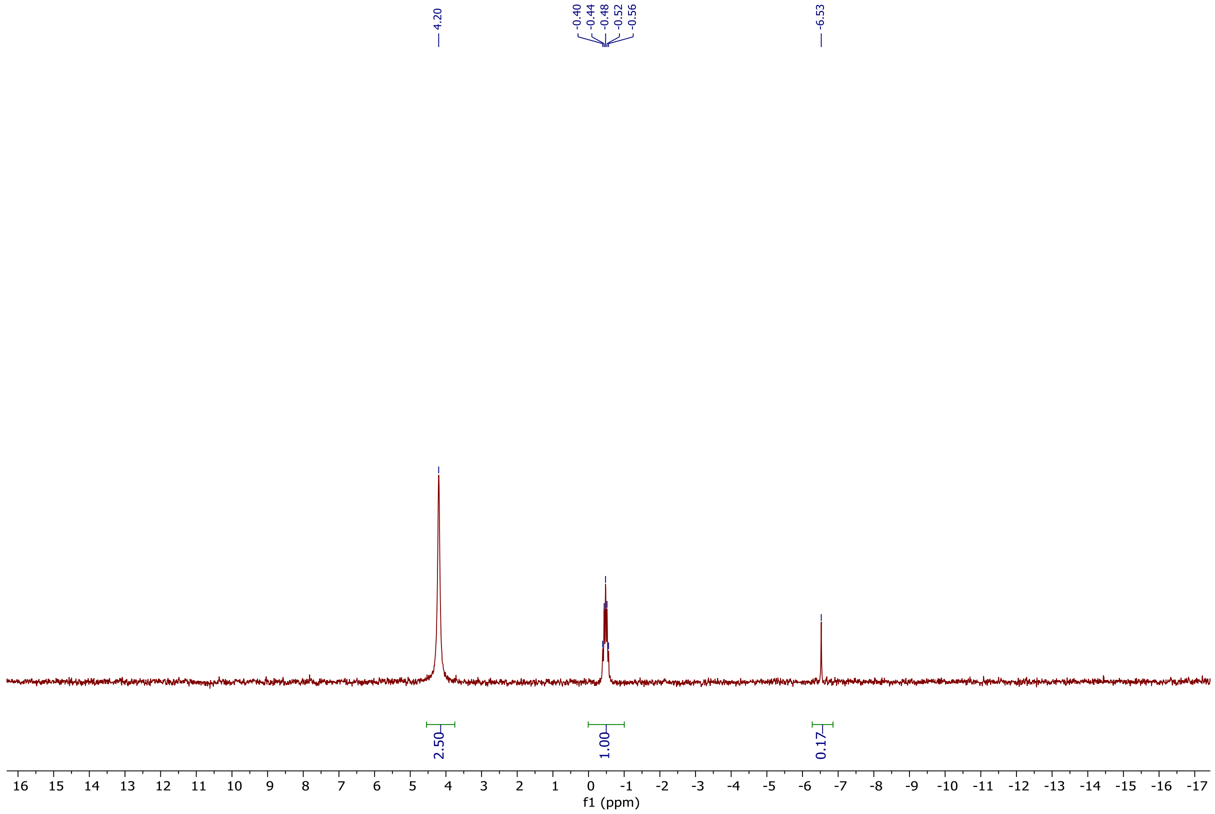

Figure S48. Quantitative ^{31}P NMR spectrum (203 MHz) of an aliquot of first run recovered $K_3PO_4^{CYC}$ (36 mg) and triethyl phosphate (10.5 mg, as an internal standard) in D_2O (10 atom% D) indicates phosphate (δ_P = 4.2, 90%), pyrophosphate (δ_P = -6.5, 6%) recovery, and phosphate (85%) and pyrophosphate (4%) in $K_3PO_4^{CYC}$.

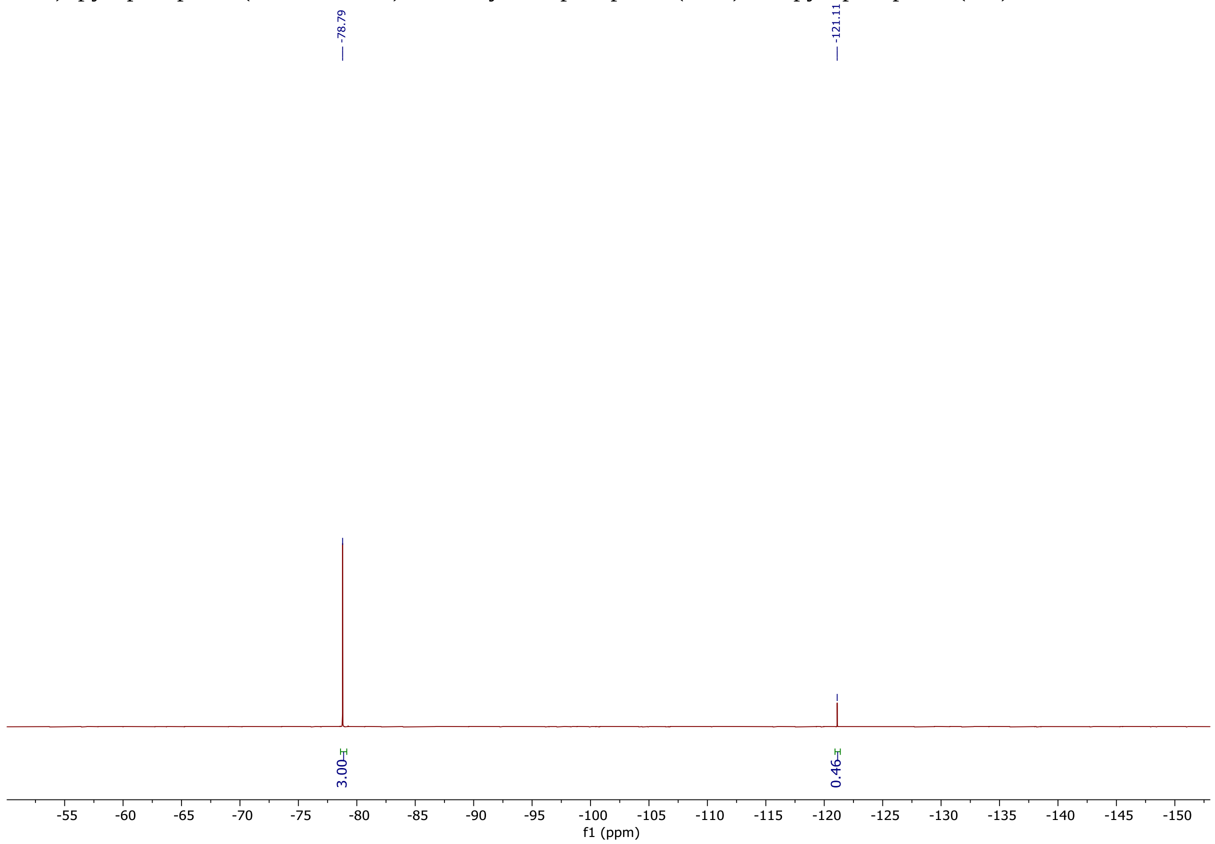

Figure S49. Quantitative ^{19}F NMR spectrum (471 MHz) of first run recovered $K_3PO_4^{CYC}$ (36 mg) and NaOTf (10.0 mg, as an internal standard) in D_2O (10 atom% D) indicates KF (4%) in the first run recovered $K_3PO_4^{CYC}$.

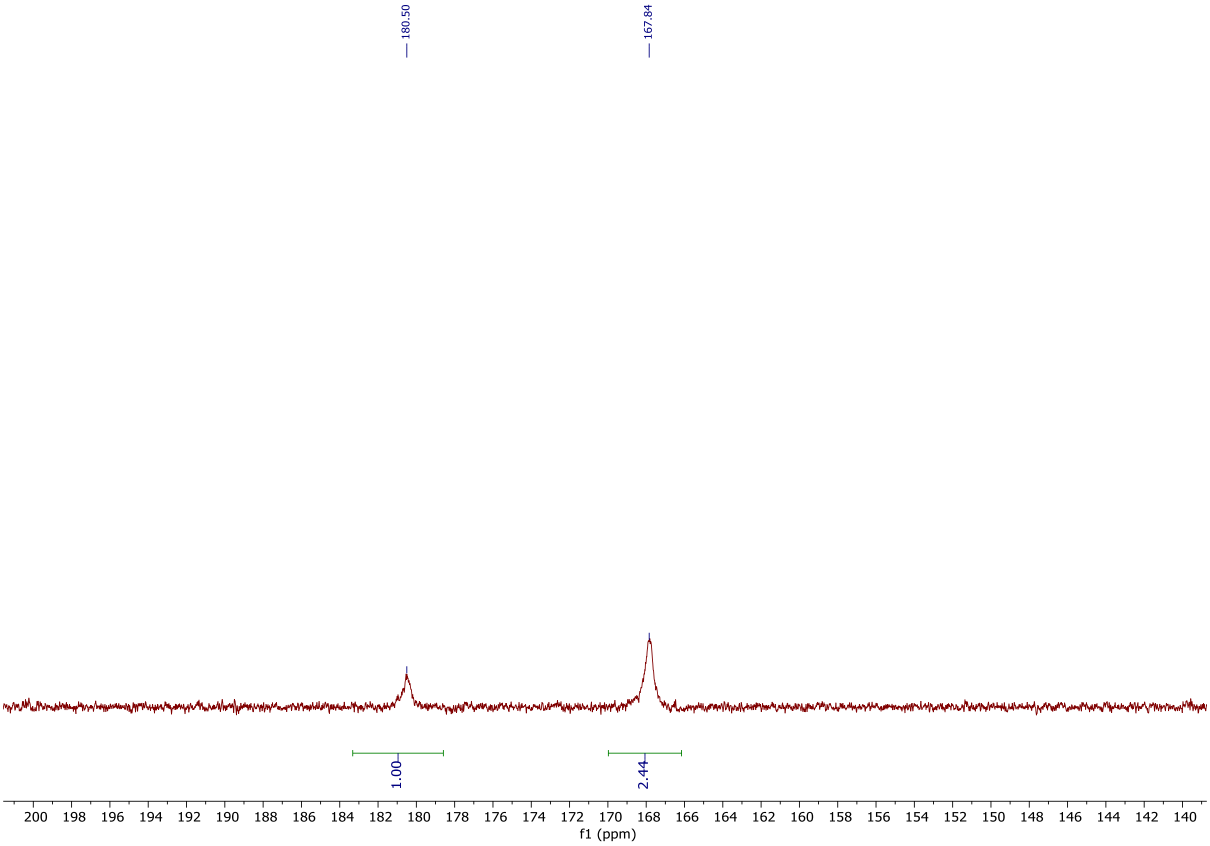

Figure S50. Quantitative $^{13}C\{^{1}H\}$ NMR spectrum (151 MHz, 256 scans at 40 s recycle delay) of first run recovered $K_3PO_4^{CYC}$ (500 mg) in D_2O (10 atom% D) with KOAc (200 µL of a 0.509 M solution in D_2O) as an internal standard indicates carbonate (δ_C = 167.8, 7%) in the first run recovered $K_3PO_4^{CYC}$.

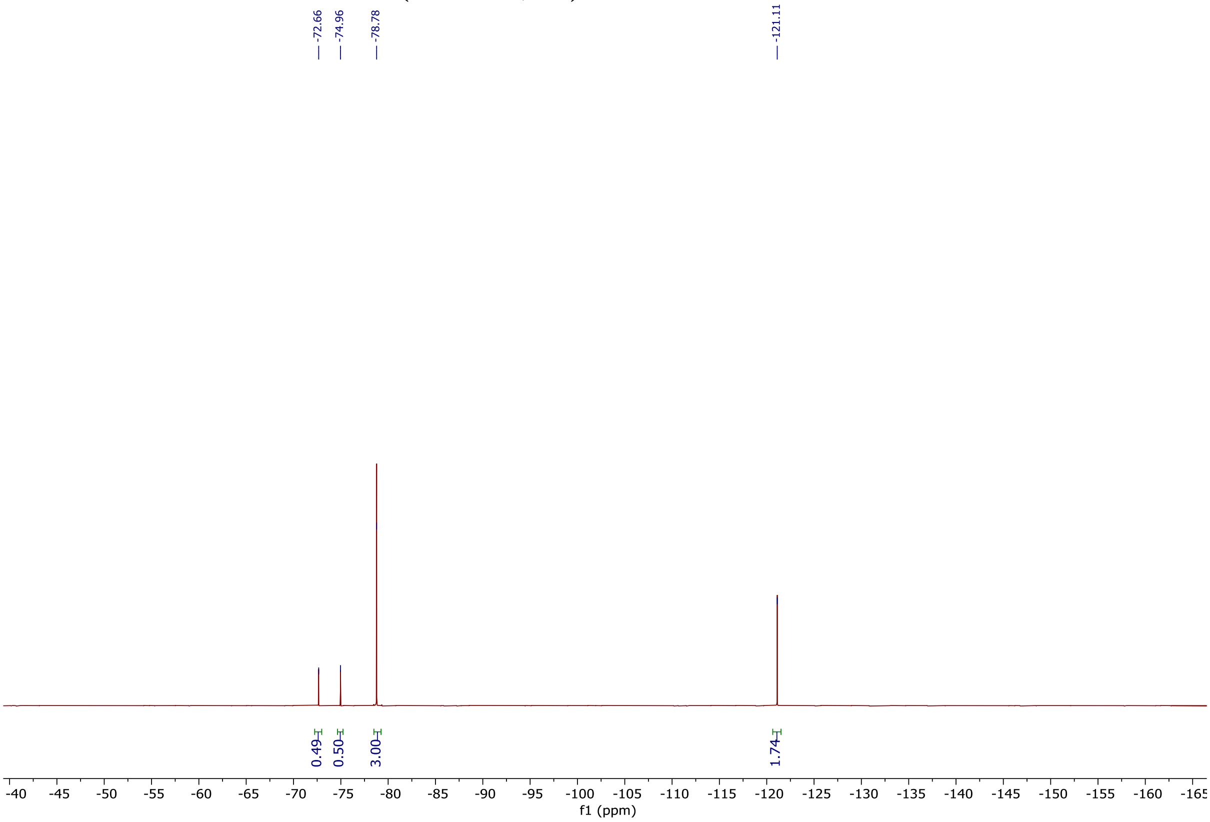

Figure S51. Quantitative ^{19}F NMR spectrum (377 MHz) of an aliquot of second run PTFE-mix^{CYC} (25 mg) in D_2O (10 atom% D) using sodium triflate (10mg) as an internal standard, indicates fluorine content of fluoride (δ_F = −121.1, 58%), and fluorophosphate (δ_P = −73.7 (d, $^{1}J_{PF}$ = 867 Hz), 33%).

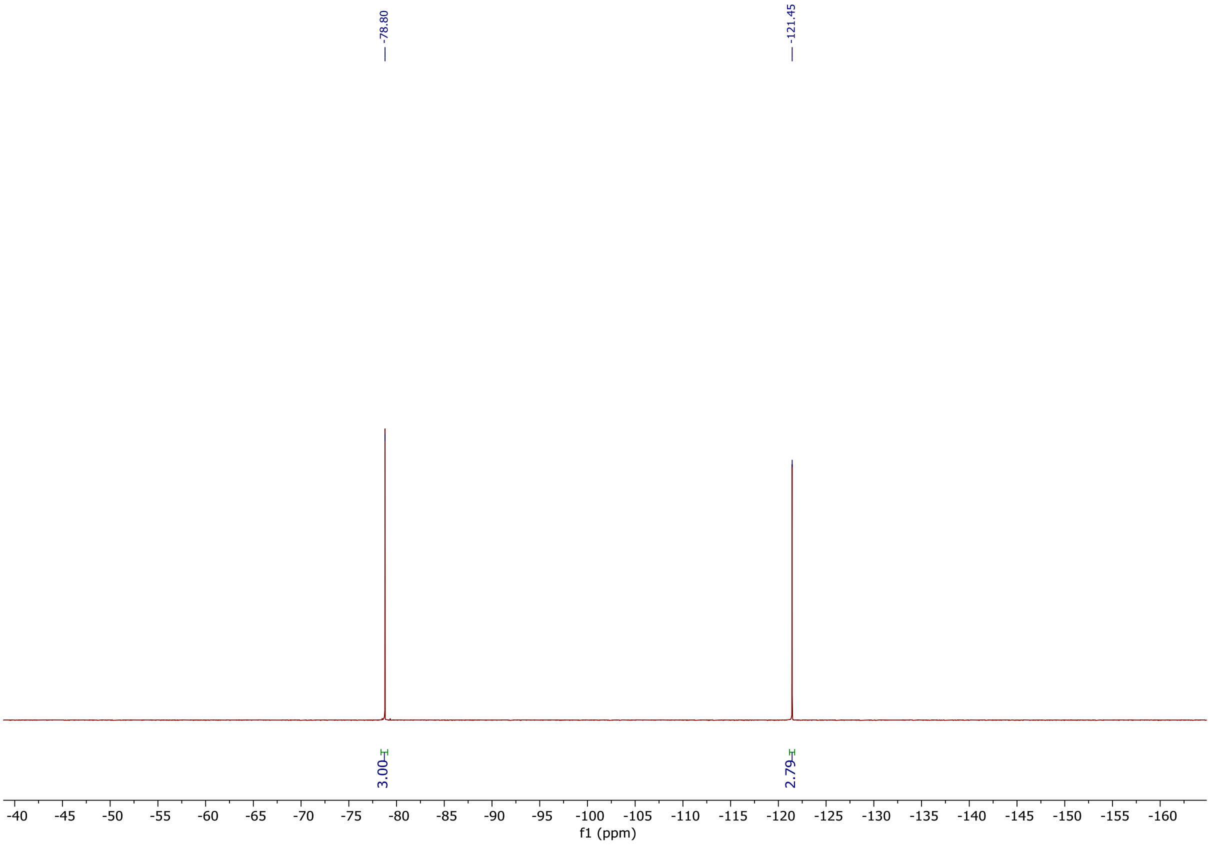

Figure S52. Quantitative ^{19}F NMR spectrum (377 MHz) of KF (10.0 mg, isolated from second run) and NaOTf (10.0 mg, as an internal standard) in D_2O (10 atom% D) indicates 94% purity of KF.

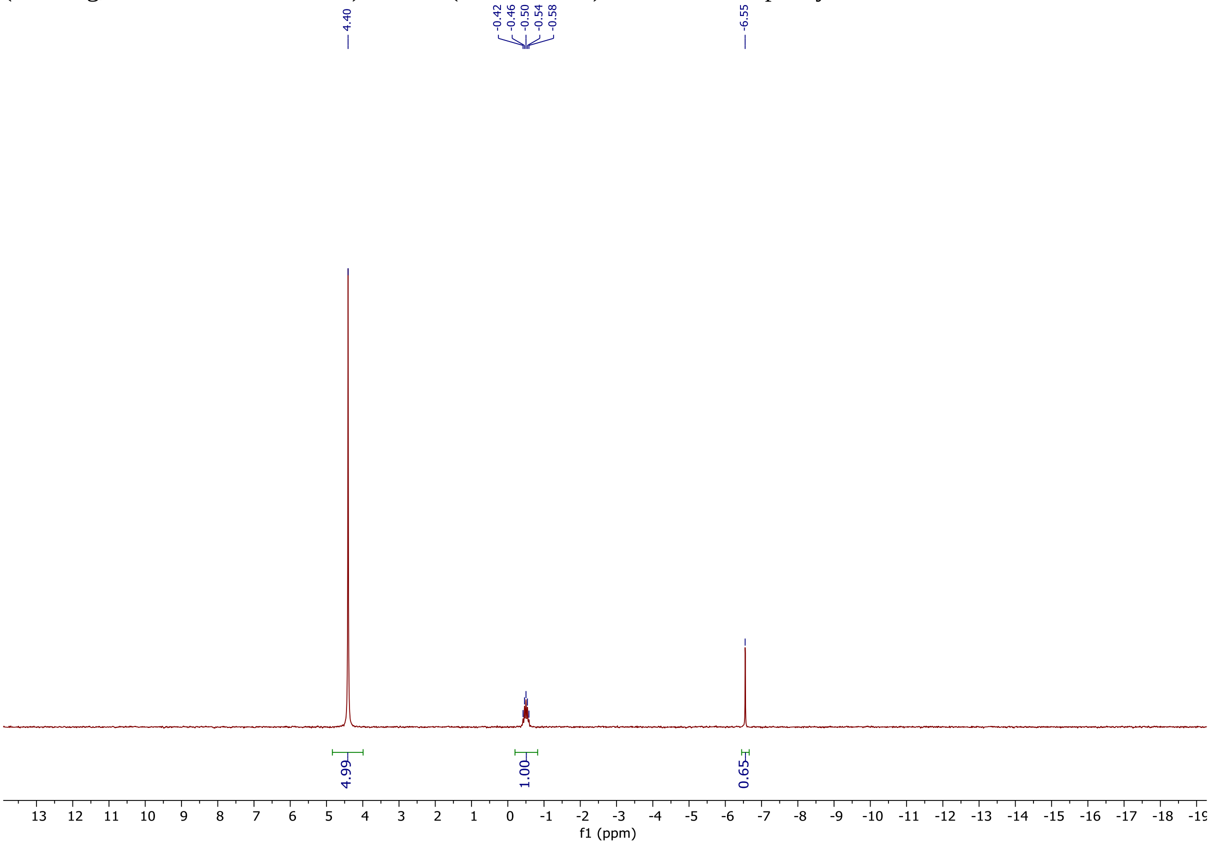

Figure S53. Quantitative ^{31}P NMR spectrum (203 MHz) of an aliquot of second run recovered $K_3PO_4^{CYC}$ (69 mg) and triethyl phosphate (10.0 mg, as an internal standard) in D_2O (10 atom% D) indicates phosphate (δ_P = 4.4, 85%), pyrophosphate (δ_P = -6.5, 5%) recovery, and phosphate (84%) and pyrophosphate (9%) in $K_3PO_4^{CYC}$.

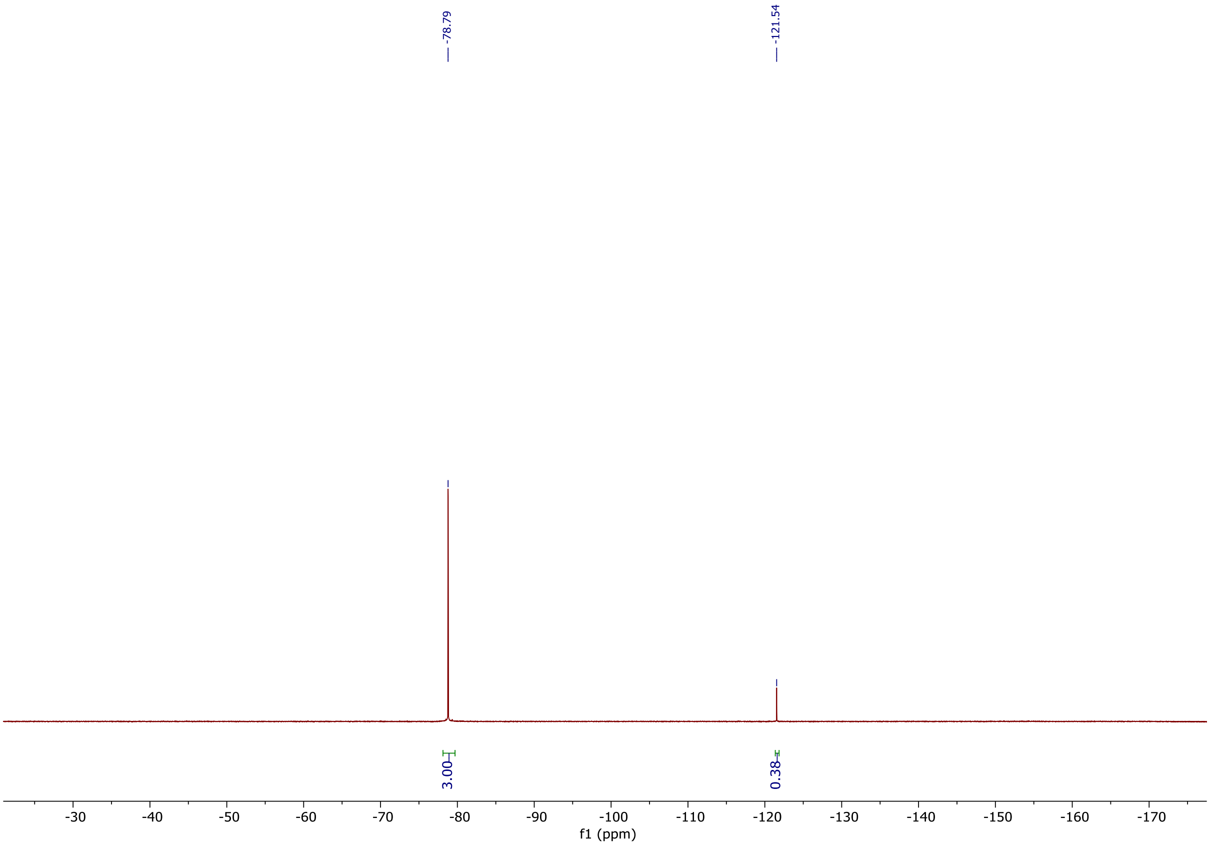

Figure S54. Quantitative ^{19}F NMR spectrum (377 MHz) of second run recovered $K_3PO_4^{CYC}$ (21 mg) and NaOTf (10.0 mg, as an internal standard) in D_2O (10 atom% D) indicates KF (6%) in the second run recovered $K_3PO_4^{CYC}$.

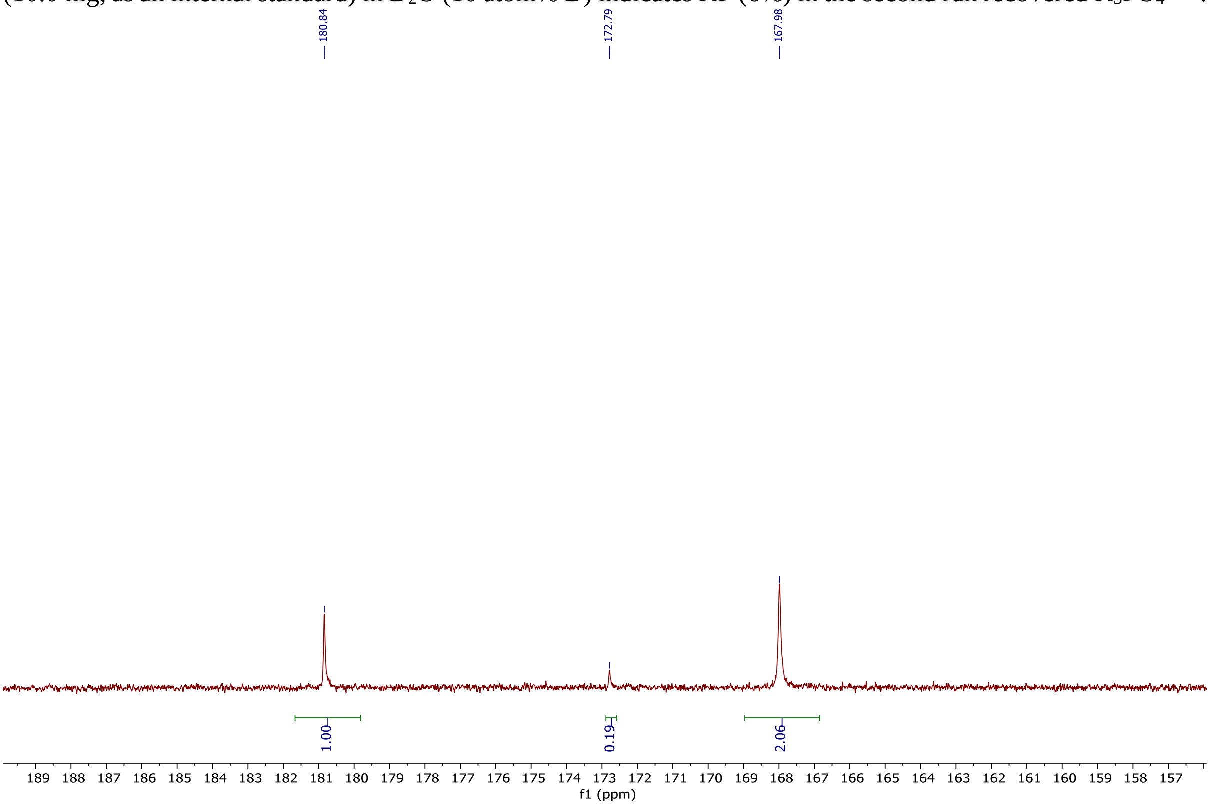

Figure S55. Quantitative $^{13}C\{^1H\}$ NMR spectrum (126 MHz, 1910 scans at 40 s recycle delay) of second run recovered $K_3PO_4^{CYC}$ (350 mg) in D_2O (10 atom% D) with KOAc (200 µL of a 0.509 M solution in D_2O) as an internal standard indicates carbonate (δ_C = 168.0, 8%) and oxalate (δ_C = 172.8, 1%) in the second run recovered $K_3PO_4^{CYC}$.

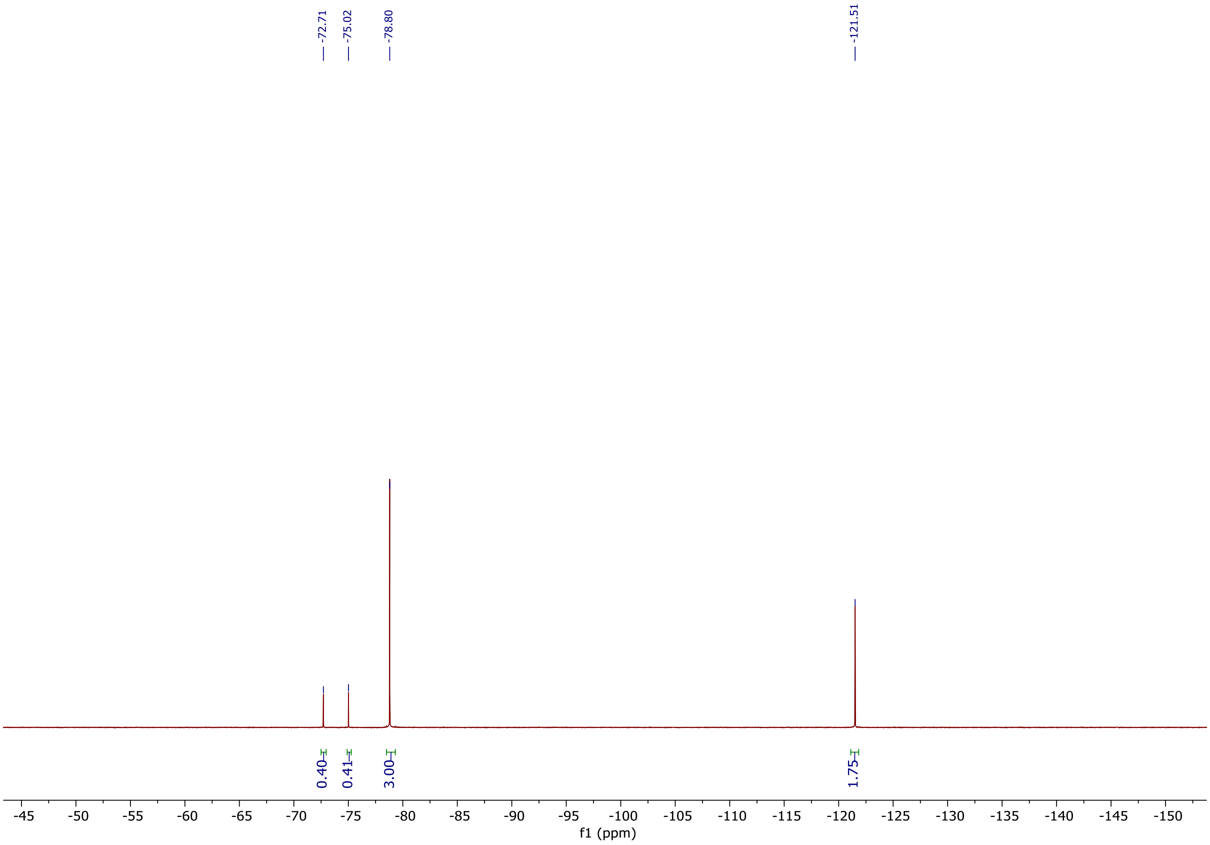

Figure S56. Quantitative ^{19}F NMR spectrum (377 MHz) of an aliquot of third run PTFE-mix^{CYC} (23 mg) in D_2O (10 atom% D) using sodium triflate (10mg) as an internal standard, indicates fluorine content of fluoride (δ_F = −121.5, 61%), and fluorophosphate (δ_P = −73.7 (d, $^1J_{PF}$ = 867 Hz), 28%).

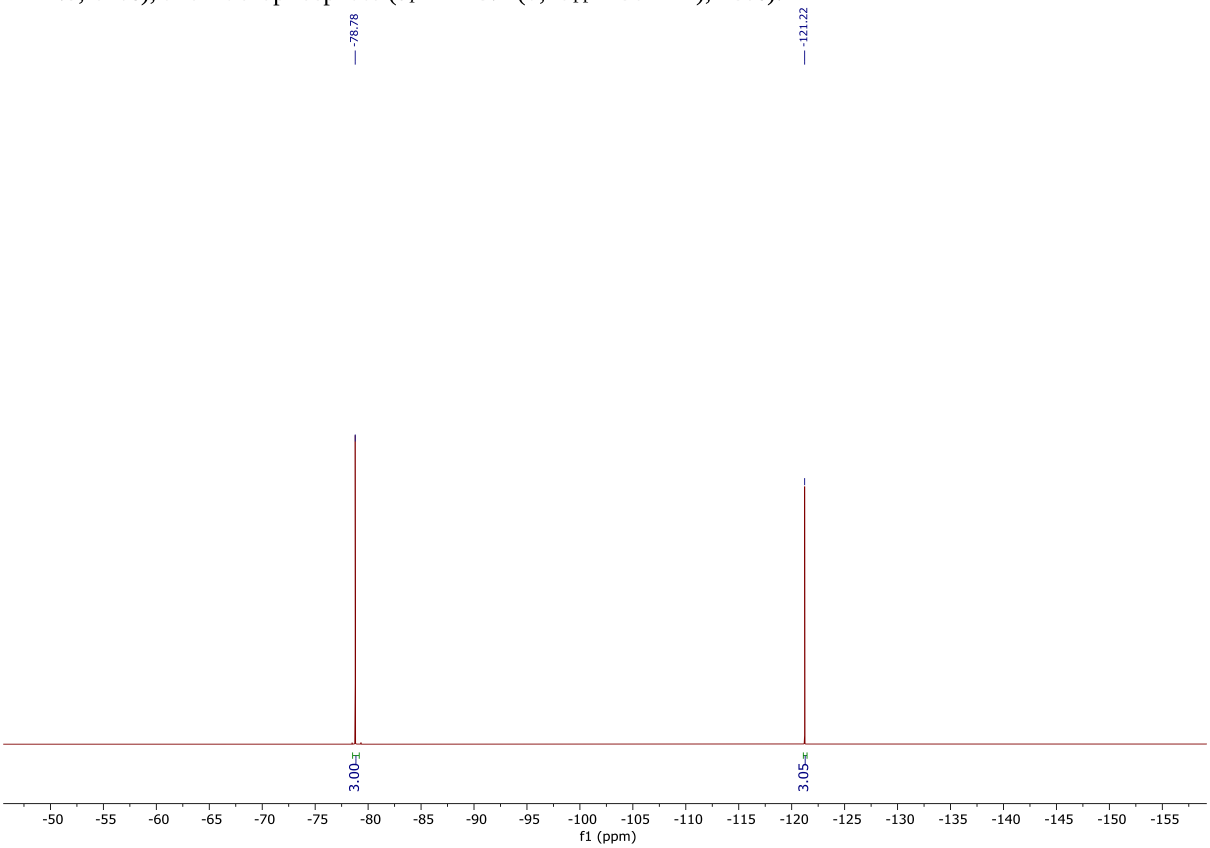

Figure S57. Quantitative ^{19}F NMR spectrum (377 MHz) of KF (11.3 mg, isolated from third run) and NaOTf (10.0 mg, as an internal standard) in D_2O (10 atom% D) indicates 91% purity of KF.

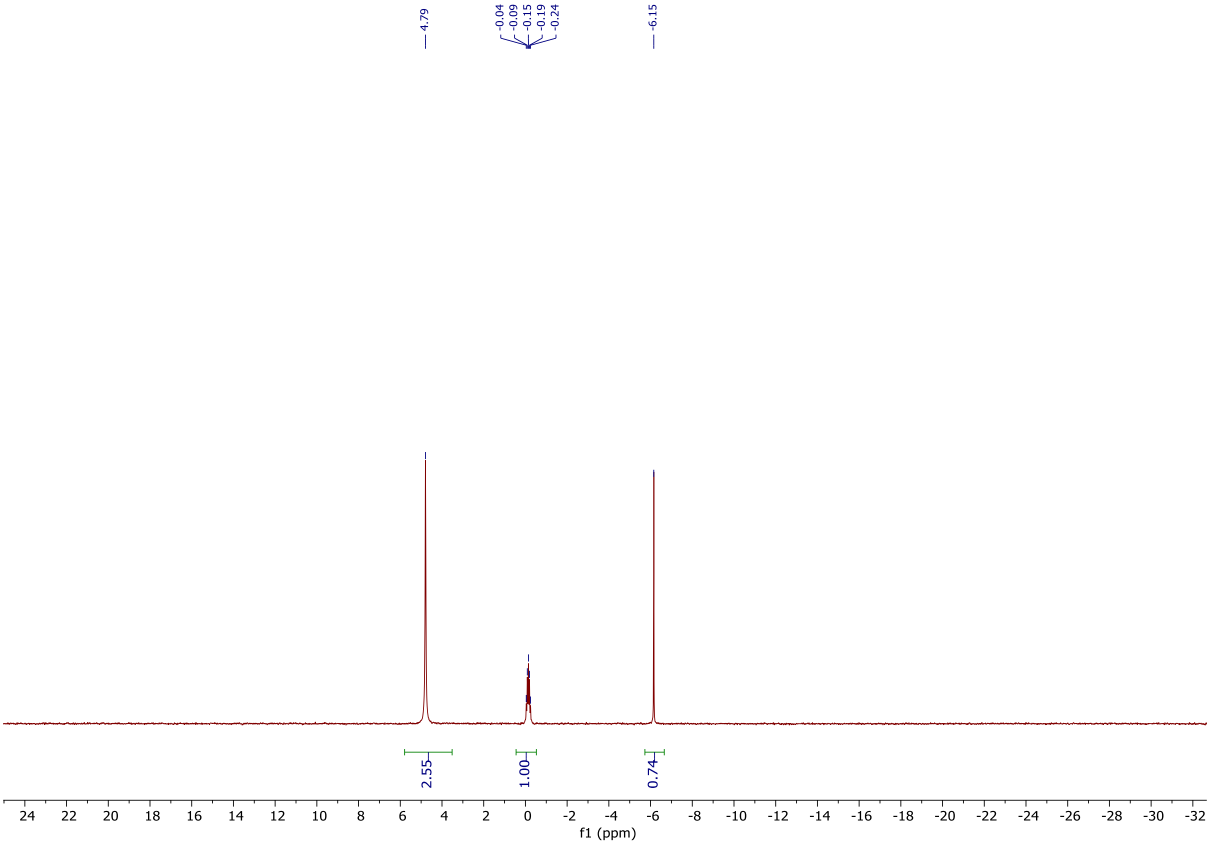

Figure S58. Quantitative ^{31}P NMR spectrum (162 MHz) of an aliquot of third run recovered $K_3PO_4^{CYC}$ (40 mg) and triethyl phosphate (10.0 mg, as an internal standard) in D_2O (10 atom% D) indicates phosphate (δ_P = 4.8, 74%), pyrophosphate (δ_P = -6.2, 11%) recovery, and phosphate (74%) and pyrophosphate (17%) in $K_3PO_4^{CYC}$.

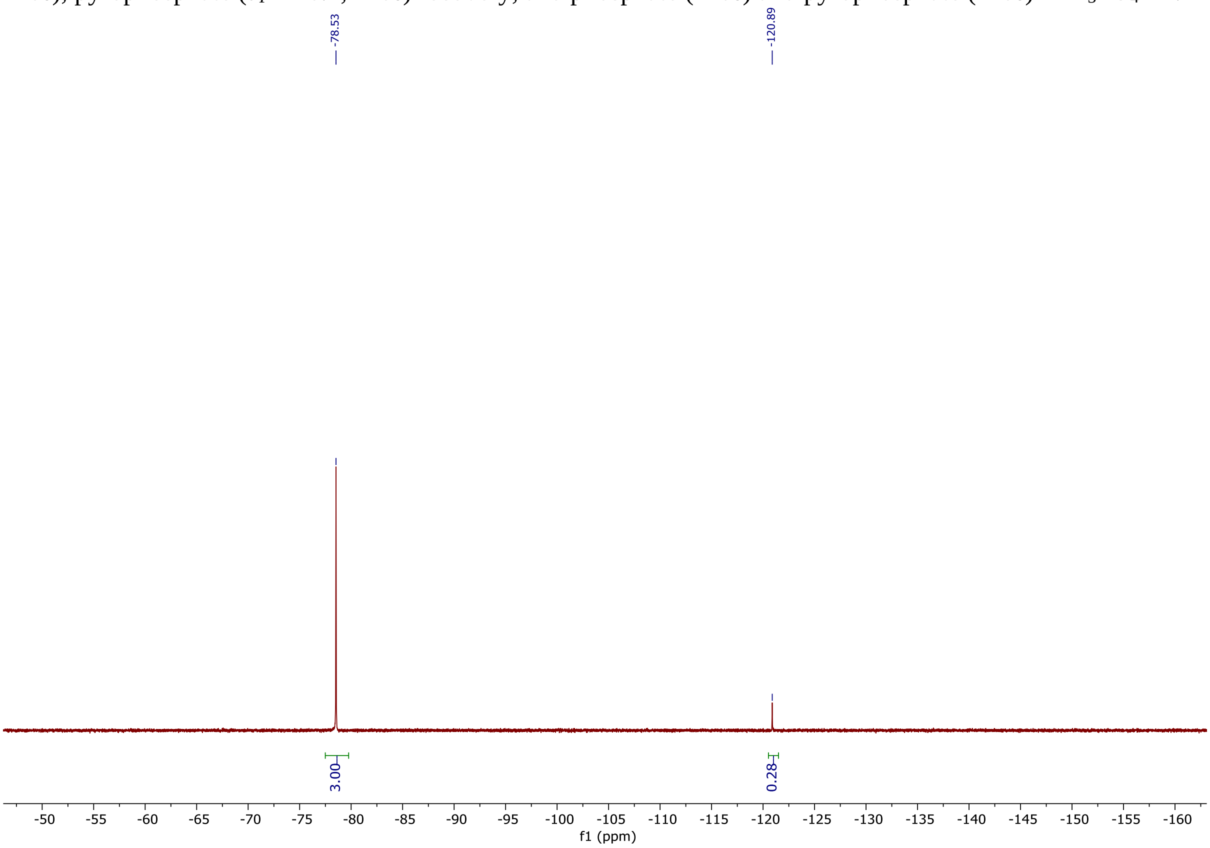

Figure S59. Quantitative ^{19}F NMR spectrum (377 MHz) of third run recovered $K_3PO_4^{CYC}$ (27 mg) and NaOTf (10.0 mg, as an internal standard) in D_2O (10 atom% D) indicates KF (3%) in the third run recovered $K_3PO_4^{CYC}$.

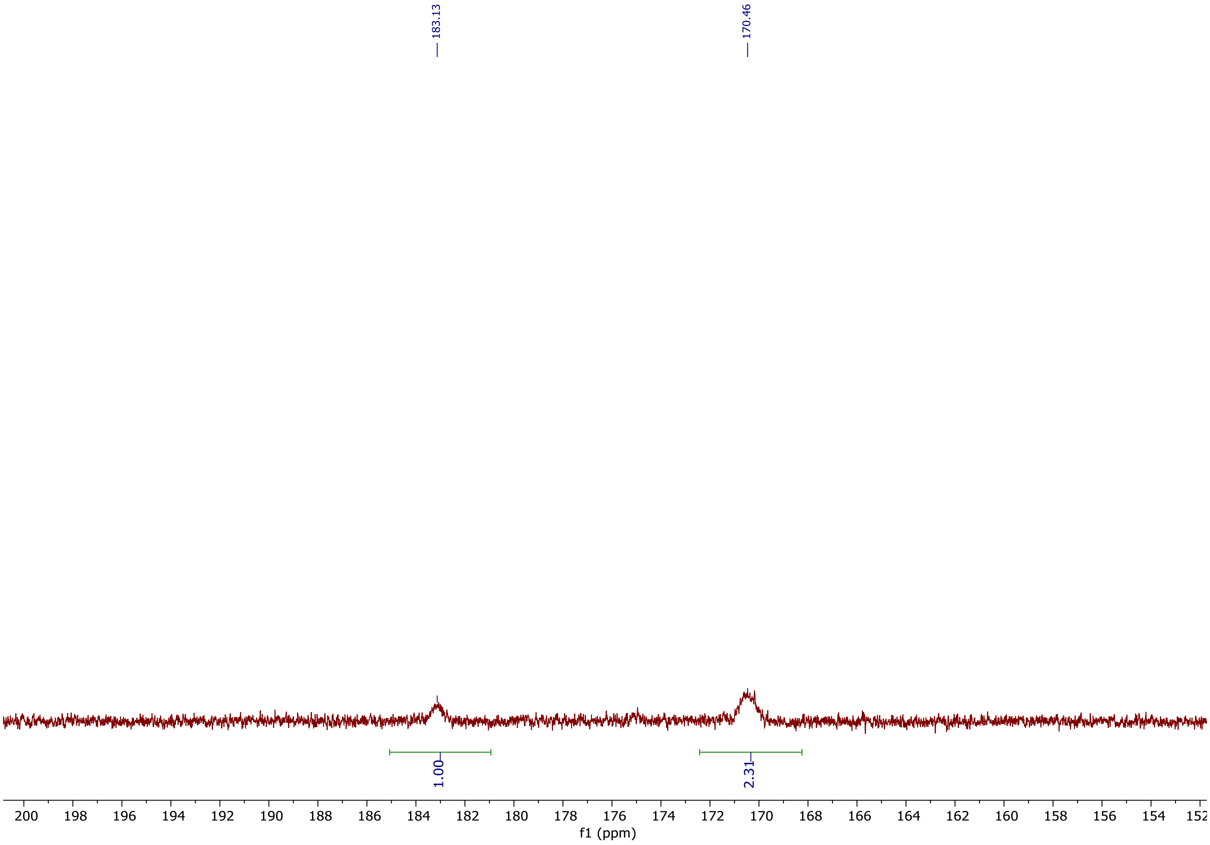

Figure S60. Quantitative $^{13}C\{^{1}H\}$ NMR spectrum (126 MHz, 2200 scans at 40 s recycle delay) of third run recovered $K_3PO_4^{CYC}$ (400 mg) in D_2O (10 atom% D) with KOAc (200 µL of a 0.509 M solution in D_2O) as an internal standard indicates carbonate (δ_C = 170.5, 8%) in the third run recovered $K_3PO_4^{CYC}$.

8. Synthesis of fluorochemicals using fluorinating reagents derived from PFAS

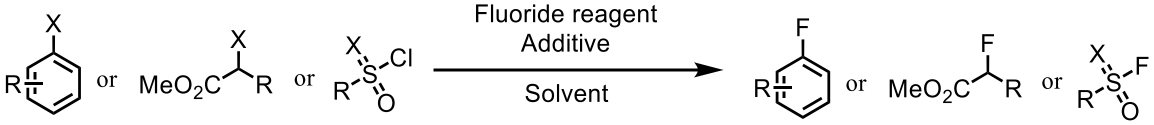

SAFETY NOTE: *DMSO undergoes thermal decomposition at temperatures around its boiling point of 189 °C [61]. The presence of impurities and/or acidic substances can cause it to decompose at significantly lower temperatures and potentially result in uncontrollable autocatalytic decomposition of DMSO, leading to thermal runaway or even explosions. All S_NAr reactions using KF and DMSO as solvent (2.5 mL) were carried out ≤ 130 °C (0.5 mmol scale). All S_NAr reactions using TMAF(tAmylOH) and DMSO as solvent (2.5 mL) were carried out ≤ 100 °C (0.5 mmol scale). All S_NAr reactions using DMSO with heating should be conducted in the presence of a blast shield. DMSO should be avoided for large-scale experimentation, and a suitable alternative should be used.*

General procedure (GP1): To an oven dried Schlenk tube under N_2 atmosphere was added dry fluoride reagent (amount as indicated), the corresponding substrate (0.5 mmol, 1 equiv.), additives (amount as indicated) and anhydrous solvent (amount as indicated). After stirring at 25 °C − 100 °C in an oil bath, the resulting suspension was cooled to room temperature, quenched with brine (10 mL) and extracted with DCM (2 × 5 mL). The combined organic layers were dried over $MgSO_4$, filtered and concentrated in vacuo. The crude product was purified by flash column chromatography on silica gel to afford the corresponding fluorinated product.

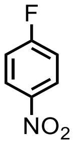

28

Prepared following GP1 using 1,4-dinitrobenzene (84 mg, 0.5 mmol) as substrate, $TMAF^{PTFE}$ (1.5 equiv., 0.75 mmol, 136 mg) as fluoride reagent in anhydrous DMSO (0.2 M). The reaction was stirred at 30 °C for 12 h. Purification by flash column chromatography on silica (2% diethyl ether in pentane) gave 4-fluoronitrobenzene (**28**) as a light-yellow oil (64 mg, 91%).

^{1}H NMR (400 MHz, $CDCl_3$) δ 8.41 – 8.19 (m, 2H), 7.29 – 7.15 (m, 2H).

^{13}C NMR (101 MHz, $CDCl_3$) δ 166.2 (d, *J* = 258 Hz), 144.4, 126.3 (dd, *J* = 10 Hz), 116.4 (d, *J* = 24 Hz).

^{19}F NMR (377 MHz, $CDCl_3$) δ -102.1 (s).

The spectroscopic data are in accordance with those in the literature [62].

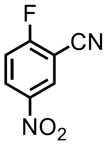

29

Prepared following GP1 using 2-chloro-5-nitrobenzonitrile (91 mg, 0.5 mmol) as substrate, KF^{PTFE} (1.5 equiv., 0.75 mmol, 44 mg) as fluoride reagent, TMACl (5 mol%, 2.7 mg) and 18-crown-6 (5 mol%, 6.6 mg) as additives in anhydrous DMSO (0.2 M). The reaction was stirred at 100 °C for 6 h. Purification by flash column chromatography on silica (2% diethyl ether in pentane) gave 2-fluoro-5-nitrobenzonitrile (**29**) as a colorless oil (71 mg, 86%).

^{1}H NMR (400 MHz, $CDCl_3$) δ 8.51 (dd, *J* = 5.4, 2.8 Hz, 1H), 8.46 (ddd, *J* = 9.2, 4.4, 2.8 Hz, 1H), 7.40 (dd, *J* = 9.2, 7.7 Hz, 1H).

^{13}C NMR (101 MHz, $CDCl_3$) δ 166.2 (d, *J* = 270 Hz), 144.3, 130.5 (d, *J* = 10 Hz), 129.6 (d, *J* = 2 Hz), 117.9 (d, *J* = 22 Hz), 111.71, 103.2 (d, *J* = 18 Hz).

^{19}F NMR (377 MHz, $CDCl_3$) δ -95.8 (m).

The spectroscopic data are in accordance with those in the literature [63].

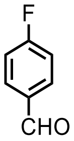

30

Prepared following GP1 using potassium (4-formylphenyl)trifluoroborate (106 mg, 0.5 mmol) as substrate, KF^{PTFE} (4.0 equiv., 2.0 mmol, 116 mg) as fluoride reagent and $Cu(OTf)_2$ (4.0 equiv., 2.0 mmol, 723 mg) as additive in anhydrous CH_3CN (0.083 M). The reaction was stirred at 60 °C for 20 h. Purification by flash column chromatography on silica (2% diethyl ether in pentane) gave 4-fluorobenzaldehyde (**30**) as a colorless oil (35 mg, 56%).

^{1}H NMR (400 MHz, $CDCl_3$) 9.97 (s, 1H), 7.91 (dd, *J* = 8.7, 5.4 Hz, 2H), 7.21 (t, *J* = 8.6 Hz, 2H).

^{13}C NMR (101 MHz, $CDCl_3$) δ 190.6, 166.6 (d, *J* = 256.7 Hz), 133.1 (d, *J* = 2.8 Hz), 132.3 (d, *J* = 9.8 Hz), 116.4 (d, *J* = 22.3 Hz).

^{19}F NMR (377 MHz, $CDCl_3$) δ -102.3 – -102.4 (m).

The spectroscopic data are in accordance with those in the literature [64].

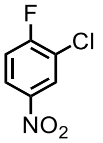

31

Prepared following GP1 using ethyl 1,2-dichloro-4-nitrobenzene (96 mg, 0.5 mmol) as substrate, TMAF(*t*AmylOH)^{PTFE} (1.5 equiv., 0.75 mmol, 136 mg) as fluoride reagent in anhydrous DMSO (0.2 M). The reaction was stirred at 100 °C for 12 h. Purification by flash column chromatography on silica (2% diethyl ether in pentane) gave 2-chloro-1-fluoro-4-nitrobenzene (**31**) as a colorless oil (82 mg, 93%).

^{1}H NMR (400 MHz, $CDCl_3$) δ 8.38 – 8.29 (m, 1H), 8.22 – 8.12 (m, 1H), 7.32 (t, *J* = 8.5 Hz, 1H).

^{13}C NMR (101 MHz, $CDCl_3$) δ 161.9 (d, *J* = 259.9 Hz), 126.7 (d, *J* = 1.7 Hz), 124.1 (d, *J* = 9.0 Hz), 122.6 (d, *J* = 19.7 Hz), 117.2 (d, *J* = 23.4 Hz).

^{19}F NMR (377 MHz, $CDCl_3$) δ -103.9 – -104.0 (m).

The spectroscopic data are in accordance with those of an authentic commercial sample.

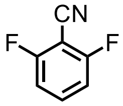

32

Prepared following GP1 using ethyl 2,6-dinitrobenzonitrile (97 mg, 0.5 mmol) as substrate, TMAF(*t*AmylOH)^{PTFE} (2.5 equiv., 1.25 mmol, 226 mg) as fluoride reagent in anhydrous DMSO (0.2 M). The reaction was stirred at 30 °C for 16 h. Purification by flash column chromatography on silica (2% diethyl ether in pentane) gave 2-fluoro-5-nitrobenzonitrile (**32**) as a colorless oil (61 mg, 88%).

^{1}H NMR (400 MHz, $CDCl_3$) δ 7.64 (tt, *J* = 8.6, 6.3 Hz, 1H), 7.13 – 7.05 (m, 2H).

^{13}C NMR (101 MHz, $CDCl_3$) δ 163.3 (dd, *J* = 262, 4 Hz), 135.6 (t, *J* = 10 Hz), 112.2 (dd, *J* = 19, 4 Hz), 109.1, 92.5 (t, *J* = 19 Hz).

^{19}F NMR (377 MHz, $CDCl_3$) δ -103.6.

The spectroscopic data are in accordance with those in the literature [65].

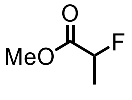

33

Prepared following GP1 using methyl 2-bromopropanoate (56 µL, 0.5 mmol) as substrate, TBAF(*t*BuOH)$_4^{PTFE}$ (1.5 equiv., 0.75 mmol, 419 mg) as fluoride reagent in anhydrous *t*BuOH (0.25 M). The reaction was stirred at 90 °C for 1 h. The product yield (90%) was determined by quantitative ^{19}F-NMR spectroscopy. Spectroscopic data are in accordance with those in literature [31].

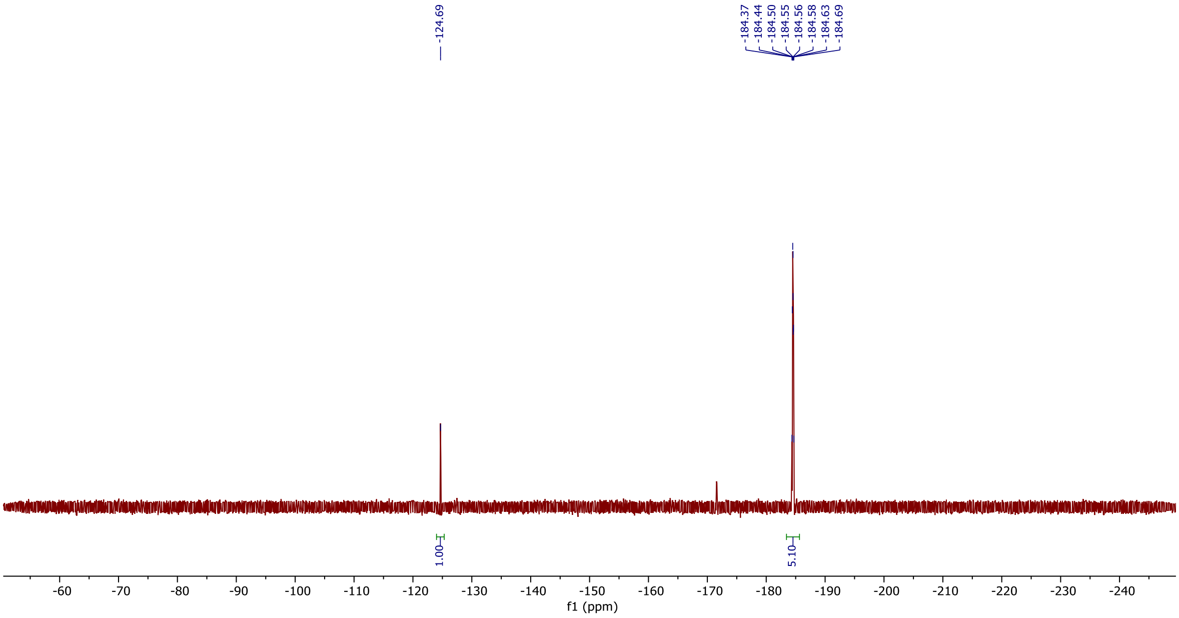

Figure S61. Quantitative ^{19}F NMR spectrum (377 MHz) of reaction mixture with 10 µL 4-fluoroanisole (as an internal standard) in $CDCl_3$.

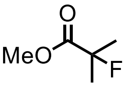

34

Prepared following GP1 using methyl 2-methyl-2-((methylsulfonyl)oxy)propanoate (98 mg, 0.5 mmol) as substrate, KF^{PTFE} (1.5 equiv., 0.75 mmol, 44 mg) as fluoride reagent, 18-crown-6 (1 equiv., 0.5 mmol, 132 mg) as additive in *t*AmylOH (0.25 M). The reaction was stirred at 90 °C for 5 h. The product yield (49%) was determined by quantitative ^{19}F-NMR spectroscopy using 4-fluoroanisole as internal standard. Spectroscopic data are in accordance with those in literature. [66]

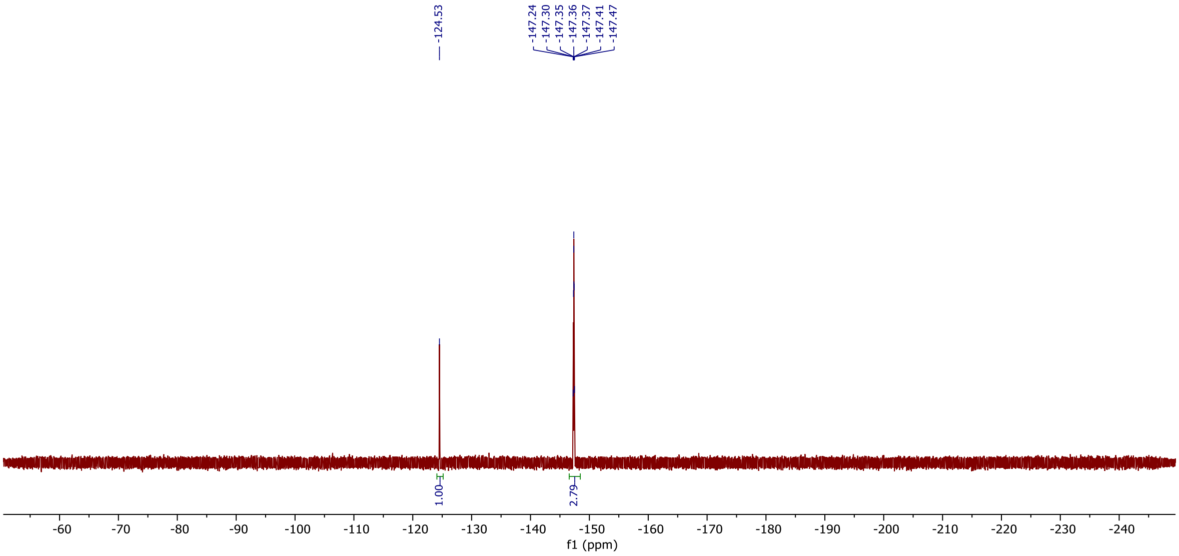

Figure S62. Quantitative ^{19}F NMR spectrum (377 MHz) of reaction mixture with 10 µL 4-fluoroanisole (as an internal standard) in $CDCl_3$.

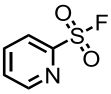

35

Prepared following GP1 using pyridine-2-sulfonyl chloride (89 mg, 0.5 mmol) as substrate, KF^{PTFE} (1.2 equiv., 0.6 mmol, 35 mg) as fluoride reagent, 18-crown-6 (2 equiv., 1.0 mmol, 264 mg) and H_2O (12 equiv., 6.0 mmol, 108 µL) as additives in *t*AmylOH (0.25 M). The reaction was stirred at 100 °C for 10 min. Purification by flash

column chromatography on silica (pentane/ethyl acetate 100:0 to 90:10) gave pyridine-2-sulfonyl fluoride (**35**) as a colorless oil (68 mg, 84%).

Prepared following GP1 using pyridine-2-sulfonyl chloride (89 mg, 0.5 mmol) as substrate, PTFE-mix^{KF} (1.2 equiv., 174 mg) as fluoride reagent, 18-crown-6 (2 equiv., 1.0 mmol, 264 mg) and H_2O (12 equiv., 6.0 mmol, 108 µL) as additives in *t*AmylOH (0.25 M). The reaction was stirred at 100 °C for 10 min. Purification by flash column chromatography on silica (pentane/ethyl acetate 100:0 to 90:10) gave pyridine-2-sulfonyl fluoride (**35**) as a colorless oil (35 mg, 43%).

^{1}H NMR (400 MHz, $CDCl_3$) δ 8.84 (d, *J* = 4.7 Hz, 1H), 8.13 (d, *J* = 7.9 Hz, 1H), 8.06 (tt, *J* = 7.9, 1.5 Hz, 1H), 7.72 (ddd, *J* = 7.7, 4.8, 1.2 Hz, 1H).

^{13}C NMR (101 MHz, $CDCl_3$) δ 151.4 (d, *J* = 30.4 Hz), 151.1, 138.9, 129.4, 124.2 (d, *J* = 2.1 Hz).

^{19}F NMR (377 MHz, $CDCl_3$) δ 55.8 (s).

Spectroscopic data are in accordance with those in literature. [31]

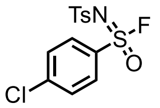

36

Prepared following GP1 using 4-chloro-*N*-tosylbenzenesulfonimidoyl chloride (182 mg, 0.5 mmol) as substrate, KF^{PTFE} (2.0 equiv., 1.0 mmol, 58 mg) as fluoride reagent, 18-crown-6 (2 mol%, 0.01 mmol, 2.6 mg) as additive in CH_3CN (0.4 M). The reaction was stirred at 45 °C for 3 h. Purification by flash column chromatography on silica (pentane/ethyl acetate 100:0 to 90:10) gave 4-chloro-*N*-tosylbenzenesulfonimidoyl fluoride (**36**) as a colorless solid (160 mg, 92%).

Prepared following GP1 using 4-chloro-*N*-tosylbenzenesulfonimidoyl chloride (182 mg, 0.5 mmol) as substrate, PTFE-mix^{KF} (1.2 equiv., 174 mg) as fluoride reagent, 18-crown-6 (2 equiv., 1.0 mmol, 264 mg) and H_2O (12 equiv., 6.0 mmol, 108 µL) as additives in *t*AmylOH (0.25 M). The reaction was stirred at 100 °C for 10 min. Purification by flash column chromatography on silica (pentane/ethyl acetate 100:0 to 90:10) gave 4-chloro-*N*-tosylbenzenesulfonimidoyl fluoride (**36**) as a colorless solid (123 mg, 71%).

^{1}H NMR (400 MHz, $CDCl_3$) δ 7.91 (d, *J* = 8.9 Hz, 2H), 7.86 (d, *J* = 8.4 Hz, 2H), 7.52 (d, *J* = 8.7 Hz, 2H), 7.27 (d, *J* = 8.0 Hz, 2H), 2.37 (s, 3H).

^{13}C NMR (101 MHz, $CDCl_3$) δ 144.5, 143.4, 138.6, 131.4 (d, *J* = 21.7 Hz), 130.2, 129.7, 129.5, 127.1, 21.7.

^{19}F NMR (377 MHz, $CDCl_3$) δ 74.7 (s).

Spectroscopic data are in accordance with those in literature. [31]

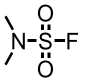

37

Prepared following GP1 using dimethylsulfamoyl chloride (72 mg, 0.5 mmol) as substrate, KF^{PTFE} (1.2 equiv., 0.6 mmol, 35 mg) as fluoride reagent, 18-crown-6 (2 equiv., 1.0 mmol, 264 mg) and H_2O (12 equiv., 6.0 mmol, 108 µL) as additives in *t*AmylOH (0.25 M). The reaction was stirred at 100 °C for 10 min. The product yield (91%) was determined by quantitative ^{19}F-NMR spectroscopy. Spectroscopic data are in accordance with those in literature. [67]

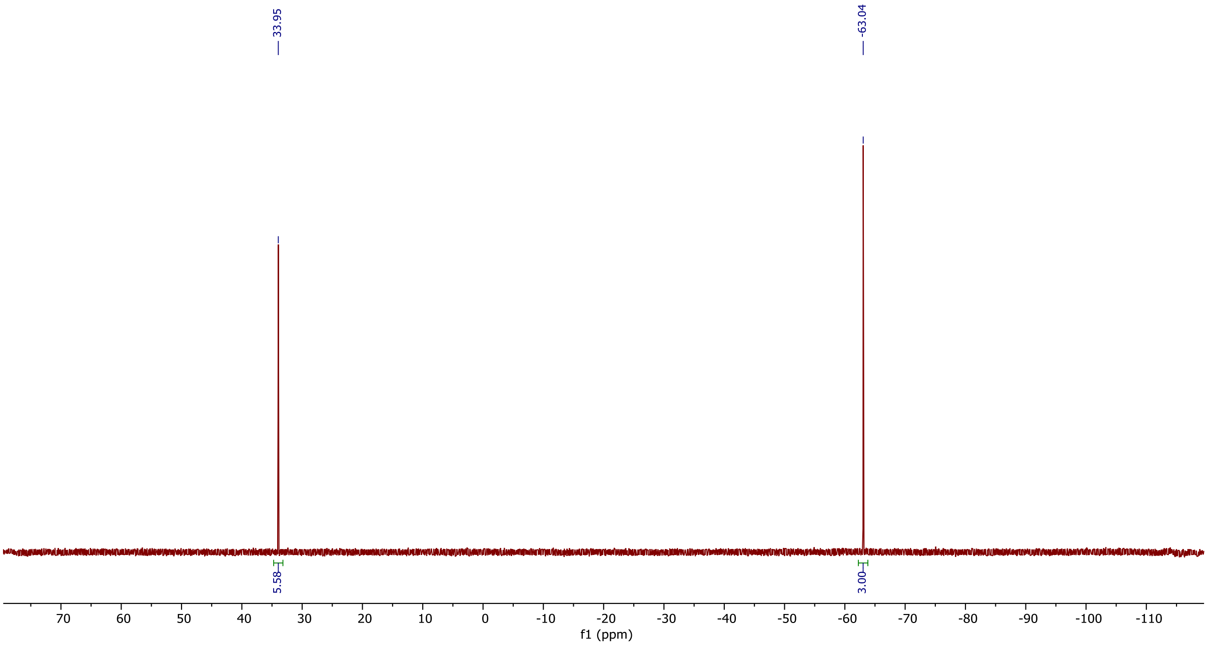

Figure S63. Quantitative ^{19}F NMR spectrum (377 MHz) of reaction mixture (KF^{PTFE}) with 10 µL α,α,α-trifluorotoluene (as an internal standard) in $CDCl_3$.

Prepared following GP1 using dimethylsulfamoyl chloride (72 mg, 0.5 mmol) as substrate, KF^{PFOA} (1.2 equiv., 0.6 mmol, 35 mg) as fluoride reagent, 18-crown-6 (2 equiv., 1.0 mmol, 264 mg) and H_2O (12 equiv., 6.0 mmol, 108 µL) as additives in *t*AmylOH (0.25 M). The reaction was stirred at 100 °C for 10 min. The product yield (92%) was determined by quantitative ^{19}F-NMR spectroscopy. Spectroscopic data are in accordance with those in literature. [67]

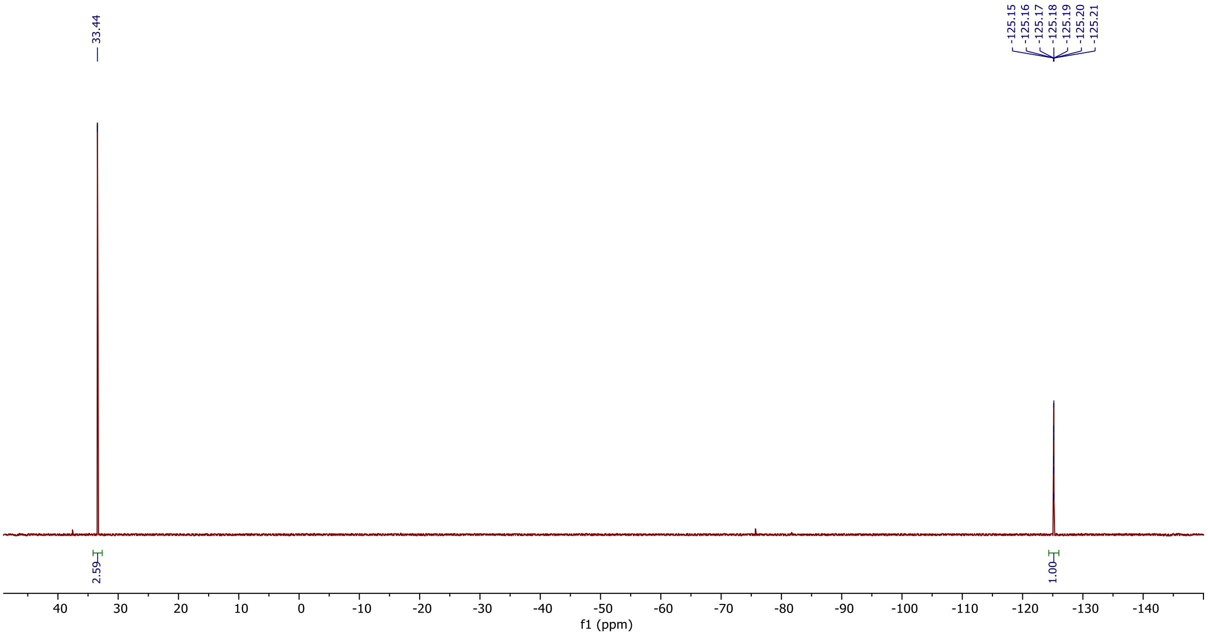

Figure S64. Quantitative ^{19}F NMR spectrum (377 MHz) of reaction mixture (KF^{PFOA}) with 20 µL 4-fluoroanisole (as an internal standard) in $CDCl_3$.

Prepared following GP1 using dimethylsulfamoyl chloride (72 mg, 0.5 mmol) as substrate, KF^{PFAS} (1.2 equiv., 0.6 mmol, 35 mg) as fluoride reagent, 18-crown-6 (2 equiv., 1.0 mmol, 264 mg) and H_2O (12 equiv., 6.0 mmol, 108 µL) as additives in *t*AmylOH (0.25 M). The reaction was stirred at 100 °C for 10 min. The product yield (91%) was determined by quantitative ^{19}F-NMR spectroscopy. Spectroscopic data are in accordance with those in literature. [67]

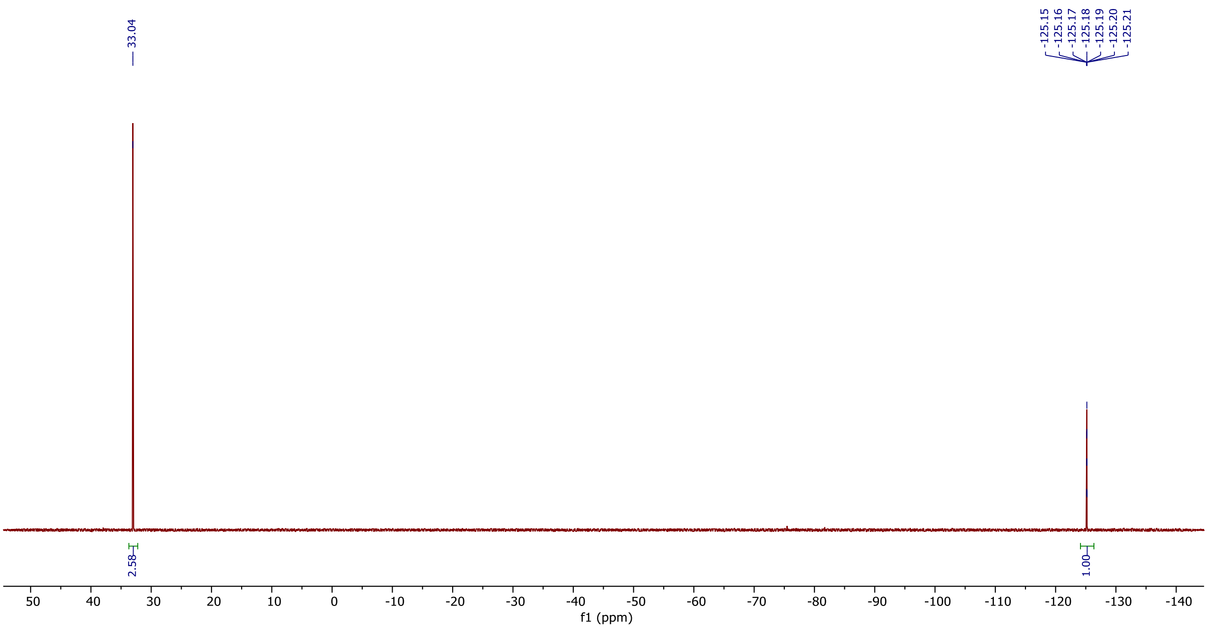

Figure S65. Quantitative ^{19}F NMR spectrum (377 MHz) of reaction mixture (KF^{PFAS}) with 20 µL 4-fluoroanisole (as an internal standard) in $CDCl_3$.

Prepared following GP1 using dimethylsulfamoyl chloride (72 mg, 0.5 mmol) as substrate, PTFE-mix^{KF} (1.2 equiv., 174 mg) as fluoride reagent, 18-crown-6 (2 equiv., 1.0 mmol, 264 mg) and H_2O (12 equiv., 6.0 mmol, 108 µL) as additives in *t*AmylOH (0.25 M). The reaction was stirred at 100 °C for 10 min. The product yield (87%) was determined by quantitative ^{19}F-NMR spectroscopy. Spectroscopic data are in accordance with those in literature. [67]

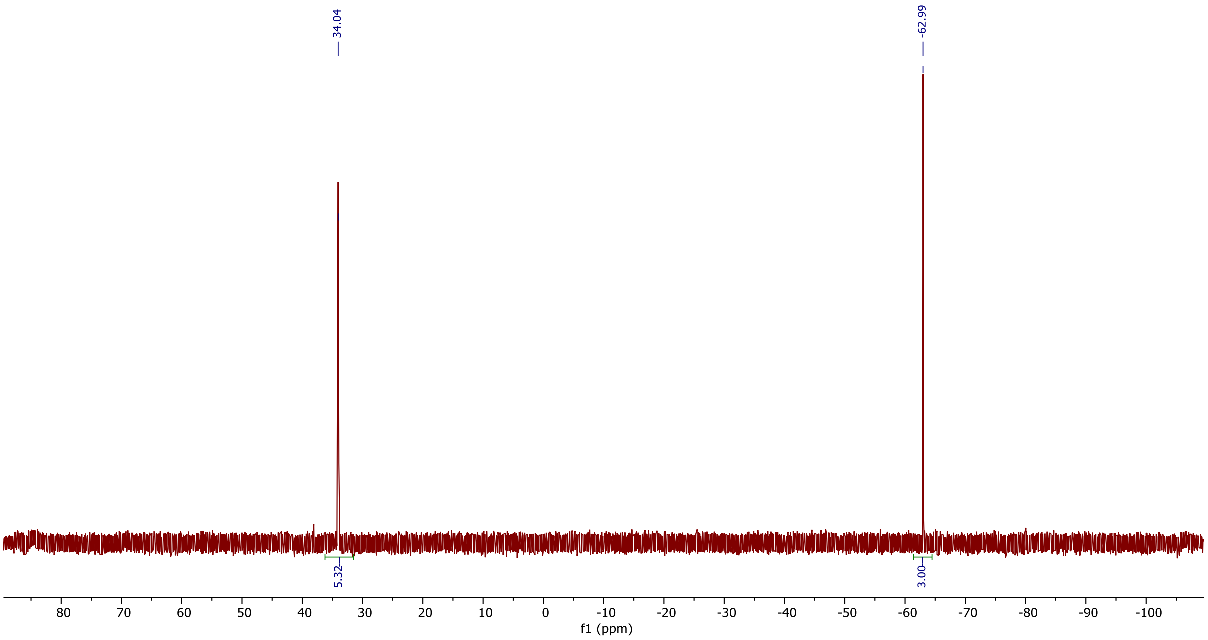

Figure S66. Quantitative ^{19}F NMR spectrum (377 MHz) of reaction mixture (PTFE-mix^{KF}) with 10 µL α,α,α-trifluorotoluene (as an internal standard) in $CDCl_3$.

Prepared following GP1 using dimethylsulfamoyl chloride (72 mg, 0.5 mmol) as substrate, PFOA-mix^{KF} (1.7 equiv., 0.85 mmol, 250 mg) as fluoride reagent, 18-crown-6 (2 equiv., 1.0 mmol, 264 mg) and H_2O (12 equiv., 6.0 mmol, 108 µL) as additives in *t*Amyl-OH (0.25 M). The reaction was stirred at 100 °C for 10 min. The product yield (89%) was determined by quantitative ^{19}F-NMR spectroscopy. Spectroscopic data are in accordance with those in literature. [67]

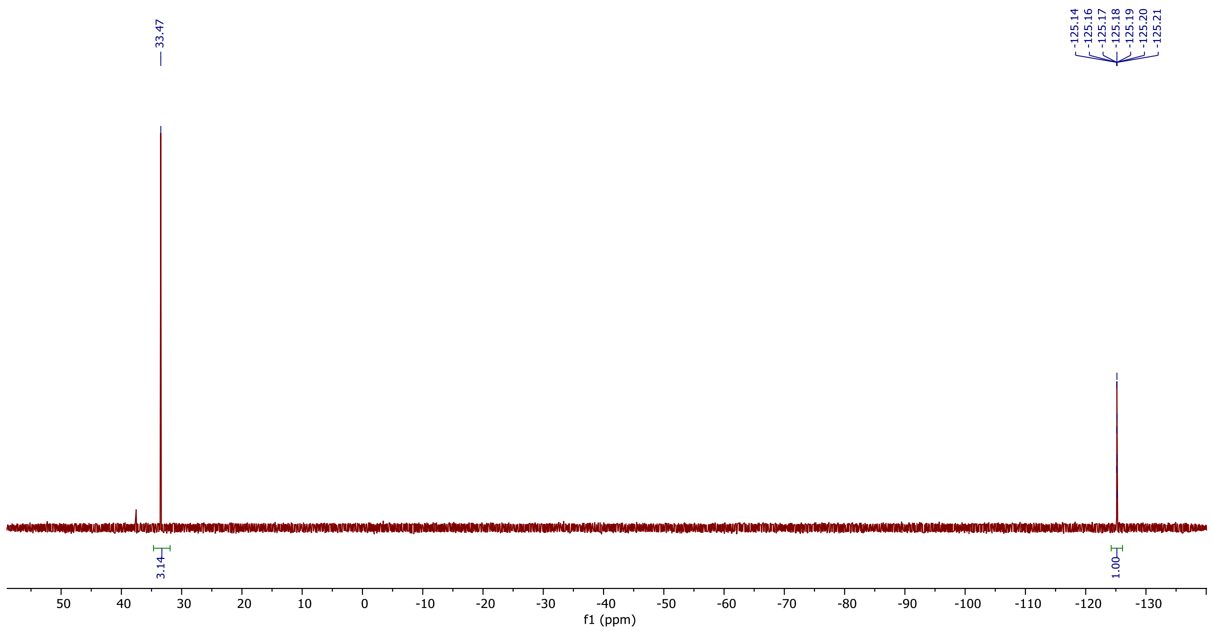

Figure S67. Quantitative ^{19}F NMR spectrum (377 MHz) of reaction mixture (PFOA-mix^{KF}) with 16 μL 4-fluoroanisole (as an internal standard) in $CDCl_3$.

9. Photographic guide for experimental setup (ball milling)

Mechanochemical reactions were conducted using a Retsch MM400 mixer mill, Retsch MM500 Vario mixer mill and an IST636 high-energy mixer mill at the frequency specified.

Typical mechanochemistry setup:

1. PTFE and activator are added to stainless-steel 15 mL milling jar (equipped with rubber O-ring).
2. Two 7 g chrome steel balls are added.
3. Jar is closed, taped on the outside and securely fitted to the mixer mill.
4. Ball milling at 35 Hz for 3 h.
5. Product material (solid powder) scraped from milling jar using a metal spatula.
6. Product material stored in borosilicate glass vial until further usage.

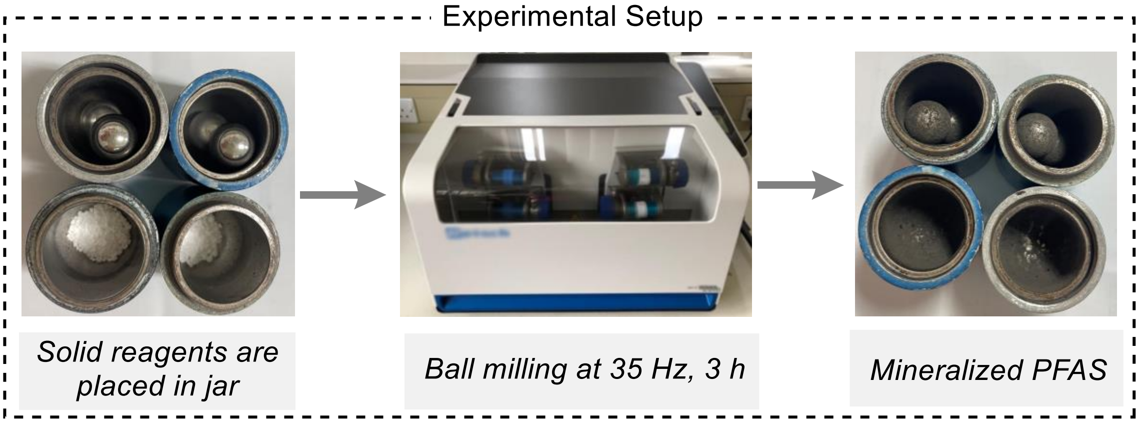

Figure S68. Photographic guide for ball milling of PTFE and K_3PO_4.

10. Mechanochemical destruction of various PFAS

10.1. General procedures

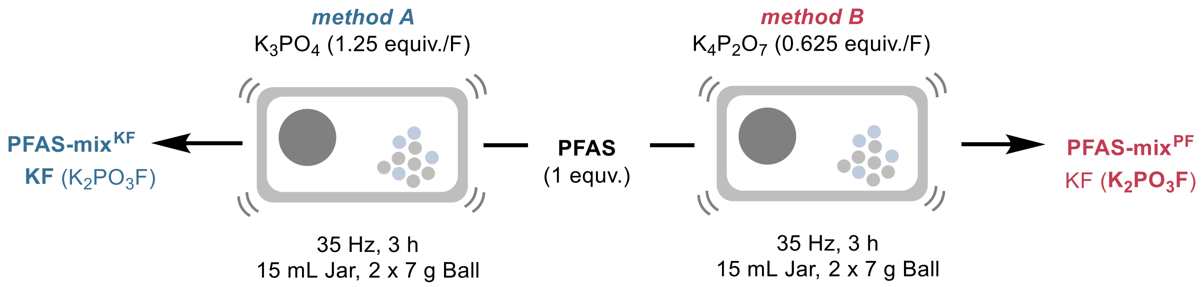

PFAS-mix^{KF} (GP 2): To a 15 mL stainless-steel milling jar was added two chrome steel balls (2 × 7 g), PFAS (1 equiv.) and K_3PO_4 (1.25 equiv./F). The total material of the jar (PTFE and K_3PO_4) was kept constant at 500 mg. The jar was closed and securely fitted to the mill which was set for 3 h at a frequency of 35 Hz. Upon completion, the jar was opened and the powder was collected. An aliquot of PTFE-mix^{KF} (30-70 mg) and sodium triflate (10 mg, as internal standard) was extracted with D_2O (10 atom% D), centrifugated for 15-30 min and analysed by quantitative ^{19}F-NMR spectroscopy.

PFAS-mix^{PF} (GP 3): To a 15 mL stainless-steel milling jar was added two chrome steel balls (2 × 7 g), PFAS (1 equiv.) and $K_4P_2O_7$ (0.625 equiv./F). The total material of the jar (PTFE and $K_4P_2O_7$) was kept constant at 500 mg. The jar was closed and securely fitted to the mill which was set for 3 h at a frequency of 35 Hz. Upon completion, the jar was opened and the powder was collected. An aliquot of PTFE-mix^{PF} (30-70 mg) and sodium triflate (10 mg, as internal standard) was extracted with D_2O (10 atom% D), centrifugated for 15-30 min and analysed by quantitative ^{19}F-NMR spectroscopy.

10.2. PFAS scope

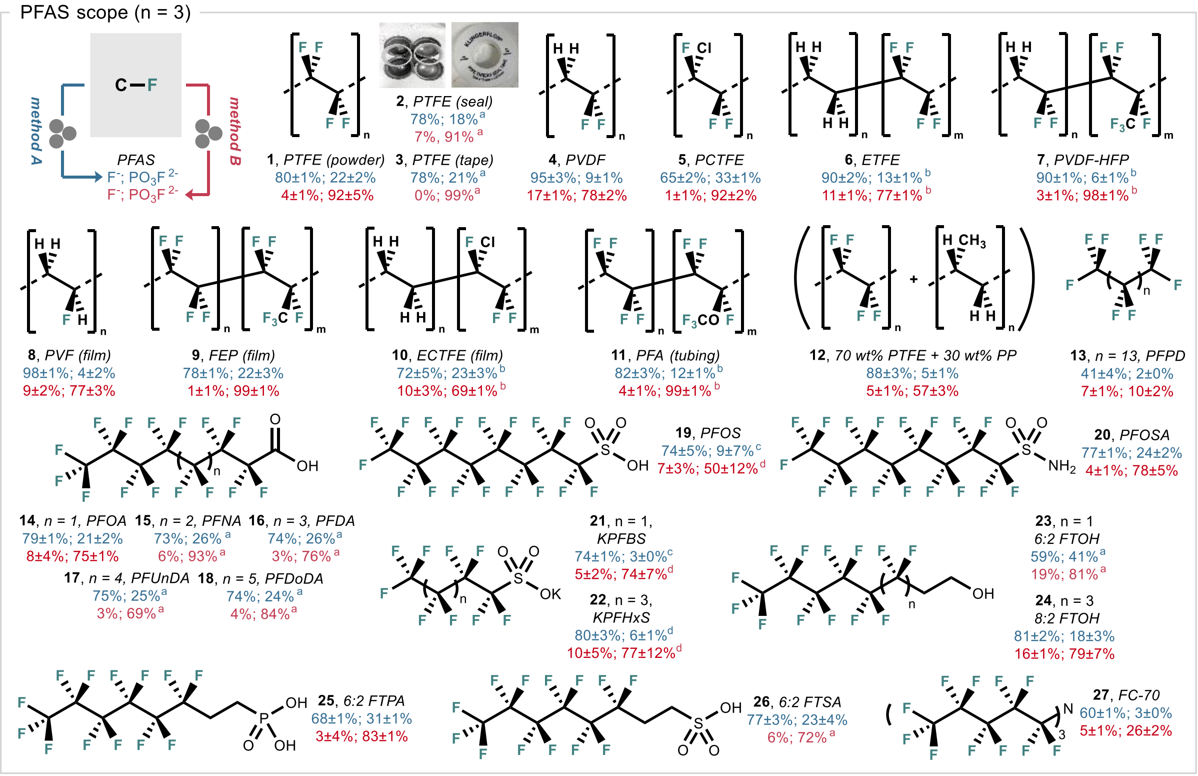

Figure S69. Scope of PFAS destruction. The total yield of released fluoride (both F^- and PO_3F^{2-}) was determined by quantitative ^{19}F NMR spectroscopy (in 10% D_2O in H_2O using NaOTf as an internal standard). Reactions were performed in triplicates and average yields with standard deviations are reported. [a] Reaction was performed once.

[b] The yield was calculated based on the fluorine content of the co-polymer as determined by elemental analysis. [c] The amount of K_3PO_4 was increased to 2 equiv./F. [d] Reaction was carried out for 6 h.

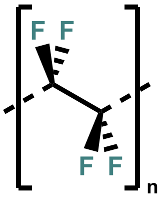

1, *PTFE (powder)*

PTFE-mix^{KF} was prepared according to GP 2 using PTFE (1 equiv., 43 mg) and K_3PO_4 (1.25 equiv./F, 457 mg) in triplicates and analysed by quantitative ^{19}F-NMR spectroscopy. Entry 1: 80% KF, 23% K_2PO_3F; entry 2: 78% KF, 19% K_2PO_3F; entry 3: 81% KF, 23% K_2PO_3F. (Average total yield: quantitative; KF/K_2PO_3F = 3.7:1). **^{19}F NMR** (377 MHz, D_2O, 10% D): δ -73.7 (d, $^1J_{PF}$ = 866 Hz), -120.2. **^{31}P NMR** (162 MHz, D_2O): δ 2.7, 1.0 (d, $^1J_{PF}$ = 866 Hz), -5.5 (d, $^2J_{PP}$ = 20 Hz), -6.3, -20.3 (t, $^2J_{PP}$ = 20 Hz).

PTFE-mix^{PF} was prepared according to GP 3 using PTFE (1 equiv., 54 mg) and $K_4P_2O_7$ (0.625 equiv./F, 446 mg) in triplicates and analysed by quantitative ^{19}F-NMR spectroscopy. Entry 1: 4% KF, 85% K_2PO_3F; entry 2: 5% KF, 98% K_2PO_3F; entry 3: 5% KF, 93% K_2PO_3F. (Average total yield: 96%; KF/K_2PO_3F < 1:20). **^{19}F NMR** (377 MHz, D_2O, 10% D): δ -73.7 (d, $^1J_{PF}$ = 866 Hz), -120.2. **^{31}P NMR** (162 MHz, D_2O): δ 2.7, 1.0 (d, $^1J_{PF}$ = 866 Hz), -5.5 (d, $^2J_{PP}$ = 20 Hz), -6.3, -20.3 (t, $^2J_{PP}$ = 20 Hz).

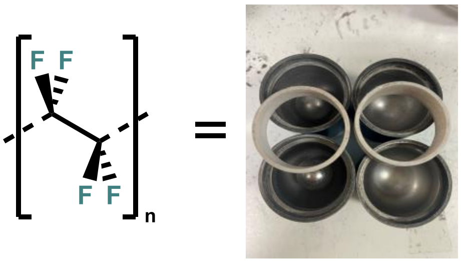

2, *PTFE (seal)*

PTFE-mix^{KF} was prepared according to GP 2 using PTFE seal (1 equiv., 43 mg) and K_3PO_4 (1.25 equiv./F, 457 mg), and analysed by quantitative ^{19}F-NMR spectroscopy. 74% KF, 19% K_2PO_3F. (Total yield: 96%, KF/K_2PO_3F = 4.3:1). **^{19}F NMR** (377 MHz, D_2O, 10% D): δ -73.7 (d, $^1J_{PF}$ = 866 Hz), -120. **^{31}P NMR** (162 MHz, D_2O): δ 2.6, 0.9 (d, $^1J_{PF}$ = 866 Hz), -5.7 (d, $^2J_{PP}$ = 20 Hz), -6.6.

PTFE-mix^{PF} was prepared according to GP 3 using PTFE seal (1 equiv., 54 mg) and $K_4P_2O_7$ (0.625 equiv./F, 446 mg), and analysed by quantitative ^{19}F-NMR spectroscopy. 7% KF, 91% K_2PO_3F. (Total yield: 98%, KF/K_2PO_3F = 1:13). **^{19}F NMR** (377 MHz, D_2O, 10% D): δ -73.7 (d, $^1J_{PF}$ = 866 Hz), -120.7. **^{31}P NMR** (162 MHz, D_2O): δ 2.47, 0.9 (d, $^1J_{PF}$ = 866 Hz).

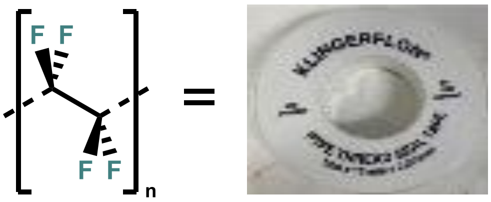

3, *PTFE (tape)*

PTFE-mix^{KF} was prepared according to GP 2 using PTFE tape (1 equiv., 43 mg) and K_3PO_4 (1.25 equiv./F, 457 mg), and analysed by quantitative ^{19}F-NMR spectroscopy. 84% KF, 16% K_2PO_3F. (Total yield: quantitative, KF/K_2PO_3F = 5.3:1). **^{19}F NMR** (377 MHz, D_2O, 10% D): δ -73.7 (d, $^1J_{PF}$ = 866 Hz), -120.5. **^{31}P NMR** (162 MHz, D_2O): δ 2.6, 0.9 (d, $^1J_{PF}$ = 866 Hz), -5.7 (d, $^2J_{PP}$ = 20 Hz), -6.6.

PTFE-mix^{PF} was prepared according to GP 3 using PTFE tape (1 equiv., 54 mg) and $K_4P_2O_7$ (0.625 equiv./F, 446 mg), and analysed by quantitative ^{19}F-NMR spectroscopy. 0% KF, quant. K_2PO_3F. (Total yield: quantitative, KF/K_2PO_3F < 1:20). **^{19}F NMR** (377 MHz, D_2O, 10% D): δ -73.7 (d, $^1J_{PF}$ = 866 Hz). **^{31}P NMR** (162 MHz, D_2O): δ 2.45, 0.9 (d, $^1J_{PF}$ = 866 Hz), -6.3 (d, $^2J_{PP}$ = 20 Hz), -7.1, -20.9 (m).

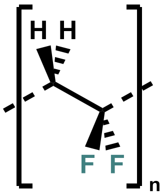

4, *PVDF*

PVDF-mix^{KF} was prepared according to GP 2 using PVDF (1 equiv., 54 mg) and K_3PO_4 (1.25 equiv./F, 446 mg) in triplicates and analysed by quantitative ^{19}F-NMR spectroscopy. Entry 1: 91% KF, 7% K_2PO_3F; entry 2: 99% KF, 9% K_2PO_3F; entry 3: 96% KF, 11% K_2PO_3F. (Average total yield: quantitative; KF/K_2PO_3F = 11:1). **^{19}F NMR** (377 MHz, D_2O, 10% D): δ -73.7 (d, $^1J_{PF}$ = 866 Hz), -120.2. **^{31}P NMR** (162 MHz, D_2O): δ 2.7, 0.8 (d, $^1J_{PF}$ = 866 Hz), -6.5.

PVDF-mix^{PF} was prepared according to GP 3 using PVDF (1 equiv., 67 mg) and $K_4P_2O_7$ (0.625 equiv./F, 433 mg) in triplicates and analysed by quantitative ^{19}F-NMR spectroscopy. Entry 1: 16% KF, 79% K_2PO_3F; entry 2: 19% KF, 80% K_2PO_3F; entry 3: 17% KF, 76% K_2PO_3F. (Average total yield: 96%; KF/K_2PO_3F = 1:4.5). **^{19}F NMR** (377 MHz, D_2O, 10% D): δ -73.7 (d, $^1J_{PF}$ = 866 Hz), -120.4. **^{31}P NMR** (162 MHz, D_2O): δ 1.7, 0.9 (d, $^1J_{PF}$ = 866 Hz).

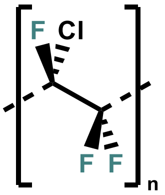

5, *PCTFE*

PCTFE-mix^{KF} was prepared according to GP 2 using PCTFE (1 equiv., 54 mg) and K_3PO_4 (1.25 equiv./F, 445 mg) in triplicates and analysed by quantitative ^{19}F-NMR spectroscopy. Entry 1: 64% KF, 34% K_2PO_3F; entry 2: 67% KF, 32% K_2PO_3F; entry 3: 63% KF, 33% K_2PO_3F. (Average total yield: 98%; KF/K_2PO_3F = 2.0:1). **^{19}F NMR** (377 MHz, D_2O, 10% D): δ -73.7 (d, $^1J_{PF}$ = 866 Hz), -120.2. **^{31}P NMR** (162 MHz, D_2O): δ 2.6, 0.9 (d, $^1J_{PF}$ = 866 Hz), -5.5 (d, $^2J_{PP}$ = 20 Hz), -6.4, -20.4 (t, $^2J_{PP}$ = 20 Hz).

PCTFE-mix^{PF} was prepared according to GP 3 using PCTFE (1 equiv., 79 mg) and $K_4P_2O_7$ (0.625 equiv./F, 421 mg) in triplicates and analysed by quantitative ^{19}F-NMR spectroscopy. Entry 1: 0% KF, 95% K_2PO_3F; entry 2: 1% KF, 94% K_2PO_3F; entry 3: 1% KF, 90% K_2PO_3F. (Average total yield: 94%; KF/K_2PO_3F < 1:20). **^{19}F NMR** (377 MHz, D_2O, 10% D): δ -73.7 (d, $^1J_{PF}$ = 866 Hz). **^{31}P NMR** (162 MHz, D_2O): δ 0.7 (d, $^1J_{PF}$ = 866 Hz), 0.4. -21.5.

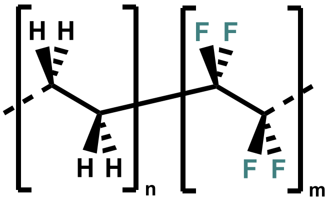

6, *ETFE*

ETFE-mix^{KF} was prepared according to GP 2 using ETFE (1 equiv., 54 mg) and K_3PO_4 (1.25 equiv./F, 446 mg) in triplicates and analysed by quantitative ^{19}F-NMR spectroscopy. Entry 1: 88% KF, 12% K_2PO_3F; entry 2: 91% KF, 13% K_2PO_3F; entry 3: 92% KF, 13% K_2PO_3F. (Average total yield: quantitative; KF/K_2PO_3F = 8.4:1). **^{19}F NMR** (377 MHz, D_2O, 10% D): δ -73.7 (d, $^1J_{PF}$ = 866 Hz), -120.3. **^{31}P NMR** (162 MHz, D_2O): δ 3.6, 0.9 (d, $^1J_{PF}$ = 866 Hz), -5.5 (d, $^2J_{PP}$ = 20 Hz), -6.4, -20.5 (t, $^2J_{PP}$ = 20 Hz).

ETFE-mix^{PF} was prepared according to GP 3 using ETFE (1 equiv., 67 mg) and $K_4P_2O_7$ (0.625 equiv./F, 433 mg) in triplicates and analysed by quantitative ^{19}F-NMR spectroscopy. Entry 1: 12% KF, 77% K_2PO_3F; entry 2: 10% KF, 78% K_2PO_3F; entry 3: 12% KF, 76% K_2PO_3F. (Average total yield: 88%; KF/K_2PO_3F = 1:7.0). **^{19}F NMR** (377 MHz, D_2O, 10% D): δ -73.7 (d, $^1J_{PF}$ = 866 Hz), -120.2. **^{31}P NMR** (162 MHz, D_2O): δ 1.8, 0.9 (d, $^1J_{PF}$ = 866 Hz).

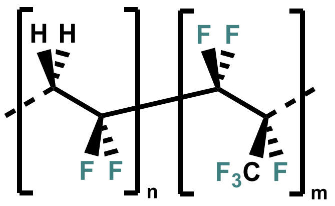

7, *PVDF-HFP*

PVDF-HFP-mix^{KF} was prepared according to GP 2 using PVDF-HFP (1 equiv., 46 mg) and K_3PO_4 (1.25 equiv./F, 454 mg) in triplicates and analysed by quantitative ^{19}F-NMR spectroscopy. Entry 1: 88% KF, 8% K_2PO_3F; entry 2: 87% KF, 5% K_2PO_3F; entry 3: 86% KF, 6% K_2PO_3F. (Average total yield: 93%; KF/K_2PO_3F = 14:1). **^{19}F NMR** (377 MHz, D_2O, 10% D): δ -73.7 (d, $^1J_{PF}$ = 866 Hz), -120.3. **^{31}P NMR** (162 MHz, D_2O): δ 2.7, 0.9 (d, $^1J_{PF}$ = 866 Hz), -5.6 (d, $^2J_{PP}$ = 20 Hz), -6.6.

PVDF-HFP-mix^{PF} was prepared according to GP 3 using PVDF-HFP (1 equiv., 57 mg) and $K_4P_2O_7$ (0.625 equiv./F, 443 mg) in triplicates and analysed by quantitative ^{19}F-NMR spectroscopy. Entry 1: 2% KF, 94% K_2PO_3F; entry 2: 4% KF, 95% K_2PO_3F; entry 3: 3% KF, 94% K_2PO_3F. (Average total yield: 97%; KF/K_2PO_3F < 1:20). **^{19}F NMR** (377 MHz, D_2O, 10% D): δ -73.7 (d, $^1J_{PF}$ = 866 Hz), -120.0. **^{31}P NMR** (162 MHz, D_2O): δ 2.1, 0.9 (d, $^1J_{PF}$ = 866 Hz).

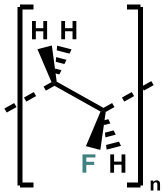

8, *PVF (film)*

PVF-mix^{KF} was prepared according to GP 2 using PVF film (1 equiv., 74 mg) and K_3PO_4 (1.25 equiv./F, 436 mg) in triplicates and analysed by quantitative ^{19}F-NMR spectroscopy. Entry 1: 97% KF, 3% K_2PO_3F; entry 2: 99% KF, 2% K_2PO_3F; entry 3: 98% KF, 6% K_2PO_3F. (Average total yield: quantitative; KF/K_2PO_3F > 20:1). **^{19}F NMR** (377 MHz, D_2O, 10% D): δ -73.7 (d, $^1J_{PF}$ = 866 Hz), -120.5. **^{31}P NMR** (162 MHz, D_2O): δ 2.8, 0.9 (d, $^1J_{PF}$ = 866 Hz), -6.6.

PVF-mix^{PF} was prepared according to GP 3 using PVF film (1 equiv., 91 mg) and $K_4P_2O_7$ (0.625 equiv./F, 409 mg) in triplicates and analysed by quantitative ^{19}F-NMR spectroscopy. Entry 1: 12% KF, 78% K_2PO_3F; entry 2: 7% KF, 74% K_2PO_3F; entry 3: 8% KF, 80% K_2PO_3F. (Average total yield: 86%; KF/K_2PO_3F = 1:8.6). **^{19}F NMR** (377 MHz, D_2O, 10% D): δ -73.7 (d, $^1J_{PF}$ = 866 Hz), -120.0. **^{31}P NMR** (162 MHz, D_2O): δ 2.0, 0.9 (d, $^1J_{PF}$ = 866 Hz).

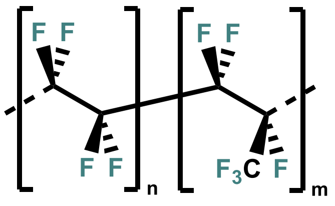

9, *FEP (film)*

FEP-mix^{KF} was prepared according to GP 2 using FEP film (1 equiv., 43 mg) and K_3PO_4 (1.25 equiv./F, 457 mg) in triplicates and analysed by quantitative ^{19}F-NMR spectroscopy. Entry 1: 77% KF, 23% K_2PO_3F; entry 2: 78% KF, 25% K_2PO_3F; entry 3: 82% KF, 18% K_2PO_3F. (Average total yield: quantitative; KF/K_2PO_3F = 3.6:1). **^{19}F NMR** (377 MHz, D_2O, 10% D): δ -73.7 (d, $^1J_{PF}$ = 866 Hz), -120.2. **^{31}P NMR** (162 MHz, D_2O): δ 2.6, 0.9 (d, $^1J_{PF}$ = 866 Hz), -5.6 (d, $^2J_{PP}$ = 20 Hz), -6.5.

FEP-mix^{PF} was prepared according to GP 3 using FEP film (1 equiv., 54 mg) and $K_4P_2O_7$ (0.625 equiv./F, 446 mg). Upon milling completion, yield was analysed by quantitative ^{19}F-NMR spectroscopy. Entry 1: 0% KF, 97% K_2PO_3F; entry 2: 1% KF, 98% K_2PO_3F; entry 3: 2% KF, 99% K_2PO_3F. (Average total yield: quantitative; KF/K_2PO_3F < 1:20). **^{19}F NMR** (377 MHz, D_2O, 10% D): δ -73.7 (d, $^1J_{PF}$ = 866 Hz). **^{31}P NMR** (162 MHz, D_2O): δ 2.5, 0.9 (d, $^1J_{PF}$ = 866 Hz). -6.4 (d, $^2J_{PP}$ = 20 Hz), -7.2, -20.9 (m).

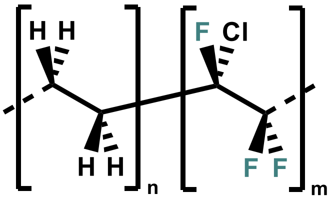

10, *ECTFE (film)*

ECTFE-mix^{KF} was prepared according to GP 2 using ECTFE film (1 equiv., 77 mg) and K_3PO_4 (1.25 equiv./F, 423 mg) in triplicates and analysed by quantitative ^{19}F-NMR spectroscopy. Entry 1: 63% KF, 29% K_2PO_3F; entry 2: 62% KF, 30% K_2PO_3F; entry 3: 64% KF, 24% K_2PO_3F. (Average total yield: 90%; KF/K_2PO_3F = 2.2:1). **^{19}F NMR** (377 MHz, D_2O, 10% D): δ -73.7 (d, $^1J_{PF}$ = 866 Hz), -120.2. **^{31}P NMR** (162 MHz, D_2O): δ 2.6, 0.9 (d, $^1J_{PF}$ = 866 Hz), -6.7.

ECTFE-mix^{PF} was prepared according to GP 3 using ECTFE film (1 equiv., 86 mg) and $K_4P_2O_7$ (0.625 equiv./F, 414 mg) in triplicates and analysed by quantitative ^{19}F-NMR spectroscopy. Entry 1: 7% KF, 68% K_2PO_3F; entry 2: 7% KF, 66% K_2PO_3F; entry 3: 7% KF, 55% K_2PO_3F. (Average total yield: 70%; KF/K_2PO_3F = 1:9). **^{19}F NMR** (377 MHz, D_2O, 10% D): δ -73.7 (d, $^1J_{PF}$ = 866 Hz), -121.9. **^{31}P NMR** (162 MHz, D_2O): δ 1.09, 0.9 (d, $^1J_{PF}$ = 866 Hz), -8.0.

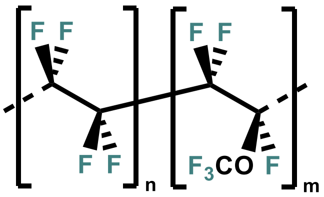

11, *PFA (tubing)*

PFA-mix^{KF} was prepared according to GP 2 using PFA tube (1 equiv., 45.6 mg) and K_3PO_4 (1.25 equiv./F, 454.4 mg) in triplicates and analysed by quantitative ^{19}F-NMR spectroscopy. Entry 1: 85% KF, 12% K_2PO_3F; entry 2: 85% KF, 14% K_2PO_3F; entry 3: 91% KF, 15% K_2PO_3F. (Average total yield: quantitative; KF/K_2PO_3F = 6.2:1). **^{19}F NMR** (377 MHz, D_2O, 10% D): δ -73.7 (d, $^1J_{PF}$ = 866 Hz), -121.7. **^{31}P NMR** (162 MHz, D_2O): δ 2.6, 0.9 (d, $^1J_{PF}$ = 866 Hz), -5.6 (d, $^2J_{PP}$ = 20 Hz), -6.6.

PFA-mix^{PF} was prepared according to GP 3 using PFA tube (1 equiv., 57 mg) and $K_4P_2O_7$ (0.625 equiv./F, 443 mg) in triplicates and analysed by quantitative ^{19}F-NMR spectroscopy. Entry 1: 4% KF, 106% K_2PO_3F; entry 2: 4% KF, 105% K_2PO_3F; entry 3: 3% KF, 105% K_2PO_3F. (Average total yield: quantitative; KF/K_2PO_3F < 1:20). **^{19}F NMR** (377 MHz, D_2O, 10% D): δ -73.7 (d, $^1J_{PF}$ = 866 Hz), -121.7. **^{31}P NMR** (162 MHz, D_2O): δ 2.6, 0.9 (d, $^1J_{PF}$ = 866 Hz), -7.0.

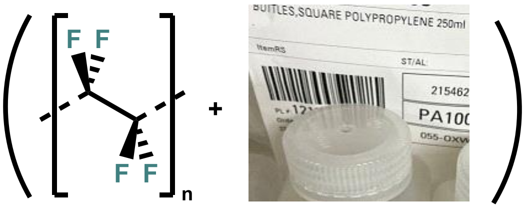

12, *70 wt% PTFE + 30 wt% PP*

PTFE-PP-mix^{KF} was prepared according to GP 2 using polypropylene PP (1 equiv., 30 wt%, 17 mg), PTFE (1 equiv., 42 mg) and K_3PO_4 (1.25 equiv./F, 441 mg) in triplicates and analysed by quantitative ^{19}F-NMR spectroscopy. Entry 1: 93% KF, 5% K_2PO_3F; entry 2: 85% KF, 4% K_2PO_3F; entry 3: 88% KF, 5% K_2PO_3F. (Average total yield: 93%; KF/K_2PO_3F = 19:1). **^{19}F NMR** (377 MHz, D_2O, 10% D): δ -73.7 (d, $^1J_{PF}$ = 866 Hz), -120.2. **^{31}P NMR** (162 MHz, D_2O): δ 2.7, 0.9 (d, $^1J_{PF}$ = 866 Hz), -5.7 (d, $^2J_{PP}$ = 20 Hz), -6.5.

PTFE-PP-mix^{PF} was prepared according to GP 3 using polypropylene PP (1 equiv., 30 wt%, 21.7 mg), PTFE (1 equiv., 51.7 mg) and $K_4P_2O_7$ (0.625 equiv./F, 426 mg) in triplicates and analysed by quantitative ^{19}F-NMR spectroscopy. Entry 1: 6% KF, 53% K_2PO_3F; entry 2: 3% KF, 59% K_2PO_3F; entry 3: 5% KF, 60% K_2PO_3F.

(Average total yield: 62%; KF/K_2PO_3F = 1:12). **^{19}F NMR** (377 MHz, D_2O, 10% D): δ -73.7 (d, $^1J_{PF}$ = 866 Hz), -120.2. **^{31}P NMR** (162 MHz, D_2O): δ 2.5, 0.9 (d, $^1J_{PF}$ = 866 Hz), -6.0 (d, $^2J_{PP}$ = 20 Hz), -6.9, -20.8 (m).

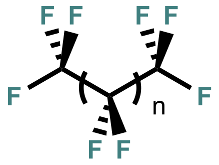

13, *n = 13, PFPD*

PFPD-mix^{KF} was prepared according to GP 2 using PFPD (1 equiv., 43 mg) and K_3PO_4 (1.25 equiv./F, 457 mg) in triplicates and analysed by quantitative ^{19}F-NMR spectroscopy. Entry 1: 45% KF, 2% K_2PO_3F; entry 2: 43% KF, 2% K_2PO_3F; entry 3: 36% KF, 2% K_2PO_3F. (Average total yield: 43%; KF/K_2PO_3F > 20:1). **^{19}F NMR** (377 MHz, D_2O, 10% D): δ -73.7 (d, $^1J_{PF}$ = 866 Hz), -120.0. **^{31}P NMR** (162 MHz, D_2O): δ 3.9, 0.9 (d, $^1J_{PF}$ = 866 Hz), -6.4.

PFPD-mix^{PF} was prepared according to GP 3 using PFPD (1 equiv., 54 mg) and $K_4P_2O_7$ (0.625 equiv./F, 446 mg). Upon milling completion, yield was analysed by quantitative ^{19}F-NMR spectroscopy. Entry 1: 7% KF, 12% K_2PO_3F; entry 2: 8% KF, 7% K_2PO_3F; entry 3: 6% KF, 10% K_2PO_3F. (Average total yield: 17%; KF/K_2PO_3F = 1:1.4). **^{19}F NMR** (377 MHz, D_2O, 10% D): δ -73.7 (d, $^1J_{PF}$ = 866 Hz), -120.0. **^{31}P NMR** (162 MHz, D_2O): δ 2.5, 0.9 (d, $^1J_{PF}$ = 866 Hz), -5.7 (d, $^2J_{PP}$ = 20 Hz), -6.7, -20.6 (t, $^2J_{PP}$ = 20 Hz).

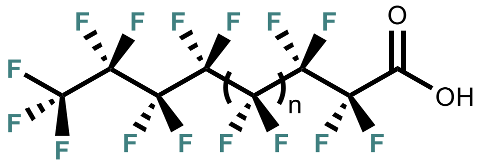

14, *n = 1, PFOA*

PFOA-mix^{KF} was prepared according to GP 2 using PFOA (1 equiv., 47 mg) and K_3PO_4 (1.25 equiv./F, 453 mg) in triplicates and analysed by quantitative ^{19}F-NMR spectroscopy. Entry 1: 78% KF, 20% K_2PO_3F; entry 2: 80% KF, 24% K_2PO_3F; entry 3: 78% KF, 20% K_2PO_3F. (Average total yield: quantitative; KF/K_2PO_3F = 3.7:1). **^{19}F NMR** (377 MHz, D_2O, 10% D): δ -73.7 (d, $^1J_{P\text{-}F}$ = 866 Hz), -120.0. **^{31}P NMR** (162 MHz, D_2O): δ 2.6, 0.9 (d, $^1J_{PF}$ = 866 Hz), -5.6 (d, $^2J_{PP}$ = 20 Hz), -6.5, -20.5 (m).

PFOA-mix^{PF} was prepared according to GP 3 using PFOA (1 equiv., 59 mg) and $K_4P_2O_7$ (0.625 equiv./F, 441 mg). Upon milling completion, yield was analysed by quantitative ^{19}F-NMR spectroscopy. Entry 1: 14% KF, 74% K_2PO_3F; entry 2: 5% KF, 77% K_2PO_3F; entry 3: 6% KF, 75% K_2PO_3F. (Average total yield: 83%; KF/K_2PO_3F = 1:9.2). **^{19}F NMR** (377 MHz, D_2O, 10% D): δ -73.7 (d, $^1J_{PF}$ = 866 Hz), -120.0. **^{31}P NMR** (162 MHz, D_2O): δ 2.1, 0.9 (d, $^1J_{PF}$ = 866 Hz).

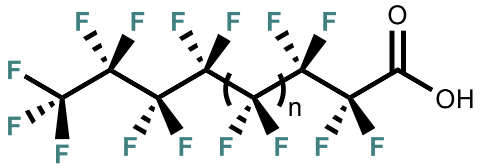

15, *n = 2, PFNA*

PFNA-mix^{KF} was prepared according to GP 2 using PFNA (1 equiv., 47 mg) and K_3PO_4 (1.25 equiv./F, 453 mg), and analysed by quantitative ^{19}F-NMR spectroscopy. 73% KF, 26% K_2PO_3F. (Total yield: 99%; KF/K_2PO_3F = 2.8:1). **^{19}F NMR** (377 MHz, D_2O, 10% D): δ -73.7 (d, $^1J_{PF}$ = 866 Hz), -120.0. **^{31}P NMR** (162 MHz, D_2O): δ 2.6, 0.9 (d, $^1J_{PF}$ = 866 Hz), -5.5 (d, $^2J_{PP}$ = 20 Hz), -6.4, -20.4 (m).

PFNA-mix^{PF} was prepared according to GP 3 using PFNA (1 equiv., 58 mg) and $K_4P_2O_7$ (0.625 equiv./F, 442 mg), and analysed by quantitative ^{19}F-NMR spectroscopy. 6% KF, 93% K_2PO_3F. (Total yield: 99%; KF/K_2PO_3F = 1:16). **^{19}F NMR** (377 MHz, D_2O, 10% D): δ -73.7 (d, $^1J_{PF}$ = 866 Hz), -120.0. **^{31}P NMR** (162 MHz, D_2O): δ 1.7, 0.9 (d, $^1J_{PF}$ = 866 Hz).

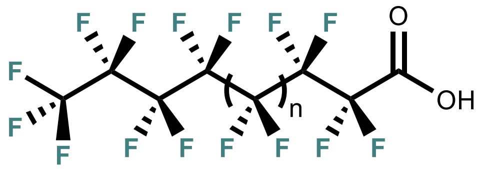

16, *n = 3, PFDA*

PFDA-mix^{KF} was prepared according to GP 2 using PFDA (1 equiv., 46 mg) and K_3PO_4 (1.25 equiv./F, 454 mg), and analysed by quantitative ^{19}F-NMR spectroscopy. 74% KF, 26% K_2PO_3F. (Total yield: quantitative; KF/K_2PO_3F = 2.8:1). **^{19}F NMR** (377 MHz, D_2O, 10% D): δ -73.7 (d, $^1J_{PF}$ = 866 Hz), -120.0. **^{31}P NMR** (162 MHz, D_2O): δ 2.6, 0.9 (d, $^1J_{PF}$ = 866 Hz), -5.6 (d, $^2J_{PP}$ = 20 Hz), -6.5, -20.5 (m).

PFDA-mix^{PF} was prepared according to GP 3 using PFDA (1 equiv., 58 mg) and $K_4P_2O_7$ (0.625 equiv./F, 442 mg), and analysed by quantitative ^{19}F-NMR spectroscopy. 3% KF, 76% K_2PO_3F. (Total yield: 79%; KF/K_2PO_3F < 1:20). **^{19}F NMR** (377 MHz, D_2O, 10% D): δ -73.7 (d, $^1J_{PF}$ = 866 Hz), -120.0. **^{31}P NMR** (162 MHz, D_2O): δ 2.4, 0.9 (d, $^1J_{PF}$ = 866 Hz), -6.5 (d, $^2J_{P\text{-}P}$ = 20 Hz), -7.2, -21.0 (t, $^2J_{PP}$ = 20 Hz).

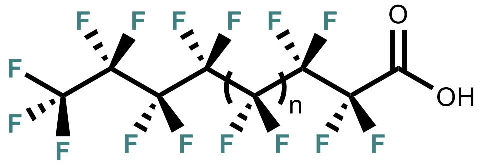

17, *n = 4, PFUnDA*

PFUnDA-mix^{KF} was prepared according to GP 2 using PFUnDA (1 equiv., 46 mg) and K_3PO_4 (1.25 equiv./F, 454 mg), and analysed by quantitative ^{19}F-NMR spectroscopy. 75% KF, 25% K_2PO_3F. (Total yield: quantitative; KF/K_2PO_3F = 3.0:1). **^{19}F NMR** (377 MHz, D_2O, 10% D): δ -73.7 (d, $^1J_{PF}$ = 866 Hz), -120.2. **^{31}P NMR** (162 MHz, D_2O): δ 2.6, 0.9 (d, $^1J_{PF}$ = 866 Hz), -5.6 (d, $^2J_{PP}$ = 20 Hz), -6.5, -20.5 (t, $^2J_{PP}$ = 20 Hz).

PFUnDA-mix^{PF} was prepared according to GP 3 using PFUnDA (1 equiv., 58 mg) and $K_4P_2O_7$ (0.625 equiv./F, 442 mg), and analysed by quantitative ^{19}F-NMR spectroscopy. 3% KF, 69% K_2PO_3F. (Total yield: 72%; KF/K_2PO_3F < 1:20). **^{19}F NMR** (377 MHz, D_2O, 10% D): δ -73.7 (d, $^1J_{PF}$ = 866 Hz), -120.0. **^{31}P NMR** (162 MHz, D_2O): δ 2.4, 0.9 (d, $^1J_{PF}$ = 866 Hz), -6.6 (d, $^2J_{PP}$ = 20 Hz), -7.2, -21.0 (m).

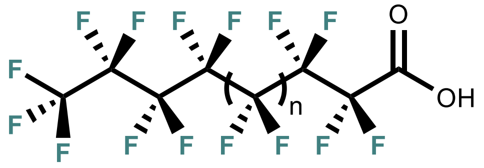

18, *n = 5, PFDoDA*

PFDoDA-mix^{KF} was prepared according to GP 2 using PFDoDA (1 equiv., 46 mg) and K_3PO_4 (1.25 equiv./F, 454 mg), and analysed by quantitative ^{19}F-NMR spectroscopy. 74% KF, 24% K_2PO_3F. (Total yield: 98%; KF/K_2PO_3F = 3.1:1). **^{19}F NMR** (377 MHz, D_2O, 10% D): δ -73.7 (d, $^1J_{PF}$ = 866 Hz), -120.0. **^{31}P NMR** (162 MHz, D_2O): δ 2.6, 0.9 (d, $^1J_{PF}$ = 866 Hz), -5.6 (d, $^2J_{PP}$ = 20 Hz), -6.5, -20.5 (t, $^2J_{PP}$ = 20 Hz).

PFDoDA-mix^{PF} was prepared according to GP 3 using PFDoDA (1 equiv., 57 mg) and $K_4P_2O_7$ (0.625 equiv./F, 443 mg), and analysed by quantitative ^{19}F-NMR spectroscopy. 4% KF, 84% K_2PO_3F. (Total yield: 88%; KF/K_2PO_3F < 1:20). **^{19}F NMR** (377 MHz, D_2O, 10% D): δ -73.7 (d, $^1J_{PF}$ = 866 Hz), -120.0. **^{31}P NMR** (162 MHz, D_2O): δ 2.2, 0.9 (d, $^1J_{PF}$ = 866 Hz), -7.4.

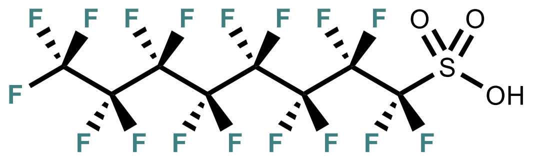

19, *PFOS*

PFOS-mix^{KF} was prepared according to GP 2 using PFOS (1 equiv., 32 mg) and K_3PO_4 (2 equiv./F, 468 mg) in triplicates and analysed by quantitative ^{19}F-NMR spectroscopy. Entry 1: 80% KF, 4% K_2PO_3F; entry 2: 74% KF, 4% K_2PO_3F; entry 3: 68% KF, 18% K_2PO_3F. (Average total yield: 83%; KF/K_2PO_3F = 8.5:1). **^{19}F NMR** (377 MHz, D_2O, 10% D): δ -73.7 (d, $^1J_{PF}$ = 866 Hz), -120.0. **^{31}P NMR** (162 MHz, D_2O): δ 3.6, 0.9 (d, $^1J_{PF}$ = 866 Hz), -6.4.

PFOS-mix^{PF} was prepared according to GP 3 with 6 h by using PFOS (1 equiv., 62 mg) and $K_4P_2O_7$ (0.625 equiv./F, 438 mg in triplicates and analysed by quantitative ^{19}F-NMR spectroscopy. Entry 1: 11% KF, 66% K_2PO_3F; entry 2: 3% KF, 41% K_2PO_3F; entry 3: 6% KF, 42% K_2PO_3F. (Average total yield: 56%; KF/K_2PO_3F = 1:7.4). **^{19}F NMR** (377 MHz, D_2O, 10% D): δ -73.7 (d, $^1J_{PF}$ = 866 Hz), -120.0. **^{31}P NMR** (162 MHz, D_2O): δ 2.0, 0.9 (d, $^1J_{PF}$ = 866 Hz).

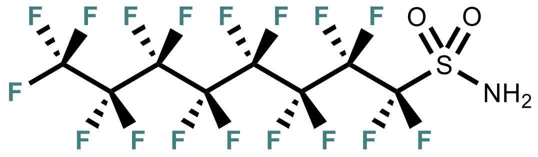

20, *PFOSA*

PFOSA-mix^{KF} was prepared according to GP 2 using PFOSA (1 equiv., 50 mg) and K_3PO_4 (1.25 equiv./F, 450 mg) in triplicates and analysed by quantitative ^{19}F-NMR spectroscopy. Entry 1: 77% KF, 21% K_2PO_3F; entry 2: 78% KF, 25% K_2PO_3F; entry 3: 76% KF, 24% K_2PO_3F. (Average total yield: quantitative; KF/K_2PO_3F = 3.3:1). **^{19}F NMR** (377 MHz, D_2O, 10% D): δ -73.7 (d, $^1J_{PF}$ = 866 Hz), -120.0. **^{31}P NMR** (162 MHz, D_2O): δ 2.6, 0.9 (d, $^1J_{PF}$ = 866 Hz), -5.6 (d, $^2J_{PP}$ = 20 Hz), -6.4.

PFOSA-mix^{PF} was prepared according to GP 3 using PFOSA (1 equiv., 62 mg) and $K_4P_2O_7$ (0.625 equiv./F, 438 mg) in triplicates and analysed by quantitative ^{19}F-NMR spectroscopy. Entry 1: 6% KF, 73% K_2PO_3F; entry 2: 3% KF, 85% K_2PO_3F; entry 3: 3% KF, 75% K_2PO_3F. (Average total yield: 82%; KF/K_2PO_3F = 1:20). **^{19}F NMR** (377 MHz, D_2O, 10% D): δ -73.7 (d, $^1J_{PF}$ = 866 Hz), -120.0. **^{31}P NMR** (162 MHz, D_2O): δ 1.9, 0.9 (d, $^1J_{PF}$ = 866 Hz), -7.5.

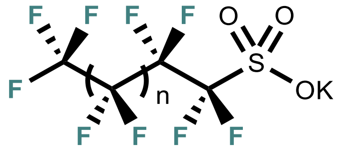

21, n = 1, *KPFBS*

KPFBS-mix^{KF} was prepared according to GP 2 using KPFBS (1 equiv., 41 mg) and K_3PO_4 (2 equiv./F, 459 mg) in triplicates and analysed by quantitative ^{19}F-NMR spectroscopy. Entry 1: 73% KF, 3% K_2PO_3F; entry 2: 74% KF, 3% K_2PO_3F; entry 3: 74% KF, 3% K_2PO_3F. (Average total yield: 77%; KF/K_2PO_3F > 20:1). **^{19}F NMR** (377 MHz, D_2O, 10% D): δ -73.7 (d, $^1J_{PF}$ = 866 Hz), -120.0. **^{31}P NMR** (162 MHz, D_2O): δ 3.8, 0.9 (d, $^1J_{PF}$ = 866 Hz), -6.4.

KPFBS-mix^{PF} was prepared according to GP 3 with 6 h milling by using KPFBS (1 equiv., 77 mg) and $K_4P_2O_7$ (0.625 equiv./F, 423 mg) in triplicates and analysed by quantitative ^{19}F-NMR spectroscopy. Entry 1: 4% KF, 82% K_2PO_3F; entry 2: 8% KF, 65% K_2PO_3F; entry 3: 4% KF, 75% K_2PO_3F. (Average total yield: 79%; KF/K_2PO_3F = 1:14). **^{19}F NMR** (377 MHz, D_2O, 10% D): δ -73.7 (d, $^1J_{PF}$ = 866 Hz), -120.0. **^{31}P NMR** (162 MHz, D_2O): δ 2.3, 0.9 (d, $^1J_{PF}$ = 866 Hz).

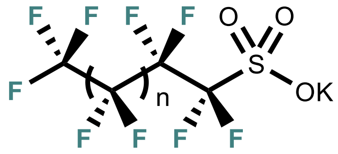

22, n = 3, *KPFHxS*

KPFHxS-mix^{KF} was prepared according to GP 2 with 6 h milling using KPFHxS (1 equiv., 56 mg) and K_3PO_4 (1.25 equiv./F, 444 mg) in triplicates and analysed by quantitative ^{19}F-NMR spectroscopy. Entry 1: 77% KF, 7% K_2PO_3F; entry 2: 83% KF, 5% K_2PO_3F; entry 3: 82% KF, 5% K_2PO_3F. (Average total yield: 86%; KF/K_2PO_3F = 14:1). **^{19}F NMR** (377 MHz, D_2O, 10% D): δ -73.7 (d, $^1J_{PF}$ = 866 Hz), -120.0. **^{31}P NMR** (162 MHz, D_2O): δ 2.7, 0.9 (d, $^1J_{PF}$ = 866 Hz), -5.6 (d, $^2J_{PP}$ = 20 Hz), -6.4.

KPFHxS-mix^{PF} was prepared according to GP 3 with 6 h milling using KPFHxS (1 equiv., 70 mg) and $K_4P_2O_7$ (0.625 equiv./F, 430 mg) in triplicates and analysed by quantitative ^{19}F-NMR spectroscopy. Entry 1: 6% KF, 89% K_2PO_3F; entry 2: 17% KF, 61% K_2PO_3F; entry 3: 8% KF, 82% K_2PO_3F. (Average total yield: 88%; KF/K_2PO_3F = 1:7.5). **^{19}F NMR** (377 MHz, D_2O, 10% D): δ -73.7 (d, $^1J_{PF}$ = 866 Hz), -120.0. **^{31}P NMR** (162 MHz, D_2O): δ 2.2, 0.9 (d, $^1J_{PF}$ = 866 Hz).

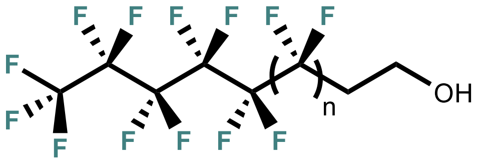

23, n = 1, *6:2 FTOH*

6:2 FTOH-mix^{KF} was prepared according to GP 2 using 6:2 FTOH (1 equiv., 48 mg) and K_3PO_4 (1.25 equiv./F, 452 mg), and analysed by quantitative ^{19}F-NMR spectroscopy. 59% KF, 41% K_2PO_3F. (Total yield: quantitative; KF/K_2PO_3F = 1.4:1). **^{19}F NMR** (377 MHz, D_2O, 10% D): δ -73.7 (d, $^1J_{PF}$ = 866 Hz), -120.7. **^{31}P NMR** (162 MHz, D_2O): δ 2.6, 0.9 (d, $^1J_{PF}$ = 866 Hz), -6.5.

6:2 FTOH-mix^{PF} was prepared according to GP 3 using 6:2 FTOH (1 equiv., 60 mg) and $K_4P_2O_7$ (0.625 equiv./F, 440 mg), and analysed by quantitative ^{19}F-NMR spectroscopy. 19% KF, 81% K_2PO_3F. (Total yield: quantitative; KF/K_2PO_3F = 1:4.3). **^{19}F NMR** (377 MHz, D_2O, 10% D): δ -73.7 (d, $^1J_{PF}$ = 866 Hz), -120.4. **^{31}P NMR** (162 MHz, D_2O): δ 0.8 (d, $^1J_{PF}$ = 866 Hz), 0.5.

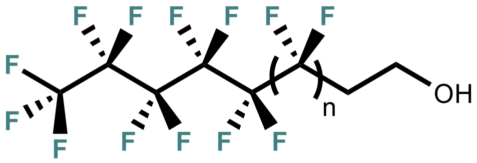

24, n = 3, *8:2 FTOH*

8:2 FTOH-mix^{KF} was prepared according to GP 2 using 8:2 FTOH (1 equiv., 47 mg) and K_3PO_4 (1.25 equiv./F, 453 mg) in triplicates and analysed by quantitative ^{19}F-NMR spectroscopy. Entry 1: 78% KF, 15% K_2PO_3F; entry 2: 82% KF, 22% K_2PO_3F; entry 3: 82% KF, 17% K_2PO_3F. (Average total yield: 99%; KF/K_2PO_3F = 4.5:1). **^{19}F NMR** (377 MHz, D_2O, 10% D): δ -73.7 (d, $^1J_{PF}$ = 866 Hz), -120.0. **^{31}P NMR** (162 MHz, D_2O): δ 2.6, 0.9 (d, $^1J_{PF}$ = 866 Hz), -5.5 (d, $^2J_{PP}$ = 20 Hz), -6.4, -20.4 (m).

8:2 FTOH-mix^{PF} was prepared according to GP 3 using 8:2 FTOH (1 equiv., 58 mg) and $K_4P_2O_7$ (0.625 equiv./F, 442 mg) in triplicates and analysed by quantitative ^{19}F-NMR spectroscopy. Entry 1: 17% KF, 71% K_2PO_3F; entry 2: 15% KF, 80% K_2PO_3F; entry 3: 15% KF, 87% K_2PO_3F. (Average total yield: 95%; KF/K_2PO_3F = 1:5.0). **^{19}F NMR** (377 MHz, D_2O, 10% D): δ -73.7 (d, $^1J_{PF}$ = 866 Hz), -120.0. **^{31}P NMR** (162 MHz, D_2O): δ 2.1, 0.9 (d, $^1J_{PF}$ = 866 Hz).

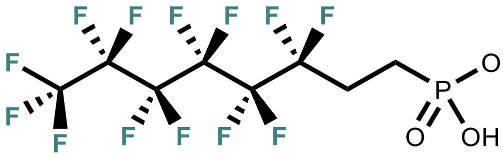

25, *6:2 FTPA*

6:2 FTPA-mix^{KF} was prepared according to GP 2 using 6:2 FTPA (1 equiv., 55 mg) and K_3PO_4 (1.25 equiv./F, 445 mg) in triplicates and analysed by quantitative ^{19}F-NMR spectroscopy. Entry 1: 65% KF, 29% K_2PO_3F; entry 2: 70% KF, 29% K_2PO_3F; entry 3: 67% KF, 31% K_2PO_3F. (Average total yield: 97%; KF/K_2PO_3F = 2.2:1). **^{19}F NMR** (377 MHz, D_2O, 10% D): δ -73.7 (d, $^1J_{PF}$ = 866 Hz), -122.0. **^{31}P NMR** (162 MHz, D_2O): δ 2.6, 0.9 (d, $^1J_{PF}$ = 866 Hz), -6.8.

6:2 FTPA-mix^{PF} was prepared according to GP 3 using 6:2 FTPA (1 equiv., 69 mg) and $K_4P_2O_7$ (0.625 equiv./F, 431 mg) in triplicates and analysed by quantitative ^{19}F-NMR spectroscopy. Entry 1: 0% KF, 82% K_2PO_3F; entry 2: 9% KF, 87% K_2PO_3F; entry 3: 1% KF, 83% K_2PO_3F. (Average total yield: 87%; KF/K_2PO_3F < 1:20). **^{19}F NMR** (377 MHz, D_2O, 10% D): δ -73.7 (d, $^1J_{PF}$ = 866 Hz). **^{31}P NMR** (162 MHz, D_2O): δ 2.2, 0.9 (d, $^1J_{PF}$ = 866 Hz), -7.6.

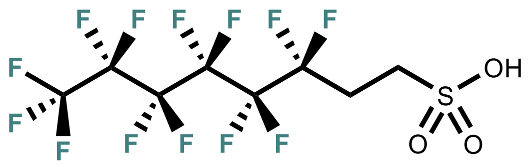

26, *6:2 FTSA*

6:2 FTSA-mix^{KF} was prepared according to GP 2 using 6:2 FTSA (1 equiv., 55 mg) and K_3PO_4 (1.25 equiv./F, 445 mg) in triplicates and analysed by quantitative ^{19}F-NMR spectroscopy. Entry 1: 74% KF, 26% K_2PO_3F; entry 2: 78% KF, 25% K_2PO_3F; entry 3: 80% KF, 17% K_2PO_3F. (Average total yield: quantitative; KF/K_2PO_3F = 3.4:1). **^{19}F NMR** (377 MHz, D_2O, 10% D): δ -73.7 (d, $^1J_{PF}$ = 866 Hz), -121.7. **^{31}P NMR** (162 MHz, D_2O): δ 2.6, 0.9 (d, $^1J_{PF}$ = 866 Hz), -6.6.

6:2 FTSA-mix^{PF} was prepared according to GP 3 using 6:2 FA (1 equiv., 69 mg) and $K_4P_2O_7$ (0.625 equiv./F, 431 mg) and analysed by quantitative ^{19}F-NMR spectroscopy. 7% KF, 72% K_2PO_3F. (Total yield: 79%; KF/K_2PO_3F = 1:10). **^{19}F NMR** (377 MHz, D_2O, 10% D): δ -73.7 (d, $^1J_{PF}$ = 866 Hz). **^{31}P NMR** (162 MHz, D_2O): δ 2.3, 0.9 (d, $^1J_{PF}$ = 866 Hz).

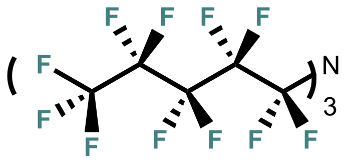

27, *FC-70*

FC-70-mix^{KF} was prepared according to GP 2 using FC-70 (1 equiv., 43 mg) and K_3PO_4 (1.25 equiv./F, 457 mg) in triplicates and analysed by quantitative ^{19}F-NMR spectroscopy. Entry 1: 58% KF, 3% K_2PO_3F; entry 2: 61% KF, 3% K_2PO_3F; entry 3: 60% KF, 3% K_2PO_3F. (Average total yield: 63%; KF/K_2PO_3F = 20:1). **^{19}F NMR** (377 MHz, D_2O, 10% D): δ -73.7 (d, $^1J_{PF}$ = 866 Hz), -120.0. **^{31}P NMR** (162 MHz, D_2O): δ 3.7, 0.9 (d, $^1J_{PF}$ = 866 Hz), -5.5 (d, $^2J_{PP}$ = 20 Hz), -6.3.

FC-70-mix^{PF} was prepared according to GP 3 using FC-70 (1 equiv., 54 mg) and $K_4P_2O_7$ (0.625 equiv./F, 446 mg) in duplicates and analysed by quantitative ^{19}F-NMR spectroscopy. Entry 1: 4% KF, 27% K_2PO_3F; entry 2: 6% KF, 23%; entry 3: 4% KF, 28%. (Average total yield: 31%; KF/K_2PO_3F = 1:5.6). **^{19}F NMR** (377 MHz, D_2O, 10% D): δ -73.7 (d, $^1J_{PF}$ = 866 Hz), -120.0. **^{31}P NMR** (162 MHz, D_2O): δ 2.5, 0.9 (d, $^1J_{PF}$ = 866 Hz), -5.8 (d, $^2J_{PP}$ = 20 Hz), -6.6, -20.5 (t, $^2J_{PP}$ = 20 Hz).

11. Thermochemistry

Thermochemistry

Ideal reaction equations were deduced from the profile of product mixture assuming only one major pathway being present Standard enthalpy change of reaction was calculated from the standard enthalpy of formation of the corresponding substances in the reaction equation [68-75]. Due to the lack of experimental enthalpy data for K_2PO_3F, simulated data from The Materials Project and The Open Quantum Materials Database were used and hence resulted in a predicted range for the enthalpy change.

Table S18. Standard enthalpy of formation. cr = crystalline solids, g = gaseous, s = solid

	ΔH^o_f [kcal·mol^{-1}]
PTFE (s)	-197.8 ± 0.4 kcal/(gfw C_2F_4) [68]
K_3PO_4 (cr)	-466.1 [69]
$K_4P_2O_7$ (cr)	-773.3 [70]
KF (cr)	-135.6 [69], -135.9 [71]
K_2PO_3F (cr)	-446.3 (predicted, MP) [72,73]<br>-432.8 (predicted, OQMD) [74,75]
K_2CO_3 (cr)	-274.9 (cr) [71], -275.1 (cr) [69]
CO_2 (g)	-94.1 [69,71]
C (graphite, cr)	0

$[C_2F_4]_n + 6n\ K_3PO_4 \longrightarrow n\ C + n\ K_2CO_3 + 4n\ KF + 3n\ K_4P_2O_7$ **$\Delta H°$ = -143.5 ± 1.1 kcal·mol^{-1}**

$[C_2F_4]_n + 2n\ K_4P_2O_7 \longrightarrow n\ C + n\ CO_2 + 4n\ K_2PO_3F$ **$\Delta H°$ = -107.9 ± 27.4 kcal·mol^{-1}**

$KF + K_4P_2O_7 \longrightarrow K_3PO_4 + K_2PO_3F$ **$\Delta H°$ = 3.4 ± 6.9 kcal·mol^{-1}**

12. DFT calculations

Computational Methods

Geometry optimisation, vibrational frequency and single point energy calculations were performed using Gaussian 16, revision C.01 [37]. Geometry optimisation, frequency and single point energy calculations were performed at the *ω*B97xD/6-311++G(2d,2p) level of theory in gas-phase [76]. Ground state geometries were identified by the absence of imaginary frequency vibrational modes. Transition state geometries (TSs) were identified by the presence of a single imaginary frequency vibrational mode. 3D structures of molecules were generated by CYLview [77]. All bond lengths are in Angstroms (Å). Quasi-harmonic corrections were applied to generate thermochemical data using GoodVibes, with a frequency cutoff value of 100 cm^{-1} [78].

Nucleophilicity index *N*

Vertical ionization potential (IP) and vertical electron affinity (EA) were determined, using the geometry of the neutral system [79].

$$IP = E(\mathrm{M}^+) - E(\mathrm{M})$$

$$EA = E(\mathrm{M}) - E(\mathrm{M}^-)$$

Chemical potential (μ) and chemical hardness (η) were defined by Parr and co-workers as following [80,40]:

$$\mu = -\frac{IP + EA}{2}$$

$$\eta = IP - EA$$

Nucleophilicity indices *N* of activators were computed with four different available equations from literature and plotted against the experimental value of released fluoride as (sum of F^- and PO_3F^{2-}) after mechanochemical degradation.

Method 1 [40,41]:

$$N = \frac{1}{\omega} \quad where \quad \omega = \frac{\mu^2}{2\eta}$$

Method 2 [81]:

$$N = \frac{1}{\omega^-} \quad where \;\; \omega^- = \frac{I^2}{2(I - A)}$$

Method 3 [81]:

$$N = \frac{1}{\omega^-} \quad where \;\; \omega^- = \frac{(3I + A)^2}{16(I - A)}$$

Method 4 [38]:

$$N = E_{HOMO(Nu)}(eV) - E_{HOMO(TCE)}(eV)$$

where tetracyanoethylene (TCE) is used as a reference molecule.

Table S19. IP = ionization potential (Hartree), EA = electron affinity (Hartree), HOMO energy was in eV. N_1, N_2, N_3, N_4 represented nucleophilicity index computed by method 1–4 respectively.

Activator	IP	EA	Homo	N_1	N_2	N_3	N_4
K_3PO_4	0.22563	-0.00149	-0.21772	36.2	8.92	7.97	0.204
$K_4P_2O_7$	0.27764	0.00375	-0.27239	27.7	7.11	6.26	0.150
K_2CO_3	0.25011	0.00515	-0.23376	30.1	7.83	6.87	0.188
Na_3PO_4	0.24528	0.00183	-0.23359	31.9	8.09	7.16	0.188
$K_2C_2O_4$	0.27315	0.00134	-0.26128	28.9	7.29	6.46	0.161
K_2HPO_4	0.29094	0.00565	-0.27233	25.9	6.74	5.92	0.378
K_2PO_3F	0.29611	0.00777	-0.28249	25.0	6.58	5.75	0.139
$(KPO_3)_3$	0.33873	0.01100	-0.33224	21.4	5.71	4.97	0.090
Li_3PO_4	0.31145	0.00443	-0.30188	24.6	6.33	5.57	0.120
KH_2PO_4	0.34887	0.01109	-0.33197	20.9	5.55	4.83	0.090
HCO_2K	0.33781	0.01137	-0.31154	21.4	5.72	4.97	0.110
KOH	0.28346	0.00903	-0.25490	25.7	6.83	5.95	0.167
$K_3PO_4 \cdot H_2O$	0.23990	0.00132	-0.23364	32.8	8.29	7.34	0.188
$K_3PO_4 \cdot 2H_2O$	0.25798	0.00377	-0.25326	29.7	7.64	6.73	0.169
$K_3PO_4 \cdot 3H_2O$	0.26780	0.00088	-0.26431	29.6	7.44	6.60	0.158
$KOH \cdot H_2O$	0.31144	0.00589	-0.28413	24.3	6.30	5.53	0.138
$KOH \cdot 2H_2O$	0.33457	0.00196	-0.30997	23.5	5.94	5.26	0.112
$KOH \cdot 3H_2O$	0.34577	0.00021	-0.32796	23.1	5.78	5.14	0.094

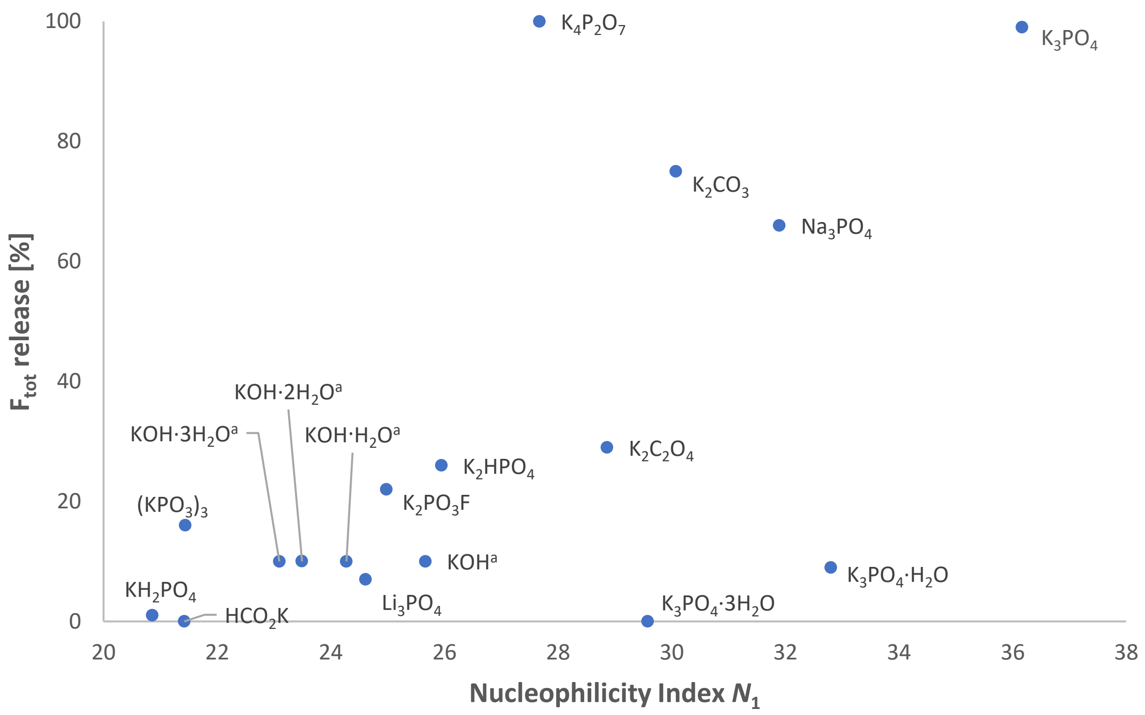

Figure S70. Nucleophilicity index N_1 calculated from method 1 plotted against the experimental value of recovered fluoride as (KF+P-F) after mechanochemical degradation. [a] Nucleophilicity indices of KOH hydrate clusters (KOH·H_2O, KOH·2H_2O, KOH·3H_2O) are compared to experimental values of untreated KOH pellets, while KOH is compared to pre-dried KOH (heated above melting point under high vacuum for 20 min).

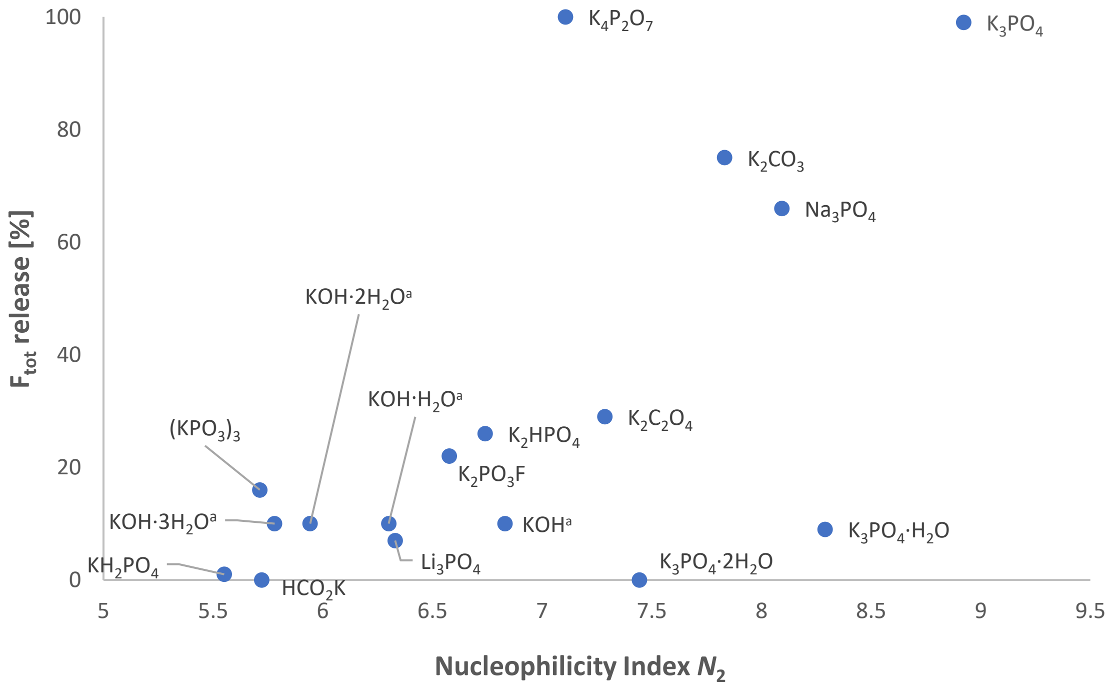

Figure S71. Nucleophilicity index N_2 calculated from method 2 plotted against the experimental value of recovered fluoride as (KF+P-F) after mechanochemical degradation. [a] Nucleophilicity indices of KOH hydrate clusters (KOH·H_2O, KOH·2H_2O, KOH·3H_2O) are compared to experimental values of untreated KOH pellets, while KOH is compared to pre-dried KOH (heated above melting point under high vacuum for 20 min).

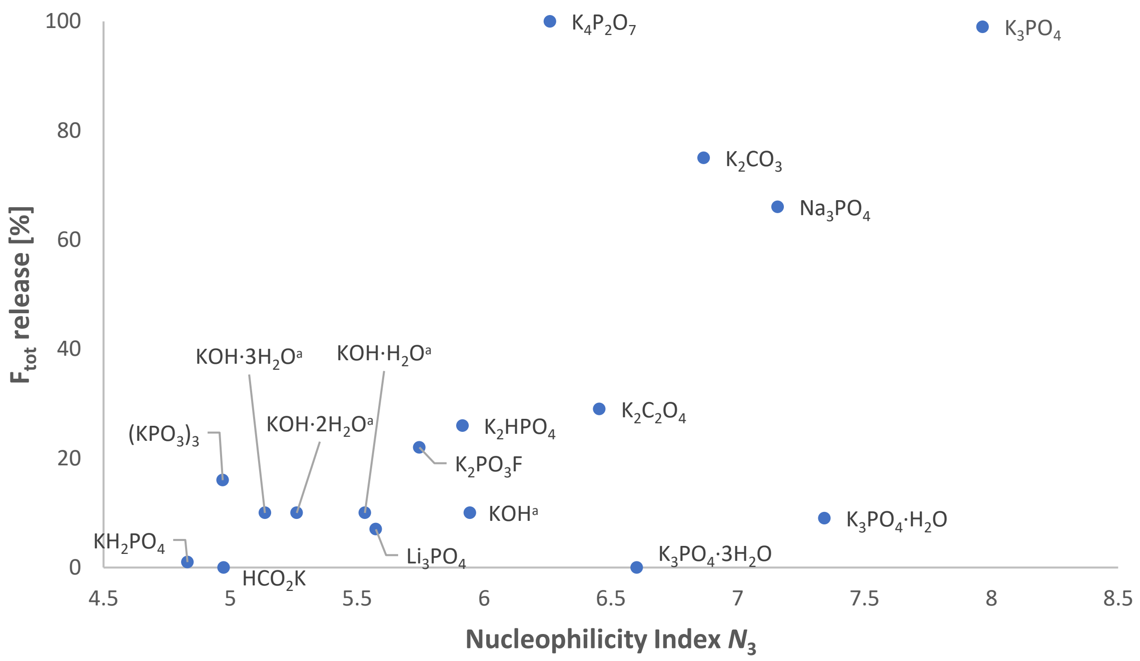

Figure S72. Nucleophilicity index N_3 calculated from method 3 plotted against the experimental value of recovered fluoride as (KF+P-F) after mechanochemical degradation. [a] Nucleophilicity indices of KOH hydrate clusters (KOH·H_2O, KOH·2H_2O, KOH·3H_2O) are compared to experimental values of untreated KOH pellets, while KOH is compared to pre-dried KOH (heated above melting point under high vacuum for 20 min).

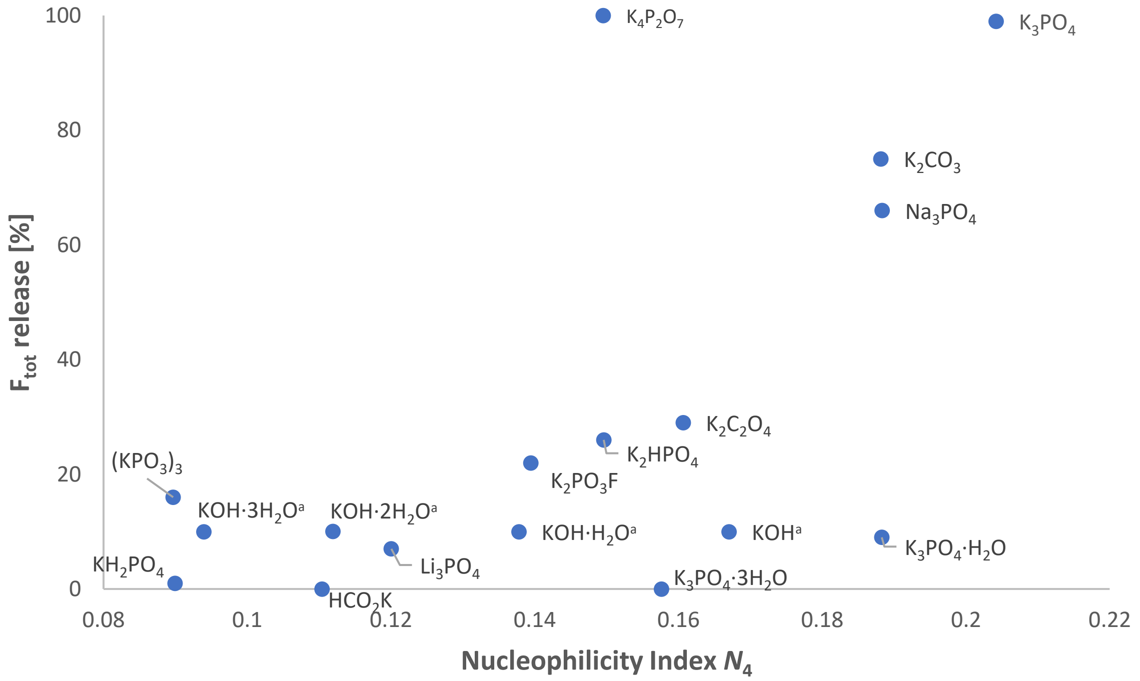

Figure S73. Nucleophilicity index N_4 calculated from method 4 plotted against the experimental value of recovered fluoride as (KF+P-F) after mechanochemical degradation. [a] Nucleophilicity indices of KOH hydrate clusters (KOH·H_2O, KOH·2H_2O, KOH·3H_2O) are compared to experimental values of untreated KOH pellets, while KOH is compared to pre-dried KOH (heated above melting point under high vacuum for 20 min).

Transition state energy

Perfluorobutane was used as the model electrophile for computing the transition state of nucleophilic substitution. Different modes of nucleophilic attack by K_3PO_4 and $K_4P_2O_7$ were compared, and S_N2 attack at the internal carbon

resulted in lowest energy transition state. Activators listed in Table 16 were assessed under the same mode and $\Delta\Delta G^{\ddagger}$ was plotted against F_{tot} release [%]from experimental data.

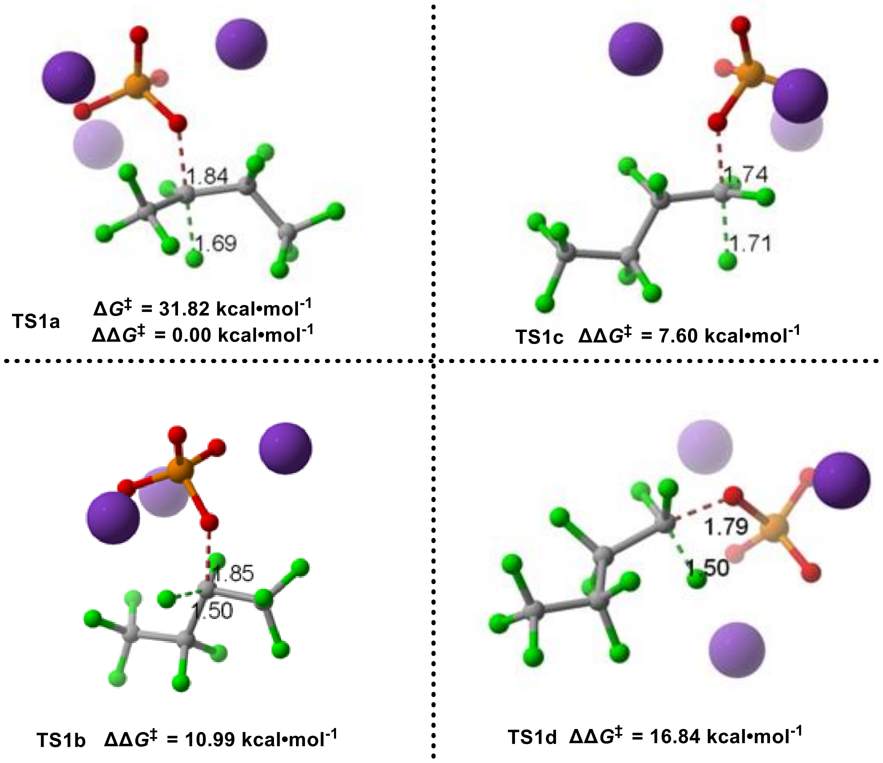

Figure S74. Transition states of nucleophilic substitution between K_3PO_4 and C_4F_{10}. S_N2 attack at the internal carbon resulted in the lowest transition state energy.

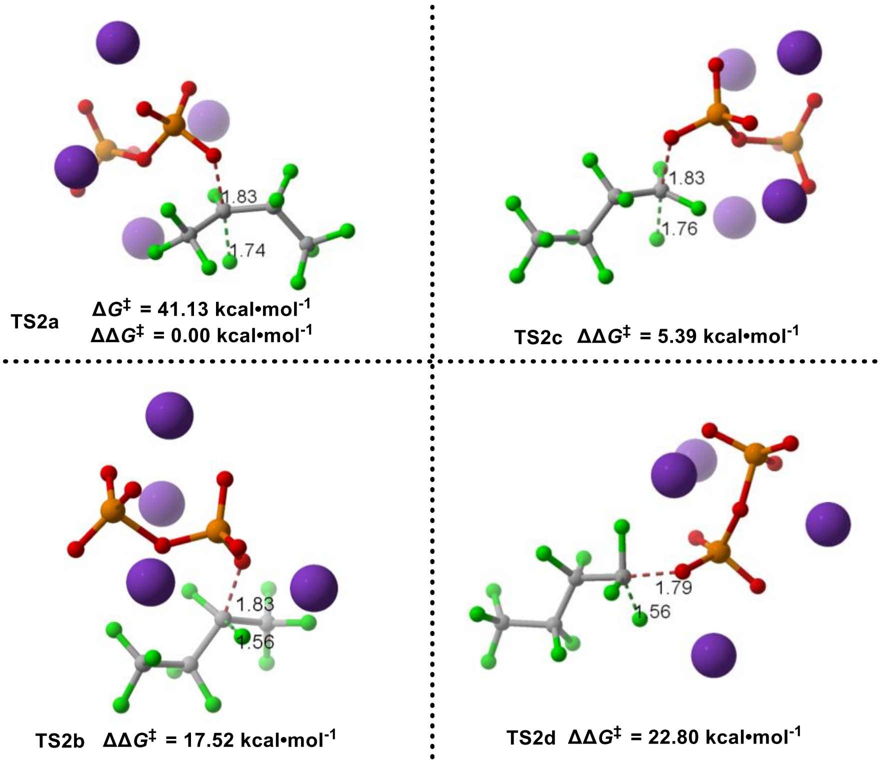

Figure S75. Transition states of nucleophilic substitution between $K_4P_2O_7$ and C_4F_{10}. S_N2 attack at the internal carbon resulted in the lowest transition state energy.

Table S20. Relative transition state energy differences (**ΔΔ*G*‡**) of nucleophilic substitution of perfluorobutane with different activators at internal carbon.

**Entry**	**Activator**	**ΔΔ*G*‡** kcal/mol
TS1a	K_3PO_4	0
TS2a	$K_4P_2O_7$	9.31
TS3	K_2CO_3	10.38
TS4	Na_3PO_4	2.36
TS5	$K_2C_2O_4$	26.69
TS6	K_2HPO_4	17.12
TS7	K_2PO_3F	18.39
TS8	$(KPO_3)_3$	31.21
TS9	Li_3PO_4	19.41
TS10	KH_2PO_4	31.29
TS11	HCO_2K	36.50
TS12	KOH	2.44
TS13	$K_3PO_4·H_2O$	5.52
TS14	$K_3PO_4·2H_2O$	8.67
TS15	$K_3PO_4·3H_2O$	12.84
TS16	$KOH·H_2O$	11.32
TS17	$KOH·2H_2O$	18.77

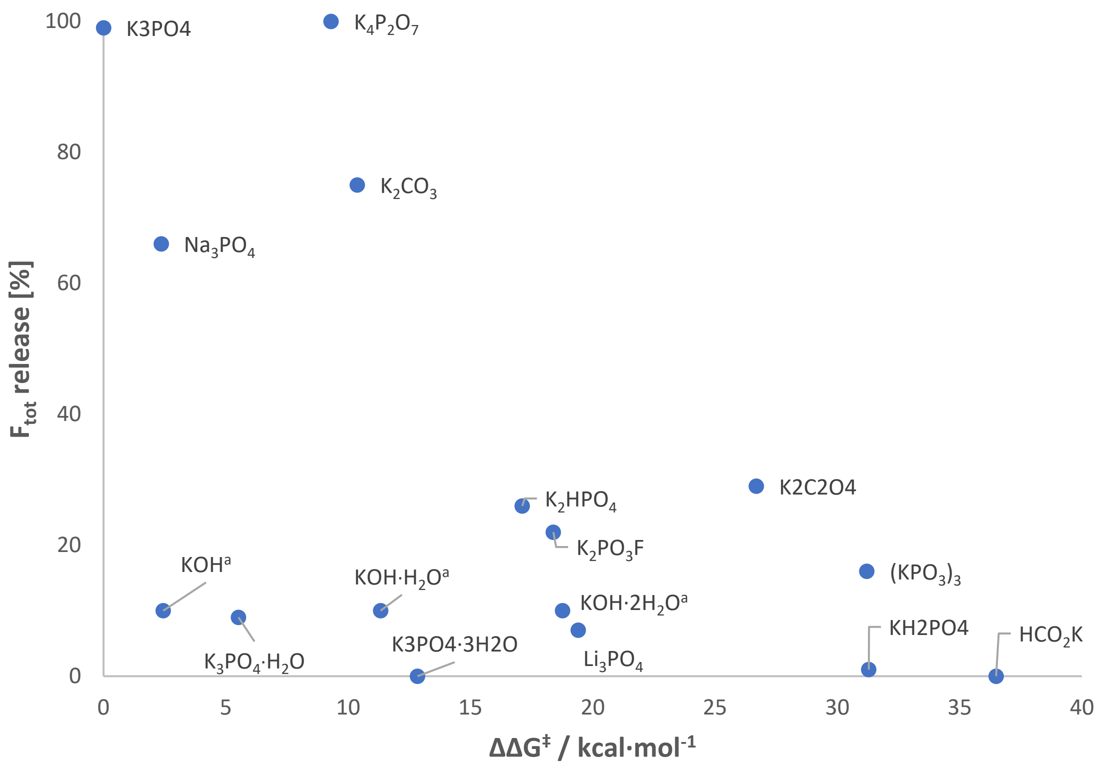

Figure S76. Relative transition state energy difference (**Δ*ΔG*‡**) of nucleophilic substitution of perfluorobutane with different activators at internal carbon plotted against F_{tot} release [%] from experimental data. [a] Nucleophilicity indices of KOH hydrate clusters (KOH·H_2O, KOH·$2H_2O$) are compared to experimental values of untreated KOH pellets, while KOH is compared to pre-dried KOH (heated above melting point under high vacuum for 20 min).

Solvent effect

Activation barrier of nucleophilic attack by K_3PO_4 and $K_4P_2O_7$ at the internal carbon of C_4F_{10} was evaluated in various solvents to estimate the possible change of kinetic feasibility in the reaction media under mechanochemical condition. Structures have been optimised under the SMD solvation model in the solvents listed in Table S21.

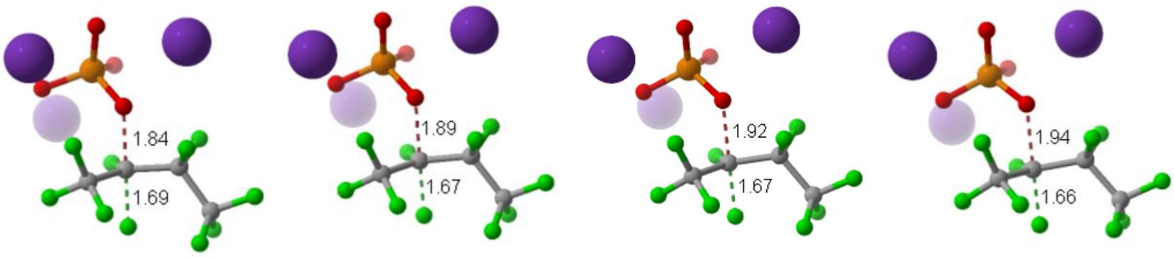

TS1a (gas-phase)	TS1e (toluene)	TS1f (dichloromethane)	TS1g (acetonitrile)
ε: 1.00	ε: 2.37	ε: 8.93	ε: 35.69
ΔΔ$G^‡$ = 0.00 kcal•mol^{-1}	ΔΔ$G^‡$ = 0.55 kcal•mol^{-1}	ΔΔ$G^‡$ = 4.24 kcal•mol^{-1}	ΔΔ$G^‡$ = 4.94 kcal•mol^{-1}

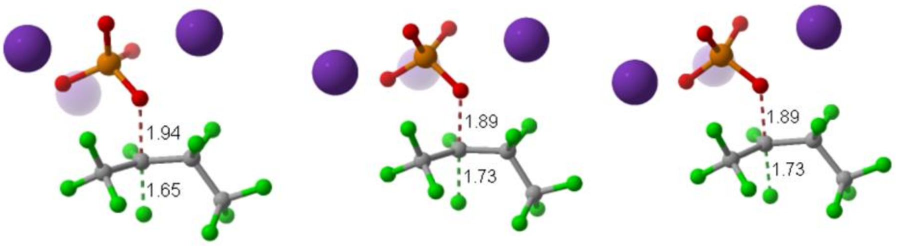

TS1h (dimethyl sulfoxide)	TS1i (water)	TS1j (formamide)
ε: 46.83	ε: 78.36	ε: 108.94
ΔΔ$G^‡$ = 2.79 kcal•mol^{-1}	ΔΔ$G^‡$ = 14.48 kcal•mol^{-1}	ΔΔ$G^‡$ = 13.71 kcal•mol^{-1}

Figure S77. Transition states of nucleophilic substitution between K_3PO_4 and C_4F_{10} at internal carbon in various solvents.

Table S21. Relative transition state energy difference (**ΔΔ*G*‡**) and key distances of nucleophilic substitution between K_3PO_4 and perfluorobutane at internal carbon in various solvents.

Entry	Solvent	Dielectric constant ε	ΔΔG‡	O---C / Å	C---F / Å
TS1a	Gas-phase	1.00	0.00	1.84	1.69
TS1e	Toluene	2.37	0.55	1.89	1.67
TS1f	Dichloromethane	8.93	4.24	1.92	1.67
TS1g	Acetonitrile	35.69	4.94	1.94	1.66
TS1h	Dimethylsulfoxide	46.83	2.79	1.94	1.65
TS1i	Water	78.36	14.48	1.89	1.73
TS1j	Formamide	108.94	13.71	1.89	1.73

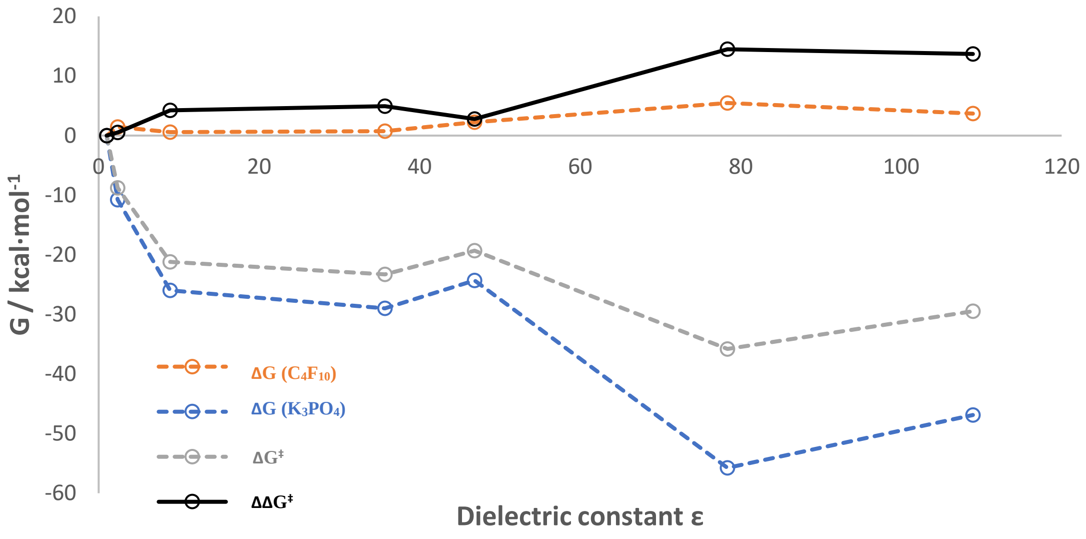

Figure S78. Relative energy difference (orange: **Δ*G*** (C_4F_{10}), blue: **Δ*G*** (K_3PO_4), grey: **Δ*G*‡, ** black: **ΔΔ*G*‡)** of nucleophilic substitution of perfluorobutane with potassium phosphate at internal carbon in different solvent.

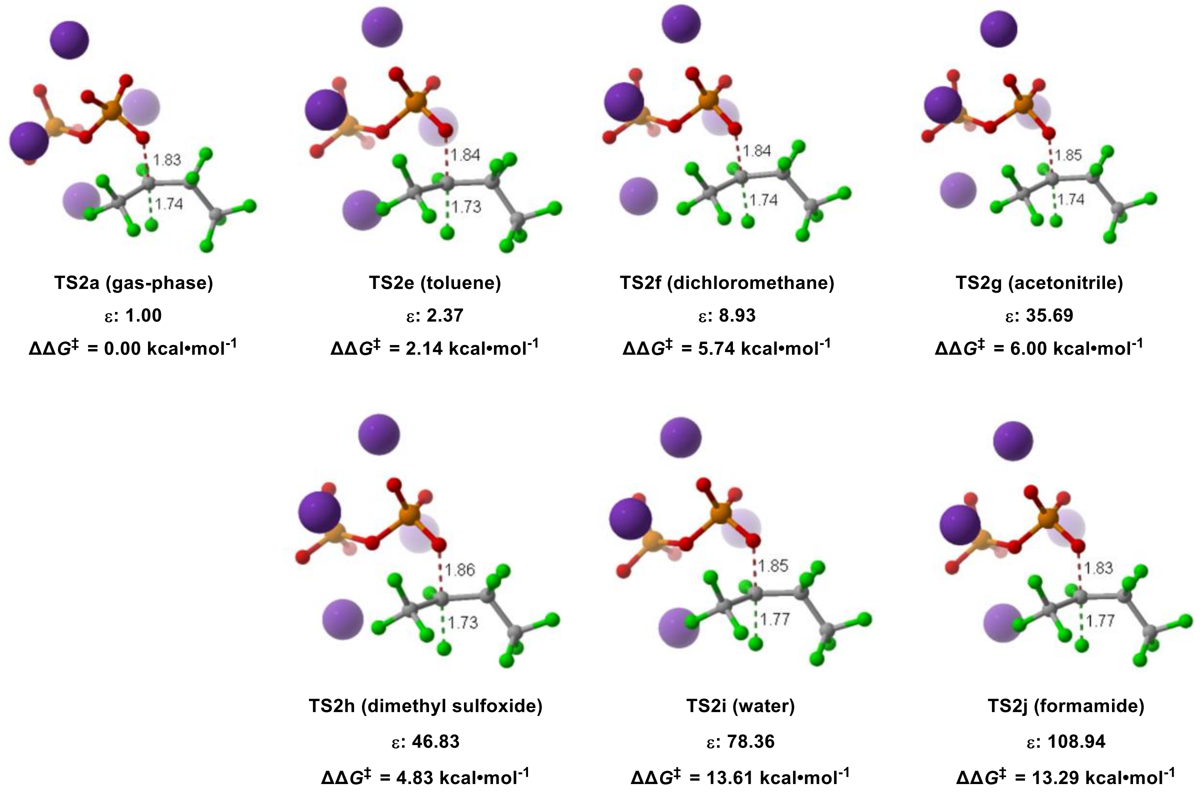

Figure S79. Transition states of nucleophilic substitution between $K_4P_2O_7$ and C_4F_{10} at internal carbon in various solvents.

Table S22. Relative transition state energy difference (**ΔΔ*G*‡**) and key distances of nucleophilic substitution between $K_4P_2O_7$ and perfluorobutane at internal carbon in various solvents.

Entry	Solvent	Dielectric constant ε	ΔΔG‡	O---C / Å	C---F / Å
TS2a	Gas-phase	1.00	0.00	1.83	1.74
TS2e	Toluene	2.37	2.14	1.84	1.73
TS2f	Dichloromethane	8.93	5.74	1.84	1.74
TS2g	Acetonitrile	35.69	6.00	1.85	1.74
TS2h	Dimethylsulfoxide	46.83	4.83	1.86	1.73
TS2i	Water	78.36	13.61	1.85	1.77
TS2j	Formamide	108.94	13.29	1.83	1.77

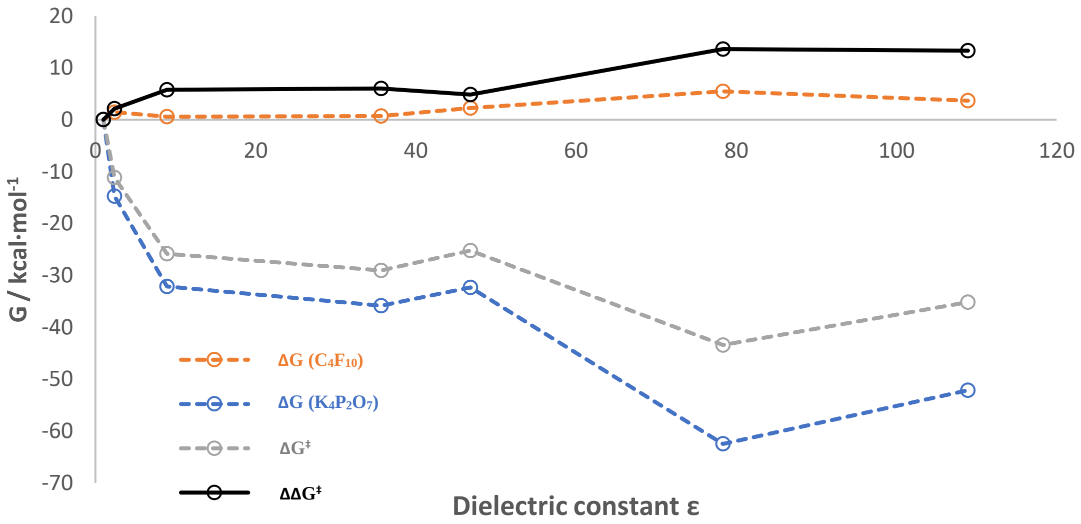

Figure S80. Relative energy difference (orange: **Δ*G*** (C_4F_{10}), blue: **Δ*G*** ($K_4P_2O_7$), grey: **Δ*G*‡**, black: **ΔΔ*G*‡)** of nucleophilic substitution of perfluorobutane with potassium phosphate at internal carbon in different solvent.

Model reaction with two units of K_3PO_4

Activation barrier of the nucleophilic substitution are both higher internally and terminally with an additional unit of K_3PO_4 in the model system (7.40 and 6.76 kcal/mol). Energy decomposition at the transition state structure revealed that the origin of such energy increase is due to the higher distortion energy in K_3PO_4 (internal: 25.52 vs. 7.20 kcal/mol; terminal: 26.87 vs. 7.43 kcal/mol).

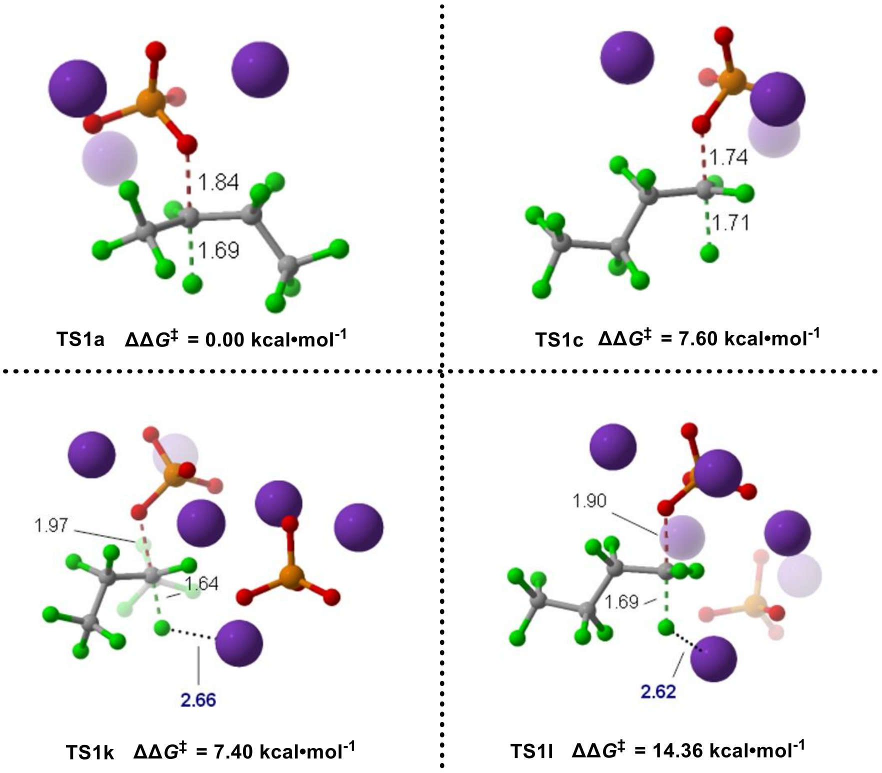

Figure S81. Transition states of nucleophilic substitution between one or two units of K_3PO_4 and C_4F_{10}.

Table S23. Distortion-interaction analysis at the transition states of the nucleophilic substitution between K_3PO_4 and perfluorobutane.

Entry	Attack position	K_3PO_4 units	$\Delta E^‡$ (total) / kcal·mol^{-1}	$\Delta E^‡_{C4H10}$ (distortion) / kcal·mol^{-1}	$\Delta E^‡_{K3PO4}$ (distortion) / kcal·mol^{-1}	$\Delta E^‡$ (interaction) / kcal·mol^{-1}
TS1a	Internal	1	16.93	61.89	7.20	-52.17
TS1k	Internal	2	25.03	49.27	25.52	-49.76
TS1c	Terminal	1	25.23	75.29	7.43	-57.50
TS1l	Terminal	2	32.30	60.52	26.87	-55.08

XYZ coordinates (All structures were optimised in gas phase unless specified in brackets.)

K_3PO_4

P	0.03727700	0.00093400	0.00277500
O	0.92615300	-1.27588100	0.00534500
K	-1.52832600	-2.27259100	-0.00226700
O	0.92192600	1.28139500	0.00343700
K	3.02321600	0.00441500	-0.00187300
O	-0.91029900	-0.00038600	1.23361100
K	-1.53914600	2.26536800	-0.00178200
O	-0.90256800	-0.00021200	-1.23353200

K_3PO_4 (toluene)

P	-0.05023700	0.00011600	0.00054900
O	-0.93280600	1.27900100	0.00064400
K	1.57582600	2.27433400	-0.00040500
O	-0.93329400	-1.27843500	0.00032200
K	-3.07731700	0.00065400	-0.00041900
O	0.89373200	-0.00025600	1.23733100
K	1.57429700	-2.27524700	-0.00040700
O	0.89364800	0.00008600	-1.23640200

K_3PO_4 (dichloromethane)

P	-0.06987100	0.00038100	-0.00109300
O	-0.95066200	1.27860600	-0.00301000
K	1.63506300	2.28086200	0.00080400
O	-0.95224500	-1.27678100	-0.00271100
K	-3.14159900	0.00211900	0.00088500
O	0.86762300	-0.00013500	1.24191900
K	1.62998300	-2.28382900	0.00079100
O	0.87310500	-0.00039100	-1.24004100

K_3PO_4 (acetonitrile)

P	-0.11878300	0.00102100	-0.00349700
O	-0.99769300	1.27835600	-0.00576400
K	1.72703200	2.19717300	0.00253100
O	-1.00000800	-1.27486300	-0.00568800
K	-3.20688500	0.00367500	0.00297700
O	0.82496600	-0.00003700	1.23734800
K	1.71689100	-2.20307700	0.00251500
O	0.83248900	-0.00007600	-1.23839400

K_3PO_4 (dimethylsulfoxide)

P	-0.11497700	0.00051200	-0.00256100
O	-0.99364300	1.27847800	-0.00398600
K	1.71325900	2.19749400	0.00188600
O	-0.99505100	-1.27650300	-0.00399600
K	-3.19437000	0.00212600	0.00220900
O	0.83088000	-0.00013500	1.23773100
K	1.70754700	-2.20074700	0.00188300
O	0.83561200	-0.00012400	-1.23914400

K_3PO_4 (water)

P	-0.22542200	-0.03083800	0.06107800
O	-1.11049600	-1.29954100	0.09083100
K	2.03844900	-2.01326900	-0.04442200
O	-1.11261400	1.23743400	0.10105100
K	-3.38750400	-0.03896200	-0.05940400
O	0.66195000	-0.02352900	-1.22048900
K	1.86060500	2.12575000	-0.04176300
O	0.76889500	-0.03114900	1.25985800

K_3PO_4 (formamide)

P	-0.22693100	-0.02812700	0.06233600
O	-1.11116300	-1.29664000	0.09559900
K	2.03077100	-2.00841400	-0.04517700
O	-1.11287800	1.24055900	0.10136500
K	-3.38094100	-0.03690500	-0.06069900
O	0.65835700	-0.02307700	-1.22026800
K	1.86476300	2.11205700	-0.04265800
O	0.76902100	-0.02660500	1.25919300

$K_4P_2O_7$

P	-0.38124700	-0.17480000	-1.49298400
O	0.19594200	1.24298100	-1.49293200
K	2.21951600	-1.79483000	-0.72081800
O	-1.90944000	-0.16701400	-1.40752600
K	1.87308100	2.15321500	0.23144500
P	0.38295300	-0.17091700	1.49359300
O	-0.28921500	-1.15053100	2.41511000
K	-2.20315400	-1.81187900	0.72087300
O	0.29955300	-1.14817600	-2.41486700
K	-1.89268400	2.13685500	-0.23261700
O	-0.20840600	1.24111000	1.49173100
O	0.00502600	-0.84486000	0.00007700
O	1.91103400	-0.14827200	1.40991900

$K_4P_2O_7$ (toluene)

P	-0.36391100	-0.18307800	-1.49313000
O	0.22882000	1.22611000	-1.49408100
K	2.25699200	-1.80606700	-0.70763000
O	-1.89215800	-0.16394800	-1.44390800
K	1.88908200	2.17122100	0.25524800
P	0.36379600	-0.18330000	1.49292600
O	-0.31370000	-1.15940600	2.41967600
K	-2.25845700	-1.80433900	0.70793300
O	0.31306500	-1.15973700	-2.41966600
K	-1.88740500	2.17238100	-0.25516200
O	-0.22785400	1.22634600	1.49403500
O	-0.00051700	-0.85844000	-0.00000400
O	1.89205700	-0.16530700	1.44340800

$K_4P_2O_7$ (dichloromethane)

P	-0.33574100	-0.19481200	-1.49000900
O	0.26123700	1.20969600	-1.48420100
K	2.32296400	-1.80797500	-0.68073900
O	-1.86337500	-0.17421400	-1.48370600
K	1.90053700	2.19734600	0.28759900
P	0.33518400	-0.19507900	1.49033700
O	-0.35443100	-1.16381500	2.42202900
K	-2.32755300	-1.80280800	0.68071700
O	0.35144700	-1.16564000	-2.42136000
K	-1.89484900	2.20187900	-0.28825000

O	-0.25809900	1.21097500	1.48414400
O	-0.00121300	-0.88003900	0.00026000
O	1.86286800	-0.17846400	1.48381700

$K_4P_2O_7$ (acetonitrile)

P	-0.32039800	-0.21102100	-1.49121000
O	0.28678800	1.18878000	-1.48075200
K	2.36233300	-1.77681400	-0.67067700
O	-1.84782900	-0.17927100	-1.50265400
K	1.89080500	2.20341700	0.31363100
P	0.31984000	-0.21132300	1.49144400
O	-0.36930700	-1.18286600	2.42185000
K	-2.36538200	-1.77348200	0.67045400
O	0.36684300	-1.18405600	-2.42147200
K	-1.88663200	2.20694600	-0.31389700
O	-0.28453100	1.18968800	1.48077500
O	-0.00093400	-0.90049800	0.00016500
O	1.84734700	-0.18254200	1.50281000

$K_4P_2O_7$ (dimethylsulfoxide)

P	-0.32554700	-0.20852900	-1.49355000
O	0.28031400	1.19241100	-1.48628200
K	2.34819500	-1.77569900	-0.67302100
O	-1.85331300	-0.17803800	-1.49848700
K	1.88633000	2.19958600	0.30186800
P	0.32548700	-0.20861300	1.49361400
O	-0.35973200	-1.18344400	2.42257600
K	-2.34829600	-1.77568500	0.67285000
O	0.35962700	-1.18336000	-2.42254900
K	-1.88611200	2.19989100	-0.30182600
O	-0.28027900	1.19236700	1.48638200
O	-0.00003900	-0.89378800	0.00001100
O	1.85325900	-0.17822600	1.49853400

$K_4P_2O_7$ (water)

P	-0.36013900	-0.18251100	-1.47243200
O	0.24710300	1.21289800	-1.49134600
K	2.34849000	-1.88932100	-0.67224900
O	-1.88534800	-0.16808400	-1.46039600
K	1.94749400	2.26425500	0.23515500
P	0.36025600	-0.18169200	1.47205200
O	-0.29418500	-1.15587200	2.43184700
K	-2.34737900	-1.89029300	0.67324500
O	0.29467000	-1.15691600	-2.43171700
K	-1.94888200	2.26306800	-0.23538100
O	-0.24754800	1.21349400	1.49026300
O	0.00028100	-0.87187100	0.00000700
O	1.88546600	-0.16657500	1.46022600

$K_4P_2O_7$ (formamide)

P	-0.35797500	-0.18477100	-1.47250200
O	0.25193800	1.20926400	-1.49200000
K	2.34626500	-1.87915200	-0.67209000
O	-1.88280800	-0.16747900	-1.46076600
K	1.94172000	2.25721300	0.23948600
P	0.35793700	-0.18415800	1.47250500
O	-0.29736200	-1.15968800	2.42934300
K	-2.34731200	-1.87762300	0.67279000
O	0.29659400	-1.16132200	-2.42880400
K	-1.94060900	2.25791200	-0.24020300
O	-0.25095300	1.21034200	1.49128300
O	-0.00027000	-0.87251900	0.00020600
O	1.88278300	-0.16794000	1.46077100

K_2CO_3

C	0.00019700	0.79641700	0.00535100
O	0.00197400	-0.54107100	0.01646500
O	-1.12029900	1.39743200	0.02271000
O	1.11817100	1.40154500	-0.02595700
K	2.47240800	-0.60115600	0.00100700
K	-2.47240500	-0.60104200	-0.00826300

Na_3PO_4

P	-0.09972200	0.00080900	0.18373300
O	-0.45791800	-1.26629800	-0.66178600
Na	1.47608000	-2.12285900	-0.18969100
O	-0.44830300	1.26984000	-0.66274300
O	1.45079800	-0.00500800	0.44195200
Na	1.49095700	2.11316200	-0.18999300
O	-0.96484200	0.00437500	1.43846100
Na	-2.52540600	0.00647800	-0.27514000

$K_2C_2O_4$

C	0.00000000	-0.80006900	0.00040300
O	1.11457000	-1.36617700	0.00015900
O	-1.11456000	-1.36616100	0.00040600
K	-3.15312800	0.00029400	-0.00041100
C	0.00001600	0.79950600	0.00061800
O	1.11453200	1.36567800	0.00099400
O	-1.11443200	1.36571100	0.00029400
K	3.15307700	0.00028300	-0.00069100

K_2HPO_4

P	0.00012600	0.69720700	-0.13634600
O	1.29861700	1.01916700	-0.84424500
K	-2.49816600	-0.93800800	0.02847600
O	-1.29870400	1.01905900	-0.84384700
K	2.49785600	-0.93819600	0.02847900
O	0.00030800	-0.76039700	0.39591100
O	0.00026800	1.55939400	1.27980600
H	0.00009400	2.49198800	1.06203100

K_2PO_3F

P	-0.00015300	0.71047900	-0.13172400
O	-1.30559400	1.04670800	-0.80005200
K	-2.50682700	-0.93635700	0.01176600
O	1.30493900	1.04702500	-0.80078200
K	2.50718700	-0.93607800	0.01184400
O	0.00022800	-0.74820200	0.39386700
F	-0.00012400	1.57275900	1.24255700

$(KPO_3)_3$

P	-1.38767100	1.41494000	0.06520300
O	-2.75872900	0.86623600	-0.01416300
O	-0.42320800	0.72937800	-1.09567200
P	-0.01883400	-0.83608800	-1.28670000
O	-1.15589200	-1.63407100	-1.79606700
O	0.14592000	-1.28409800	0.29516600
P	0.56013800	-0.21523500	1.49679600
O	0.57021200	-0.97432100	2.75052400
O	1.79676100	0.49380700	1.00121800

K	2.96230200	-1.58523600	-0.09265600
O	-0.70658400	0.77536100	1.42028600
O	1.33357500	-0.84682300	-1.91124400
K	-2.84234900	-1.67074700	0.17839200
O	-1.03377800	2.84958900	-0.00382100
K	1.48790400	2.55836600	-0.57517300

Li_3PO_4

P	-0.00001700	-0.17826000	0.00122800
O	0.93956800	0.82330100	-0.80544100
Li	-0.00236900	2.17904100	0.00025100
O	-0.97305500	-0.99001600	-0.84351700
O	-0.94364300	0.82103500	0.80382200
Li	-2.33361400	-0.20639200	0.07087800
O	0.97812900	-0.98296900	0.84699400
Li	2.33340600	-0.20495200	-0.08222000

KH_2PO_4

P	0.74542200	0.00001600	-0.00000600
O	-0.04017400	0.50439700	1.16915300
O	-0.04017100	-0.50425400	-1.16921700
K	-2.25020700	0.00000300	0.00000100
O	1.74640200	-1.14467400	0.54843200
H	2.13538100	-1.61897800	-0.18800500
O	1.74662400	1.14453200	-0.54837000
H	2.13576400	1.61867500	0.18808600

HCO_2K

C	1.47158000	0.00000100	0.00010000
O	0.90147200	1.11808100	0.00012200
H	2.57914500	0.00000300	0.00023100
O	0.90147600	-1.11808100	0.00012200
K	-1.35959000	-0.00000100	-0.00014700

C_4F_{10}

C	0.58356500	0.51526900	-0.09380500
F	0.56711800	1.17625900	-1.25664800
F	0.41040900	1.38906900	0.90777200
C	1.99606200	-0.11745700	0.07485200
F	2.15521300	-0.59232900	1.30120200
F	2.90895700	0.82054000	-0.14010500
F	2.17501400	-1.09960200	-0.79958600
C	-0.58356500	-0.51526400	-0.09380600
F	-0.56712000	-1.17625300	-1.25664900
F	-0.41040400	-1.38906400	0.90777100
C	-1.99606300	0.11745600	0.07485400
F	-2.17500000	1.09963000	-0.79955400
F	-2.15523500	0.59228400	1.30121900
F	-2.90895200	-0.82053600	-0.14015300

C_4F_{10} (toluene)

C	0.58390400	0.51484800	-0.08506000
F	0.55923000	1.18388000	-1.24355500
F	0.41843100	1.38143400	0.92353100
C	1.99834800	-0.11779800	0.06855800
F	2.16137500	-0.61578000	1.28527400
F	2.90763100	0.82775600	-0.12957700
F	2.18078600	-1.08192900	-0.82462800
C	-0.58390500	-0.51484900	-0.08507600
F	-0.55922600	-1.18385100	-1.24358800
F	-0.41843900	-1.38146100	0.92349500
C	-1.99834800	0.11779700	0.06855300
F	-2.18079000	1.08193300	-0.82462600
F	-2.16136600	0.61577400	1.28527200
F	-2.90763300	-0.82775500	-0.12958100

C_4F_{10} (dichloromethane)

C	0.58705700	0.51113700	-0.03642800
F	0.52177400	1.23562400	-1.16080000
F	0.46536800	1.32622200	1.01984700
C	2.00986000	-0.12101000	0.02979500
F	2.18028900	-0.78449800	1.16410500
F	2.90276200	0.85989600	-0.02798400
F	2.21255700	-0.94253100	-0.99067800
C	-0.58704700	-0.51112100	-0.03644100
F	-0.52174100	-1.23559700	-1.16081600
F	-0.46534400	-1.32622500	1.01981800
C	-2.00986600	0.12100700	0.02977500
F	-2.21258400	0.94251400	-0.99070500
F	-2.18033200	0.78449700	1.16407800
F	-2.90275300	-0.85991100	-0.02799900

C_4F_{10} (acetonitrile)

C	0.58625700	0.51154200	-0.00001300
F	0.49110000	1.28185300	-1.09162200
F	0.49109500	1.28182300	1.09163400
C	2.01018000	-0.12097100	-0.00000900
F	2.19793700	-0.86488200	1.08066700
F	2.90151800	0.86334400	-0.00002000
F	2.19791100	-0.86485800	-1.08070500
C	-0.58625700	-0.51154200	-0.00001700
F	-0.49111700	-1.28182400	-1.09164800
F	-0.49107700	-1.28185200	1.09160800
C	-2.01018000	0.12097100	0.00002900
F	-2.19794400	0.86486100	-1.08066000
F	-2.19790300	0.86487900	1.08071100
F	-2.90151800	-0.86334400	0.00004300

C_4F_{10} (dimethylsulfoxide)

C	-0.58625700	0.51154200	0.00001300
F	-0.49110000	1.28185300	1.09162200
F	-0.49109500	1.28182300	-1.09163400
C	-2.01018000	-0.12097100	0.00000900
F	-2.19793700	-0.86488200	-1.08066700
F	-2.90151800	0.86334400	0.00002000
F	-2.19791100	-0.86485800	1.08070500
C	0.58625700	-0.51154200	0.00001700
F	0.49111700	-1.28182400	1.09164800
F	0.49107700	-1.28185200	-1.09160800
C	2.01018000	0.12097100	-0.00002900
F	2.19794400	0.86486100	1.08066000
F	2.19790300	0.86487900	-1.08071100
F	2.90151800	-0.86334400	-0.00004300

C_4F_{10} (water)

C	0.58584900	0.51156300	-0.00016800
F	0.49066100	1.28142600	-1.09191900
F	0.49044700	1.28171200	1.09138900
C	2.00908400	-0.12092400	0.00013700
F	2.19616900	-0.86440400	1.08099600

F	2.90048200	0.86307800	-0.00015300
F	2.19633200	-0.86514600	-1.08021400
C	-0.58584800	-0.51156300	-0.00018200
F	-0.49063500	-1.28142000	-1.09193500
F	-0.49046600	-1.28172100	1.09137100
C	-2.00908400	0.12092500	0.00010500
F	-2.19636100	0.86508200	-1.08028500
F	-2.19614600	0.86446800	1.08092400
F	-2.90048300	-0.86307600	-0.00010200

C_4F_{10} (formamide)

C	-0.58607400	-0.51153200	-0.00010000
F	-0.49095100	-1.28167100	-1.09173100
F	-0.49086900	-1.28192800	1.09136300
C	-2.00960700	0.12087100	0.00006200
F	-2.19740000	0.86463700	1.08068800
F	-2.90113700	-0.86311100	0.00009200
F	-2.19763500	0.86478400	-1.08044400
C	0.58607500	0.51153400	-0.00006500
F	0.49095800	1.28174500	-1.09164500
F	0.49086900	1.28185700	1.09145000
C	2.00960600	-0.12087200	0.00005400
F	2.19757400	-0.86481800	-1.08043900
F	2.19745200	-0.86460600	1.08069400
F	2.90113800	0.86311000	0.00000600

Tetracyanoethylene $C_2(CN)_4$

C	0.00023700	0.67617200	-0.00000500
C	-0.00023700	-0.67617300	-0.00000500
C	1.21992500	-1.42184200	0.00000000
N	2.19395300	-2.02875200	0.00000200
C	1.22086500	1.42104300	0.00000000
N	2.19528900	2.02731400	0.00000200
C	-1.21992500	1.42184200	0.00000000
N	-2.19395300	2.02875200	0.00000300
C	-1.22086500	-1.42104300	0.00000000
N	-2.19528900	-2.02731400	0.00000200

TS1a

C	-3.35942100	0.35761100	-0.00846100
F	-3.67983500	0.55217900	1.25888100
F	-3.90342400	-0.75383600	-0.46417400
C	-0.48908500	-1.94624700	-0.33530300
F	0.42939400	-1.94556400	-1.32209300
F	-1.57230600	-2.46192200	-0.90969400
F	-0.02650800	-2.78436900	0.58667200
C	-1.81931500	0.45175400	-0.27309200
F	-1.51162200	1.69958500	0.17078000
F	-1.71169000	0.43188600	-1.61457900
C	-0.81663300	-0.56866600	0.35579500
F	-3.91339300	1.37522700	-0.70567400
F	-2.04638700	-1.38216000	1.17886300
F	-0.26306400	-0.27093300	1.54629000
P	1.99423300	0.62836500	0.04466000
O	2.72107500	1.12272100	-1.21163100
O	2.57973400	-0.68327700	0.59235500
O	0.51664700	0.34193700	-0.52109000
O	1.87664700	1.70688200	1.12566500
K	1.99855200	-0.02440000	2.97516300
K	0.75790900	2.97807900	-0.96141900
K	3.09774500	-1.33468700	-1.83023700

TS1b

C	2.24357000	0.54324900	-0.10397400
F	2.92513600	1.32304200	0.75828300
F	2.70844500	0.83785300	-1.33834500
C	2.73024800	-0.91410300	0.17378700
F	2.31746400	-1.76873900	-0.77690300
F	2.33752400	-1.38803300	1.34529300
F	4.06334300	-0.92907900	0.15052100
C	0.64326800	0.77617500	0.00490000
F	0.72954000	0.65095000	1.49886000
F	0.42290500	0.02156900	-1.24124300
C	0.29015900	2.26839600	-0.29685200
F	1.37547700	2.99042000	-0.60571600
F	-0.27902700	2.86770200	0.74249300
F	-0.53533400	2.38100600	-1.34382300
P	-1.91243200	-0.96428500	0.09885500
O	-1.14727600	0.45881800	0.33027300
O	-3.11510800	-0.75895700	1.01698200
O	-0.94267000	-2.05995500	0.54389100
O	-2.29225300	-1.05891100	-1.38139500
K	-3.43643400	1.24890900	-0.79795300
K	-0.26245800	-2.51326400	-1.86351300
K	-1.10994100	-0.68860400	2.82289900

TS1c

C	2.73558600	0.65424300	0.04683400
F	3.12789200	1.26833300	1.16502700
F	2.84086700	1.45895900	-1.01450200
C	3.82086300	-0.45575100	-0.19652200
F	3.65707700	-1.07772800	-1.36295000
F	3.83456000	-1.37505100	0.76560500
F	5.01400700	0.13415200	-0.21698400
C	1.28739300	0.07630600	0.19279900
F	1.34131800	-0.74961500	1.25579100
F	1.13089700	-0.67578200	-0.93423300
C	0.01766000	0.99420700	0.35967000
F	-0.57533500	1.50984200	-0.73229100
F	1.00816300	2.38601100	0.41604700
F	-0.42504800	1.32082900	1.59062500
P	-2.53013300	-0.52651400	-0.15213300
O	-2.90245600	-1.87943800	0.43993200
O	-2.50301400	-0.49729600	-1.67968200
O	-0.97370000	-0.43537800	0.36309200
O	-3.26480200	0.66474800	0.46076400
K	-3.07177200	1.99468600	-1.69343400
K	-2.33558100	-0.45071100	2.69376100
K	-0.94290100	-2.61434100	-1.27384000

TS1d

C	-2.63659400	-0.44557200	0.25464600
F	-2.42379400	-0.87330100	1.50717700
F	-2.51743400	-1.49691200	-0.58118200
C	-4.13337100	-0.01879200	0.18434000
F	-4.41428600	0.54534200	-0.98626600
F	-4.44847000	0.82258700	1.15925800
F	-4.89806900	-1.10355400	0.31694400
C	-1.63087600	0.68457400	-0.15180400
F	-2.19266000	1.85337500	0.22718100

F	-1.58303900	0.67589800	-1.50358800
C	-0.12087600	0.58201900	0.43854200
F	-0.09355000	-0.87263300	0.07647700
F	-0.31898100	0.75966800	1.75015100
F	0.23881900	1.85229900	-0.20329100
P	2.68676700	-0.31405300	-0.32422500
O	3.99323000	0.31189100	0.16306700
O	2.26831400	0.09359700	-1.73697100
O	1.63765600	0.39980600	0.70962400
O	2.61867800	-1.82491600	-0.08979600
K	0.92868000	-2.05479400	-2.01627800
K	3.20210800	-1.04926400	2.35406100
K	2.74020900	2.50350400	-0.74431500

TS1e (toluene)

C	-3.39756700	0.34104100	-0.00396500
F	-3.72248900	0.52546700	1.26568600
F	-3.93408500	-0.77648300	-0.46065000
C	-0.49879400	-1.93711200	-0.33995400
F	0.41378200	-1.91156200	-1.31559700
F	-1.58374500	-2.46015900	-0.91954900
F	-0.04095600	-2.78583900	0.57715200
C	-1.85682600	0.44392900	-0.26319400
F	-1.55947200	1.68833300	0.18111100
F	-1.75060800	0.41464700	-1.60364500
C	-0.86627700	-0.58198800	0.37653700
F	-3.96099700	1.35279000	-0.69672200
F	-2.07046400	-1.40352300	1.18852100
F	-0.29834800	-0.27064800	1.54752300
P	1.96903800	0.62506100	0.01996700
O	2.72627700	1.08585500	-1.23331200
O	2.52636900	-0.69202500	0.58867300
O	0.48678000	0.38018700	-0.52954900
O	1.89233400	1.72205200	1.09085800
K	2.09048000	0.03978300	2.99556900
K	0.75778600	3.00553100	-1.00975300
K	3.24087000	-1.37672800	-1.78180900

TS1f (dichloromethane)

C	-3.44479000	0.32024200	0.02132500
F	-3.77033800	0.45258400	1.29874500
F	-3.97486200	-0.78649000	-0.47345900
C	-0.52255800	-1.92770100	-0.40051200
F	0.40647900	-1.86317500	-1.34670400
F	-1.60486200	-2.41757000	-1.02105800
F	-0.09460500	-2.82221900	0.49039500
C	-1.90365100	0.44085200	-0.23388800
F	-1.61235100	1.66566700	0.25366800
F	-1.80094300	0.45518800	-1.57487000
C	-0.91241500	-0.60814100	0.37069800
F	-4.01609300	1.34964100	-0.63210800
F	-2.10786000	-1.46733900	1.14892300
F	-0.33817700	-0.33008600	1.54059800
P	1.93335600	0.62228400	0.00864500
O	2.71113800	1.04756400	-1.24787400
O	2.47315100	-0.69161800	0.60142500
O	0.44511000	0.41554600	-0.52065800
O	1.89897200	1.74233200	1.06176500
K	2.16674200	0.10992900	3.02923800
K	0.74848400	3.01845300	-1.10144600
K	3.48981000	-1.38744400	-1.66410000

TS1g (acetonitrile)

C	-3.46670700	0.31767800	0.04732300
F	-3.77973900	0.42169500	1.33113100
F	-4.00287200	-0.77906400	-0.46500300
C	-0.54273200	-1.92210700	-0.44627400
F	0.38432000	-1.83576700	-1.38771800
F	-1.62852500	-2.39637100	-1.07665500
F	-0.11819400	-2.83943100	0.42447100
C	-1.92740300	0.44038800	-0.21899500
F	-1.63070400	1.65427100	0.28942600
F	-1.83624000	0.47775400	-1.56004600
C	-0.93691900	-0.62430600	0.35912900
F	-4.04300000	1.35989300	-0.57787900
F	-2.12464000	-1.50140600	1.12174700
F	-0.35215500	-0.36911900	1.52582800
P	1.91034200	0.61754100	0.00534200
O	2.69430600	1.07937400	-1.23522100
O	2.45217000	-0.71257300	0.55987500
O	0.42292600	0.43073200	-0.52696100
O	1.88234900	1.70642300	1.09220000
K	2.27157200	0.03419500	3.01207100
K	0.72512200	3.05082600	-1.03768100
K	3.59063000	-1.31116600	-1.67302600

TS1h (dimethylsulfoxide)

C	-3.46948500	0.29720700	0.03368100
F	-3.78652800	0.43244000	1.31364500
F	-3.99466600	-0.81811800	-0.44963100
C	-0.52649900	-1.93114900	-0.39449800
F	0.39208700	-1.86041800	-1.34414600
F	-1.61049100	-2.43872100	-1.00391800
F	-0.08631100	-2.81721100	0.50054900
C	-1.93089700	0.42471400	-0.23266800
F	-1.64470700	1.65341800	0.24632900
F	-1.83848800	0.42786000	-1.57360100
C	-0.93620000	-0.61775900	0.37688600
F	-4.05361600	1.31676900	-0.62039100
F	-2.11035600	-1.47879200	1.16298900
F	-0.34978400	-0.32064600	1.53298700
P	1.91146400	0.62386800	-0.03471500
O	2.69167200	1.03282000	-1.29430000
O	2.44435200	-0.69023700	0.56775600
O	0.41730300	0.42882900	-0.54414100
O	1.89531800	1.75153200	1.01399800
K	2.26537800	0.14035300	2.98422300
K	0.68291500	3.04418500	-1.05834100
K	3.61292200	-1.36605800	-1.61059300

TS1i (water)

C	-3.44721400	0.05106600	0.09698900
F	-3.74774600	0.44716200	1.32300500
F	-3.85494300	-1.19034400	-0.10150200
C	-0.35499400	-1.93191000	0.04170000
F	0.53480000	-2.02850100	-0.94721200
F	-1.41591700	-2.61431200	-0.39166500
F	0.18018400	-2.55857300	1.08576500
C	-1.94042200	0.27430800	-0.26850500
F	-1.77261400	1.60302700	-0.06278200

F	-1.88134000	0.01736400	-1.58426600
C	-0.79123600	-0.48154600	0.47479100
F	-4.14777000	0.83138000	-0.74499300
F	-1.90060100	-1.25658500	1.55417000
F	-0.23292100	0.11604500	1.51506700
P	1.98973000	0.54148200	-0.46549400
O	2.67072100	0.05320600	-1.74935500
O	2.42648900	-0.30457300	0.74074800
O	0.40890400	0.40896400	-0.68424400
O	2.23980900	2.04113700	-0.20217800
K	2.31517000	1.74747400	2.41379300
K	0.14940500	2.96781200	-1.44016600
K	3.40593400	-2.26705200	-0.69665300

TS1j (formamide)

C	-3.44961200	0.05615300	0.09731600
F	-3.75105500	0.44670300	1.32499000
F	-3.85975000	-1.18353400	-0.10762500
C	-0.35826100	-1.93229100	0.03695900
F	0.53082700	-2.02502800	-0.95298900
F	-1.41864900	-2.61459800	-0.39777000
F	0.17836000	-2.56189400	1.07884200
C	-1.94190000	0.27853500	-0.26538700
F	-1.77191000	1.60587300	-0.05274000
F	-1.88168500	0.02839300	-1.58261300
C	-0.79431500	-0.48313400	0.47537300
F	-4.14804000	0.84179800	-0.74151400
F	-1.90667700	-1.26256300	1.55011400
F	-0.23690100	0.10850700	1.51951200
P	1.98923600	0.53557900	-0.46542900
O	2.66328300	0.03805800	-1.74889300
O	2.42483700	-0.30632900	0.74380000
O	0.40720700	0.41102000	-0.67742800
O	2.24881500	2.03489100	-0.21111300
K	2.32084500	1.75433000	2.40013100
K	0.15882300	2.96263500	-1.43652600
K	3.40781500	-2.26556000	-0.68355000

TS1k

C	2.02180600	3.08625200	0.85424300
F	0.78775100	3.56921700	0.85154200
F	2.75597000	3.69008400	-0.06118800
C	2.40143200	0.47753600	-1.78675200
F	3.16745400	-0.61057400	-1.74480500
F	3.24950400	1.48588600	-2.00654200
F	1.60377200	0.33419900	-2.84202600
C	2.05362700	1.52388000	0.73953700
F	1.33008900	1.13130300	1.81918000
F	3.34289500	1.20372800	0.93245100
C	1.49564200	0.82455700	-0.54273000
F	2.55633400	3.38793700	2.05216500
F	1.05409700	2.22210900	-1.28254200
F	0.24084900	0.39593300	-0.51656200
P	1.12782500	-2.15754800	0.39166700
O	2.12887500	-3.21361600	0.92207600
O	0.68458700	-2.49959000	-1.03457400
O	2.02123200	-0.83174900	0.37894900
O	-0.02693700	-1.92362800	1.37041400
K	-1.88990600	-2.05911400	-0.96405700
K	2.43374800	-1.55027400	2.85473600
K	2.89295300	-3.60199400	-1.46993700
P	-3.66087800	0.50703100	-0.35378400
O	-2.79880400	1.57300200	0.37897400
K	-1.57079700	2.28214800	-1.71296800
O	-3.65881700	-0.80591100	0.54902700
K	-1.65548400	-0.00030700	1.98081900
O	-2.94645200	0.16022100	-1.68603300
K	-6.15566300	-0.81941600	0.69967100
O	-5.11366500	0.94454400	-0.54709700

TS1l

C	2.57720200	-2.17083100	-0.32094200
F	3.24636800	-2.43839800	-1.44391100
F	1.44057900	-2.87860000	-0.25878100
C	3.46528800	-2.75105400	0.83522700
F	2.84976800	-2.69681200	2.01426100
F	4.62997500	-2.11746400	0.94381200
F	3.71300900	-4.02807600	0.56185600
C	2.29737200	-0.63430500	-0.18055800
F	3.50591600	-0.05209500	-0.21376800
F	1.77925600	-0.52345500	1.06976000
C	1.35019900	0.10908800	-1.18970500
F	0.05789800	0.16766100	-0.93778700
F	1.13060900	-1.25953800	-2.14869900
F	1.84658400	0.76458900	-2.22608600
P	0.51373200	2.65797100	0.40163700
O	1.39011500	3.81979200	0.90326300
O	-0.20059100	1.92510600	1.53617700
O	1.67626100	1.66510700	-0.15769500
O	-0.34946600	3.06728700	-0.78761600
K	-2.66565500	1.93160400	-0.76514500
K	1.94944000	3.90587400	-1.69974400
K	2.38982700	2.02716800	2.44483300
P	-3.56230500	-1.06176800	-0.24159300
O	-2.36775300	-1.93021700	0.24067200
K	-1.43872200	-0.30444400	1.91997700
O	-3.16788100	-0.42580400	-1.59966500
K	-1.33847000	-2.12753900	-2.06439600
O	-3.74162100	0.12351700	0.81048200
K	-6.11212400	-0.53522300	1.28774400
O	-4.87819700	-1.83880200	-0.30222200

TS2a

C	4.21433200	-0.54299800	0.14981100
F	4.26271000	-0.49997800	1.47474600
F	4.73603100	0.55379600	-0.36565200
C	1.32964900	1.33319400	-1.10352100
F	0.55433000	1.09720000	-2.15685500
F	2.48153300	1.76242700	-1.59641000
F	0.72959000	2.35477900	-0.45720700
C	2.77778300	-0.86368000	-0.38237100
F	2.47370100	-2.03618400	0.20438600
F	2.92075100	-1.03913400	-1.70058700
C	1.59802500	0.12484900	-0.11308400
F	4.99088000	-1.56944800	-0.23547900
F	2.54661400	1.26910200	0.78360100
F	0.93332900	-0.10187700	1.03805500
P	-2.62190000	0.73654500	1.00005500

O	-1.32273600	0.29027700	-0.00599400
O	-3.82832200	0.03935400	0.42357600
P	-1.05361400	-1.07779100	-0.84969200
O	-2.55616300	2.23615200	0.77068100
O	-2.12357900	0.31013800	2.37278500
K	-3.93537800	-2.09664400	-0.92277200
O	-1.84602000	-0.91449500	-2.12762300
O	0.48823300	-1.02865900	-1.00960900
O	-1.50820300	-2.26040800	-0.01296900
K	-0.23577600	2.26336000	2.04517200
K	-2.49464300	1.67591700	-1.89403800
K	-0.83298800	-1.87866200	2.36402600

TS2b

C	-2.62666000	1.14671400	-0.23236600
F	-3.61786100	1.15787300	0.67803200
F	-3.19870700	1.45551000	-1.41506400
C	-1.69468900	2.35197300	0.10290400
F	-0.80074700	2.56395000	-0.83573900
F	-1.07014500	2.19029900	1.27640700
F	-2.45737800	3.44335900	0.21402900
C	-1.89726000	-0.28633500	-0.31005300
F	-1.85203900	-0.32815200	1.24519700
F	-1.26857300	0.04776800	-1.59829500
C	-2.89730600	-1.43412600	-0.67455400
F	-4.13129100	-0.97040000	-0.87024400
F	-2.99839500	-2.35114500	0.30718300
F	-2.53590600	-2.07431900	-1.77348200
P	0.96425500	-1.11384200	0.37601600
O	1.16652700	0.40028200	-0.09579700
O	1.90733500	-2.01949200	-0.37571100
P	2.73492000	1.19843600	-0.32314300
O	-0.49892400	-1.45764100	-0.15699400
O	0.98922900	-1.16961400	1.88976200
K	4.39706400	-1.37869200	-0.45992300
O	3.16221800	0.66518800	-1.67918200
O	2.32340200	2.61566000	-0.14031800
O	3.54883100	0.60919000	0.82243000
K	-1.07311300	-2.55145000	2.24769500
K	1.07804900	-0.46117500	-2.64644900
K	1.58738300	1.48081400	2.37055100

TS2c

C	3.82838700	0.30903900	-0.03604400
F	3.92117500	0.93395300	1.14328800
F	3.96800600	1.17267100	-1.04684500
C	2.47920300	-0.47487200	-0.14740700
F	2.46361900	-1.32254200	0.89106200
F	2.55130600	-1.15420400	-1.29687500
C	1.12559500	0.30942500	-0.13293600
F	1.82220900	1.90472900	0.09394100
F	0.53288300	0.49578800	1.05648100
P	-3.12096400	1.11668000	0.15912600
O	-1.71174900	0.26118300	-0.27066600
O	-4.24104800	0.10914200	0.03418700
P	-1.32068400	-1.31936800	-0.18186600
O	-3.06853800	2.16446900	-0.93884700
O	-2.76773000	1.64151400	1.53766800
K	-4.13546500	-2.41339800	0.29493400
O	-2.05910100	-1.98884500	-1.32216900
O	0.21774000	-1.26861500	-0.35993400
O	-1.75859500	-1.82549300	1.18319600
K	-0.69901100	2.95704500	0.03426700
K	-2.94960900	0.10540500	-2.65908500
K	-1.50754900	-0.04144800	2.93172700
F	0.63382800	0.75372300	-1.27642500
C	5.09019800	-0.61696700	-0.11173300
F	6.16749300	0.15419800	0.04057500
F	5.18865700	-1.23160300	-1.28319900
F	5.09945400	-1.53013000	0.85214500

TS2d

C	3.83556500	0.26480100	-0.26820200
F	4.02625200	1.23446500	0.64499600
F	3.75596000	0.82106500	-1.48252500
C	5.14363700	-0.58648200	-0.27231000
F	5.00298500	-1.67559400	-1.02043800
F	5.50194400	-0.94500900	0.95365700
F	6.12966300	0.14856100	-0.78636900
C	2.54691400	-0.57049900	0.04655300
F	2.93782600	-1.52657100	0.92458200
F	2.18107000	-1.17899500	-1.08819500
C	1.28608800	0.20453100	0.67560100
F	1.54692300	1.38216800	-0.31650800
F	1.68516900	0.64767400	1.86542900
F	0.56850500	-1.05255600	0.91413400
P	-1.45021100	0.82344200	-0.29969100
O	-2.65153500	0.19686500	0.56719200
O	-0.99617600	-0.06900900	-1.40262500
P	-3.82699800	-1.05758400	0.19134600
O	-0.32041700	0.97779800	0.82655300
O	-1.91809800	2.22851800	-0.65689800
K	-2.08651700	-2.23312000	-2.04688700
O	-3.01675200	-2.31360300	0.45605000
O	-4.89098400	-0.64886800	1.15755400
O	-4.12555100	-0.80944700	-1.27535100
K	0.15423300	3.46528200	0.09245500
K	-1.58389000	-1.32472600	2.37377200
K	-4.60938600	1.69483300	-0.54162200

TS2e (toluene)

C	4.26517400	-0.58052100	0.16386200
F	4.34459100	-0.51905600	1.48602100
F	4.82016300	0.48735800	-0.38051400
C	1.39968500	1.37137800	-1.07302100
F	0.59475200	1.15282000	-2.10588300
F	2.55093300	1.77387100	-1.59760700
F	0.84446000	2.40175900	-0.40691800
C	2.81033500	-0.86086500	-0.34101100
F	2.48253400	-2.01534800	0.26581300
F	2.94203900	-1.05995500	-1.66014800
C	1.65983400	0.16669400	-0.08167400
F	5.00008100	-1.64050500	-0.21835500
F	2.67717600	1.27395300	0.78185200
F	0.97314800	0.00325100	1.05870000
P	-2.69559600	0.75091000	0.89094800
O	-1.33394600	0.31972700	-0.03172900
O	-3.85046700	-0.02473700	0.30805000
P	-0.99970400	-1.08287300	-0.79366700
O	-2.69591900	2.24050700	0.59501100

O	-2.25224400	0.41769500	2.30765400
K	-3.84302900	-2.27617500	-0.88895700
O	-1.77370500	-1.03648000	-2.09245700
O	0.53704000	-0.98098200	-0.97368900
O	-1.40739700	-2.23185800	0.10831300
K	-0.43817100	2.41935300	1.98252800
K	-2.57662400	1.51781200	-2.06428500
K	-0.94409600	-1.76388500	2.53681900

TS2f (dichloromethane)

C	4.36779600	-0.54689400	0.25067800
F	4.48145000	-0.35913200	1.55733800
F	4.93105900	0.45115200	-0.40932100
C	1.47411700	1.32561400	-1.10906400
F	0.65030400	1.02405900	-2.10263000
F	2.61307800	1.69332000	-1.69202100
F	0.93443000	2.39518300	-0.49996000
C	2.89941400	-0.84877500	-0.20015300
F	2.57271200	-1.94075100	0.51137900
F	3.01779000	-1.16837400	-1.50070200
C	1.75728500	0.20933500	-0.02949100
F	5.07776600	-1.65294100	-0.04183300
F	2.82938100	1.36435300	0.70972500
F	1.05884300	0.18794500	1.10322900
P	-2.80493100	0.77578900	0.54798900
O	-1.34898800	0.27904200	-0.14852000
O	-3.82455600	-0.28481200	0.21222400
P	-0.86677900	-1.23240900	-0.52476400
O	-2.99293200	2.09642800	-0.18273700
O	-2.44315700	0.95875400	2.01473200
K	-3.54750100	-2.79083900	-0.26370300
O	-1.62863800	-1.63586100	-1.76847700
O	0.63980600	-1.02441700	-0.82062100
O	-1.13544200	-2.12689700	0.66827800
K	-1.12248100	3.18506200	1.30689300
K	-2.72184600	0.67255000	-2.52263300
K	-1.00533400	-0.96349000	2.93543900

TS2g (acetonitrile)

C	4.35681900	-0.59655400	0.23909100
F	4.46248400	-0.44258700	1.55060500
F	4.94418400	0.40744400	-0.39110400
C	1.50423900	1.35968800	-1.09910700
F	0.68221500	1.09359800	-2.10233200
F	2.65463300	1.71947400	-1.66729100
F	0.98133100	2.42504800	-0.47027200
C	2.88675600	-0.85864500	-0.23141800
F	2.53593800	-1.96413700	0.44637500
F	3.01078700	-1.14494900	-1.53976200
C	1.76445800	0.21710100	-0.04045600
F	5.04847900	-1.70767000	-0.07555700
F	2.84883300	1.33485700	0.73021500
F	1.05703500	0.17640300	1.08339100
P	-2.78239000	0.79790700	0.60335000
O	-1.34223300	0.33127100	-0.14816400
O	-3.83148300	-0.19455200	0.16062200
P	-0.88946000	-1.16748800	-0.60241000
O	-2.93105800	2.18821000	0.00791600
O	-2.42102700	0.81948100	2.08046100
K	-3.61464400	-2.68623300	-0.44867600
O	-1.65009500	-1.48365100	-1.87234400
O	0.62559200	-0.98400400	-0.87388200
O	-1.18807600	-2.12430500	0.53385000
K	-0.92433400	2.99993000	1.54474800
K	-2.77816100	0.87282900	-2.45530500
K	-1.11034000	-1.22246100	2.91666200

TS2h (dimethylsulfoxide)

C	4.33748700	-0.63253000	0.19685000
F	4.44143000	-0.55364400	1.51524000
F	4.93133300	0.40313000	-0.37343500
C	1.50800500	1.41525400	-1.03784900
F	0.68581700	1.21209100	-2.05479300
F	2.66523500	1.78848600	-1.58395900
F	0.99714600	2.45486900	-0.35802000
C	2.86630100	-0.85763100	-0.28935700
F	2.50568600	-1.99525200	0.32665500
F	2.98990500	-1.07182400	-1.61103500
C	1.75555000	0.21730500	-0.03810900
F	5.02321700	-1.72743000	-0.17969600
F	2.83716400	1.28117300	0.79457000
F	1.04560600	0.11130800	1.08116900
P	-2.76100400	0.78716800	0.71899400
O	-1.33388600	0.36914500	-0.09517300
O	-3.84653200	-0.08328600	0.13045700
P	-0.92470400	-1.08284700	-0.71716900
O	-2.83673000	2.25284500	0.32883600
O	-2.40708200	0.57447500	2.18219900
K	-3.70810000	-2.49271600	-0.76212900
O	-1.67872800	-1.21627800	-2.02301500
O	0.60156100	-0.92926600	-0.93829200
O	-1.27720500	-2.15729200	0.29188000
K	-0.69233100	2.65935300	1.86832300
K	-2.73987700	1.22363300	-2.29804800
K	-1.19613800	-1.60227600	2.76859100

TS2i (water)

C	-4.22851200	0.64371800	-0.06293100
F	-4.30286400	0.97665600	1.21575200
F	-4.77720300	-0.54149500	-0.26371600
C	-1.39787200	-1.59321700	-0.69749900
F	-0.63640600	-1.68353800	-1.78066700
F	-2.57487500	-2.10400600	-1.04852500
F	-0.83254200	-2.38893000	0.21616000
C	-2.77658400	0.76195400	-0.63310800
F	-2.44296500	2.04249500	-0.40638000
F	-2.91248600	0.55982800	-1.95531300
C	-1.61945200	-0.14252400	-0.10040000
F	-4.96468000	1.53774300	-0.74384900
F	-2.63438800	-0.95551300	1.10030400
F	-0.92266400	0.34059100	0.92559200
P	2.52086400	-0.64329800	1.08068700
O	1.32312300	-0.47391700	-0.07821500
O	3.78930200	-0.15838300	0.40516600
P	1.04978900	0.78105800	-1.07319900
O	2.45158700	-2.14115300	1.30967500
O	2.03803400	0.16237600	2.27580500
K	4.02721400	1.88430700	-1.23196500
O	1.82906700	0.47867100	-2.33512700
O	-0.48846500	0.68570700	-1.30075500

O	1.46326400	2.06829300	-0.39768900
K	0.02447300	-1.64161800	2.74085100
K	2.72451700	-2.03660000	-1.78428300
K	1.13627300	2.57742500	2.09046100

TS2j (formamide)

C	4.27676000	-0.66120600	0.03352000
F	4.36401500	-0.84806500	1.34072800
F	4.84502300	0.48186200	-0.30898400
C	1.44885600	1.53548200	-0.80896800
F	0.65649600	1.52283400	-1.87491100
F	2.61572300	2.00262800	-1.24044000
F	0.90615100	2.42205500	0.03472800
C	2.81667100	-0.81900900	-0.50672500
F	2.46968300	-2.06253700	-0.13607900
F	2.94434100	-0.76705200	-1.84466800
C	1.67187000	0.15295700	-0.07029500
F	4.98955300	-1.64133700	-0.54848600
F	2.72688800	1.07848200	1.01243400
F	0.98141200	-0.19446600	1.01360100
P	-2.62542500	0.71055100	0.96017100
O	-1.31449900	0.43027300	-0.05194100
O	-3.82142300	0.10892900	0.25330200
P	-1.00341000	-0.88389300	-0.95456100
O	-2.59860300	2.22703300	0.99012300
O	-2.22574500	0.07745500	2.28246500
K	-3.93422100	-2.06035500	-1.22015100
O	-1.75302800	-0.68563200	-2.25402200
O	0.53703800	-0.78637000	-1.15795400
O	-1.41339500	-2.12588400	-0.19696700
K	-0.22730100	1.95175100	2.50984200
K	-2.65176100	1.87017900	-1.95618900
K	-1.27243900	-2.31764200	2.35488200

TS3

C	2.76908800	-0.51567100	-0.37050100
F	2.85850000	-1.17470300	-1.51123900
F	3.04900200	-1.30232200	0.64925000
C	-0.45621000	-1.05469400	1.28800700
F	-1.10739600	-0.19858600	2.05380600
F	0.56089000	-1.49229900	2.02033900
F	-1.30468100	-2.08939700	1.07538800
C	1.40858400	0.24151000	-0.21678000
F	1.36831100	1.05426600	-1.29428800
F	1.57583700	1.01076000	0.88730200
C	0.05237500	-0.52876400	-0.11067600
F	3.71878100	0.44338200	-0.39881100
F	0.78306600	-2.04271000	-0.31769800
F	-0.62057100	-0.74293900	-1.26137400
C	-2.09022600	1.31126800	-0.12510400
O	-0.76050000	1.10592300	0.03727000
O	-2.42341900	2.52056100	-0.16956900
O	-2.86043600	0.32414400	-0.20659000
K	-2.98638200	-1.91542100	-1.02946800
K	-0.15480100	3.51984200	0.12406500

TS4

C	3.13966600	-0.32079100	-0.04448600
F	3.44875600	-0.72269100	1.17521100
F	3.61055800	0.88609000	-0.27848000
C	0.14262800	1.81615600	-0.04058300
F	-0.77903600	1.94495100	-1.02191800
F	1.21000600	2.44123900	-0.52566200
F	-0.33925200	2.48104600	0.99860100
C	1.61431100	-0.46461900	-0.36808200
F	1.39058700	-1.80310500	-0.13760100
F	1.51862000	-0.24460000	-1.68377300
C	0.51436200	0.34490400	0.39375800
F	3.76868900	-1.15895700	-0.89716200
F	1.69245600	1.07723500	1.39882900
F	-0.00628200	-0.21345200	1.51331000
P	-2.27057900	-0.64602400	-0.20815200
O	-3.04791700	-0.63612700	-1.50505200
O	-2.67831600	0.53115100	0.70275400
O	-0.72243000	-0.45923600	-0.63397000
O	-2.28830500	-1.96706400	0.58920500
Na	-2.19741500	-0.70112200	2.48055700
Na	-0.68818800	-2.74274700	-0.79043300
Na	-3.30960500	1.57994900	-1.20076600

TS5

C	3.23827300	-0.14718400	0.04850200
F	3.77481800	-0.47439200	-1.10262600
F	3.47096200	-1.03853900	0.98175200
C	-0.03573700	-1.67674800	0.49973100
F	-1.16575000	-1.23795900	1.16467700
F	0.79799300	-1.94985000	1.48340400
F	-0.43073400	-2.76490500	-0.12808200
C	1.73936600	0.28798900	-0.06143300
F	1.77359800	1.34194600	-0.93201000
F	1.44653500	0.80982500	1.16805900
C	0.52199200	-0.58869400	-0.49919300
F	3.87371900	0.99287100	0.45095400
F	1.86109000	-2.01679900	-0.67082600
F	0.37285200	-0.76186900	-1.79446700
C	-2.75621200	1.54729500	-0.06567600
O	-3.84744500	1.09049100	0.31211500
O	-2.31776300	2.70799900	-0.00282200
K	0.10216700	3.18374500	0.31117700
C	-1.80535000	0.45761300	-0.66108200
O	-2.22629800	-0.48955400	-1.29025700
O	-0.55059700	0.63057000	-0.30459100
K	-4.09442500	-1.44167300	0.16944600

TS6

C	-3.00524500	0.42123500	0.53807500
F	-3.08365700	0.21724000	1.83817200
F	-3.72299100	-0.45129100	-0.13169400
C	-0.51187500	-1.73302700	-1.07733400
F	0.23292200	-1.48747900	-2.15830800
F	-1.74470900	-1.91179700	-1.53958800
F	-0.04990800	-2.85793700	-0.54832100
C	-1.53499700	0.56165000	0.01821400
F	-1.02661700	1.56425800	0.80677800
F	-1.66007600	1.04972100	-1.23069600
C	-0.48023900	-0.59034900	0.00999300
F	-3.56554600	1.63767000	0.31423500
F	-1.69962800	-1.62467500	0.86988000
F	0.26535300	-0.75094900	1.12758400
P	2.23627400	0.28029200	-0.47132800

O	2.71629800	-1.12867500	-0.48268700
K	1.02441500	3.01246300	-0.13835500
O	0.67376000	0.42593100	-0.74475100
K	2.72868400	-1.31456200	2.04508500
O	2.58714900	1.10486200	0.74639600
O	2.79626400	1.15244600	-1.72826900
H	2.64708300	0.67908600	-2.54952700

TS7

C	3.04280000	0.08487900	-0.28962300
F	3.25130900	-0.34815900	-1.51792600
F	3.50377100	-0.76660400	0.60058700
C	0.05197000	-1.40067100	1.19522600
F	-0.79227900	-0.88385800	2.06874400
F	1.15627100	-1.67680700	1.86892600
F	-0.51469500	-2.55856600	0.78227600
C	1.55707500	0.50757100	-0.03685600
F	1.32786200	1.43999400	-1.00583300
F	1.57251400	1.13908600	1.15133500
C	0.36957800	-0.50532200	-0.06656600
F	3.76014900	1.22262800	-0.15383900
F	1.45789100	-1.82684500	-0.52549100
F	-0.21950300	-0.70051400	-1.26133600
P	-2.33230100	0.77584400	0.08637500
O	-2.83568000	-0.54250700	-0.39499500
K	-2.04420700	-2.59949100	-1.41939200
O	-2.68615300	2.04538500	-0.61615900
K	-0.57388000	3.33699800	-0.30983100
O	-0.75818900	0.79831200	0.31751000
F	-2.85442300	0.93980300	1.58096300

TS8

C	-4.39256200	1.45099100	-0.43114700
F	-4.60907900	1.94764800	0.77000300
F	-5.24524500	0.49276100	-0.72206600
C	-2.39331500	-1.56928600	-0.24787700
F	-1.47408500	-2.07733800	-1.13925900
F	-3.54456200	-1.76515700	-0.84942000
F	-2.26657700	-2.33696300	0.82373900
C	-2.89511400	1.07149700	-0.68037000
F	-2.21960500	2.21597900	-0.45561000
F	-2.81862800	0.77340800	-1.99446000
C	-2.11286700	-0.05097300	0.07804900
F	-4.63271900	2.45524900	-1.30820400
F	-3.74798500	-0.39506300	1.11630200
F	-1.65189600	0.28153500	1.30151200
P	2.72655000	0.96231400	1.35032100
O	3.42131400	2.20171300	0.94286900
O	3.30811800	-0.32081000	0.52781600
P	3.21789600	-0.68251600	-1.05473800
O	3.91430600	0.33313700	-1.87151100
O	1.56564900	-0.44483900	-1.30546800
P	0.59799700	-0.22400400	-0.07795300
O	-0.73584300	0.20549300	-0.71368100
O	0.53249400	-1.41728600	0.80781300
K	1.21069700	-3.40241000	-0.79390500
O	1.19348800	1.03780000	0.66874700
O	3.42230500	-2.13942600	-1.19271100
K	4.36349600	2.73423100	-1.31314700
O	2.53178600	0.55162800	2.75572300
K	0.17796500	-0.41803600	3.24305300

TS9

C	2.84881400	-0.38882600	-0.01344800
F	3.13016900	-0.74979900	1.22287700
F	3.31262900	0.80950100	-0.28595100
C	-0.20584800	1.68202800	-0.10424900
F	-1.19567800	1.76463400	-1.03982000
F	0.82672500	2.27415800	-0.67878200
F	-0.63802500	2.38254100	0.92575000
C	1.33522100	-0.56134100	-0.37355900
F	1.12319600	-1.91606300	-0.10109000
F	1.25424600	-0.39712200	-1.69124100
C	0.15852000	0.20414600	0.31809500
F	3.49654100	-1.25374700	-0.82483100
F	1.38835200	1.05289900	1.34197100
F	-0.27806200	-0.32624800	1.50388700
P	-2.53614900	-0.71615000	-0.16849600
O	-3.38446900	-0.56388700	-1.39206500
O	-2.86320600	0.45209500	0.79582700
O	-0.97731400	-0.62607500	-0.60257800
O	-2.52838300	-2.00809800	0.67743600
Li	-2.47143400	-0.70256800	2.19839300
Li	-0.79057500	-2.45401500	0.07173900
Li	-3.58195500	1.26562000	-0.80930000

TS10

C	-0.26909900	-0.49319900	0.09036800
F	-0.55431900	-2.08555400	-0.69583200
F	0.10485600	-0.11973700	-1.16934600
P	1.66076500	1.57821900	0.19668500
O	0.28995200	1.04563400	0.68942300
K	2.10245900	-1.50555100	-1.90320100
O	1.41256200	2.75604400	-0.84073500
H	0.68799000	3.32643400	-0.57656700
O	2.58982900	0.59088700	-0.39220400
O	2.23909400	2.37398400	1.45171600
H	2.13898600	1.87045700	2.26208700
C	-1.77714400	-0.21274300	0.42535600
F	-2.52044500	-1.31549000	0.47468500
C	0.52359500	-1.37249900	1.15739200
F	1.55159500	-2.03642300	0.59686700
F	1.06237700	-0.68269000	2.16376400
F	-0.29366900	-2.24251600	1.71897900
F	-1.87041600	0.36707400	1.63400800
C	-2.47920100	0.72065700	-0.61155200
F	-2.53426300	0.13895300	-1.80247300
F	-1.88094600	1.89984100	-0.74111900
F	-3.72316700	0.93760900	-0.19175200

TS11

C	1.53387400	-1.72134700	-0.03397700
F	1.53020200	-1.84416300	1.28519400
F	2.75113800	-1.60928900	-0.50256000
C	0.56394600	2.15495000	0.11900400
F	-0.55499200	2.91206000	-0.01852700
F	1.24006300	2.32996000	-1.00083400
F	1.19571700	2.67105300	1.15069800
C	0.56020800	-0.57850000	-0.54052400
F	-0.62479600	-1.30084700	-0.80150000

F	0.94357700	-0.15778500	-1.73766400
C	0.19254700	0.63661900	0.39558500
F	1.06479400	-2.90005800	-0.51407000
F	2.12658800	0.53907300	0.47055800
F	0.14182200	0.34410400	1.68661200
C	-2.06563400	0.89147400	-0.88638500
O	-1.44458000	0.67113400	0.24717300
H	-1.49806600	1.38856300	-1.67987000
O	-3.21590300	0.53427700	-1.03449300
K	-2.85551200	-1.48357800	0.71026500

TS12

C	-1.99171100	-0.84406500	-0.09973000
F	-2.02376200	-1.38251500	-1.30553500
F	-2.76064400	0.22204300	-0.03641300
C	0.34746300	1.98922200	0.07640300
F	1.00853400	2.37208000	1.18736000
F	-0.90144700	2.38508600	0.27735600
F	0.88911000	2.66243800	-0.92725200
C	-0.53607100	-0.58651900	0.41277100
F	0.05269300	-1.82124600	0.30550900
F	-0.67838600	-0.29622000	1.71827500
C	0.43058600	0.44995600	-0.23821500
F	-2.51576700	-1.75959500	0.74361700
F	-0.71349500	0.76627800	-1.49436500
F	1.27193700	0.00544400	-1.21710800
O	1.70975700	0.05041400	0.90978400
H	1.55698300	0.48850000	1.74352500
K	2.76865300	-1.85934400	-0.16800800

TS13

C	-3.42731100	0.22170800	0.07290000
F	-3.77936000	0.16664100	1.34490300
F	-3.93222300	-0.79513700	-0.59853500
C	-0.51702200	-1.92653100	-0.62319800
F	0.42398300	-1.73130600	-1.57298100
F	-1.58544200	-2.31734000	-1.31216100
F	-0.07045800	-2.92944600	0.12337500
C	-1.88537900	0.40023100	-0.12555300
F	-1.60713600	1.53727400	0.54098200
F	-1.75508500	0.63815900	-1.45650000
C	-0.86355000	-0.70201000	0.30778600
F	-3.99203400	1.34026300	-0.43497000
F	-2.09760000	-1.68209200	0.94944600
F	-0.33999200	-0.62407000	1.54772500
P	1.95131300	0.46161400	0.18560300
O	2.69150500	1.00892900	-1.04097600
O	2.44414700	-0.94476700	0.57198300
O	0.44363400	0.35927200	-0.34792400
O	1.97462100	1.37831300	1.41346100
K	1.91173300	-0.58094800	3.02467800
K	0.66879800	2.60860000	-1.61098900
K	3.24983700	-1.39210100	-1.75472700
O	0.86235000	3.69658200	0.80923200
H	1.31969800	2.86563000	1.11993800
H	0.08124400	3.76116900	1.35636700

TS14

C	-3.68181400	0.21565200	-0.25818300
F	-4.16790500	0.13443200	0.96764600
F	-4.09273400	-0.79461600	-0.99650600
C	-0.69088400	-1.86693700	-0.67657600
F	0.34904300	-1.65561600	-1.50871600
F	-1.68082300	-2.22855400	-1.48650000
F	-0.33633200	-2.89190700	0.08943900
C	-2.13027300	0.42612600	-0.29238900
F	-1.96330400	1.55154800	0.46356700
F	-1.85834200	0.72724800	-1.57137600
C	-1.12627500	-0.65230300	0.22834000
F	-4.20761900	1.33618900	-0.80381100
F	-2.42478300	-1.66926300	0.73349900
F	-0.75807700	-0.58054400	1.52325900
P	1.67559100	0.41228500	0.40719000
O	2.62980400	0.81828300	-0.73654400
O	2.00775300	-0.99610600	0.91066300
O	0.22584900	0.43084700	-0.26613900
O	1.63631300	1.42607200	1.55444900
K	1.30303600	-0.45780500	3.26843700
K	0.23021800	3.10739500	0.11872000
K	3.12872700	-1.81415400	-1.21910600
O	5.09059500	-0.03682600	-1.06049700
H	5.83722800	0.43129800	-0.69472900
H	4.28146800	0.48681200	-0.83692400
O	1.93224700	3.08107000	-1.91175500
H	1.76999300	2.92748700	-2.84047100
H	2.26269000	2.21464200	-1.55177900

TS15

C	-3.66318700	0.04370500	-0.34453900
F	-4.13982800	0.49173300	0.80244400
F	-4.09618600	-1.17545200	-0.59413900
C	-0.69665800	-2.07240800	0.15978600
F	0.35930800	-2.23643700	-0.67475600
F	-1.67842900	-2.73582400	-0.43879900
F	-0.35135100	-2.68263100	1.28572900
C	-2.10999000	0.19605400	-0.45617400
F	-1.90413700	1.51739200	-0.28187500
F	-1.84620600	-0.10996700	-1.75606700
C	-1.11513400	-0.58495400	0.46602300
F	-4.17257900	0.84112300	-1.31067400
F	-2.43786000	-1.29622700	1.33650200
F	-0.74638600	-0.00253300	1.62014900
P	1.69271800	0.47214300	0.11759000
O	2.54164000	0.29313700	-1.15264200
O	2.06816100	-0.51540300	1.22008600
O	0.21706200	0.17172300	-0.41636200
O	1.71340700	1.91551500	0.64618800
K	1.34143800	1.02303500	3.15906600
K	0.64988000	1.21003900	-2.74673200
K	3.16509600	-2.24948900	-0.26163700
O	5.00165700	-0.59306800	-1.25499900
H	5.73022100	-0.00617500	-1.06511300
H	4.17817400	-0.04225500	-1.23648700
O	1.20727800	3.46980200	-1.48848300
H	0.73825000	4.24512500	-1.18538700
H	1.47901900	2.98847900	-0.66720600
O	-0.25771000	2.96916000	2.12462300
H	-1.13120400	2.69646000	1.84395500
H	0.34362600	2.66622100	1.40866500

TS16

C	-2.28259600	-0.79571900	-0.09302500
F	-2.32633100	-1.35182600	-1.28877200
F	-3.02817400	0.28607100	-0.04046600
C	0.10631400	1.98346000	0.05004000
F	0.79688900	2.38441700	1.14221700
F	-1.13231300	2.39157500	0.26841800
F	0.64083800	2.63025000	-0.97364100
C	-0.82249900	-0.56891800	0.42095700
F	-0.25988400	-1.81546600	0.33275300
F	-0.96036800	-0.26439600	1.72814000
C	0.18827600	0.43548800	-0.21343300
F	-2.82469500	-1.68929400	0.76456200
F	-0.98309300	0.76228800	-1.50919800
F	1.00638400	-0.02443800	-1.19568000
O	1.42494100	0.04792800	0.92058200
H	1.20575000	0.40710600	1.77927300
K	2.44690400	-2.04023800	-0.21485800
O	3.97879400	0.07863600	0.09724100
H	4.44797400	0.80835400	-0.30294400
H	3.12497700	0.42806200	0.42115800

TS17

C	-2.60877500	-0.68070000	-0.26000600
F	-2.56381200	-1.29798800	-1.42459200
F	-3.23512600	0.47327200	-0.35544600
C	-0.02403400	1.86663800	0.06936000
F	0.53513800	2.27598800	1.23557200
F	-1.23179100	2.39426600	0.07699700
F	0.72163400	2.40728400	-0.88813100
C	-1.20746100	-0.57531600	0.42725200
F	-0.76667700	-1.85036300	0.48492300
F	-1.47845900	-0.15935900	1.69232700
C	-0.03415700	0.30378400	-0.10432100
F	-3.34581900	-1.46037400	0.56064700
F	-0.98867400	0.66707100	-1.58108500
F	0.85903800	-0.26139500	-0.95690800
O	1.01632600	-0.10157800	1.18603000
H	0.63547700	0.18648200	2.01688700
K	3.34965500	-1.02760000	-0.93639400
O	2.21140300	-2.50909600	0.88773900
H	1.66374500	-3.29067400	0.93968500
H	1.63057200	-1.75916700	1.13557800
O	3.41893300	1.03918000	0.64146500
H	2.53782200	0.78335900	0.98692400
H	3.41313100	1.99430600	0.59810700

13. References

1. Leung, S. C. E., Wanninayake, D., Chen, D., Nguyen, N.-T. & Li, Q. Physicochemical properties and interactions of perfluoroalkyl substances (PFAS) - Challenges and opportunities in sensing and remediation. *Science of The Total Environment* **905**, 166764 (2023).
2. Meegoda, J. N., Bezerra de Souza, B., Casarini, M. M. & Kewalramani, J. A. A Review of PFAS Destruction Technologies. *International Journal of Environmental Research and Public Health* **19**, 16397 (2022).
3. Merino, N. *et al.* Degradation and Removal Methods for Perfluoroalkyl and Polyfluoroalkyl Substances in Water. *Environmental Engineering Science* **33**, 615–649 (2016).
4. Verma, S., Lee, T., Sahle-Demessie, E., Ateia, M. & Nadagouda, M. N. Recent advances on PFAS degradation via thermal and nonthermal methods. *Chemical Engineering Journal Advances* **13**, 100421 (2023).
5. Glüge, J. *et al.* An overview of the uses of per- and polyfluoroalkyl substances (PFAS). *Environmental Science: Processes & Impacts* **22**, 2345–2373 (2020).
6. Hoskins, T. D. *et al.* Chronic Exposure to a PFAS Mixture Resembling AFFF-Impacted Surface Water Decreases Body Size in Northern Leopard Frogs (Rana pipiens). *Environmental Science & Technology* **57**, 14797–14806 (2023).
7. Mikkonen, A. T. *et al.* Spatio-temporal trends in livestock exposure to per- and polyfluoroalkyl substances (PFAS) inform risk assessment and management measures. *Environmental Research* **225**, 115518 (2023).
8. Ateia, M., Alsbaiee, A., Karanfil, T. & Dichtel, W. Efficient PFAS Removal by Amine-Functionalized Sorbents: Critical Review of the Current Literature. *Environmental Science & Technology Letters* **6**, 688–695 (2019).
9. Ilango, A. K. *et al.* Enhanced Adsorption of Mixtures of Per- and Polyfluoroalkyl Substances in Water by Chemically Modified Activated Carbon. *ACS ES&T Water* **3**, 3708–3715 (2023).
10. Cui, J., Gao, P. & Deng, Y. Destruction of Per- and Polyfluoroalkyl Substances (PFAS) with Advanced Reduction Processes (ARPs): A Critical Review. *Environmental Science & Technology* **54**, 3752–3766 (2020).
11. Khan, Q. *et al.* Advanced oxidation/reduction processes (AO/RPs) for wastewater treatment, current challenges, and future perspectives: a review. *Environmental Science and Pollution Research* **31**, 1863–1889 (2024).
12. Zhang, H., Chen, J.-X., Qu, J.-P. & Kang, Y.-B. Photocatalytic low-temperature defluorination of PFASs. *Nature* **635**, 610–617 (2024).
13. Liu, X. et al. Photocatalytic C–F bond activation in small molecules and polyfluoroalkyl substances. *Nature* https://doi.org/10.1038/s41586-024-08327-7 (2024).
14. Zhang, K. *et al.* Destruction of Perfluorooctane Sulfonate (PFOS) and Perfluorooctanoic Acid (PFOA) by Ball Milling. *Environmental Science & Technology* **47**, 6471–6477 (2013).
15. Zhang, Q., Lu, J., Saito, F. & Baron, M. Mechanochemical solid-phase reaction between polyvinylidene fluoride and sodium hydroxide. *Journal of Applied Polymer Science* **81**, 2249–2252 (2001).

16. Ateia, M., Skala, L. P., Yang, A. & Dichtel, W. R. Product analysis and insight into the mechanochemical destruction of anionic PFAS with potassium hydroxide. *Journal of Hazardous Materials Advances* **3**, 100014 (2021).
17. Yang, N. *et al.* Solvent-Free Nonthermal Destruction of PFAS Chemicals and PFAS in Sediment by Piezoelectric Ball Milling. *Environmental Science & Technology Letters* **10**, 198–203 (2023).
18. Cagnetta, G. *et al.* Mechanochemical destruction of perfluorinated pollutants and mechanosynthesis of lanthanum oxyfluoride: A Waste-to-Materials process. *Chemical Engineering Journal* **316**, 1078–1090 (2017).
19. Turner, L. P. *et al.* Mechanochemical remediation of perfluorooctanesulfonic acid (PFOS) and perfluorooctanoic acid (PFOA) amended sand and aqueous film-forming foam (AFFF) impacted soil by planetary ball milling. *Science of The Total Environment* **765**, 142722 (2021).
20. Battye, N. J. *et al*. Use of a horizontal ball mill to remediate per- and polyfluoroalkyl substances in soil. Science of *The Total Environment* **835**, 155506 (2022).
21. Turner, L. P., Kueper, B. H., Patch, D. J., Weber, K. P. Elucidating the relationship between PFOA and PFOS destruction, particle size and electron generation in amended media commonly found in soils. *Science of The Total Environment* **888**, 164188 (2023).
22. Battye, N. et al. Mechanochemical degradation of per- and polyfluoroalkyl substances in soil using an industrial-scale horizontal ball mill with comparisons of key operational metrics. *Science of The Total Environment* **928**, 172274 (2024).
23. Gobindlal, K., Shields, E., Whitehill, A., Weber, C. C. & Sperry, J. Mechanochemical destruction of per- and polyfluoroalkyl substances in aqueous film-forming foams and contaminated soil. *Environmental Science: Advances* **2**, 982–989 (2023).
24. Gobindlal, K., Zujovic, Z., Jaine, J., Weber, C. C. & Sperry, J. Solvent-Free, Ambient Temperature and Pressure Destruction of Perfluorosulfonic Acids under Mechanochemical Conditions: Degradation Intermediates and Fluorine Fate. *Environmental Science & Technology* **57**, 277–285 (2023).
25. Trang, B. *et al.* Low-temperature mineralization of perfluorocarboxylic acids. *Science* **377**, 839–845 (2022).
26. Monsky, R. J., Li, Y., Houk, K. N. & Dichtel, W. R. Low-Temperature Mineralization of Fluorotelomers with Diverse Polar Head Groups. *Journal of the American Chemical Society* **146**, 17150–17157 (2024).
27. United States Environmental Protection Agency, "Per- and Polyfluoroalkyl Substances (PFAS): Incineration to Manage PFAS Waste Streams", https://www.epa.gov/sites/default/files/2019-09/documents/technical_brief_pfas_incineration_ioaa_approved_final_july_2019.pdf (2020).
28. Fang, J. *et al.* Treatment of per- and polyfluoroalkyl substances (PFAS): A review of transformation technologies and mechanisms. *Journal of Environmental Chemical Engineering* **12**, 111833 (2024).
29. Berhanu, A. *et al.* A review of microbial degradation of per- and polyfluoroalkyl substances (PFAS): Biotransformation routes and enzymes. *Science of The Total Environment* **859**, 160010 (2023).
30. Harsanyi, A. & Sandford, G. Organofluorine chemistry: applications, sources and sustainability. *Green Chemistry* **17**, 2081–2086 (2015).
31. Patel, C. *et al.* Fluorochemicals from fluorspar via a phosphate-enabled mechanochemical process that bypasses HF. *Science* **381**, 302–306 (2023).

32. Lee, J., Zhang, Q. & Saito, F. Synthesis of nano-sized lanthanum oxyfluoride powders by mechanochemical processing. *Journal of Alloys and Compounds* **348**, 214–219 (2003).
33. Qu, J., He, X., Zhang, Q., Liu, X. & Saito, F. Decomposition pathways of polytetrafluoroethylene by co-grinding with strontium/calcium oxides. *Environmental Technology* **38**, 1421–1427 (2017).
34. Sheldon, D. J., Parr, J. M. & Crimmin, M. R. Room Temperature Defluorination of Poly(tetrafluoroethylene) by a Magnesium Reagent. *Journal of the American Chemical Society* **145**, 10486–10490 (2023).
35. Améduri, B. & Hori, H. Recycling and the end of life assessment of fluoropolymers: recent developments, challenges and future trends. *Chemical Society Reviews* **52**, 4208–4247 (2023).
36. Saravanan, M., Ganesan, M. & Ambalavanan, S. An in situ generated carbon as integrated conductive additive for hierarchical negative plate of lead-acid battery. *Journal of Power Sources* **251**, 20–29 (2014).
37. Frisch, M. J. *et al.* Gaussian 16, Revision C.01, Gaussian, Inc., Wallingford CT (2016).
38. Domingo, L. R., Chamorro, E. & Pérez, P. Understanding the Reactivity of Captodative Ethylenes in Polar Cycloaddition Reactions. A Theoretical Study. *The Journal of Organic Chemistry* **73**, 4615–4624 (2008).
39. Domingo, L. R. & Pérez, P. The nucleophilicity N index in organic chemistry. *Organic & Biomolecular Chemistry* **9**, 7168–7175 (2011).
40. Parr, R. G., Szentpály, L. V. & Liu, S. Electrophilicity Index. *Journal of the American Chemical Society* **121**, 1922–1924 (1999).
41. Chattaraj, P. K. & Maiti, B. Reactivity Dynamics in Atom−Field Interactions: A Quantum Fluid Density Functional Study. *The Journal of Physical Chemistry A* **105**, 169–183 (2001).
42. Dixon, D. A., Smart, B. E., Krusic, P. J. & Matsuzawa, N. Bond energies in organofluorine systems: applications to Teflon® and fullerenes. *Journal of Fluorine Chemistry* **72**, 209–214 (1995).
43. Giannetti, E. Thermal stability and bond dissociation energy of fluorinated polymers: A critical evaluation. *Journal of Fluorine Chemistry* **126**, 623–630 (2005).
44. Wu, E. C. & Rodgers, A. S. Kinetics of the gas phase reaction of pentafluoroethyl iodide with hydrogen iodide. Enthalpy of formation of the pentafluoroethyl radical and the π bond dissociation energy in tetrafluoroethylene. *Journal of the American Chemical Society* **98**, 6112–6115 (1976).
45. Iashin, V., Wirtanen, T. & Perea-Buceta, J. E. Tetramethylammonium Fluoride: Fundamental Properties and Applications in C-F Bond-Forming Reactions and as a Base. *Catalysts* **12**, 233 (2022).
46. Morales-Colón, M. T. *et al.* Tetramethylammonium Fluoride Alcohol Adducts for S_NAr Fluorination. *Organic Letters* **23**, 4493–4498 (2021).
47. Kim, D. W., Jeong, H.-J., Lim, S. T. & Sohn, M.-H. Tetrabutylammonium Tetra(tert-Butyl Alcohol)-Coordinated Fluoride as a Facile Fluoride Source. *Angewandte Chemie International Edition* **47**, 8404–8406 (2008).
48. Ye, Y., Schimler, S. D., Hanley, P. S. & Sanford, M. S. $Cu(OTf)_2$-Mediated Fluorination of Aryltrifluoroborates with Potassium Fluoride. *Journal of the American Chemical Society* **135**, 16292–16295 (2013).
49. Zarganes-Tzitzikas, T., Neochoritis, C. G. & Dömling, A. Atorvastatin (Lipitor) by MCR. *ACS Medicinal Chemistry Letters* **10**, 389–392 (2019).
50. Jupp, A. R., Beijer, S., Narain, G. C., Schipperc, W. & Slootweg, J. C. Phosphorus recovery and recycling – closing the loop. *Chemical Society Reviews* **50**, 87–101 (2021).

51. Brouwer, D. H. & Horvath, M. Minimizing the effects of RF inhomogeneity and phase transients allows resolution of two peaks in the ^{1}H CRAMPS NMR spectrum of adamantane. *Solid State Nuclear Magnetic Resonance* **71**, 30−40 (2015).

52. Griffin, J. M., Yates, J. R., Berry, A. J., Wimperis, S. & Ashbrook, S. E. High-Resolution 19F MAS NMR Spectroscopy: Structural Disorder and Unusual J Couplings in a Fluorinated Hydroxy-Silicate. *Journal of the American Chemical Society* **132**, 15651−15660 (2010).

53. DeVience, S. J., Walsworth, R. L. & Rosen, M. S. NMR of ^{31}P nuclear spin singlet states in organic diphosphates. *Journal of Magnetic Resonance* **333**, 107101 (2021).

54. Earl, W. L. & Vanderhart, D. L. Measurement of ^{13}C chemical shifts in solids. *Journal of Magnetic Resonance (1969)* **48**, 35−54 (1982).

55. Robinson, M. T. The Crystal Structures of β-K_2SO_4 and β-K_2PO_3F. *The Journal of Physical Chemistry* **62**, 925−928 (1958).

56. Miller, J. M. Fluorine-19 magic-angle spinning NMR. *Progress in Nuclear Magnetic Resonance Spectroscopy* **28**, 255−281 (1996).

57. Hayashi, S. & Hayamizu, K. High-Resolution Solid-State ^{31}P NMR of Alkali Phosphates. *Bulletin of the Chemical Society of Japan* **62**, 3061−3068 (1989).

58. Freias, J. C., Emmerich, F. G., Cernicchiaro, G. R., Sampaio, L. C. & Bonagamba, T. J. Magnetic susceptibility effects on ^{13}C MAS NMR spectra of carbon materials and graphite. *Solid State Nuclear Magnetic Resonance* **20**, 61−73 (2001).

59. Van der Hart, D. L., Earl, W. L. & Garroway, A. N. Resolution in ^{13}C NMR of organic solids using high-power proton decoupling and magic-angle sample spinning. *Journal of Magnetic Resonance* **44**, 361−401 (1981).

60. Lennox, A. J. J. & Lloyd-Jones, G. C. Preparation of Organotrifluoroborate Salts: Precipitation-Driven Equilibrium under Non-Etching Conditions. *Angewandte Chemie International Edition* **51**, 9385−9388 (2012).

61. Yang, Q. *et al.* Potential Explosion Hazards Associated with the Autocatalytic Thermal Decomposition of Dimethyl Sulfoxide and Its Mixtures. *Organic Process Research & Development* **24**, 916−939 (2020).

62. Tang, P., Wang, W. & Ritter, T. Deoxyfluorination of Phenols. *Journal of the American Chemical Society* **133**, 11482−11484 (2011).

63. Cohen, D. T. & Buchwald, S. L. Mild Palladium-Catalyzed Cyanation of (Hetero)aryl Halides and Triflates in Aqueous Media. *Organic Letters* **17**, 202−205 (2015).

64. Zhang, G. *et al.* l-Proline: an efficient N,O-bidentate ligand for copper-catalyzed aerobic oxidation of primary and secondary benzylic alcohols at room temperature. *Chemical Communications* **49**, 7908−7910 (2013).

65. Hartmer, M. F. & Waldvogel, S. R. Electroorganic synthesis of nitriles via a halogen-free domino oxidation–reduction sequence. *Chemical Communications* **51**, 16346−16348 (2015).

66. Purrington, S. T. & Woodard, D. L. Preparation of α-fluorocarboxylic acids and derivatives. *The Journal of Organic Chemistry* **55**, 3423−3424 (1990).

67. Appel, R. & Montenarh, M. Additionsreaktionen von N,N-Dialkylsulfamiden, tertiären Aminen und Phosphinen mit Fluorsulfonylisocyanat. *Chemische Berichte* **110**, 2368−2373 (1977).
68. Domalski, E. S. & Armstrong, G. T. The Heats of Combustion of Polytetrafluoroethylene (Teflon) and Graphite in Elemental Fluorine. *Journal of Research of the National Bureau of Standards – A. Physics and Chemistry* **71a**, 105118 (1967).
69. Reed, J. J., The NBS Tables of Chemical Thermodynamic Properties: Selected Values for Inorganic and C1 and C2 Organic Substances in SI Units, National Institute of Standards and Technology, https://doi.org/10.18434/M32124 (1989).
70. Luff, B. B. & Reed, R. B. Standard enthalpy of formation of tetrapotassium pyrophosphate. *Journal of Chemical & Engineering Data* **24**, 227−228 (1979).
71. Chase Jr., M.W. NIST-JANAF Themochemical Tables. 4th Edition. *Journal of Physical and Chemical Reference Data*, Monograph 9, 1–1951 (1998).
72. Jain, A. *et al.* Commentary: The Materials Project: A materials genome approach to accelerating materials innovation. *APL Materials* **1**, 011002 (2013).
73. Jain, A. *et al.* Formation enthalpies by mixing GGA and GGA + U calculations. *Physical Review B* **84**, 045115 (2011).
74. Saal, J. E., Kirklin, S., Aykol, M., Meredig, B. & Wolverton, C. Materials Design and Discovery with High-Throughput Density Functional Theory: The Open Quantum Materials Database (OQMD). *JOM* **65**, 1501-1509 (2013).
75. Kirklin, S. *et al.* The Open Quantum Materials Database (OQMD): assessing the accuracy of DFT formation energies. *npj Computational Materials* **1**, 15010 (2015).
76. Chai, J.-D. & Head-Gordon, M. Long-range corrected hybrid density functionals with damped atom–atom dispersion corrections. *Physical Chemistry Chemical Physics* **10**, 6615−6620 (2008).
77. Legault, C. Y. "CYLview20: Visualization and analysis software for computational chemistry" (CYLview, 2020); http://www.cylview.org
78. Luchini, G., Alegre-Requena, J. V., Funes-Ardoiz, I., Paton, R. S. GoodVibes: automate thermochemistry for heterogeneous computational chemistry data. *F1000Research*. **9**, 291 (2020).
79. Zhan, C.-G., Nichols, J. A. & Dixon, D. A. Ionization Potential, Electron Affinity, Electronegativity, Hardness, and Electron Excitation Energy: Molecular Properties from Density Functional Theory Orbital Energies. *The Journal of Physical Chemistry A* **107**, 4184−4195 (2003).
80. Parr, R. G. & Pearson, R. G. Absolute hardness: companion parameter to absolute electronegativity. *Journal of the American Chemical Society* **105**, 7512−7516 (1983).
81. Gázquez, J. L., Cedillo, A. & Vela, A. Electrodonating and Electroaccepting Powers. *The Journal of Physical Chemistry A* **111**, 1966−1970 (2007).

14. PFAS-mix NMR characterization

n(OTf⁻)	m (sample)	Yield of F⁻ %	Yield of PO_3F^{2-} %
0.053 mmol	48.8 mg	80%	23%

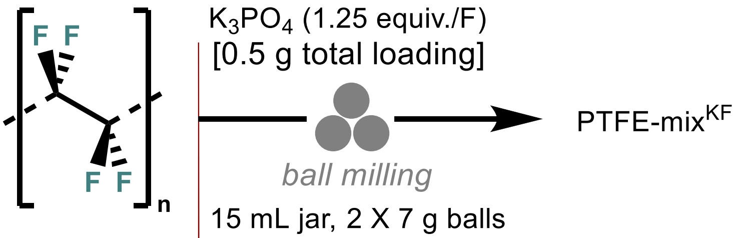

1, PTFE (1 eq)

^{19}F NMR (377 MHz, 90% H_2O + 10% D_2O)

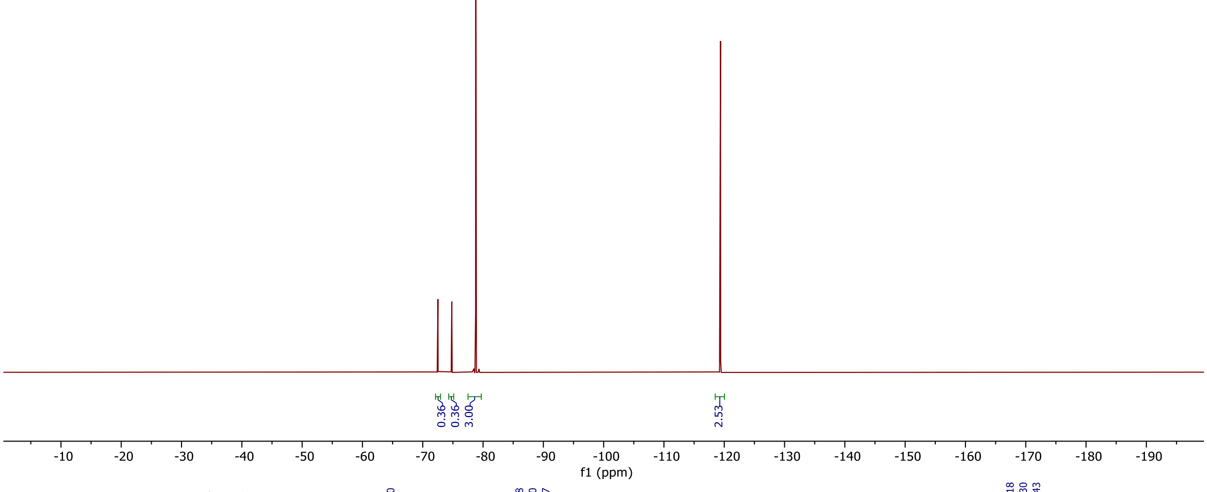

^{31}P NMR (162 MHz, 90% H_2O + 10% D_2O)

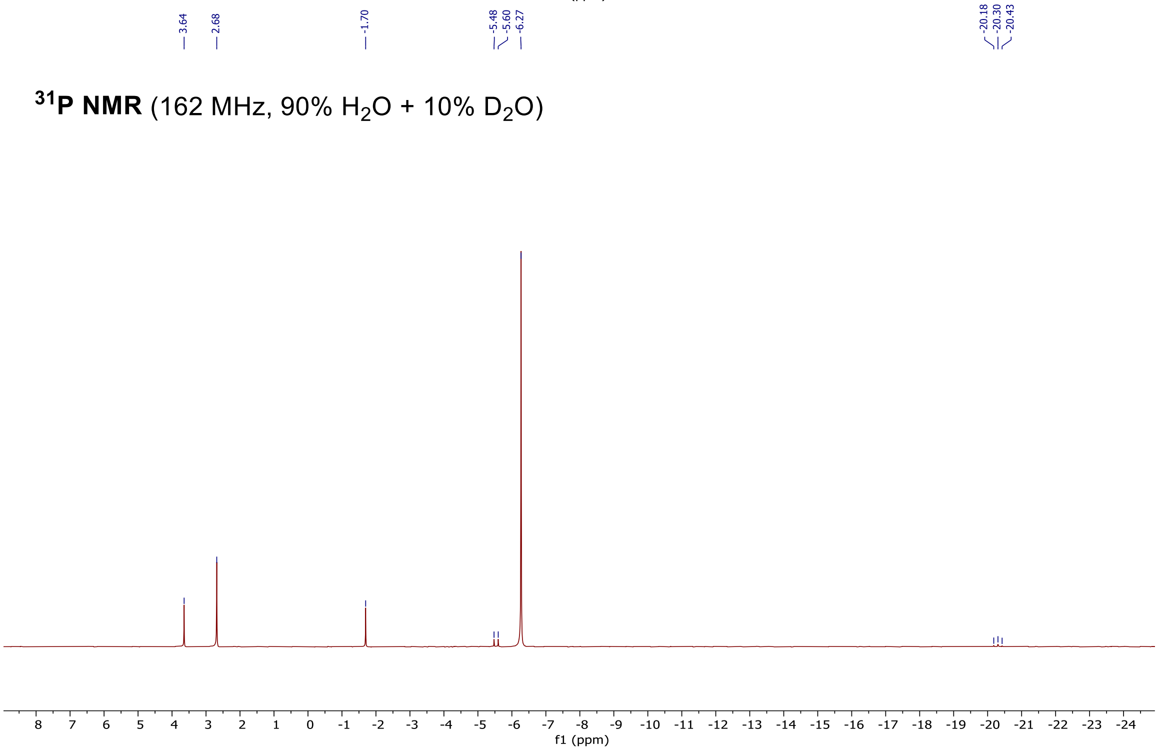

n(OTf⁻)	m (sample)	Yield of F⁻ %	Yield of PO_3F^{2-} %
0.069 mmol	50.1 mg	4%	85%

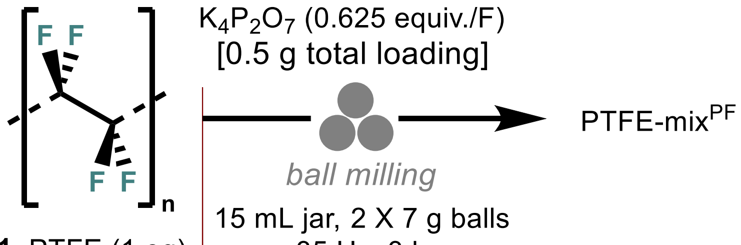

1, PTFE (1 eq)

^{19}F NMR (377 MHz, 90% H_2O + 10% D_2O)

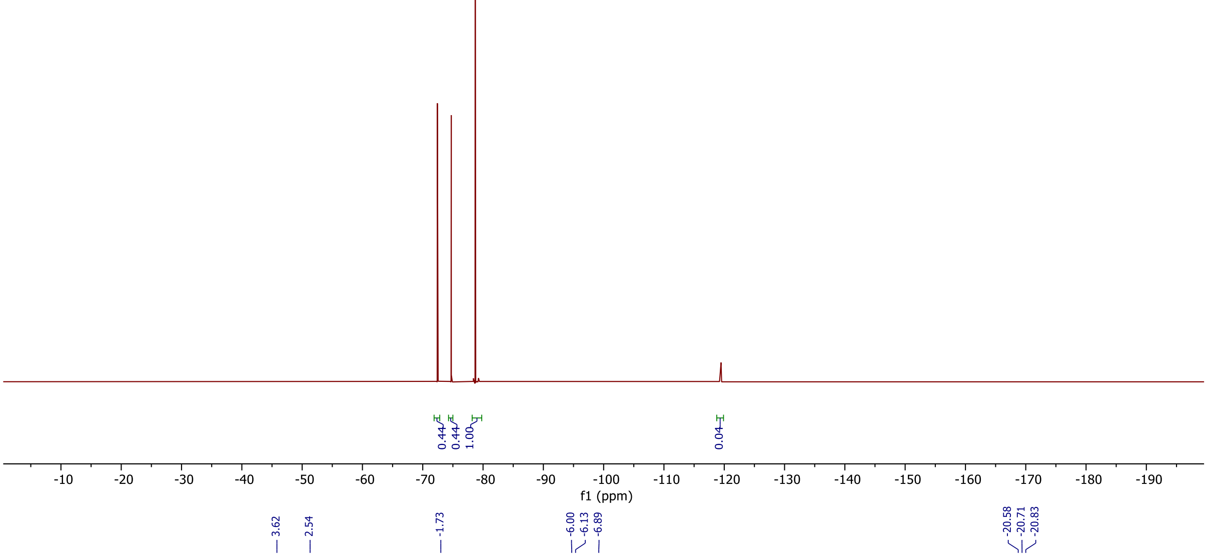

^{31}P NMR (162 MHz, 90% H_2O + 10% D_2O)

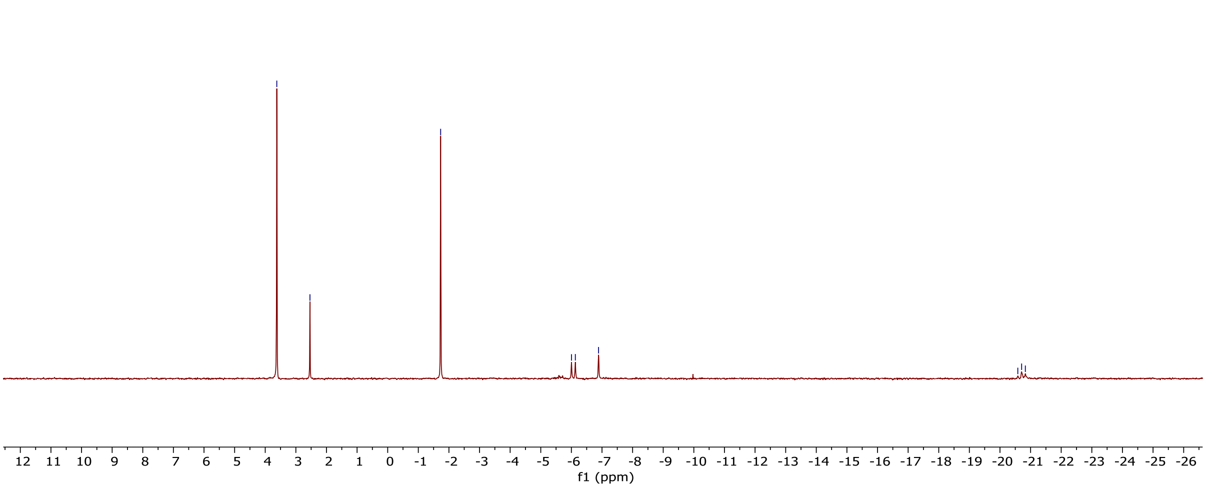

n(OTf⁻)	m (sample)	Yield of F⁻ %	Yield of PO_3F^{2-} %
0.058 mmol	37.2 mg	74%	19%

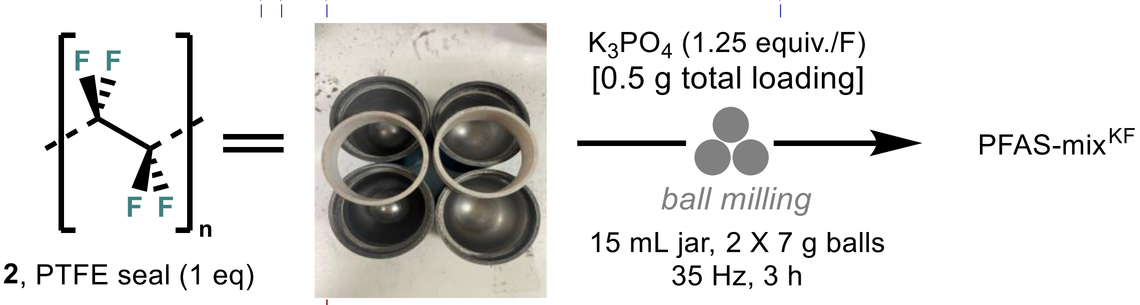

^{19}F NMR (471 MHz, 90% H_2O + 10% D_2O)

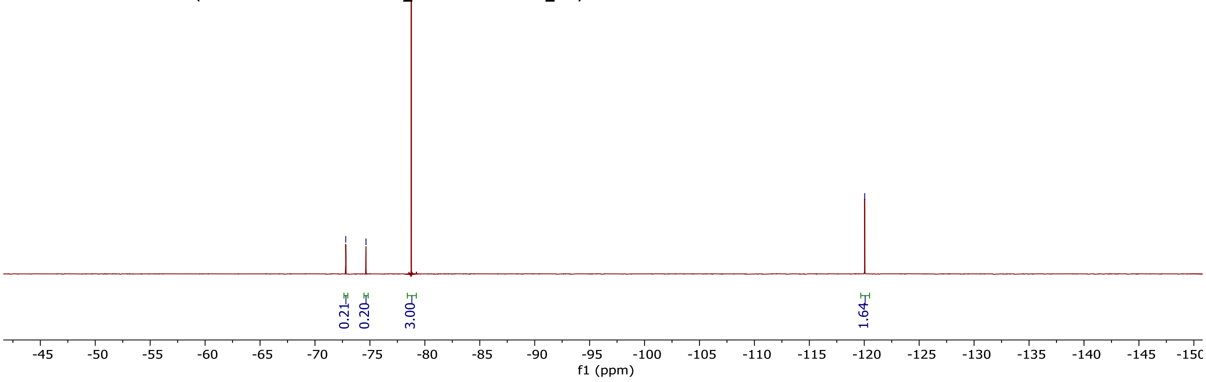

^{31}P NMR (203 MHz, 90% H_2O + 10% D_2O)

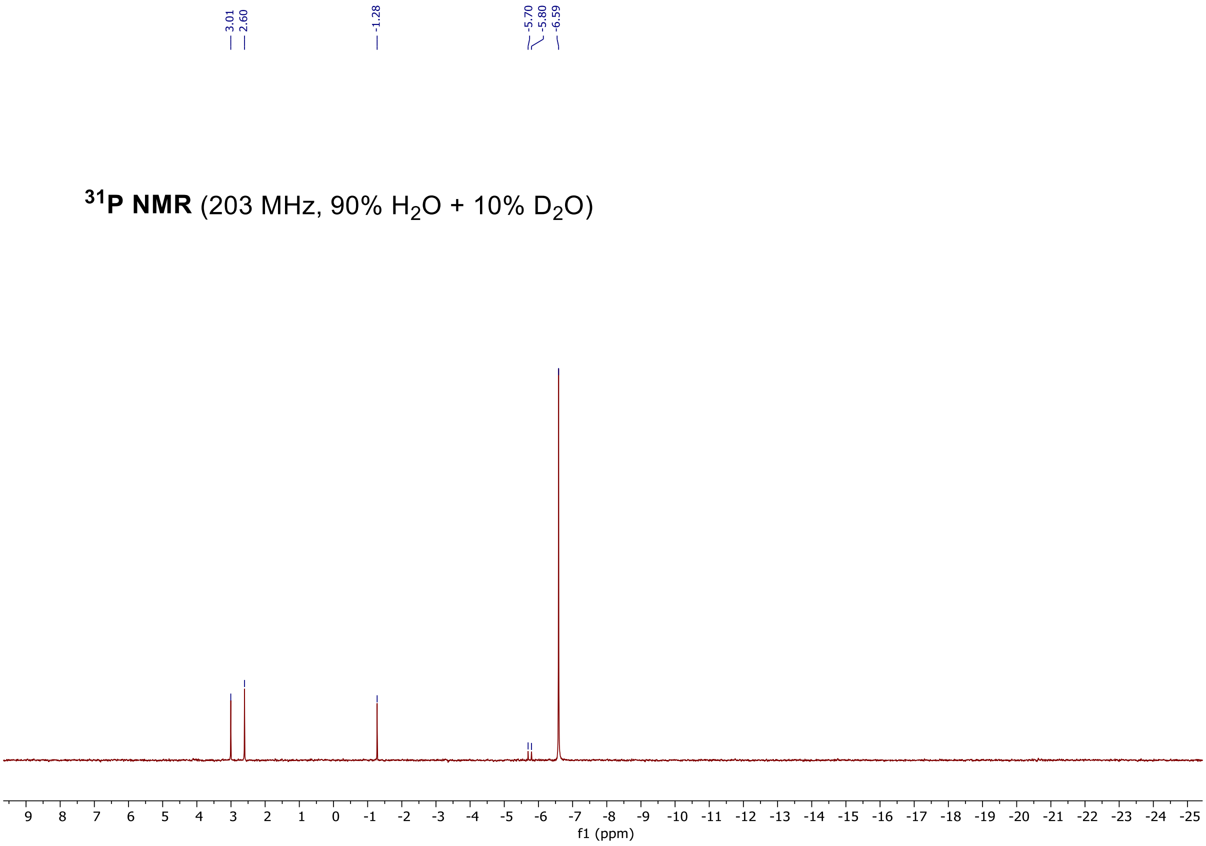

n(OTf$^-$)	m (sample)	Yield of F$^-$ %	Yield of PO_3F^{2-} %
0.058 mmol	36.6 mg	7%	91%

^{19}F NMR (471 MHz, 90% H_2O + 10% D_2O)

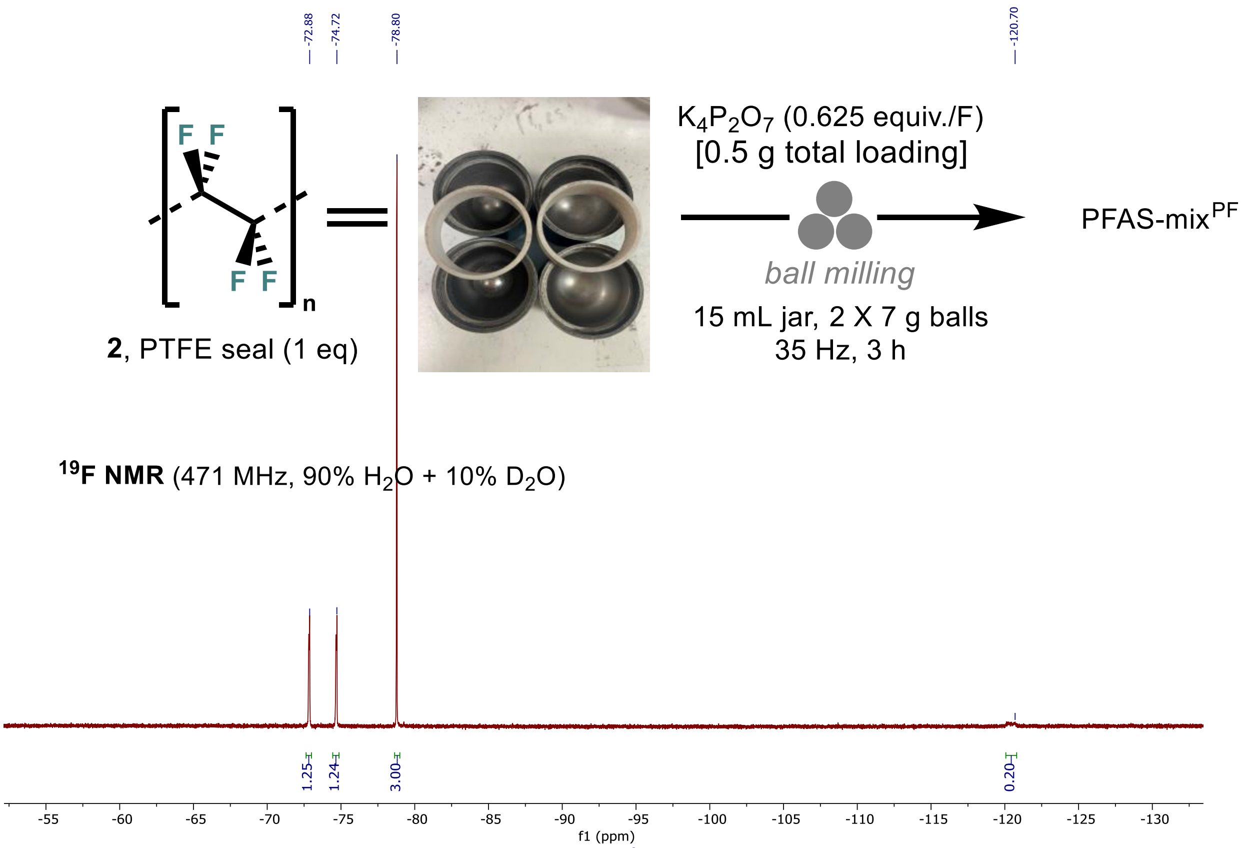

^{31}P NMR (203 MHz, 90% H_2O + 10% D_2O)

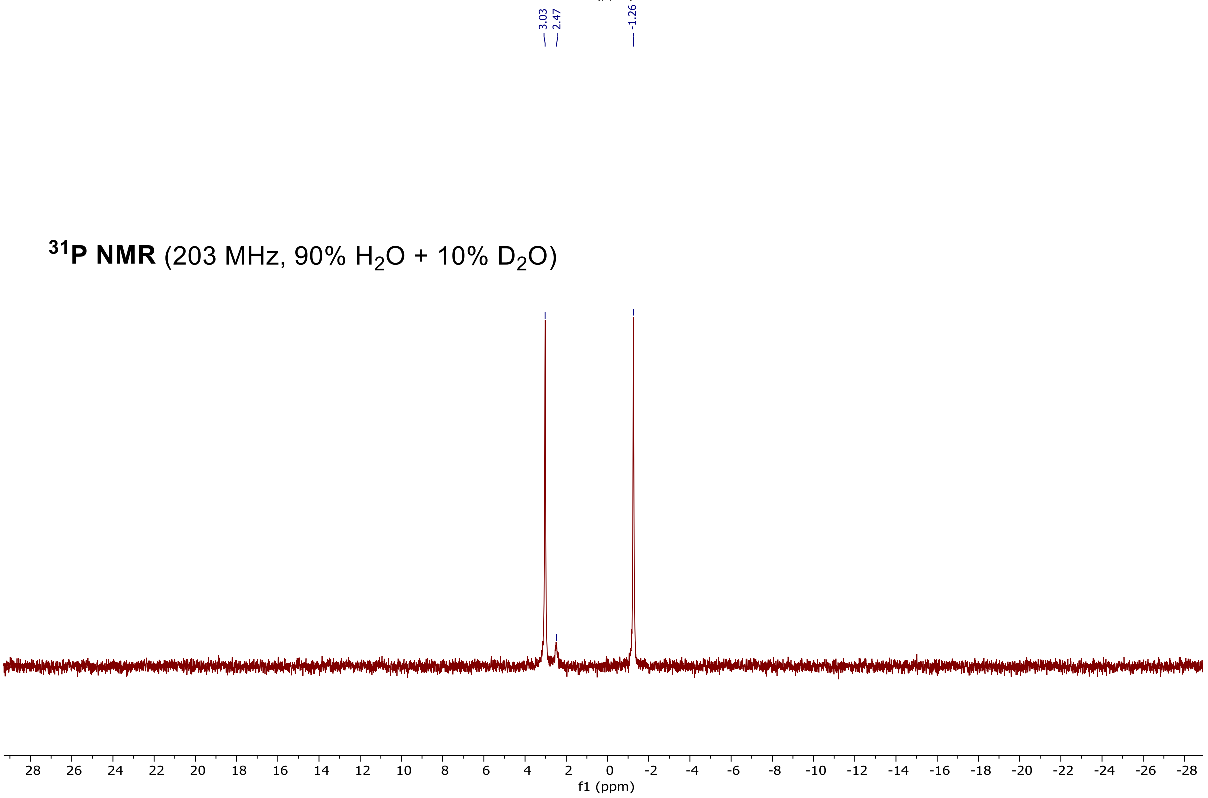

n(OTf$^-$)	m (sample)	Yield of F$^-$ %	Yield of PO_3F^{2-} %
0.058 mmol	38.0 mg	84%	16%

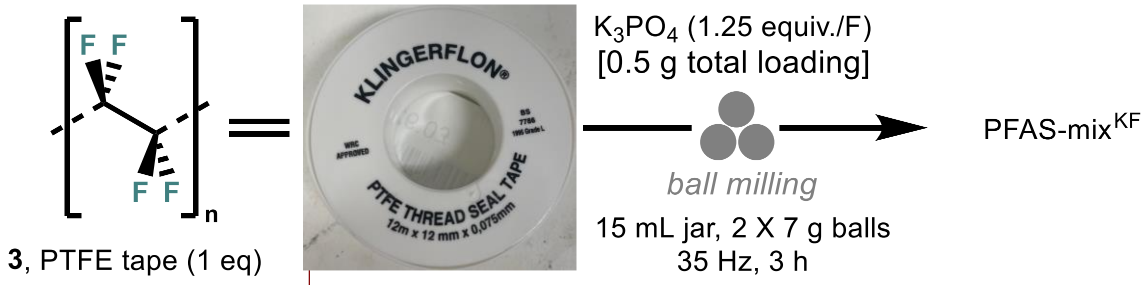

^{19}F NMR (471 MHz, 90% H_2O + 10% D_2O)

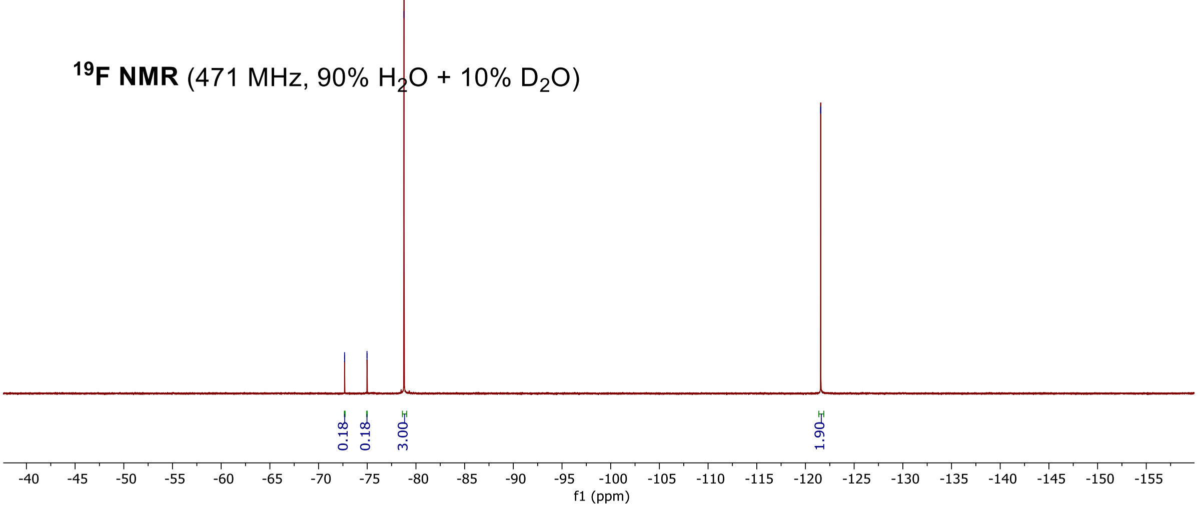

^{31}P NMR (203 MHz, 90% H_2O + 10% D_2O)

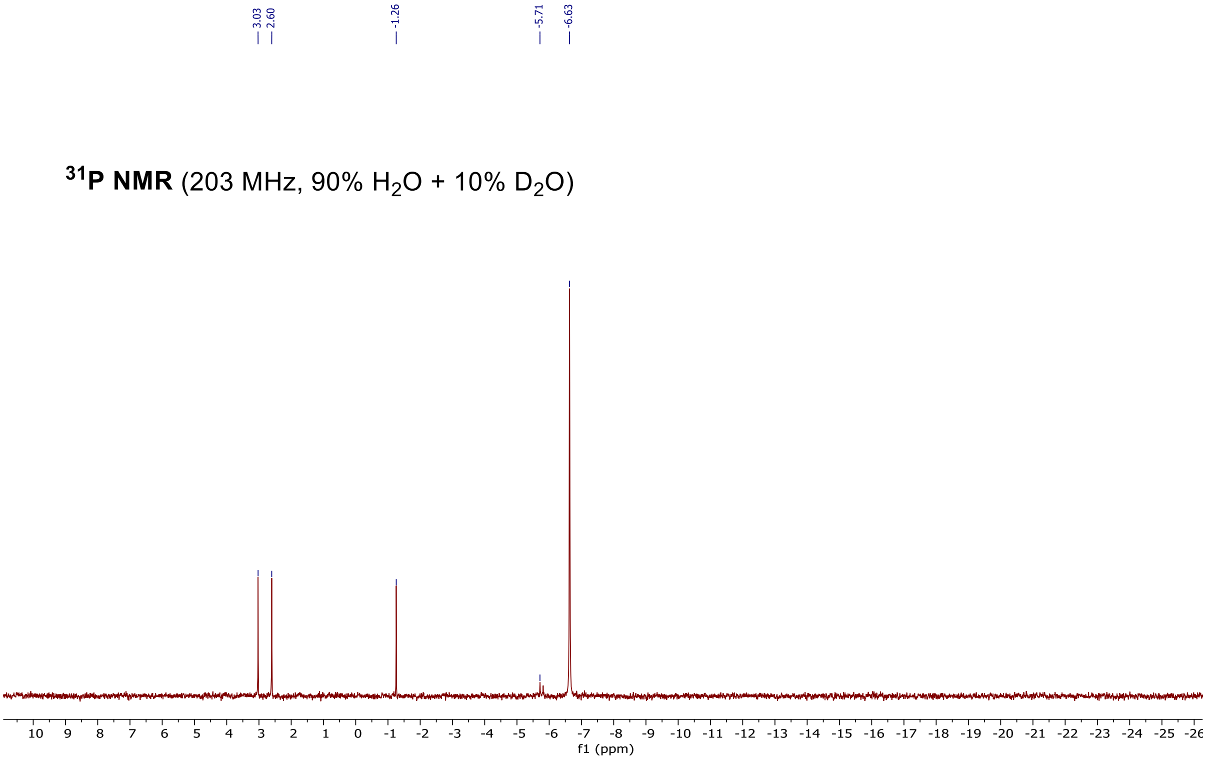

n(OTf⁻)	m (sample)	Yield of F⁻ %	Yield of PO_3F^{2-} %
0.058 mmol	30.9 mg	0%	quant.

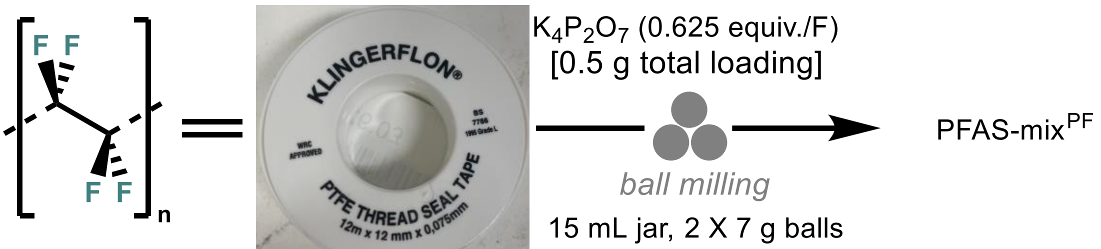

3, PTFE tape (1 eq)

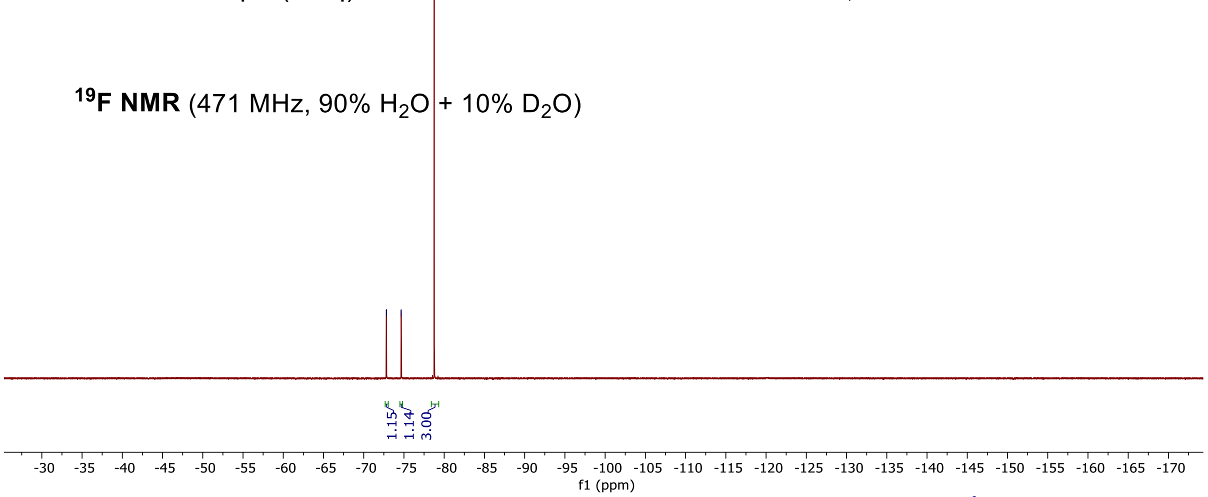

^{19}F NMR (471 MHz, 90% H_2O + 10% D_2O)

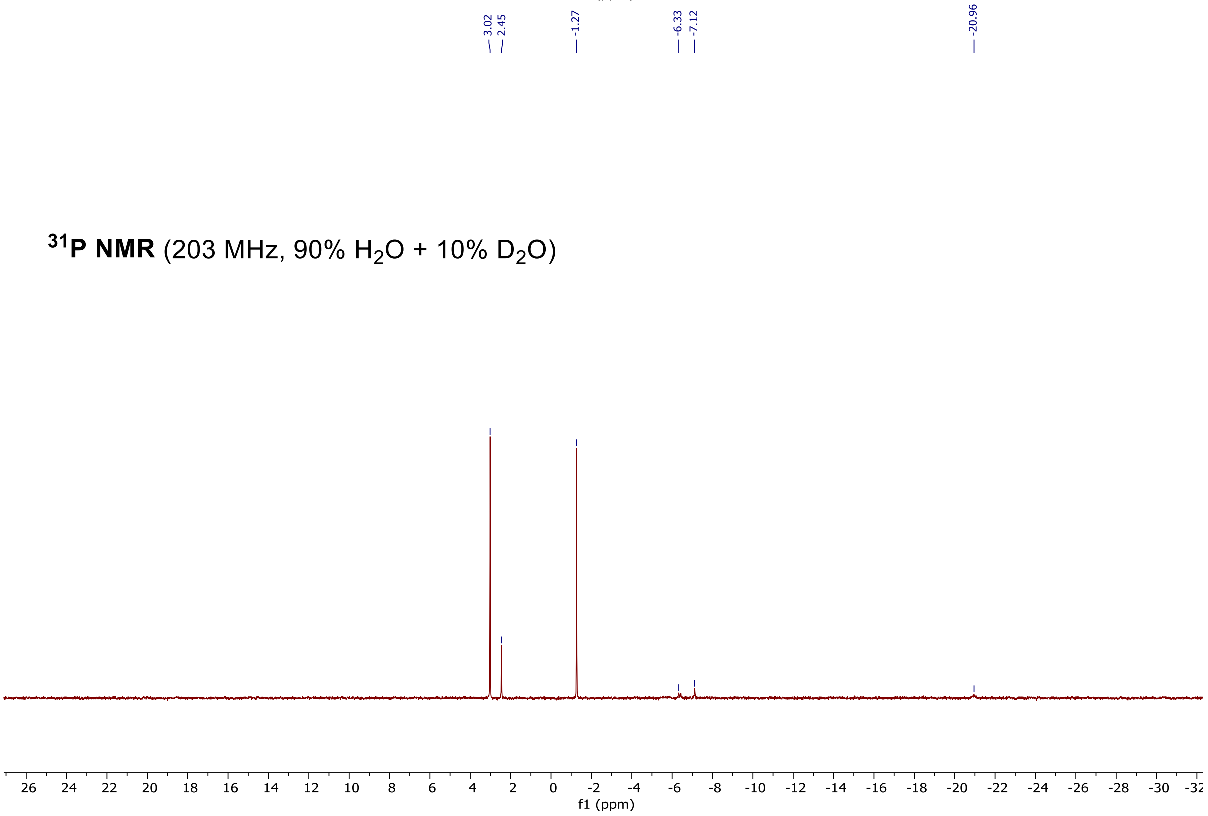

^{31}P NMR (203 MHz, 90% H_2O + 10% D_2O)

n(OTf^-)	m (sample)	Yield of F^- %	Yield of PO_3F^{2-} %
0.058 mmol	47.6 mg	91%	7%

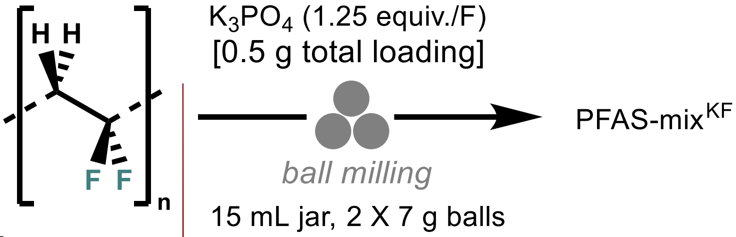

4, PVDF (1 eq)

^{19}F NMR (471 MHz, 90% H_2O + 10% D_2O)

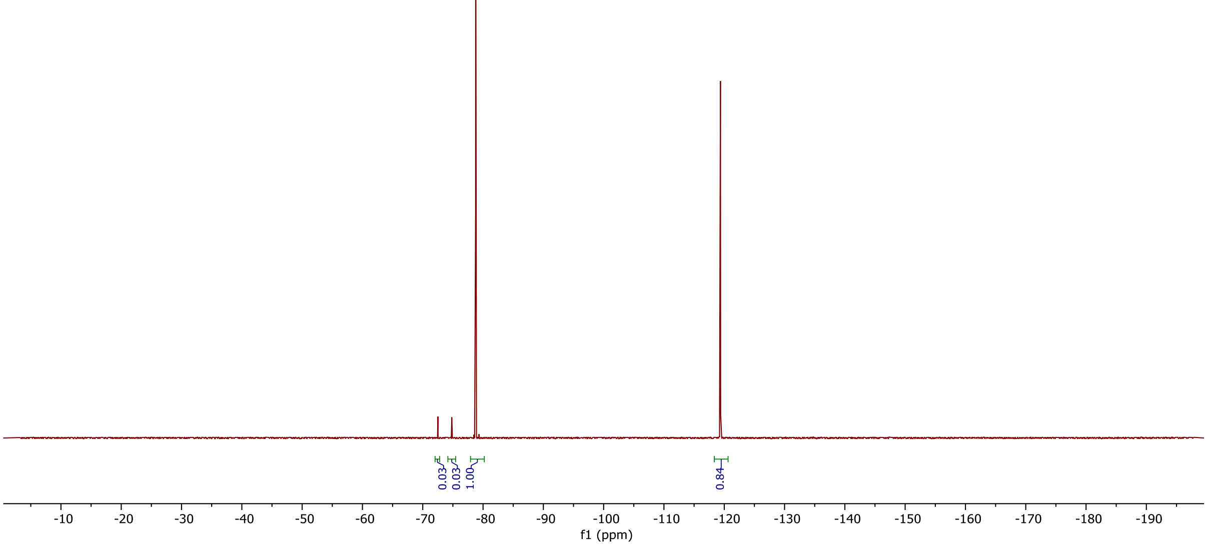

^{31}P NMR (203 MHz, 90% H_2O + 10% D_2O)

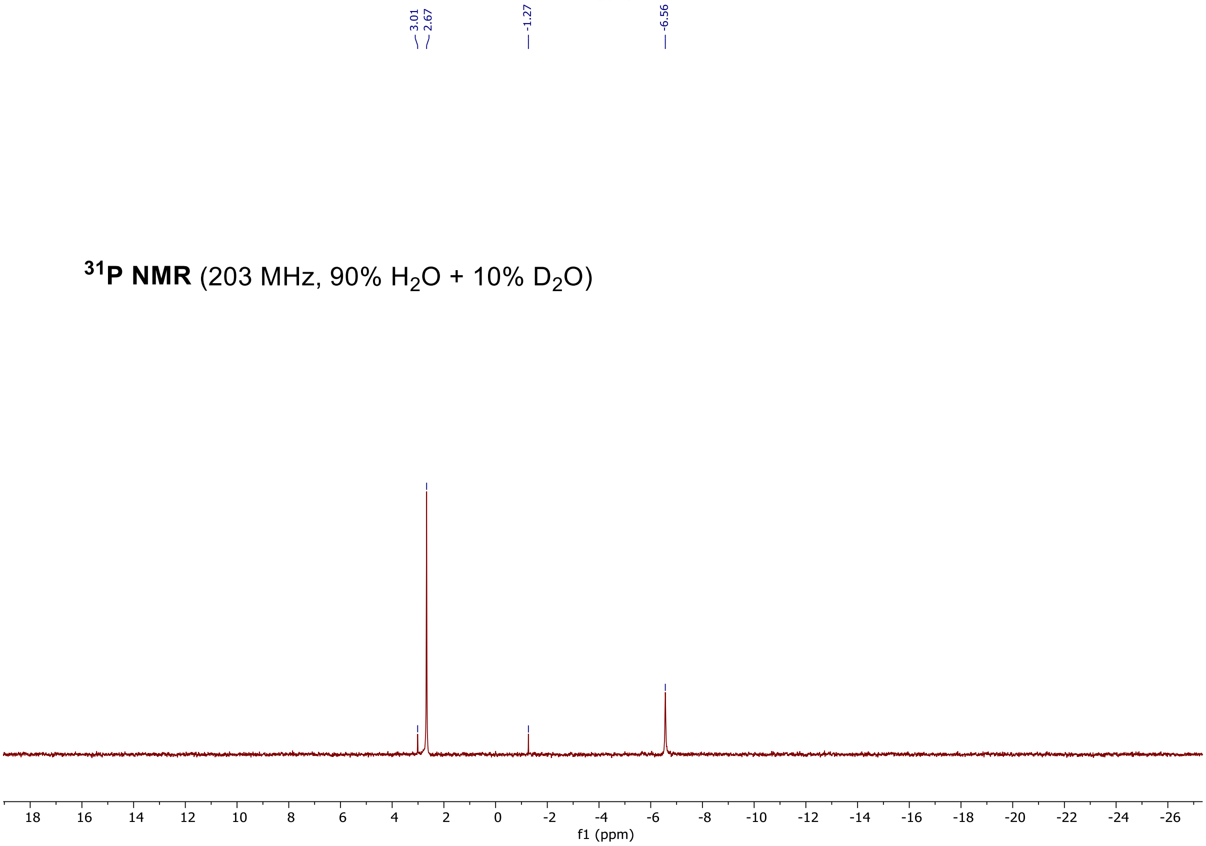

n(OTf⁻)	m (sample)	Yield of F⁻ %	Yield of PO_3F^{2-} %
0.063 mmol	51.5 mg	16%	79%

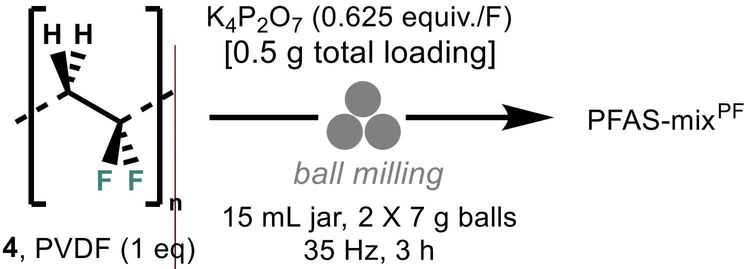

4, PVDF (1 eq)

^{19}F NMR (471 MHz, 90% H_2O + 10% D_2O)

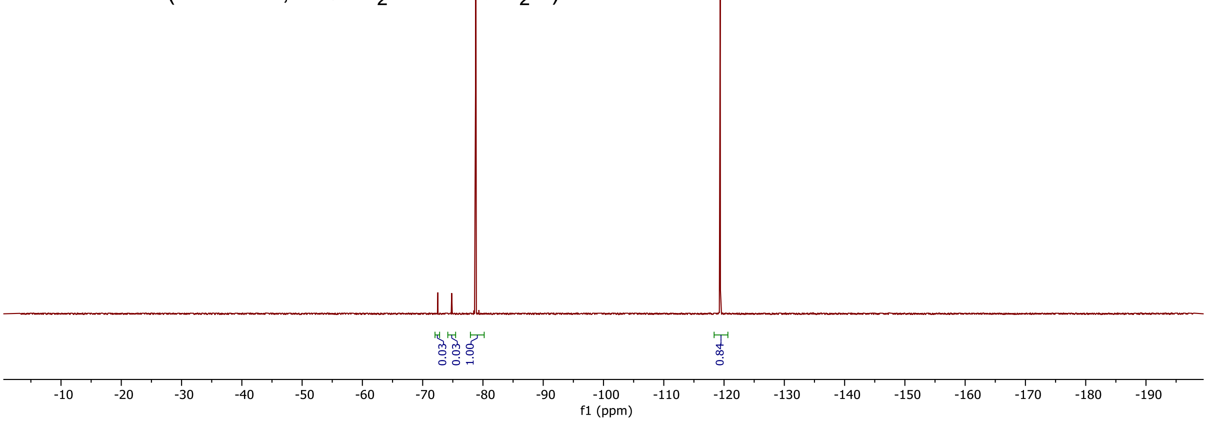

^{31}P NMR (203 MHz, 90% H_2O + 10% D_2O)

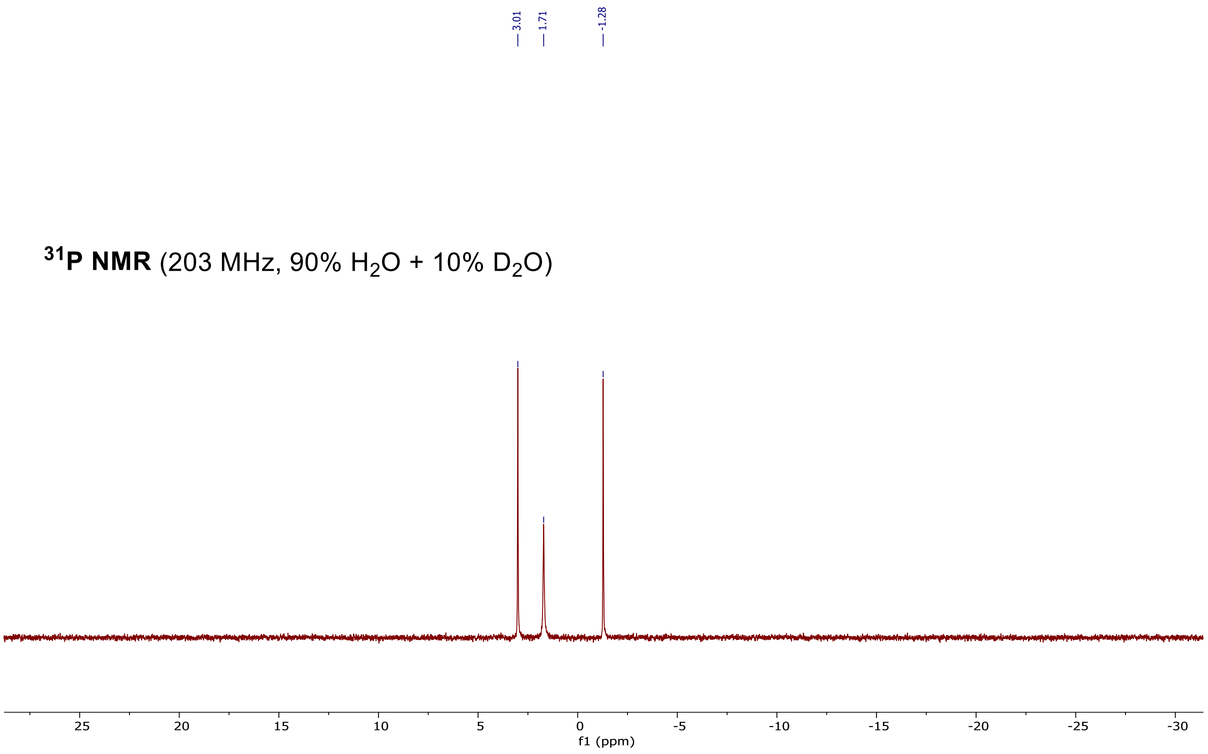

n(OTf$^-$)	m (sample)	Yield of F$^-$ %	Yield of PO_3F^{2-} %
0.058 mmol	70.6 mg	64%	34%

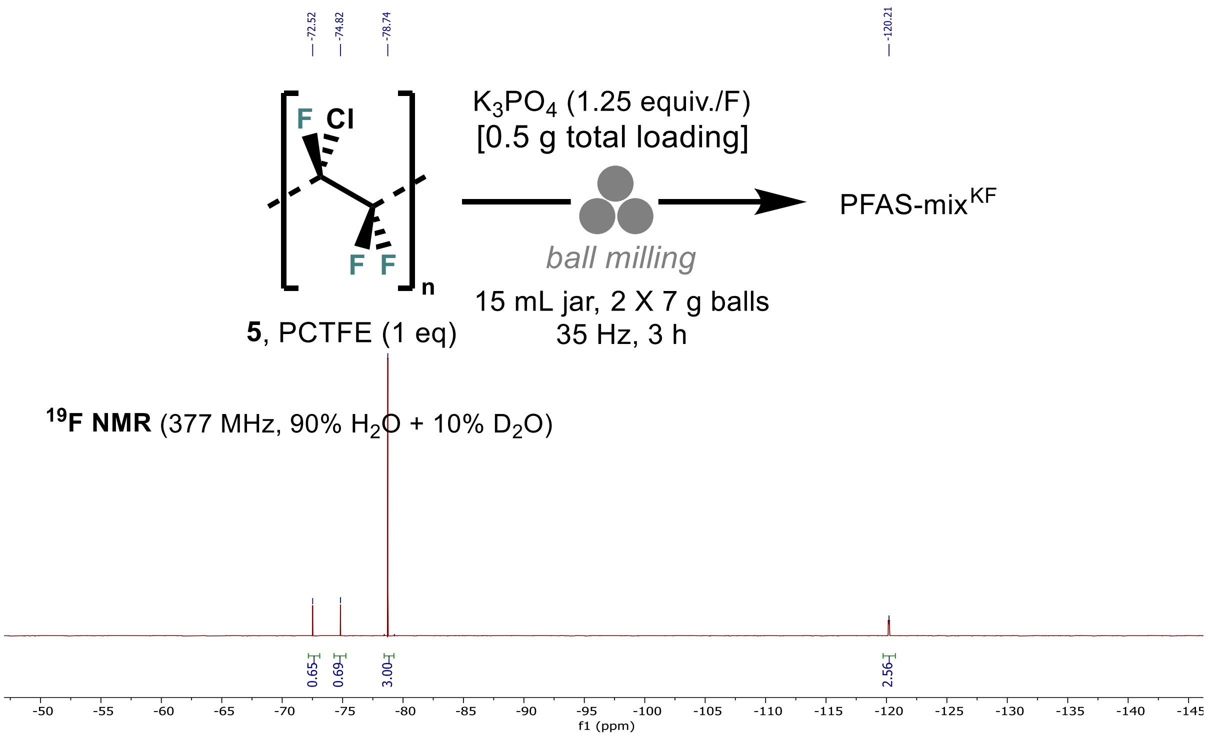

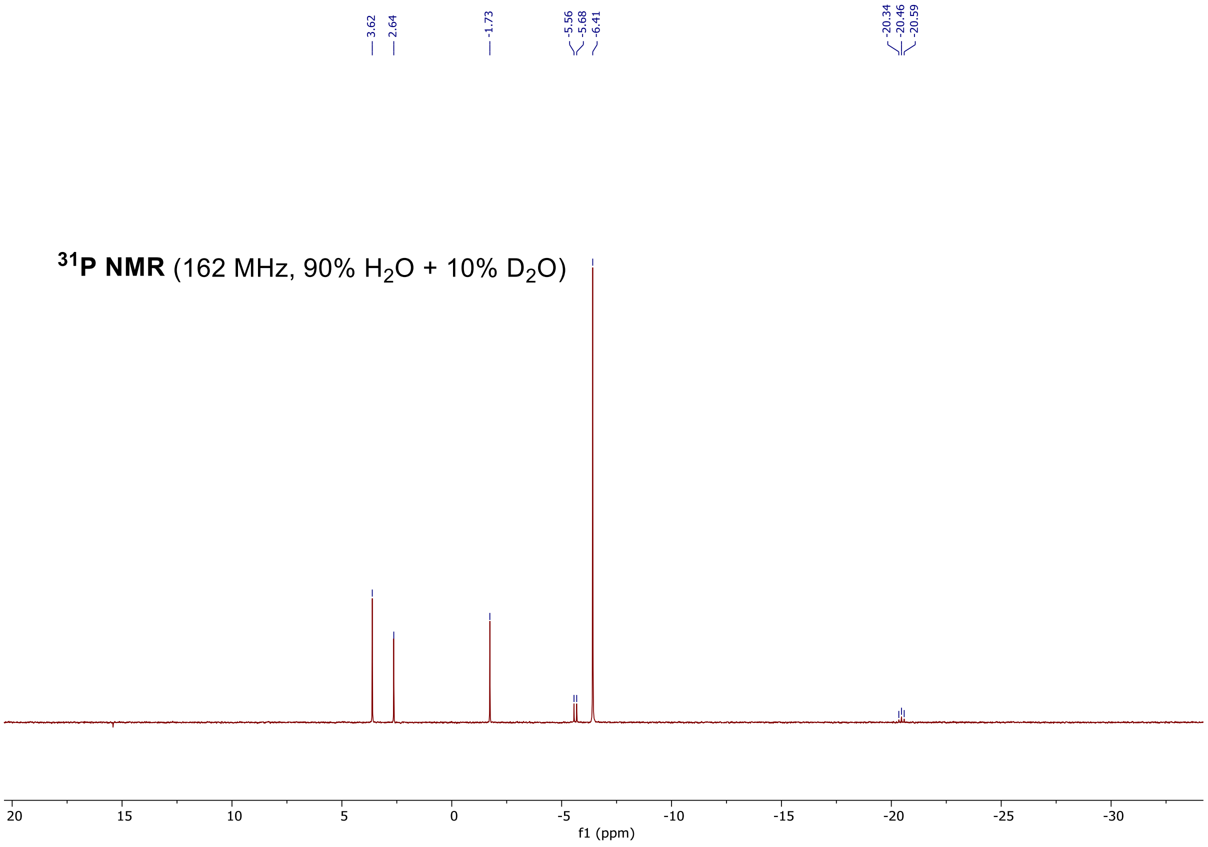

n(OTf⁻)	m (sample)	Yield of F⁻ %	Yield of PO_3F^{2-} %
0.058 mmol	38.2 mg	0%	95%

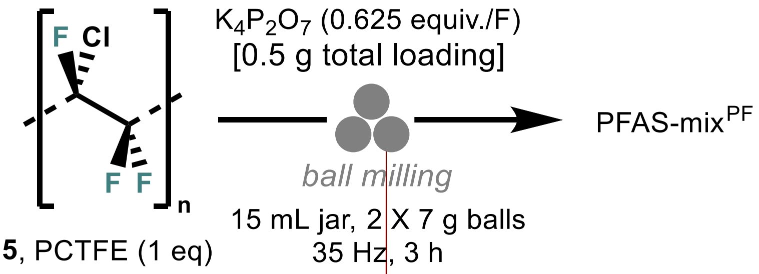

^{19}F NMR (471 MHz, 90% H_2O + 10% D_2O)

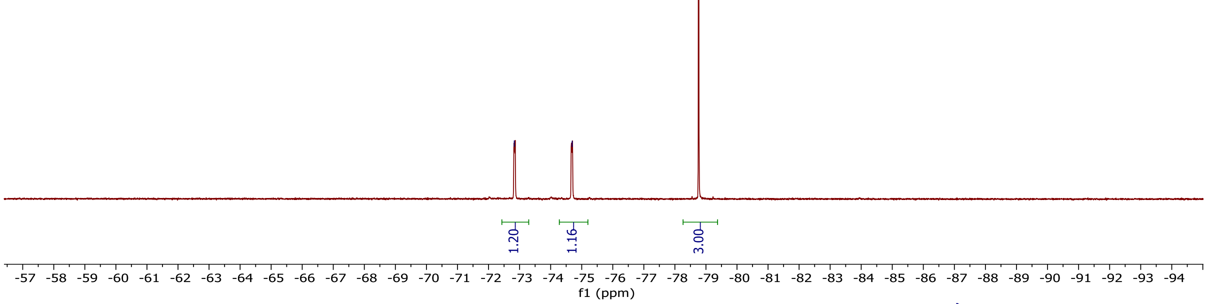

^{31}P NMR (203 MHz, 90% H_2O + 10% D_2O)

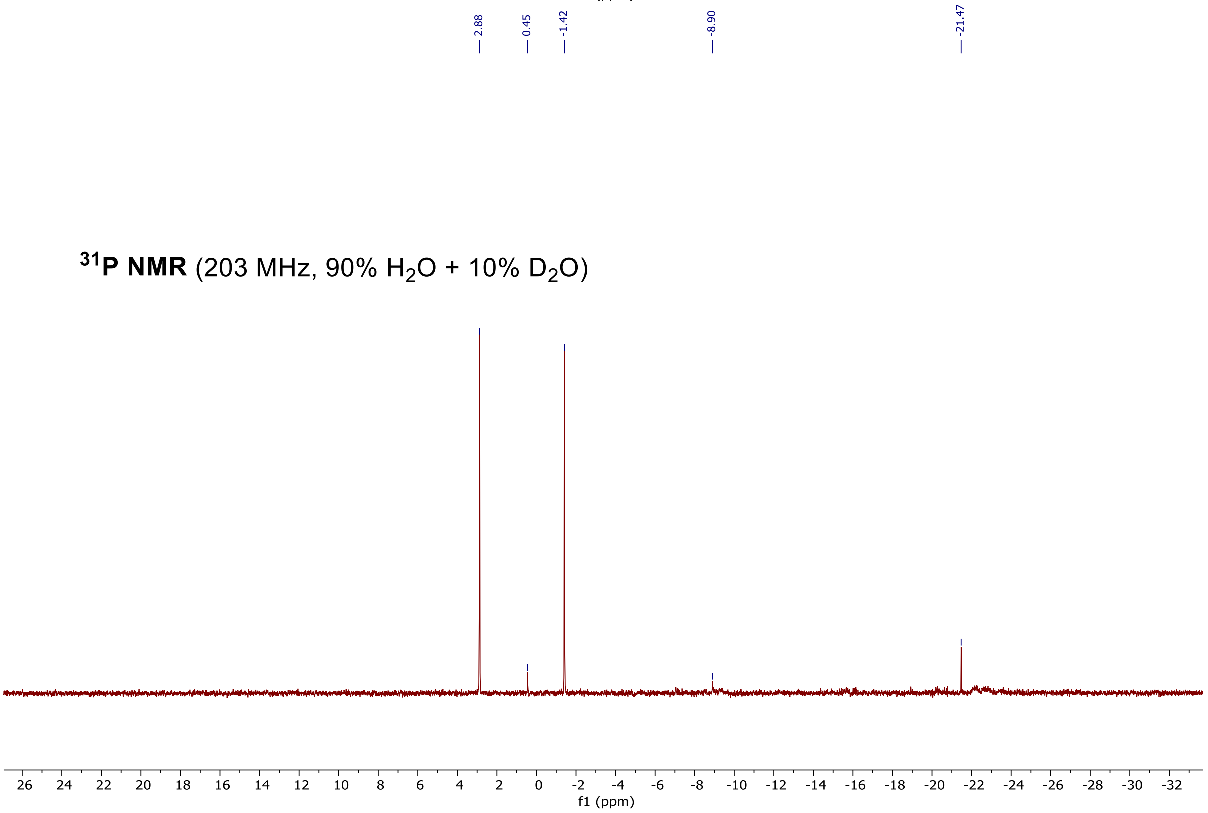

n(OTf⁻)	m (sample)	Yield of F^- %	Yield of PO_3F^{2-} %
0.058 mmol	45.0 mg	91%	13%

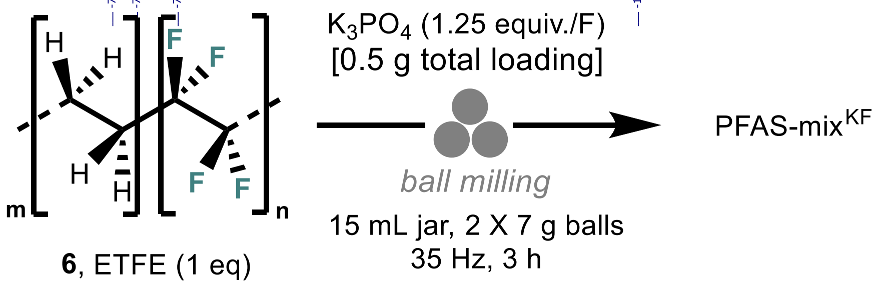

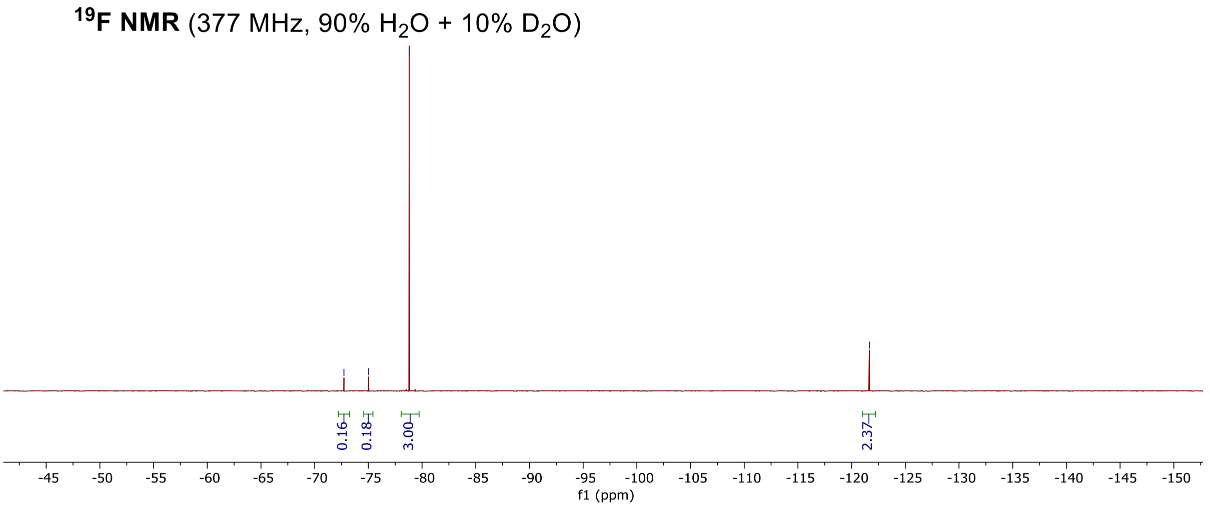

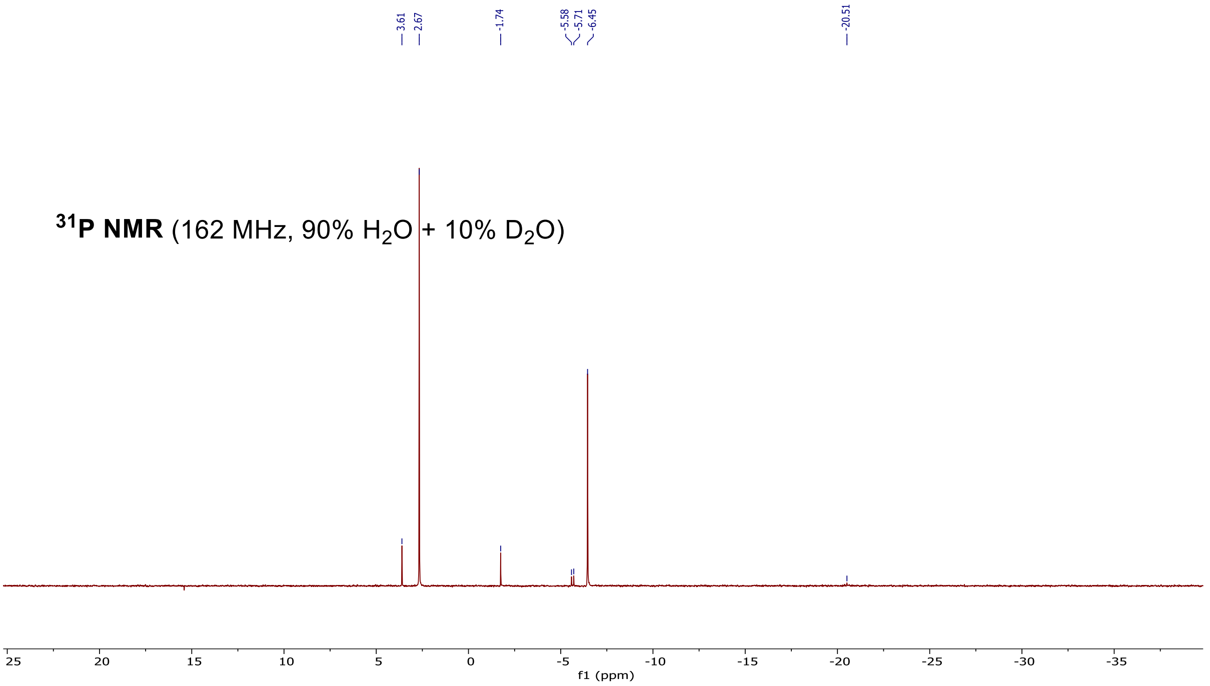

n(OTf⁻)	m (sample)	Yield of F⁻ %	Yield of PO_3F^{2-} %
0.058 mmol	47.6 mg	12%	77%

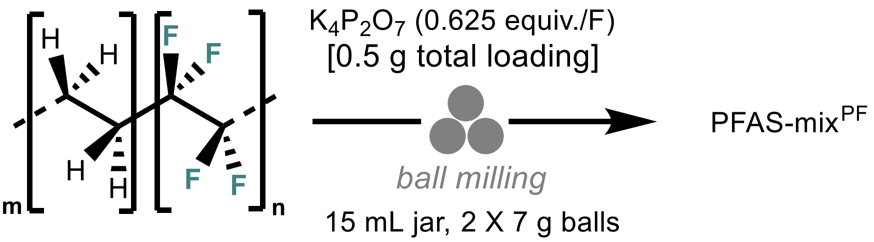

6, ETFE (1 eq)

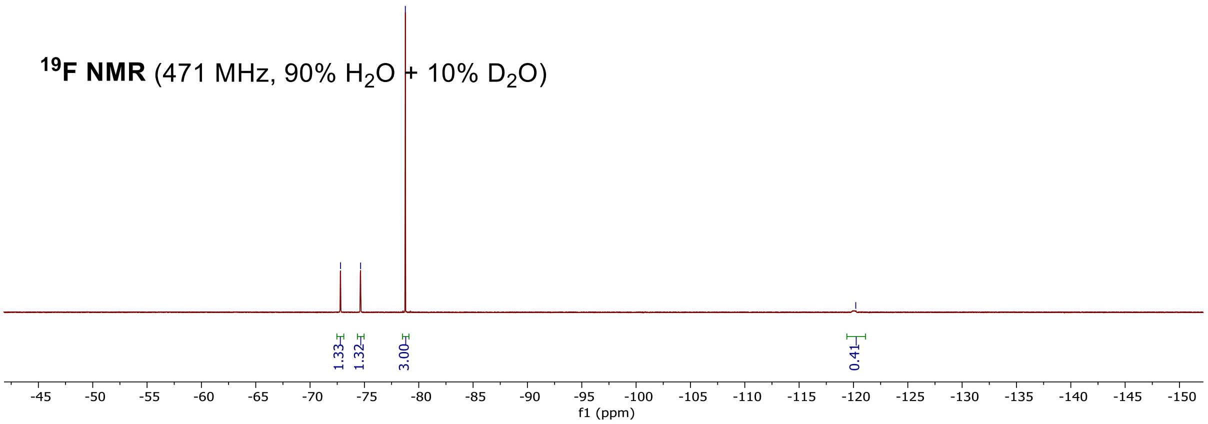

^{19}F NMR (471 MHz, 90% H_2O + 10% D_2O)

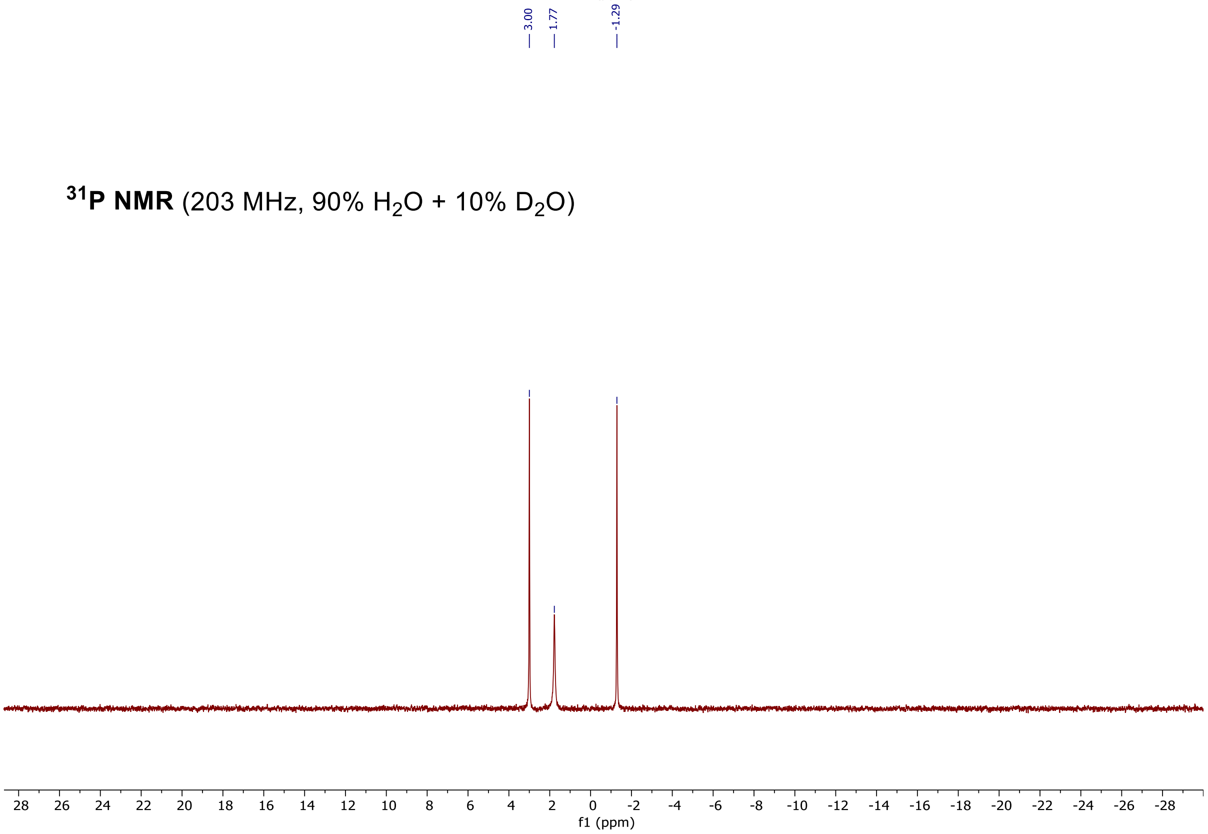

^{31}P NMR (203 MHz, 90% H_2O + 10% D_2O)

n(OTf⁻)	m (sample)	Yield of F⁻ %	Yield of PO_3F^{2-} %
0.058 mmol	42 mg	88%	8%

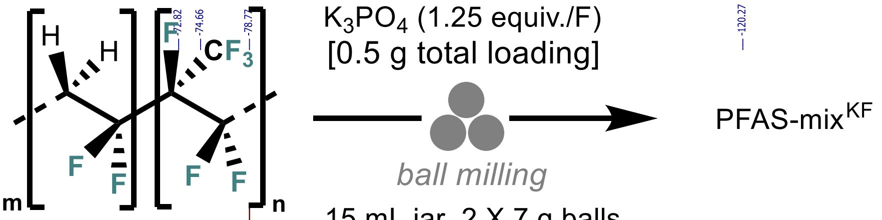

7, PVDF-HFP (1 eq)

^{19}F NMR (471 MHz, 90% H_2O + 10% D_2O)

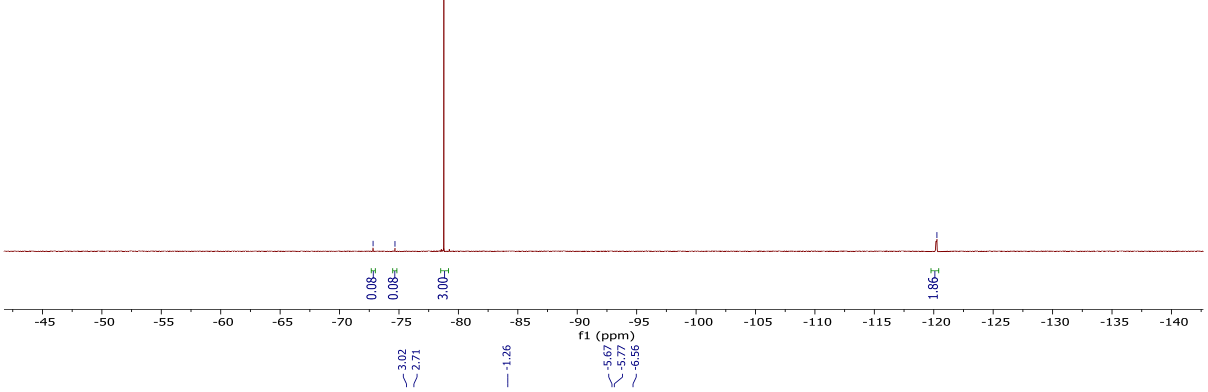

^{31}P NMR (203 MHz, 90% H_2O + 10% D_2O)

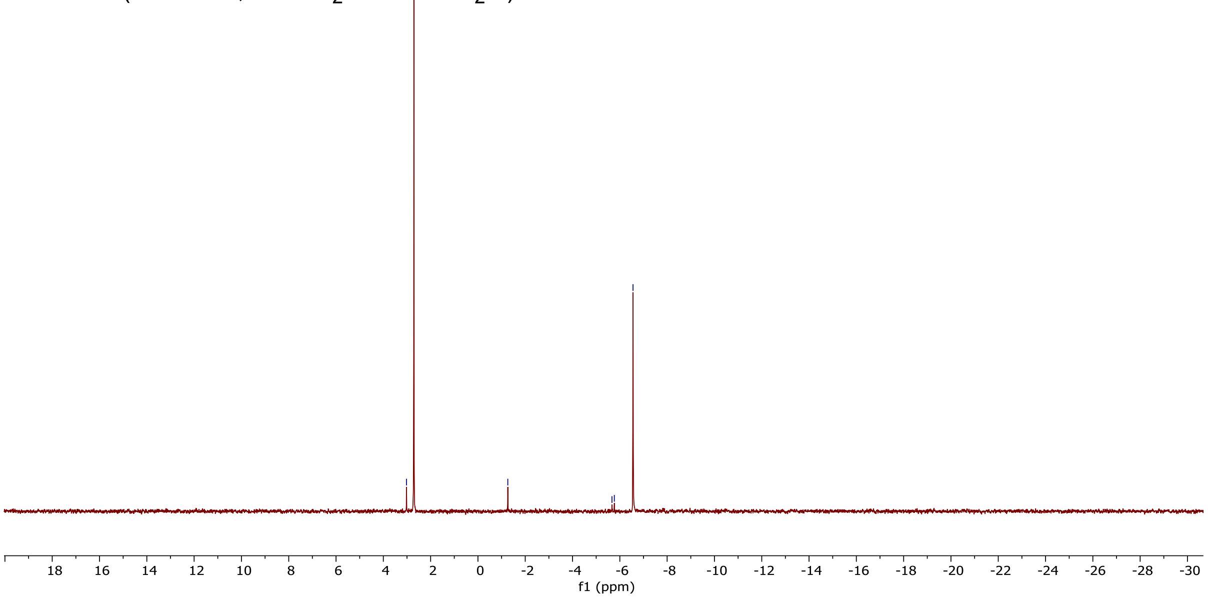

$n(OTf^-)$	m (sample)	Yield of F^- %	Yield of PO_3F^{2-} %
0.058 mmol	35.5 mg	2%	94%

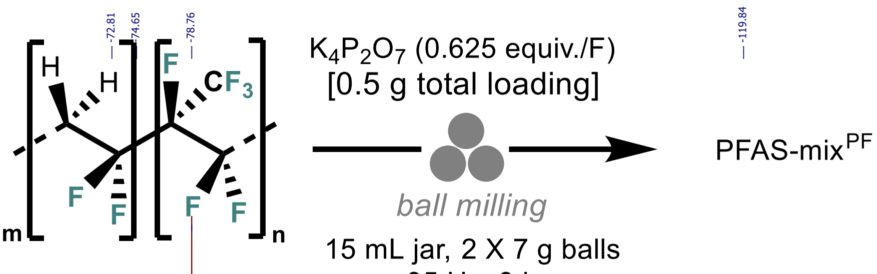

7, PVDF-HFP (1 eq)

^{19}F NMR (471 MHz, 90% H_2O + 10% D_2O)

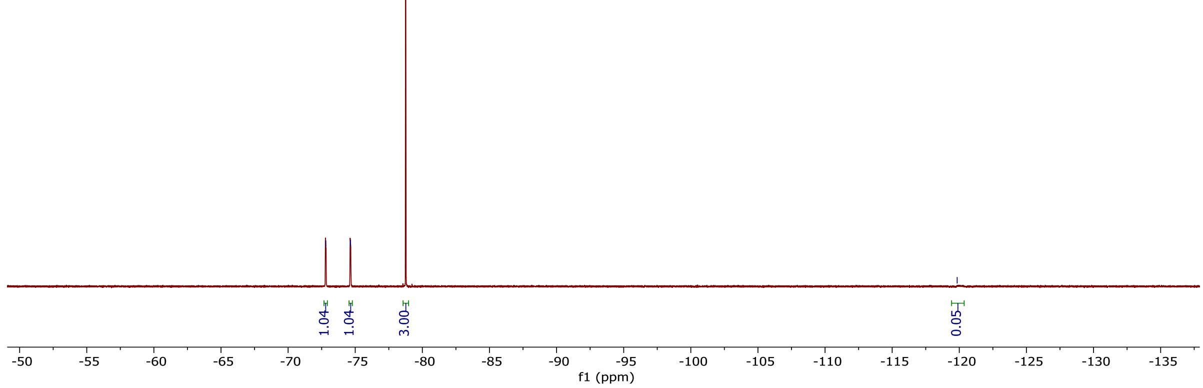

^{31}P NMR (203 MHz, 90% H_2O + 10% D_2O)

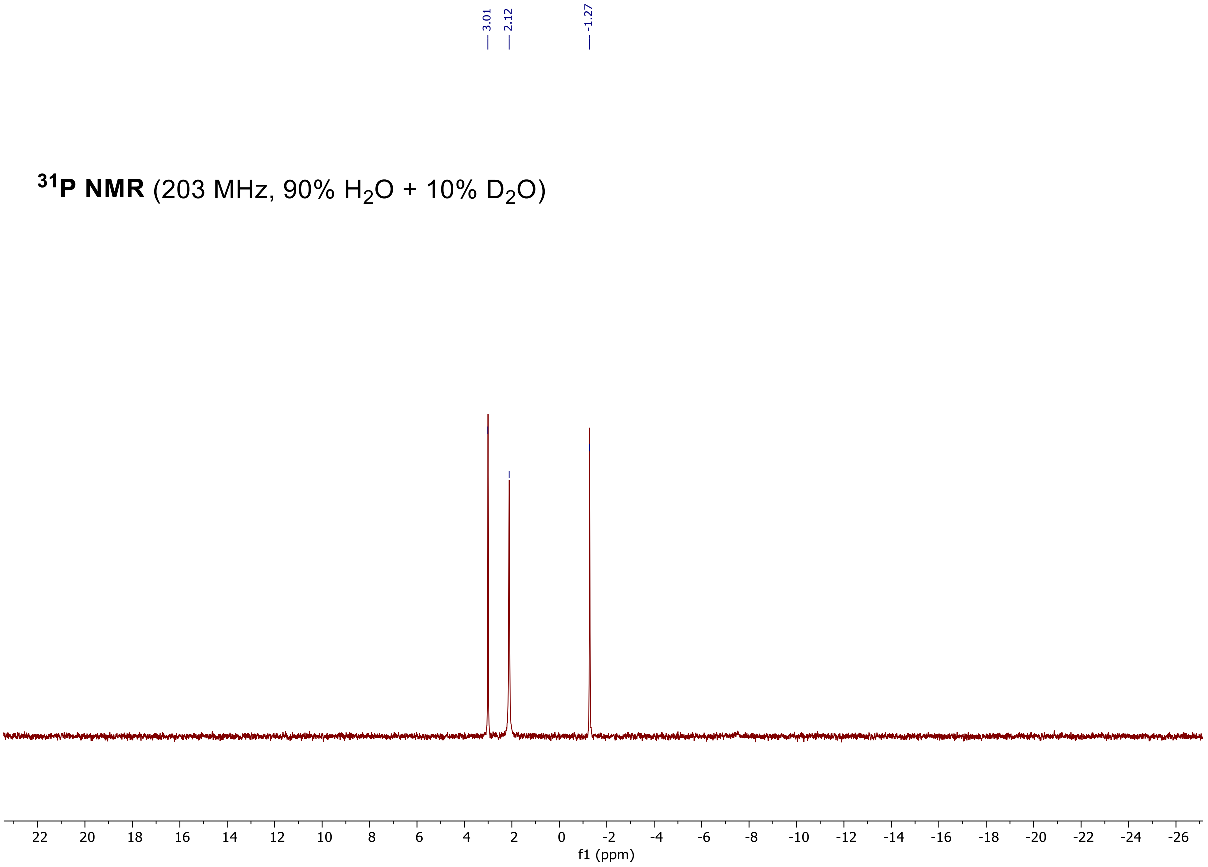

n(OTf⁻)	m (sample)	Yield of F⁻ %	Yield of PO_3F^{2-} %
0.058 mmol	51.7 mg	99%	2%

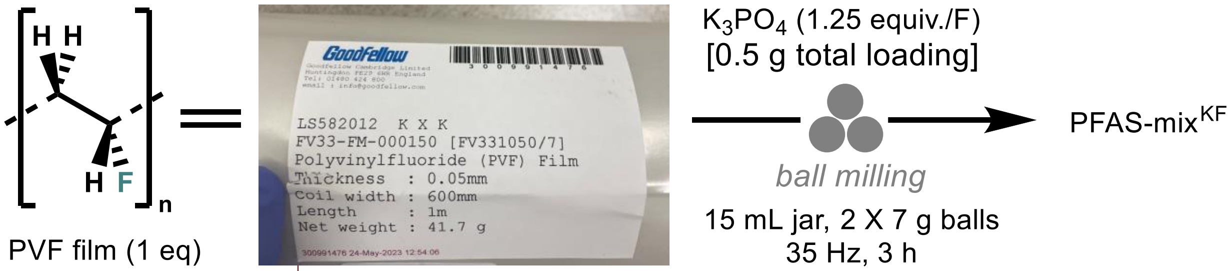

8, PVF film (1 eq)

^{19}F NMR (471 MHz, 90% H_2O + 10% D_2O)

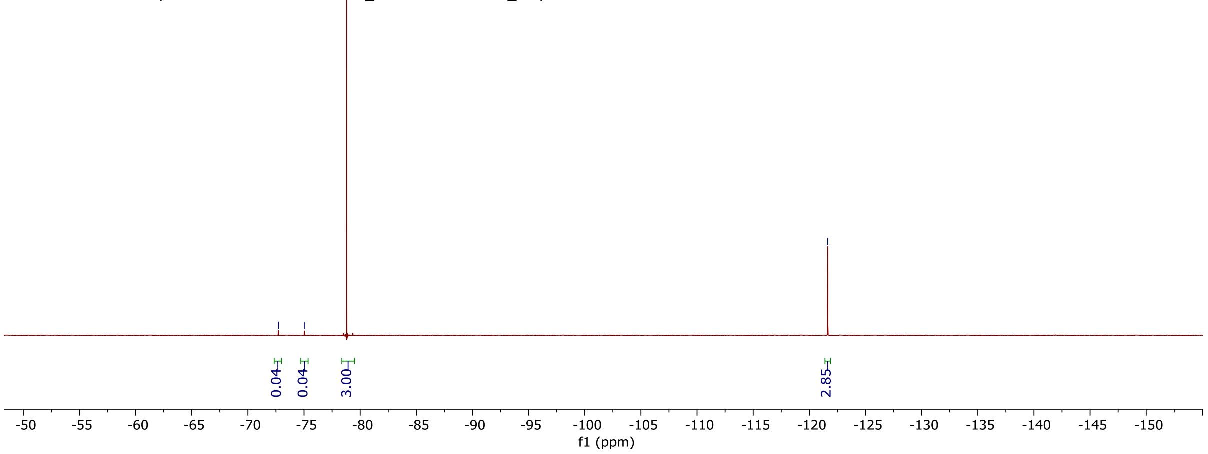

^{31}P NMR (203 MHz, 90% H_2O + 10% D_2O)

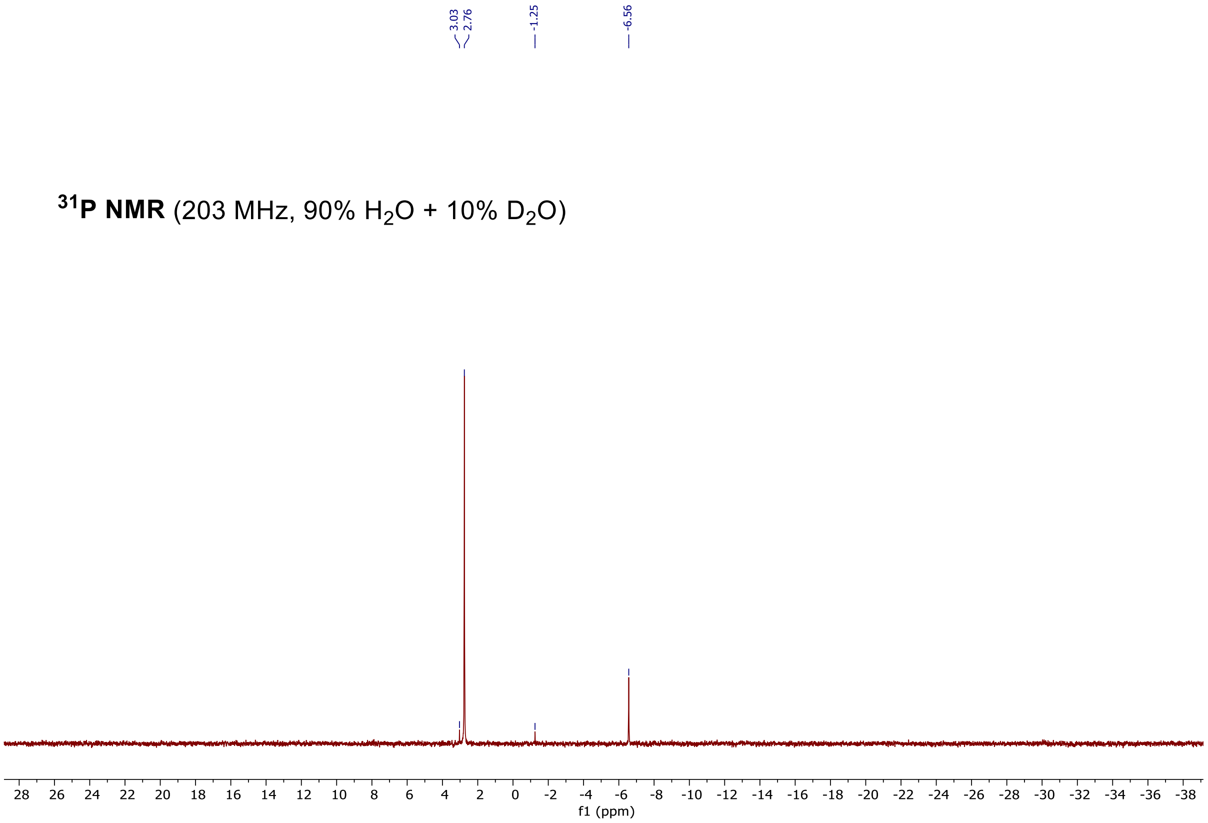

n(OTf⁻)	m (sample)	Yield of F⁻ %	Yield of PO_3F^{2-} %
0.058 mmol	36.6 mg	12%	78%

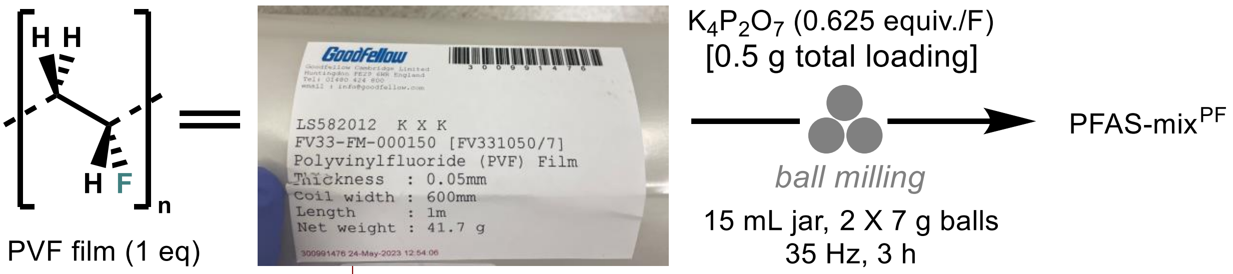

8, PVF film (1 eq)

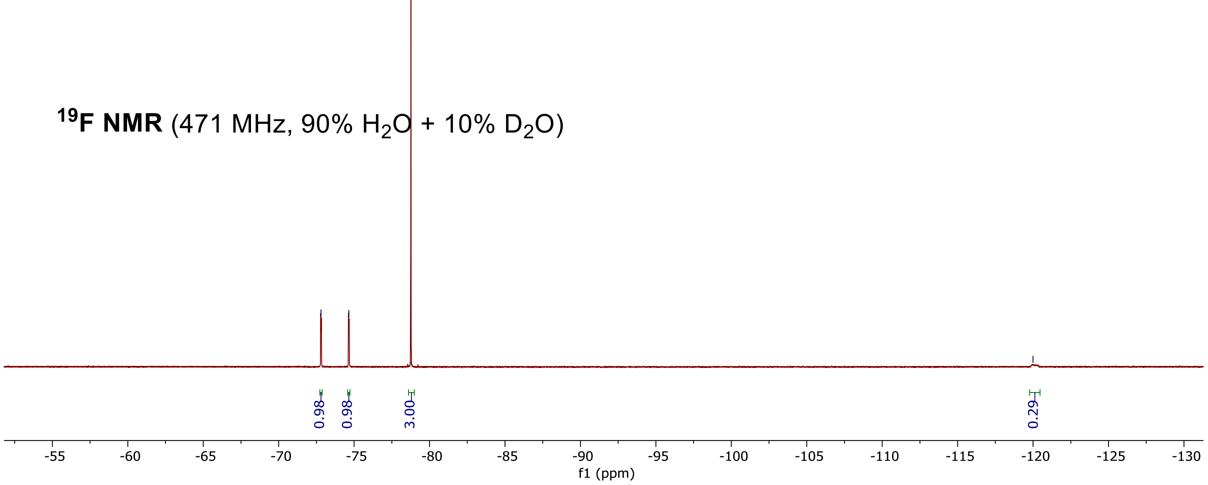

^{19}F NMR (471 MHz, 90% H_2O + 10% D_2O)

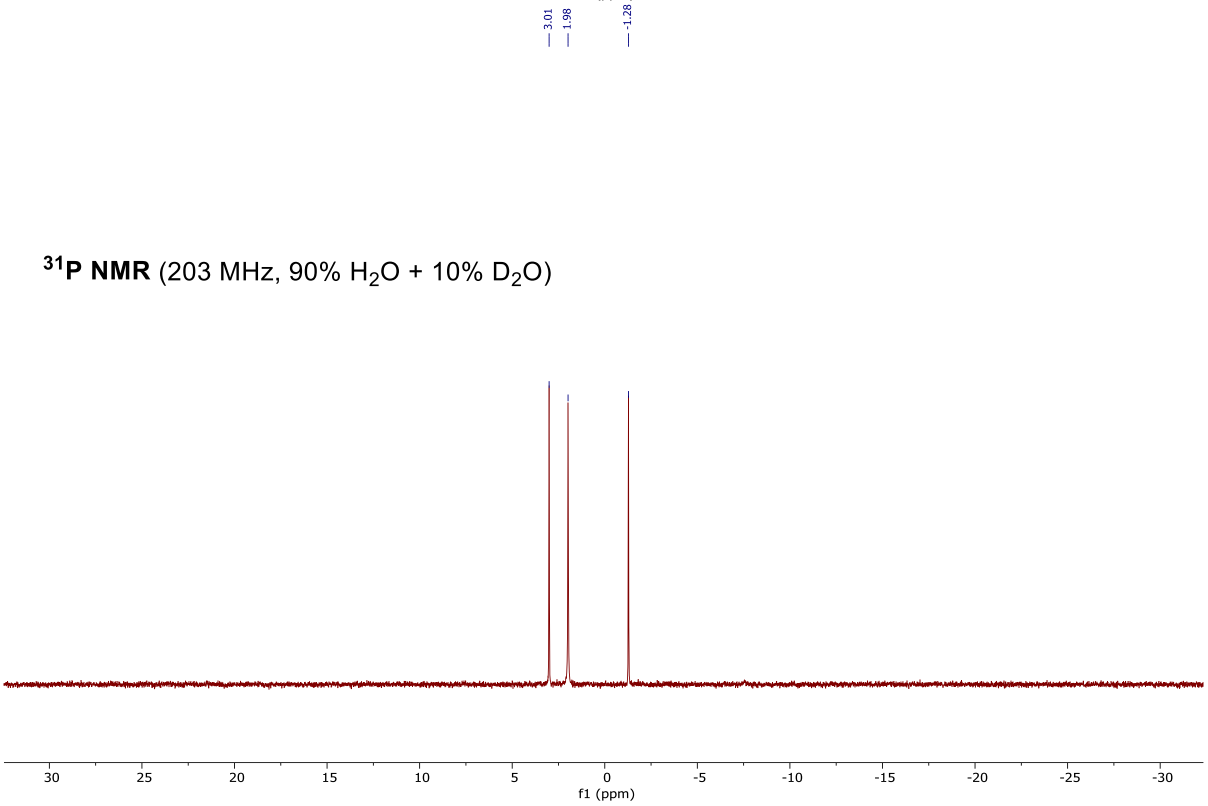

^{31}P NMR (203 MHz, 90% H_2O + 10% D_2O)

n(OTf⁻)	m (sample)	Yield of F⁻ %	Yield of PO_3F^{2-} %
0.058 mmol	38.2 mg	82%	18%

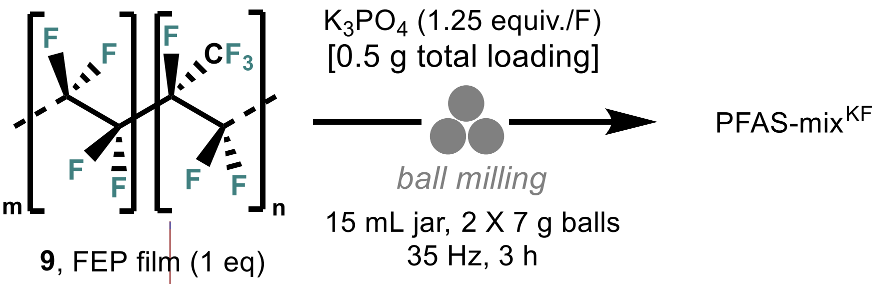

^{19}F NMR (377 MHz, 90% H_2O + 10% D_2O)

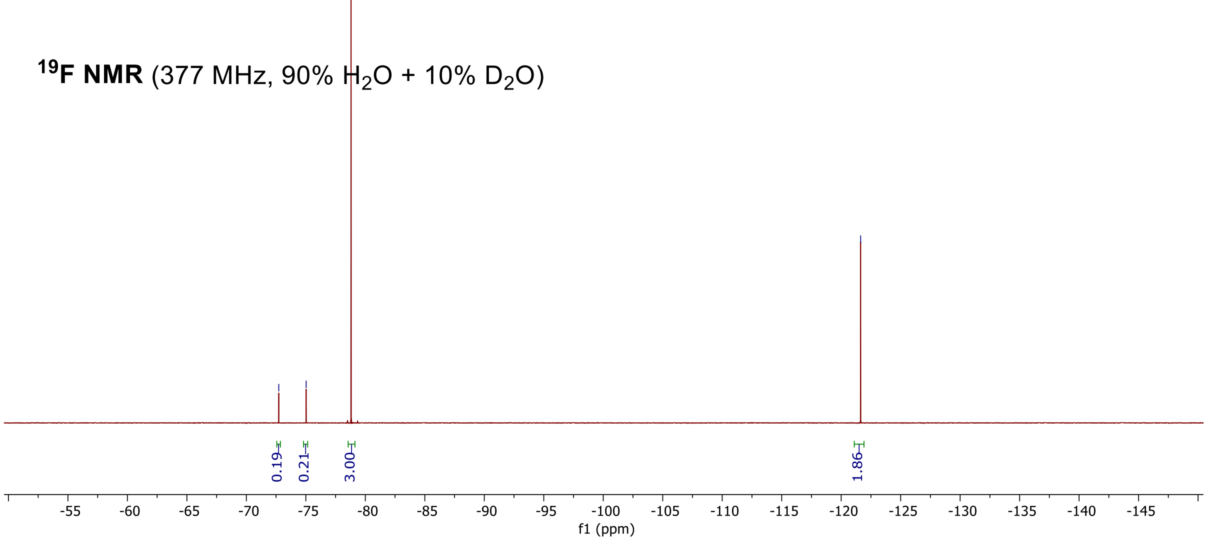

^{31}P NMR (162 MHz, 90% H_2O + 10% D_2O)

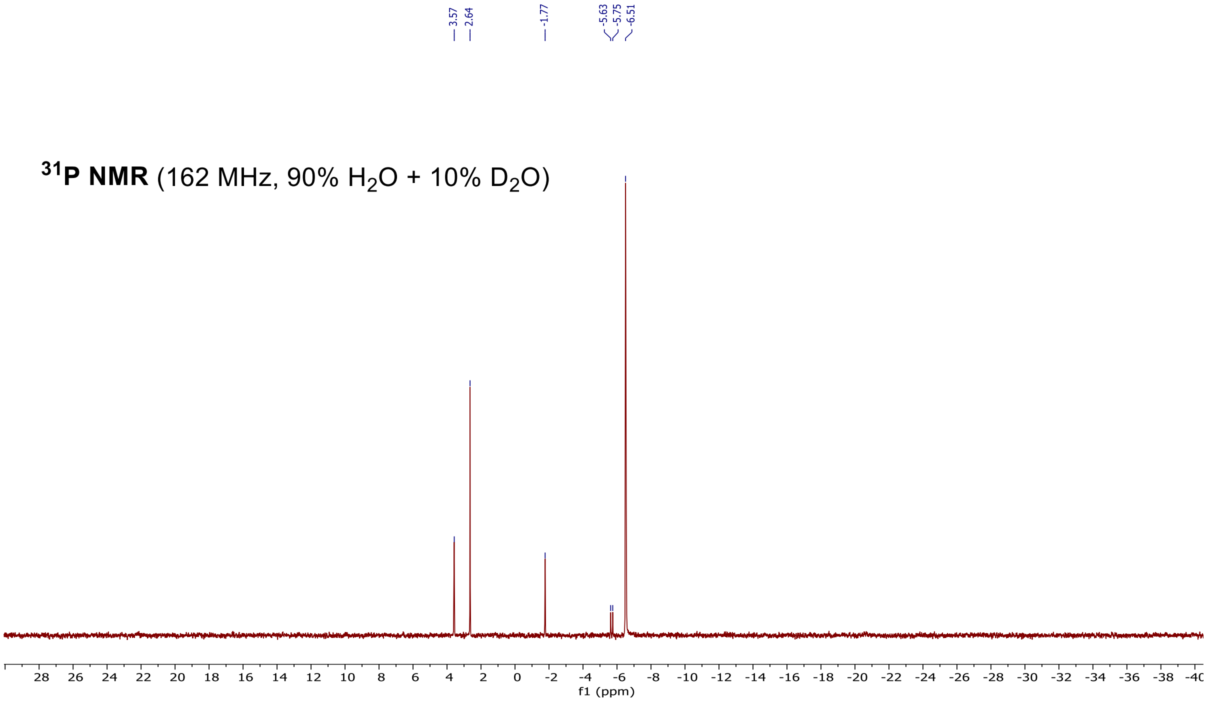

n(OTf⁻)	m (sample)	Yield of F⁻ %	Yield of PO_3F^{2-} %
0.058 mmol	54.7 mg	0%	97%

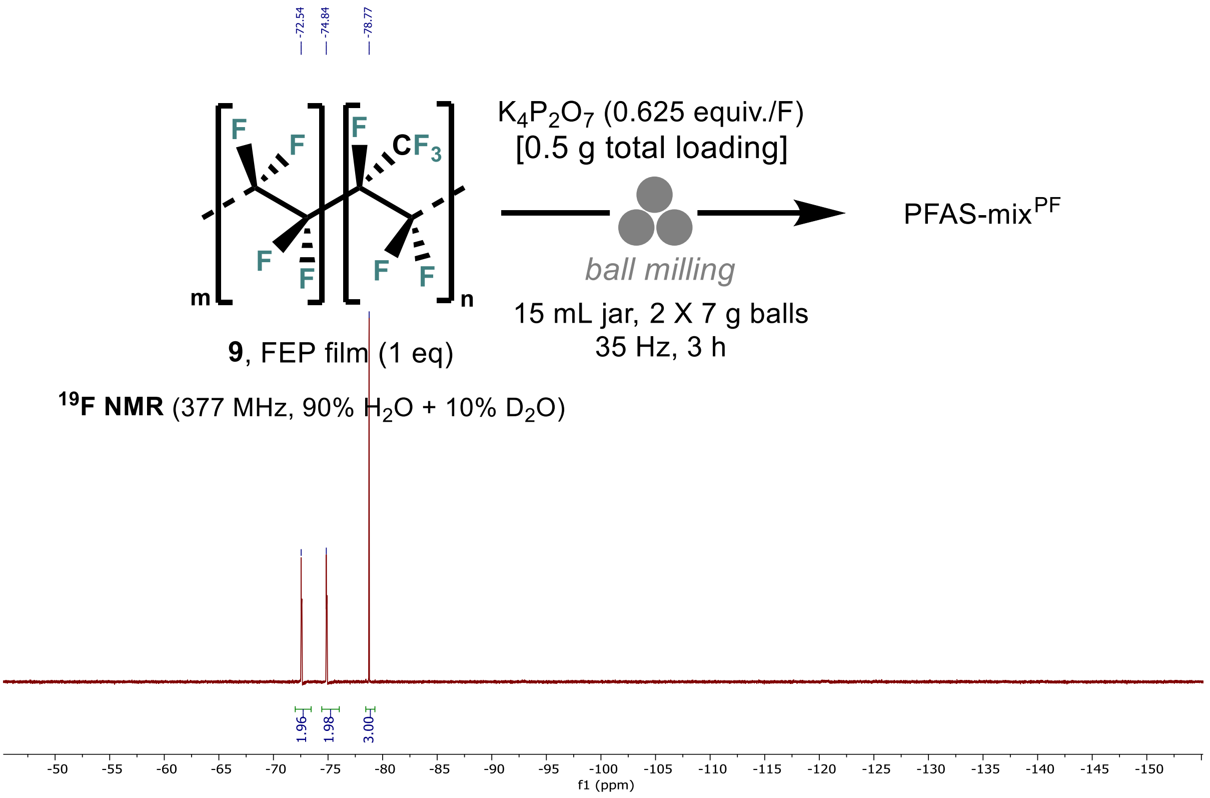

^{19}**F NMR** (377 MHz, 90% H_2O + 10% D_2O)

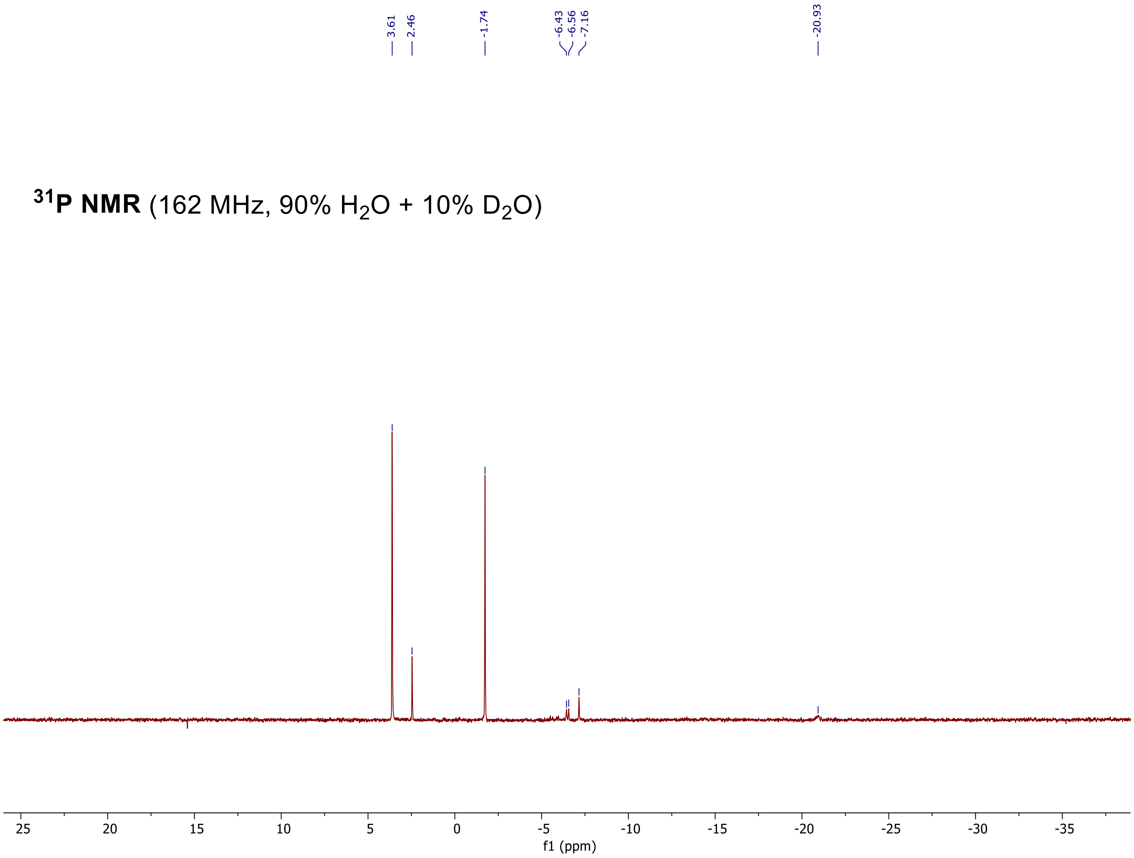

^{31}**P NMR** (162 MHz, 90% H_2O + 10% D_2O)

n(OTf⁻)	m (sample)	Yield of F⁻ %	Yield of PO_3F^{2-} %
0.058 mmol	51.5 mg	63%	29%

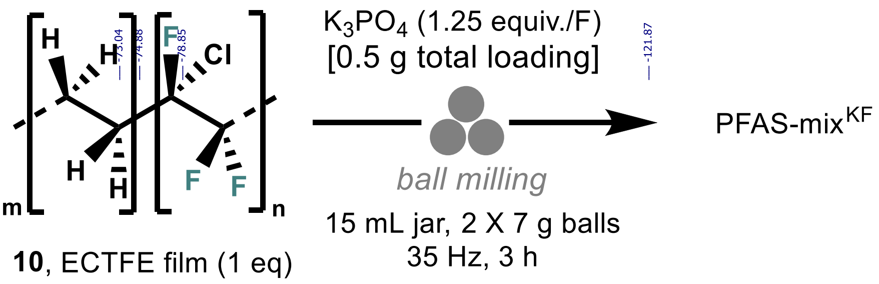

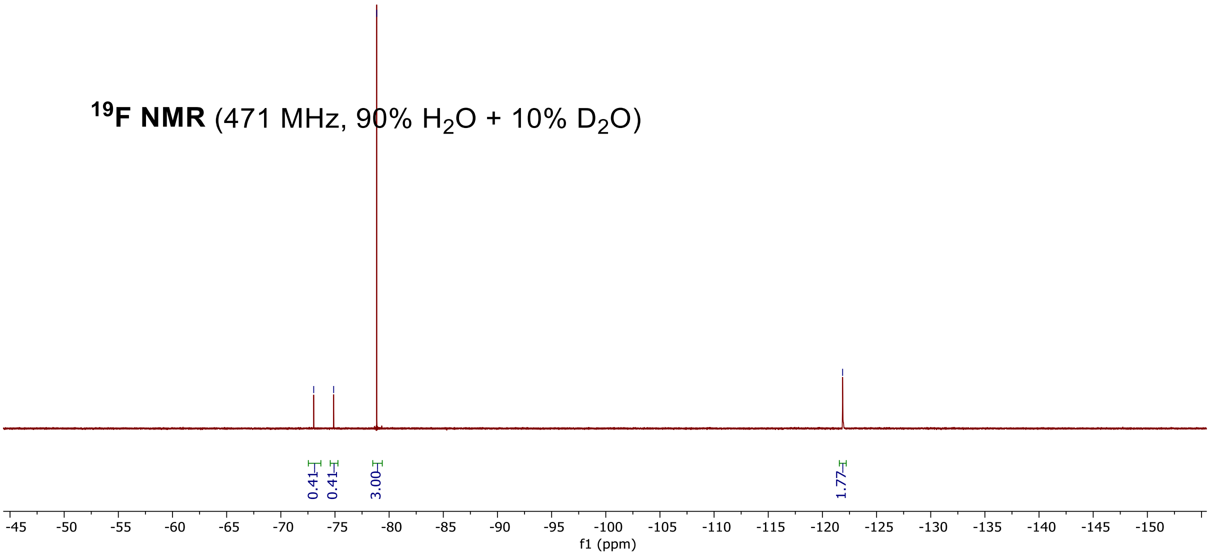

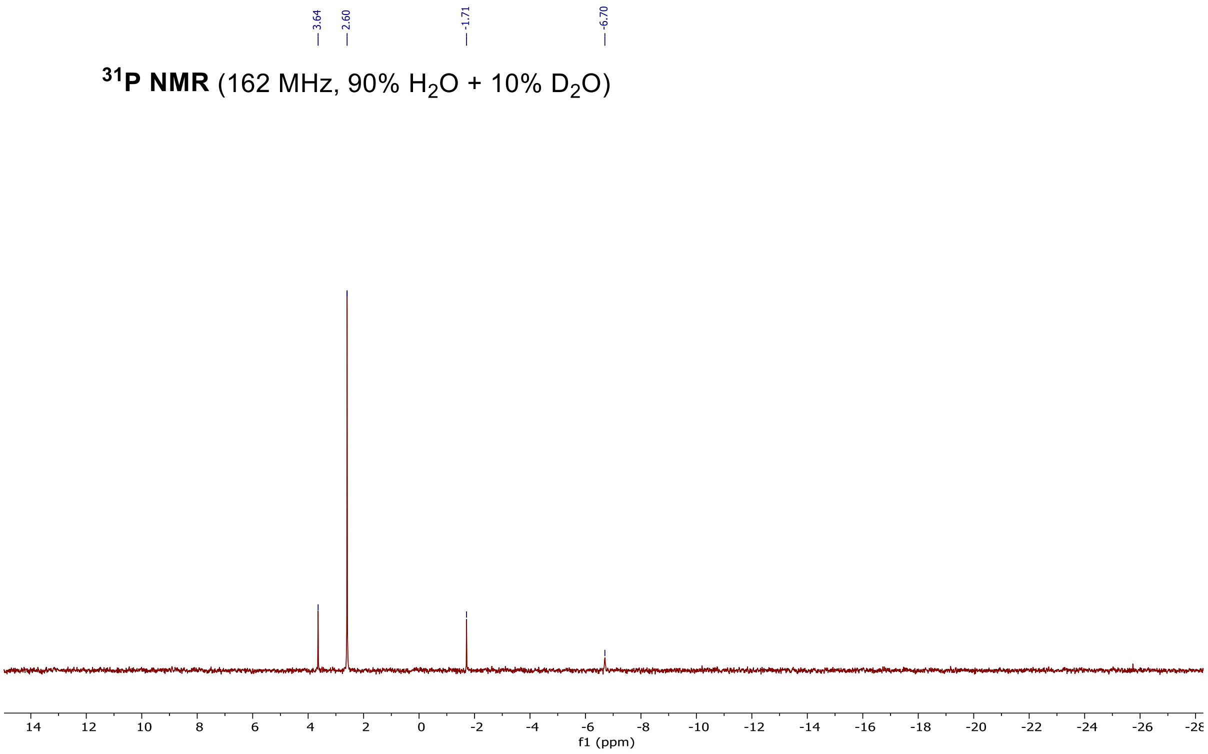

n(OTf⁻)	m (sample)	Yield of F⁻ %	Yield of PO_3F^{2-} %
0.058 mmol	51.2 mg	68%	7%

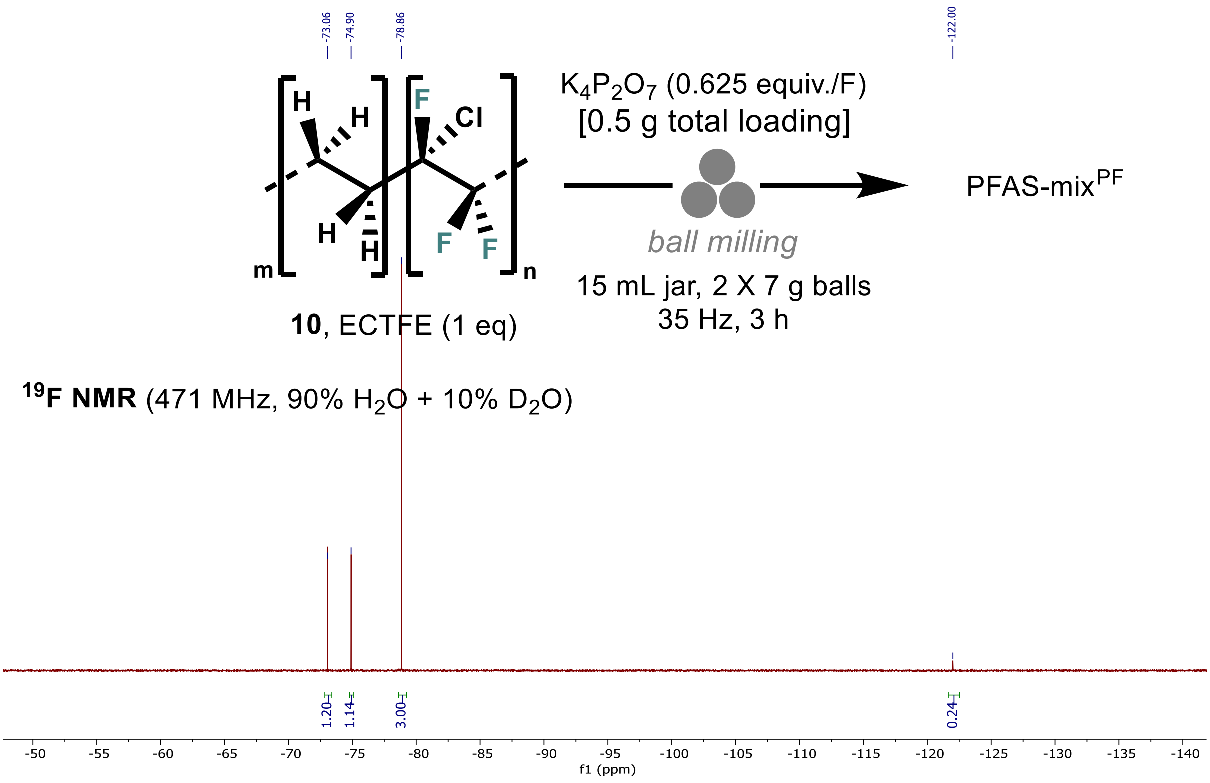

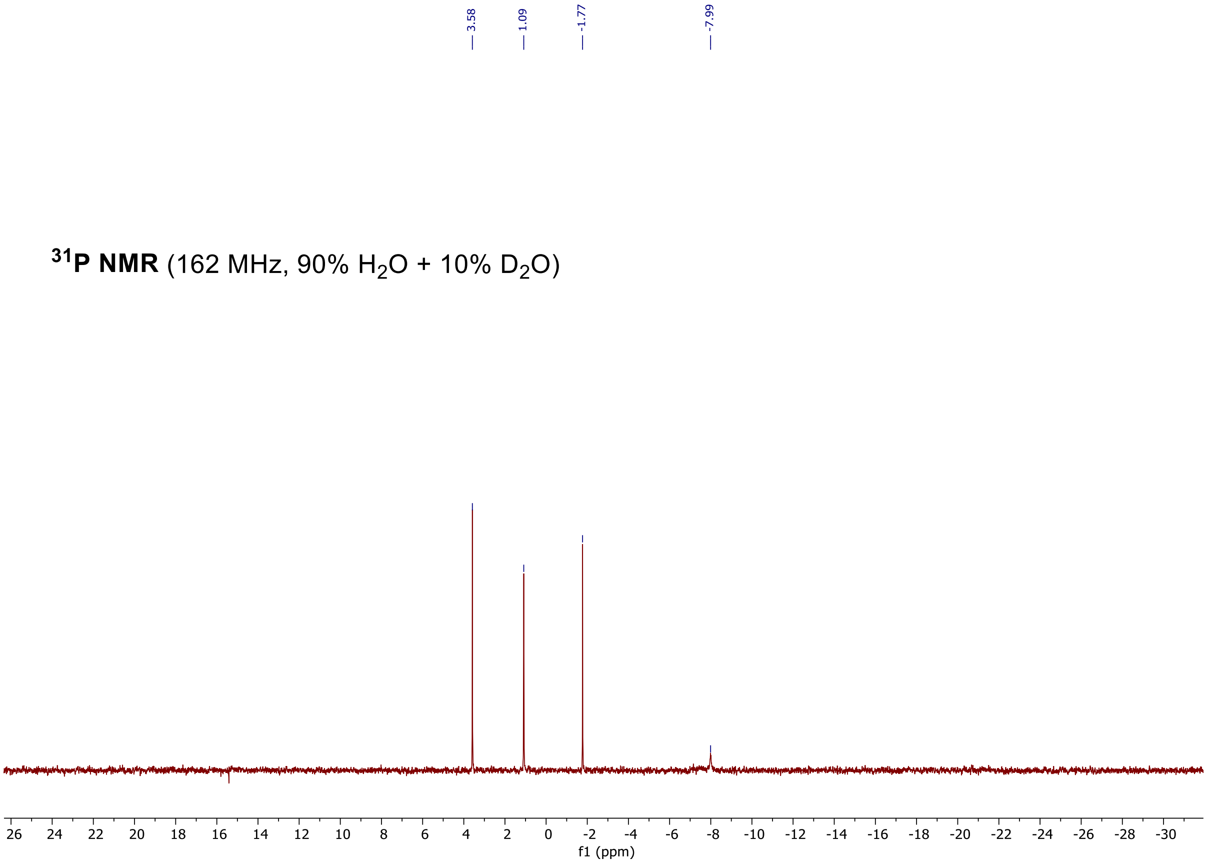

n(OTf^-)	m (sample)	Yield of F^- %	Yield of PO_3F^{2-} %
0.058 mmol	34.0 mg	91%	15%

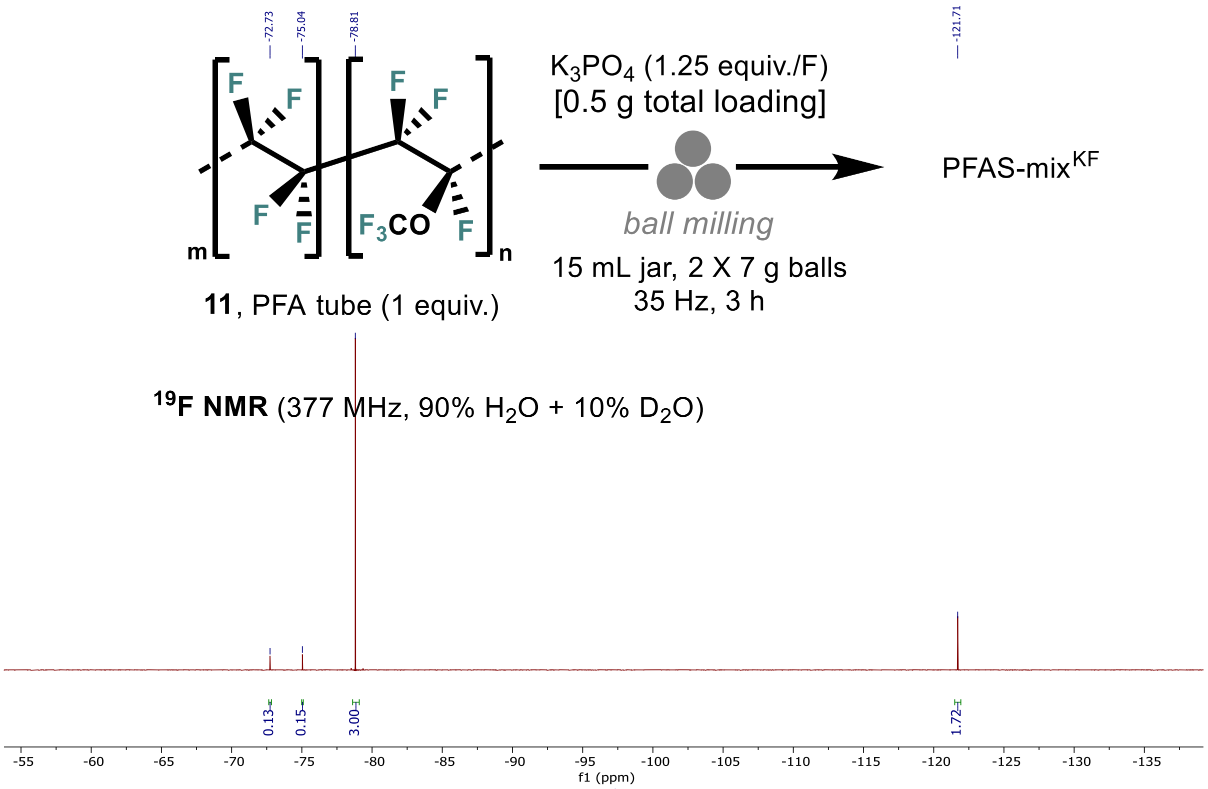

^{19}**F NMR** (377 MHz, 90% H_2O + 10% D_2O)

^{31}**P NMR** (162 MHz, 90% H_2O + 10% D_2O)

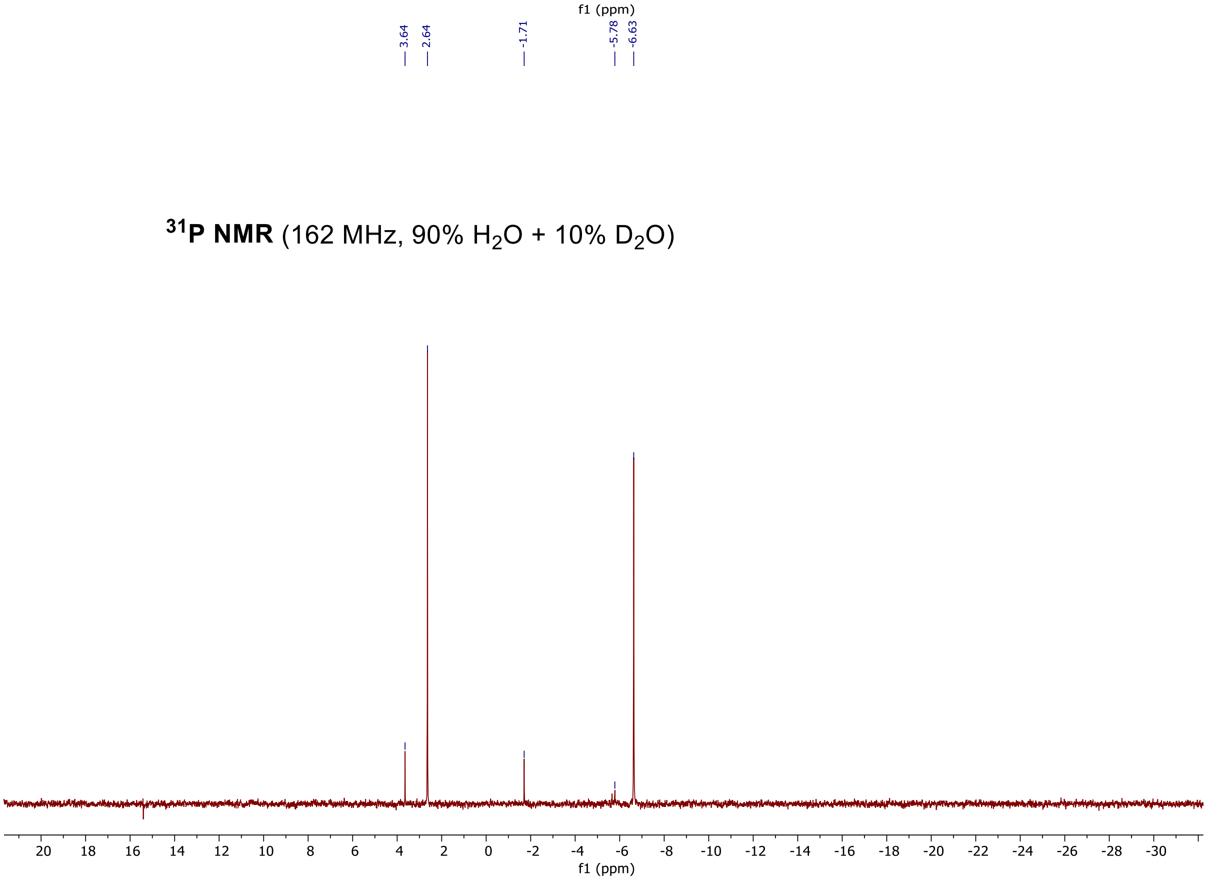

n(OTf⁻)	m (sample)	Yield of F^- %	Yield of PO_3F^{2-} %
0.058 mmol	50 mg	4%	quant.

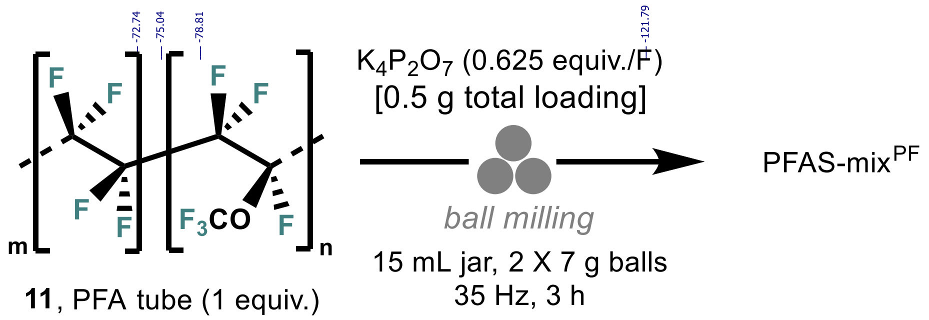

11, PFA tube (1 equiv.)

^{19}F NMR (377 MHz, 90% H_2O + 10% D_2O)

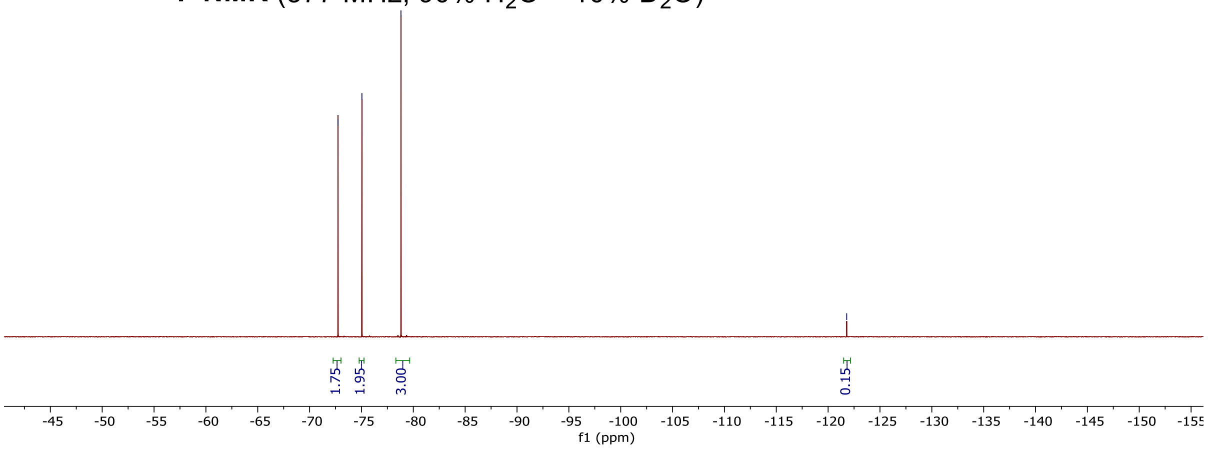

^{31}P NMR (162 MHz, 90% H_2O + 10% D_2O)

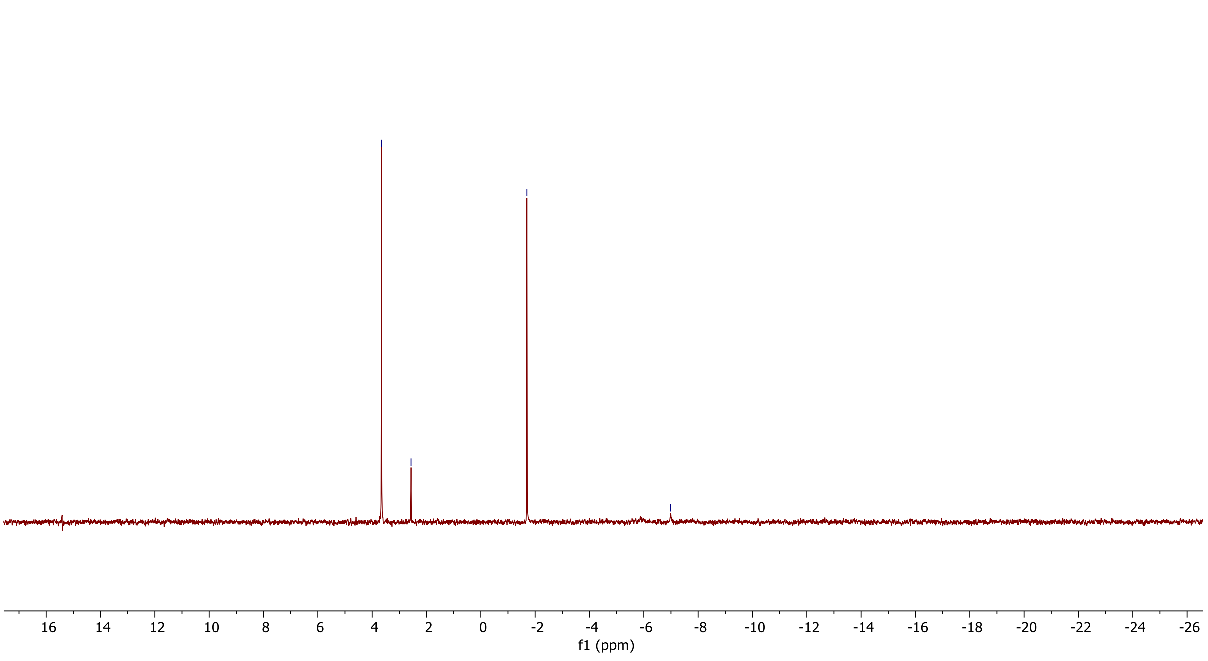

n(OTf⁻)	m (sample)	Yield of F^- %	Yield of PO_3F^{2-} %
0.058 mmol	70 mg	93%	5%

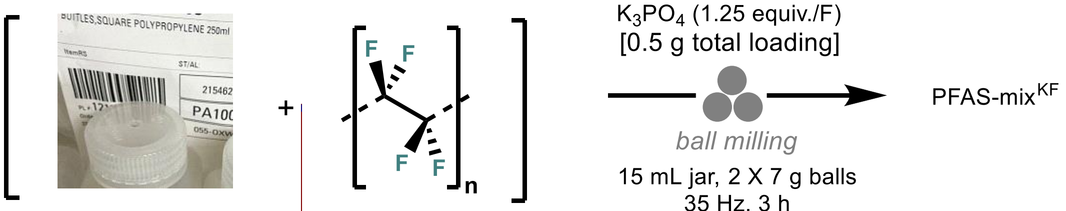

12, [*PP: 30 wt%* + *PTFE: 70 wt%*]

^{19}F NMR (377 MHz, 90% H_2O + 10% D_2O)

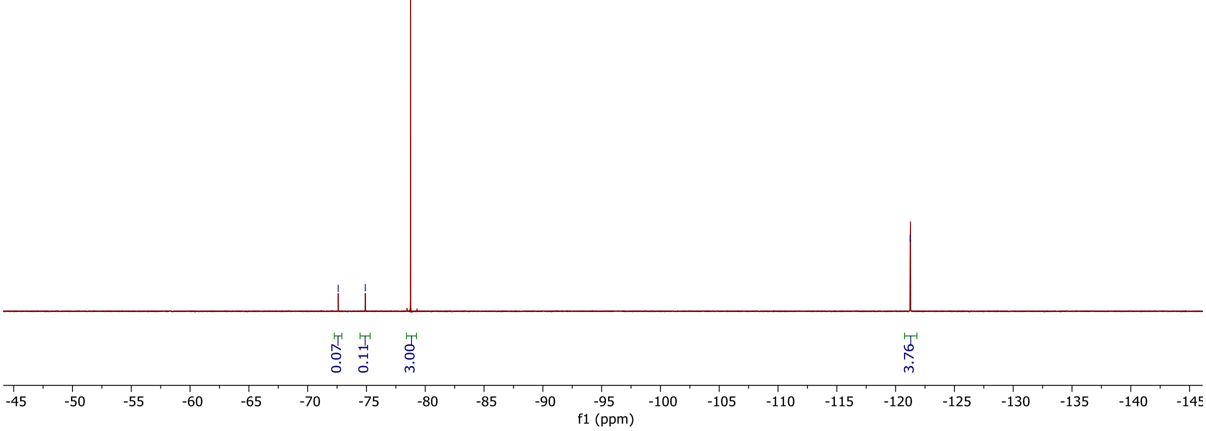

^{31}P NMR (162 MHz, 90% H_2O + 10% D_2O)

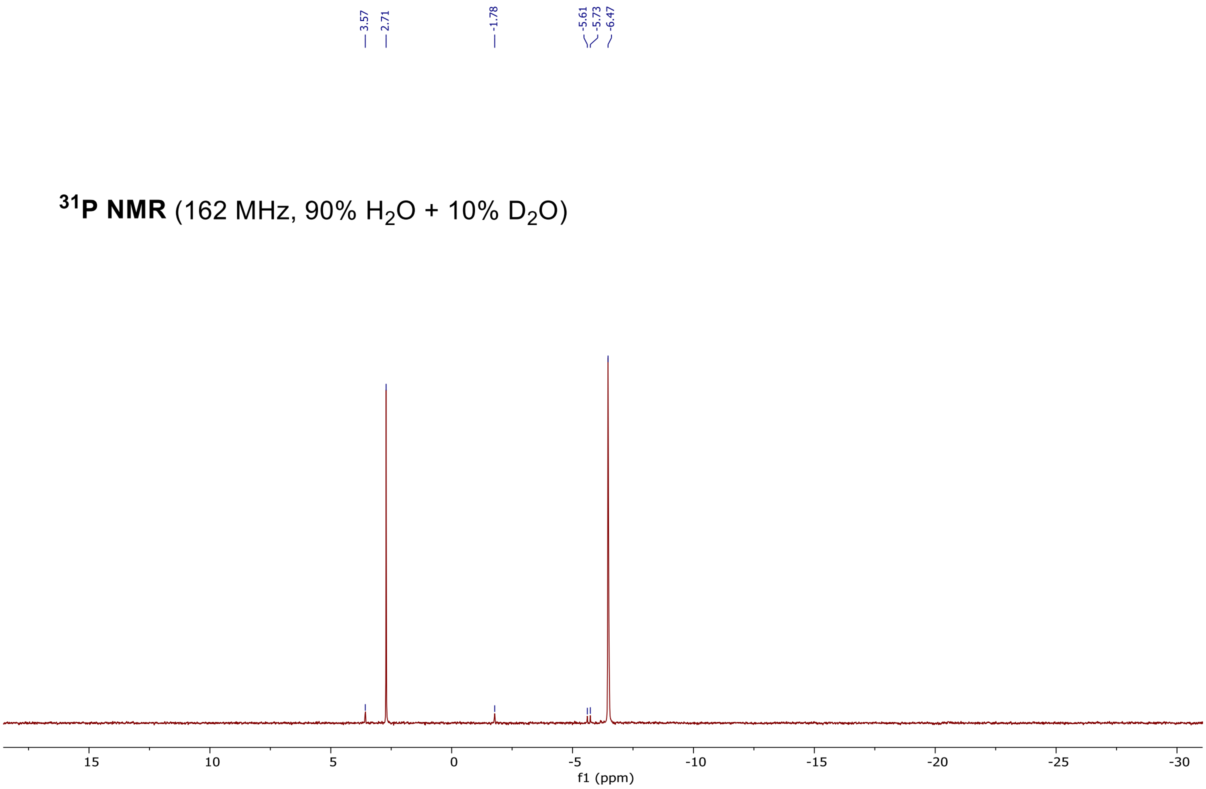

n(OTf⁻)	m (sample)	Yield of F⁻ %	Yield of PO_3F^{2-} %
0.058 mmol	57.0 mg	6%	53%

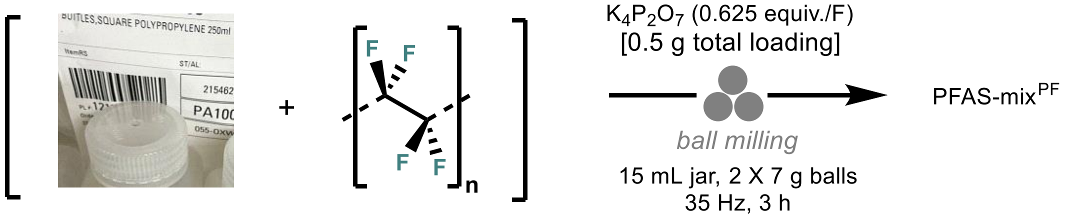

12, [*PP: 30 wt%* + *PTFE: 70 wt%*]

^{19}**F NMR** (377 MHz, 90% H_2O + 10% D_2O)

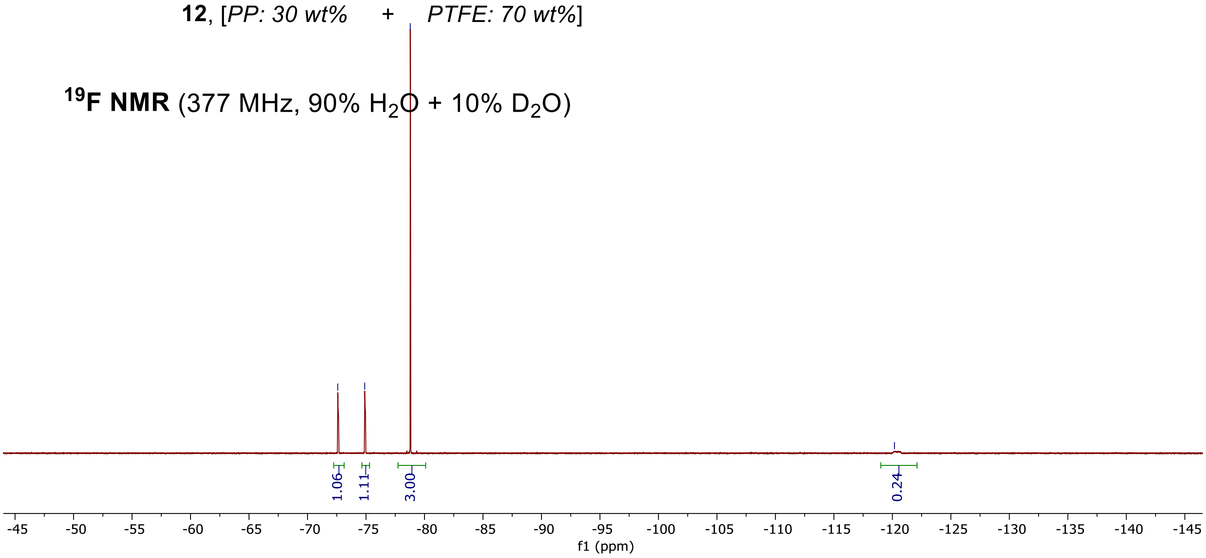

^{31}**P NMR** (162 MHz, 90% H_2O + 10% D_2O)

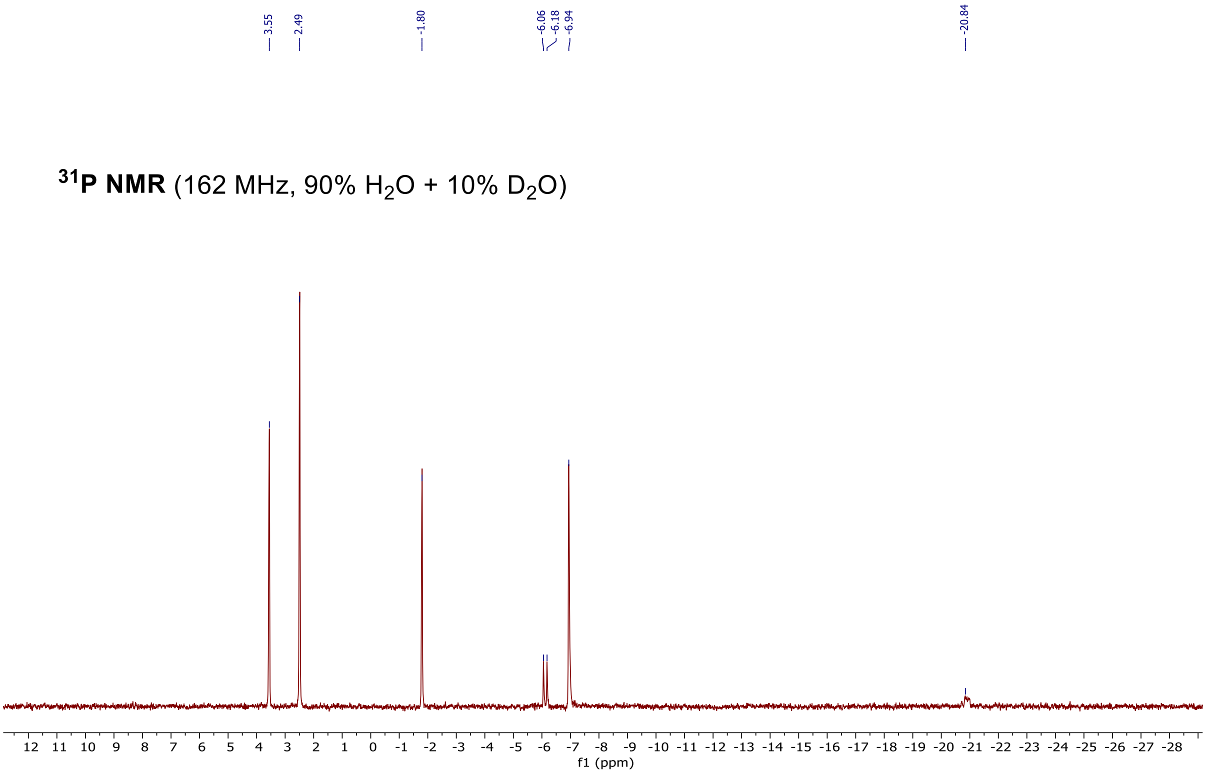

n(OTf⁻)	m (sample)	Yield of F^- %	Yield of PO_3F^{2-} %
0.058 mmol	55.3 mg	45%	2%

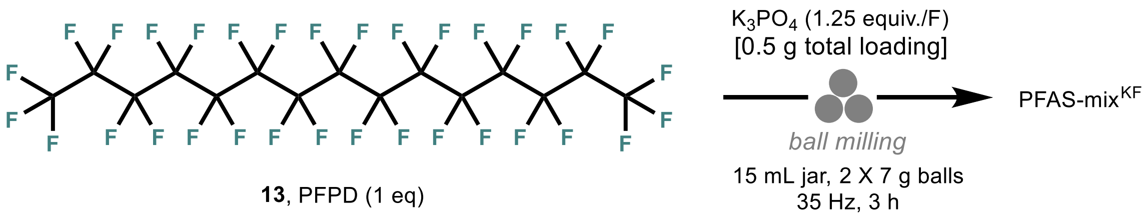

13, PFPD (1 eq)

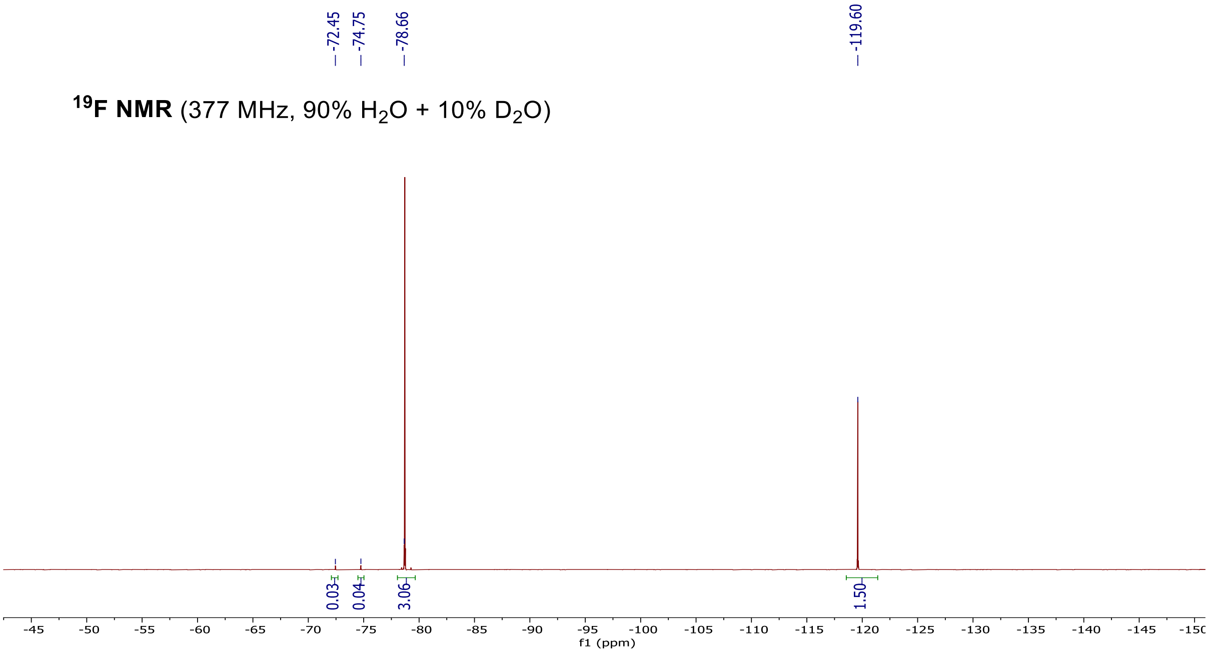

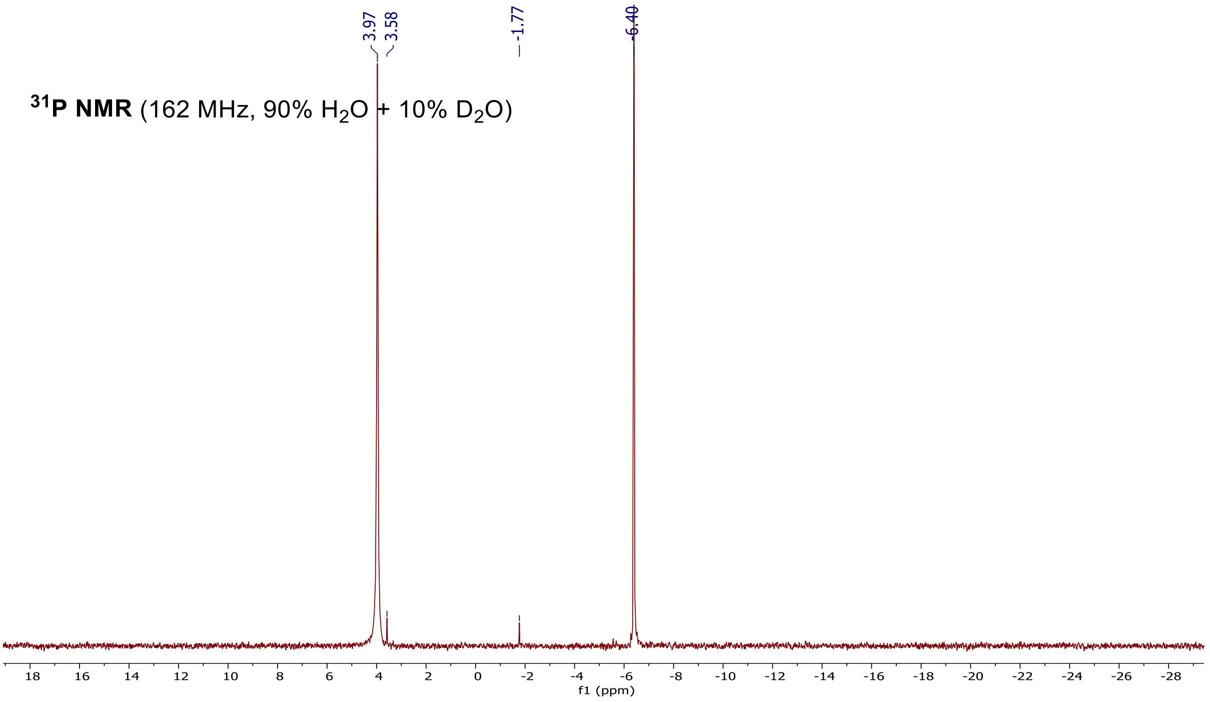

n(OTf^-)	m (sample)	Yield of F^- %	Yield of PO_3F^{2-} %
0.059 mmol	59.4 mg	7%	12%

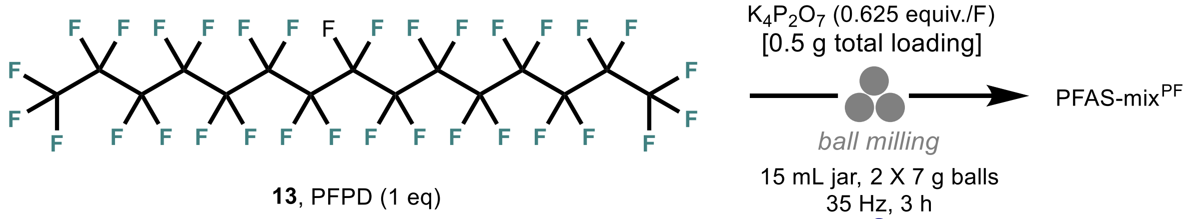

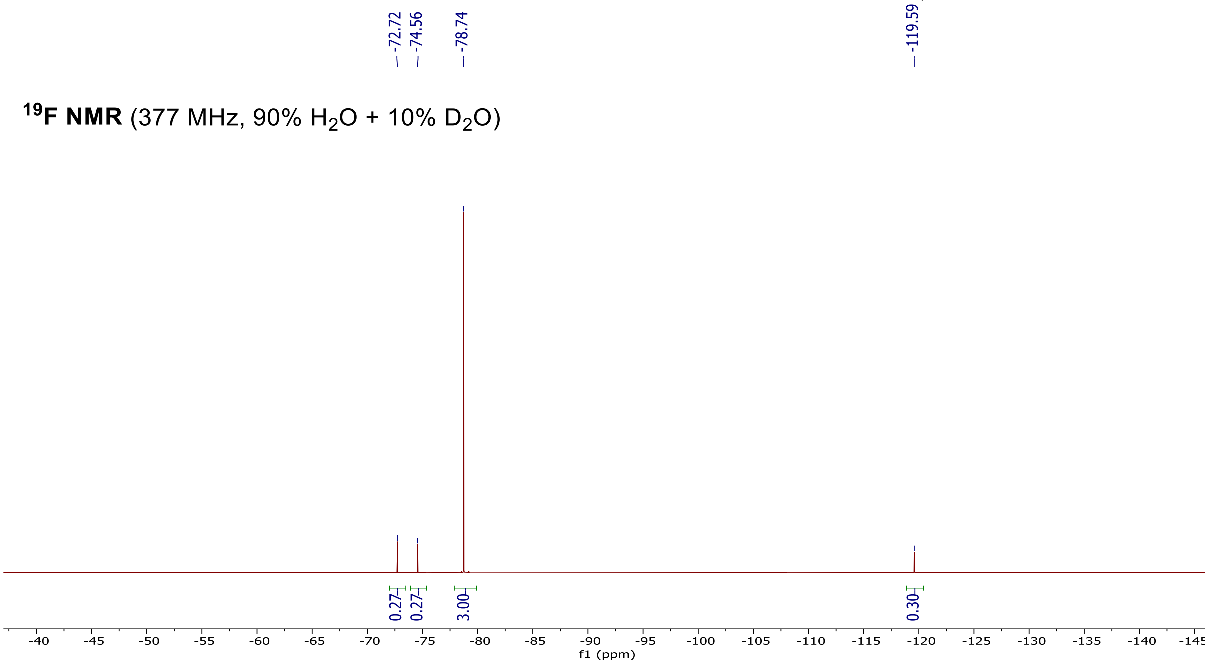

^{19}F NMR (377 MHz, 90% H_2O + 10% D_2O)

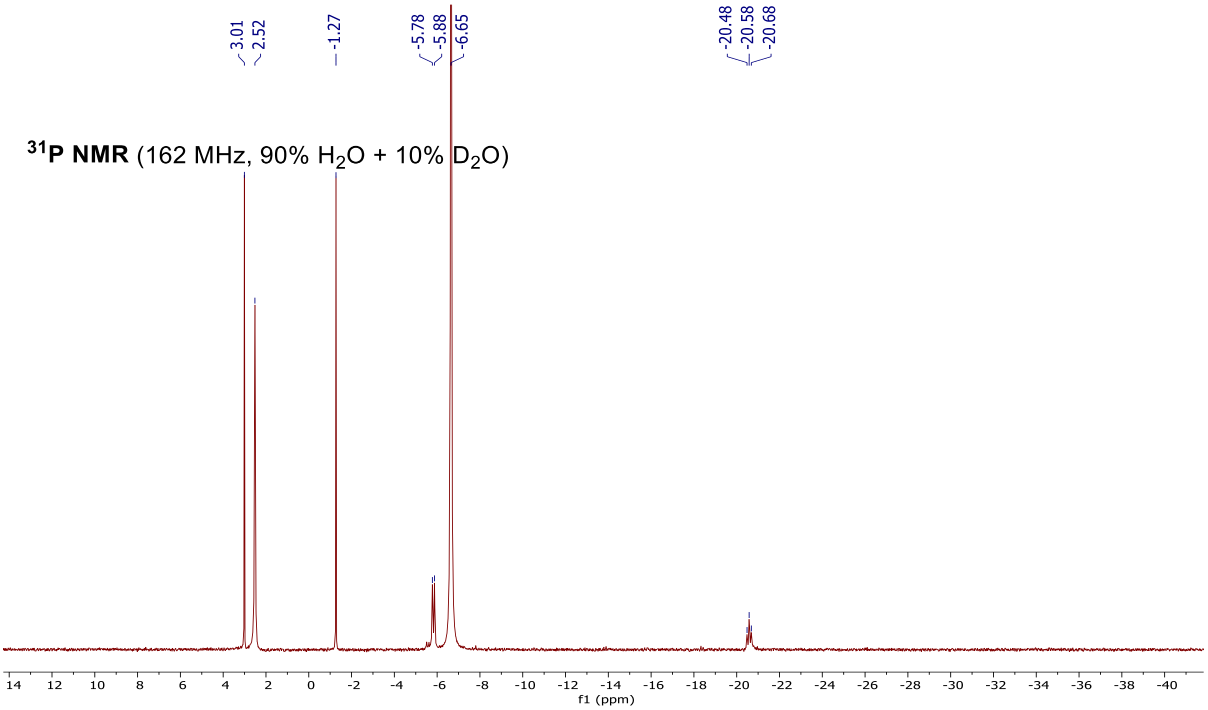

^{31}P NMR (162 MHz, 90% H_2O + 10% D_2O)

n(OTf⁻)	m (sample)	Yield of F⁻ %	Yield of PO_3F^{2-} %
0.054 mmol	49.6 mg	78%	20%

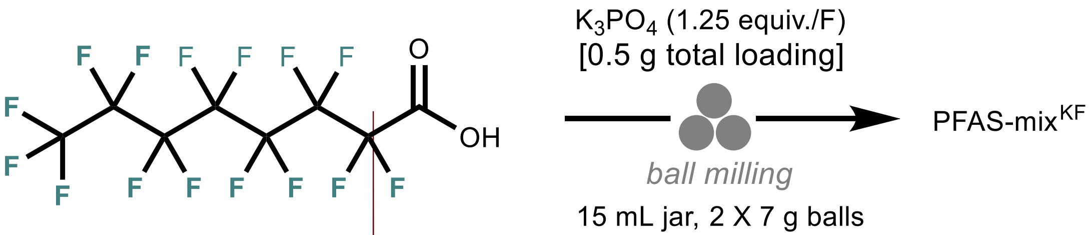

14, PFOA (1 eq)

^{19}F NMR (377 MHz, 90% H_2O + 10% D_2O)

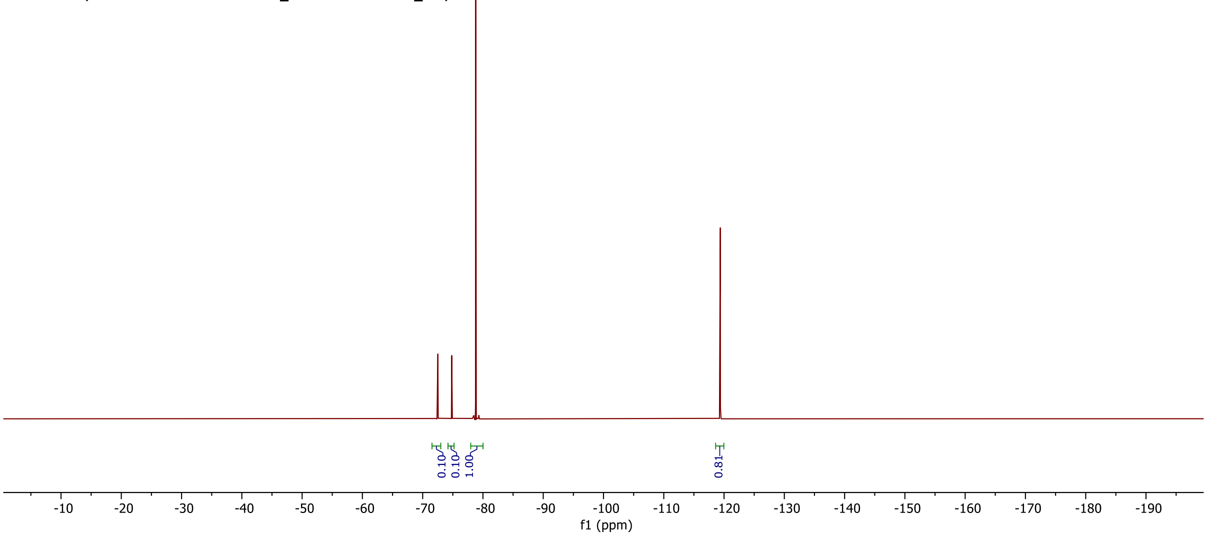

^{31}P NMR (162 MHz, 90% H_2O + 10% D_2O)

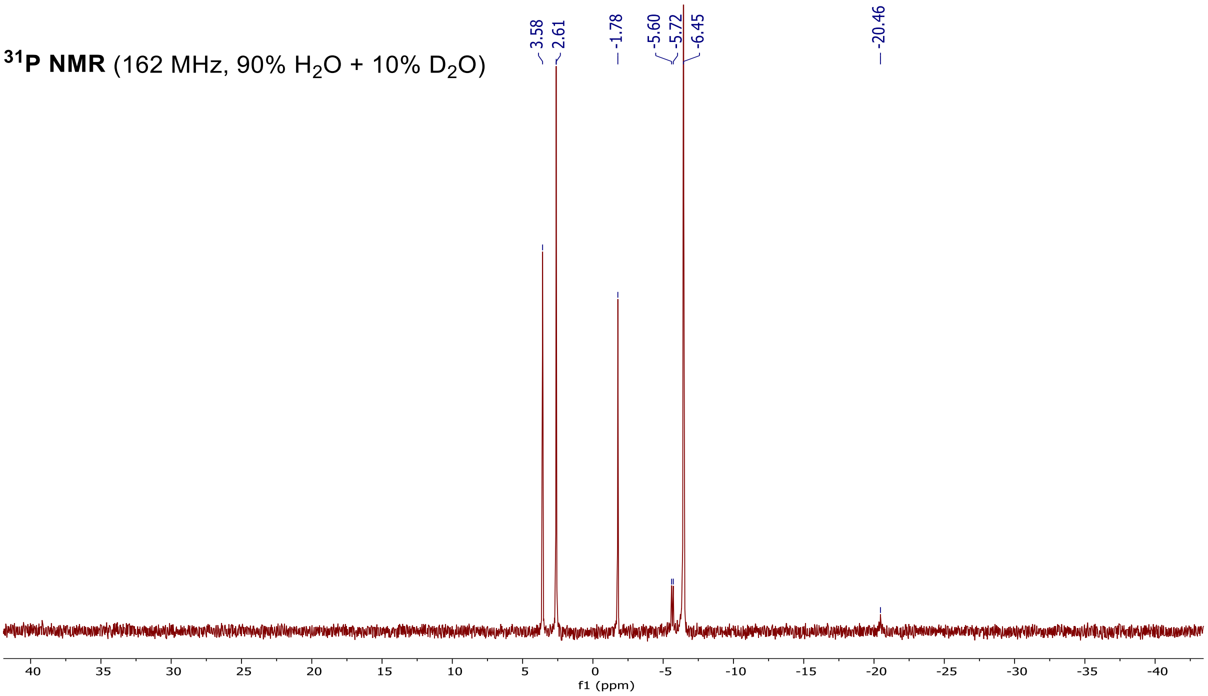

n(OTf⁻)	m (sample)	Yield of F⁻ %	Yield of PO_3F^{2-} %
0.028 mmol	22.4 mg	14%	74%

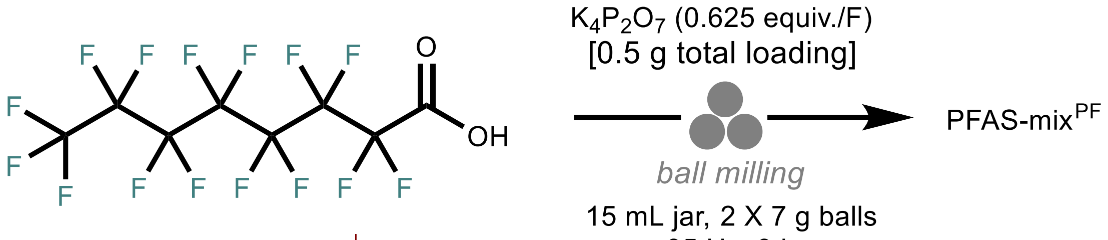

14, PFOA (1 eq)

^{19}F NMR (377 MHz, 90% H_2O + 10% D_2O)

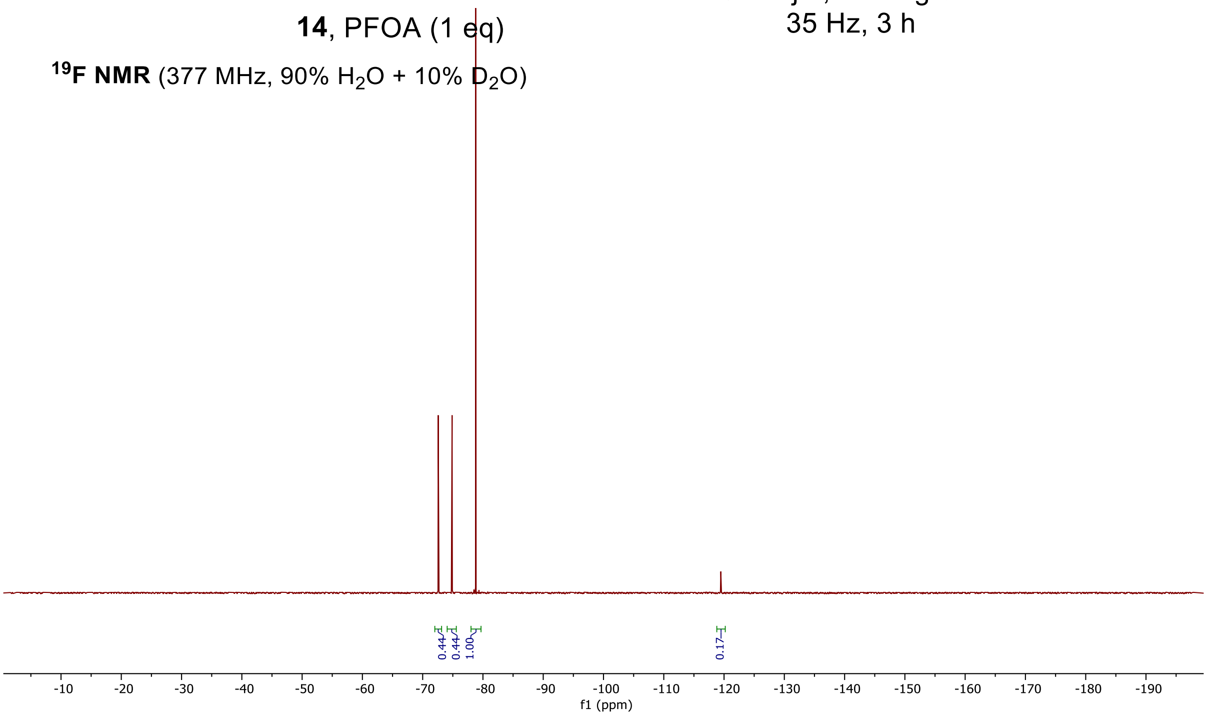

^{31}P NMR (162 MHz, 90% H_2O + 10% D_2O)

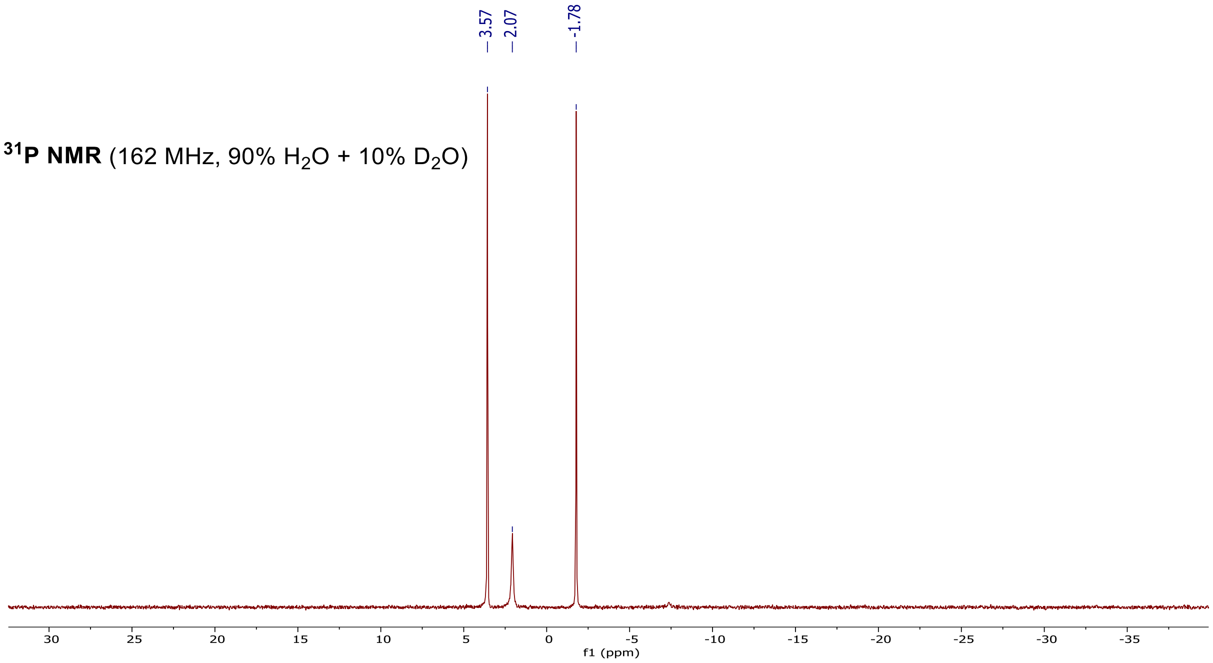

n(OTf⁻)	m (sample)	Yield of F⁻ %	Yield of PO_3F^{2-} %
0.084 mmol	70.8 mg	73%	26%

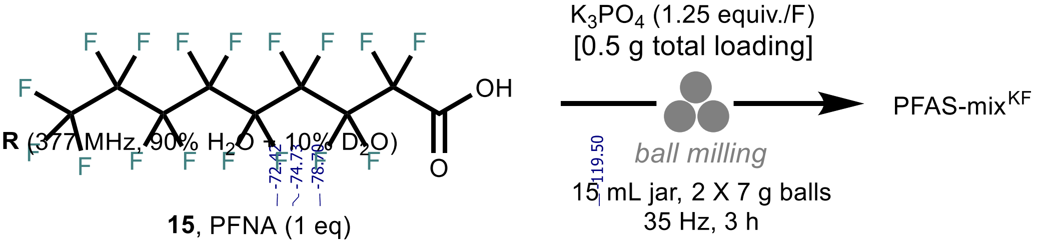

15, PFNA (1 eq)

^{19}F NMR (377 MHz, 90% H_2O + 10% D_2O)

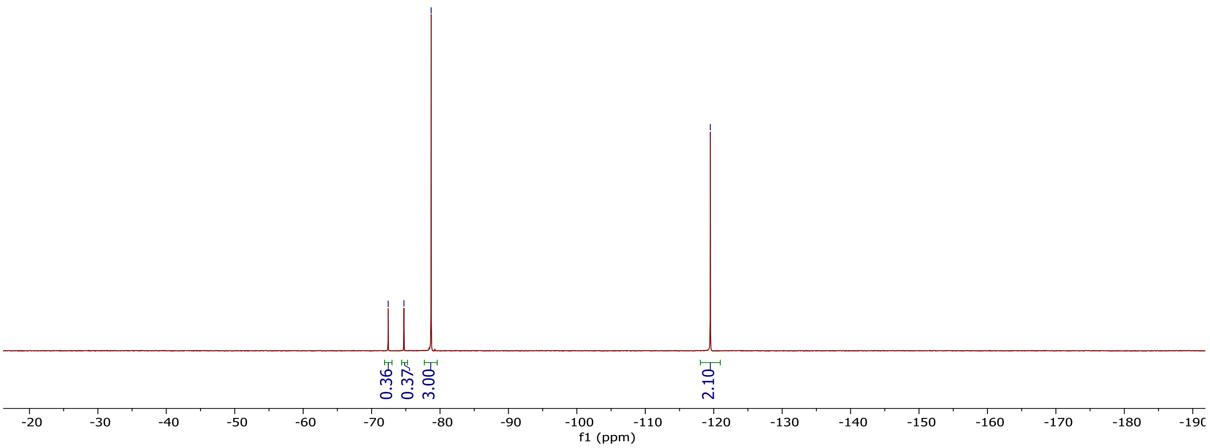

^{31}P NMR (162 MHz, 90% H_2O + 10% D_2O)

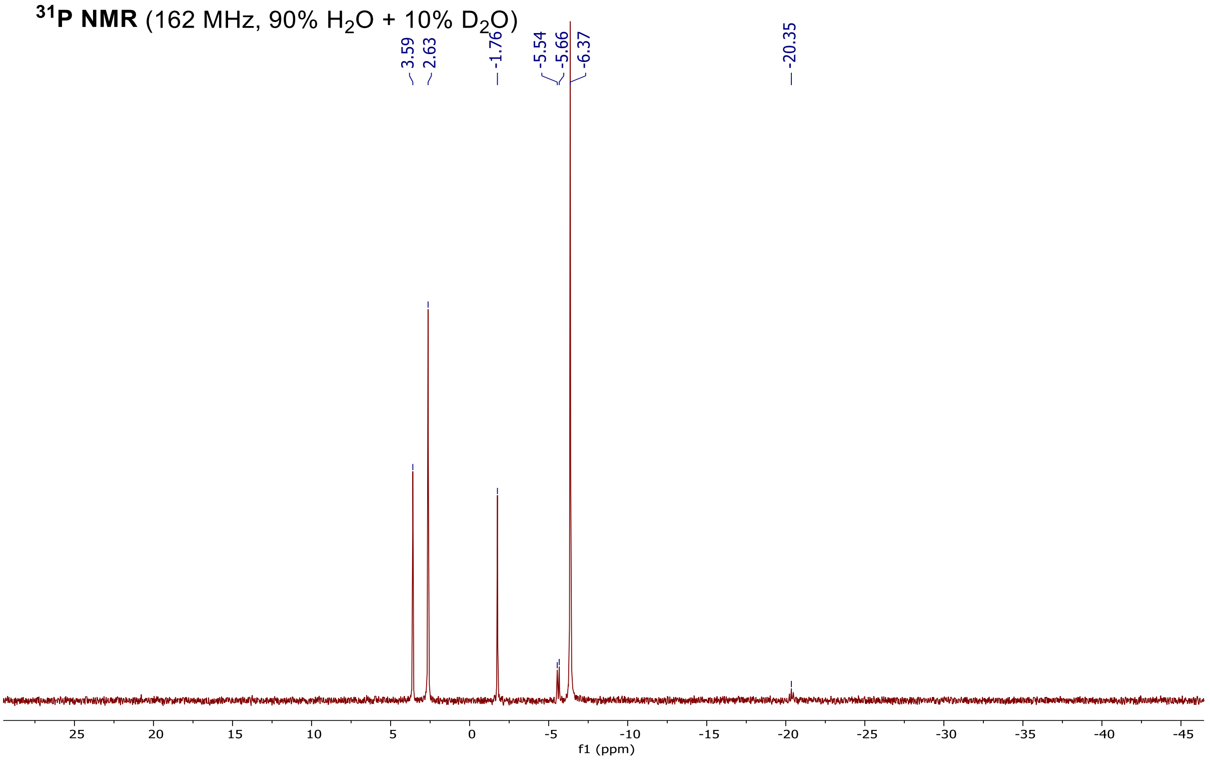

n(OTf^-)	m (sample)	Yield of F^- %	Yield of PO_3F^{2-} %
0.075 mmol	58.5 mg	6%	93%

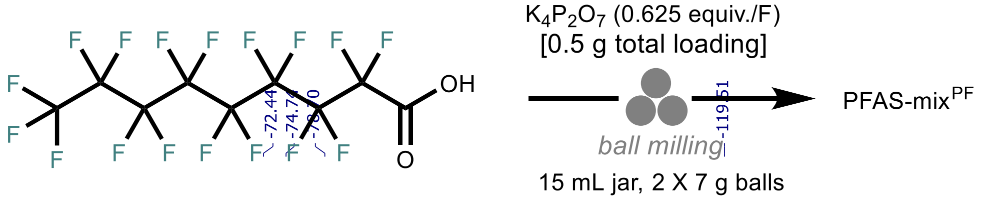

15, PFNA (1 eq)

^{19}F NMR (377 MHz, 90% H_2O + 10% D_2O)

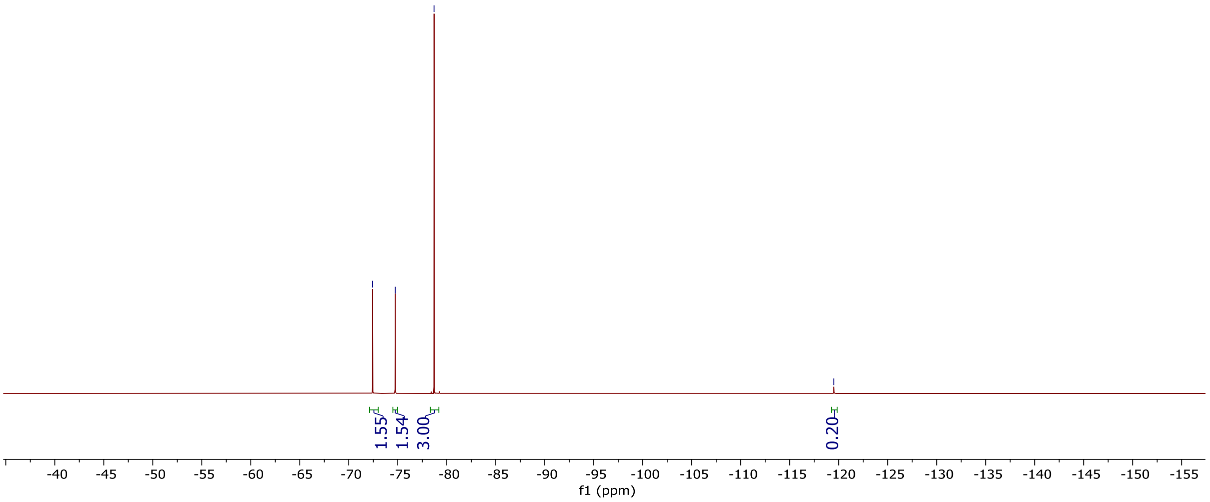

^{31}P NMR (162 MHz, 90% H_2O + 10% D_2O)

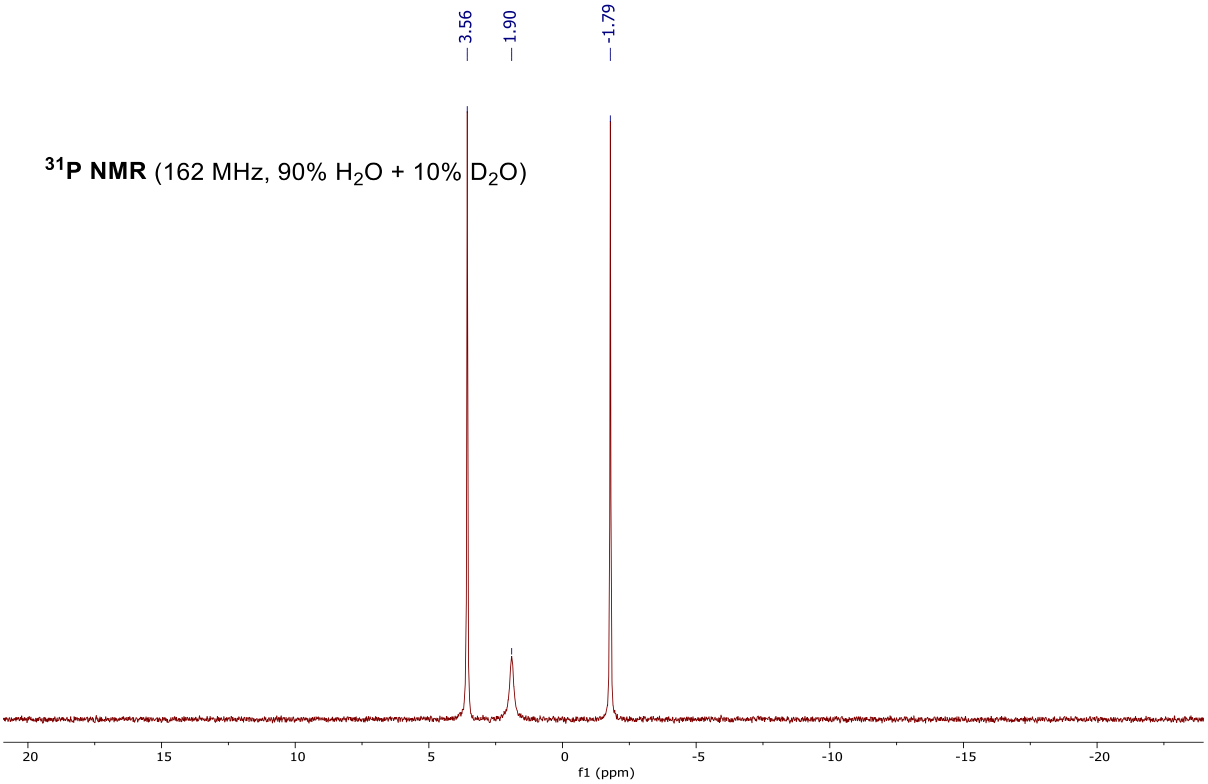

n(OTf⁻)	m (sample)	Yield of F^- %	Yield of PO_3F^{2-} %
0.058 mmol	60.5 mg	74%	26%

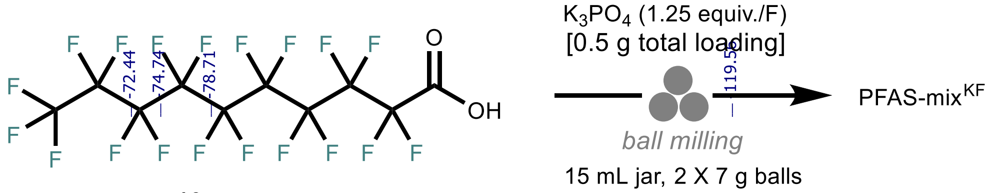

16, PFDA (1 eq)

^{19}F NMR (377 MHz, 90% H_2O + 10% D_2O)

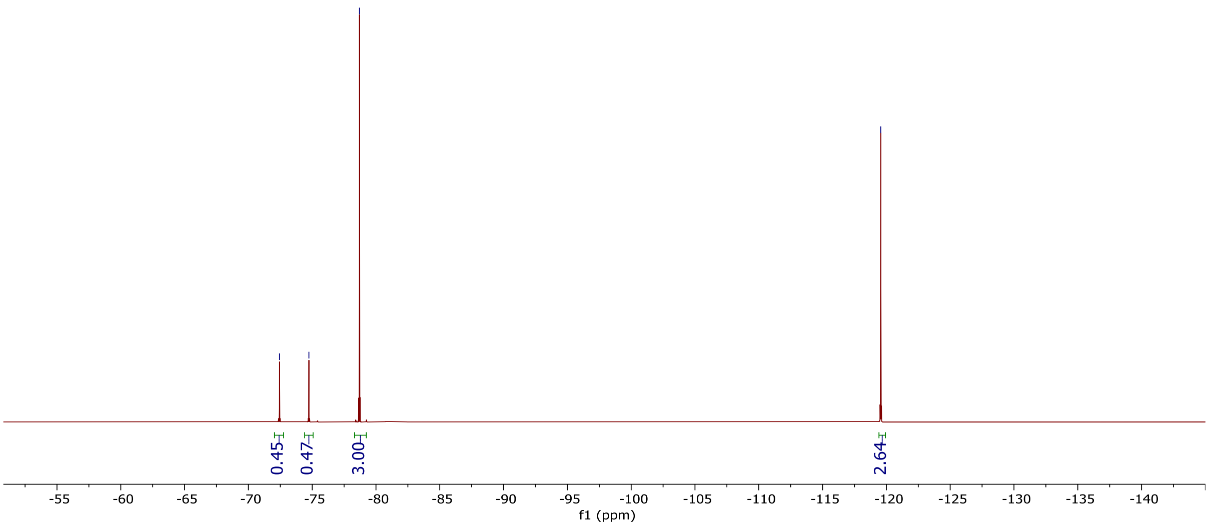

^{31}P NMR (162 MHz, 90% H_2O + 10% D_2O)

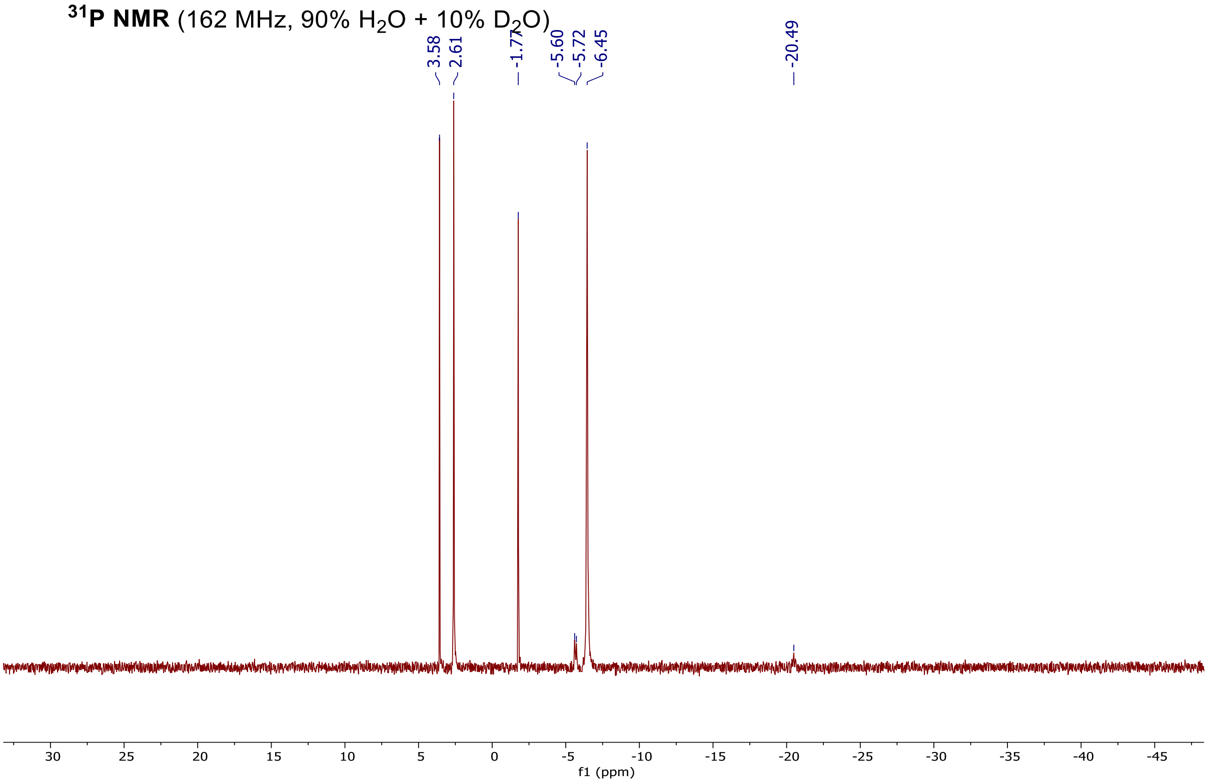

n(OTf⁻)	m (sample)	Yield of F⁻ %	Yield of PO_3F^{2-} %
0.058 mmol	54.7 mg	3%	76%

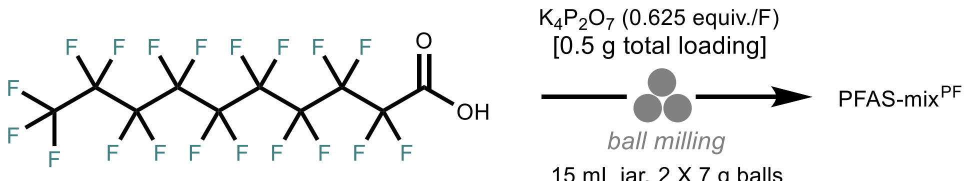

16, PFDA (1 eq)

[19]**F NMR** (377 MHz, 90% H_2O + 10% D_2O)

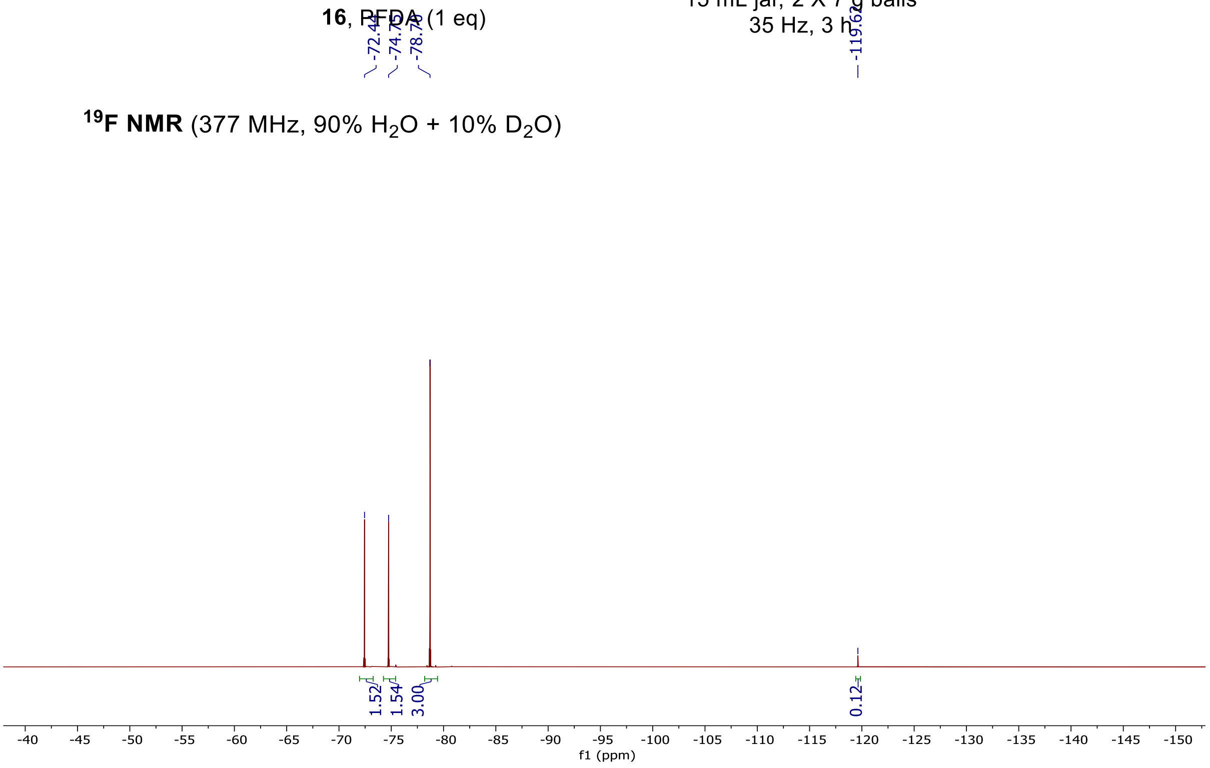

[31]**P NMR** (162 MHz, 90% H_2O + 10% D_2O)

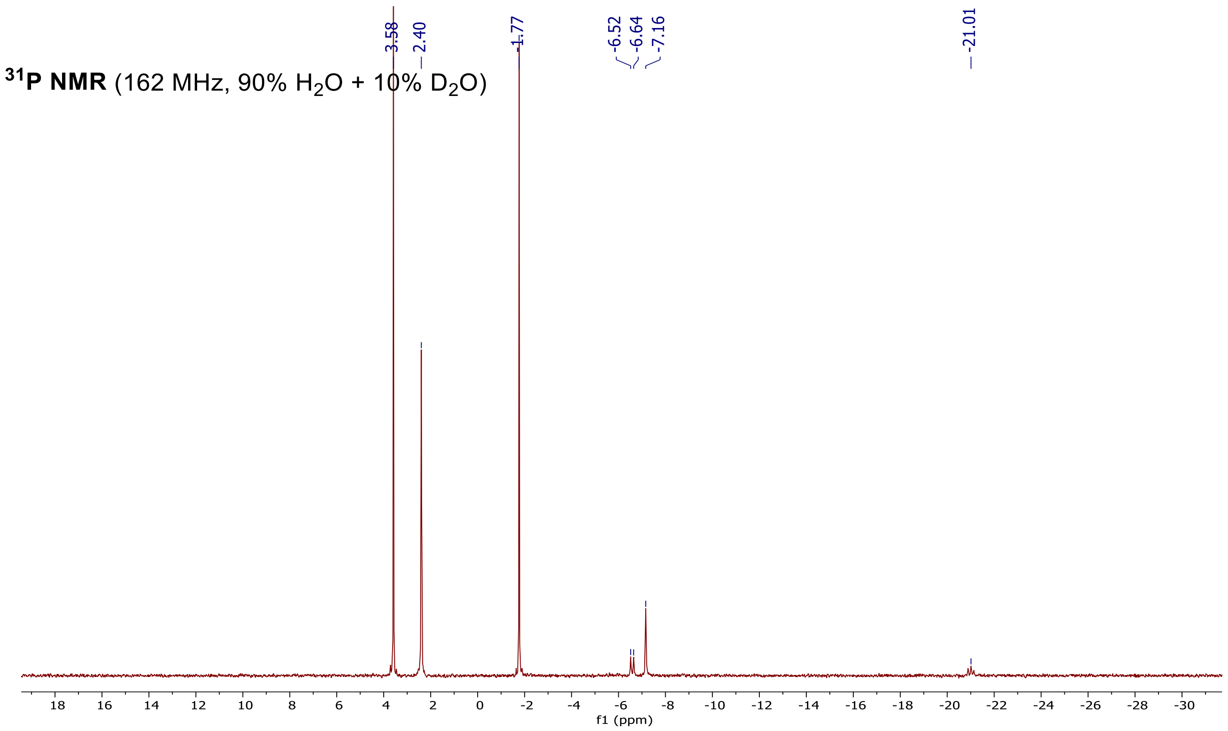

n(OTf⁻)	m (sample)	Yield of F⁻ %	Yield of PO_3F^{2-} %
0.058 mmol	58.4 mg	75%	25%

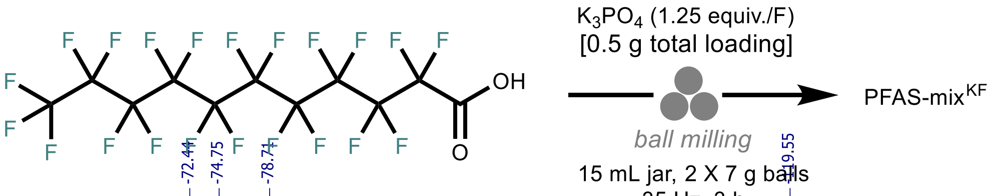

17, PFUnDA (1 eq)

^{19}F NMR (377 MHz, 90% H_2O + 10% D_2O)

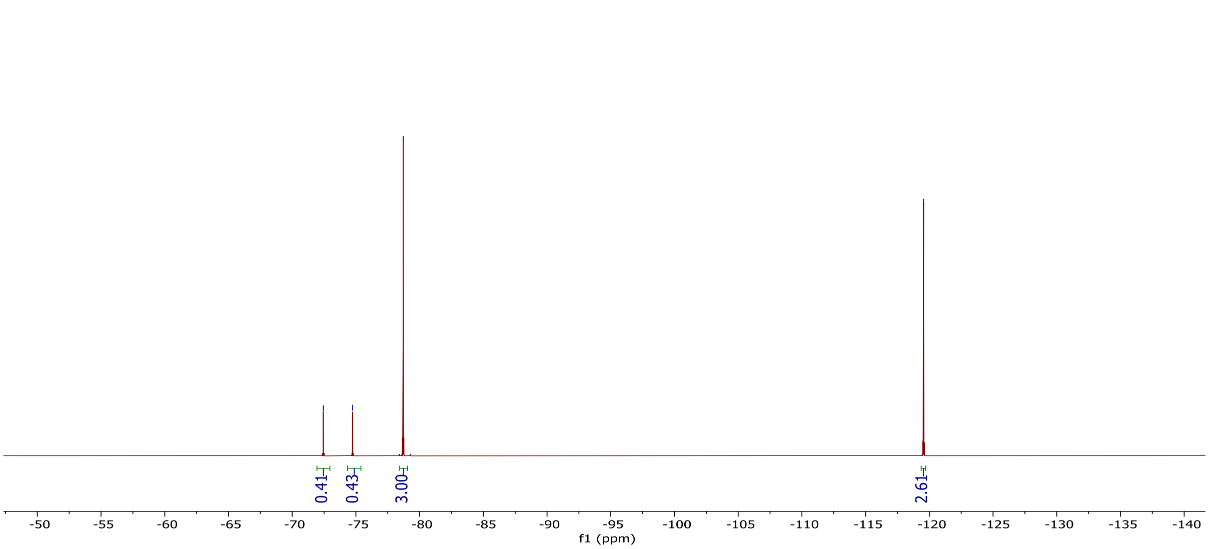

^{31}P NMR (162 MHz, 90% H_2O + 10% D_2O)

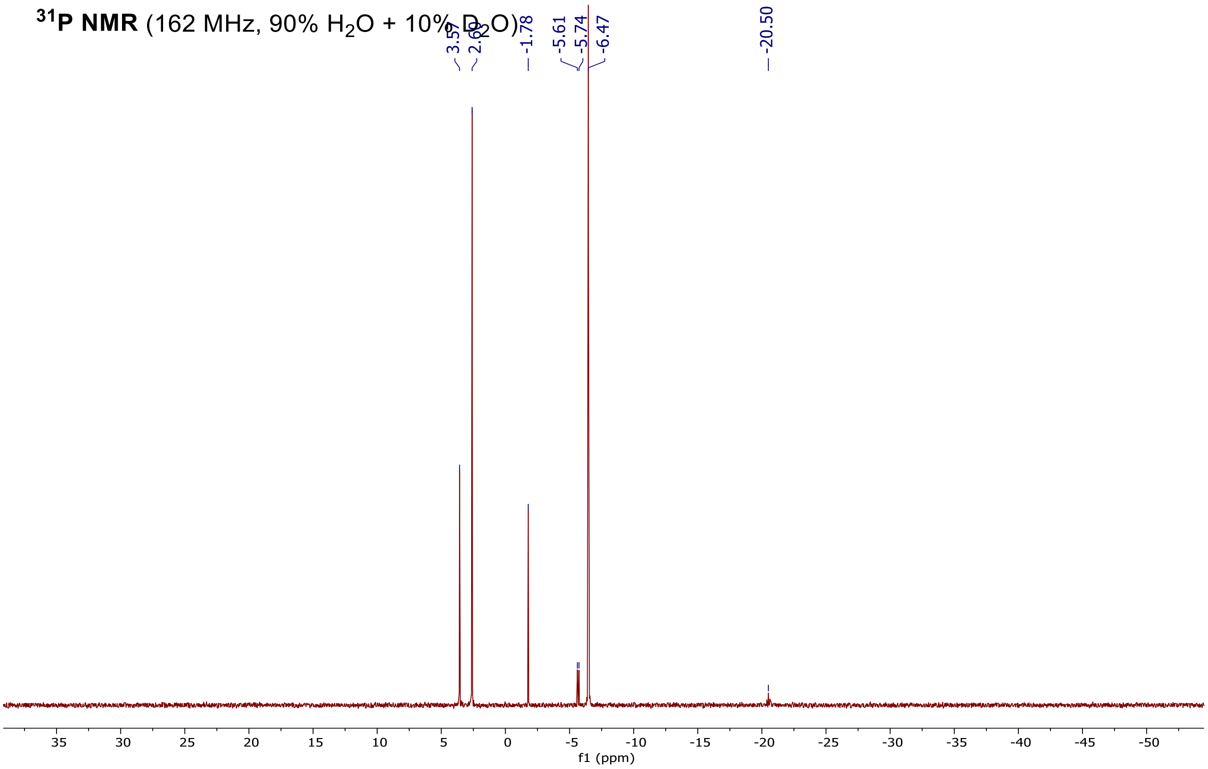

n(OTf⁻)	m (sample)	Yield of F^- %	Yield of PO_3F^{2-} %
0.058 mmol	48.9 mg	3%	69%

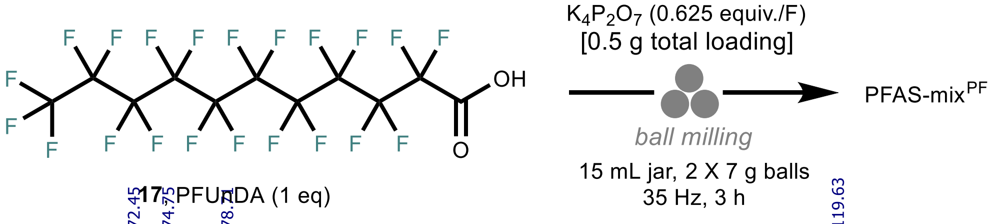

^{19}**F NMR** (377 MHz, 90% H_2O + 10% D_2O)

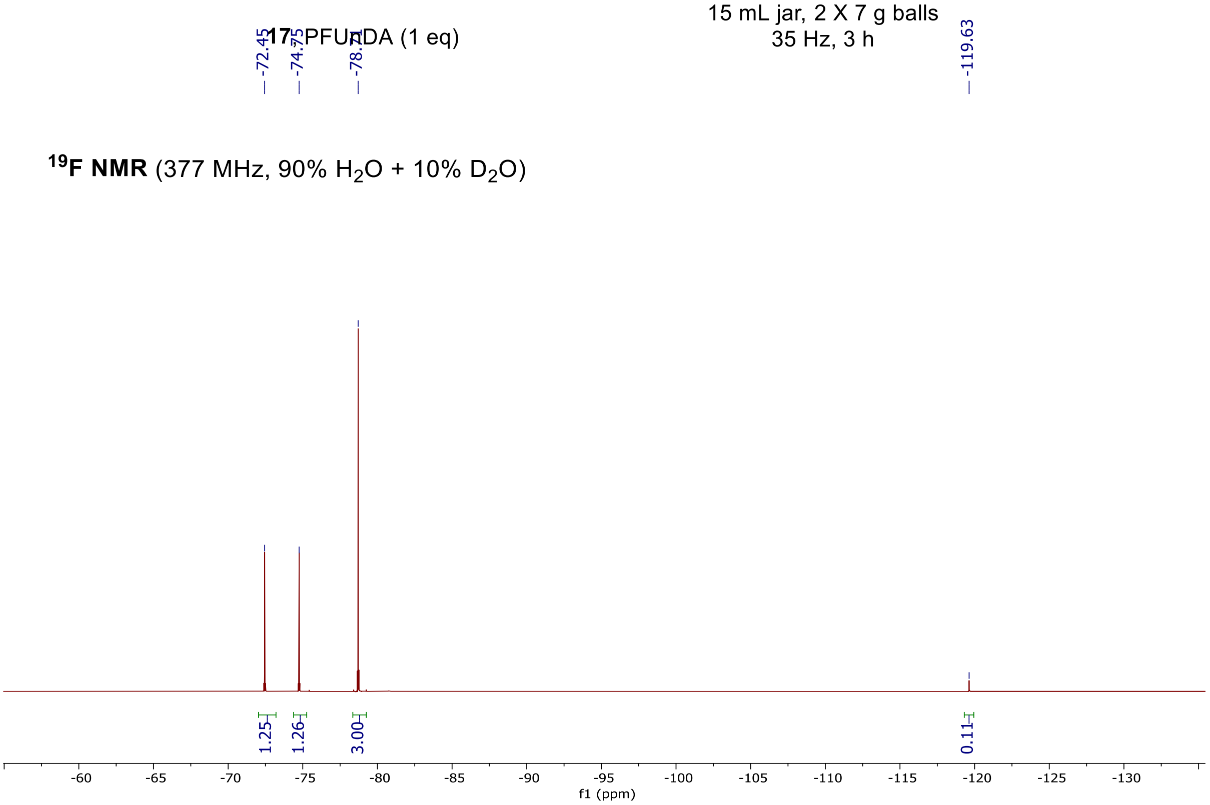

^{31}**P NMR** (162 MHz, 90% H_2O + 10% D_2O)

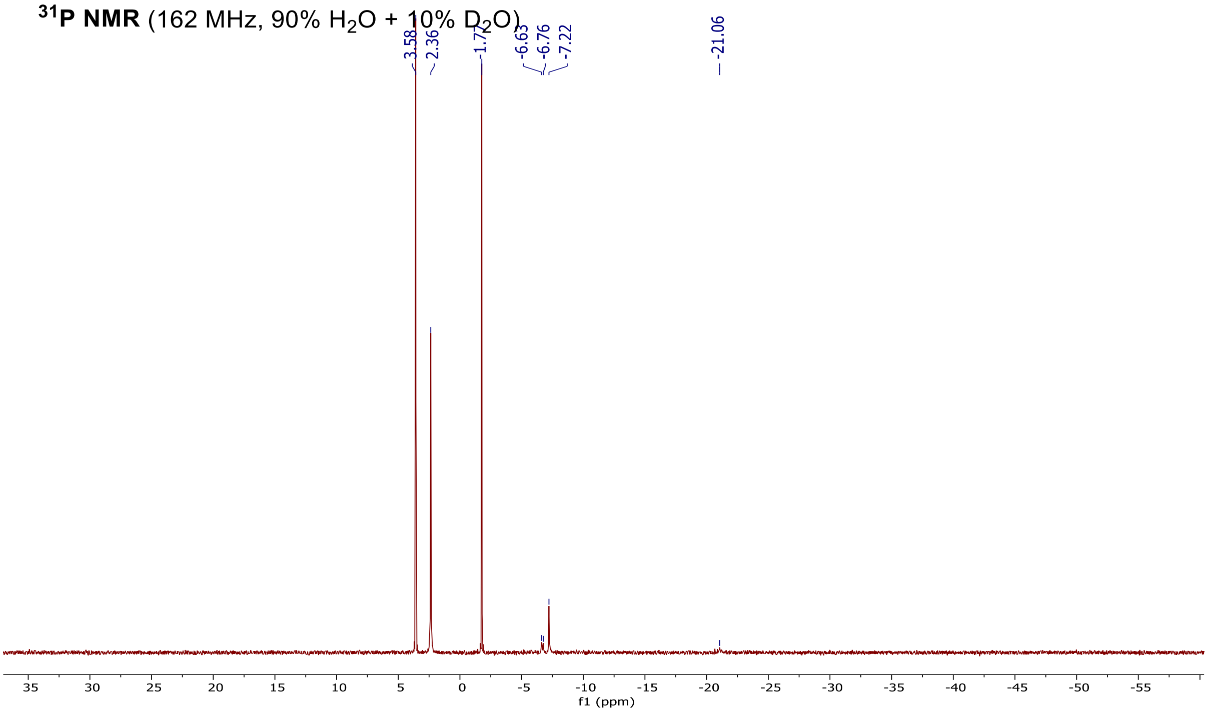

n(OTf^-)	m (sample)	Yield of F^- %	Yield of PO_3F^{2-} %
0.058 mmol	56.8 mg	74%	24%

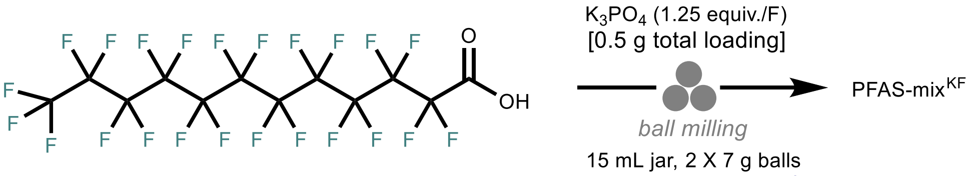

18, PFDoDA (1 eq)

^{19}F NMR (377 MHz, 90% H_2O + 10% D_2O)

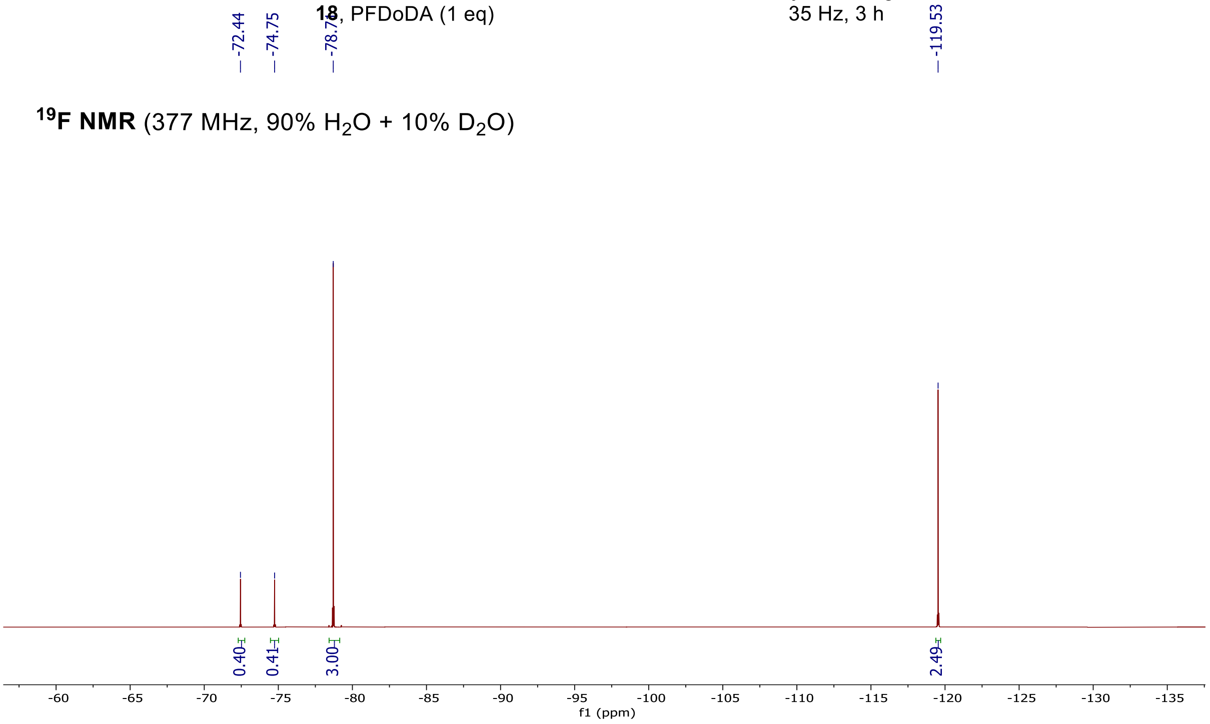

^{31}P NMR (162 MHz, 90% H_2O + 10% D_2O)

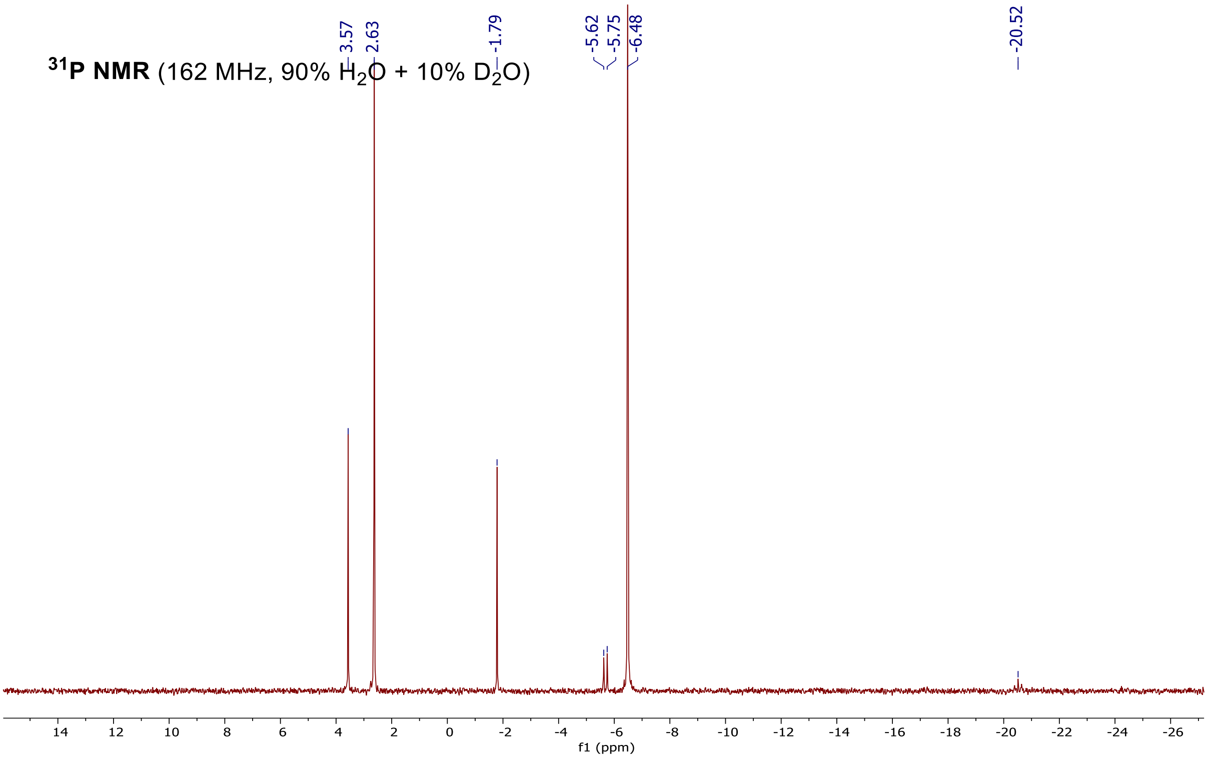

n(OTf⁻)	m (sample)	Yield of F⁻ %	Yield of PO_3F^{2-} %
0.058 mmol	50 mg	4%	84%

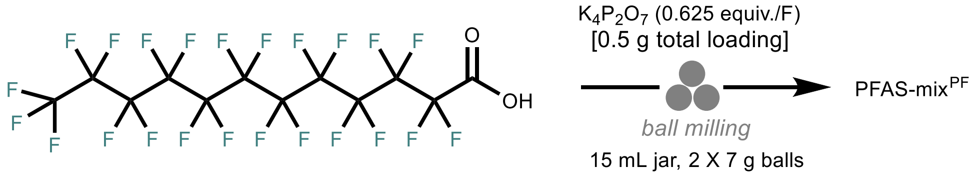

18, PFDoDA (1 eq)

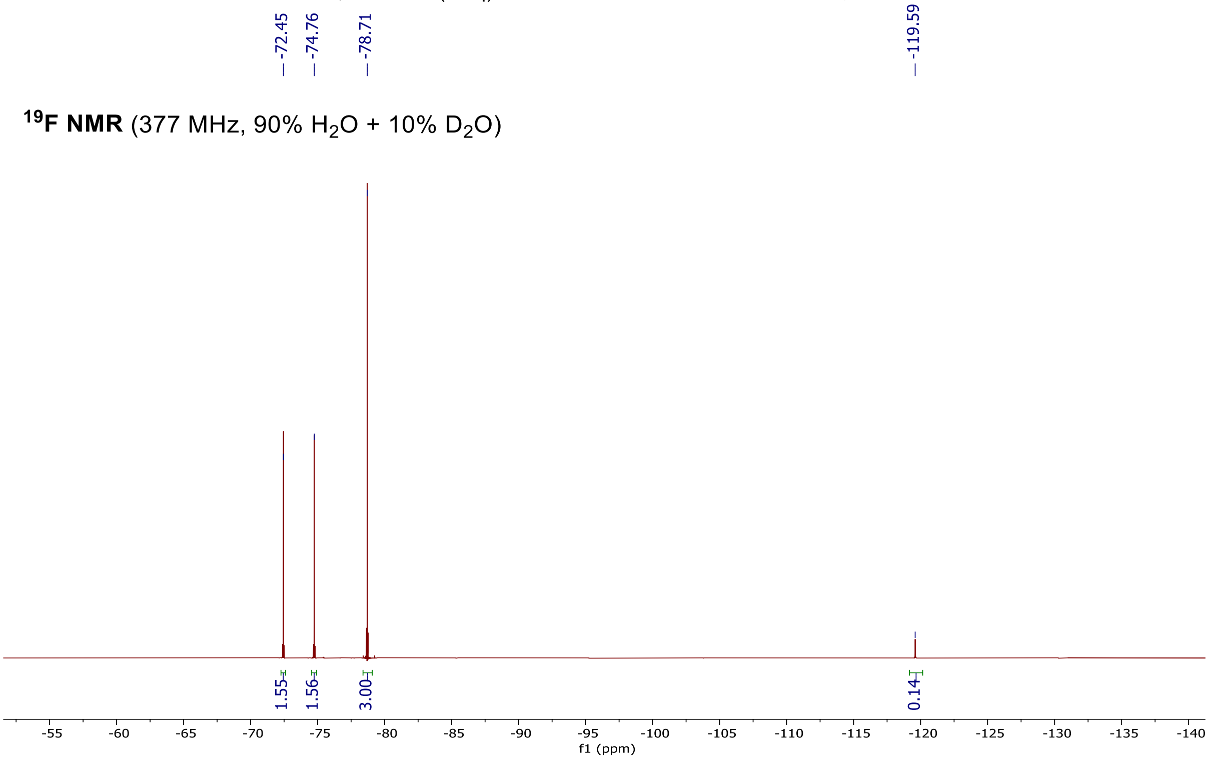

^{19}F NMR (377 MHz, 90% H_2O + 10% D_2O)

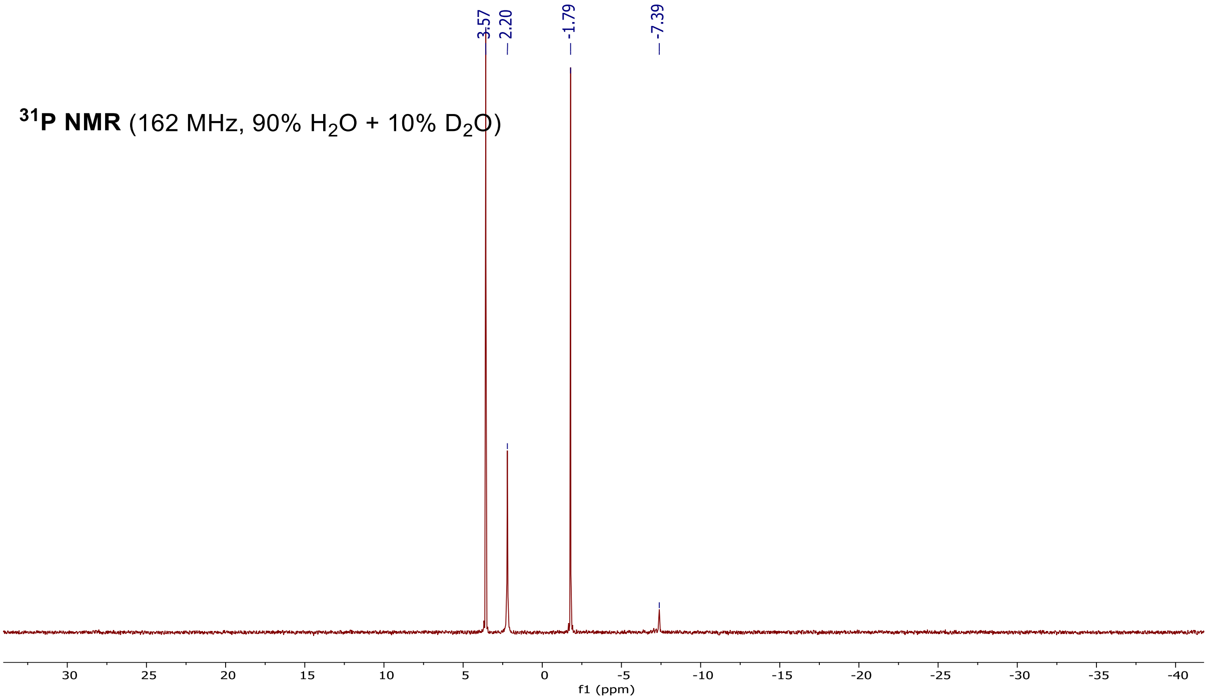

^{31}P NMR (162 MHz, 90% H_2O + 10% D_2O)

n(OTf⁻)	m (sample)	Yield of F⁻ %	Yield of PO_3F^{2-} %
0.061 mmol	51.9 mg	80%	4%

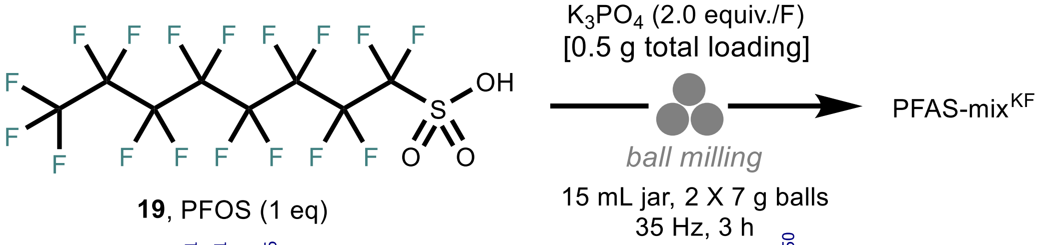

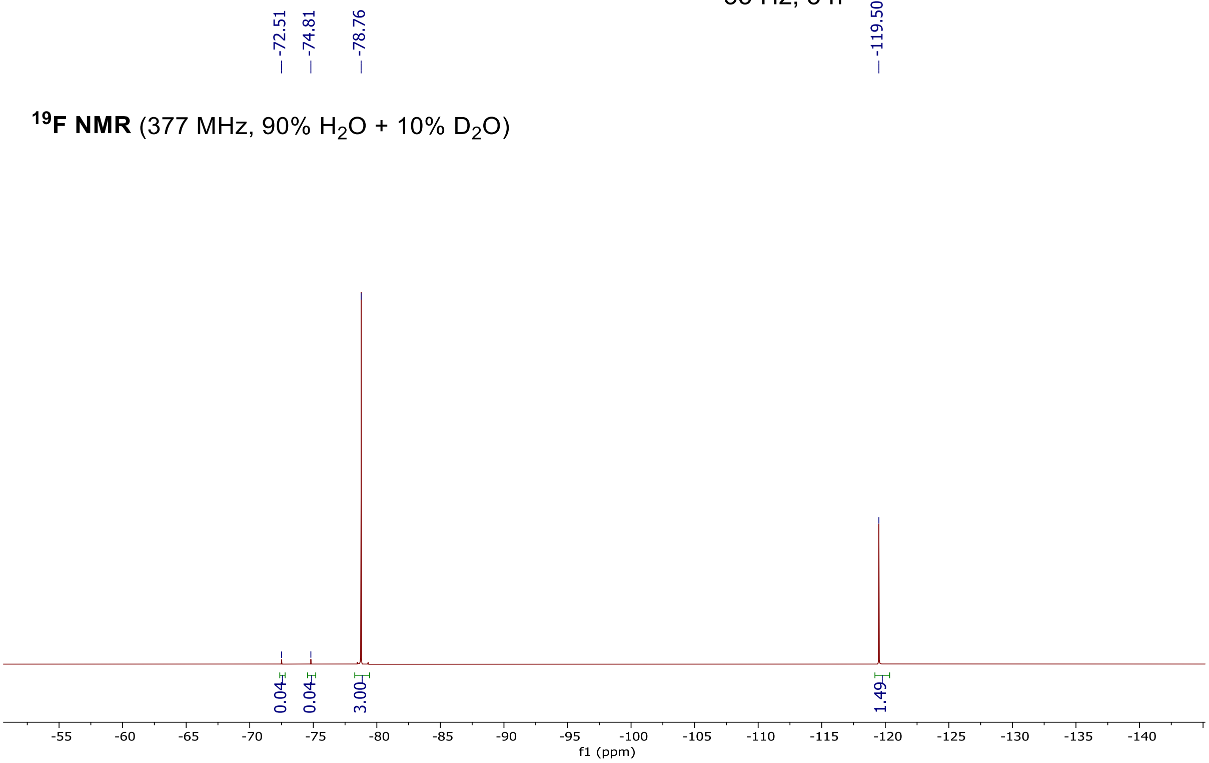

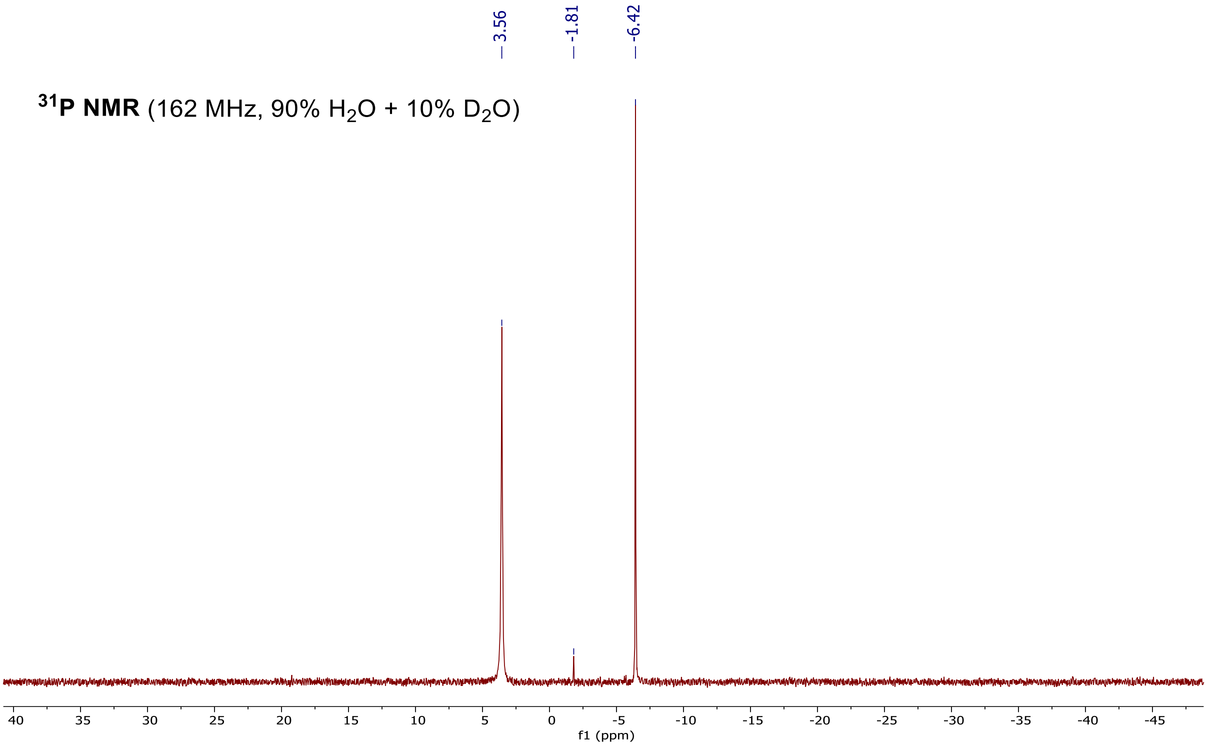

n(OTf⁻)	m (sample)	Yield of F⁻ %	Yield of PO_3F^{2-} %
0.058 mmol	54.5 mg	11%	66%

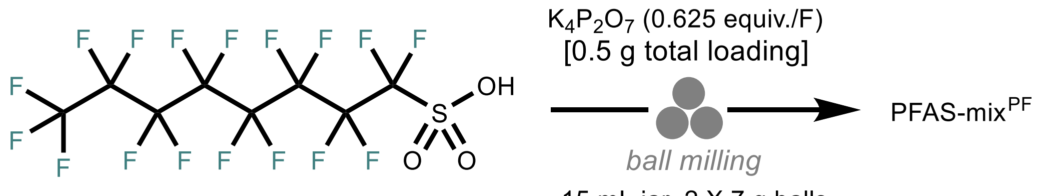

^{19}F NMR (377 MHz, 90% H_2O + 10% D_2O)

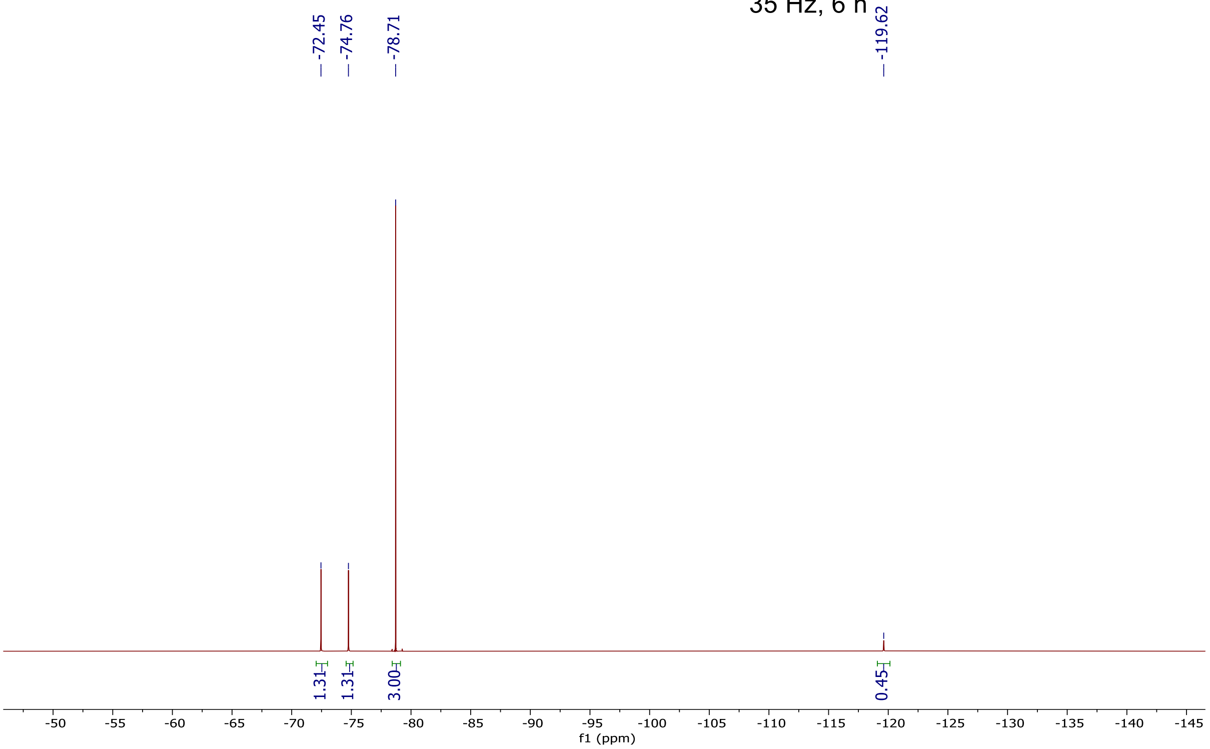

^{31}P NMR (162 MHz, 90% H_2O + 10% D_2O)

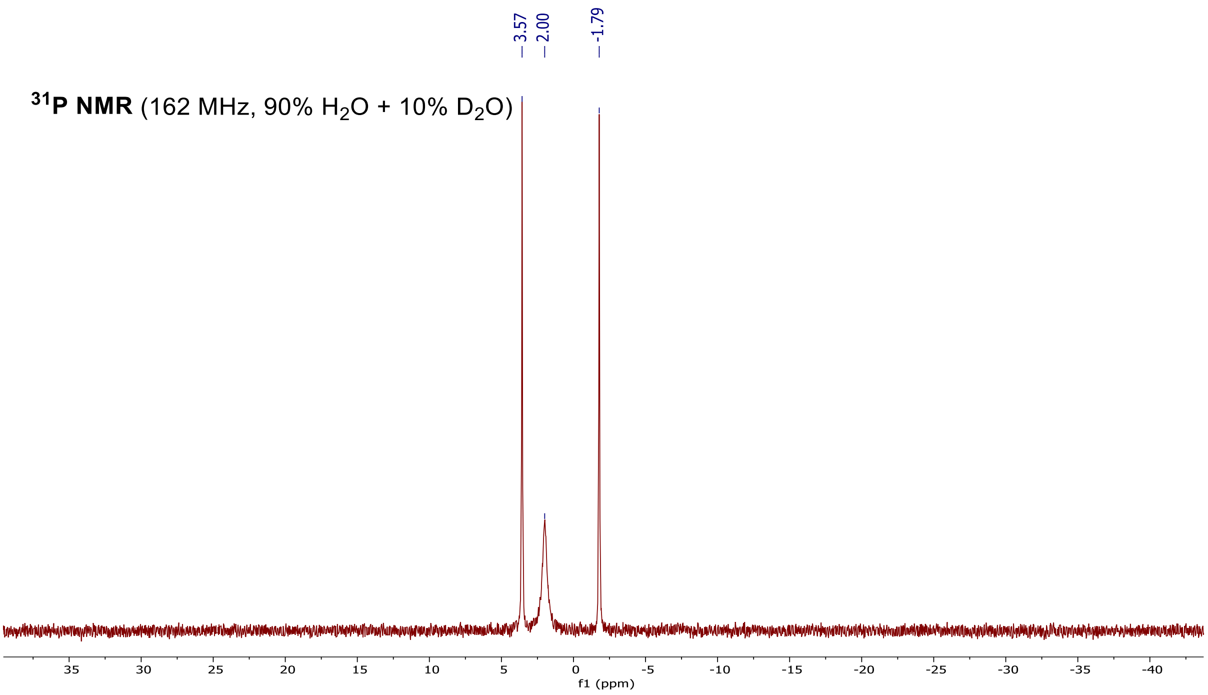

n(OTf^-)	m (sample)	Yield of F^- %	Yield of PO_3F^{2-} %
0.051 mmol	54.4 mg	77%	21%

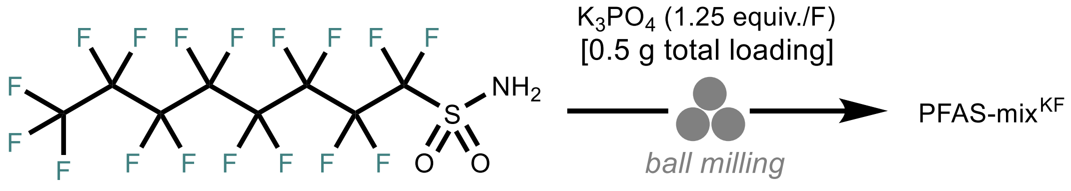

20, PFOSA (1 eq)

^{19}F NMR (377 MHz, 90% H_2O + 10% D_2O)

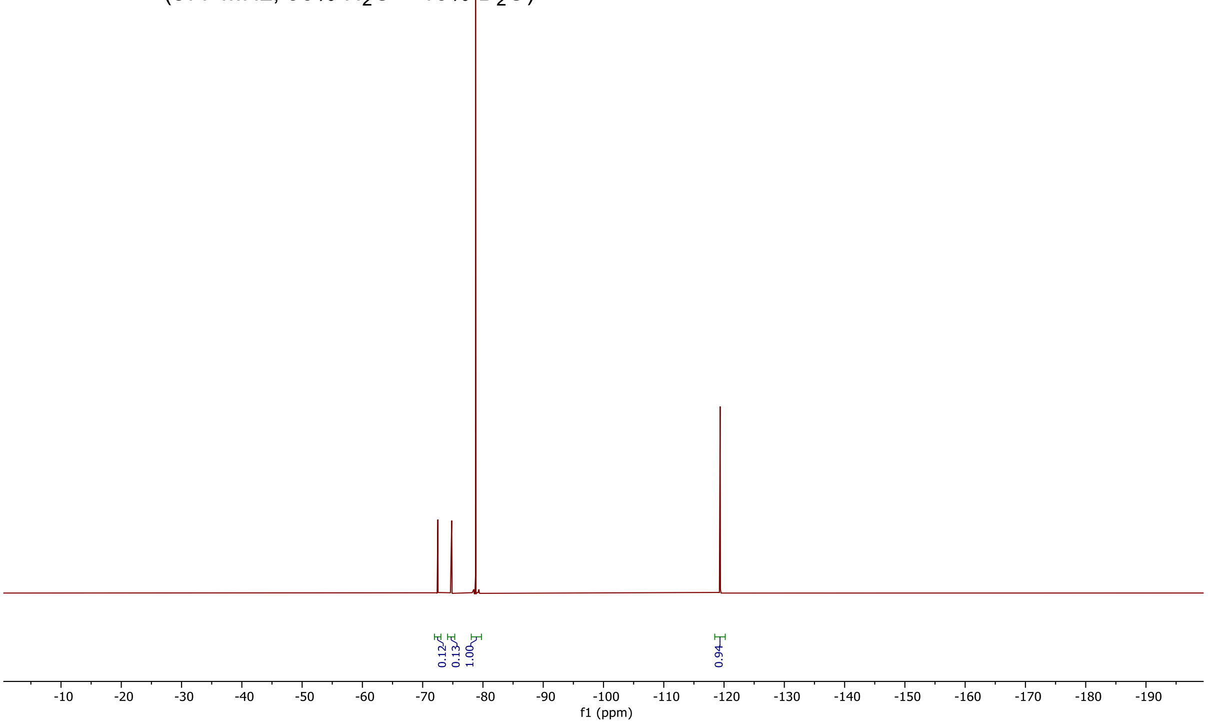

^{31}P NMR (162 MHz, 90% H_2O + 10% D_2O)

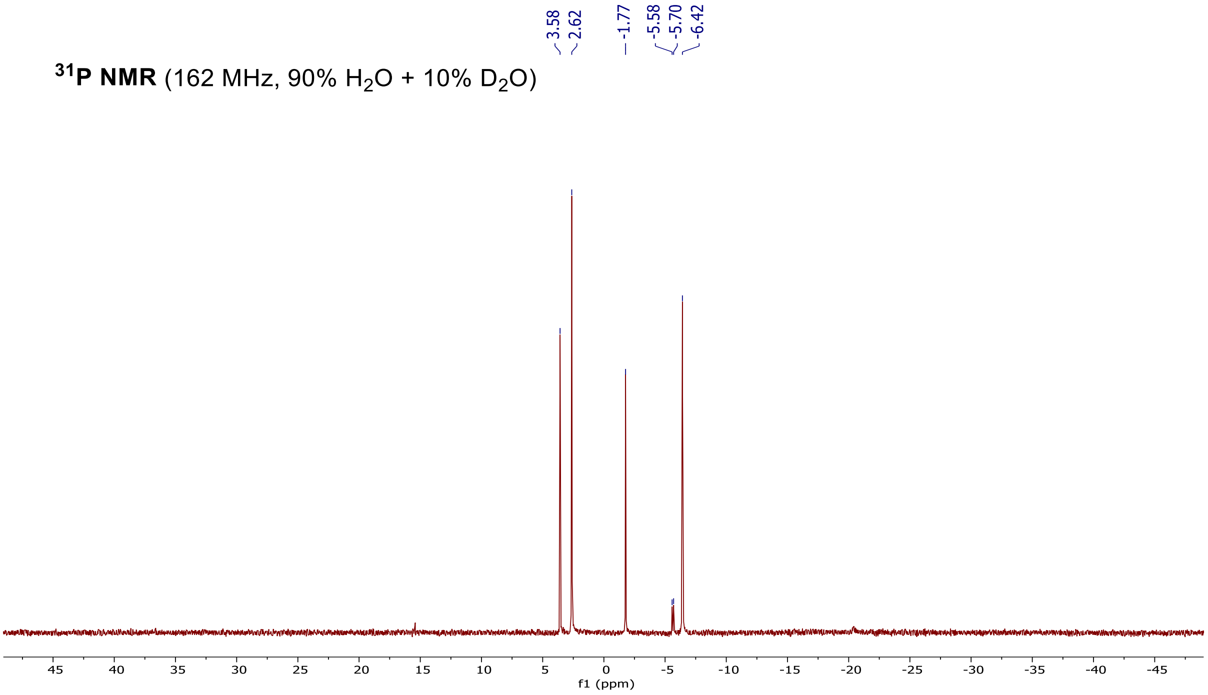

n(OTf^-)	m (sample)	Yield of F^- %	Yield of PO_3F^{2-} %
0.058 mmol	51.6 mg	6%	73%

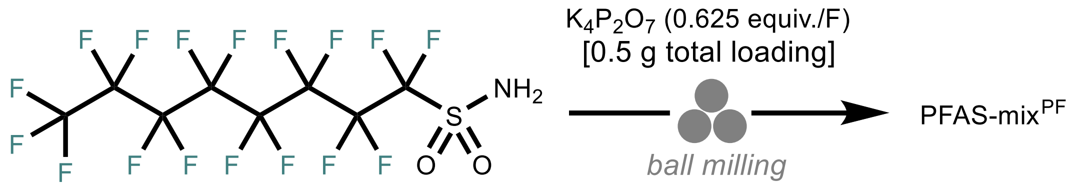

20, PFOSA (1 eq)

15 mL jar, 2 X 7 g balls
35 Hz, 3 h

^{19}F NMR (377 MHz, 90% H_2O + 10% D_2O)

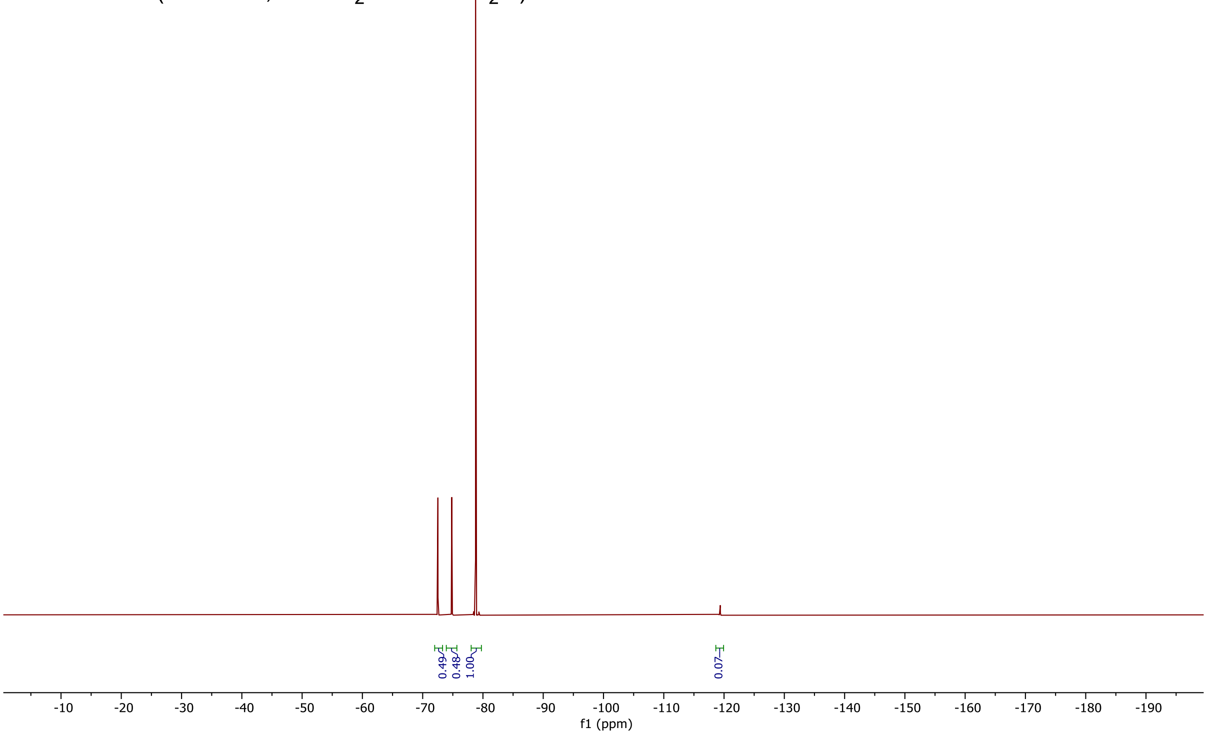

^{31}P NMR (162 MHz, 90% H_2O + 10% D_2O)

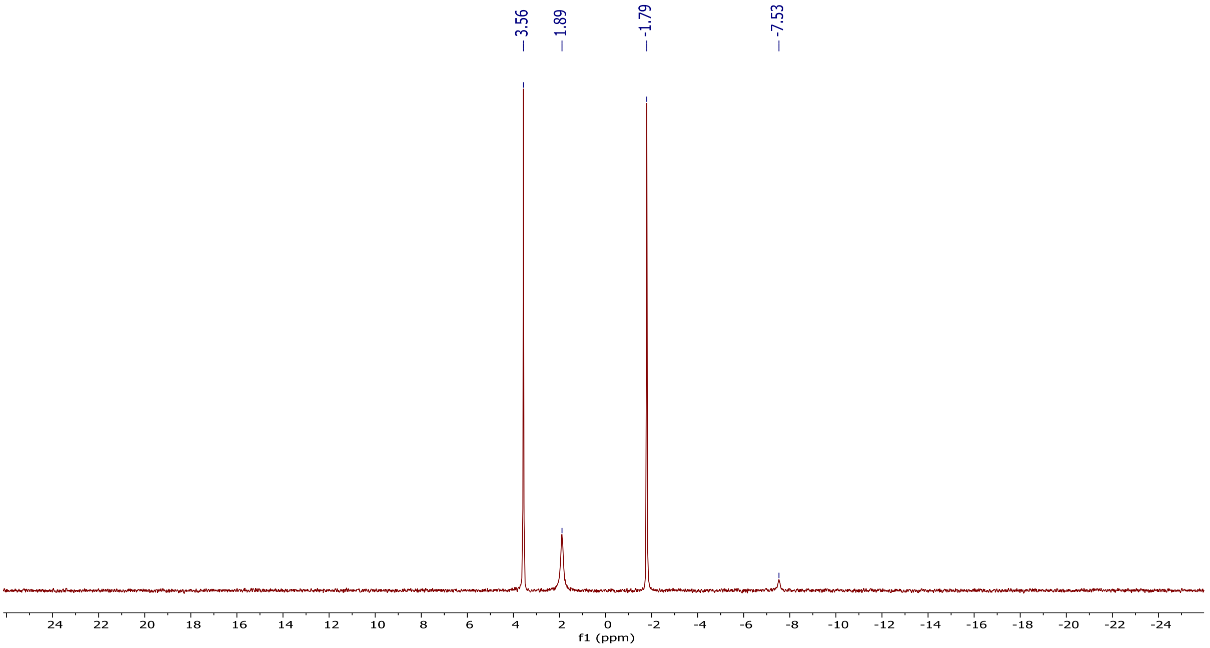

n(OTf⁻)	m (sample)	Yield of F⁻ %	Yield of PO_3F^{2-} %
0.066 mmol	54.0 mg	73%	3%

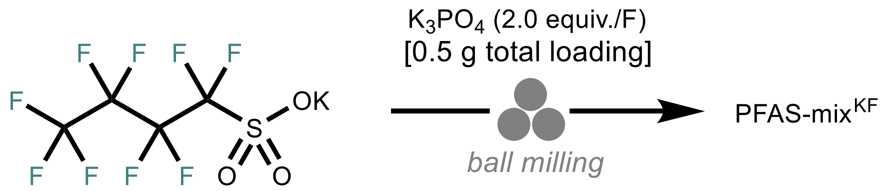

21, KPFBS (1 eq)

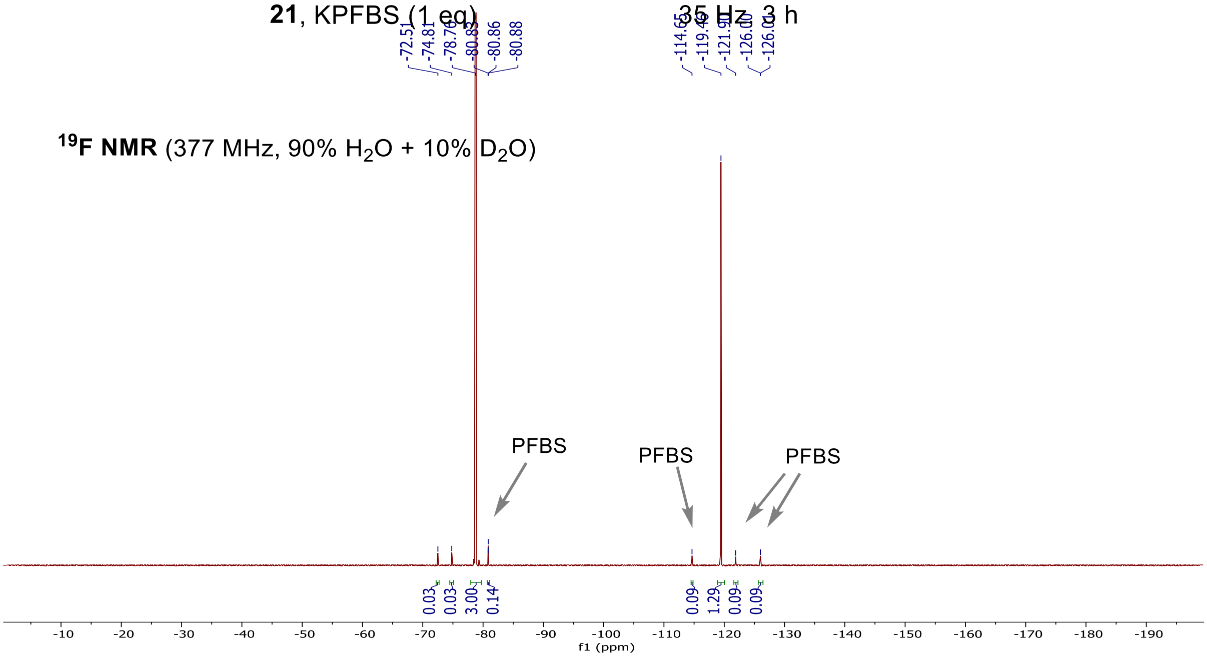

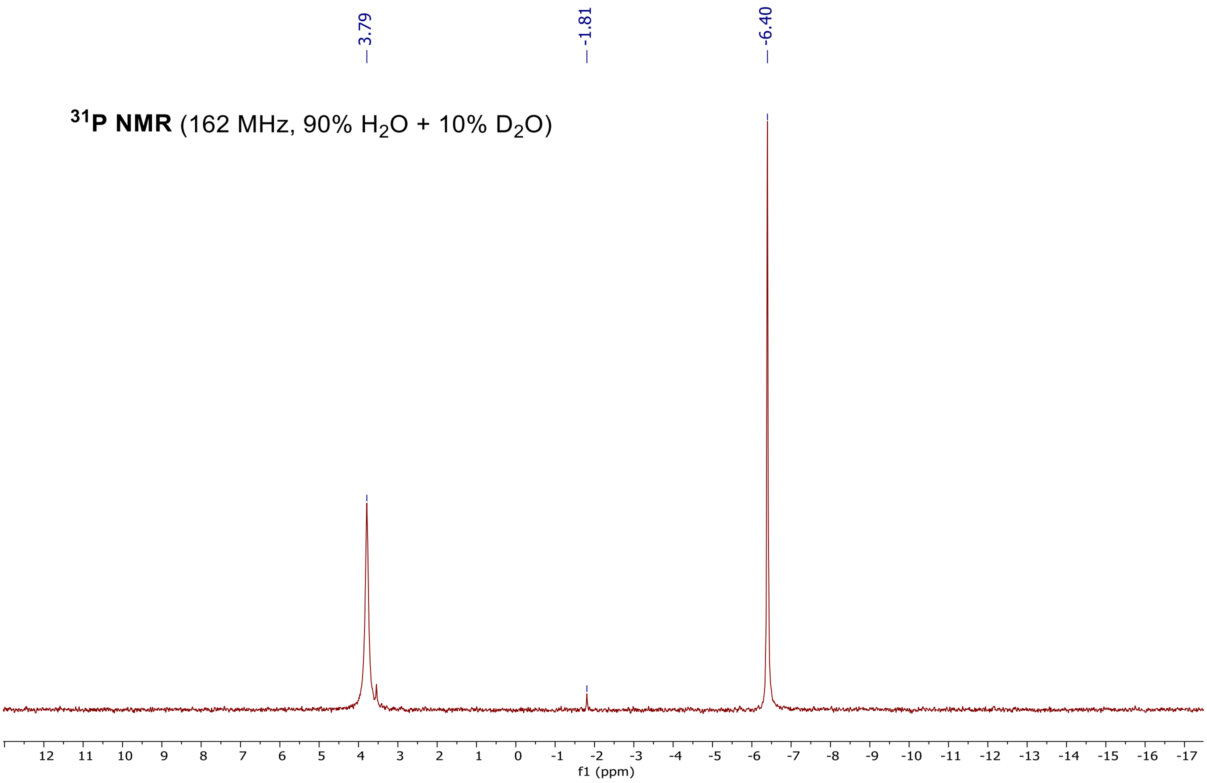

n(OTf⁻)	m (sample)	Yield of F⁻ %	Yield of PO_3F^{2-} %
0.065 mmol	61.5 mg	4%	82%

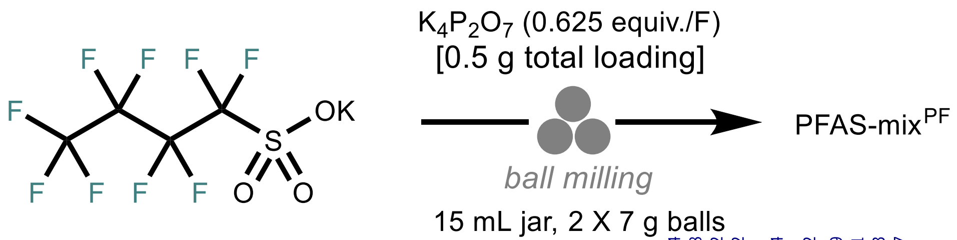

21, KPFBS (1 eq)

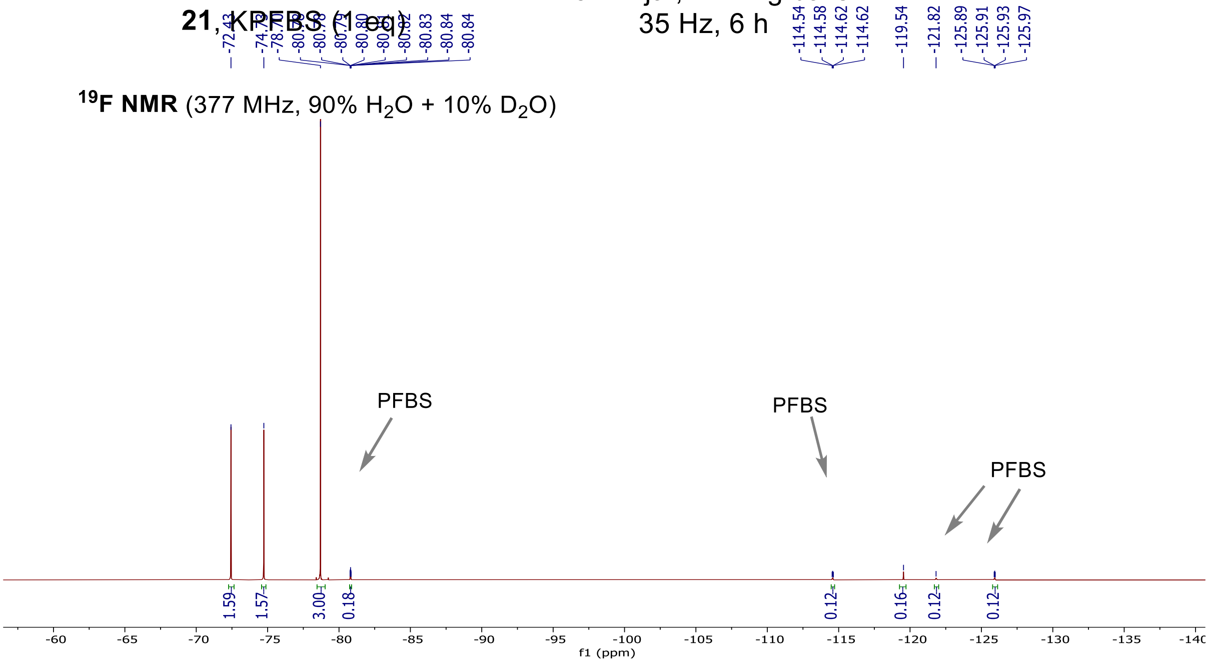

^{19}**F NMR** (377 MHz, 90% H_2O + 10% D_2O)

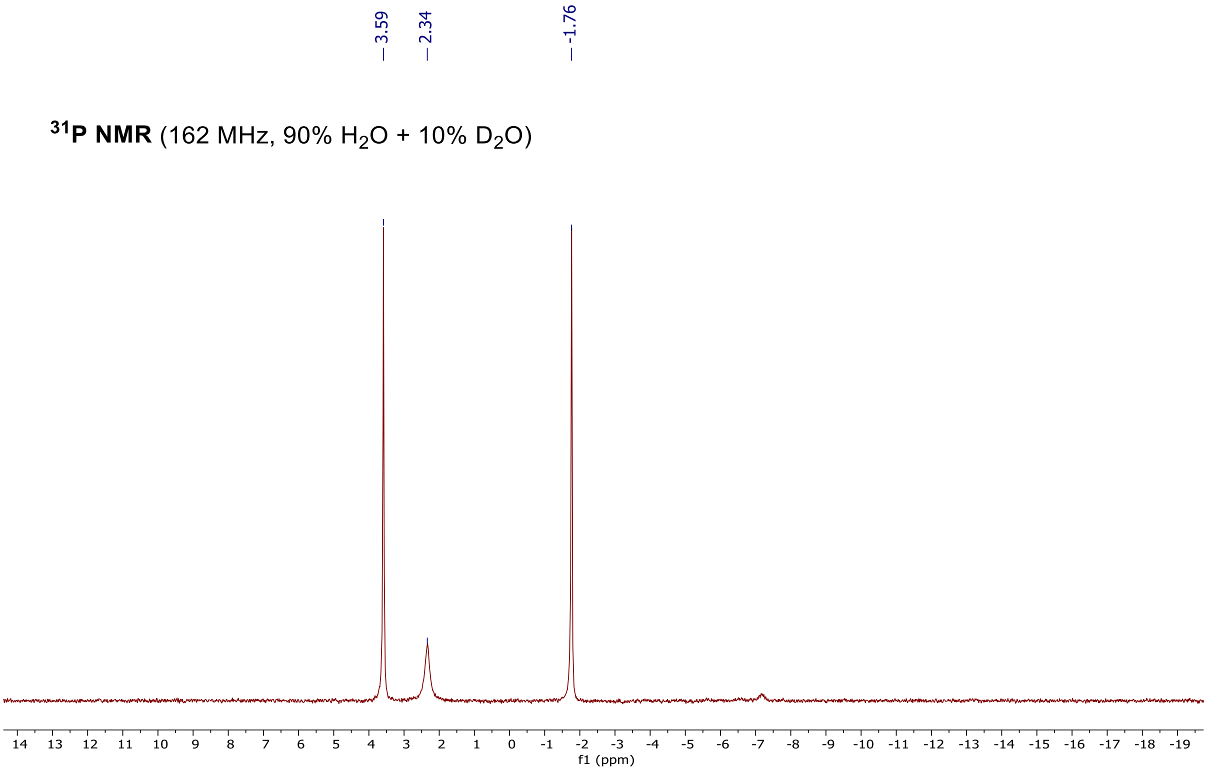

^{31}**P NMR** (162 MHz, 90% H_2O + 10% D_2O)

n(OTf⁻)	m (sample)	Yield of F⁻ %	Yield of PO_3F^{2-} %
0.081 mmol	53.4 mg	77%	7%

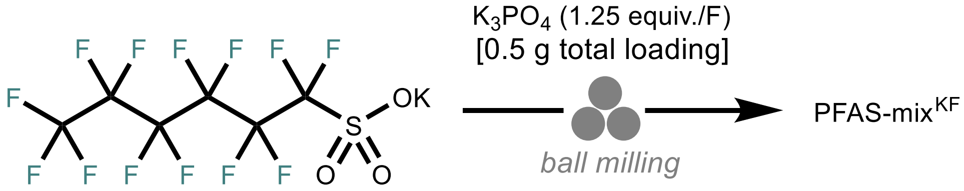

^{19}F NMR (377 MHz, 90% H_2O + 10% D_2O)
22, KPFHxS (1 eq)

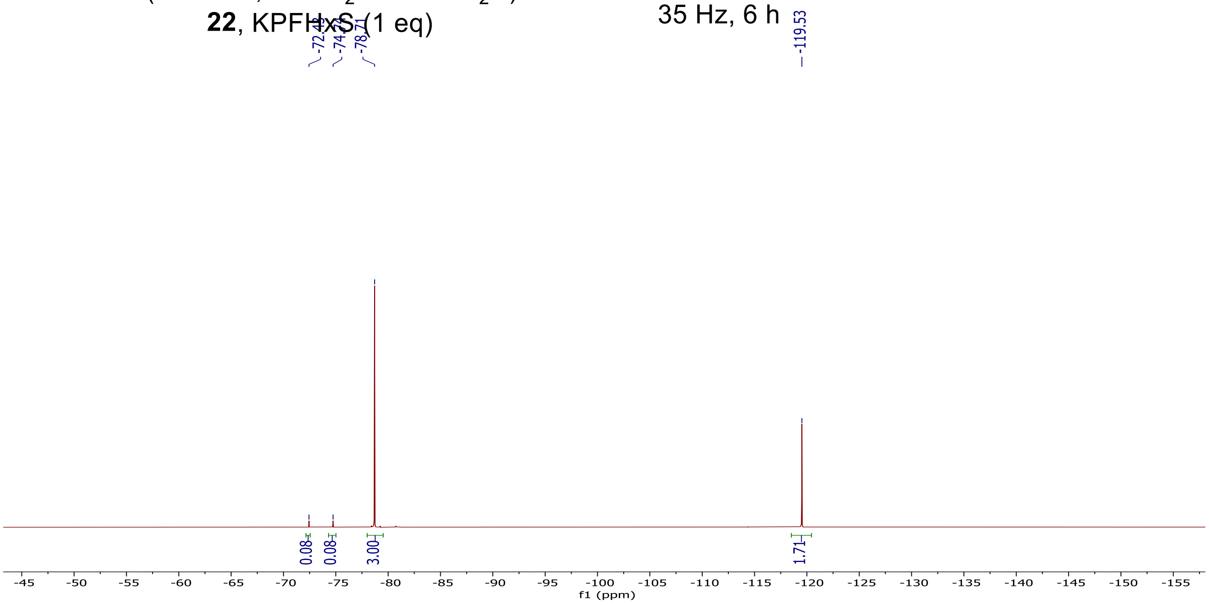

^{31}P NMR (162 MHz, 90% H_2O + 10% D_2O)

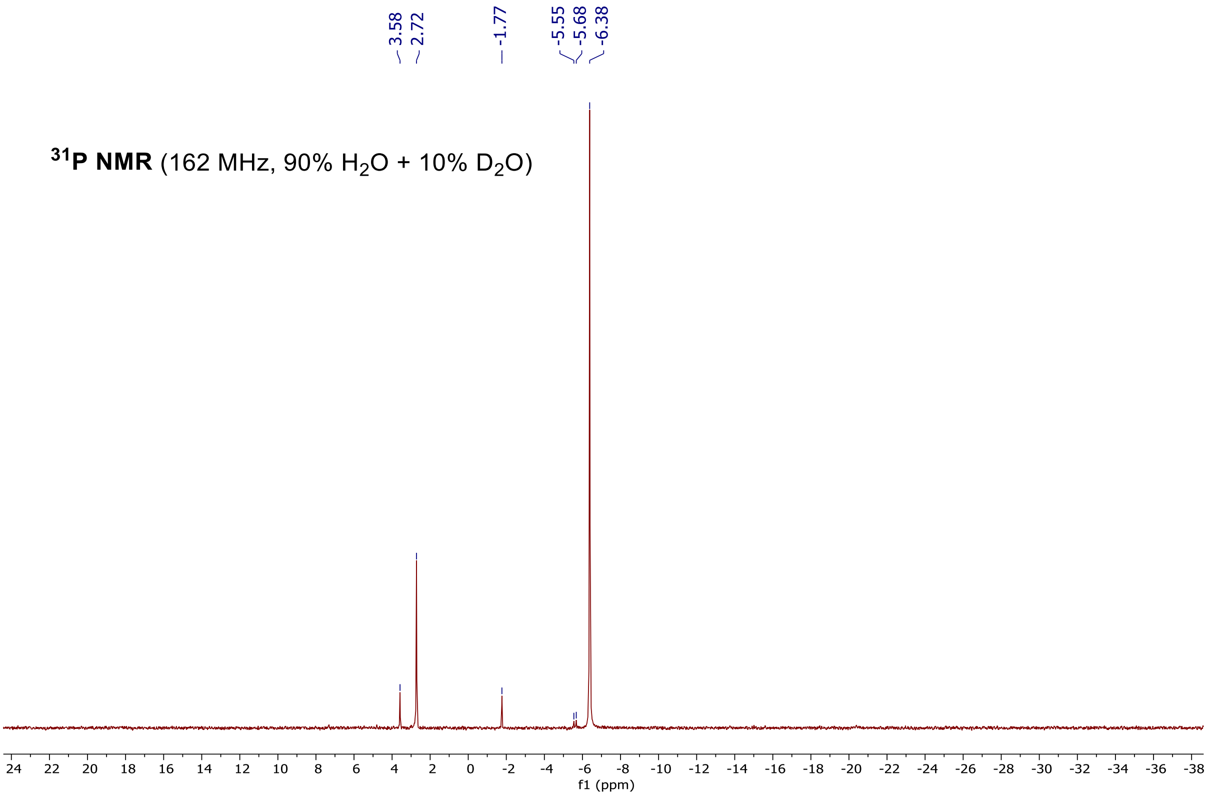

n(OTf⁻)	m (sample)	Yield of F^- %	Yield of PO_3F^{2-} %
0.068 mmol	52.4 mg	6%	89%

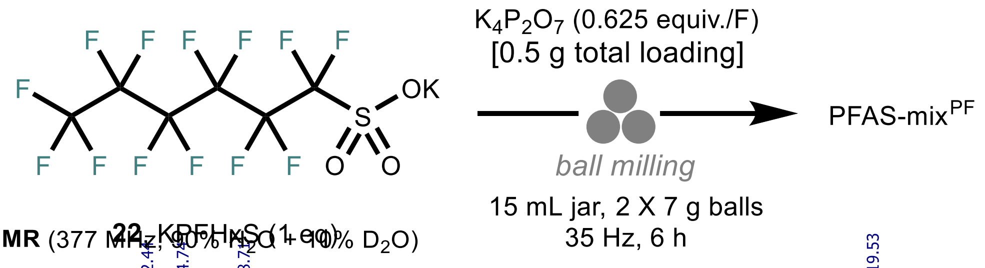

22, KPFHxS (1 eq)

^{19}F NMR (377 MHz, 90% H_2O + 10% D_2O)

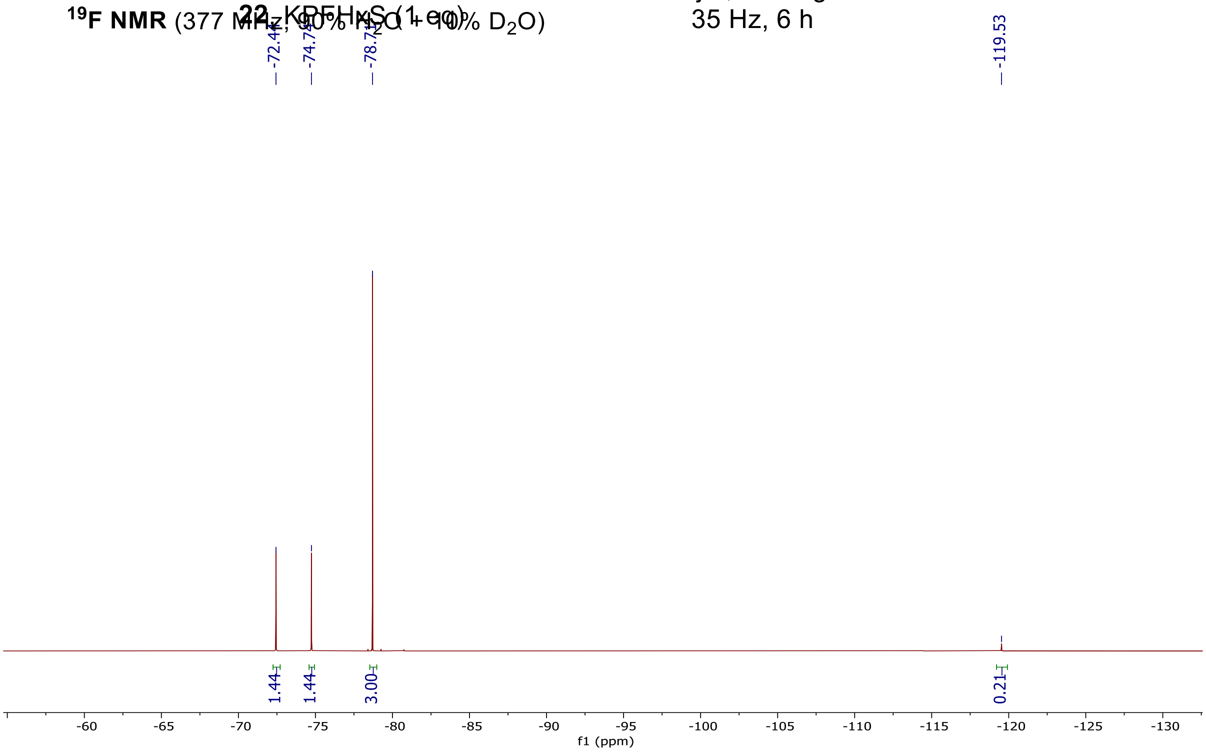

^{31}P NMR (162 MHz, 90% H_2O + 10% D_2O)

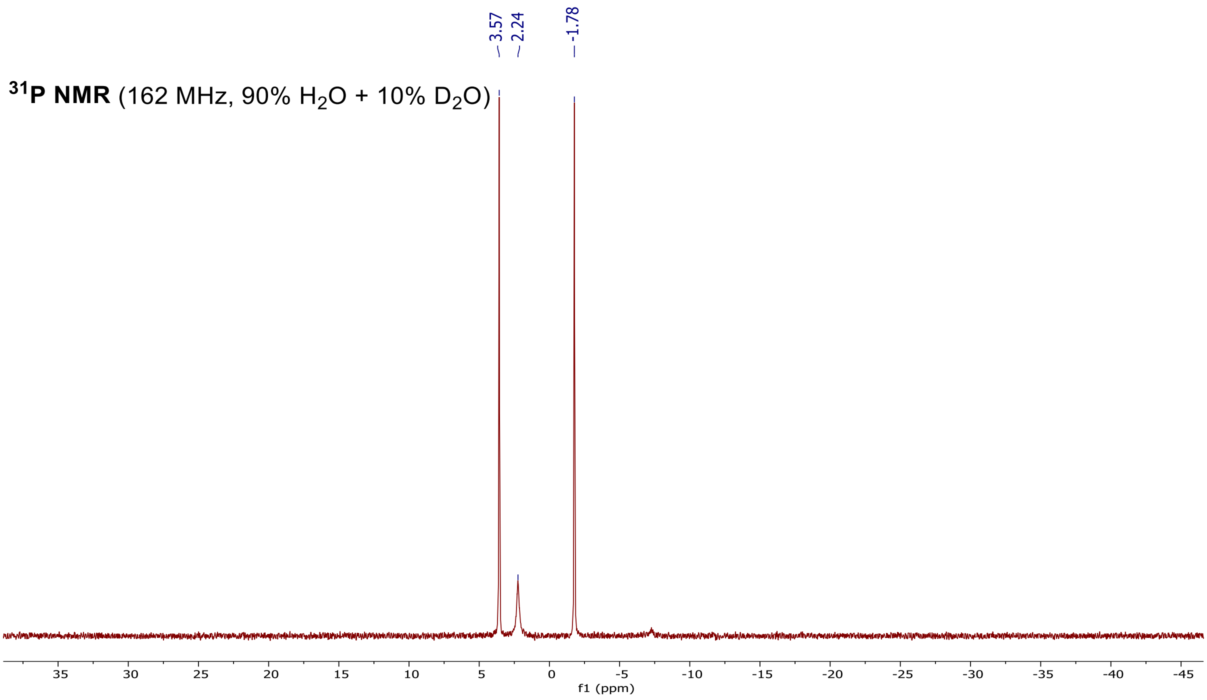

n(OTf⁻)	m (sample)	Yield of F⁻ %	Yield of PO_3F^{2-} %
0.058 mmol	73 mg	59%	41%

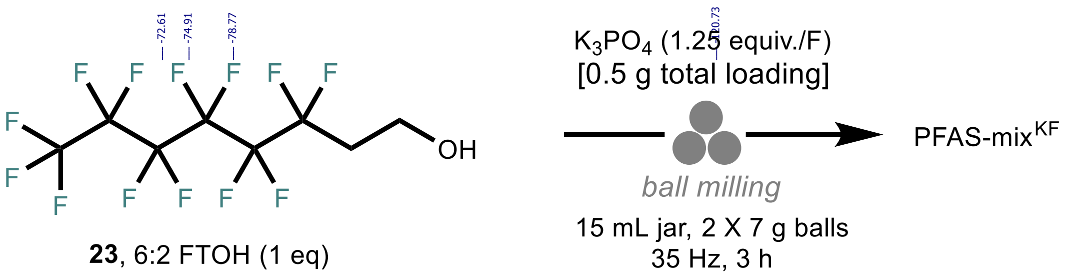

^{19}F NMR (377 MHz, 90% H_2O + 10% D_2O)

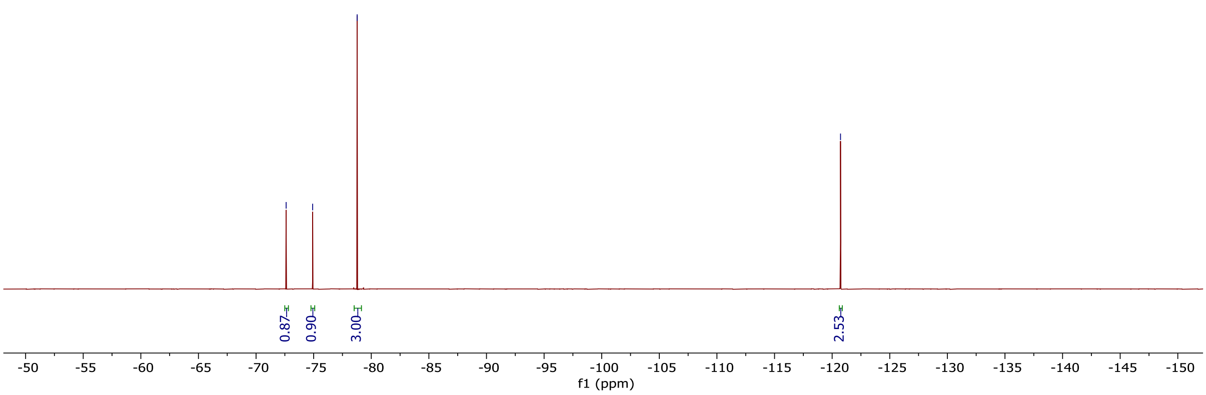

^{31}P NMR (162 MHz, 90% H_2O + 10% D_2O)

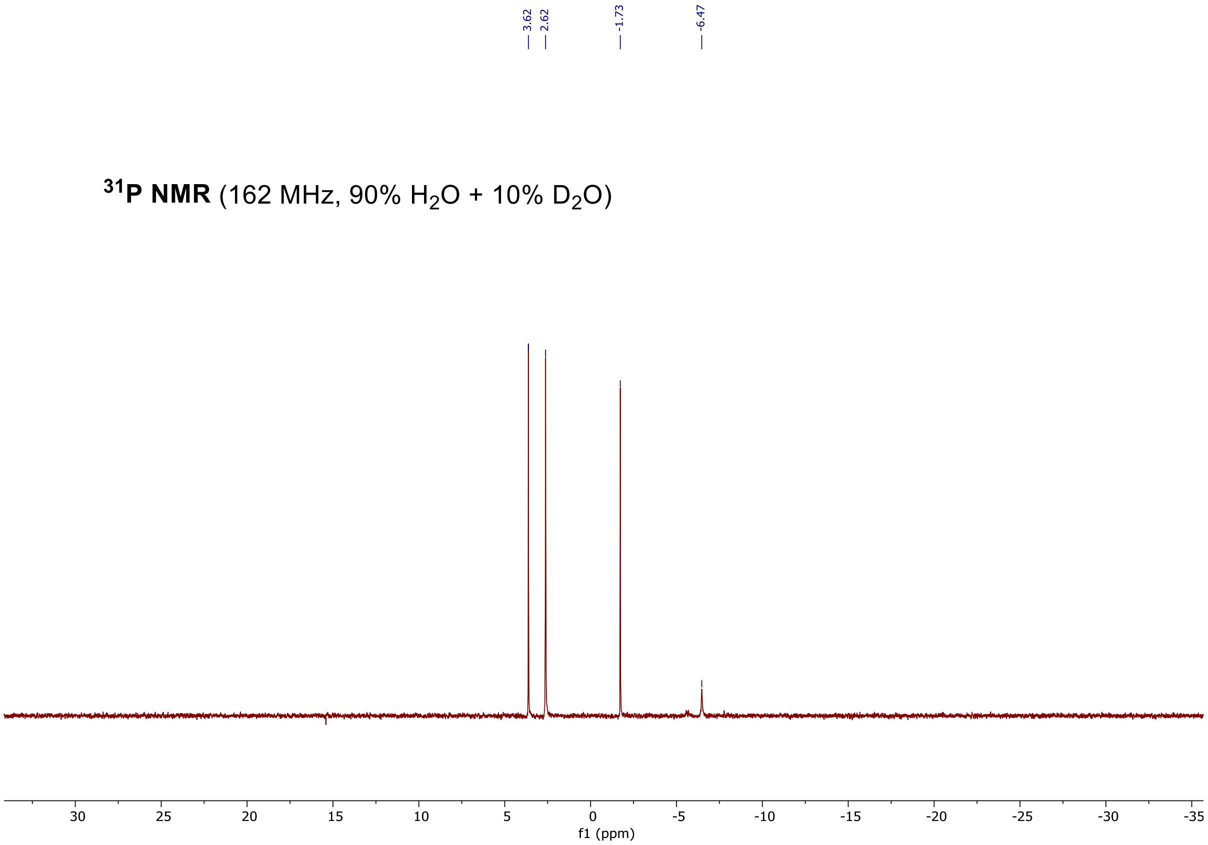

n(OTf⁻)	m (sample)	Yield of F⁻ %	Yield of PO_3F^{2-} %
0.058 mmol	76 mg	19%	81%

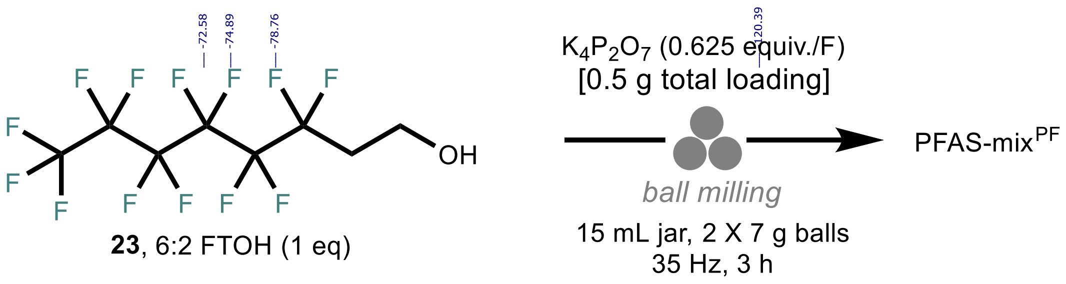

^{19}F NMR (377 MHz, 90% H_2O + 10% D_2O)

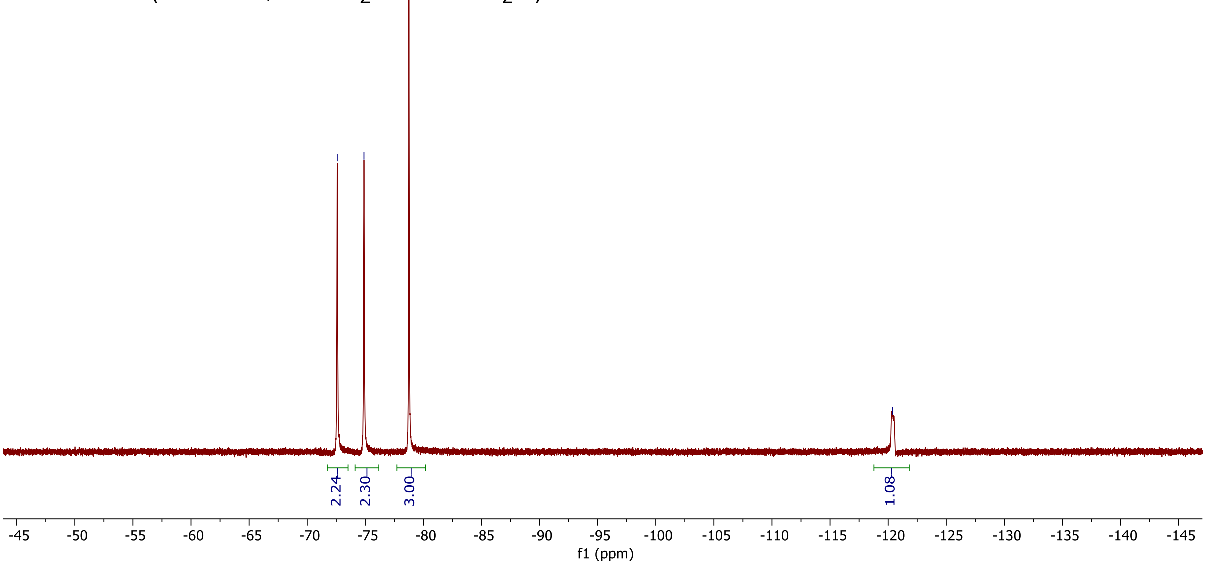

^{31}P NMR (162 MHz, 90% H_2O + 10% D_2O)

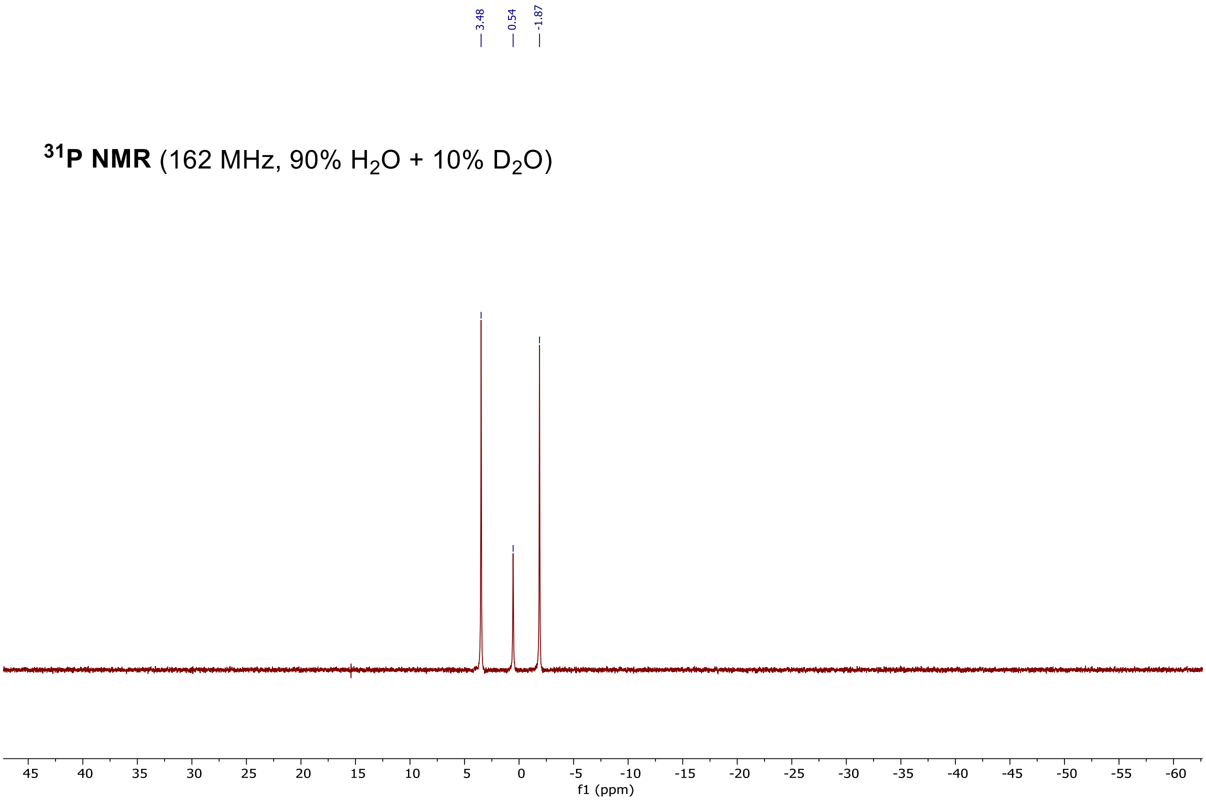

n(OTf⁻)	m (sample)	Yield of F⁻ %	Yield of PO_3F^{2-} %
0.050 mmol	48.1 mg	78%	15%

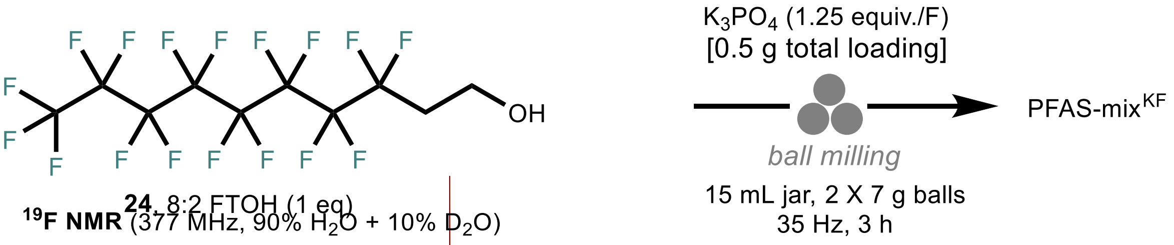

24, 8:2 FTOH (1 eq)

^{19}F NMR (377 MHz, 90% H_2O + 10% D_2O)

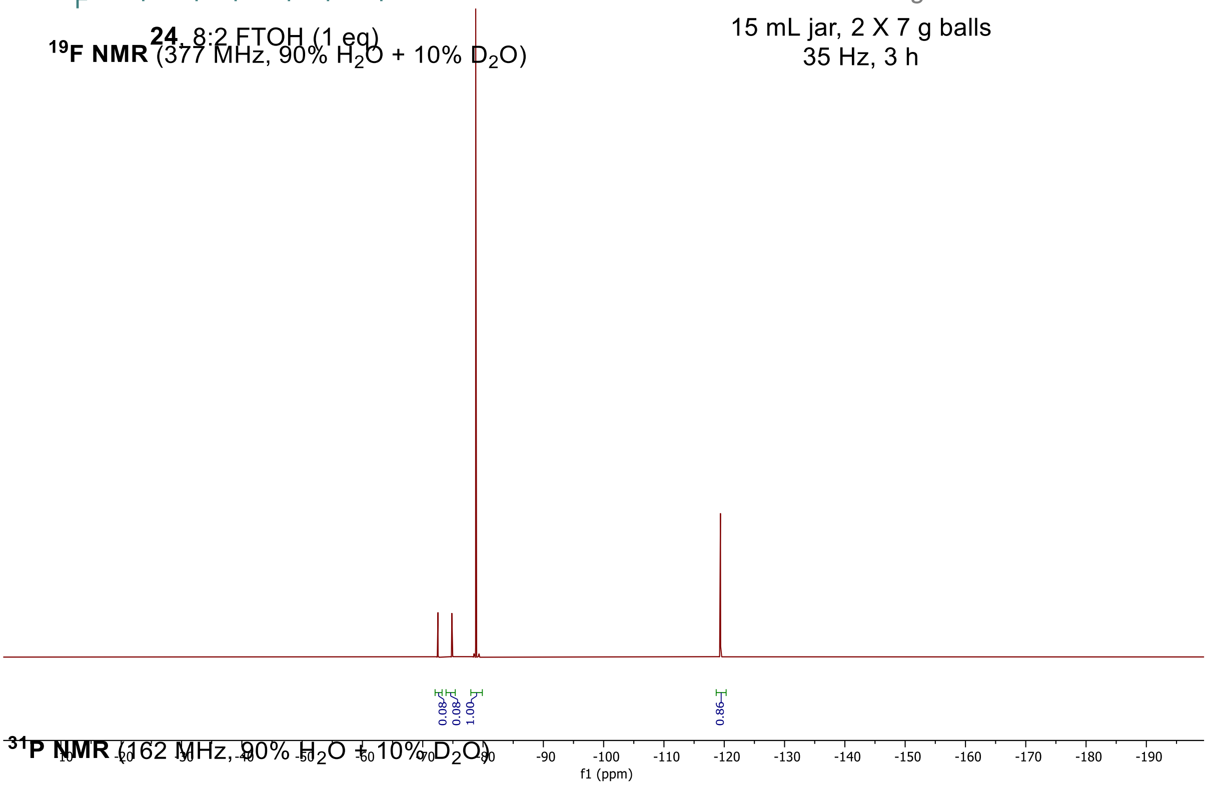

^{31}P NMR (162 MHz, 90% H_2O + 10% D_2O)

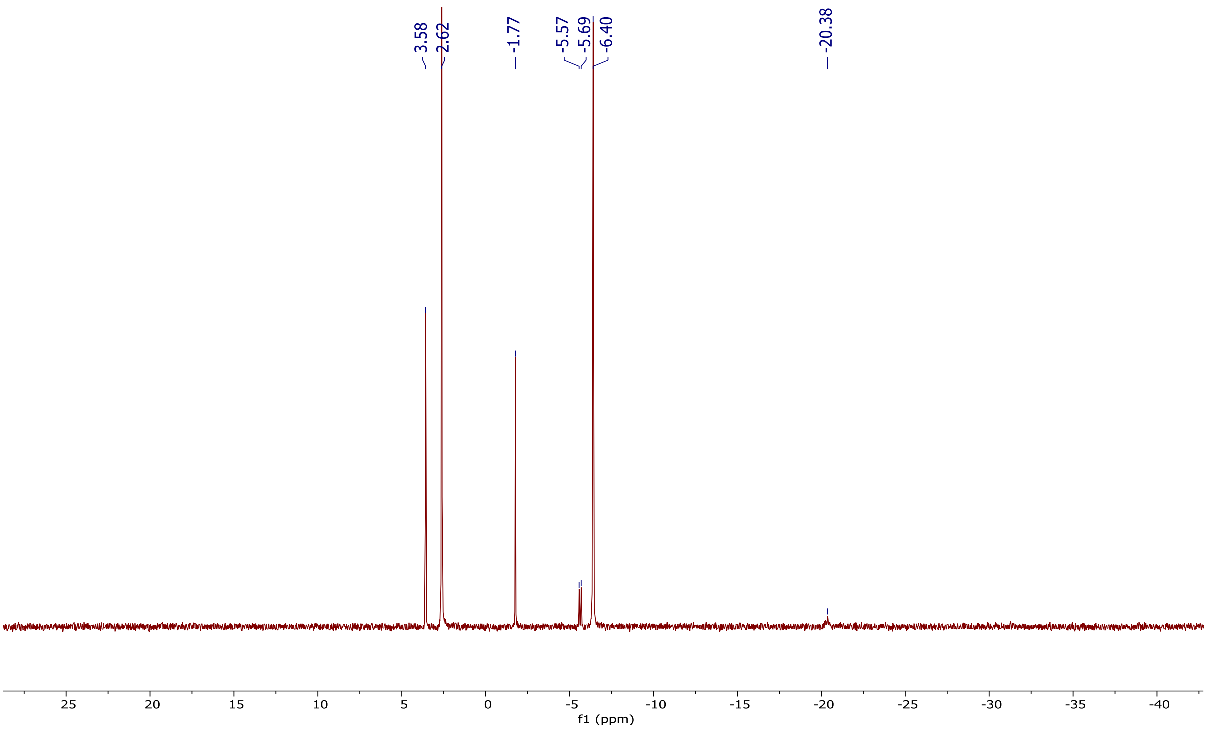

n(OTf⁻)	m (sample)	Yield of F⁻ %	Yield of PO_3F^{2-} %
0.053 mmol	46.8 mg	17%	71%

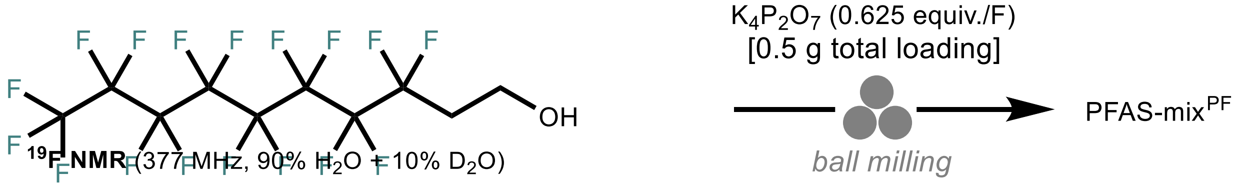

24, 8:2 FTOH (1 eq)

^{19}F NMR (377 MHz, 90% H_2O + 10% D_2O)

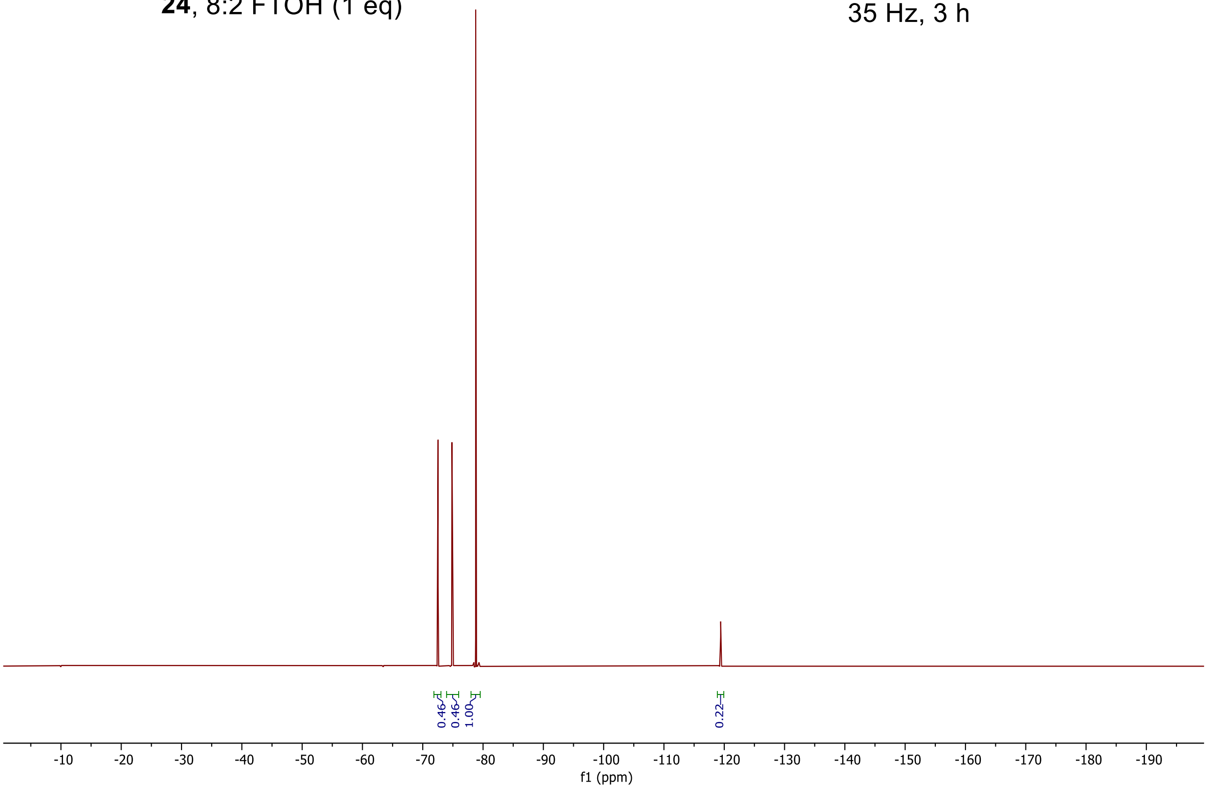

^{31}P NMR (162 MHz, 90% H_2O + 10% D_2O)

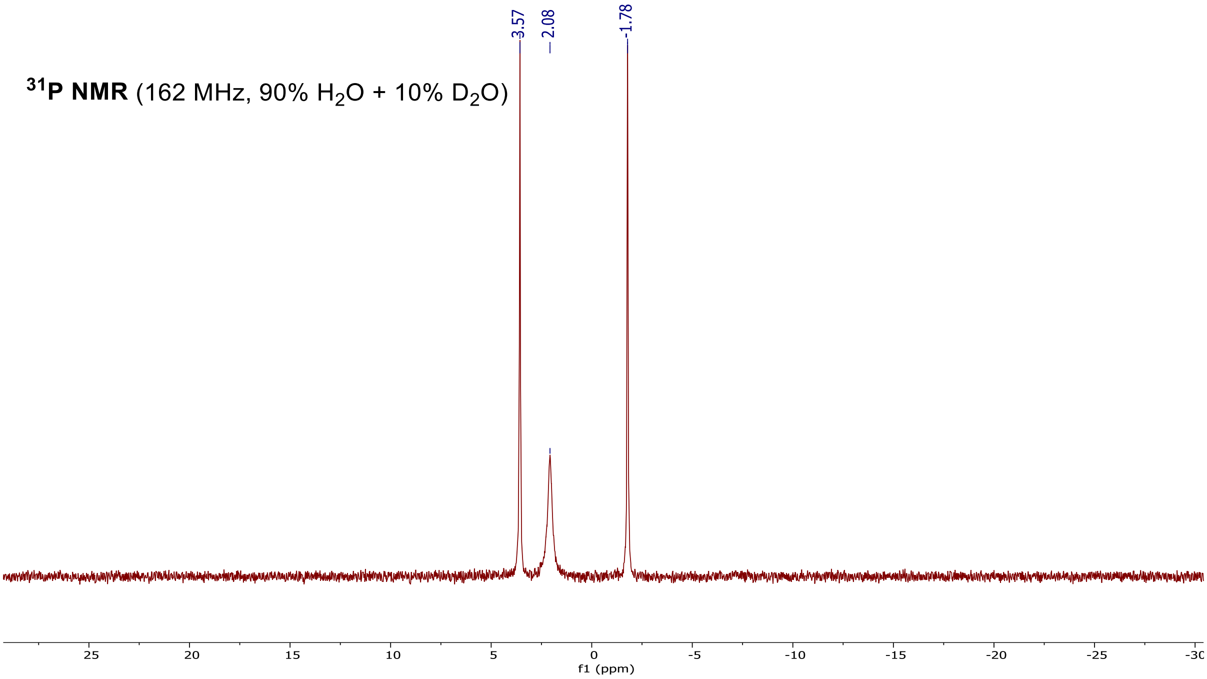

n(OTf⁻)	m (sample)	Yield of F^- %	Yield of PO_3F^{2-} %
0.058 mmol	41.0 mg	65%	29%

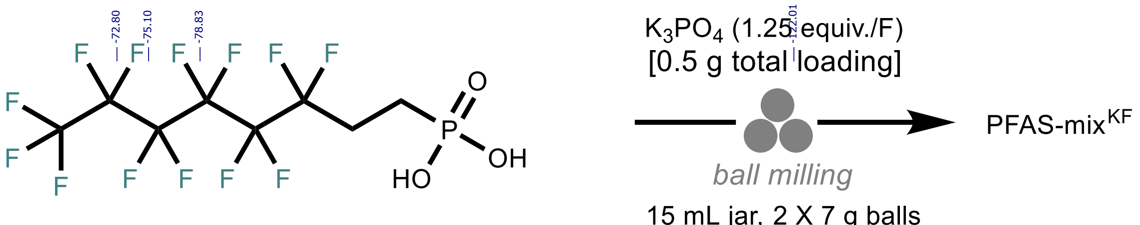

25, 6:2 FTPA (1 eq)

^{19}F NMR (377 MHz, 90% H_2O + 10% D_2O)

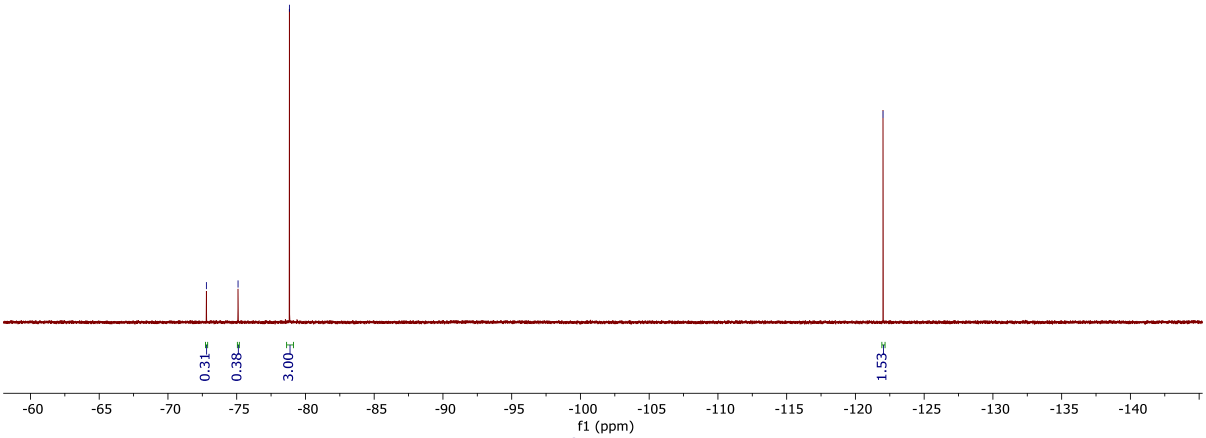

^{31}P NMR (162 MHz, 90% H_2O + 10% D_2O)

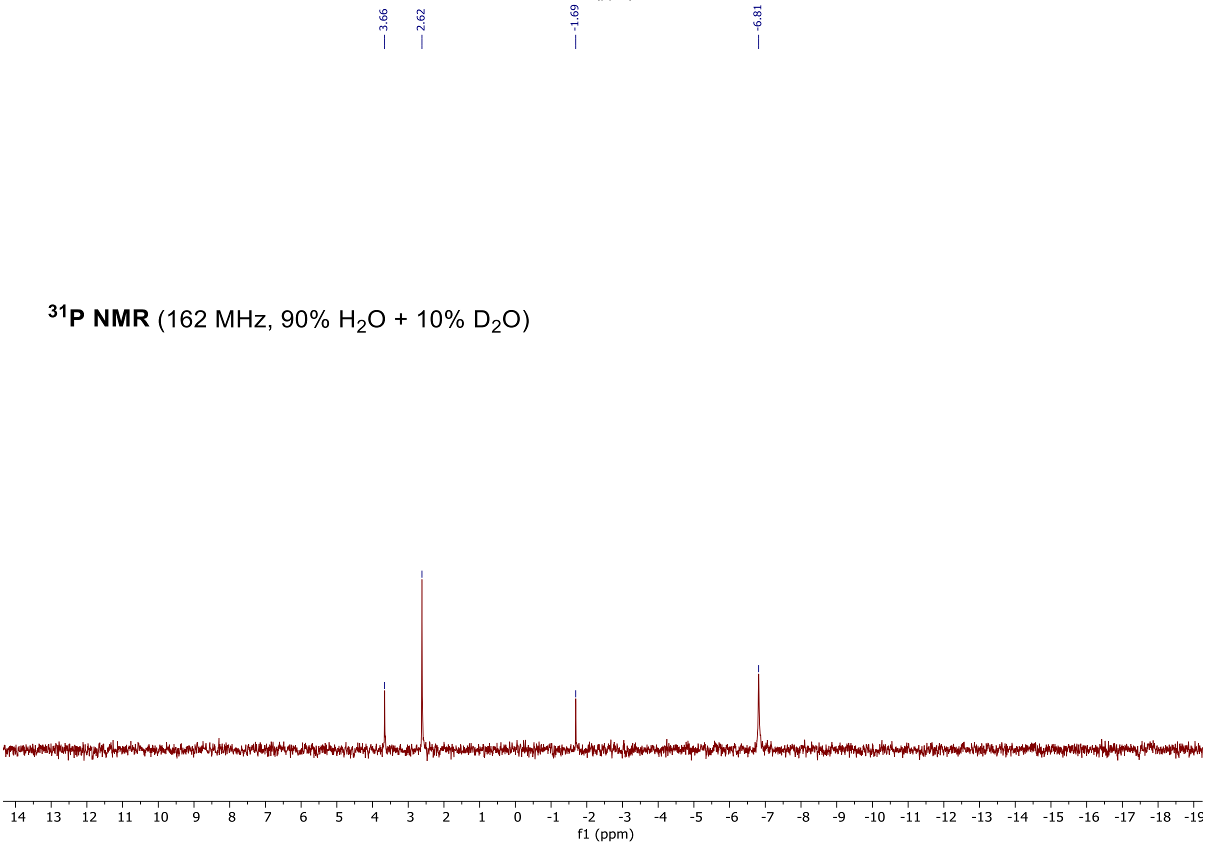

n(OTf⁻)	m (sample)	Yield of F⁻ %	Yield of PO_3F^{2-} %
0.068 mmol	46.2 mg	9%	87%

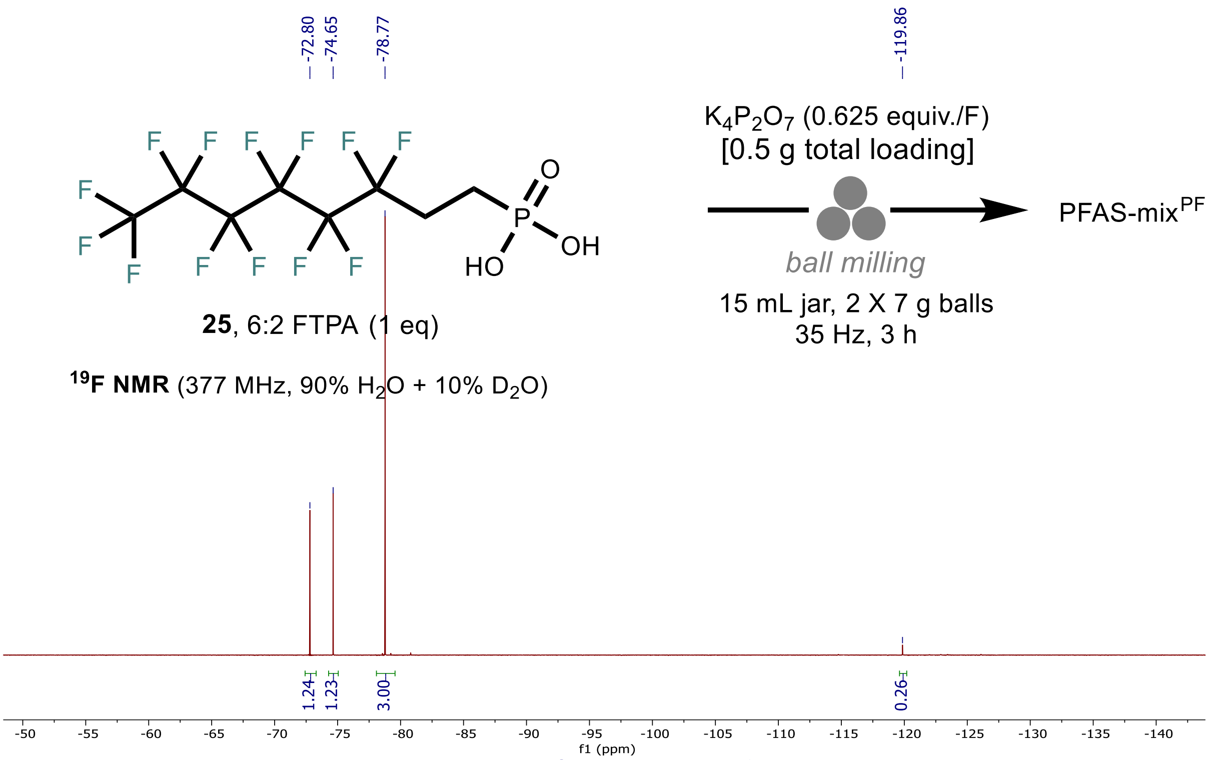

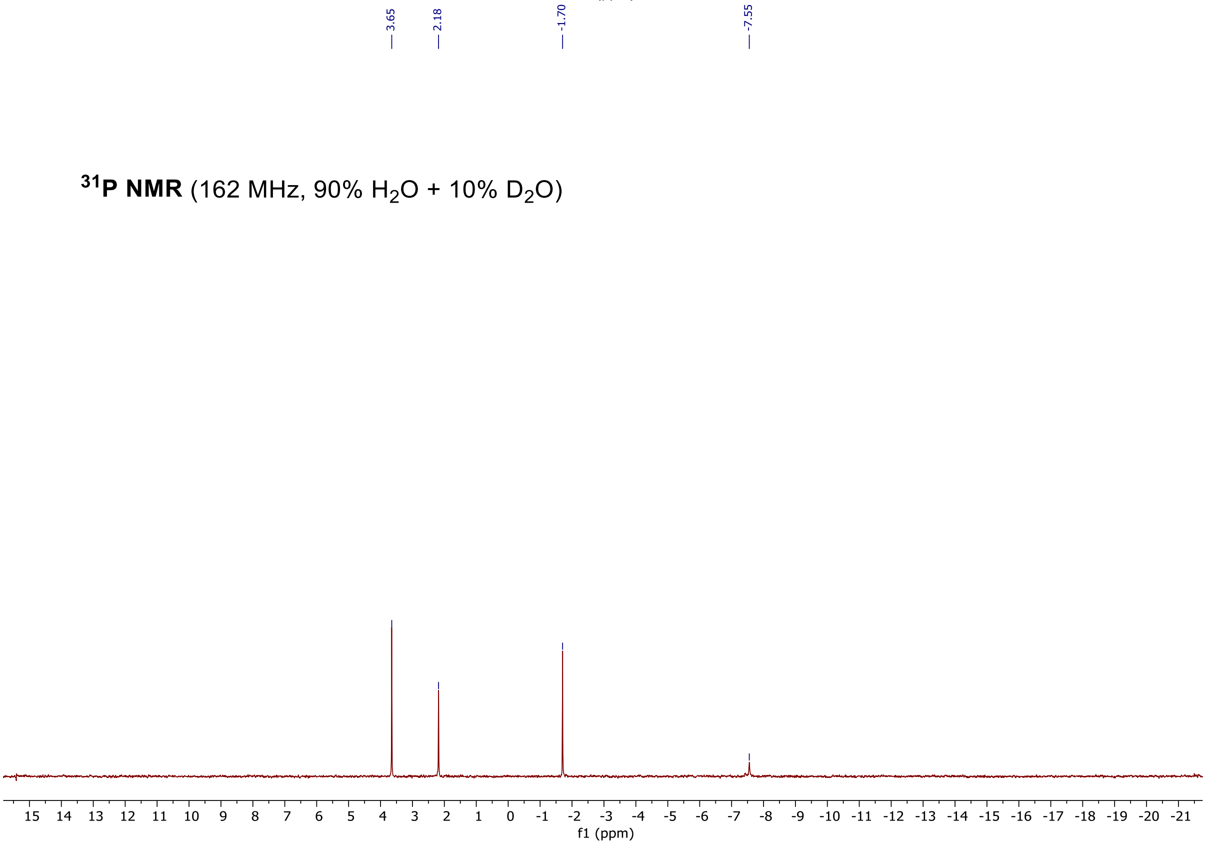

n(OTf^-)	m (sample)	Yield of F^- %	Yield of PO_3F^{2-} %
0.058 mmol	46 mg	74%	26%

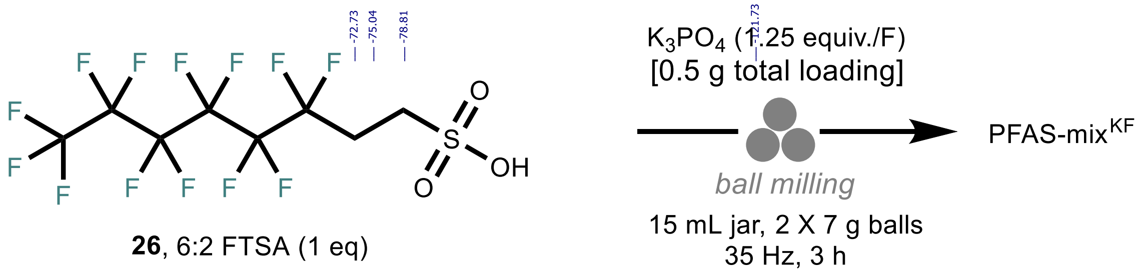

^{19}F NMR (377 MHz, 90% H_2O + 10% D_2O)

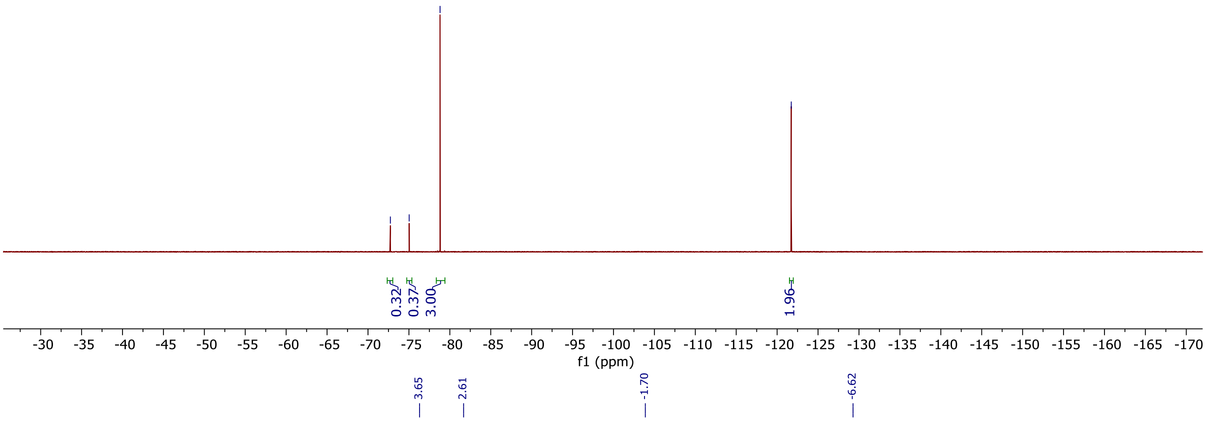

^{31}P NMR (162 MHz, 90% H_2O + 10% D_2O)

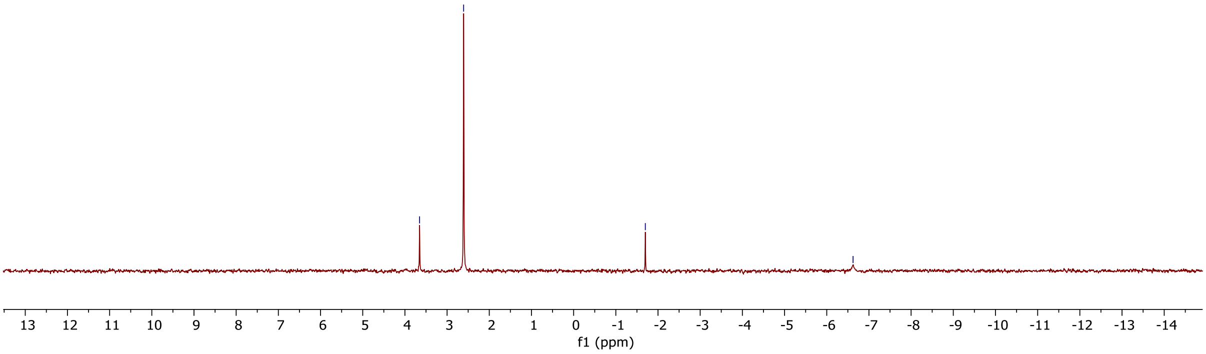

n(OTf⁻)	m (sample)	Yield of F⁻ %	Yield of PO_3F^{2-} %
0.070 mmol	43.6 mg	7%	72%

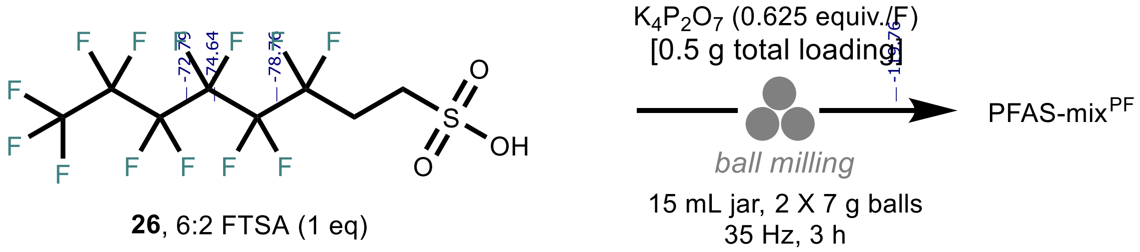

^{19}F NMR (377 MHz, 90% H_2O + 10% D_2O)

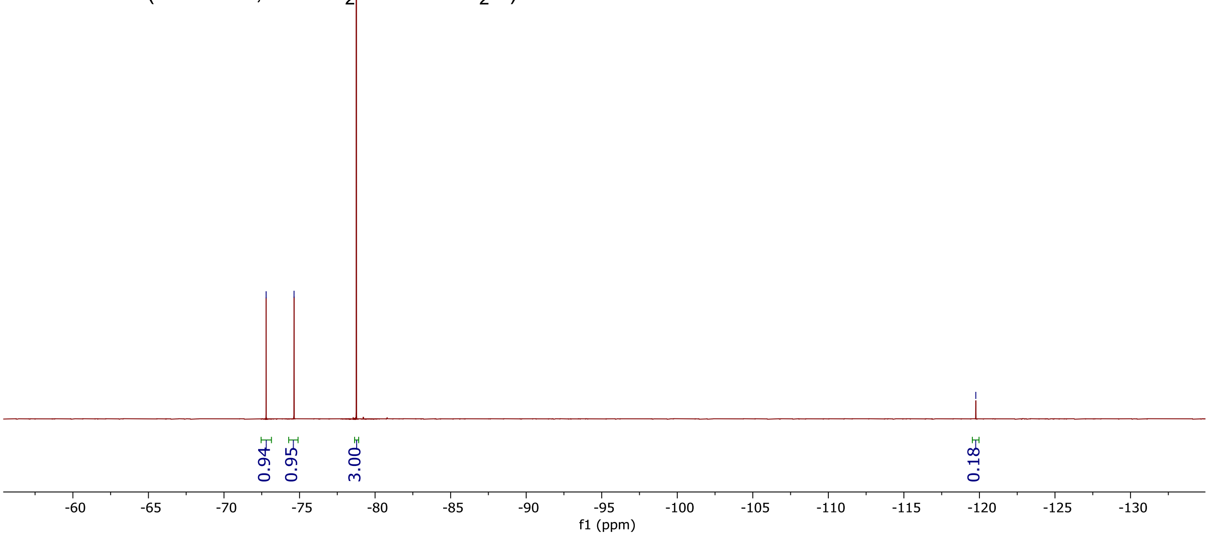

^{31}P NMR (162 MHz, 90% H_2O + 10% D_2O)

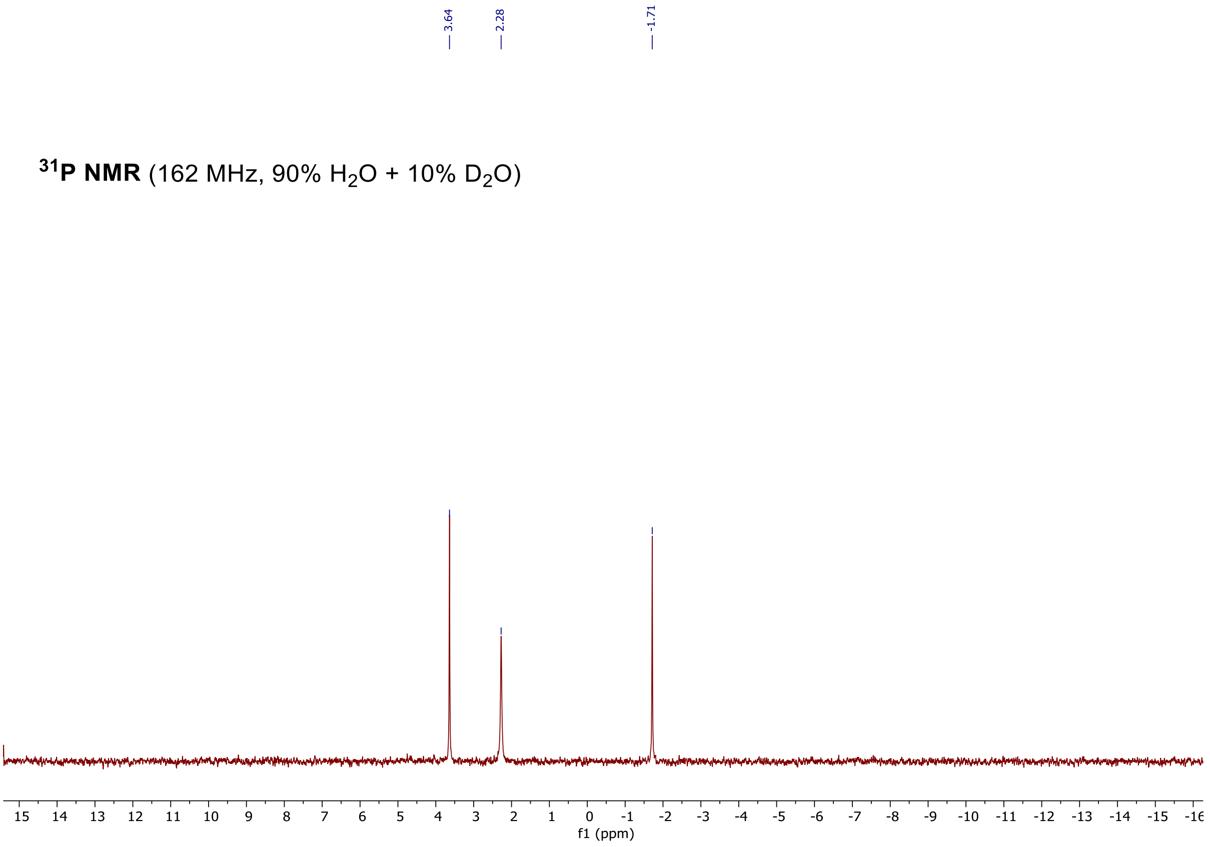

n(OTf⁻)	m (sample)	Yield of F⁻ %	Yield of PO_3F^{2-} %
0.067 mmol	56.7 mg	58%	3%

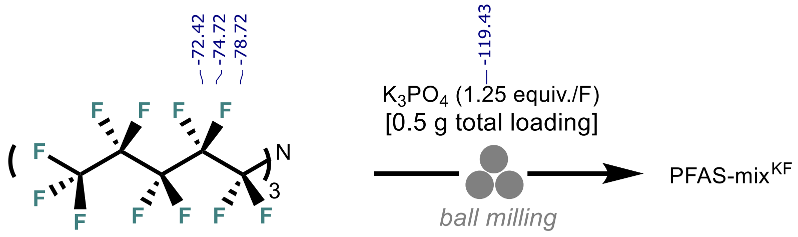

27, *FC-70*

^{19}F NMR (377 MHz, 90% H_2O + 10% D_2O)

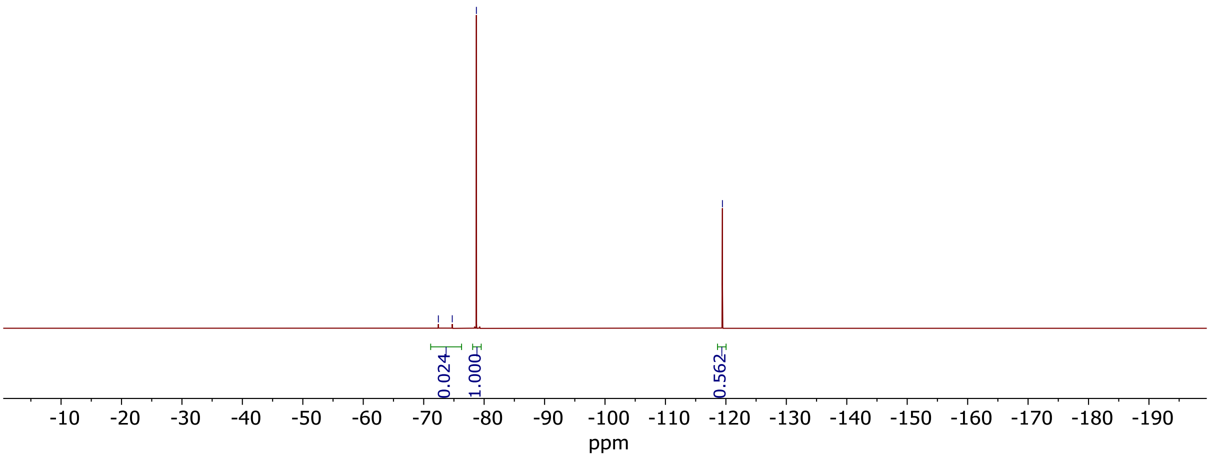

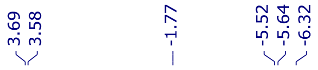

^{31}P NMR (162 MHz, 90% H_2O + 10% D_2O)

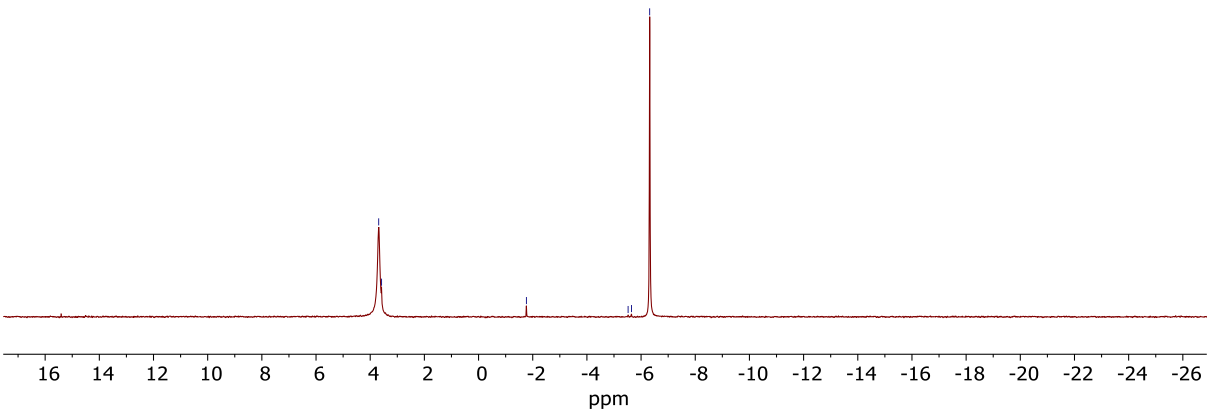

n(OTf⁻)	m (sample)	Yield of F⁻ %	Yield of PO_3F^{2-} %
0.063 mmol	50.7 mg	4%	27%

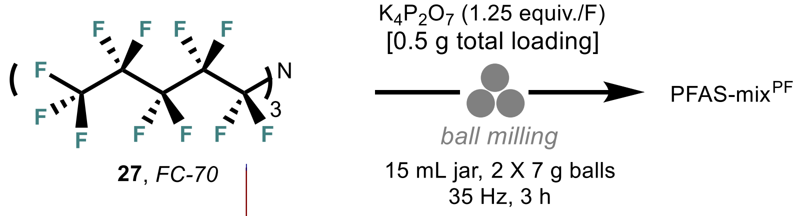

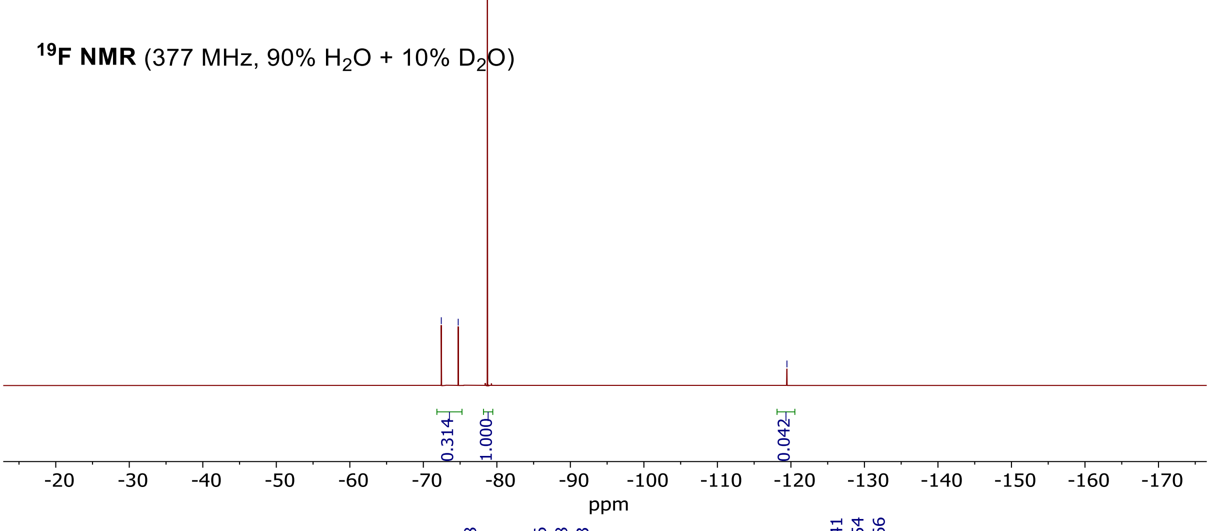

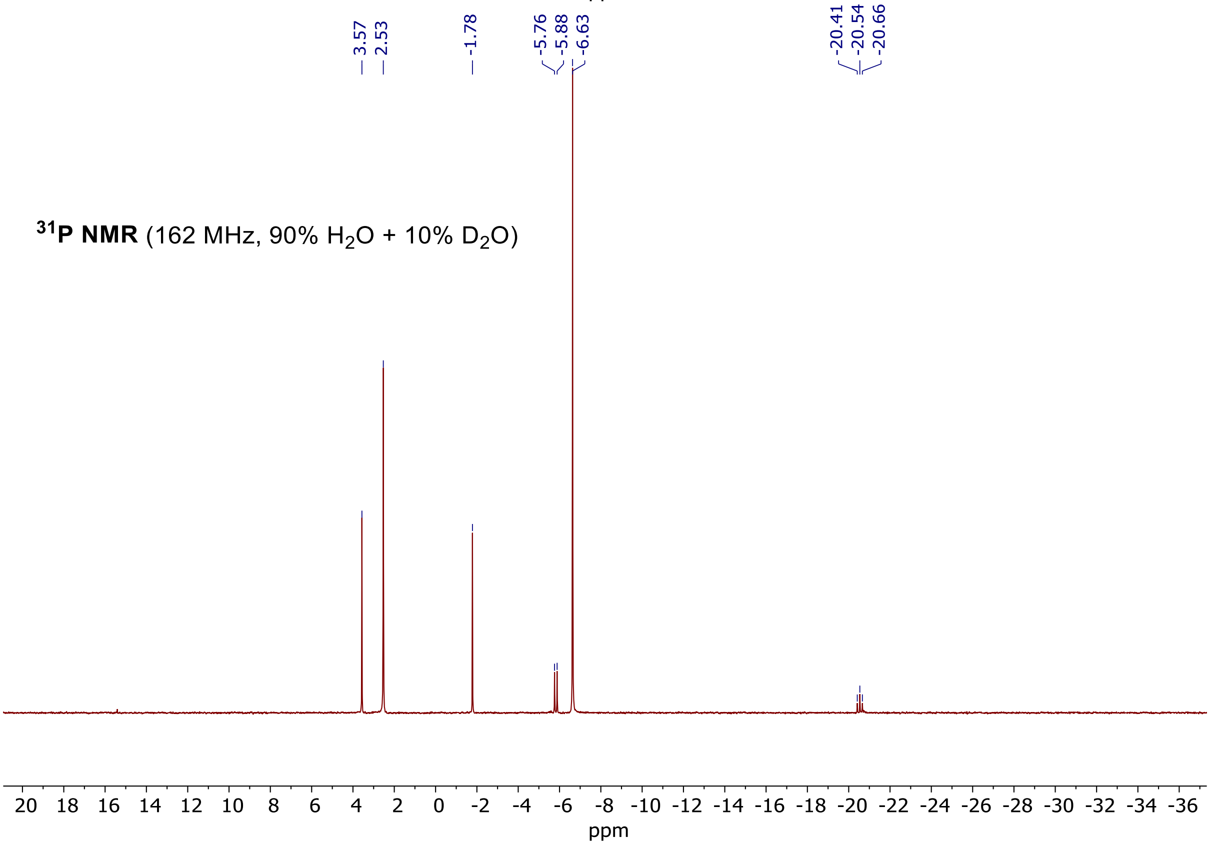

15. Reagents and fluorochemicals NMR characterization

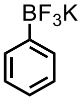

^{1}H NMR (400 MHz, DMSO-d6)

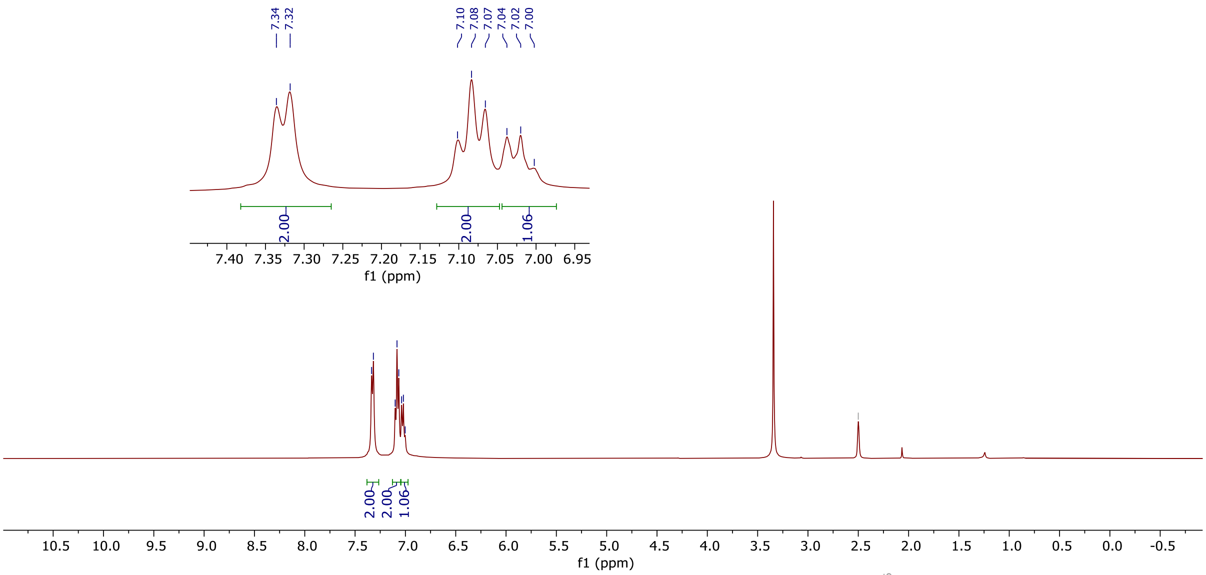

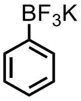

^{13}C NMR (101 MHz, DMSO-d6)

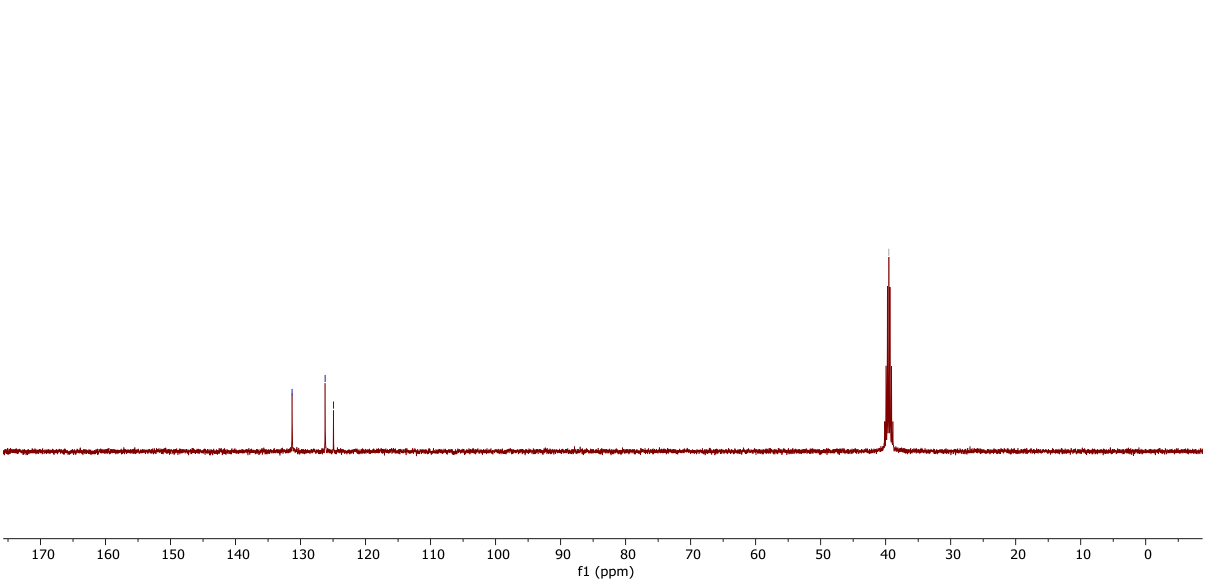

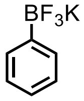

^{19}F NMR (377 MHz, DMSO-d6)

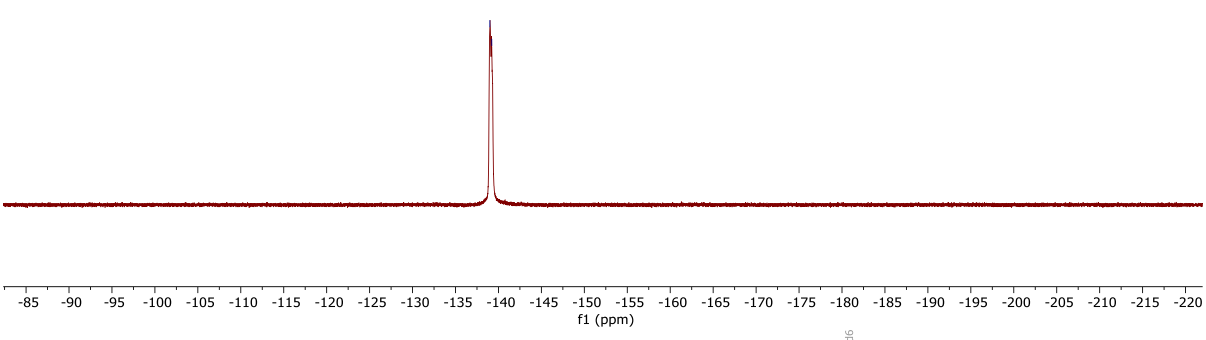

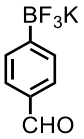

^{1}H NMR (400 MHz, DMSO-d6)

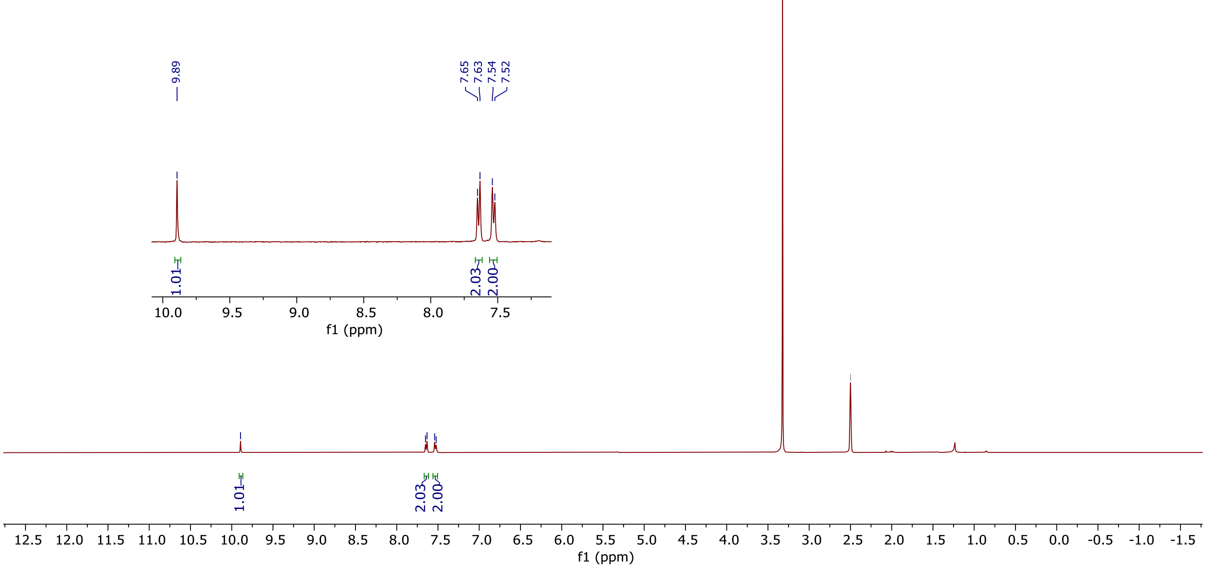

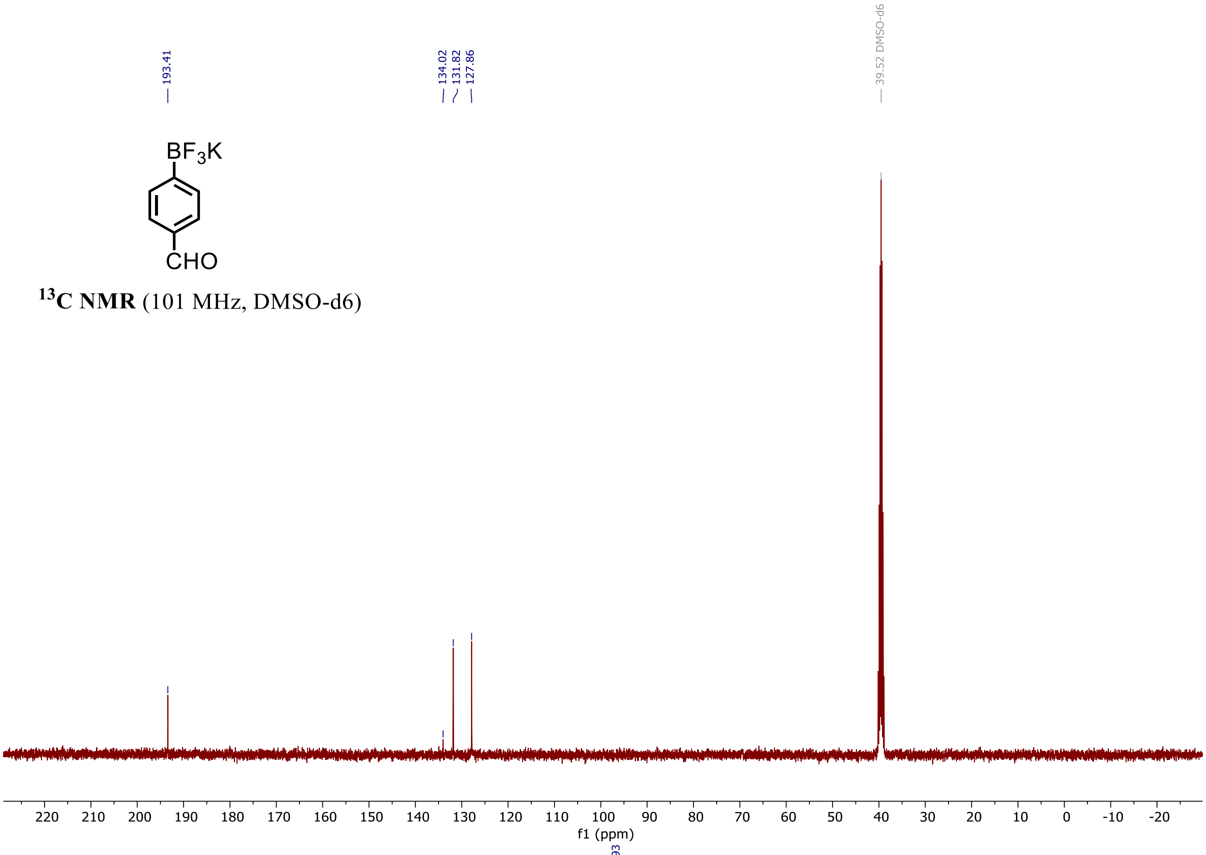

13C NMR (101 MHz, DMSO-d6)

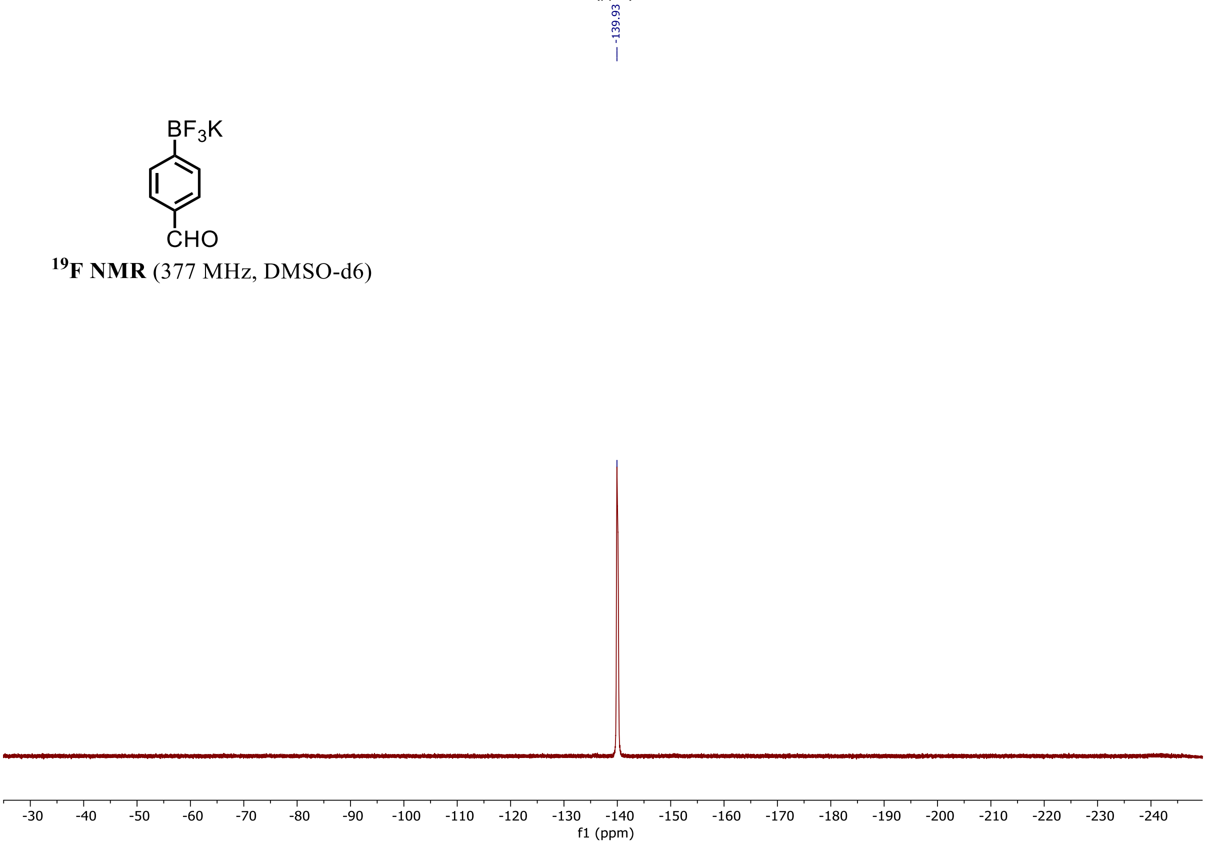

19F NMR (377 MHz, DMSO-d6)

TMAF

^{1}H NMR (400 MHz, D_2O)

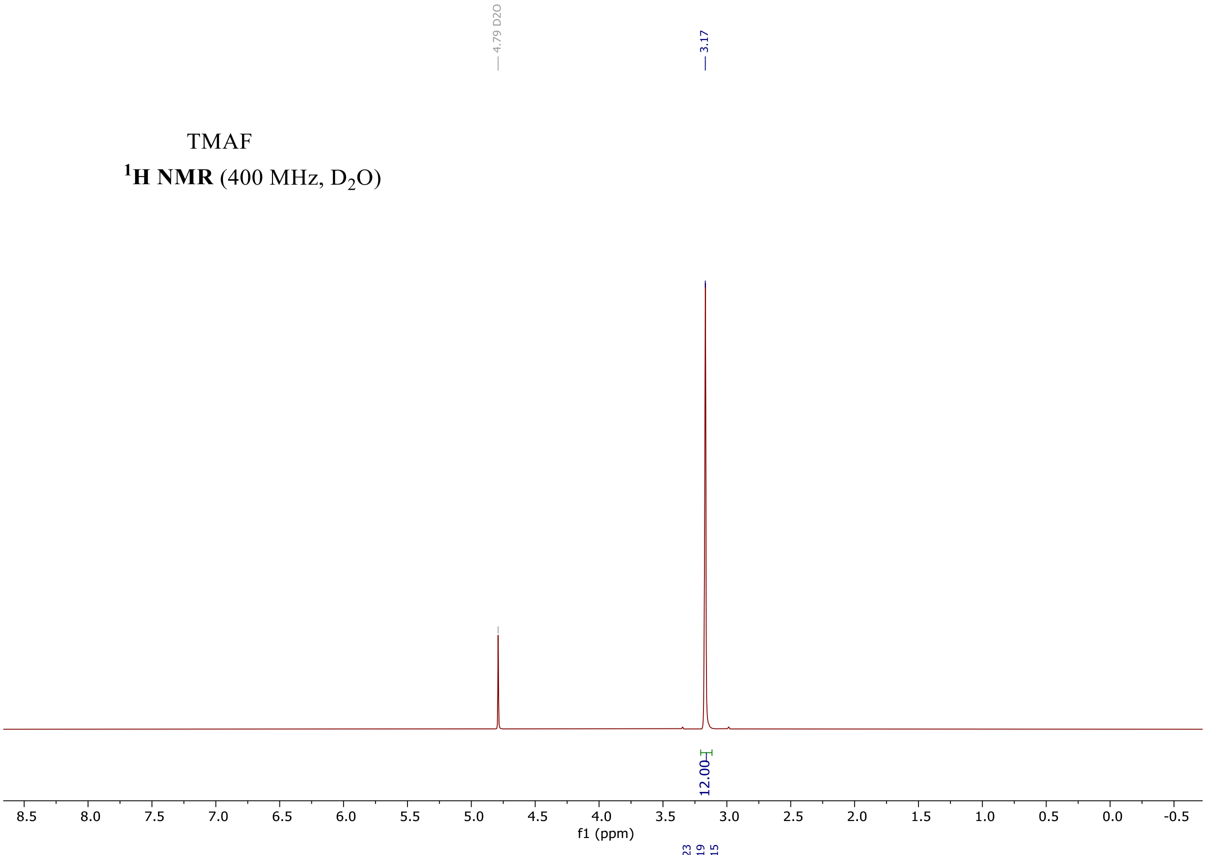

TMAF

^{13}C NMR (101 MHz, D_2O)

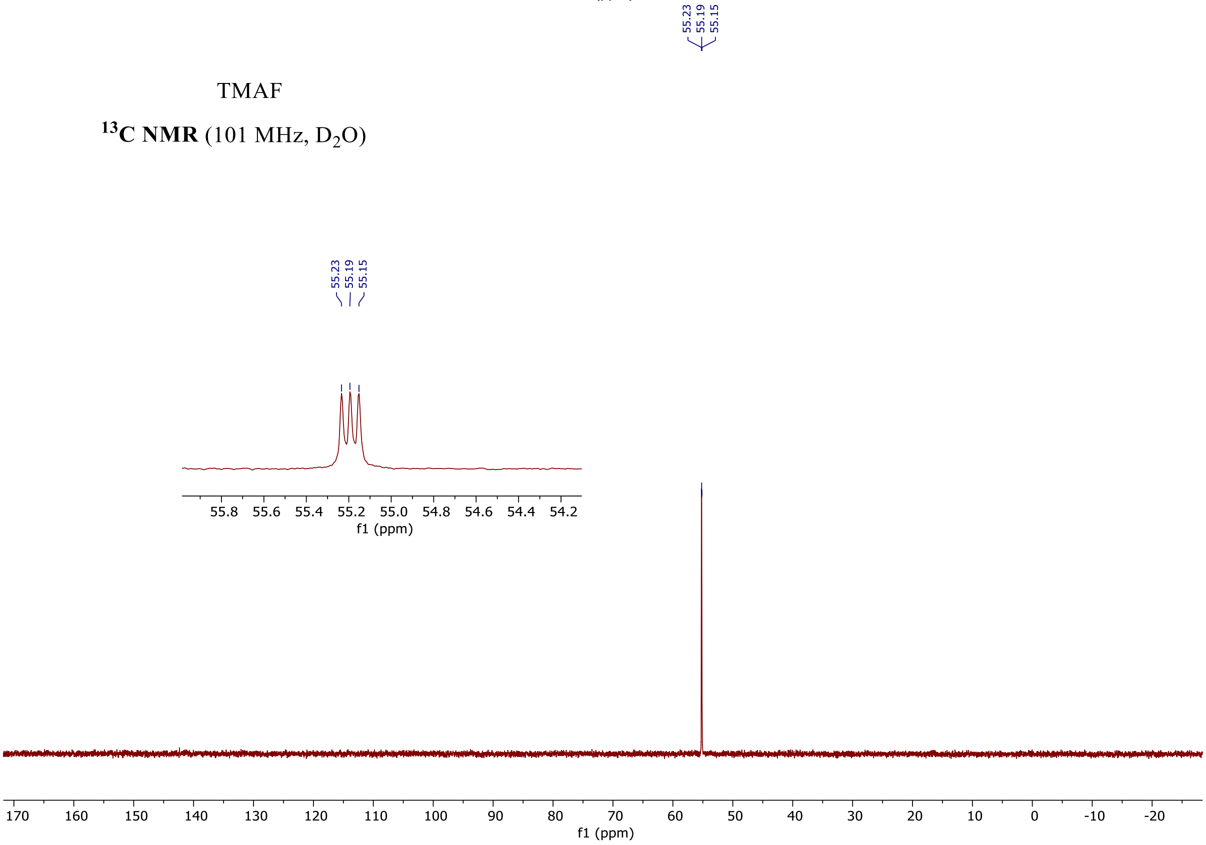

TMAF
^{19}F NMR (377 MHz, D_2O)

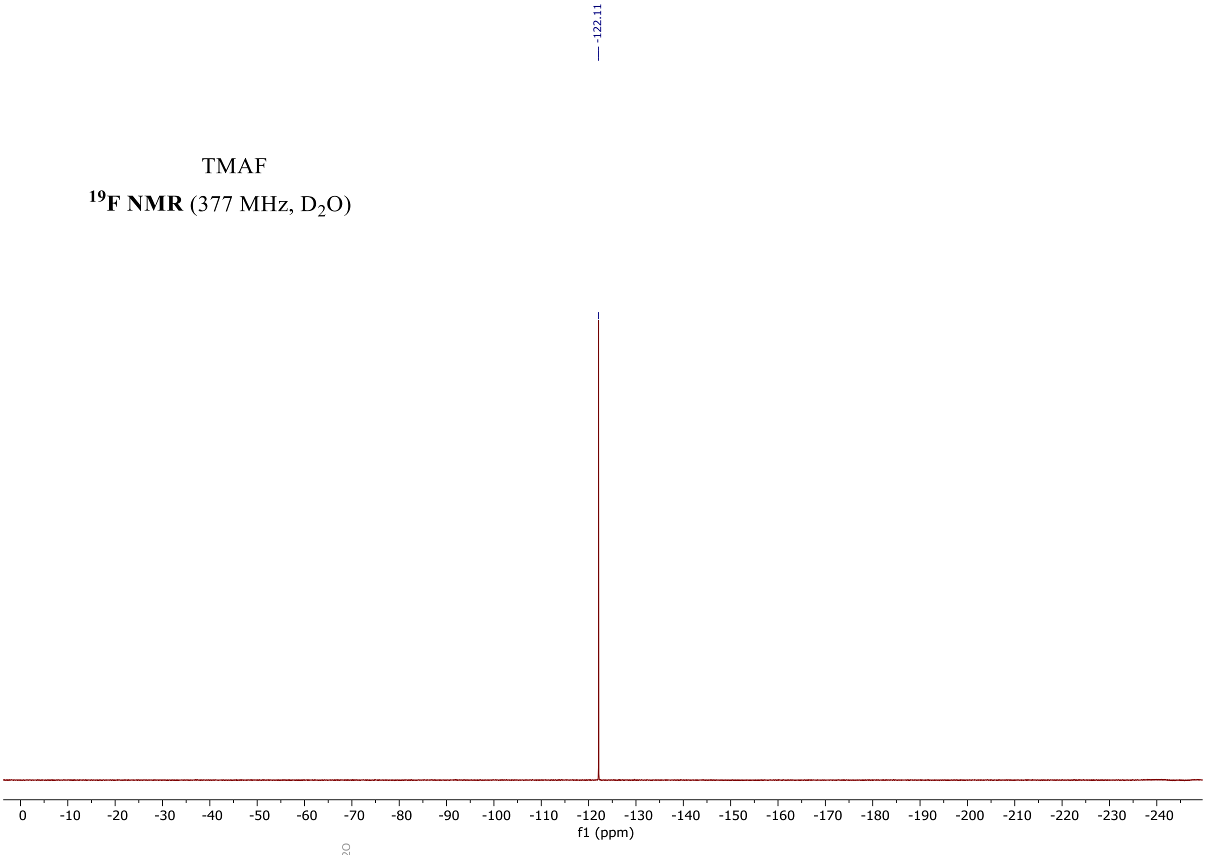

TMAF(*t*AmylOH)
^{1}H NMR (400 MHz, D_2O

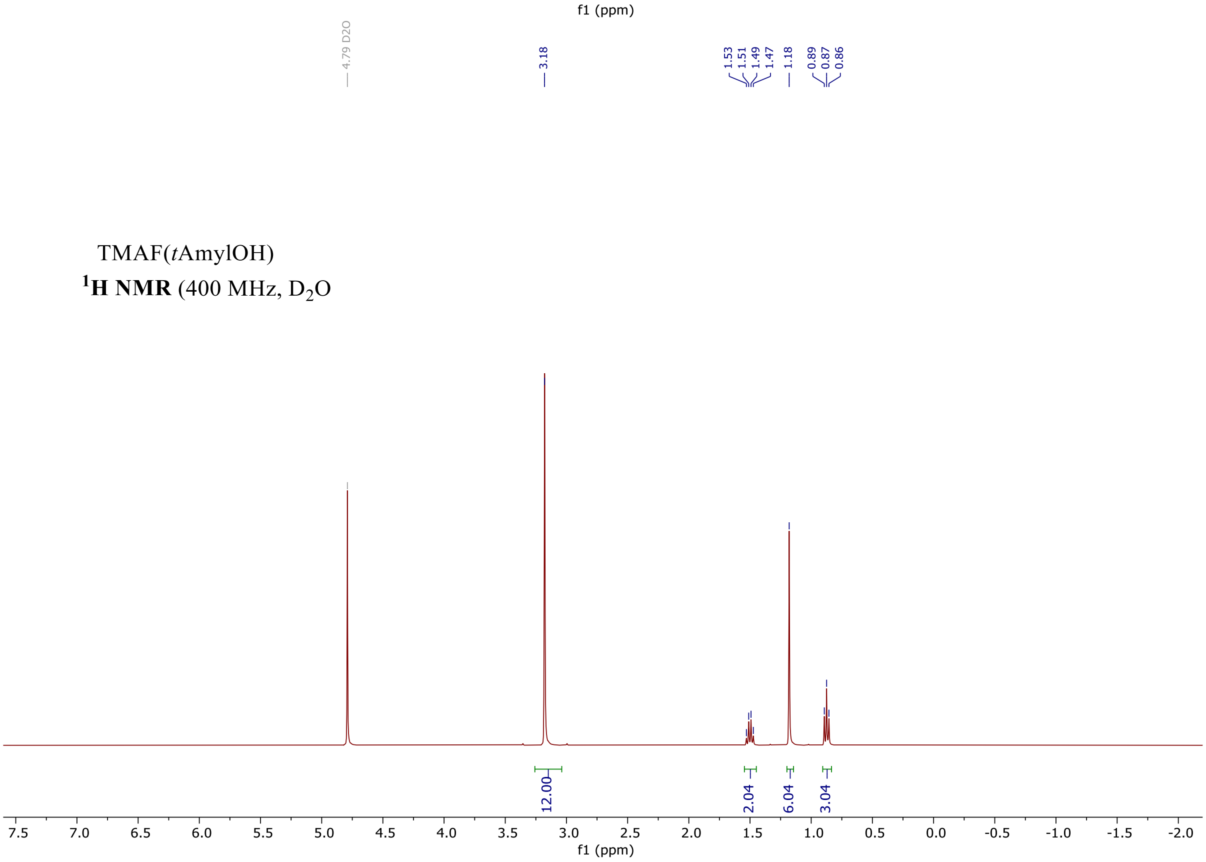

TMAF(*t*AmylOH)
^{13}C NMR (101 MHz, D_2O)

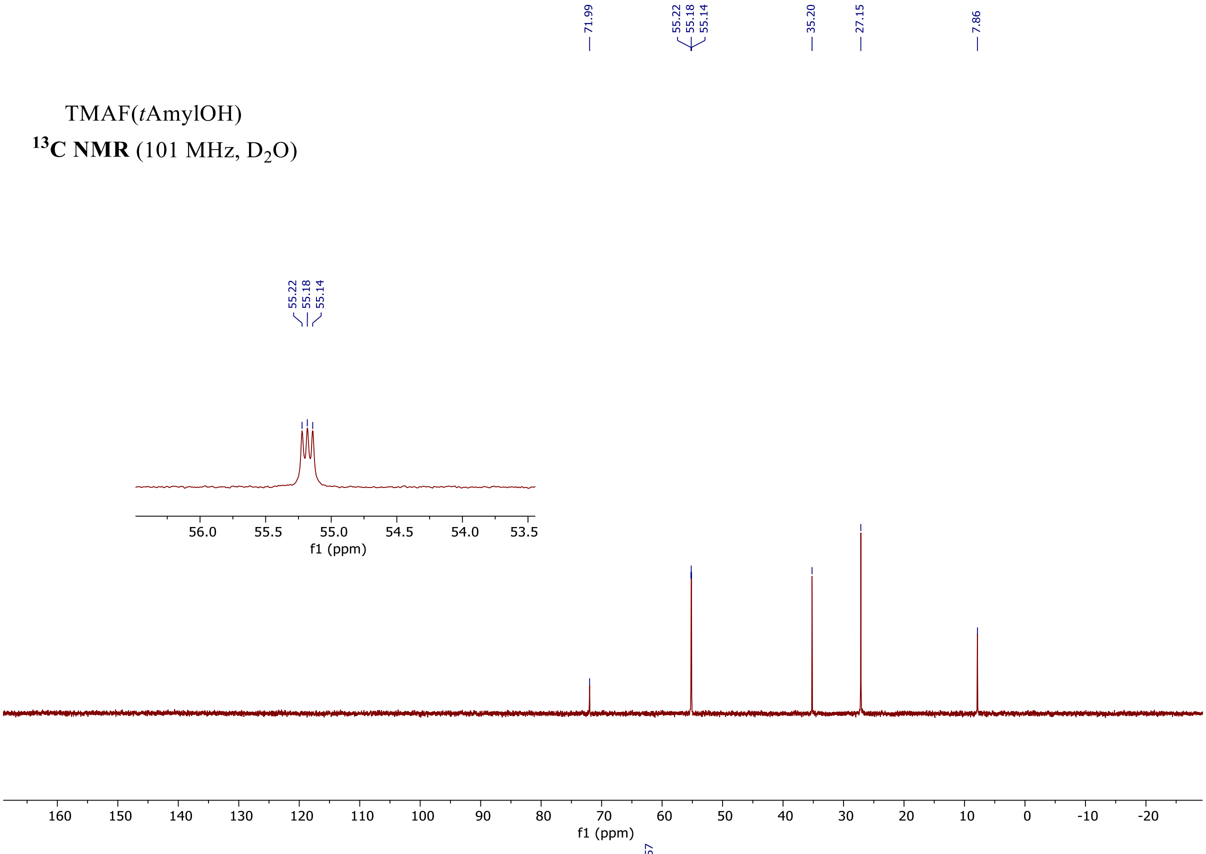

TMAF(*t*AmylOH)
^{19}F NMR (377 MHz, D_2O)

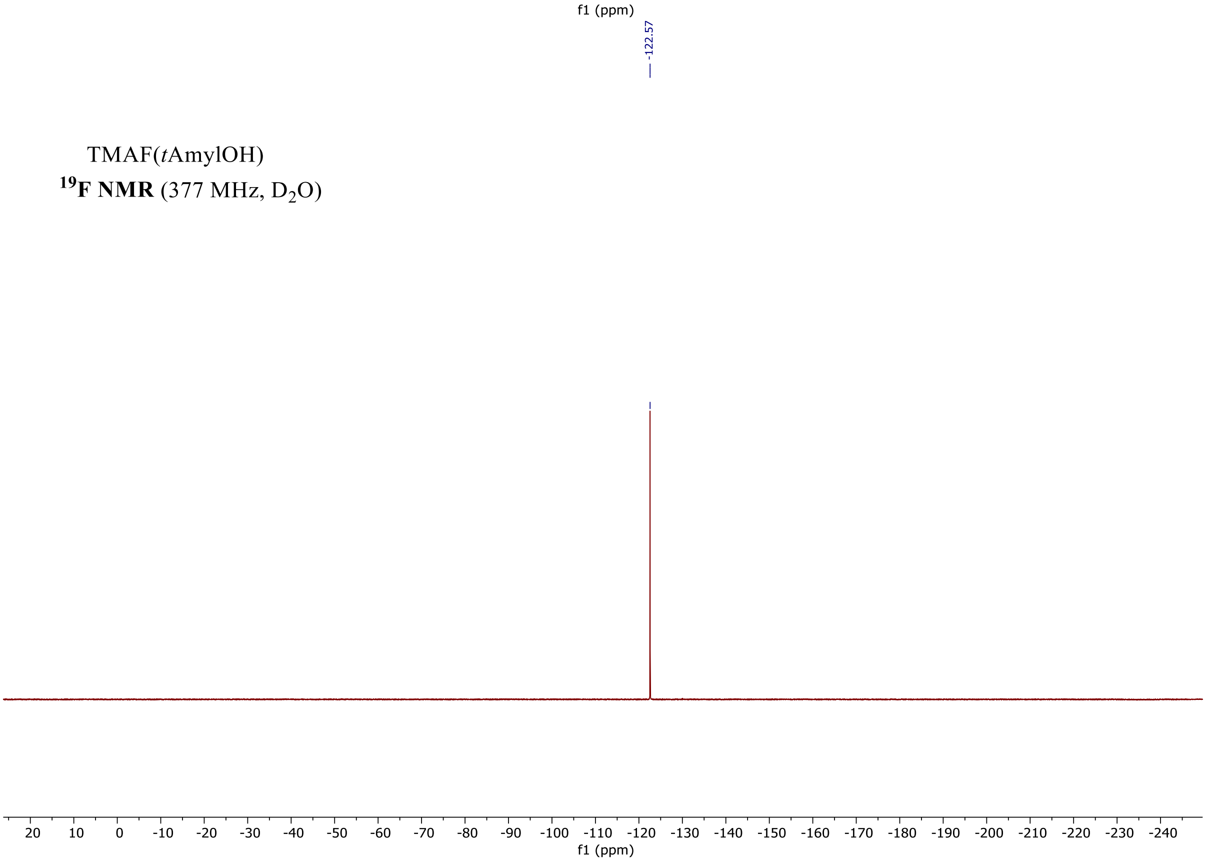

TBAF(tBuOH)$_4$
^{1}H NMR (400 MHz, $CDCl_3$)

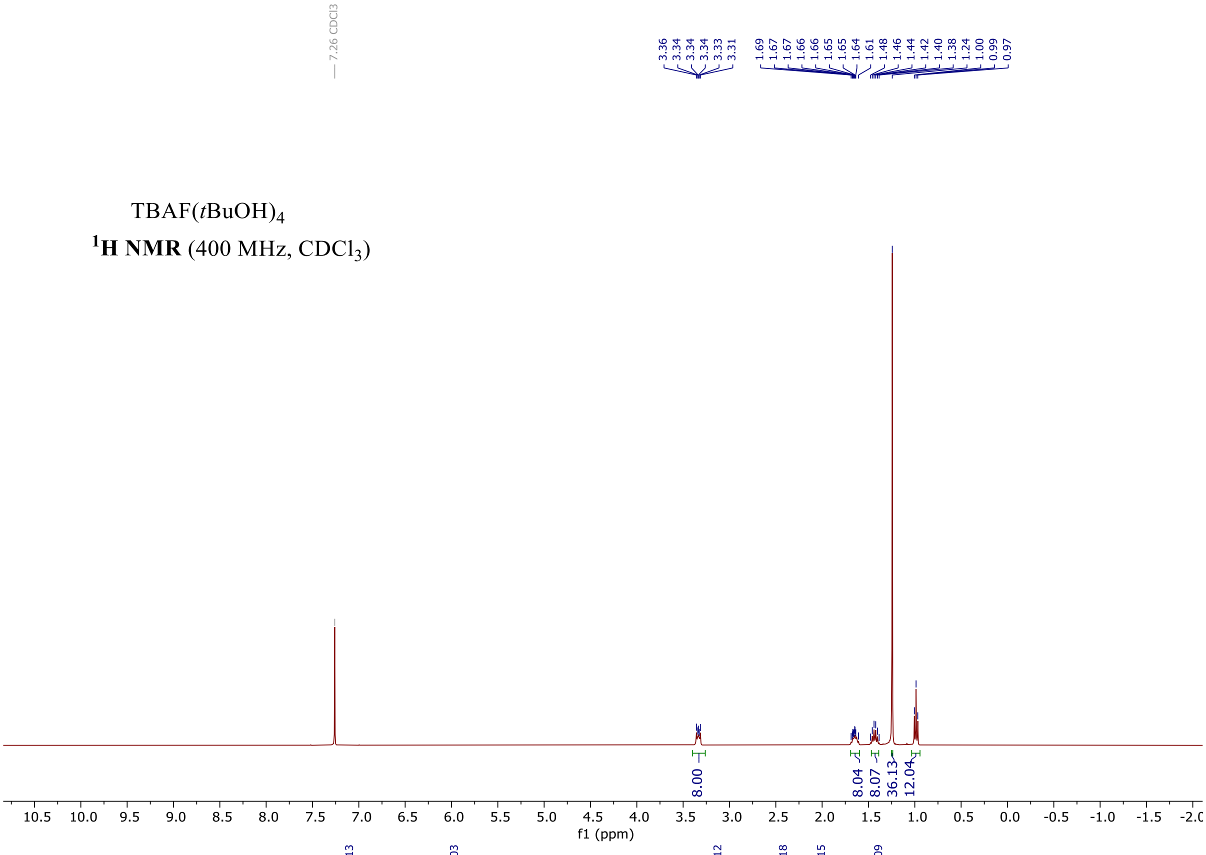

TBAF(tBuOH)$_4$
^{13}C NMR (101 MHz, D_2O)

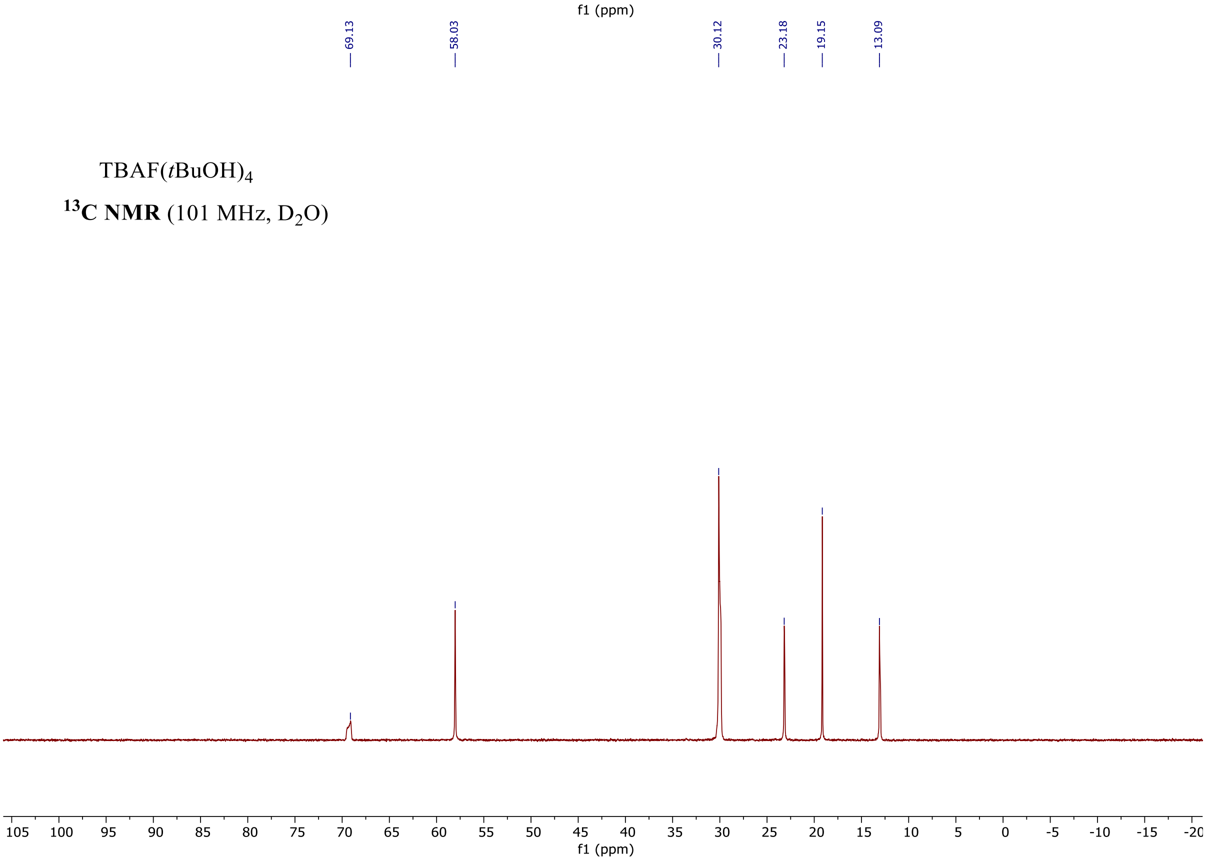

TBAF(tBuOH)$_4$

^{19}F NMR (377 MHz, D_2O)

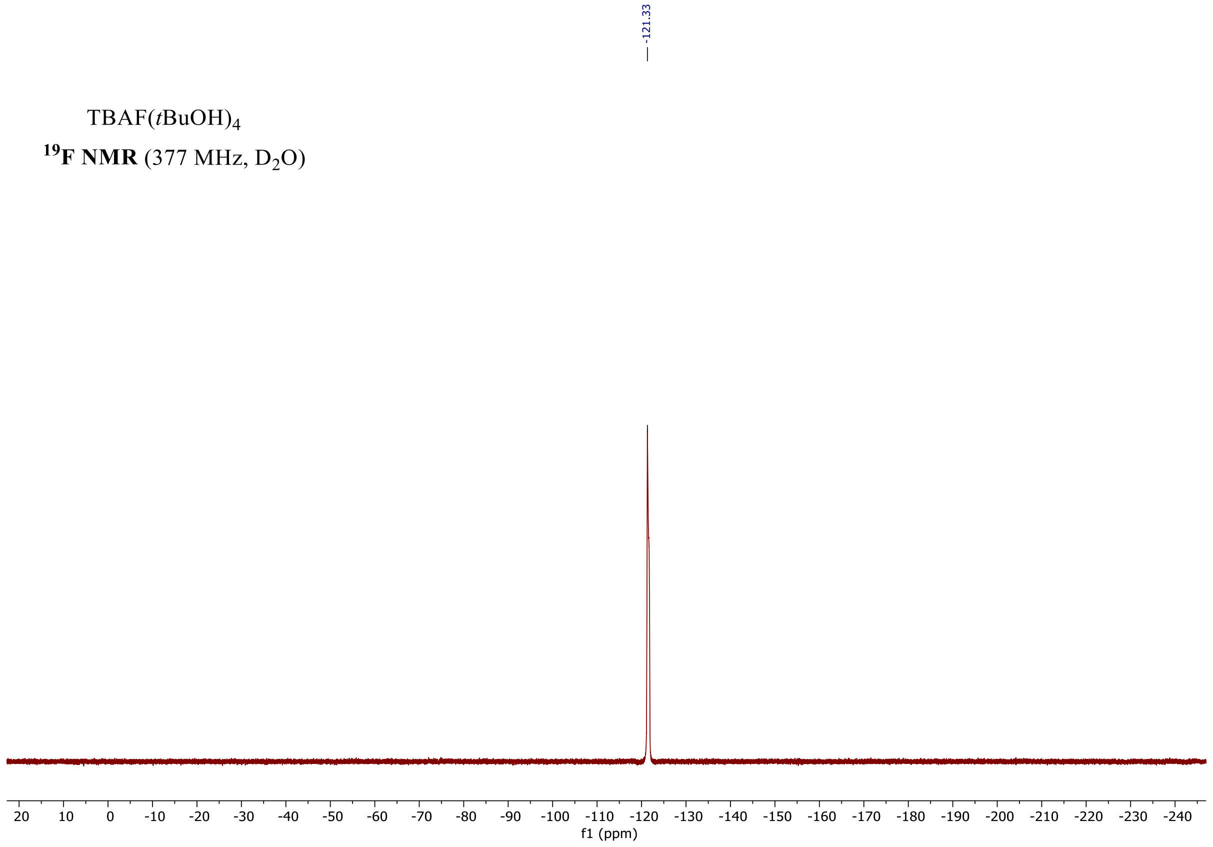

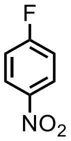

28

^{1}H NMR (400 MHz, $CDCl_3$)

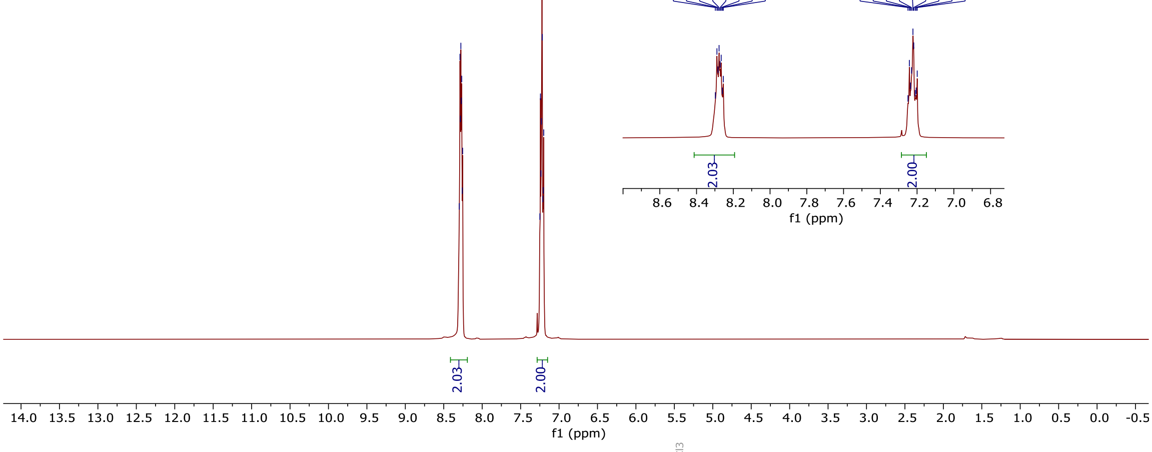

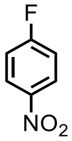

28

^{13}C NMR (101 MHz, $CDCl_3$)

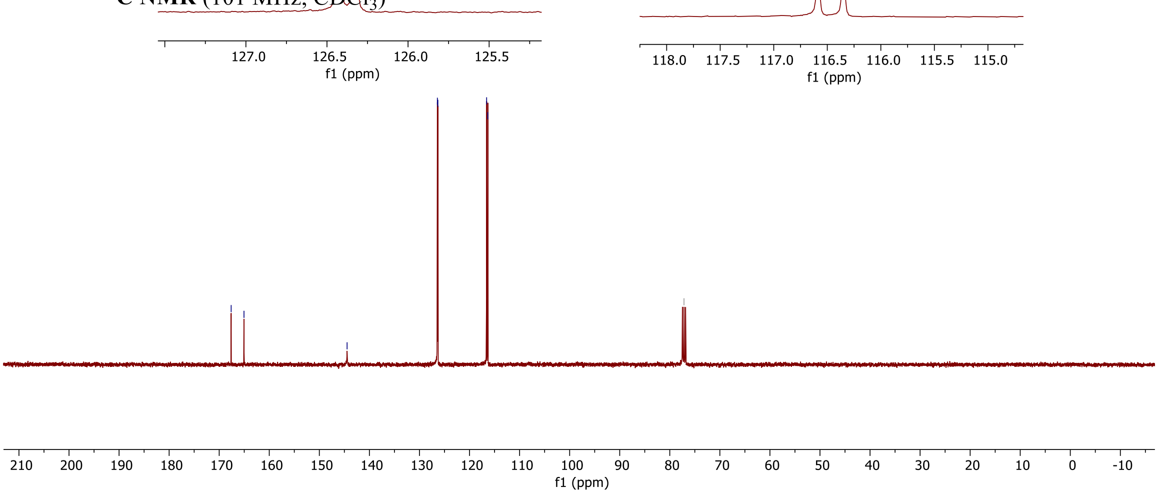

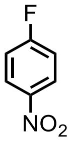

28

^{19}F NMR (377 MHz, $CDCl_3$)

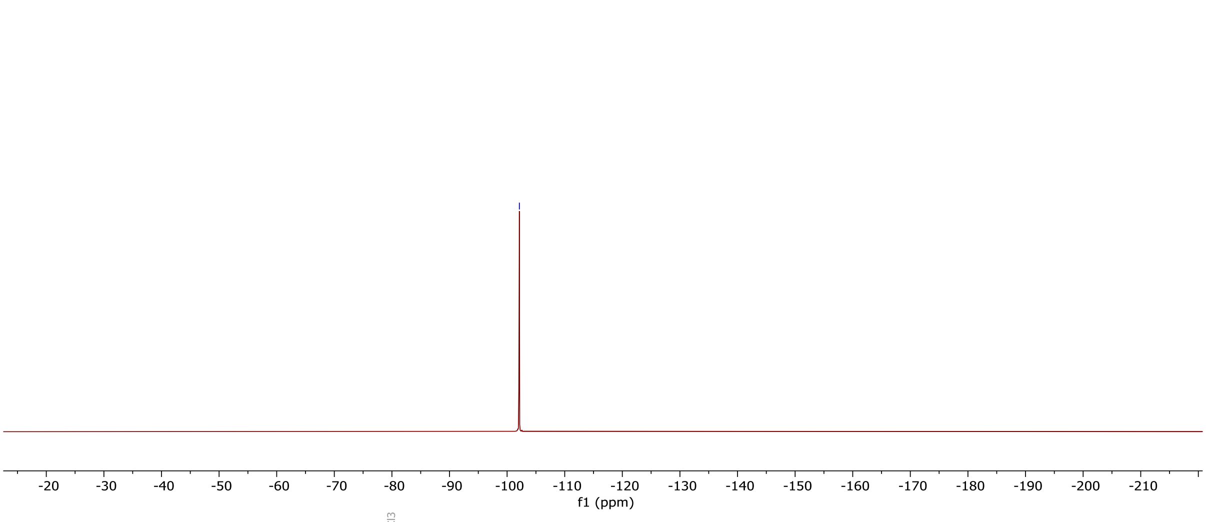

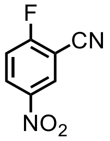

29

^{1}H NMR (400 MHz, $CDCl_3$)

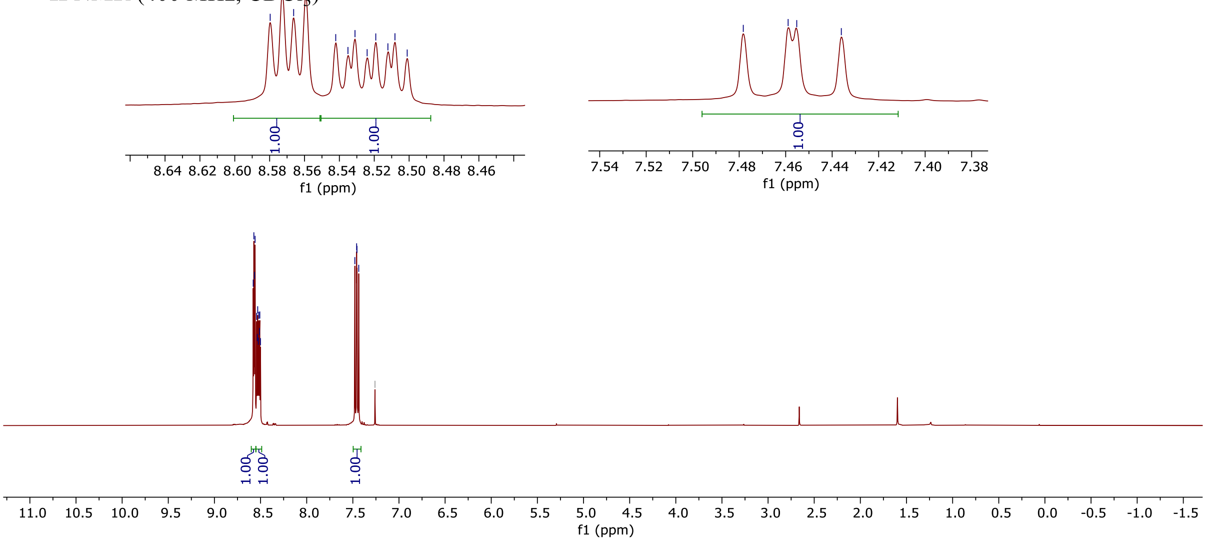

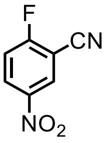

29

^{13}C NMR (101 MHz, $CDCl_3$)

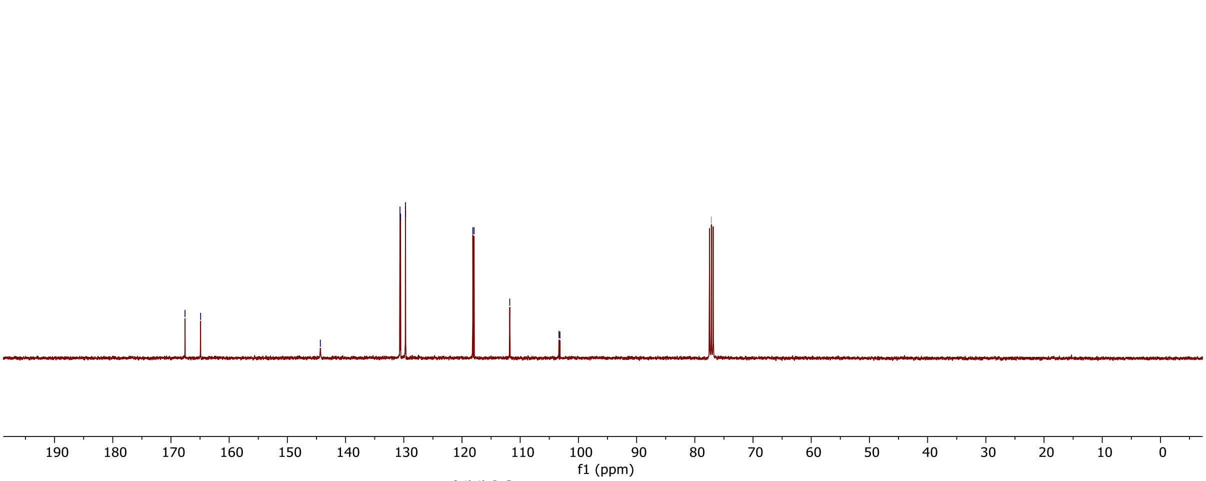

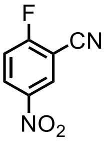

29

^{19}F NMR (377 MHz, $CDCl_3$)

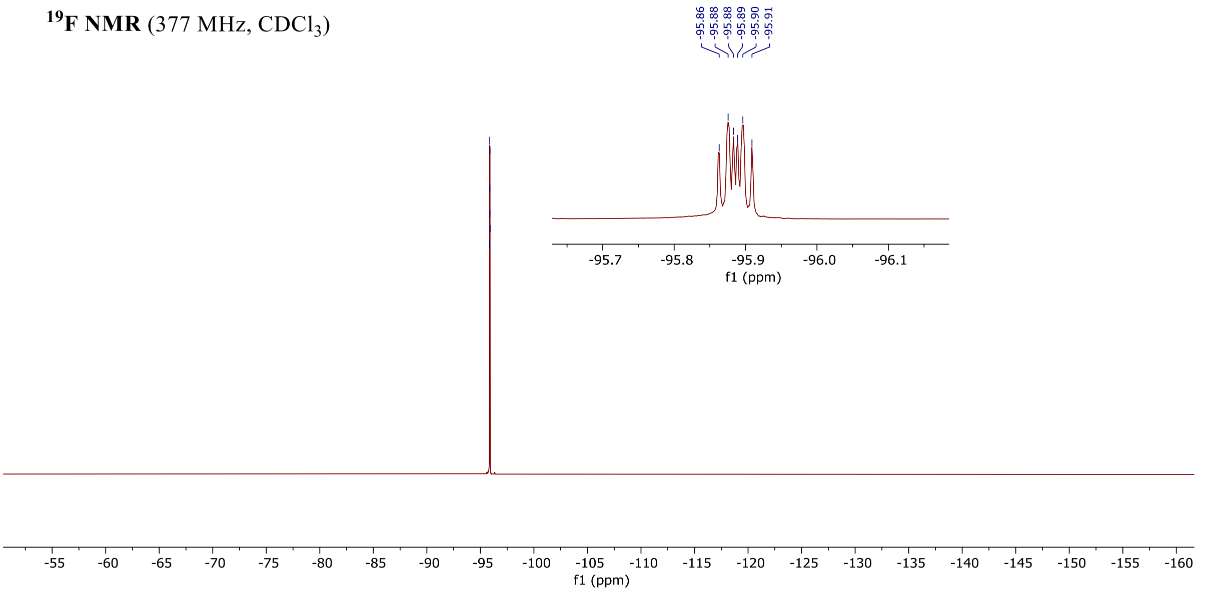

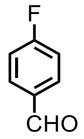

30

^{1}H NMR (400 MHz, $CDCl_3$)

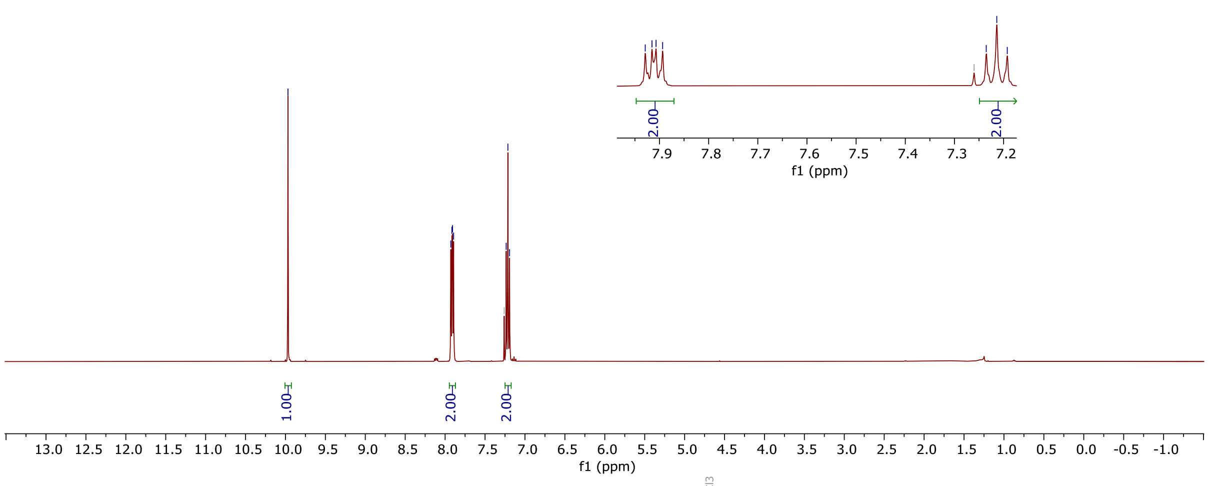

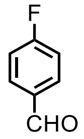

30

^{13}C NMR (101 MHz, $CDCl_3$)

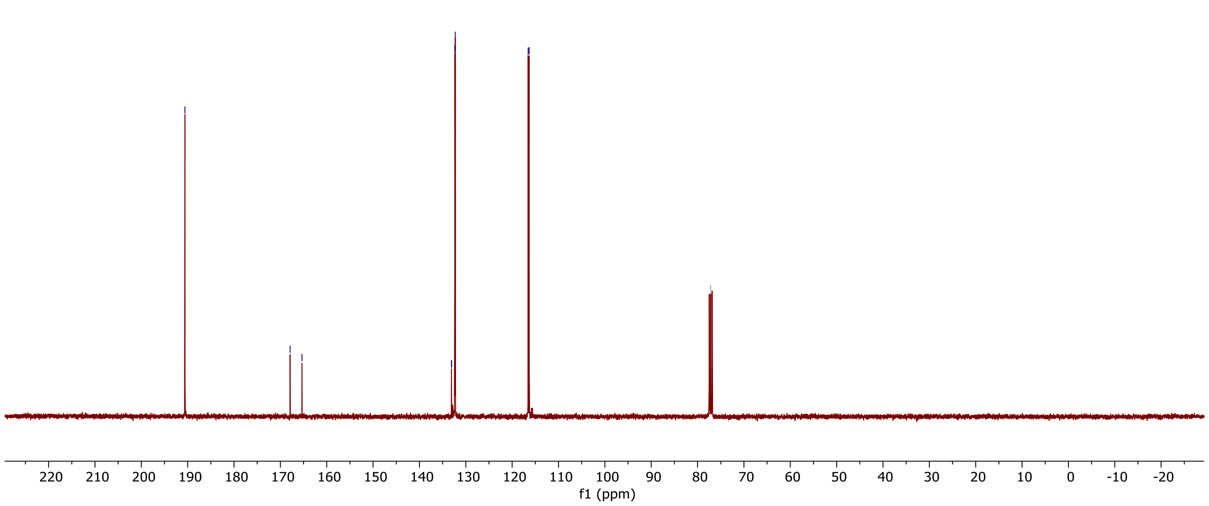

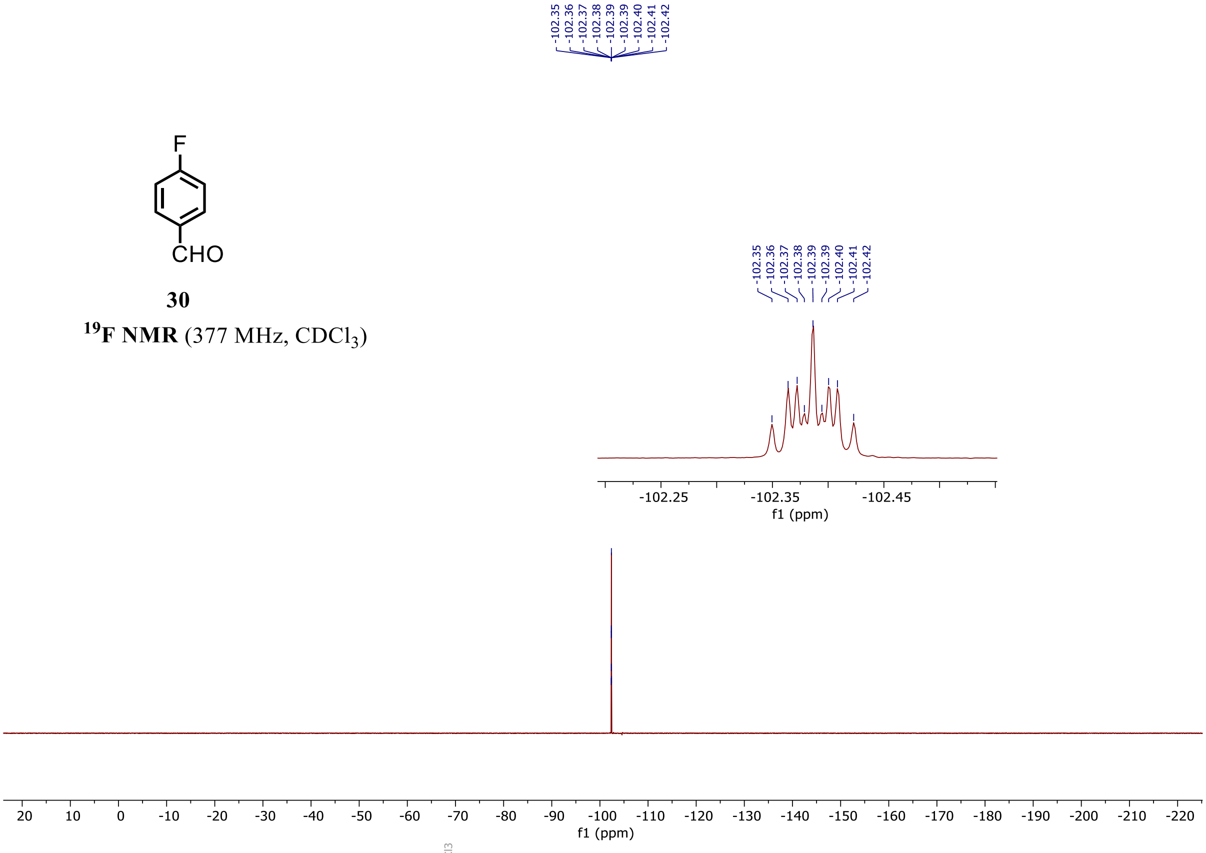

30

^{19}F NMR (377 MHz, $CDCl_3$)

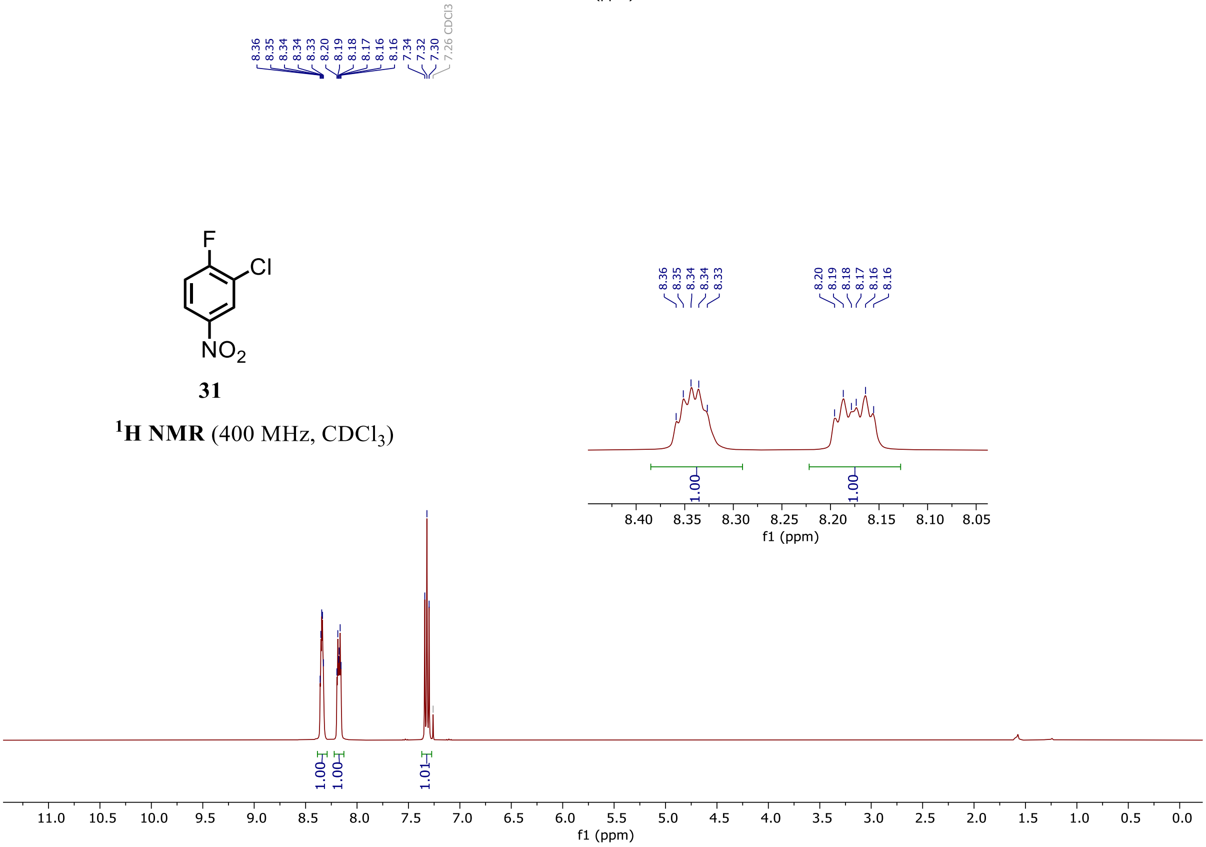

31

^{1}H NMR (400 MHz, $CDCl_3$)

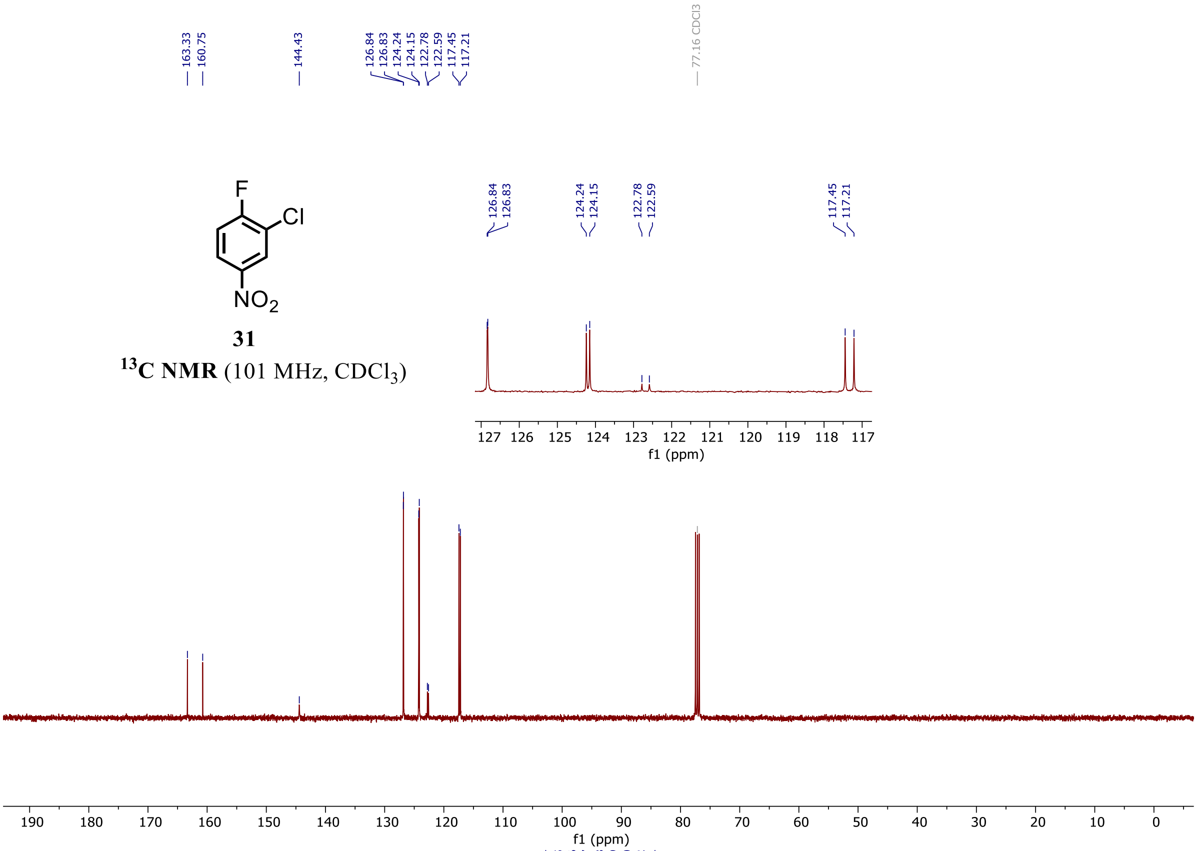

31

^{13}C NMR (101 MHz, $CDCl_3$)

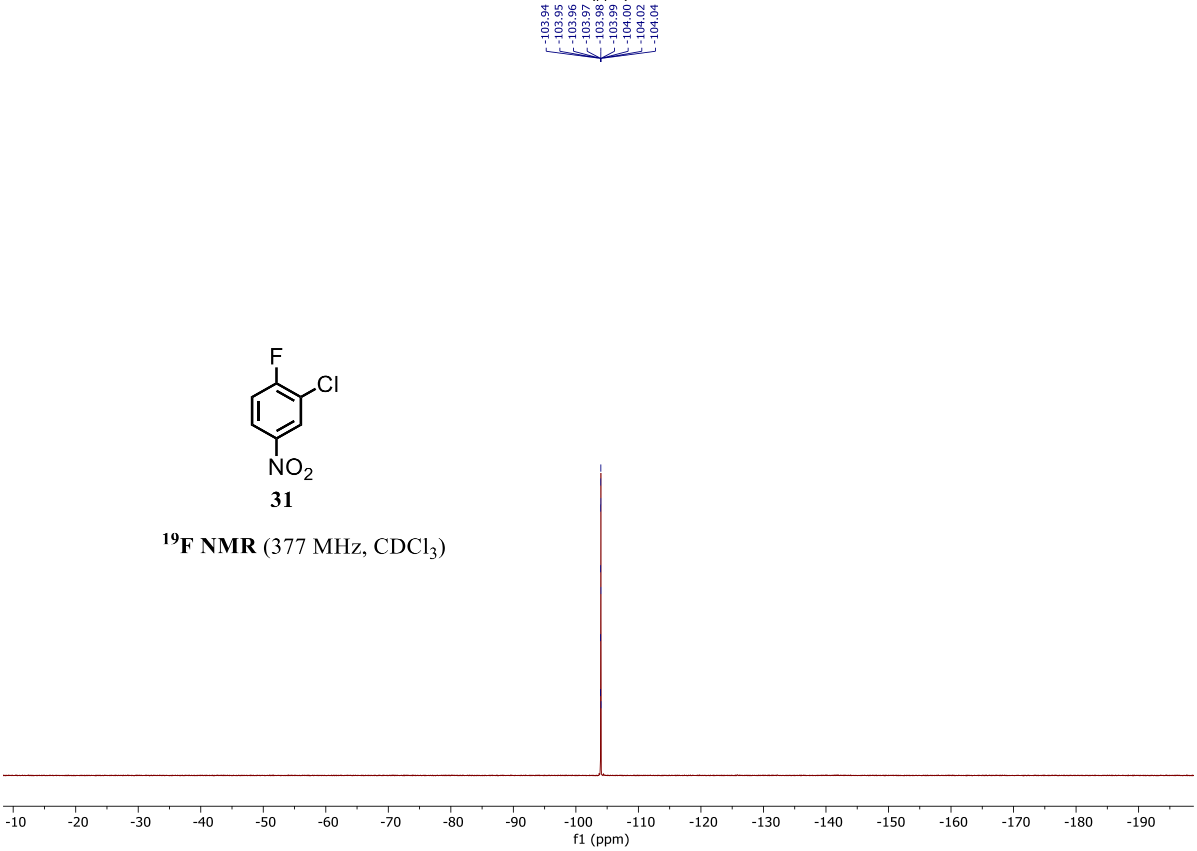

31

^{19}F NMR (377 MHz, $CDCl_3$)

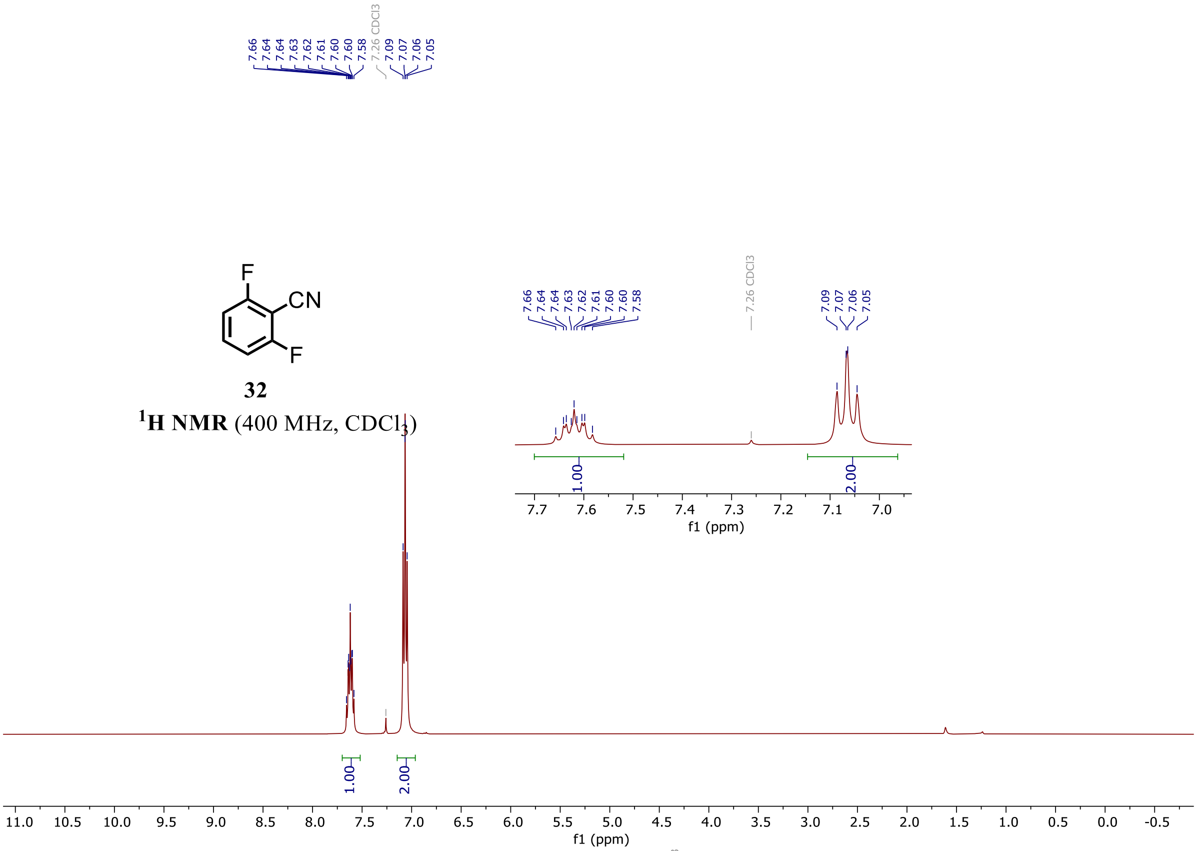
7.66
7.64
7.64
7.63
7.62
7.61
7.60
7.60
7.58
7.26 CDCl3
7.09
7.07
7.06
7.05
F
CN
F
32
1H NMR (400 MHz, CDCl3)
1.00
2.00
f1 (ppm)

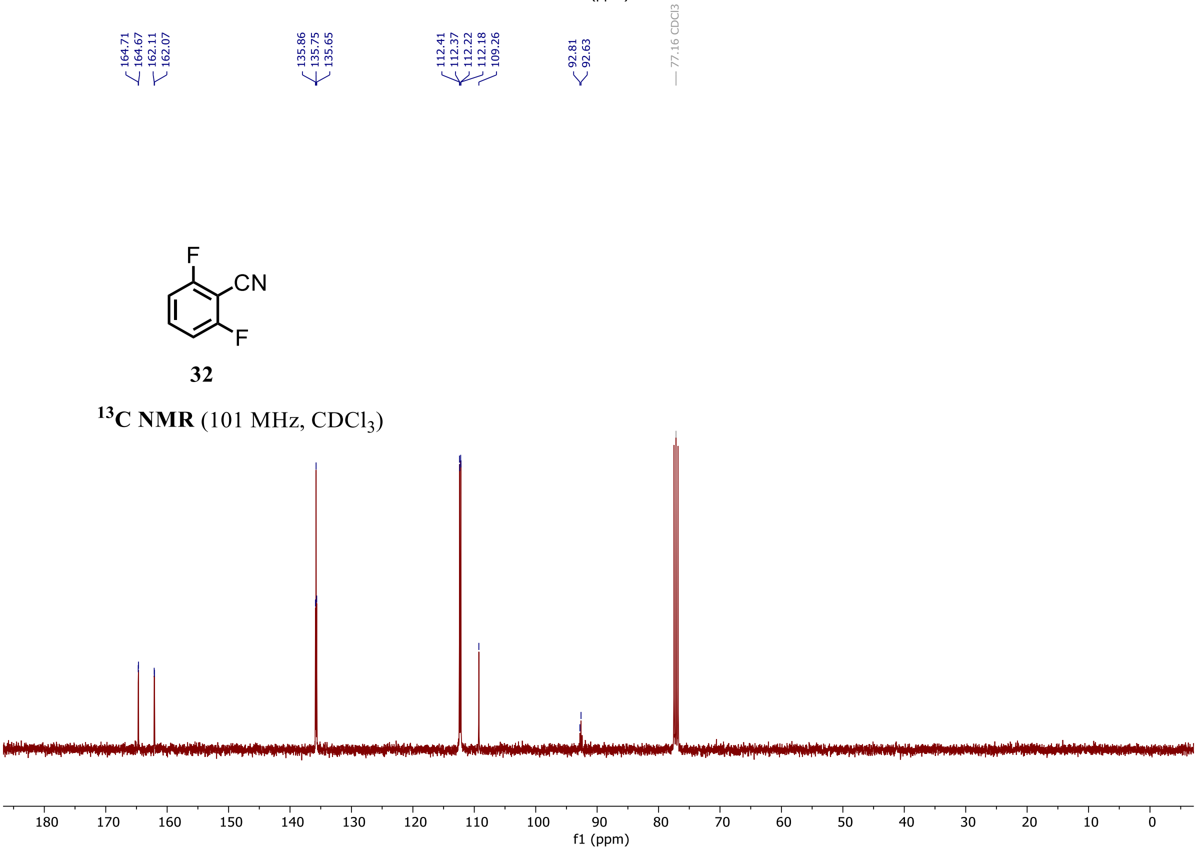
164.71
164.67
162.11
162.07
135.86
135.75
135.65
112.41
112.37
112.22
112.18
109.26
92.81
92.63
77.16 CDCl3
F
CN
F
32
13C NMR (101 MHz, CDCl3)
f1 (ppm)

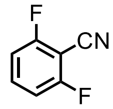

32

^{19}F NMR (377 MHz, $CDCl_3$)

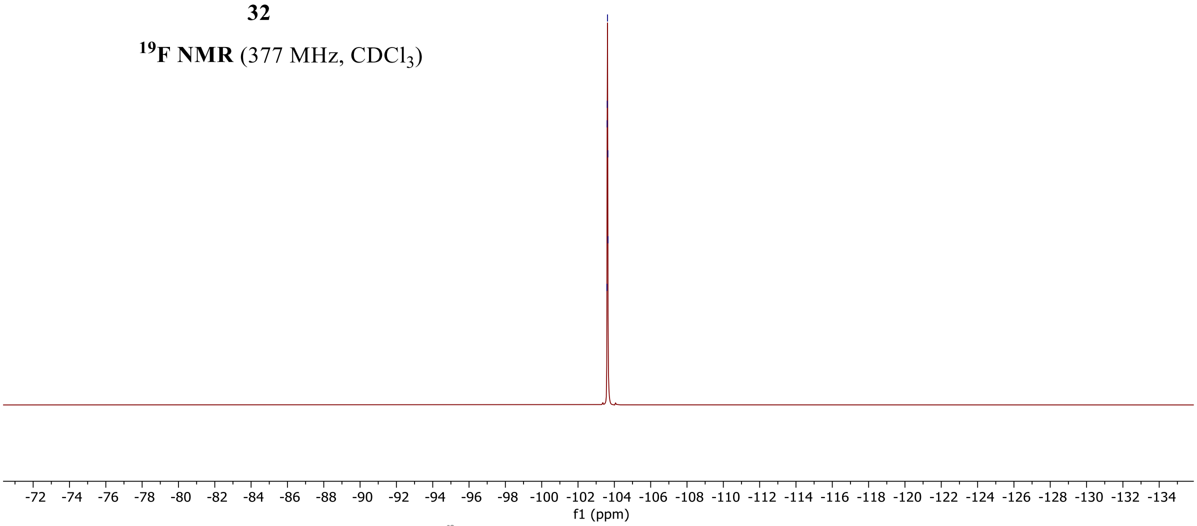

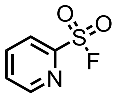

35

^{1}H NMR (400 MHz, $CDCl_3$)

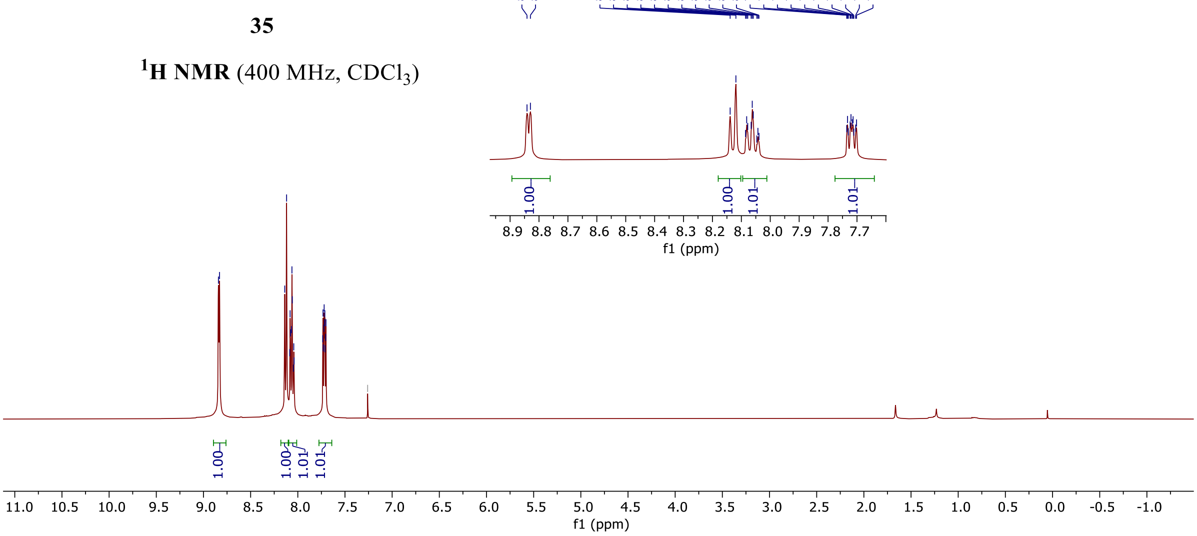

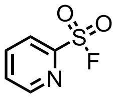

35

^{13}**C NMR** (101 MHz, $CDCl_3$)

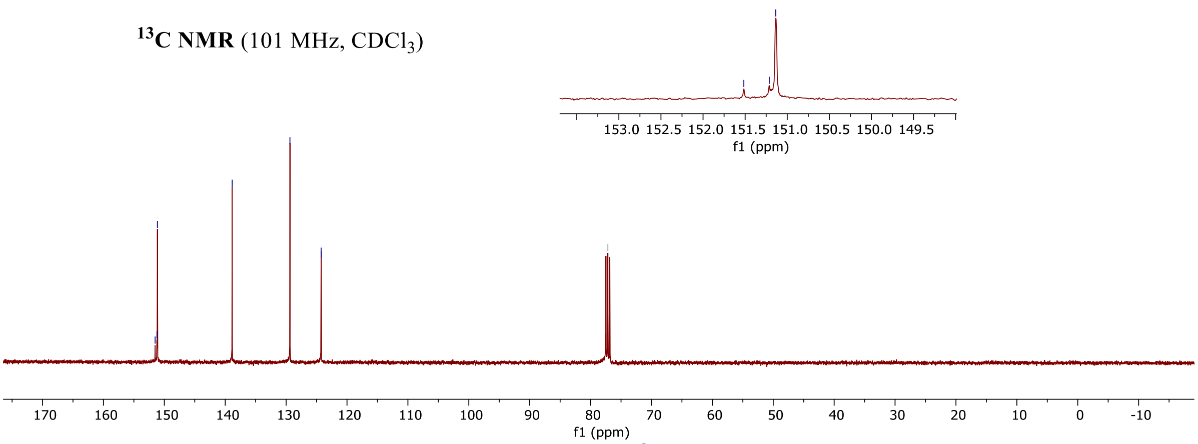

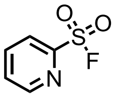

35

^{19}**F NMR** (377 MHz, $CDCl_3$)

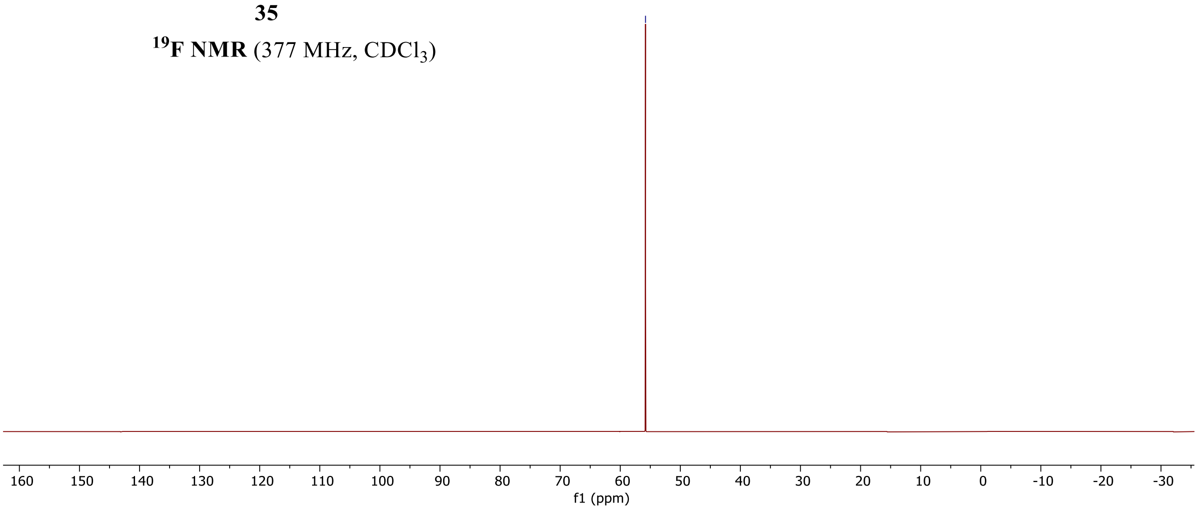

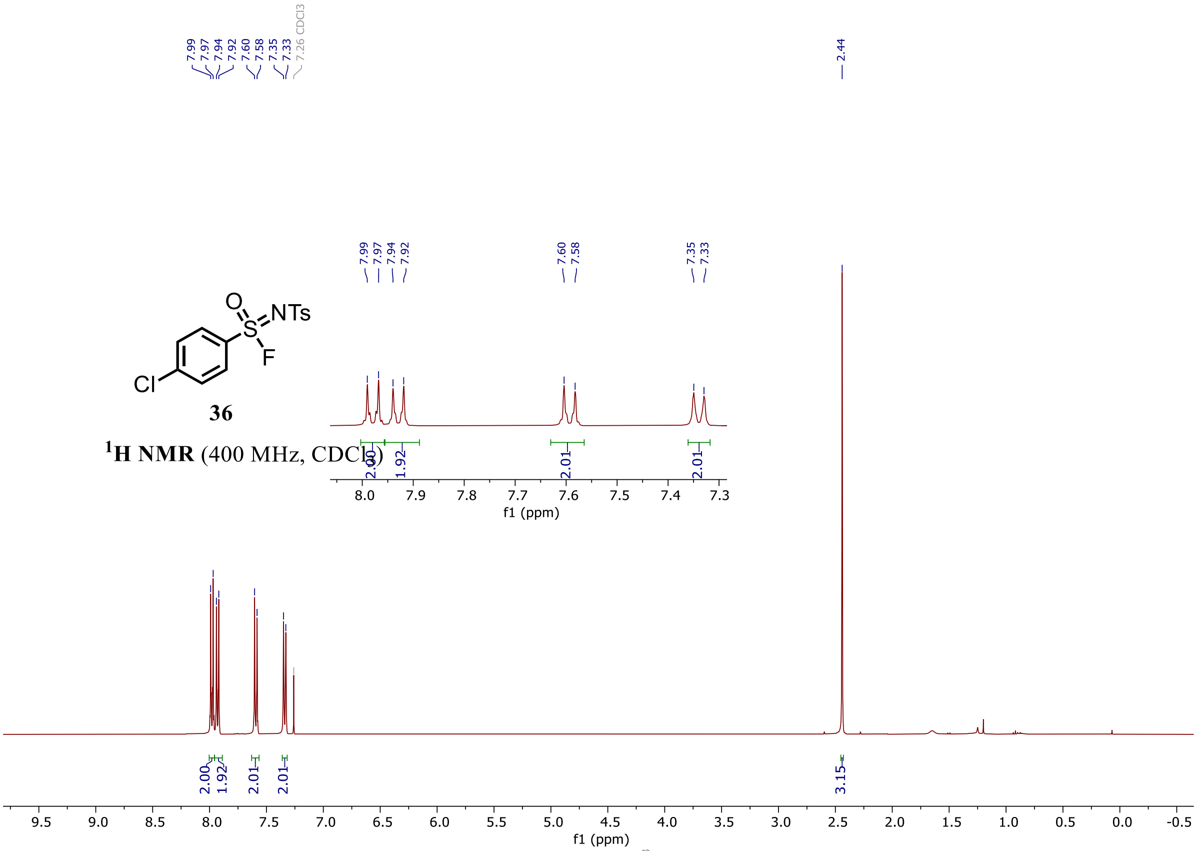

36

^{1}H NMR (400 MHz, $CDCl_3$)

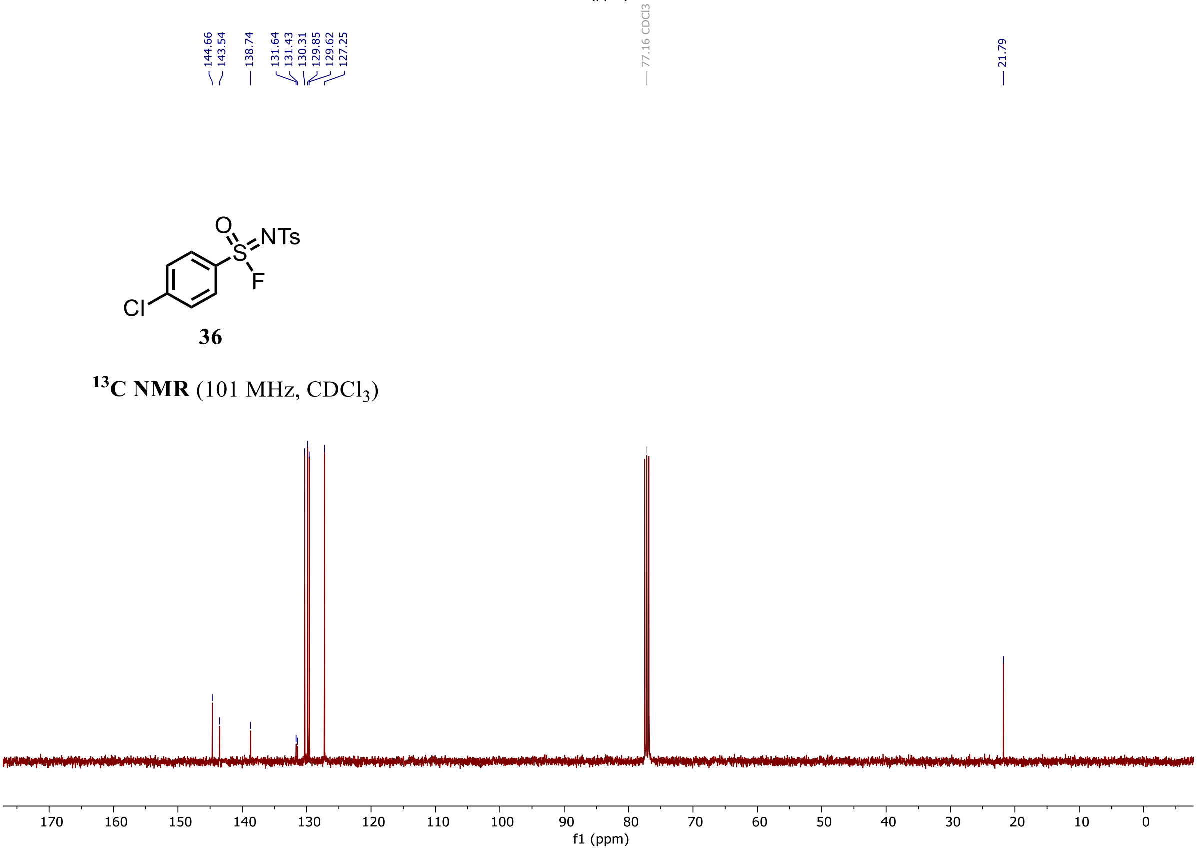

36

^{13}C NMR (101 MHz, $CDCl_3$)

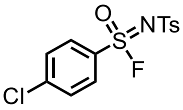

36

^{19}F NMR (377 MHz, $CDCl_3$)

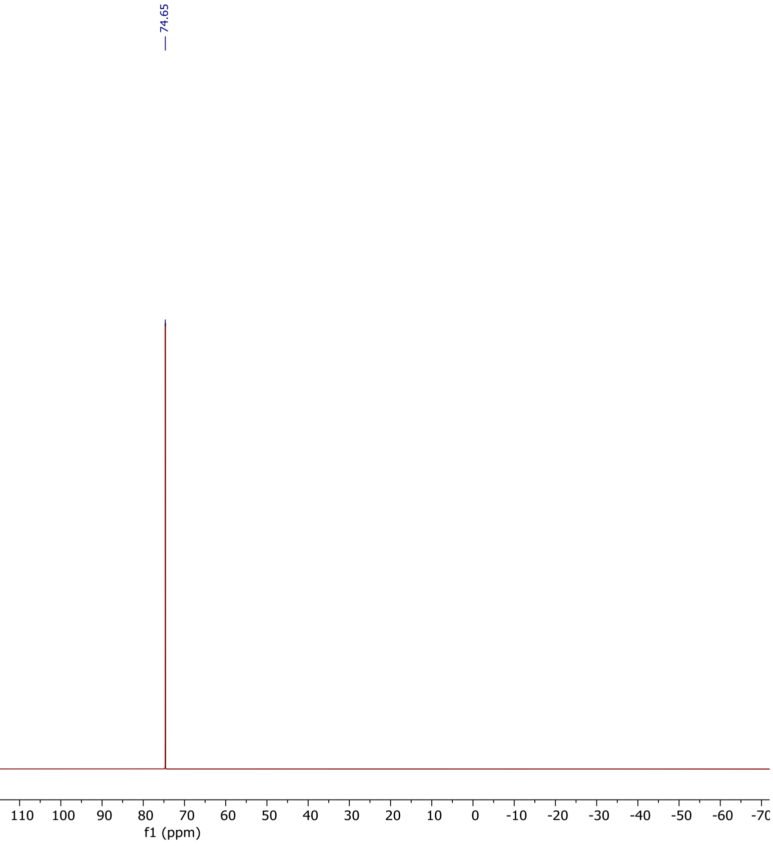

Supplement: Supplementary file 1 — This file contains Supplementary Sections 1–15, including Supplementary Figs. 1–81 and Supplementary Tables 1–23 – see contents for details. [file 41586_2025_8698_MOESM1_ESM.pdf]
